# Organocatalytic cycloaddition of alkynylindoles with azonaphthalenes for atroposelective construction of indole-based biaryls

**DOI:** 10.1038/s41467-022-28211-0

**Published:** 2022-02-02

**Authors:** Hui Yang, Huai-Ri Sun, Rui-Qing He, Le Yu, Wei Hu, Jie Chen, Sen Yang, Gong-Gu Zhang, Ling Zhou

**Affiliations:** Key Laboratory of Synthetic and Natural Functional Molecule of the Ministry of Education, College of Chemistry & Materials Science, National Demonstration Center for Experimental Chemistry Education, Northwest University, Xi’an, 710127 P. R. China

**Keywords:** Organocatalysis, Synthetic chemistry methodology, Organic chemistry

## Abstract

The axially chiral indole-aryl motifs are present in natural products and biologically active compounds as well as in chiral ligands. Atroposelective indole formation is an efficient method to construct indole-based biaryls. We report herein the result of a chiral phosphoric acid catalyzed asymmetric cycloaddition of 3-alkynylindoles with azonaphthalenes. A class of indole-based biaryls were prepared efficiently with excellent yields and enantioselectivities (up to 98% yield, 99% ee). Control experiment and DFT calculations illustrate a possible mechanism in which the reaction proceeds via a dearomatization of indole to generate an allene-iminium intermediate, followed by an intramolecular aza-Michael addition. This approach provides a convergent synthetic strategy for enantioselective construction of axially chiral heterobiaryl backbones.

## Abstract (web summary)

There is great interest in methods for catalytic enantioselective construction of axially chiral compounds found in natural products. Here, the authors develop a cycloaddition strategy for atroposelective construction of indole-based biaryls via chiral phosphoric acid-catalysed cycloaddition.

## Introduction

Axially chiral compounds are present in a plethora of natural products, biologically active compounds, and privileged chiral ligands and catalysts^1–3^. Consequently, developing protocols for the catalytic enantioselective construction of axially chiral scaffolds has attracted a great deal of interest among researchers in synthetic chemistry^4,5^. Many strategies have been developed, including desymmetrization or kinetic resolution of prochiral biaryls^6–12^, aryl–aryl coupling^13–15^, direct arylation^16–22^, chirality conversion^23–33^, cycloaddition^34–39^, and so on^40–48^. Recently, the catalytic enantioselective construction of chiral atropisomeric heterobiaryl backbones has received increasing attention from the chemistry community. However, there are still some challenges to be overcome in this field of research.

**Fig. 1:**
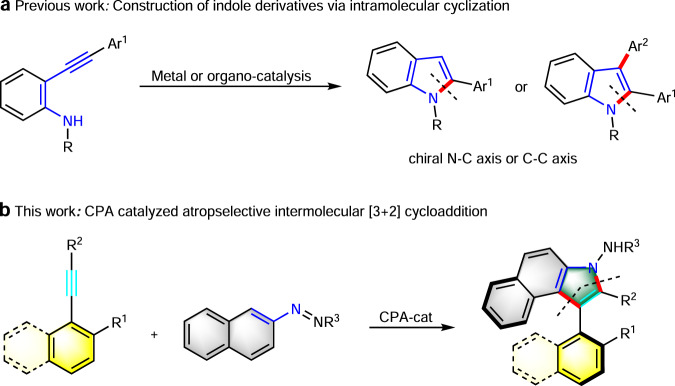
Asymmetric construction of indole-based biaryls via cycloaddition strategies. **a** Construction of indole derivatives via intramolecular cyclization. **b** CPA-catalyzed atroposelective intermolecular [3 + 2] cycloaddition.

Indole-based biaryls have been found in nature and have been used as ligands for asymmetric synthesis^49–51^. Recently, a few outstanding cases of the enantioselective synthesis of indole–aryl skeletons have been reported^22,29,33,52–66^. Generally, there are two strategies to construct indole-based biaryls enantioselectively. One is asymmetric coupling reaction of indole derivatives with other aryls^22,56–62^, the other is in situ construction of indole skeletons atroposelectively^63–66^. Interestingly, *o*-alkynylanilines were usually employed for the synthesis of indoles possessing chiral axis by the latter strategy. For instance, Kitagawa and coworkers reported an elegant Pd(II)-catalyzed enantioselective hydroamination reaction to construct indoles bearing an N-aryl chiral axis^53,55^. Li and coworkers merged C−H activation with nucleophilic cyclization to realize oxidative coupling of indoles and *o*-alkynylanilines for the synthesis of biindolyls^63^. Zhu and coworkers developed a palladium-catalyzed asymmetric Cacchi reaction for the construction of indoles bearing a chiral axis^66^. Yan and coworkers established an organocatalytic enantioselective construction of chiral indole-aryl skeletons through an asymmetric annulation^64^. Notably, the intramolecular annulation strategy, a linear synthesis has been adopted to form a single C–N bond to generate the indole skeleton in all these protocols (Fig. 1a). While the intermolecular strategy, a convergent synthesis strategy for the atroposelective construction of indole–aryls remains limited. Therefore, to realize a divergent synthesis of such indole-based biaryls, designing of more efficient strategies is of great significance.

In fact, transition metal catalyzed cyclization reaction of azobenzene derivatives with alkynes to construct indole derivatives without chiral axis has been reported^67–69^. Although organo-catalyzed cycloaddition of alkenes with azonaphthalenes has been well documented^22,29,33,70^, the organo-catalyzed asymmetric cycloaddition reaction of azo benzene derivatives with alkynes is still lacking. The reason may be ascribed to a relative lower reactivity of alkyne than that of alkene in the cycloaddition reaction with azonaphthalenes. We envisioned that installation of electron-donating groups to alkyne moiety would enhance such reactivity, and further activated by organocatalyst would realize the desired cycloaddition to give the corresponding indole–aryl atropisomers.

In order to achieve this goal, we needed to overcome the following issues: (1) realization of efficient annulation of alkynes with azonaphthalenes; (2) the installation of a suitable group onto the 1-ethynylnaphthene to avoid free rotation around the C–C axis; (3) the choice of a competent chiral catalyst to activate reaction partners and to control the stereoselectivity. Herein, we report a formal [3 + 2] cycloaddition strategy for atroposelective construction of indole-based biaryls via chiral phosphoric acid (CPA)-catalyzed cycloaddition reaction of 3-alkynylindoles with azonaphthalenes (Fig. 1b).

## Results

We commenced the reaction of 1-ethynylnaphthol with azonaphthalene to examine the possibility of our strategy. Unfortunately, no reaction occurred when a series of reaction conditions were attempted (for details, see Supplementary Table 1). The reason may be due to that the electron density of the alkyne was still insufficient to perform the nucleophilic addition to azonaphthalenes. Then we changed the naphthols with more electron-rich indoles, 3-alkynylindol derivatives as the substrate. To our delight, the desired cycloaddition products could be obtained smoothly.

As shown in Table 1, chiral phosphoric acid **16a** (10 mol%) catalyzed the reaction of 3-alkynylindole **1a** with azonaphthalene **8a** in toluene at −50 ^o^C for 72 h, the desired cycloaddition product **9a** was obtained in moderate yield and enantioselectivity (entry 1). An increase in enantioselectivity was realized when catalyst **16b** was used (entry 2). The CPA **16c**, bearing a 9-phenanthryl group at the 6,6′-position of SPINOL, provided the desired product **9a** with good yield and enantioselectivity (87%, 86% ee, entry 3). Having realized the efficiency of the annulation, we turned our effort to improve the enantioselectivity. First, the installation of suitable protection groups onto the naphthol of 3-alkynylindole derivatives was performed to avoid free rotation around the C–C axis during the reaction. As expected, the enantioselectivity declined to around 40% when methoxymethyl was substituted with a less bulky methyl group (entry 4). On the other hand, the more bulky group benzyl or *tert*-Bu has an enhancing effect on the enantioselectivity, albeit with the reaction efficiency was dramatically decreased, probably due to the steric reason (entries 5,6 vs entry 4). These results demonstrated that the R group plays an important role in the stereocontrol in this reaction. The use of substrate **5a** containing a cyclohexyl group could afford the corresponding product in 65% yield with 87% ee (entry 7). Interestingly, *iso*-Pr returned the desired product in 95% yield with 90% ee (entry 8). Moreover, **7a** bearing a 3-pentyl group was found to be the optimal substrate, affording the desired product **15a** in good yield and excellent enantioselectivity (75%, 94% ee, entry 9). Further optimization of the reaction by a variety of solvents (CH_2_Cl_2_, CH_3_CN, CCl_4_, and CHCl_3_) was subsequently investigated (entries 10–13), and CHCl_3_ gave the best result (97% ee, entry 13). In addition, a further decrease of the reaction temperature could not improve the enantioselectivity of the reaction (entry 14). Meanwhile, increasing the temperature to −25 ^o^C or 0 ^o^C has a negative effect on the reaction transformation (entries 15–16). Fortunately, when the catalyst loading was reduced to 5 mol% or 2 mol%, the enantioselectivity almost remained unchanged and excellent yields were still obtained (entries 17–18). Further reducing the amount of the catalyst to 1 mol% resulted in a measurable decrease in the yield (entry 19). The thermal racemization of these atropisomers was conducted to investigate the configurational stability (for details, see Supplementary Information). Compound **15a** displays an excellent stability with a barrier to enantiomerization of 173 kJ mol^−1^, and the half-life time of racemization was determined to be 3.3 × 10^9^ years at 25 ^o^C.

**Table 1:** Optimization of the reaction conditions^a^.


entry	R	cat (mol%)	solvent	T (°C)	time (h)	yield (%)^b^	ee (%)^c^
1	MOM	**16a** (10)	toluene	−50	72	45	57
2	MOM	**16b** (10)	toluene	−50	72	80	76
3	MOM	**16c** (10)	toluene	−50	72	87	86
4	Me	**16c** (10)	toluene	−50	72	91	45
5	Bn	**16c** (10)	toluene	−50	72	41	75
6	*t*Bu	**16c** (10)	toluene	−50	72	46	72
7	Cy	**16c** (10)	toluene	−50	72	65	87
8	*i*Pr	**16c** (10)	toluene	−50	72	95	90
9	3-Pen	**16c** (10)	toluene	−50	72	75	94
10	3-Pen	**16c** (10)	CH_2_Cl_2_	−50	76	97	96
11	3-Pen	**16c** (10)	CH_3_CN	−25	76	81	86
12	3-Pen	**16c** (10)	CCl_4_	−25	76	90	91
13	3-Pen	**16c** (10)	CHCl_3_	−50	76	97	98
14	3-Pen	**16c** (10)	CHCl_3_	−60	80	97	98
15	3-Pen	**16c** (10)	CHCl_3_	−25	85	98	96
16	3-Pen	**16c** (10)	CHCl_3_	0	85	96	95
17	3-Pen	**16c** (5)	CHCl_3_	−50	82	97	97
18	3-Pen	**16c** (2)	CHCl_3_	−50	82	96	97
19	3-Pen	**16c** (1)	CHCl_3_	−50	82	80	96

^a^Reactions were carried out with **1**-**7a** (0.24 mmol), **8a** (0.2 mmol), catalyst (0.02 mmol) in solvent (2.0 mL) under N_2_.

^b^Isolated yield.

^c^Determined by HPLC analysis.

**Fig. 2:**
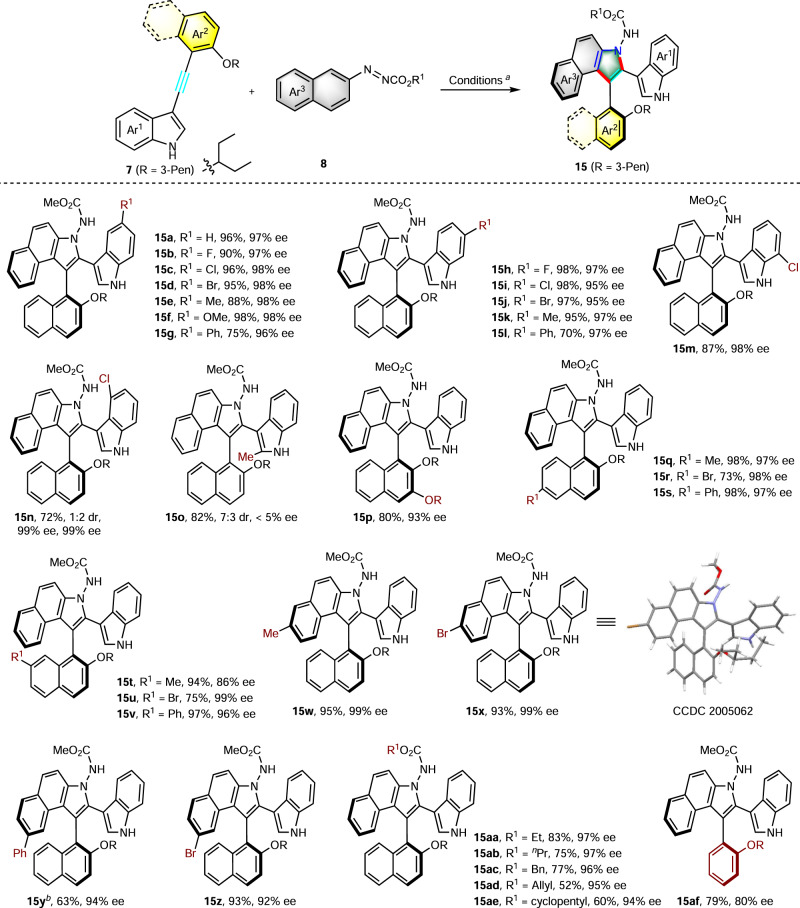
Substrate scope. ^a^Reactions were carried out with **7** (0.24 mmol), **8** (0.20 mmol), and **16c** (0.004 mmol) in CHCl_3_ (2.0 mL) at −50 °C under N_2_. The yields shown are for isolated products and the ee values were determined by HPLC analysis. ^b^The reaction temperature is 40 °C.

With the optimal reaction conditions in hand, we examined the substrate scope of the protocol (Fig. 2). First, the substitution effect at the indole was examined. Substituents of the phenyl ring of the indole core with halogens at the 5-position, such as F, Cl, and Br, afforded the corresponding axially chiral products with excellent yields and enantioselectivities (**15b–d**, 90–96%, 97–98% ee). The methyl or methoxy-group substituent at the 5-position of the indole could be well tolerated to give the expected products **15e** and **15f**, respectively, with excellent results (up to 98% yield, 98% ee). In addition, product **15g** with a 5-Ph substituent indole was obtained in 75% yield and 96% ee. The halogen (F, Cl, and Br)-containing substrates at the 6-position of the indole were tolerated under the standard conditions, all proceeding in excellent yields and enantioselectivities (**15h–j**, 97–98%, 95–97% ee). Substrates with the substituents CH_3_ and Ph at the 6-position of the indole all resulted in the corresponding desired products with good yields and excellent enantioselectivities (**15k**, **15l**, 97% ee). A 7-chloro-substituted indole substrate **7m** returned an 87% yield with 98% ee. Interestingly, when chloro substituent was installed at the 4-position of the indole, the corresponding product **15n** bearing two stereogenic axes was obtained in good yield with excellent enantioselectivity and moderate diastereoselectivity. Substrate **7o** was also used to construct the compound containing two stereogenic axes, unfortunately, poor stereoselectivity was observed.

Next, we examined the substrate scope with regard to the naphthyl ring moiety and other azonaphthalenes. 3-Pentan-3-yloxy at the 3-position of the naphthyl ring resulted in the desired product **15p** in 80% yield with 93% ee. Different substituents at the 6-position of the naphthyl ring, such as CH_3_, Br, and Ph all could afford the desired products in good yields and excellent enantioselectivities (**15q–s**, 73–98% yields, 97–98% ee). Moreover, both electron-rich and electron-deficient substituents at the 7-position of the naphthyl ring returned the desired axially chiral compounds in up to 97% yield with up to 99% ee (**15t–v**). On the other hand, the substrates with different substituents at the 6 or 7-position of the azonaphthalene were well tolerated and delivered the corresponding products **15w–z** in high yields (63–95%) and excellent enantioselectivities (92–99% ee). Among them, the product **15y** was obtained in 63% yield and 93% ee at 40 °C. Finally, azonaphthalene with different N-protecting groups could be employed, delivering the corresponding axially chiral products in moderate-to-good yields (52–83%) and excellent enantioselectivities (**15aa–ae**, 94–97% ee). Besides naphthyl-substituted alkynylindole, phenyl-substituted alkynylindole **7af** was also treated under the optimal reaction conditions, satisfyingly, the desired axially chiral product **15af** was obtained in 79% yield and 80% ee. The relatively lower enantioselectivity may be due to its lower configurational stability. Indeed, compound **15af** displays a lower stability with a barrier to enantiomerization of 100 kJ mol^−1^, and the half-life time of racemization was determined to be 4.8 hours at 25 ^o^C (for details, see Supplementary Information). The absolute configuration of the product was assigned on the basis of the X-ray crystallographic structure of **15x**.

**Fig. 3:**
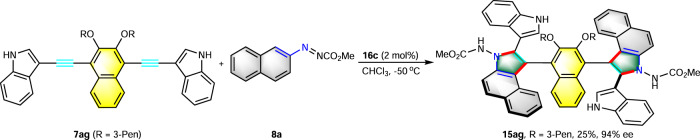
Construction of diaxial compound 15ag. The reaction was carried out with **7ag** (0.05 mmol), **8a** (0.125 mmol), and **16c** (0.002 mmol) in CHCl_3_ (0.5 mL) at −50 °C under N_2_.

**Fig. 4:**
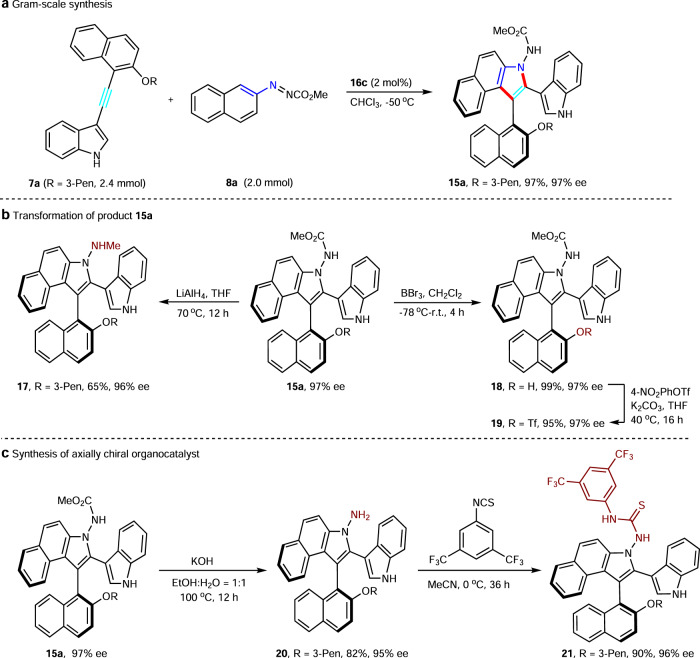
Gram-scale synthesis and transformations. **a** Gram-scale synthesis of axially chiral compound **15a**. **b** Transformation of compound **15a**. **c** Synthesis of axially chiral organocatalyst.

Interestingly, compound **15ag** bearing two chiral axes can be generated with 94% ee by this strategy starting from **7ag**, albeit a lower yield (Fig. 3). To test the practicality of this method, a gram-scale reaction of **7a** with **8a** was carried out under the standard reaction conditions. As expected, compound **15a** was obtained successfully with excellent yield and enantioselectivity (Fig. 4a). In addition, reduction of **15a** with LiAlH_4_ provided the secondary amine product **17** in good yield and no changes occurred in the stereoselectivity during this reaction. The 3-pentyl group could also be removed smoothly by using BBr_3_ in CH_2_Cl_2_ for 4 h, giving phenol product **18** in quantitative yield without any loss of enantiopurity, which is a useful building block for further transformation. Trifluoromethylation of **18** afforded product **19** in excellent yield and enantioselectivity (95% yield, 97% ee), which is a potential partner in metal-catalyzed coupling reactions (Fig. 4b). The protecting group on the azonitrogen center could be removed, affording compound **20** in good yield. The axially chiral benzoindolenaphthyl-derived thiourea compound **21** bearing 3,5-bis(trifluoromethyl)phenyl group was prepared smoothly in 90% yield according to the classical approach for the preparation of cinchona-derived thiourea catalyst (Fig. 4c).

**Fig. 5:**
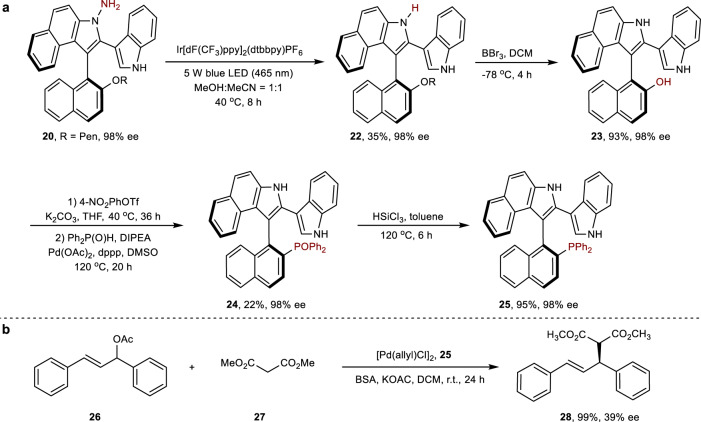
Synthesis and application of chiral phosphine ligand. **a** Synthesis of chiral phosphine ligand **25**. **b** Asymmetric allylation using **25** as the ligand.

On the basis of the above transformation, we attempted to transform compound **15a** into potential useful phosphine ligands to showcase the practical value of the products. As shown in Fig. 5, treatment of **20** with [Ir[dF(CF_3_)ppy]_2_(dtbbpy)PF_6_] catalyst under 5w blue LED lamp, in CH_3_OH/CH_3_CN provided **22**. Compound **24** was prepared from **22**, and the enantioselectivity was retained even after three steps (98% ee). Finally, compound **24** was transformed into the benzoindolenaphthyl phosphine ligand **25** by reduction with HSiCl_3_ reagent. A preliminary application of **25** (98% ee) as ligand in the asymmetric allylation reaction of (*E*)-1,3-diphenylallyl acetate with dimethyl malonate revealed that **25** is efficient at promoting the reaction (99% yield), although the enantioselectivity of product **28** needs to be improved (39% ee).

To get mechanistic insights into this reaction, a control experiment was carried out under the optimal conditions. The desired product could not be obtained when the N–H group of **7a** was protected with methyl, which demonstrated that the N–H group plays an important role in the process. To better understand the mechanism and origins of stereo- and regioselectivity, density-functional theory (DFT) calculations were conducted using Gaussian 09 program. All the intermediates and transition states were optimized by employing the xc-functional ωB97X-D with dispersion correction (ωB97X-D) and the 6-31G(d) basis set. Normal vibrational mode analysis at the same level of theory confirmed that the optimized structures are minima (zero imaginary frequency) or saddle points (one imaginary frequency). Based on optimized geometries, single-point energies and solvent effects in chloroform were computed with the range-separated functional ωB97X-D and the 6-311G (d,p) basis set using the SMD model. To save computational time, the phenanthryl moieties in the catalyst were replaced with methyls and 3-pentyl in the substrate with methyl. The free-energy profiles (in kcal/mol) of the most favorable pathway for the phosphoric acid-catalyzed cycloaddition of alkynylindoles with azonaphthalenes are shown in Supplementary Fig. 2. First, the chiral phosphoric acid acts as a bifunctional catalyst to simultaneously activate 3-alkynylindoles and azosubstrates through a dual hydrogen-bonding activation mode to generate a complex. Subsequent dearomatization of 3-substituted indole generates the transient allene-iminium intermediate^62,71–74^, which further undergoes an intramolecular aza-Michael addition to afford the axially chiral intermediate. Finally, deprotonation and aromatization yields the desired product.

**Fig. 6:**
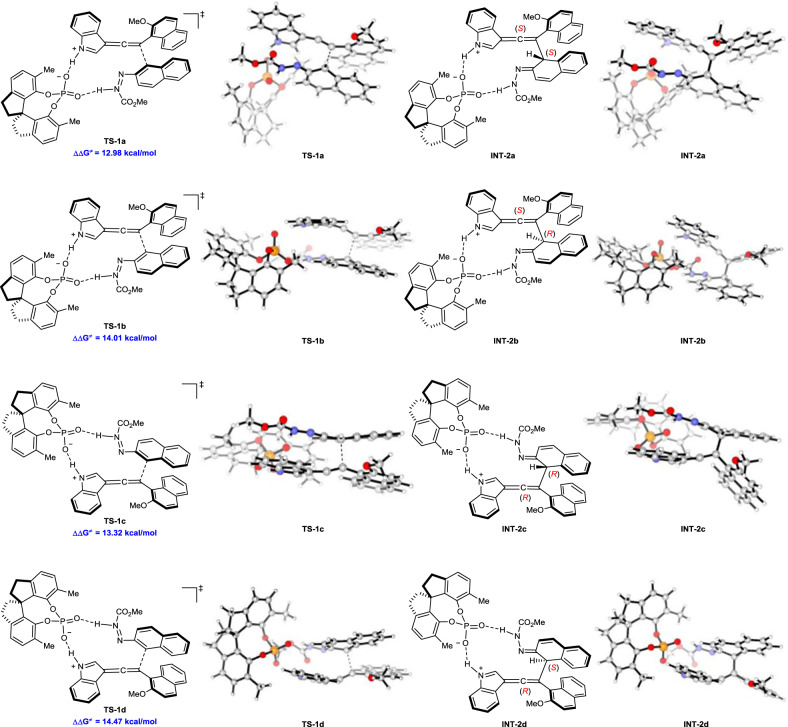
DFT calculations. DFT-optimized chiral phosphoric acid-catalyzed transition states **TS-1a–d**, and their corresponding allene–iminium intermediates **INT-2a–d**.

Figure 6 shows the optimized structures and relative free energies of the four competing stereoselectivity-determining allene–iminium transition states **TS-1a**, **TS-1b**, **TS-1c**, and **TS-1d**, and the structures of their corresponding allene–iminium intermediates. Indeed, the calculated *re*-face attack **TS**-**1a** is found to be 3.7 kcal/mol lower in the relative free energy than the *si*-face attack in **TS**-**1b**. The potential energy of the allene–iminium intermediate **INT-2a** was also found to be lower than that of the intermediate **INT-2b** by 0.89 kcal/mol (for details, see Supplementary Information). Transition states **TS**-**1a** and **TS**-**1c** would generate *S*- and *R*-configuration product, respectively. The relative activation-free energy (ΔΔG) for **TS**-**1a** is 12.98 kcal/mol more favorable than **TS**-**1c**, which corroborated the observed enantioselectivity that the (*S*)-selective product was obtained. In addition, the **TS-1a** that ultimately leads to the major conformation product is 6.22 kcal/mol more stable than **TS-1d**, which is in good agreement with the experimental result.

## Discussion

In this work, we show the CPA-catalyzed intermolecular [3 + 2] formal cycloaddition of 3-alkynylindoles with azonaphthalene for the enantioselective construction of indole-based atropisomers. A type of axially chiral arylindole derivatives were afforded in high yields and excellent stereoselectivities (up to 98% yield, 99% ee). In addition, this transformation allows lowering of the catalyst loading to 2 mol% without considerable loss of reactivity and enantioselectivity. This reaction not only provides a useful method for constructing enantioenriched indole-based biaryl atropisomer scaffolds, but also advances the chemistry of catalytic asymmetric reactions of alkynylindoles.

## Methods

### General procedure for the enantioselective synthesis of 15

To a stirred solution of **7** (0.24 mmol), **8** (0.20 mmol) and CPA**16c** (0.004 mmol) in CHCl_3_ (2.0 mL) at −50 °C in one portion, the mixture was stirred until TLC revealed the absence of the starting material. The solvent was removed under reduced pressure, the residue was purified by flash-column chromatography (petroleum ether/EtOAc) to yield the corresponding product **15**.

### Synthesis of 18

To a mixture of **15a** (113 mg, 0.2 mmol, 1.0 eq) in CH_2_Cl_2_ (2 mL) at −78 °C was added BBr_3_ (0.2 mL, 1.0 mol/L CH_2_Cl_2_, 1.0 eq) dropwise, and the mixture was stirred for 4 hours at room temperature. TLC showed complete consumption of the starting material. The reaction was quenched with 10% NaHCO_3_ solution and extracted with CH_2_Cl_2_ (3 × 5 mL). The combined organics were washed with brine and dried over Na_2_SO_4_. The solvent was removed under reduced pressure, the residue was purified by column chromatography on silica gel (20% EtOAc in hexane) to yield **18** as a white solid (98.4 mg, 99% yield).

### Synthesis of 19

The reaction was performed using **18** (98.4 mg, 0.2 mmol, 1.0 eq), 4-nitrophenyl trifluoromethanesulfonate (65 mg, 0.24 mmol, 1.2 eq), and K_2_CO_3_ (41.5 mg. 0.3 mmol, 1.5 eq) in THF (2.0 mL) at 40 ^o^C. After finishing, the reaction was quenched with H_2_O (5 mL). The solution was extracted with CH_2_Cl_2_ (3 × 5 mL), washed with brine (5.0 mL), and dried over anhydrous Na_2_SO_4_. The solvent was evaporated under reduced pressure. The crude product was purified by flash-column chromatography (10% EtOAc in hexane) giving **19** as a white solid (119.5 mg, 95% yield).

## Supplementary information

Supplementary Information

## Data Availability

The X-ray crystallographic coordinates for structures that support the findings of this study have been deposited at the Cambridge Crystallographic Data Centre (CCDC) with the accession code CCDC 2005065 (15x) (www.ccdc.cam.ac.uk/data_request/cif). The authors declare that all other data supporting the findings of this study are available within the article and Supplementary Information files, and also are available from the corresponding author upon request.
