# Supplementary material for: Organocatalytic cycloaddition of alkynylindoles with azonaphthalenes for atroposelective construction of indole-based biaryls
Source: Nat Commun. 2022 Feb 2;13:632. doi: 10.1038/s41467-022-28211-0 (PMC8810779; doi:10.1038/s41467-022-28211-0)
Supplement: Supplementary file 1 — Supplementary Information [file 41467_2022_28211_MOESM1_ESM.pdf]

Supplementary Information

Organocatalytic Cycloaddition of Alkynylindoles with Azonaphthalenes for Atroposelective Construction of Indole-Based Biaryls

Hui Yang[1,2], Huai-Ri Sun[1,2], Rui-Qing He[1], Le Yu[1], Wei Hu[1], Jie Chen[1], Sen Yang[1], Gong-Gu Zhang[1] and Ling Zhou[1]✉

[1]Key Laboratory of Synthetic and Natural Functional Molecule of the Ministry of Education, College of Chemistry & Materials Science, National Demonstration Center for Experimental Chemistry Education, Northwest University, Xi'an 710127, P. R. China. [2]These authors contributed equally: Hui, Yang, Huai-Ri Sun. ✉email: zhoul@nwu.edu.cn

Table of Contents

**S2**	**General information**
**S2-S104**	**Experimental procedures and physical data**
**S105-S172**	**^{1}H and ^{13}C NMR spectra**
**S173-S174**	**CD spectra**
**S175**	**Figure 1 X-ray structure of 15x**
**S176-S232**	**Computational Studies**
**S233**	**References**

General information

All reactions that required anhydrous conditions were carried by standard procedures under nitrogen atmosphere. Commercially available reagents were used as received. The solvents were dried by distillation over the appropriate drying reagents. Infrared spectra were recorded on a TENSOR 27 FT-IR spectrophotometer and reported in wave numbers (cm^{-1}). ^{1}H NMR and ^{13}C NMR spectra were recorded on Bruker (400 MHz, 600 MHz) spectrometer. Chemical shifts (δ) are reported in ppm relative to TMS (δ 0.00) for the ^{1}H NMR and to chloroform (δ 77.0) for the ^{13}C NMR measurements. Reactions were followed with TLC (0.254mm silica gel 60-F plates). Visualization was accomplished with UV light. Flash chromatography separations were performed on 200-300 mesh silica gel.

Experimental procedures and physical data

(1) Compounds 8[1-3] were synthesized according to literature known procedures.

(2) General procedure for the preparation of substrates 7a -7l, 7n-7s, and 7ab [4-7]:

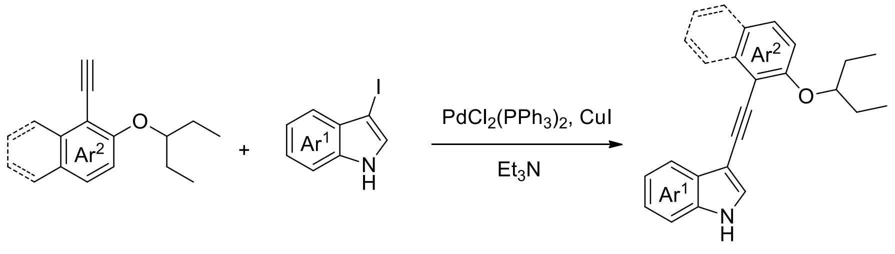

A solution of 3-iodo-1H-indole (3.0 mmol, 1.0 eq), $PdCl_2(PPh_3)_2$ (0.06mmol, 0.02 eq) and CuI (0.09 mmol, 0.03 eq) in Et_3N (5.0 mL) was stirred at room temperature for 0.5 h at under N_2. Subsequently, 1-ethynyl-2-(pentan-3-yloxy)naphthalene (3.6 mmol, 1.2 eq) was added. The reaction was stirred at room temperature for 12 h. After removal of the solvent under reduced pressure, the residue was purified by column chromatography on silica gel (30% CH_2Cl_2 in hexane) gave **7**.

Table 1. Optimization of the [3+2] Formal Cycloaddition Reaction Conditions [a].

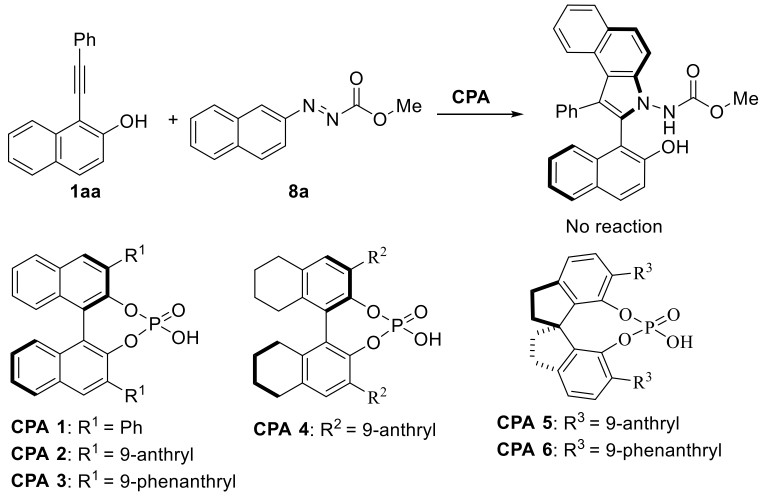

Entry	Cat	Solvent	T (°C)	Time (h)	Yield (%)
1	**CPA 1**	CH_2Cl_2	r.t.	72	NR
2	**CPA 2**	CH_2Cl_2	r.t.	72	NR
3	**CPA 3**	CH_2Cl_2	r.t.	72	NR
4	**CPA 4**	CH_2Cl_2	r.t.	72	NR
5	**CPA 5**	CH_2Cl_2	r.t.	72	NR
6	**CPA 6**	CH_2Cl_2	r.t.	72	NR
7	**CPA 1**	CH_2Cl_2	40	72	NR
8	**CPA 1**	$CHCl_3$	r.t.	72	NR
9	**CPA 1**	toluene	r.t.	72	NR
10	**CPA 1**	CH_3CN	r.t.	72	NR

[a] Reactions were carried out with **1aa** (0.24 mmol), **8a** (0.20 mmol), **CPA** (0.02 mmol) in solvent (2.0 mL) under N_2.

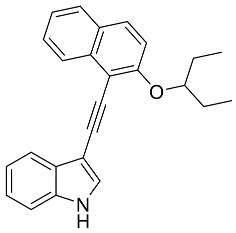

3-((2-(Pentan-3-yloxy)naphthalen-1-yl)ethynyl)-1H-indole (7a)

Yellow solid, mp 146.0-147.0 ℃; IR (KBr): 3421, 2928, 1586, 1459, 809 cm^{-1}; ^1H NMR (400 MHz, $CDCl_3$) δ 8.47 (d, *J* = 8.3 Hz, 1H), 8.26 (s, 1H), 7.95 (s, 1H), 7.76 (dd, *J* = 13.5, 8.7 Hz, 2H), 7.55 (t, *J* = 7.4 Hz, 1H), 7.46 – 7.29 (m, 4H), 7.24 (d, *J* = 6.2 Hz, 2H), 4.57 – 4.19 (m, 1H), 1.81 (dq, *J* = 13.7, 6.9 Hz, 4H), 1.05 (t, *J* = 7.3 Hz, 6H); ^{13}C NMR (100 MHz, $CDCl_3$) δ 157.8, 135.2, 134.4, 128.9, 128.8, 128.6, 128.0, 127.5, 126.9, 125.6, 124.2, 123.0, 120.6, 120.1, 117.0, 111.4, 109.7, 99.6, 92.2, 85.9, 82.9, 26.4, 9.6; HRMS (ESI) calcd for $C_{25}H_{23}NONa$ *m/z* $[M + Na]^+$: 376.1672; found: 376.1675.

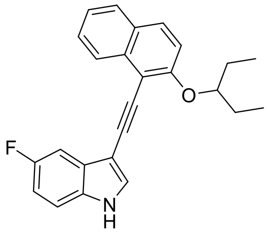

5-Fluoro-3-((2-(pentan-3-yloxy)naphthalen-1-yl)ethynyl)-1H-indole (7b)

Yellow solid, mp 139.0-140.0 ℃; IR (KBr): 3416, 2927, 1585, 1458, 1271, 806 cm^{-1}; ^1H NMR (400 MHz, $CDCl_3$) δ 8.43 (d, *J* = 8.4 Hz, 1H), 8.34 (s, 1H), 7.87 – 7.70 (m, 2H), 7.61 – 7.54 (m, 2H), 7.52 (s, 1H), 7.39 (t, *J* = 7.5 Hz, 1H), 7.26 (d, *J* = 9.1 Hz, 2H), 7.00 (t, *J* = 8.8 Hz, 1H), 4.54 – 4.26 (m, 1H), 2.02 – 1.72 (m, *J* = 7.1 Hz, 4H), 1.07 (t, *J* = 7.4 Hz, 6H); ^{13}C NMR (100 MHz, $CDCl_3$) δ 158.5 (d, *J* = 236.5 Hz), 157.9, 134.3, 131.7, 129.3 (d, *J* = 10.2 Hz), 129.1, 128.9, 128.7, 128.0, 127.0, 125.4, 124.2, 116.7, 112.1 (d, *J* = 9.6 Hz), 111.5 (d, *J* = 26.5 Hz), 109.2, 105.2 (d, *J* = 24.0 Hz), 100.0, 91.4, 86.3, 82.8, 26.4, 9.7; HRMS (ESI) calcd for $C_{25}H_{22}FNONa$ *m/z* $[M + Na]^+$: 394.1578; found: 394.1579.

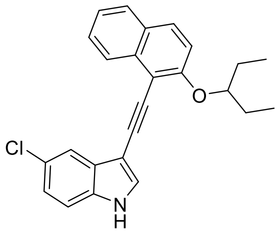

5-Chloro-3-((2-(pentan-3-yloxy)naphthalen-1-yl)ethynyl)-1H-indole (7c)

Yellow solid, mp 146.0-147.0 ℃; IR (KBr): 3411, 2921, 2198, 1583, 1458, 1240, 800 cm^{-1}; ^1H NMR (400 MHz, $CDCl_3$) δ 8.42 (d, *J* = 8.4 Hz, 1H), 8.30 (s, 1H), 7.92 (d, *J* = 1.9 Hz, 1H), 7.77 (t, *J* = 9.0 Hz, 2H), 7.56 (t, *J* = 8.2 Hz, 1H), 7.46 (d, *J* = 2.6 Hz, 1H), 7.39 (t, *J* = 8.0 Hz, 1H), 7.26 (d, *J* = 3.0 Hz, 1H), 7.25 – 7.23 (m, 1H), 7.19 (dd, *J* = 8.6, 2.0 Hz, 1H), 4.42 (p, *J* = 5.8 Hz, 1H), 1.98 – 1.69 (m, 4H), 1.07 (t, *J* = 7.4 Hz, 6H); ^{13}C NMR (100 MHz, $CDCl_3$) δ 158.0, 134.3, 133.6, 129.8, 129.2, 128.7, 128.3, 128.0, 127.0, 126.6, 125.4, 124.2, 123.4, 119.7, 116.6, 112.4, 109.0, 99.6, 91.2, 86.5, 82.7, 26.4, 9.7; HRMS (ESI) calcd for $C_{25}H_{22}ClNONa$ *m/z* $[M + Na]^+$: 410.1282; found: 410.1287.

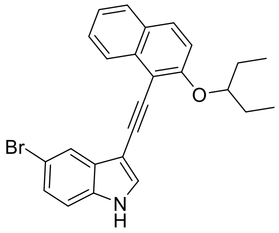

5-Bromo-3-((2-(pentan-3-yloxy)naphthalen-1-yl)ethynyl)-1H-indole (7d)

Yellow solid, mp 155.0-156.0 ℃; IR (KBr): 3382, 2360, 1550, 848, 634 cm^{-1}; ^1H NMR (400 MHz, $CDCl_3$) δ 8.45 (d, *J* = 8.4 Hz, 1H), 8.33 (s, 1H), 8.12 (d, *J* = 1.8 Hz, 1H), 7.81 (t, *J* = 8.6 Hz, 2H), 7.60 (ddd, *J* = 8.2, 6.9, 1.2 Hz, 1H), 7.53 (d, *J* = 2.6 Hz, 1H), 7.43 (ddd, *J* = 8.0, 6.9, 1.1 Hz, 1H), 7.38 (dd, *J* = 8.6, 1.9 Hz, 1H), 7.30 (d, *J* = 4.4 Hz, 1H), 7.28 (d, *J* = 3.6 Hz, 1H), 4.58 – 4.24 (m, 1H), 1.89 (dq, *J* = 15.7, 7.9 Hz, 4H), 1.12 (t, *J* = 7.4 Hz, 6H); ^{13}C NMR (100 MHz, $CDCl_3$) δ 158.0, 134.3, 133.9, 130.4, 129.2, 128.7, 128.1, 128.0, 127.0, 126.0, 125.5, 124.2, 122.9, 116.5, 114.1, 112.8, 109.0, 99.7, 91.1, 86.5, 82.7, 26.5, 9.7; HRMS (ESI) calcd for $C_{25}H_{22}BrNONa$ *m/z* $[M + Na]^+$: 454.0777; found: 454.0779.

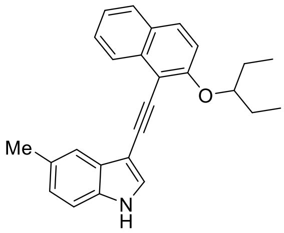

5-Methyl-3-((2-(pentan-3-yloxy)naphthalen-1-yl)ethynyl)-1H-indole (7e)

Yellow solid, mp 141.0-142.0 ℃; IR (KBr): 3406, 2920, 2118, 1456, 1242 cm^{-1}; ^{1}H NMR (400 MHz, $CDCl_3$) δ 8.47 (d, *J* = 8.4 Hz, 1H), 8.13 (s, 1H), 7.77 (d, *J* = 8.1 Hz, 1H), 7.76 – 7.71 (m, 2H), 7.55 (t, *J* = 7.6 Hz, 1H), 7.41 (d, *J* = 2.7 Hz, 1H), 7.37 (d, *J* = 7.9 Hz, 1H), 7.29 – 7.16 (m, 2H), 7.06 (d, *J* = 9.4 Hz, 1H), 4.42 (p, *J* = 5.7 Hz, 1H), 2.49 (s, 3H), 1.82 (dh, *J* = 14.1, 6.8 Hz, 4H), 1.06 (t, *J* = 7.4 Hz, 6H); ^{13}C NMR (100 MHz, $CDCl_3$) δ 157.7, 134.4, 133.5, 130.0, 128.9, 128.8, 127.9, 127.5, 126.9, 125.6, 124.6, 124.1, 119.8, 116.9, 111.0, 109.7, 99.1, 92.5, 85.7, 82.8, 26.4, 21.4, 9.7; HRMS (ESI) calcd for $C_{26}H_{25}NONa$ *m/z* $[M + Na]^+$: 390.1828; found: 390.1832.

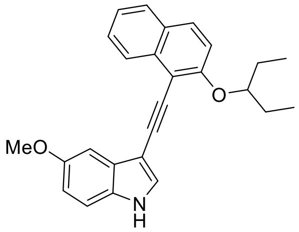

5-Methoxy-3-((2-(pentan-3-yloxy)naphthalen-1-yl)ethynyl)-1H-indole (7f)

Yellow solid, mp 153.0-156.0 ℃; IR (KBr): 3409, 2923, 1456, 1265, 802 cm^{-1}; ^{1}H NMR (400 MHz, $CDCl_3$) δ 8.48 (d, *J* = 8.4 Hz, 1H), 8.24 (s, 1H), 7.76 (dd, *J* = 13.5, 8.6 Hz, 2H), 7.54 (t, *J* = 7.6 Hz, 1H), 7.45 (s, 1H), 7.42 – 7.34 (m, 2H), 7.29 – 7.15 (m, 2H), 6.90 (dd, *J* = 8.8, 2.4 Hz, 1H), 4.41 (p, *J* = 5.7 Hz, 1H), 3.89 (s, 3H), 1.96 – 1.61 (m, *J* = 6.7 Hz, 4H), 1.05 (t, *J* = 7.4 Hz, 6H); ^{13}C NMR (100 MHz, $CDCl_3$) δ 157.7, 154.9, 134.4, 130.3, 129.1, 128.9, 128.8, 128.2, 128.0, 126.9, 125.6, 124.2, 117.1, 113.5, 112.2, 109.7, 101.4, 99.3, 92.3, 85.8, 82.9, 55.7, 26.4, 9.7; HRMS (ESI) calcd for $C_{26}H_{25}NO_2Na$ *m/z* $[M + Na]^+$: 406.1778; found: 406.1777.

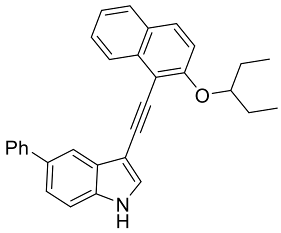

3-((2-(Pentan-3-yloxy)naphthalen-1-yl)ethynyl)-5-phenyl-1H-indole (7g)

Yellow solid, mp 147.0-148.0 ℃; IR (KBr): 3409, 2924, 2200, 1589, 1461, 1267, 752 cm^{-1}; ^{1}H NMR (400 MHz, $CDCl_3$) δ 8.48 (d, *J* = 8.4 Hz, 1H), 8.31 (s, 1H), 8.16 (s, 1H), 7.77 (dd, *J* = 11.8, 8.7 Hz, 2H), 7.70 (d, *J* = 7.4 Hz, 2H), 7.54 (q, *J* = 8.0, 7.5 Hz, 3H), 7.48 – 7.42 (m, 3H), 7.39 (t, *J* = 7.4 Hz, 1H), 7.32 (t, *J* = 7.3 Hz, 1H), 7.25 (d, *J* = 10.0 Hz, 1H), 4.60 – 4.18 (m, 1H), 1.81 (dh, *J* = 14.0, 6.8 Hz, 4H), 1.04 (t, *J* = 7.4 Hz, 6H); ^{13}C NMR (100 MHz, $CDCl_3$) δ 157.9, 142.1, 134.7, 134.4, 134.3, 129.2, 129.0, 128.8, 128.6, 128.0, 128.0, 127.4, 126.9, 126.5, 125.6, 124.2, 122.9, 118.7, 116.9, 111.6, 109.6, 100.3, 91.9, 86.2, 82.8, 29.7, 26.4, 9.7; HRMS (ESI) calcd for $C_{31}H_{27}NONa$ *m/z* $[M + Na]^+$: 452.1985; found: 452.1980.

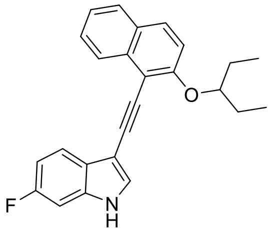

6-Fluoro-3-((2-(pentan-3-yloxy)naphthalen-1-yl)ethynyl)-1H-indole (7h)

Yellow solid, mp 164.0-165.0 ℃; IR (KBr): 3415, 2921, 2200, 1454, 1232, 1134, 802 cm^{-1}; ^{1}H NMR (400 MHz, $CDCl_3$) δ 8.43 (d, *J* = 8.4 Hz, 1H), 8.24 (s, 1H), 7.84 (dd, *J* = 8.6, 5.3 Hz, 1H), 7.77 (t, *J* = 9.3 Hz, 2H), 7.55 (t, *J* = 7.6 Hz, 1H), 7.46 (d, *J* = 2.5 Hz, 1H), 7.39 (t, *J* = 7.5 Hz, 1H), 7.24 (s, 1H), 7.08 – 7.02 (m, 1H), 7.02 – 6.96 (m, 1H), 4.41 (p, *J* = 5.7 Hz, 1H), 1.96 – 1.69 (m, 4H), 1.06 (t, *J* = 7.4 Hz, 6H); ^{13}C NMR (100 MHz, $CDCl_3$) δ 160.5 (d, *J* = 239.0 Hz), 157.9, 135.2 (d, *J* = 12.6 Hz), 134.4, 129.1, 128.8, 128.0, 127.6 (d, *J* = 3.3 Hz), 127.0, 125.5, 125.1, 124.2, 121.0 (d, *J* = 10.2 Hz), 116.9, 109.6, 109.3, 100.0, 97.8 (d, *J* = 26.4 Hz), 91.6, 86.1, 82.9, 26.4, 9.6; HRMS (ESI) calcd for $C_{25}H_{22}FNONa$ *m/z* $[M + Na]^+$: 394.1578; found: 394.1577.

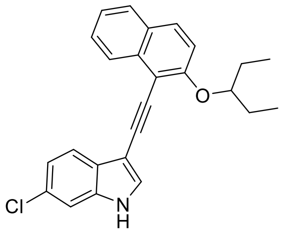

6-Chloro-3-((2-(pentan-3-yloxy)naphthalen-1-yl)ethynyl)-1H-indole (7i)

Yellow solid, mp 148.0-149.0 ℃; IR (KBr): 3413, 2920, 2196, 1620, 1454, 1265, 752 cm^{-1}; ^{1}H NMR (400 MHz, $CDCl_3$) δ 8.42 (d, *J* = 8.4 Hz, 1H), 8.29 (s, 1H), 7.83 (d, *J* = 8.4 Hz, 1H), 7.77 (t, *J* = 9.1 Hz, 2H), 7.56 (t, *J* = 7.6 Hz, 1H), 7.46 (d, *J* = 2.5 Hz, 1H), 7.39 (t, *J* = 7.5 Hz, 1H), 7.34 (d, *J* = 1.5 Hz, 1H), 7.25 (d, *J* = 9.4 Hz, 1H), 7.21 (dd, *J* = 8.4, 1.7 Hz, 1H), 4.41 (p, *J* = 5.8 Hz, 1H), 2.02 – 1.67 (m, *J* = 6.6 Hz, 4H), 1.05 (t, *J* = 7.4 Hz, 6H); ^{13}C NMR (100 MHz, $CDCl_3$) δ 158.0, 135.6, 134.4, 129.3, 129.0, 128.8, 128.1, 128.0, 127.2, 127.1, 125.5, 124.3, 121.5, 121.1, 116.9, 111.4, 109.3, 100.1, 91.4, 86.4, 82.9, 26.5, 9.7; HRMS (ESI) calcd for $C_{25}H_{22}ClNONa$ *m/z* $[M + Na]^{+}$: 410.1282; found: 410.1285.

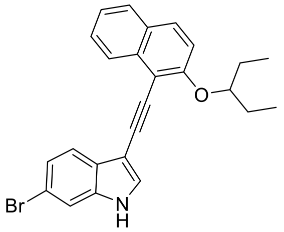

6-Bromo-3-((2-(pentan-3-yloxy)naphthalen-1-yl)ethynyl)-1H-indole (7j)

Yellow solid, mp 163.0-164.0 ℃; IR (KBr): 3411, 2923, 2200, 1456, 1263, 1099, 752 cm^{-1}; ^{1}H NMR (400 MHz, $CDCl_3$) δ 8.42 (d, *J* = 8.3 Hz, 1H), 8.26 (s, 1H), 7.81 – 7.73 (m, 3H), 7.56 (ddd, *J* = 8.3, 6.8, 1.2 Hz, 1H), 7.51 (s, 1H), 7.46 (d, *J* = 2.5 Hz, 1H), 7.39 (ddd, *J* = 8.1, 6.9, 1.2 Hz, 1H), 7.34 (dd, *J* = 8.4, 1.7 Hz, 1H), 7.25 (d, *J* = 8.1 Hz, 1H), 4.41 (p, *J* = 5.8 Hz, 1H), 1.91 – 1.72 (m, 4H), 1.05 (t, *J* = 7.4 Hz, 6H); ^{13}C NMR (100 MHz, $CDCl_3$) δ 158.0, 136.0, 134.4, 129.2, 128.8, 128.0, 127.8, 127.5, 127.0, 125.4, 124.2, 124.0, 121.5, 116.8, 116.6, 114.3, 109.2, 100.2, 91.3, 86.4, 82.9, 26.4, 9.6; HRMS (ESI) calcd for $C_{25}H_{22}BrNONa$ *m/z* $[M + Na]^{+}$: 454.0777; found: 454.0778.

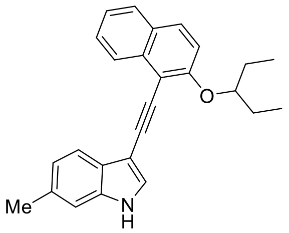

6-Methyl-3-((2-(pentan-3-yloxy)naphthalen-1-yl)ethynyl)-1H-indole (7k)

Yellow solid, mp 150.0-151.0 ℃; IR (KBr): 3429, 2921, 1627, 1265, 754 cm^{-1}; ^{1}H NMR (400 MHz, $CDCl_3$) δ 8.48 (s, 1H), 8.11 (s, 1H), 7.82 (d, *J* = 8.0 Hz, 1H), 7.78 (d, *J* = 8.1 Hz, 1H), 7.74 (d, *J* = 9.0 Hz, 1H), 7.61 – 7.52 (m, 1H), 7.43 (d, *J* = 2.5 Hz, 1H), 7.41 – 7.36 (m, 1H), 7.24 (s, 1H), 7.15 (s, 1H), 7.08 (d, *J* = 8.1 Hz, 1H), 4.42 (p, *J* = 5.7 Hz, 1H), 2.47 (s, 3H), 1.81 (dh, *J* = 13.9, 6.6 Hz, 4H), 1.06 (t, *J* = 7.4 Hz, 6H); ^{13}C NMR (100 MHz, $CDCl_3$) δ 157.77, 135.68, 134.43, 132.91, 128.84, 127.92, 126.86, 126.45, 125.64, 124.16, 122.43, 119.83, 117.12, 111.26, 109.78, 99.57, 92.42, 85.72, 82.91, 77.32, 77.00, 76.68, 26.41, 21.71, 9.64; HRMS (ESI) calcd for $C_{26}H_{25}NONa$ *m/z* [M + Na]$^+$: 390.1828; found: 390.1824.

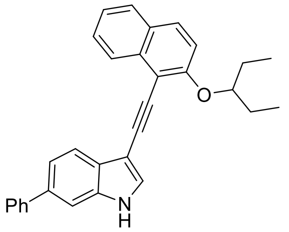

3-((2-(Pentan-3-yloxy)naphthalen-1-yl)ethynyl)-6-phenyl-1H-indole (7l)

Yellow solid, mp 151.0-152.0 ℃; IR (KBr): 3412, 2923, 1586, 1459, 809 cm^{-1}; ^{1}H NMR (400 MHz, $CDCl_3$) δ 8.48 (d, *J* = 8.4 Hz, 1H), 8.33 (s, 1H), 7.99 (d, *J* = 8.2 Hz, 1H), 7.77 (dd, *J* = 12.7, 8.6 Hz, 2H), 7.64 (d, *J* = 7.3 Hz, 2H), 7.56 (q, *J* = 5.6, 3.6 Hz, 2H), 7.53 – 7.49 (m, 2H), 7.44 (t, *J* = 7.7 Hz, 2H), 7.39 (d, *J* = 7.2 Hz, 1H), 7.33 (t, *J* = 7.3 Hz, 1H), 7.24 (d, *J* = 3.4 Hz, 1H), 4.64 – 4.17 (m, 1H), 1.83 (dh, *J* = 13.9, 6.7 Hz, 4H), 1.07 (t, *J* = 7.4 Hz, 6H); ^{13}C NMR (100 MHz, $CDCl_3$) δ 157.9, 141.9, 136.6, 135.8, 134.4, 129.0, 128.8, 128.7, 128.0, 128.0, 127.4, 126.9, 126.8, 125.6, 124.2, 120.6, 120.4, 117.0, 109.9, 109.6, 99.8, 92.1, 86.0, 82.9, 29.7, 26.4, 9.7; HRMS (ESI) calcd for $C_{31}H_{27}NONa$ *m/z* [M + Na]$^+$: 452.1985; found: 452.1987.

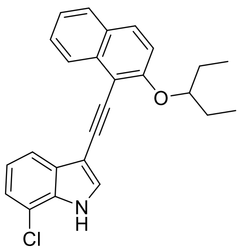

7-Chloro-3-((2-(pentan-3-yloxy)naphthalen-1-yl)ethynyl)-1H-indole (7m)

Yellow solid, mp 115.0-117.0 ℃; IR (KBr): 3423, 2860, 1563, 1243, 748 cm^{-1}; ^{1}H NMR (400 MHz, $CDCl_3$) δ 8.59 – 8.53 (m, 2H), 7.98 (d, *J* = 7.8 Hz, 1H), 7.87 (dd, *J* = 11.1, 8.8 Hz, 2H), 7.68 (t, *J* = 7.5 Hz, 1H), 7.58 (d, *J* = 2.5 Hz, 1H), 7.50 (t, *J* = 7.5 Hz, 1H), 7.38 – 7.32 (m, 2H), 7.28 (dd, *J* = 8.6, 6.8 Hz, 1H), 4.58 – 4.41 (m, 1H), 1.91 (ddd, *J* = 21.6, 14.5, 7.1 Hz, 4H), 1.17 (t, *J* = 7.4 Hz, 6H); ^{13}C NMR (100 MHz, $CDCl_3$) δ 157.9, 134.3, 132.5, 129.9, 129.2, 128.7, 128.0, 127.9, 127.0, 125.4, 124.2, 122.3, 121.3, 118.7, 116.8, 109.1, 100.7, 91.4, 86.3, 82.8, 26.3, 9.6; HRMS (ESI) calcd for $C_{25}H_{22}ClNONa$ *m/z* [M + Na]$^+$: 410.1282; found: 410.1282.

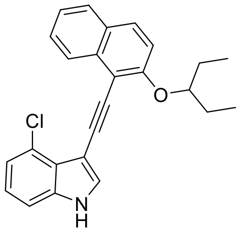

4-Chloro-3-((2-(pentan-3-yloxy)naphthalen-1-yl)ethynyl)-1H-indole (7n)

Yellow solid, mp 143.0-145.0 ℃; IR (KBr): 3423, 2936, 2248, 1610, 1558, 1240 cm^{-1}; ^{1}H NMR (400 MHz, $CDCl_3$) δ 8.66 – 8.57 (m, 2H), 7.82 (dd, *J* = 13.1, 8.5 Hz, 2H), 7.59 (dd, *J* = 8.3, 1 Hz, 1H), 7.50 – 7.41 (m, 2H), 7.33 – 7.27 (m, 1H), 7.24 – 7.16 (m, 2H), 7.12 (t, *J* = 7.8 Hz, 1H), 4.53 – 4.39 (m, 1H), 1.91 – 1.73 (m, 4H), 1.09 (t, *J* = 7.5 Hz, 6H);^{13}C NMR (100 MHz,$CDCl_3$) δ 157.8, 136.4, 134.8, 129.8, 129.0, 128.8, 127.9, 127.0, 126.9, 125.8, 124.6, 124.2, 123.3, 121.3, 117.0, 110.3, 109.7, 98.8, 92.8, 85.9, 83.0, 26.3, 9.6; HRMS (ESI) calcd for $C_{25}H_{22}ClNONa$ *m/z* [M + Na]$^+$: 410.1282; found: 410.1284.

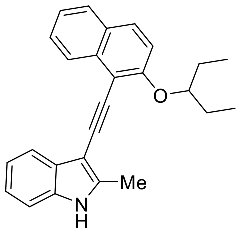

2-Methyl-3-((2-(pentan-3-yloxy)naphthalen-1-yl)ethynyl)-1H-indole (7o)

Yellow solid, mp 153.0-155.0 ℃; IR (KBr): 3329, 2846, 1443, 1276, 756 cm^{-1}; ^{1}H NMR (400 MHz, $CDCl_3$) δ 8.56 (d, *J* = 8.4 Hz, 1H), 8.00 (s, 1H), 7.91 (d, *J* = 7.4 Hz, 1H), 7.82 (dd, *J* = 19.3, 8.6 Hz, 2H), 7.70 – 7.54 (m, 1H), 7.45 (t, *J* = 7.4 Hz,1H), 7.27 (ddd, *J* = 12.4, 10.6, 5.1 Hz, 4H), 4.58 – 4.42 (m, 1H), 2.64 (s, 3H), 1.88 (dd, *J* = 13.5, 6.7 Hz, 4H), 1.11 (t, *J* = 7.4 Hz, 6H); ^{13}C NMR (100 MHz, $CDCl_3$) δ 157.5, 139.3, 134.9, 134.3, 129.3, 128.8, 128.7, 128.1, 127.0, 125.6, 124.2, 122.1, 120.5, 119.5, 116.5, 110.7, 109.7, 97.5, 92.8, 87.7, 82.4, 26.6, 12.9, 9.8; HRMS (ESI) calcd for $C_{26}H_{25}NONa$ *m/z* [M + Na]$^+$: 390.1828; found: 390.1826.

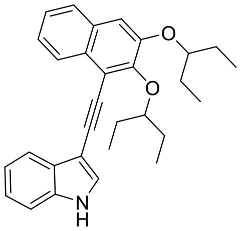

3-((2,3-bis(pentan-3-yloxy)naphthalen-1-yl)ethynyl)-1H-indole(7p)

Yellow solid, mp 146.0-147.0 ℃; IR (KBr): 3427, 2893, 2186, 1483, 1362, 846 cm^{-1}; ^{1}H NMR (400 MHz, $CDCl_3$) δ 8.68–8.50 (m, 2H), 8.09 (dd, *J* = 5.6, 2.2 Hz, 1H), 7.82 (d, *J* = 7.6 Hz, 1H), 7.63–7.51 (m, 3H), 7.46 (d, *J* = 7.9 Hz, 1H), 7.36 (dd, *J* = 9.9, 6.4 Hz, 3H), 4.78–4.64 (m, 1H), 4.64–4.45 (m, 1H), 2.07–1.83 (m, 8H), 1.15 (t, *J* = 7.4 Hz, 12H); ^{13}C NMR (100 MHz, $CDCl_3$) δ 151.0, 150.0, 135.3, 130.6 , 129.0, 128.5 , 127.8, 126.4, 125.7, 125.2, 124.2, 123.0, 120.7, 120.2, 115.5, 111.4, 109.1, 99.5, 92.1, 86.2, 85.3, 80.1, 26.0, 25.7, 9.6; HRMS (ESI) calcd for $C_{30}H_{33}NO_2Na$ *m/z* [M + Na]$^+$: 462.2404; found: 462.2410.

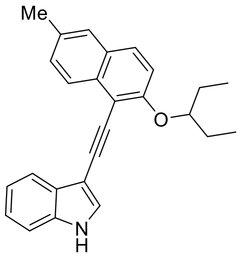

3-((6-Methyl-2-(pentan-3-yloxy)naphthalen-1-yl)ethynyl)-1H-indole (7q)

Yellow solid, mp 126.0-127.0 ℃; IR (KBr): 3405, 2925, 1459, 1249, 752 cm^{-1}; ^{1}H NMR (400 MHz, $CDCl_3$) δ 8.36 (d, *J* = 8.5 Hz, 1H), 8.24 (s, 1H), 7.95 (dd, *J* = 5.5, 2.8 Hz, 1H), 7.66 (d, *J* = 9.0 Hz, 1H), 7.55 (s, 1H), 7.49 (d, *J* = 2.6 Hz, 1H), 7.43 – 7.35 (m, 2H), 7.27 – 7.25 (m, 1H), 7.21 (d, *J* = 9.0 Hz, 2H), 4.39 (p, *J* = 5.7 Hz, 1H), 2.49 (s, 3H), 1.81 (dh, *J* = 14.0, 6.6 Hz, 4H), 1.06 (t, *J* = 7.4 Hz, 6H); ^{13}C NMR (100 MHz, $CDCl_3$) δ 157.2, 135.3, 133.7, 132.6, 129.2, 129.1, 128.6, 128.3, 127.4, 126.9, 125.5, 123.0, 120.6, 120.2, 117.3, 111.3, 109.7, 99.9, 91.9, 86.1, 83.0, 26.4, 21.4, 9.6; HRMS (ESI) calcd for $C_{26}H_{25}NONa$ *m/z* [M + Na]$^{+}$: 390.1828; found: 390.1825.

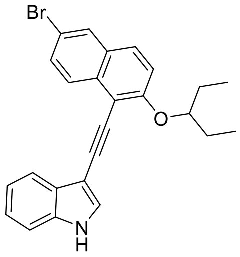

3-((6-Bromo-2-(pentan-3-yloxy)naphthalen-1-yl)ethynyl)-1H-indole (7r)

Yellow solid, mp 110.0-111.0 ℃; IR (KBr): 3418, 2920, 2210, 1459, 1262, 755 cm^{-1}; ^{1}H NMR (400 MHz, $CDCl_3$) δ 8.44 – 8.14 (m, 2H), 7.93 (q, *J* = 3.1 Hz, 2H), 7.75 – 7.58 (m, 2H), 7.53 (d, *J* = 2.6 Hz, 1H), 7.40 (t, *J* = 6.7 Hz, 1H), 7.32 – 7.18 (m, 3H), 4.40 (dq, *J* = 11.0, 5.6 Hz, 1H), 1.81 (tp, *J* = 14.1, 7.0 Hz, 4H), 1.06 (t, *J* = 7.4 Hz, 6H); ^{13}C NMR (100 MHz, $CDCl_3$) δ 158.0, 135.2, 133.0, 130.1, 129.8, 128.6, 127.8, 127.5, 123.1, 120.7, 120.2, 120.1, 117.9, 117.8, 116.9, 111.4, 99.6, 92.6, 85.4, 82.8, 26.4, 9.6; HRMS (ESI) calcd for $C_{25}H_{22}BrNONa$ *m/z* [M + Na]$^{+}$: 454.0777; found: 454.0775.

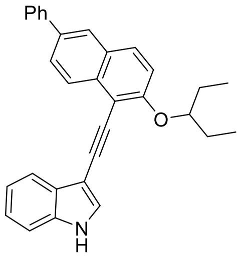

3-((2-(Pentan-3-yloxy)-6-phenylnaphthalen-1-yl)ethynyl)-1H-indole (7s)

Yellow solid, mp 128.0-129.0 ℃; IR (KBr): 3410, 2926, 2208, 1588, 1462, 752 cm^{-1}; ^{1}H NMR (600 MHz, $CDCl_3$) δ 8.53 (d, *J* = 8.7 Hz, 1H), 8.23 (s, 1H), 7.97 (d, *J* = 10.8 Hz, 2H), 7.82 (dd, *J* = 17.8, 8.8 Hz, 2H), 7.72 (d, *J* = 7.6 Hz, 2H), 7.52 – 7.50 (m, 1H), 7.47 (t, *J* = 7.6 Hz, 2H), 7.37 (q, *J* = 7.4, 6.2 Hz, 2H), 7.30 – 7.20 (m, 3H), 4.43 (p, *J* = 5.6 Hz, 1H), 1.83 (ddh, *J* = 21.2, 14.2, 7.4 Hz, 4H), 1.07 (t, *J* = 7.4 Hz, 6H); ^{13}C NMR (150 MHz, $CDCl_3$) δ 157.9, 141.0, 136.9, 135.3, 133.7, 129.2, 129.0, 128.8, 128.6, 127.4, 127.2, 127.1, 126.5, 126.2, 125.8, 123.1, 120.7, 120.2, 117.5, 111.4, 109.6, 99.8, 92.2, 85.9, 83.0, 26.5, 9.6; HRMS (ESI) calcd for $C_{31}H_{27}NONa$ *m/z* $[M + Na]^+$: 452.1985; found: 452.1987.

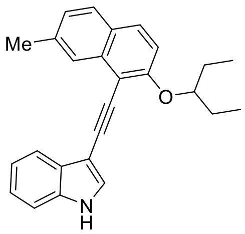

3-((7-Methyl-2-(pentan-3-yloxy)naphthalen-1-yl)ethynyl)-1H-indole (7t)

Yellow solid, mp 108.0-109.0 ℃; IR (KBr): 3410, 2925, 1456, 1241, 746 cm^{-1}; ^{1}H NMR (400 MHz, $CDCl_3$) δ 8.24 (s, 2H), 7.97 (d, *J* = 7.5 Hz, 1H), 7.69 (t, *J* = 8.6 Hz, 2H), 7.52 (d, *J* = 2.3 Hz, 1H), 7.46 – 7.35 (m, 1H), 7.26 (dd, *J* = 6.9, 2.8 Hz, 2H), 7.22 (s, 1H), 7.18 (d, *J* = 9.0 Hz, 1H), 4.40 (q, *J* = 5.7 Hz, 1H), 2.56 (s, 3H), 1.94 – 1.66 (m, *J* = 6.8 Hz, 4H), 1.06 (t, *J* = 7.4 Hz, 6H); ^{13}C NMR (100 MHz, $CDCl_3$) δ 157.9, 136.8, 135.3, 134.5, 128.7, 128.6, 127.8, 127.6, 127.0, 126.4, 124.6, 122.8, 120.4, 120.0, 116.1, 111.5, 109.0, 99.3, 92.3, 85.9, 82.9, 26.3, 22.0, 9.6; HRMS (ESI) calcd for $C_{26}H_{25}NONa$ *m/z* $[M + Na]^+$: 390.1828; found: 390.1825.

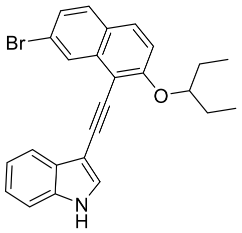

3-((7-Bromo-2-(pentan-3-yloxy)naphthalen-1-yl)ethynyl)-1H-indole (7u)

Yellow solid, mp 150.0-151.0 ℃; IR (KBr): 3418, 2923, 2200, 1458, 1266, 1098, 750 cm^{-1}; ^{1}H NMR (600 MHz, $CDCl_3$) δ 8.62 (s, 1H), 8.29 (s, 1H), 7.95 (d, *J* = 8.5 Hz, 1H), 7.69 (d, *J* = 9.0 Hz, 1H), 7.63 (d, *J* = 8.6 Hz, 1H), 7.54 (d, *J* = 2.5 Hz, 1H), 7.45 (dd, *J* = 8.6, 1.8 Hz, 1H), 7.39 (d, *J* = 2.7 Hz, 1H), 7.30 – 7.25 (m, 2H), 7.24 (t, *J* = 4.5 Hz, 1H), 4.42 (p, *J* = 5.7 Hz, 1H), 1.81 (ddp, *J* = 21.3, 14.2, 6.9 Hz, 4H), 1.06 (t, *J* = 7.4 Hz, 6H); ^{13}C NMR (150 MHz, $CDCl_3$) δ 158.4, 135.7, 135.3, 129.5, 128.7, 128.6, 127.9, 127.6, 127.5, 127.1, 123.1, 121.6, 120.8, 120.2, 117.0, 111.4, 108.9, 99.6, 92.7, 85.3, 82.8, 26.4, 9.6; HRMS (ESI) calcd for $C_{25}H_{22}BrNONa$ *m/z* [M + Na]$^{+}$: 454.0777; found: 454.0780.

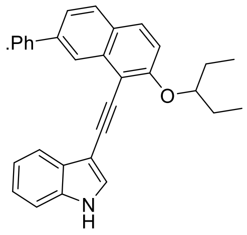

3-((2-(Pentan-3-yloxy)-7-phenylnaphthalen-1-yl)ethynyl)-1H-indole (7v)

Yellow solid, mp 112.0-113.0 ℃; IR (KBr): 3407, 2924, 2200, 1466, 1269, 758 cm^{-1}; ^{1}H NMR (600 MHz, $CDCl_3$) δ 8.73 (s, 1H), 8.21 (s, 1H), 7.97 (d, *J* = 7.5 Hz, 1H), 7.84 (d, *J* = 8.4 Hz, 1H), 7.80 (d, *J* = 7.6 Hz, 2H), 7.75 (d, *J* = 9.0 Hz, 1H), 7.66 (d, *J* = 8.3 Hz, 1H), 7.53 – 7.43 (m, 3H), 7.36 (dd, *J* = 18.6, 7.6 Hz, 2H), 7.29 – 7.15 (m, 3H), 4.42 (p, *J* = 5.5 Hz, 1H), 1.82 (ddh, *J* = 21.1, 14.1, 7.2 Hz, 4H), 1.07 (t, *J* = 7.4 Hz, 6H); ^{13}C NMR (150 MHz, $CDCl_3$) δ 158.1, 141.4, 139.5, 135.3, 134.7, 128.8, 128.7, 128.6, 128.5, 128.0, 127.6, 127.4, 127.3, 123.9, 123.8, 123.0, 120.6, 120.3, 117.2, 111.4, 110.1, 99.8, 92.7, 86.0, 83.0, 26.4, 9.6; HRMS (ESI) calcd for $C_{31}H_{27}NONa$ *m/z* [M + Na]$^{+}$: 452.1985; found: 452.1985.

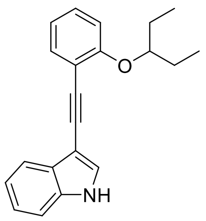

3-((2-(Pentan-3-yloxy)phenyl)ethynyl)-1H-indole (7af)

Yellow solid, mp 110.0-112.0 ℃; IR (KBr): 3401, 2923, 2100, 1478, 1279, 758 cm^{-1}; ^{1}H NMR (400 MHz, $CDCl_3$) δ 8.21 (s, 1H), 7.87 (d, *J* = 7.6 Hz, 1H), 7.51 (d, *J* = 7.8 Hz, 1H), 7.44 (d, *J* = 2.3 Hz, 1H), 7.38 (d, *J* = 7.8 Hz, 1H), 7.27 (s, 1H), 7.22 (d, *J* = 8.0 Hz, 2H), 6.91 (dd, *J* = 8.0, 5.3 Hz, 2H), 4.26 (p, *J* = 5.7 Hz, 1H), 1.78 (dh, *J* = 13.8, 6.7 Hz, 4H), 1.04 (t, *J* = 7.4 Hz, 6H); ^{13}C NMR (100 MHz, $CDCl_3$) δ 159.0, 135.2, 133.0, 128.7, 128.6, 127.4, 123.0, 120.5, 120.3, 120.2, 115.0, 114.4, 111.3, 99.5, 87.9, 86.6, 81.6, 26.1, 9.6; HRMS (ESI) calcd for $C_{21}H_{21}NONa$ *m/z* $[M + Na]^+$: 326.1515; found: 326.1518.

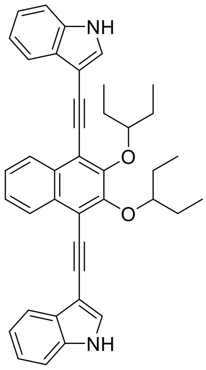

3,3'-((2,3-Bis(pentan-3-yloxy)naphthalene-1,4-diyl)bis(ethyne-2,1-diyl))bis(1H-indole) (7ag)

Yellow solid, mp 163.0-164.0 ℃; IR (KBr): 3424, 2983, 2345, 1623, 1568, 1256 cm^{-1}; ^{1}H NMR (400 MHz, $CDCl_3$) δ 8.54 (dd, *J* = 6.3, 3.3 Hz, 2H), 8.36 (s, 2H), 7.99 (dd, *J* = 6.4, 2.7 Hz, 2H), 7.59 (dd, *J* = 6.4, 3.3 Hz, 2H), 7.55 (d, *J* = 2.6 Hz, 2H), 7.46 – 7.39 (m, 2H), 7.34 – 7.27 (m, 4H), 4.81 (t, *J* = 5.7 Hz, 2H), 1.99 – 1.74 (m, 8H), 1.03 (t, *J* = 7.5 Hz, 12H); ^{13}C NMR (100 MHz, $CDCl_3$) δ 152.4, 135.3, 130.7, 128.4, 127.9, 126.0, 125.8, 123.1, 120.8, 120.2, 115.3, 111.5, 99.4, 94.0, 86.4, 85.0, 25.6, 9.4; HRMS (ESI) calcd for $C_{40}H_{38}N_2O_2Na$ *m/z* $[M + Na]^+$: 601.2825; found: 601.2827.

Phosphoric Acid Catalyzed Cycloaddition of 3-Alkynylindoles with Azonaphthalene

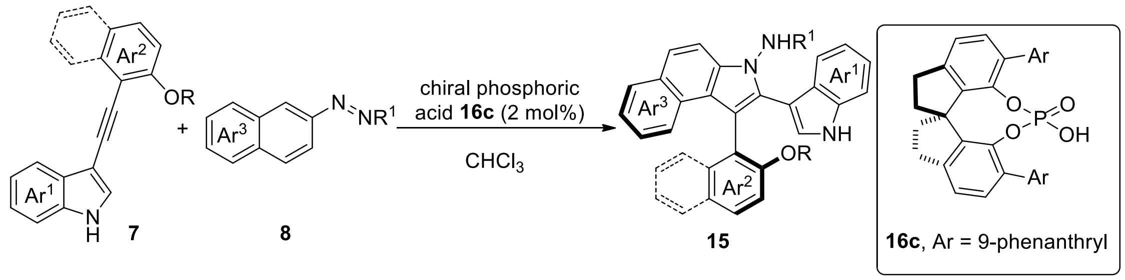

To a stirred solution of **7** (0.24 mmol), **8** (0.20 mmol) and **CPA 16c** (0.004 mmol) in $CHCl_3$ (2.0 mL) at -50 ℃ in one portion, the mixture was stirred until TLC revealed the absence of the starting material. The solvent was removed under reduced pressure, the residue was purified by flash column chromatography (petroleum ether/EtOAc) to yield the corresponding product **15**.

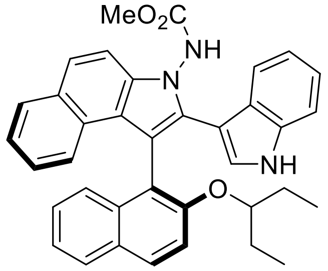

(*S*)-Methyl-(2-(1H-indol-3-yl)-1-(2-(pentan-3-yloxy)naphthalen-1-yl)-3H-benzo[e] indol-3-yl)carbamate (15a)

Brown solid (109 mg, 96% yield), mp 136.0-137.0 ℃; $[\alpha]_D^{24}$ -39.2 (*c* 1.0, $CHCl_3$, 97% ee); IR (KBr): 3420, 2960, 1458, 1242, 742 cm^{-1}; ^1H NMR (600 MHz, $CDCl_3$, 60 °C) δ 7.83 (d, *J* = 8.0 Hz, 2H), 7.76 (dd, *J* = 18.3, 8.4 Hz, 2H), 7.66 (dd, *J* = 27.4, 8.7 Hz, 3H), 7.47 (d, *J* = 7.1 Hz, 1H), 7.28 (s, 1H), 7.21 (dd, *J* = 12.1, 7.9 Hz, 4H), 7.20 – 7.12 (m, 2H), 7.07 (t, *J* = 7.3 Hz, 1H), 7.04 – 6.87 (m, 3H), 4.01 (s, 1H), 3.62 (s, 3H), 1.37 (d, *J* = 41.7 Hz, 2H), 1.31 – 1.10 (m, 2H), 0.54 (s, 3H), 0.34 (t, *J* = 7.3 Hz, 3H); ^{13}C NMR (100 MHz, $CDCl_3$) δ 156.0, 154.5, 135.5, 134.0, 132.0, 130.2, 129.1, 128.8, 128.5, 128.4, 127.6, 127.0, 126.3, 125.9, 125.4, 123.6, 123.2, 123.0, 121.8, 120.8, 120.0, 119.0, 117.9, 111.2, 110.8, 110.3, 105.5, 81.9, 53.0, 25.7, 9.2, 8.7; HRMS (ESI) calcd for $C_{37}H_{33}N_3O_3Na$ *m/z* $[M + Na]^+$: 590.2414; found: 590.2418; HPLC (Daicel

Chiralpak IA, *i*-PrOH/hexane = 20/80, flow rate 0.8 mL/min, λ = 230 nm): t_1 (major) = 8.0 min, t_2 (minor) = 9.4 min.

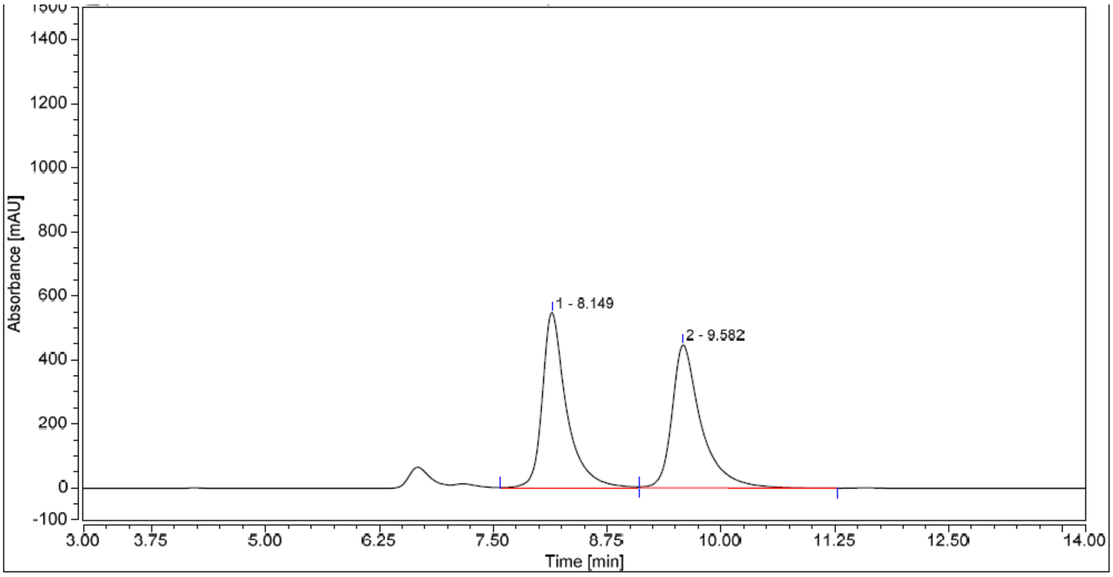

Integration Results							
No.	Peak Name	Retention Time min	Area mAU*min	Height mAU	Relative Area %	Relative Height %	Amount n.a.
1		8.149	168.572	545.972	50.46	54.99	n.a.
2		9.582	165.527	446.807	49.54	45.01	n.a.
**Total:**			**334.098**	**992.779**	**100.00**	**100.00**	

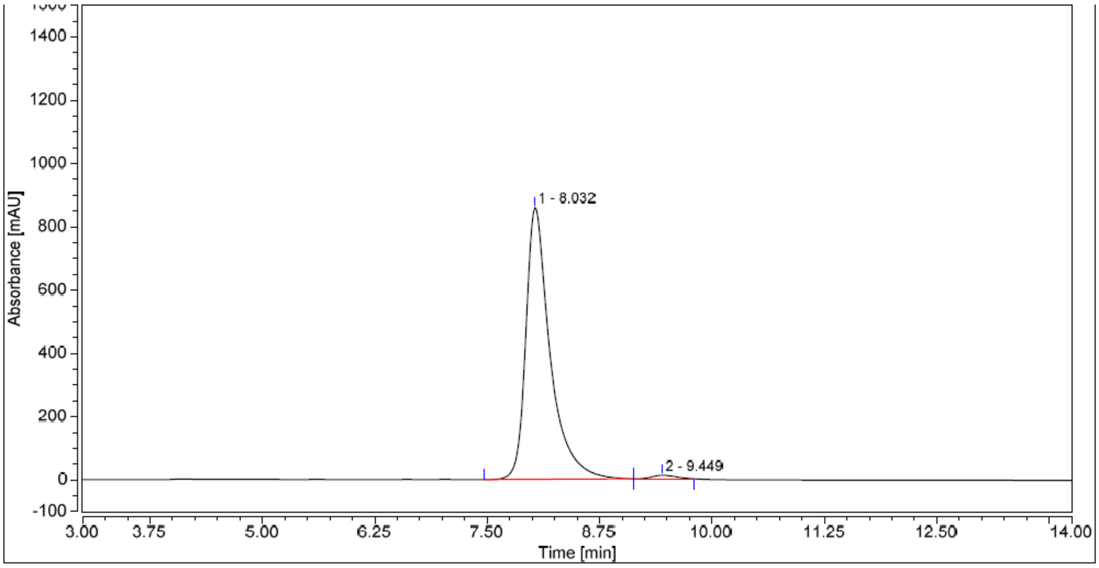

Integration Results							
No.	Peak Name	Retention Time min	Area mAU*min	Height mAU	Relative Area %	Relative Height %	Amount n.a.
1		8.032	268.228	859.132	98.77	98.70	n.a.
2		9.449	3.350	11.359	1.23	1.30	n.a.
**Total:**			**271.578**	**870.491**	**100.00**	**100.00**	

Scale up reaction of 15a:

To a stirred solution of **7a** (2.4 mmol), **8a** (2.0 mmol) and **CPA 16c** (0.04 mmol) in $CHCl_3$ (10.0 mL) at -50 ℃ in one portion, the mixture was stirred until TLC revealed the absence of the starting material. After removal of the solvent, the residue was purified by flash column chromatography (petroleum ether/AcOEt) to yield the corresponding product **15a** (1.1 g, 97% yield, 97% ee), yields are based on azonaphthalene derivative **8a**.

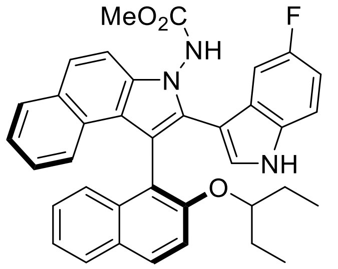

(*S*)-Methyl-(2-(5-fluoro-1H-indol-3-yl)-1-(2-(pentan-3-yloxy)naphthalen-1-yl)-3H-benzo[e]indol-3-yl)carbamate (15b)

Brown solid (105 mg, 90% yield), mp 136.0-137.0 ℃; $[\alpha]_D^{24}$ -40.8 (*c* 1.0, $CHCl_3$, 97% ee); IR (KBr): 3417, 2923, 1726, 1460, 1265, 754 cm^{-1}; ^1H NMR (600 MHz, $CDCl_3$, 60 ℃) δ 7.83 (d, *J* = 8.5 Hz, 2H), 7.78 (d, *J* = 9.0 Hz, 1H), 7.74 (d, *J* = 8.1 Hz, 1H), 7.69 (d, *J* = 8.8 Hz, 1H), 7.61 (d, *J* = 8.8 Hz, 2H), 7.28 (s, 1H), 7.25 – 7.19 (m, 4H), 7.18 – 7.08 (m, 2H), 7.04 – 6.87 (m, 3H), 6.78 (td, *J* = 9.0, 2.0 Hz, 1H), 4.14 – 3.86 (m, 1H), 3.66 (s, 3H), 1.36 (s, 2H), 1.28 – 1.08 (m, 2H), 0.57 (s, 3H), 0.34 (t, *J* = 7.4 Hz, 3H); ^{13}C NMR (100 MHz, $CDCl_3$) δ 158.07 (d, *J* = 234.0 Hz), 156.0, 154.4, 135.5, 133.9, 131.9, 131.5, 130.2, 129.1, 128.9, 128.4, 127.7, 127.4, 127.3, 126.5, 125.7, 125.5, 123.8, 123.7, 123.1, 121.6, 120.6, 118.4, 111.70 (d, *J* = 9.4 Hz), 110.9, 110.2, 110.1, 105.5, 103.96 (d, *J* = 24.8 Hz), 82.0, 53.1, 25.7, 9.2, 8.7; HRMS (ESI) calcd for $C_{37}H_{32}FN_3O_3Na$ *m/z* $[M + Na]^+$: 608.2320; found: 608.2324; HPLC (Daicel Chiralpak IA, *i*-PrOH/hexane = 20/80, flow rate 0.8 mL/min, λ = 230 nm): t_1 (major) = 7.8 min, t_2 (minor) = 9.8 min.

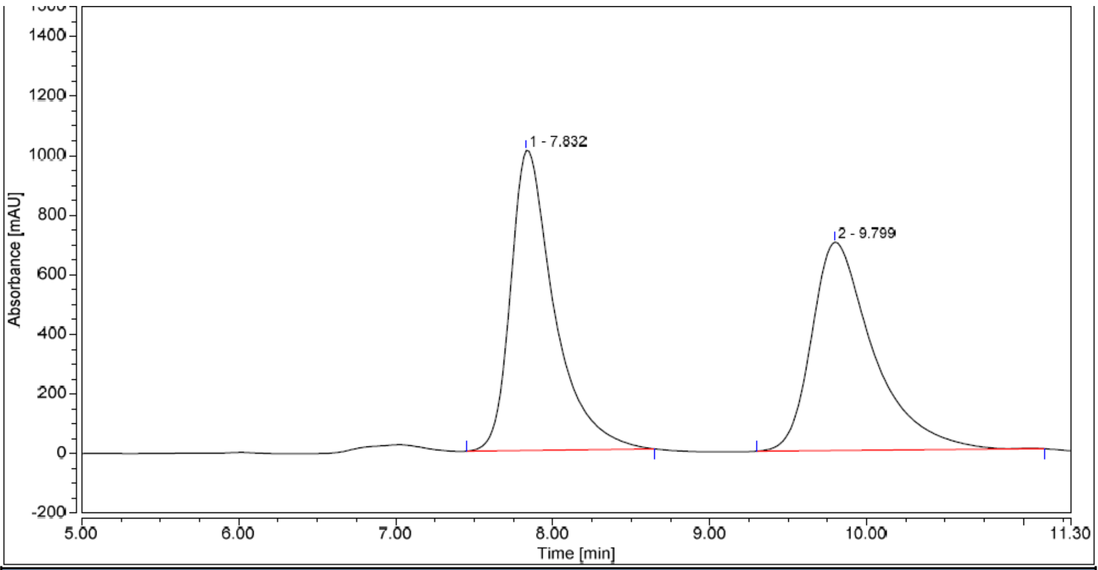

Integration Results							
No.	Peak Name	Retention Time min	Area mAU*min	Height mAU	Relative Area %	Relative Height %	Amount n.a.
1		7.832	324.441	1007.207	50.41	59.03	n.a.
2		9.799	319.107	699.030	49.59	40.97	n.a.
**Total:**			**643.548**	**1706.237**	**100.00**	**100.00**	

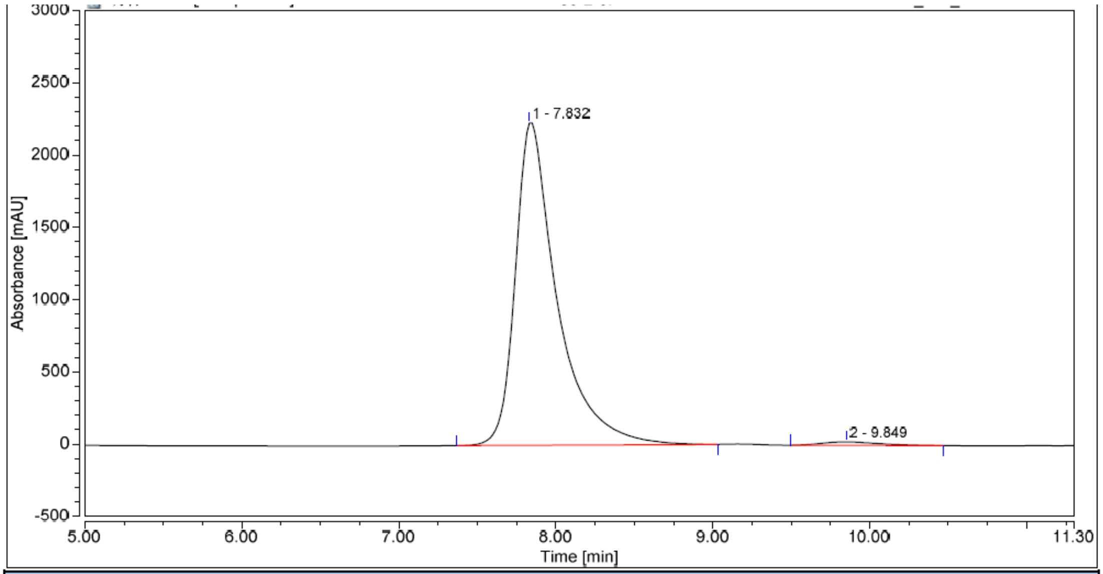

Integration Results							
No.	Peak Name	Retention Time min	Area mAU*min	Height mAU	Relative Area %	Relative Height %	Amount n.a.
1		7.832	687.266	2231.121	98.63	98.96	n.a.
2		9.849	9.569	23.522	1.37	1.04	n.a.
**Total:**			**696.836**	**2254.643**	**100.00**	**100.00**	

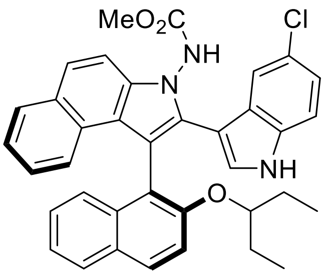

(*S*)-Methyl-(2-(5-chloro-1H-indol-3-yl)-1-(2-(pentan-3-yloxy)naphthalen-1-yl)-3H -benzo[e]indol-3-yl)carbamate (15c)

Brown solid (115 mg, 96% yield), mp 148.0-149.0 ℃; $[\alpha]_D^{24}$ -57.4 (*c* 1.0, $CHCl_3$, 98% ee); IR (KBr): 3413, 2925, 1730, 1265, 754 cm^{-1}; ^1H NMR (400 MHz, $CDCl_3$) δ 7.85 (d, *J* = 7.5 Hz, 1H), 7.78 (dd, *J* = 15.5, 8.2 Hz, 3H), 7.70 (d, *J* = 8.7 Hz, 1H), 7.62 (d, *J* = 8.6 Hz, 2H), 7.40 (s, 2H), 7.27 – 7.19 (m, 4H), 7.16 (s, 1H), 6.99 (t, *J* = 7.4 Hz, 1H), 6.85 (dd, *J* = 46.4, 7.7 Hz, 3H), 4.01 (s, 1H), 3.68 (s, 3H), 1.41 – 1.10 (m, 4H), 0.54 (s, 3H), 0.33 (s, 3H); ^{13}C NMR (100 MHz, $CDCl_3$) δ 156.1, 154.4, 135.4, 133.9, 133.6, 131.3, 130.2, 129.1, 129.0, 128.4, 127.7, 126.8, 126.5, 125.5, 123.8, 123.1, 122.0, 121.2, 120.6, 120.3, 118.5, 117.8, 112.0, 111.1, 110.2, 104.9, 81.8, 53.2, 25.6, 9.2, 8.6; HRMS (ESI) calcd for $C_{37}H_{32}ClN_3O_3Na$ *m/z* $[M + Na]^+$: 624.2024; found: 624.2020; HPLC (Daicel Chiralpak IA, *i*-PrOH/hexane = 20/80, flow rate 0.8 mL/min, λ = 230 nm): t_1 (major) = 7.8 min, t_2 (minor) = 9.7 min.

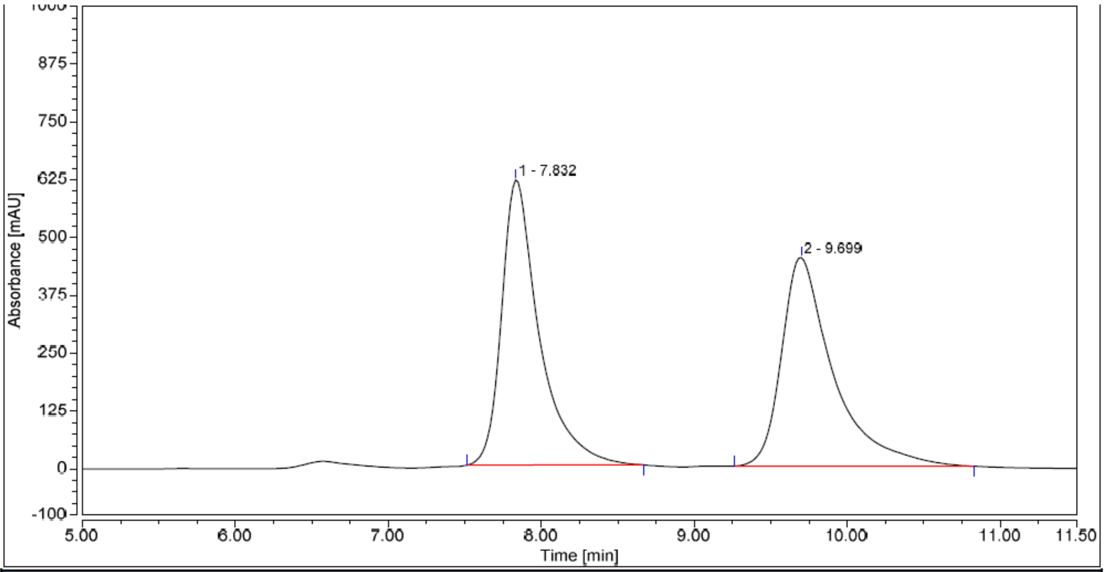

Integration Results

No.	Peak Name	Retention Time min	Area mAU*min	Height mAU	Relative Area %	Relative Height %	Amount n.a.
1		7.832	175.652	616.162	49.91	57.73	n.a.
2		9.699	176.310	451.078	50.09	42.27	n.a.
**Total:**			**351.962**	**1067.240**	**100.00**	**100.00**	

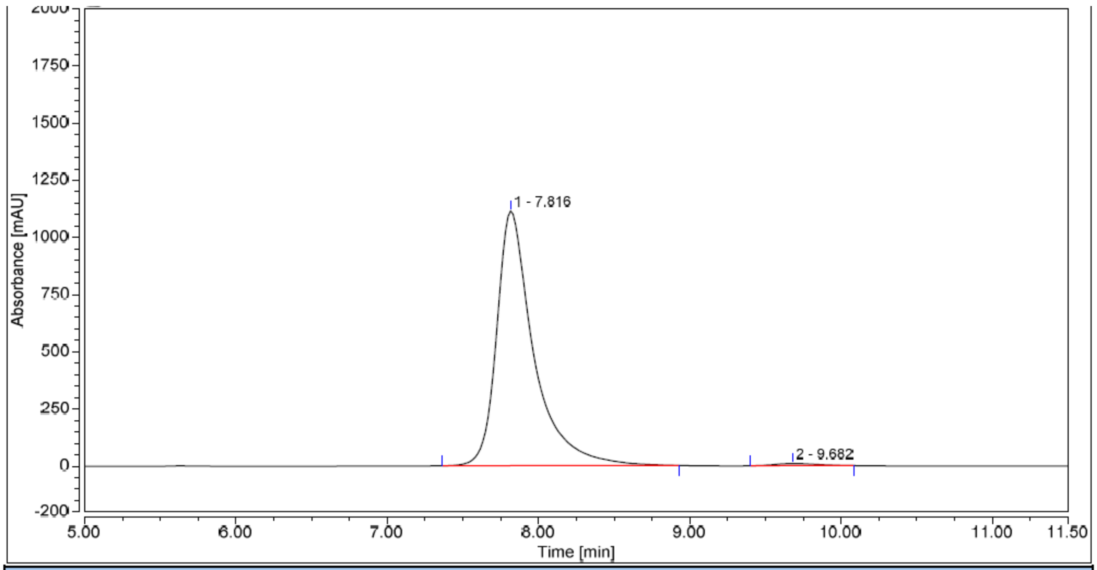

Integration Results							
No.	Peak Name	Retention Time min	Area mAU*min	Height mAU	Relative Area %	Relative Height %	Amount n.a.
1		7.816	315.081	1113.508	99.05	99.15	n.a.
2		9.682	3.013	9.515	0.95	0.85	n.a.
Total:			**318.095**	**1123.023**	**100.00**	**100.00**	

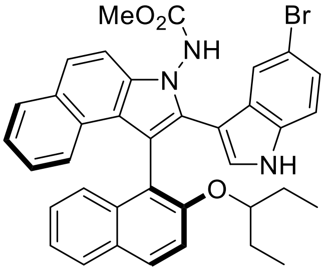

(*S*)-Methyl-(2-(5-bromo-1H-indol-3-yl)-1-(2-(pentan-3-yloxy)naphthalen-1-yl)-3H -benzo[e]indol-3-yl)carbamate (15d)

Brown solid (122 mg, 95% yield), mp 138.0-139.0 ℃; $[\alpha]_D^{24}$ -53.0 (*c* 1.0, $CHCl_3$, 98% ee); IR (KBr): 3413, 293, 1726, 1458, 1267, 754 cm^{-1}; ^1H NMR (400 MHz, $CDCl_3$) δ 7.83 (d, *J* = 7.7 Hz, 1H), 7.77 (d, *J* = 9.0 Hz, 1H), 7.73 (d, *J* = 8.0 Hz, 1H), 7.62 (dd, *J* = 37.2, 8.8 Hz, 4H), 7.46 (s, 1H), 7.21 (t, *J* = 3.9 Hz, 5H), 7.12 (s, 1H), 6.98 (t, *J* = 7.2 Hz, 1H), 6.91 (d, *J* = 7.5 Hz, 1H), 6.66 (s, 1H), 6.46 (s, 1H), 4.00 (s, 1H), 3.64 (s, 3H), 1.40 – 1.07 (m, 4H), 0.54 (s, 3H), 0.32 (s, 3H); ^{13}C NMR (100 MHz, $CDCl_3$) δ 156.1, 154.3, 135.4, 133.9, 131.2, 130.1, 129.0, 128.4, 127.7, 126.5, 126.5, 125.6, 125.5, 124.5, 123.8, 123.1, 121.6, 120.5, 118.4, 117.7, 113.2, 112.4, 111.1, 110.2, 104.8, 81.6, 53.3, 25.5, 9.1, 8.6; HRMS (ESI) calcd for $C_{37}H_{32}BrN_3O_3Na$ *m/z* $[M + Na]^+$:

668.1519; found: 668.1520; HPLC (Daicel Chiralpak IA, *i*-PrOH/hexane = 20/80, flow rate 0.8 mL/min, λ = 230 nm): t_1 (major) = 8.2 min, t_2 (minor) = 10.3 min.

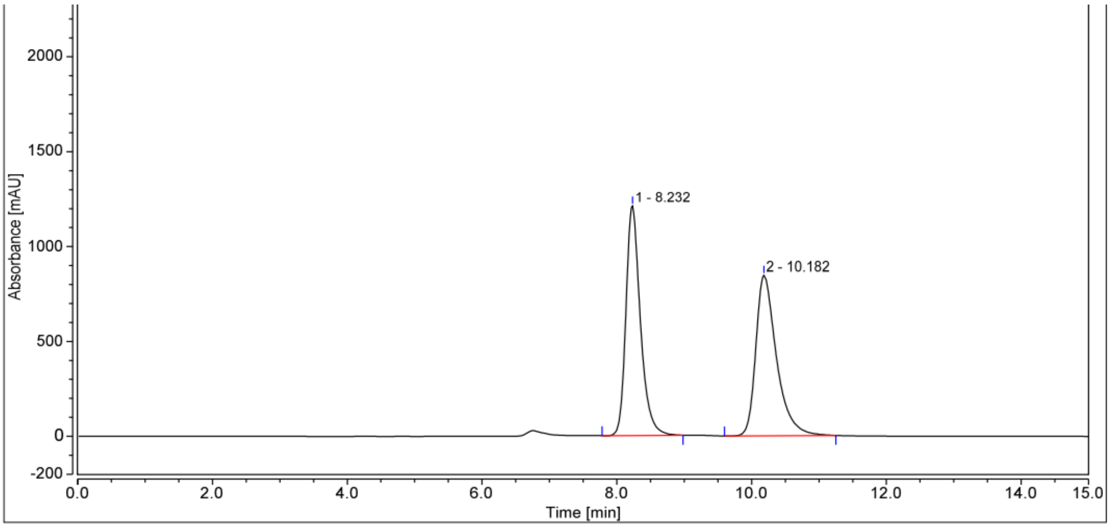

Integration Results							
No.	Peak Name	Retention Time min	Area mAU*min	Height mAU	Relative Area %	Relative Height %	Amount n.a.
1		8.232	300.500	1209.745	49.99	58.84	n.a.
2		10.182	300.657	846.115	50.01	41.16	n.a.
**Total:**			**601.157**	**2055.860**	**100.00**	**100.00**	

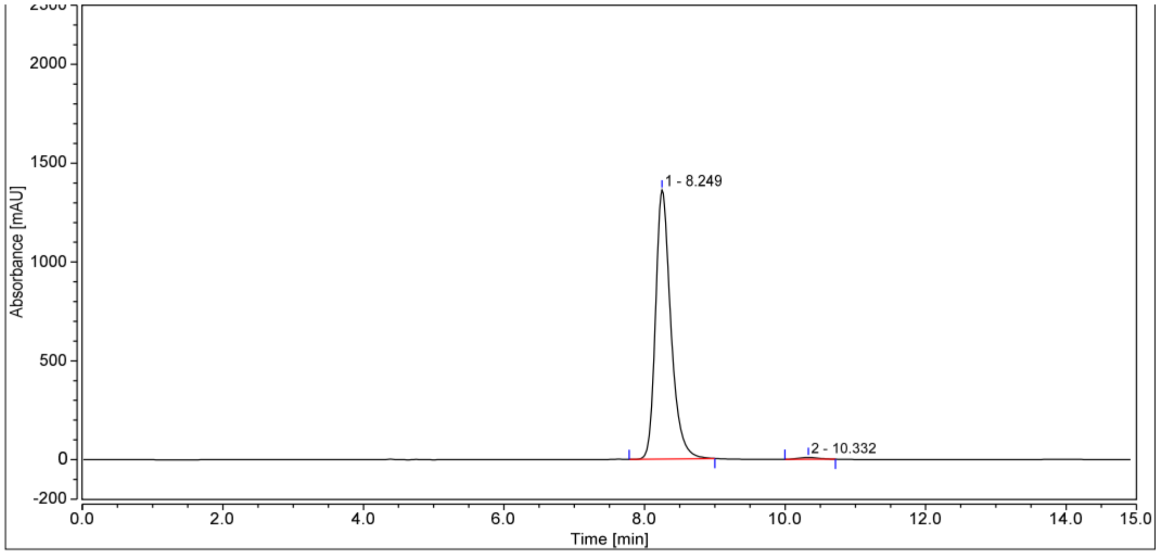

Integration Results							
No.	Peak Name	Retention Time min	Area mAU*min	Height mAU	Relative Area %	Relative Height %	Amount n.a.
1		8.249	343.748	1361.631	99.10	99.33	n.a.
2		10.332	3.114	9.183	0.90	0.67	n.a.
**Total:**			**346.862**	**1370.814**	**100.00**	**100.00**	

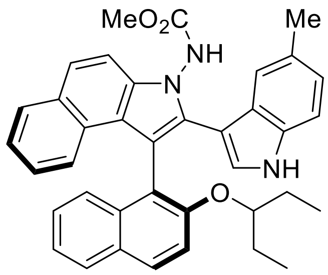

(*S*)-Methyl-(2-(5-methyl-1H-indol-3-yl)-1-(2-(pentan-3-yloxy)naphthalen-1-yl)-3H-benzo[e]indol-3-yl)carbamate (15e)

Brown solid (102 mg, 88% yield), mp 149.0-150.0 ℃; $[\alpha]_D^{24}$ -21.4 (*c* 1.0, $CHCl_3$, 98% ee); IR (KBr): 3411, 2923, 1728, 1460, 1242, 802 cm^{-1}; ^1H NMR (600 MHz, $CDCl_3$, 60 °C) δ 7.83 (d, *J* = 8.1 Hz, 1H), 7.76 (dd, *J* = 14.6, 8.6 Hz, 3H), 7.66 (dd, *J* = 28.6, 8.8 Hz, 3H), 7.29 (s, 1H), 7.26 – 7.20 (m, 5H), 7.17 (d, *J* = 8.9 Hz, 2H), 7.04 (d, *J* = 8.3 Hz, 1H), 6.97 (t, *J* = 7.6 Hz, 1H), 6.88 (d, *J* = 8.2 Hz, 1H), 3.99 (s, 1H), 3.63 (s, 3H), 2.29 (s, 3H), 1.38 – 1.25 (m, 2H), 1.27 – 1.10 (m, 2H), 0.53 (s, 3H), 0.35 (t, *J* = 7.4 Hz, 3H); ^{13}C NMR (100 MHz, $CDCl_3$) δ 156.0, 154.6, 135.7, 133.9, 133.8, 132.3, 130.2, 129.1, 128.7, 128.4, 128.3, 127.6, 127.2, 126.3, 125.9, 125.4, 123.6, 123.5, 123.2, 123.0, 122.0, 120.7, 119.0, 118.7, 110.8, 110.6, 110.3, 104.9, 81.8, 53.0, 25.6, 21.3, 9.1, 8.6; HRMS (ESI) calcd for $C_{38}H_{35}N_3O_3Na$ *m/z* $[M + Na]^+$: 604.2571; found: 604.2576; HPLC (Daicel Chiralpak IA, *i*-PrOH/hexane = 10/90, flow rate 0.8 mL/min, λ = 260 nm): t_1 (major) = 19.4 min, t_2 (minor) = 22.9 min.

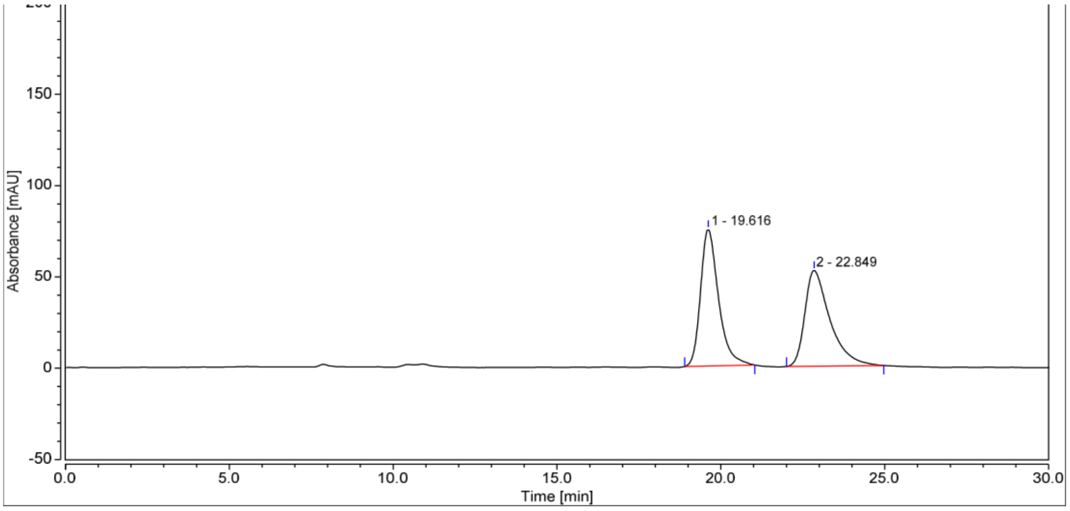

Integration Results							
No.	Peak Name	Retention Time min	Area mAU*min	Height mAU	Relative Area %	Relative Height %	Amount n.a.
1		19.616	47.595	74.831	50.56	58.82	n.a.
2		22.849	46.535	52.394	49.44	41.18	n.a.
Total:			94.130	127.225	100.00	100.00	

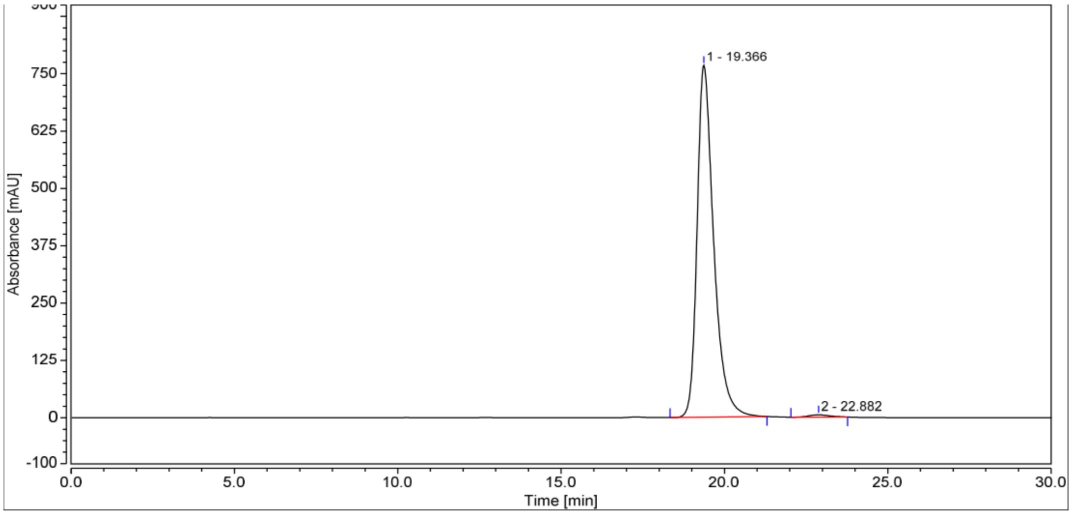

Integration Results							
No.	Peak Name	Retention Time min	Area mAU*min	Height mAU	Relative Area %	Relative Height %	Amount n.a.
1		19.366	452.474	767.163	99.19	99.37	n.a.
2		22.882	3.684	4.889	0.81	0.63	n.a.
Total:			456.158	772.052	100.00	100.00	

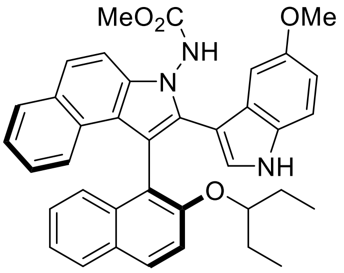

(*S*)-Methyl-(2-(5-methoxy-1H-indol-3-yl)-1-(2-(pentan-3-yloxy)naphthalen-1-yl)-3H-benzo[e]indol-3-yl)carbamate (15f)

Brown solid (117 mg, 98% yield), mp 132.0-133.0 ℃; $[\alpha]_D^{24}$ +20.4 (*c* 1.0, $CHCl_3$, 98% ee); IR (KBr): 3415, 29231, 1730, 1458, 1244, 802 cm^{-1}; ^1H NMR (400 MHz, $CDCl_3$) δ 7.85 (d, *J* = 8.0 Hz, 2H), 7.82 – 7.76 (m, 2H), 7.70 (d, *J* = 8.8 Hz, 2H), 7.63 (d, *J* = 8.8 Hz, 1H), 7.45 (s, 1H), 7.24 (s, 2H), 7.20 (q, *J* = 10.8, 8.8 Hz, 4H), 6.97 (t, *J* = 8.1 Hz, 1H), 6.90 (d, *J* = 7.8 Hz, 1H), 6.73 (s, 1H), 6.63 (s, 1H), 3.94 (s, 1H), 3.69 (s, 3H), 3.49 (s, 3H), 1.30 – 1.10 (m, 4H), 0.50 (s, 3H), 0.36 (s, 3H); ^{13}C NMR (100 MHz, DMSO) δ 156.3, 154.4, 153.4, 135.2, 133.3, 132.8, 130.6, 129.7, 128.9, 128.7, 128.5, 127.9, 127.3, 126.4, 125.2, 125.0, 123.4, 122.8, 122.5, 121.0, 120.7, 119.7, 118.6, 117.4, 112.0, 111.7, 111.0, 108.8, 104.8, 101.1, 100.7, 80.2, 54.8, 52.5, 25.4, 9.2, 8.7; HRMS (ESI) calcd for $C_{38}H_{35}N_3O_4Na$ *m/z* $[M + Na]^+$: 620.2520; found: 620.2525; HPLC (Daicel Chiralpak IA, *i*-PrOH/hexane = 20/80, flow rate 0.8 mL/min, λ = 230 nm): t_1 (major) = 8.9 min, t_2 (minor) = 11.3 min.

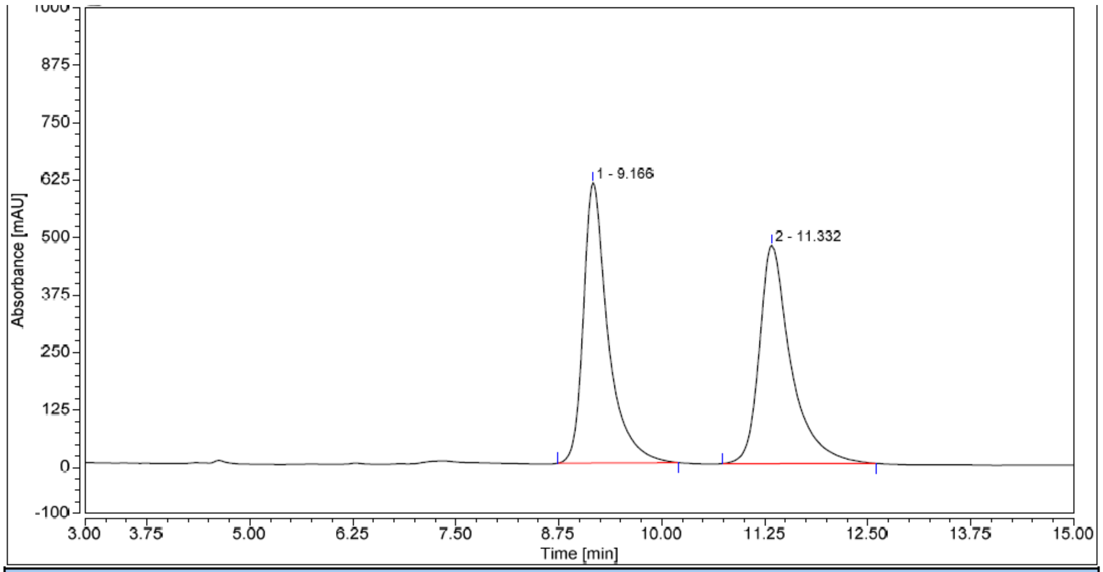

Integration Results

No.	Peak Name	Retention Time min	Area mAU*min	Height mAU	Relative Area %	Relative Height %	Amount n.a.
1		9.166	211.470	609.929	50.07	56.24	n.a.
2		11.332	210.903	474.663	49.93	43.76	n.a.
**Total:**			**422.373**	**1084.591**	**100.00**	**100.00**	

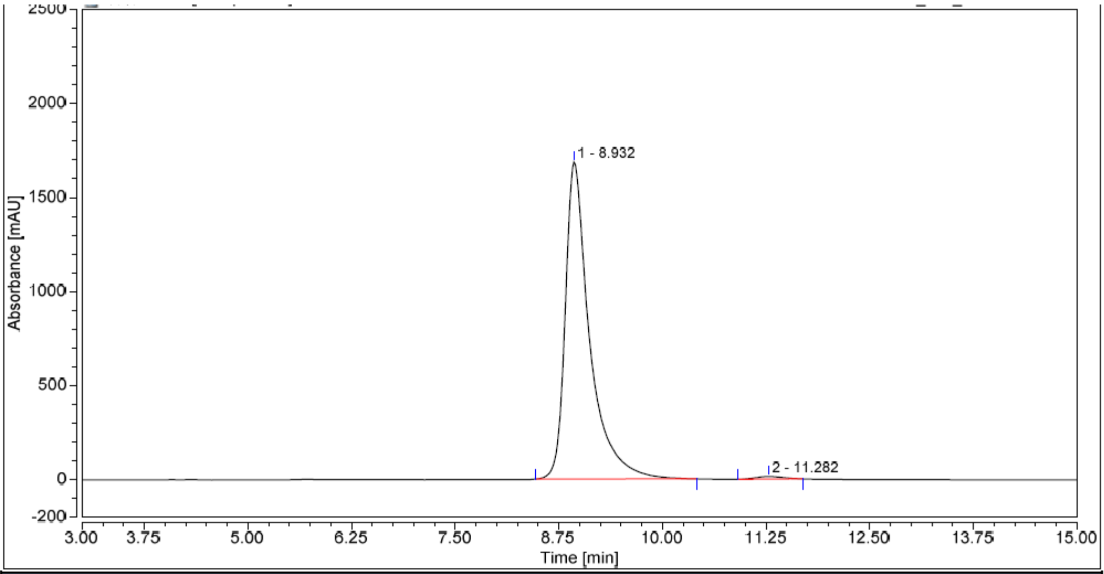

Integration Results							
No.	Peak Name	Retention Time min	Area mAU*min	Height mAU	Relative Area %	Relative Height %	Amount n.a.
1		8.932	575.954	1685.530	99.12	99.17	n.a.
2		11.282	5.112	14.022	0.88	0.83	n.a.
Total:			**581.067**	**1699.552**	**100.00**	**100.00**	

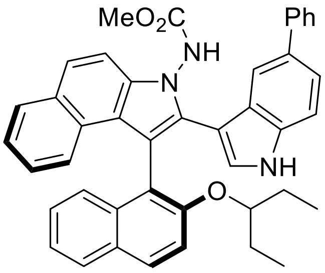

(*S*)-Methyl-(1-(2-(pentan-3-yloxy)naphthalen-1-yl)-2-(5-phenyl-1H-indol-3-yl)-3 H-benzo[e]indol-3-yl)carbamate (15g)

Brown solid (96 mg, 75% yield), mp 145.0-146.0 ℃; $[\alpha]_D^{24}$ +18.6 (*c* 1.0, $CHCl_3$, 96% ee); IR (KBr): 3419, 2921, 2364, 1625, 1458, 1238, 802 cm^{-1}; ^{1}H NMR (400 MHz, $CDCl_3$) δ 7.99 (s, 1H), 7.86 (d, *J* = 8.0 Hz, 1H), 7.80 (d, *J* = 8.8 Hz, 2H), 7.71 (d, *J* = 8.8 Hz, 2H), 7.63 (d, *J* = 8.8 Hz, 1H), 7.42 – 7.27 (m, 7H), 7.26 – 7.16 (m, 6H), 7.13 – 6.96 (m, 3H), 3.96 (s, 1H), 3.61 (s, 3H), 1.26 (s, 4H), 0.46 (s, 3H), 0.36 (s, 3H); ^{13}C NMR (100 MHz, $CDCl_3$) δ 156.8, 156.1, 154.3, 142.0, 135.6, 134.9, 133.9, 133.0, 132.0, 130.2, 129.3, 128.9, 128.4, 127.8, 127.2, 127.0, 126.6, 126.0, 125.9, 125.4, 123.8, 123.6, 123.2, 123.0, 121.4, 120.7, 117.5, 111.3, 110.7, 110.3, 105.7, 82.2, 53.0, 25.6, 9.0, 8.7; HRMS (ESI) calcd for $C_{43}H_{37}N_3O_3Na$ *m/z* $[M + Na]^+$: 666.2727; found: 666.2730; HPLC (Daicel Chiralpak IA, *i*-PrOH/hexane = 20/80, flow rate 0.8 mL/min, λ = 230 nm): t_1 (major) = 9.5 min, t_2 (minor) = 10.8 min.

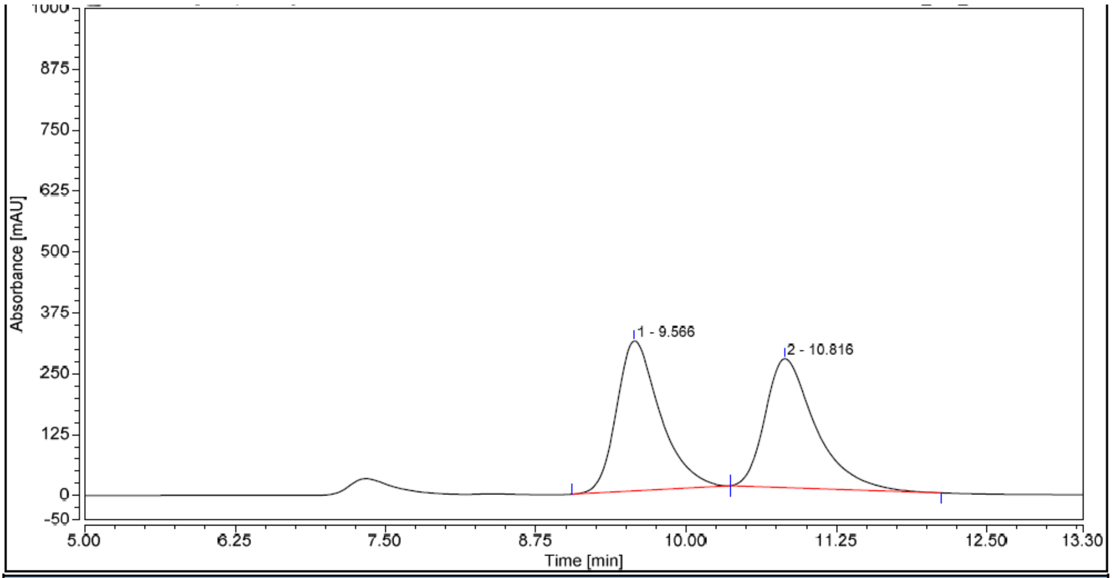

Integration Results

No.	Peak Name	Retention Time min	Area mAU*min	Height mAU	Relative Area %	Relative Height %	Amount n.a.
1		9.566	129.354	307.693	50.18	53.77	n.a.
2		10.816	128.426	264.530	49.82	46.23	n.a.
**Total:**			**257.780**	**572.223**	**100.00**	**100.00**	

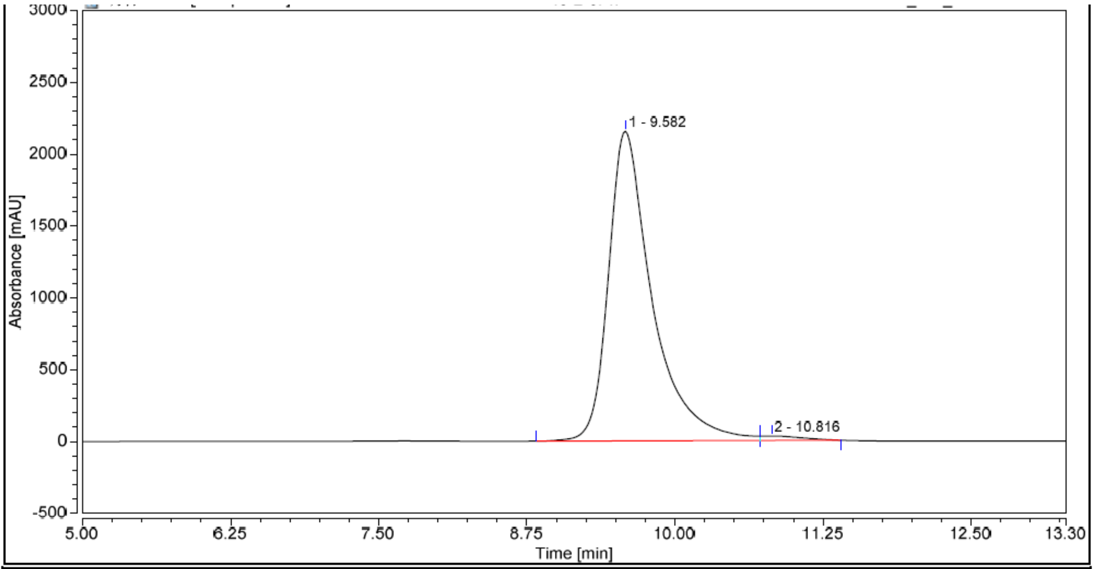

Integration Results

No.	Peak Name	Retention Time min	Area mAU*min	Height mAU	Relative Area %	Relative Height %	Amount n.a.
1		9.582	922.124	2154.520	98.74	98.63	n.a.
2		10.816	11.750	30.008	1.26	1.37	n.a.
**Total:**			**933.874**	**2184.528**	**100.00**	**100.00**	

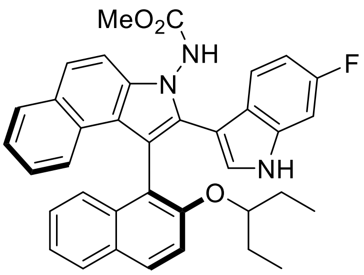

(*S*)-Methyl-(2-(6-fluoro-1H-indol-3-yl)-1-(2-(pentan-3-yloxy)naphthalen-1-yl)-3H-benzo[e]indol-3-yl)carbamate (15h)

Brown solid (115 mg, 98% yield), mp 142.0-143.0 ℃; $[\alpha]_D^{24}$ -38.0 (*c* 1.0, $CHCl_3$, 97% ee); IR (KBr): 3409, 2920, 1620, 1453, 1261, 752 cm^{-1}; ^1H NMR (600 MHz, $CDCl_3$, 60 °C) δ 7.83 (d, *J* = 8.1 Hz, 1H), 7.76 (dd, *J* = 23.7, 8.6 Hz, 3H), 7.68 (d, *J* = 8.7 Hz, 1H), 7.60 (d, *J* = 8.8 Hz, 2H), 7.34 (s, 1H), 7.28 (s, 1H), 7.21 (dd, *J* = 12.8, 5.1 Hz, 4H), 7.14 (d, *J* = 7.3 Hz, 1H), 6.97 (t, *J* = 7.6 Hz, 1H), 6.86 (s, 1H), 6.70 (dd, *J* = 22.6, 9.3 Hz, 2H), 4.01 (s, 1H), 3.63 (s, 3H), 1.40 – 1.24 (m, 2H), 1.30 – 1.09 (m, 2H), 0.56 (s, 3H), 0.33 (t, *J* = 7.4 Hz, 3H); ^{13}C NMR (100 MHz, $CDCl_3$) δ 159.47 (d, *J* = 237.5 Hz), 156.0, 154.3, 135.4, 135.2, 133.9, 131.6, 130.2, 129.2, 129.0, 128.4, 128.4, 127.7, 126.5, 125.7, 125.5, 123.7, 123.3, 123.1, 121.9, 120.6, 119.8, 118.7, 117.9, 110.9, 110.2, 108.48, 105.2, 97.20, 82.0, 53.1, 25.6, 9.2, 8.7; HRMS (ESI) calcd for $C_{37}H_{32}FN_3O_3Na$ *m/z* $[M + Na]^+$: 608.2320; found: 608.2315; HPLC (Daicel Chiralpak IA, *i*-PrOH/hexane = 20/80, flow rate 0.8 mL/min, *λ* = 230 nm): t_1 (major) = 7.7 min, t_2 (minor) = 9.7 min.

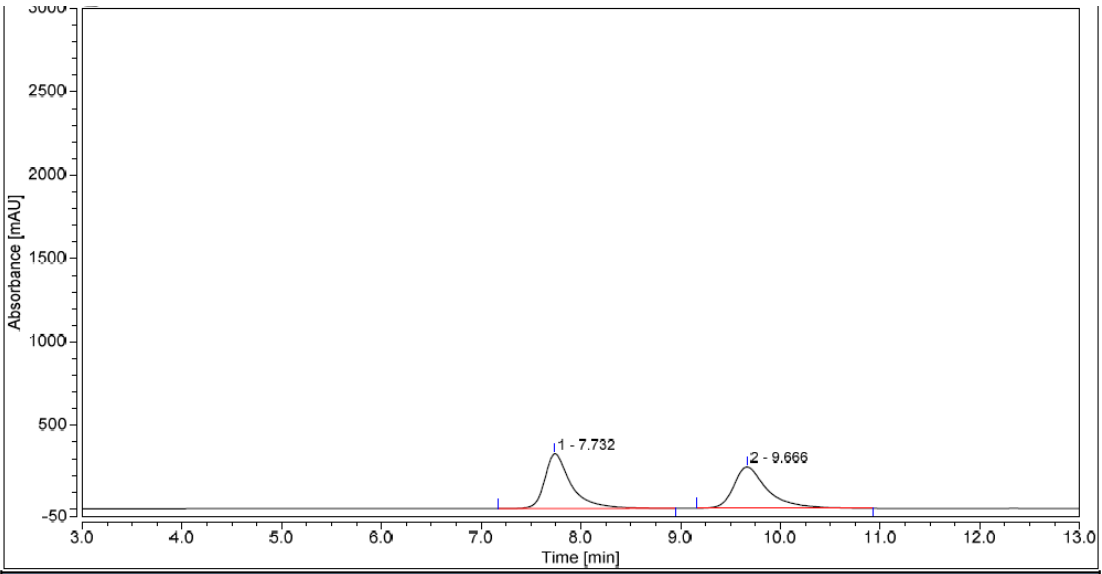

Integration Results							
No.	Peak Name	Retention Time min	Area mAU*min	Height mAU	Relative Area %	Relative Height %	Amount n.a.
1		7.732	100.343	327.221	50.61	56.84	n.a.
2		9.666	97.938	248.509	49.39	43.16	n.a.
Total:			198.281	575.730	100.00	100.00	

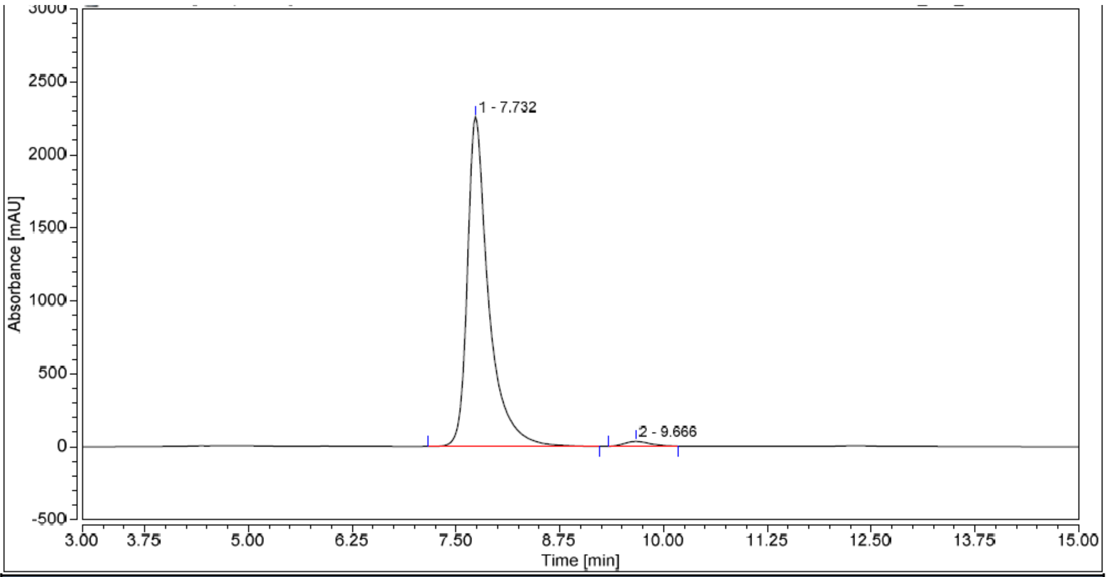

Integration Results

No.	Peak Name	Retention Time min	Area mAU*min	Height mAU	Relative Area %	Relative Height %	Amount n.a.
1		7.732	672.318	2257.994	98.28	98.56	n.a.
2		9.666	11.743	33.058	1.72	1.44	n.a.
**Total:**			**684.061**	**2291.051**	**100.00**	**100.00**	

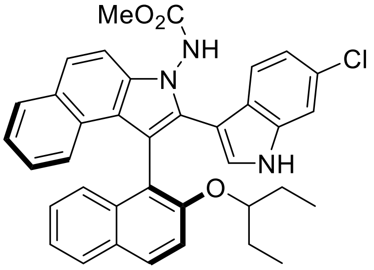

(*S*)-Methyl-(2-(6-chloro-1H-indol-3-yl)-1-(2-(pentan-3-yloxy)naphthalen-1-yl)-3H-benzo[e]indol-3-yl)carbamate (15i)

Brown solid (118 mg, 98% yield), mp 210.0-211.0 ℃; $[\alpha]_D^{24}$ -27.2 (*c* 1.0, $CHCl_3$, 95% ee); IR (KBr): 3415, 2923, 1731, 1458, 1244, 804 cm^{-1}; ^1H NMR (400 MHz, $CDCl_3$) δ 7.81 (td, *J* = 17.3, 14.6, 7.9 Hz, 4H), 7.71 (d, *J* = 8.7 Hz, 1H), 7.63 (d, *J* = 8.6 Hz, 1H), 7.42 (s, 2H), 7.29 – 7.09 (m, 6H), 7.05 – 6.81 (m, 4H), 4.02 (s, 1H), 3.69 (s, 3H), 1.26 (t, *J* = 33.5 Hz, 4H), 0.54 (s, 3H), 0.34 (s, 3H); ^{13}C NMR (100 MHz, $CDCl_3$) δ 156.5, 154.4, 135.6, 135.4, 134.0, 131.4, 130.2, 129.0, 128.4, 127.8, 126.5, 125.9, 125.7, 125.5, 125.3, 123.8, 123.1, 122.0, 120.5, 119.9, 117.7, 110.9, 110.2, 105.4, 81.9, 53.1, 25.7, 9.3, 8.6; HRMS (ESI) calcd for $C_{37}H_{32}ClN_3O_3Na$ *m/z* $[M + Na]^+$: 624.2024; found: 624.2029; HPLC (Daicel Chiralpak IA, *i*-PrOH/hexane = 20/80, flow rate 0.8 mL/min, λ = 230 nm): t_1 (major) = 8.3 min, t_2 (minor) = 10.4 min.

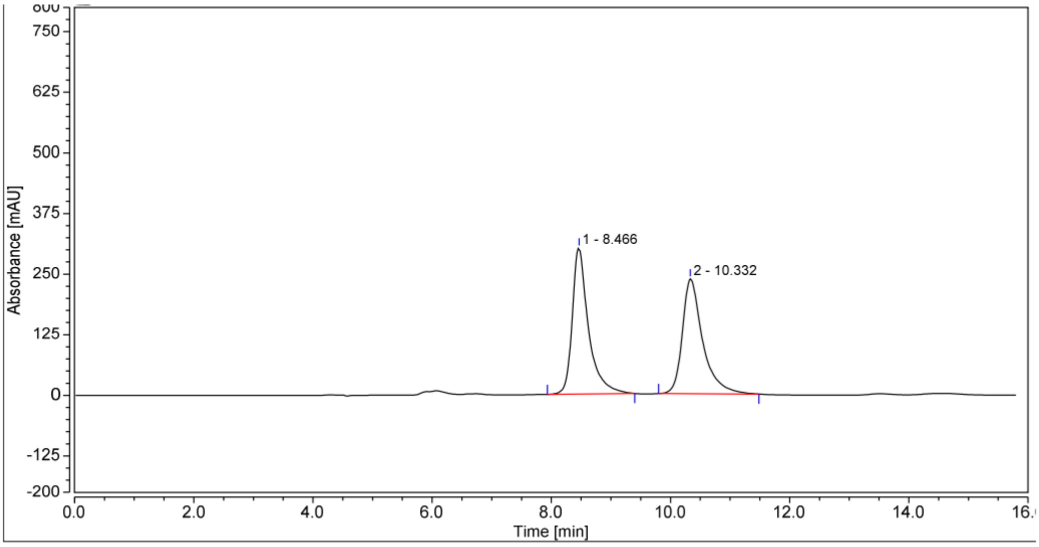

Integration Results

No.	Peak Name	Retention Time min	Area mAU*min	Height mAU	Relative Area %	Relative Height %	Amount n.a.
1		8.466	94.901	301.387	50.31	55.98	n.a.
2		10.332	93.720	236.949	49.69	44.02	n.a.
**Total:**			**188.621**	**538.336**	**100.00**	**100.00**	

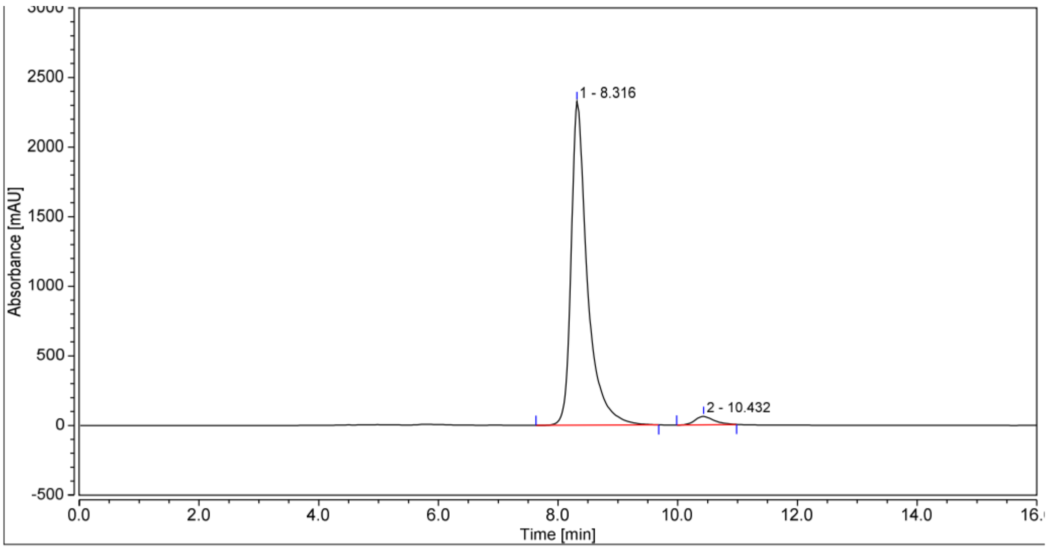

Integration Results

No.	Peak Name	Retention Time min	Area mAU*min	Height mAU	Relative Area %	Relative Height %	Amount n.a.
1		8.316	733.748	2324.315	97.05	97.47	n.a.
2		10.432	22.289	60.241	2.95	2.53	n.a.
**Total:**			**756.036**	**2384.556**	**100.00**	**100.00**	

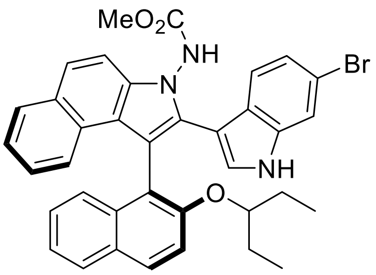

(*S*)-Methyl-(2-(6-bromo-1H-indol-3-yl)-1-(2-(pentan-3-yloxy)naphthalen-1-yl)-3H -benzo[e]indol-3-yl)carbamate (15j)

Brown solid (125 mg, 97% yield), mp 143.0-144.0 ℃; $[\alpha]_D^{24}$ -27.0 (*c* 1.0, $CHCl_3$, 95% ee); IR (KBr): 3417, 2926, 1729, 1456, 1243, 802 cm^{-1}; ^1H NMR (400 MHz, $CDCl_3$) δ 7.85 (d, *J* = 7.9 Hz, 1H), 7.81 (d, *J* = 9.0 Hz, 1H), 7.77 (d, *J* = 6.6 Hz, 1H), 7.66 (dd, *J* = 35.2, 8.6 Hz, 4H), 7.39 (s, 1H), 7.30 – 7.19 (m, 6H), 7.17 (s, 1H), 6.98 (dd, *J* = 16.4, 8.8 Hz, 3H), 4.00 (s, 1H), 3.68 (s, 3H), 1.31 – 1.15 (m, 4H), 0.56 (s, 3H), 0.32 (s, 3H); ^{13}C NMR (100 MHz, $CDCl_3$) δ 156.4, 154.3, 136.0, 135.4, 134.0, 131.3, 130.1, 129.1, 128.4, 127.8, 126.5, 125.5, 123.8, 123.1, 121.8, 120.4, 120.2, 118.6, 117.8, 115.4, 113.8, 111.0, 110.2, 105.3, 82.0, 53.1, 25.6, 9.2, 8.6; HRMS (ESI) calcd for $C_{37}H_{32}BrN_3O_3Na$ *m/z* $[M + Na]^+$: 668.1519; found: 668.1523; HPLC (Daicel Chiralpak IA, *i*-PrOH/hexane = 20/80, flow rate 0.8 mL/min, *λ* = 230 nm): t_1 (major) = 8.9 min, t_2 (minor) = 11.0 min.

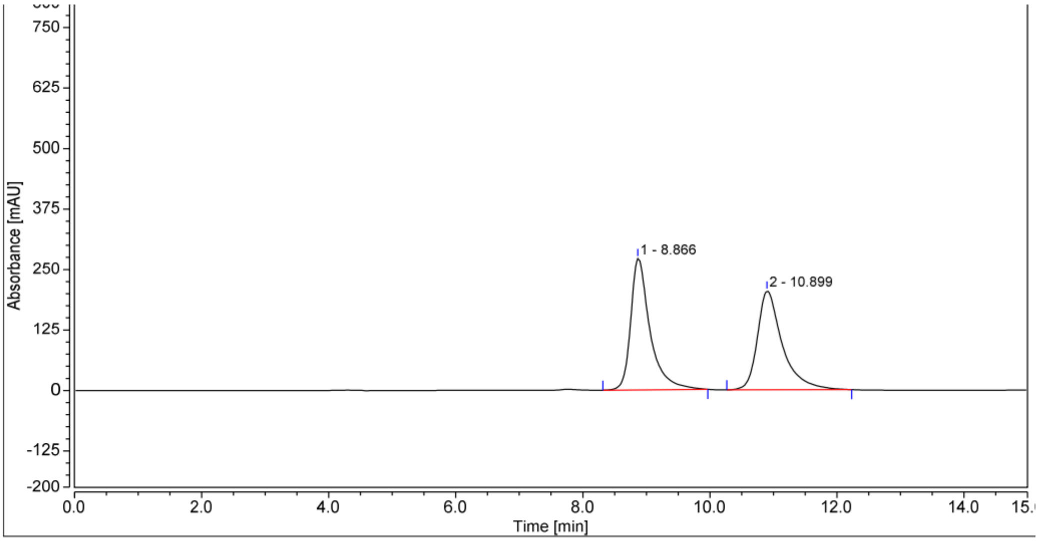

Integration Results

No.	Peak Name	Retention Time min	Area mAU*min	Height mAU	Relative Area %	Relative Height %	Amount n.a.
1		8.866	95.526	271.580	50.72	57.07	n.a.
2		10.899	92.820	204.270	49.28	42.93	n.a.
**Total:**			**188.346**	**475.850**	**100.00**	**100.00**	

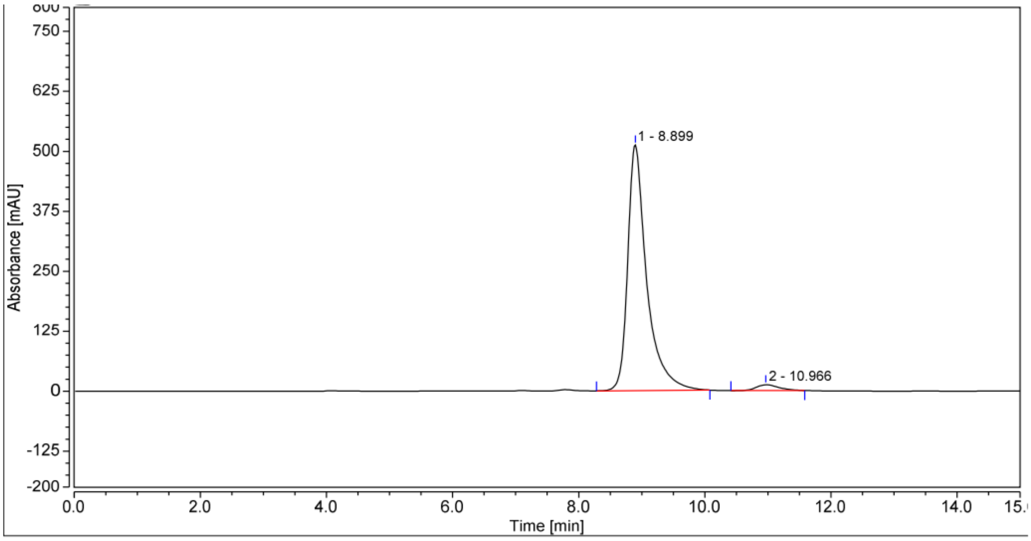

Integration Results							
No.	Peak Name	Retention Time min	Area mAU*min	Height mAU	Relative Area %	Relative Height %	Amount n.a.
1		8.899	178.543	512.226	97.24	97.68	n.a.
2		10.966	5.066	12.179	2.76	2.32	n.a.
**Total:**			**183.610**	**524.405**	**100.00**	**100.00**	

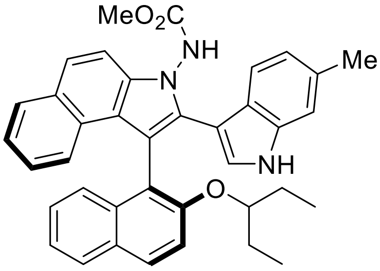

(*S*)-Methyl-(2-(6-methyl-1H-indol-3-yl)-1-(2-(pentan-3-yloxy)naphthalen-1-yl)-3H-benzo[e]indol-3-yl)carbamate (15k)

Brown solid (110 mg, 95% yield), mp 136.0-137.0 ℃; $[\alpha]_D^{24}$ -27.8 (*c* 1.0, $CHCl_3$, 97% ee); IR (KBr): 3419, 2921, 1625, 1456, 1238, 802 cm^{-1}; ^1H NMR (600 MHz, $CDCl_3$, 60 °C) δ 7.82 (d, *J* = 8.1 Hz, 1H), 7.77 (d, *J* = 9.0 Hz, 1H), 7.73 (d, *J* = 8.1 Hz, 1H), 7.65 (dd, *J* = 31.3, 8.8 Hz, 4H), 7.38 – 7.25 (m, 2H), 7.21 (dd, *J* = 14.9, 7.3 Hz, 5H), 7.14 (d, *J* = 6.8 Hz, 1H), 6.96 (t, *J* = 7.6 Hz, 1H), 6.86 (s, 1H), 6.82 (d, *J* = 8.1 Hz, 1H), 4.01 (s, 1H), 3.61 (s, 3H), 2.32 (s, 3H), 1.34 (s, 2H), 1.27 – 1.07 (m, 2H), 0.55 (s, 3H), 0.34 (t, *J* = 7.4 Hz, 3H);^{13}C NMR (100 MHz, $CDCl_3$) δ 155.9, 154.5, 135.9, 135.6, 133.9, 132.3, 131.4, 130.1, 129.2, 128.8, 128.4, 128.3, 127.6, 126.3, 125.9, 125.4, 124.9, 124.7, 123.7, 123.5, 123.1, 122.9, 122.3, 121.6, 120.6, 118.5, 111.1, 110.5, 110.3, 105.0, 82.0, 53.0, 25.6, 21.3, 9.2, 8.6; HRMS (ESI) calcd for $C_{38}H_{35}N_3O_3Na$ *m/z* $[M + Na]^+$: 604.2571; found: 604.2575; HPLC (Daicel Chiralpak

IA, *i*-PrOH/hexane = 20/80, flow rate 0.8 mL/min, λ = 230 nm): t_1 (major) = 8.6 min, t_2 (minor) = 10.8 min.

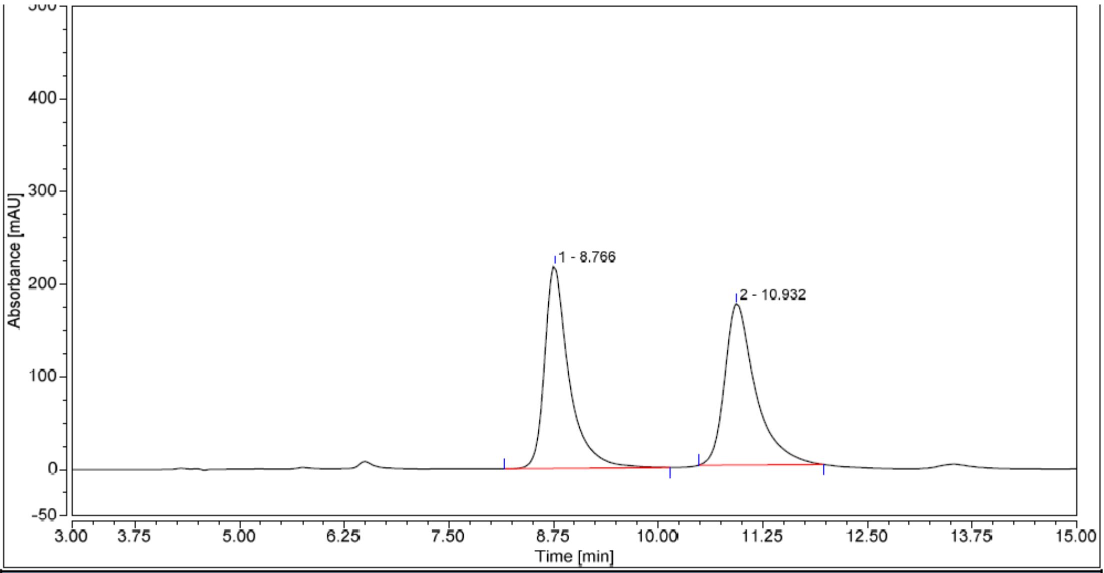

Integration Results

No.	Peak Name	Retention Time min	Area mAU*min	Height mAU	Relative Area %	Relative Height %	Amount n.a.
1		8.766	73.940	218.348	49.87	55.65	n.a.
2		10.932	74.324	173.981	50.13	44.35	n.a.
**Total:**			**148.265**	**392.329**	**100.00**	**100.00**	

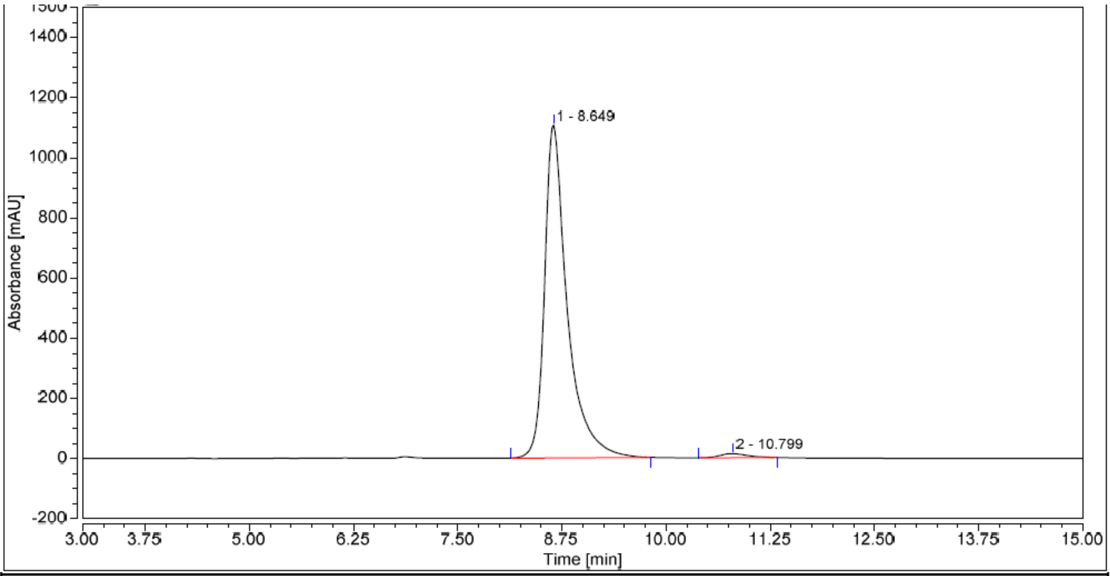

Integration Results

No.	Peak Name	Retention Time min	Area mAU*min	Height mAU	Relative Area %	Relative Height %	Amount n.a.
1		8.649	349.063	1105.595	98.53	98.77	n.a.
2		10.799	5.223	13.764	1.47	1.23	n.a.
**Total:**			**354.287**	**1119.358**	**100.00**	**100.00**	

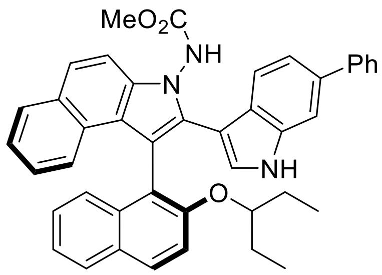

(*S*)-Methyl-(1-(2-(pentan-3-yloxy)naphthalen-1-yl)-2-(6-phenyl-1H-indol-3-yl)-3H-benzo[e]indol-3-yl)carbamate (15l)

Brown solid (90 mg, 70% yield), mp 137.0-138.0 ℃; $[\alpha]_D^{24}$ -17.4 (*c* 1.0, $CHCl_3$, 97% ee); IR (KBr): 3406, 2921, 2360, 1623, 1261, 754 cm^{-1}; ^1H NMR (400 MHz, $CDCl_3$) δ 7.95 (s, 1H), 7.82 (td, *J* = 16.7, 14.2, 8.0 Hz, 3H), 7.74 – 7.63 (m, 3H), 7.47 (s, 3H), 7.36 (s, 3H), 7.31 – 7.17 (m, 9H), 6.99 (t, *J* = 7.5 Hz, 1H), 4.00 (s, 1H), 3.67 (s, 3H), 1.23 (m, 4H), 0.55 (s, 3H), 0.35 (s, 3H); ^{13}C NMR (100 MHz,) δ 155.9, 154.4, 135.5, 133.9, 132.8, 131.3, 131.2, 130.1, 129.0, 128.9, 128.6, 128.5, 128.4, 127.7, 127.6, 126.4, 126.2, 125.7, 125.5, 123.8, 123.6, 123.2, 123.1, 121.4, 121.2, 121.0, 120.8, 120.7, 120.5, 118.1, 116.6, 111.4, 111.3, 111.2, 110.2, 106.9, 81.7, 53.0, 25.7, 9.3, 8.7; HRMS (ESI) calcd for $C_{43}H_{37}N_3O_3Na$ *m/z* $[M + Na]^+$: 666.2727; found: 666.2730; HPLC (Daicel Chiralpak IA, *i*-PrOH/hexane = 20/80, flow rate 0.8 mL/min, λ = 230 nm): t_1 (major) = 11.1 min, t_2 (minor) = 15.5 min.

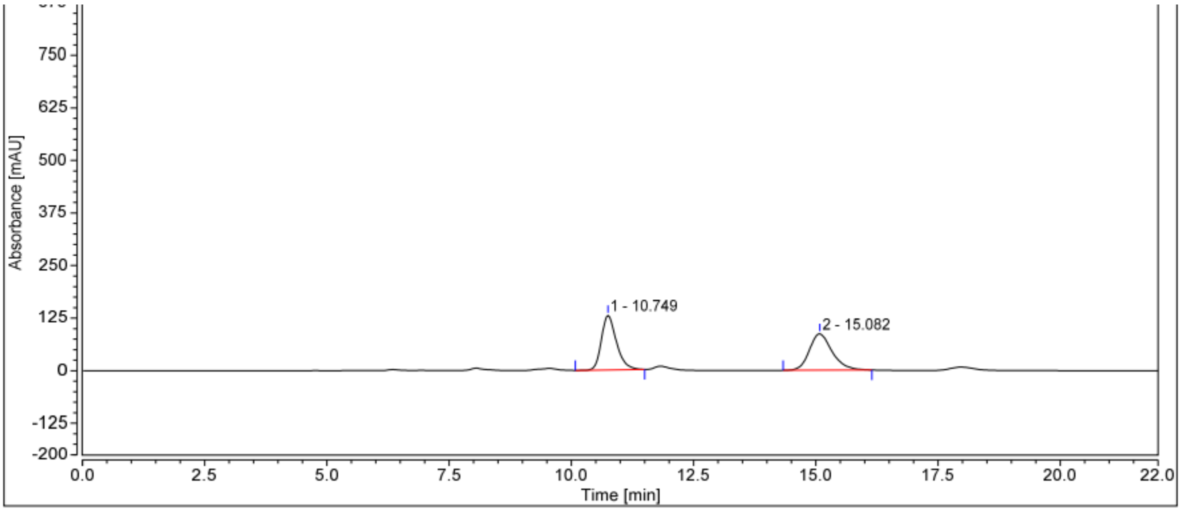

Integration Results							
No.	Peak Name	Retention Time min	Area mAU*min	Height mAU	Relative Area %	Relative Height %	Amount n.a.
1		10.749	46.320	129.645	49.67	59.90	n.a.
2		15.082	46.928	86.793	50.33	40.10	n.a.
**Total:**			**93.248**	**216.438**	**100.00**	**100.00**	

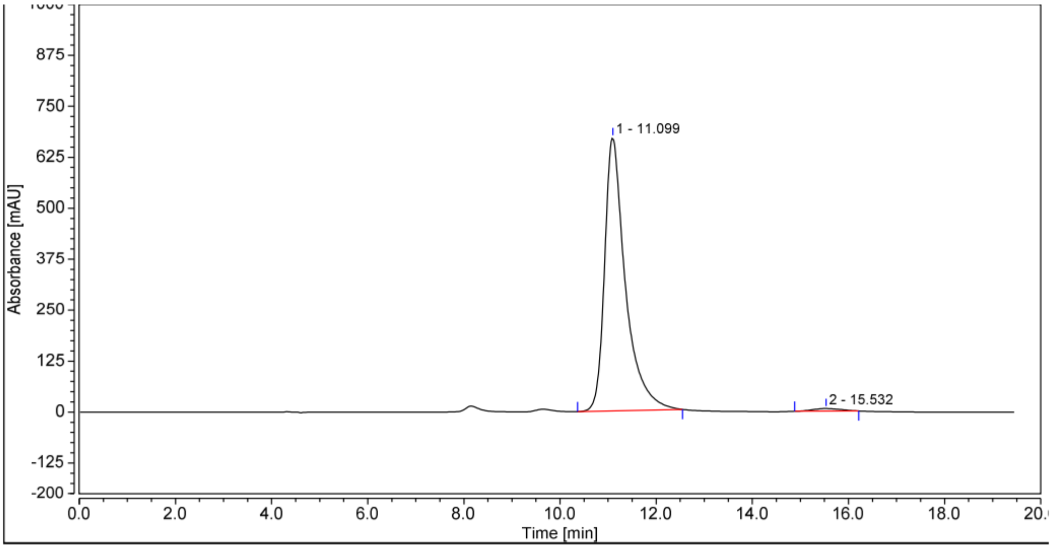

Integration Results							
No.	Peak Name	Retention Time min	Area mAU*min	Height mAU	Relative Area %	Relative Height %	Amount n.a.
1		11.099	336.285	670.119	98.75	99.07	n.a.
2		15.532	4.271	6.269	1.25	0.93	n.a.
**Total:**			**340.556**	**676.388**	**100.00**	**100.00**	

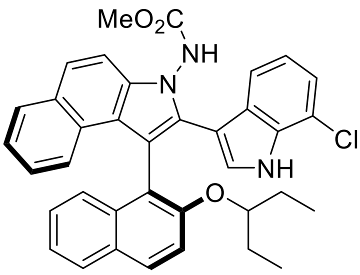

(*S*)-Methyl-2-(7-chloro-1H-indol-3-yl)-1-(2-(pentan-3-yloxy)naphthalen-1-yl)-3H-benzo[e]indol-3-yl)carbamate (15m)

Brown solid (104 mg, 87% yield), mp 146.0-147.0 ℃; $[\alpha]_D^{24}$ -49.2 (*c* 1.0, $CHCl_3$, 98% ee); IR (KBr): 3413, 2960, 1648, 1342, 762 cm^{-1}; ^1H NMR (400 MHz, $CDCl_3$) δ 8.16 (s, 1H), 7.92 (d, *J* = 8.0 Hz, 1H), 7.89 – 7.78 (m, 2H), 7.66-7.76 (m, 4H), 7.57 – 7.43 (m, 1H), 7.40 – 7.27 (m, 4H), 7.20 – 6.79 (m, 5H), 4.08 (s, 1H), 3.63 (s, 3H), 1.29-1.37 (m, 4H), 0.41-0.61 (m, 6H); ^{13}C NMR (100 MHz, $CDCl_3$) δ 155.9, 154.3, 135.5, 133.9, 132.8, 131.2, 130.1, 129.0, 128.9, 128.6, 128.5, 128.4, 127.6, 126.3, 125.7, 125.5, 123.8, 123.5, 123.2, 123.0, 121.3, 120.8, 118.1, 116.6, 111.2, 110.2, 106.9, 81.7, 53.0, 25.7, 9.3, 8.6; HRMS (ESI) calcd for $C_{37}H_{32}N_3O_3Na$ *m/z* $[M + Na]^+$: 624.2024; found: 624.2022; HPLC (Daicel Chiralpak IF, *i*-PrOH/hexane = 10/90, flow rate 0.6 mL/min, *λ* = 260 nm): t_1 (minor) = 14.7 min, t_2 (major) = 16.4 min.

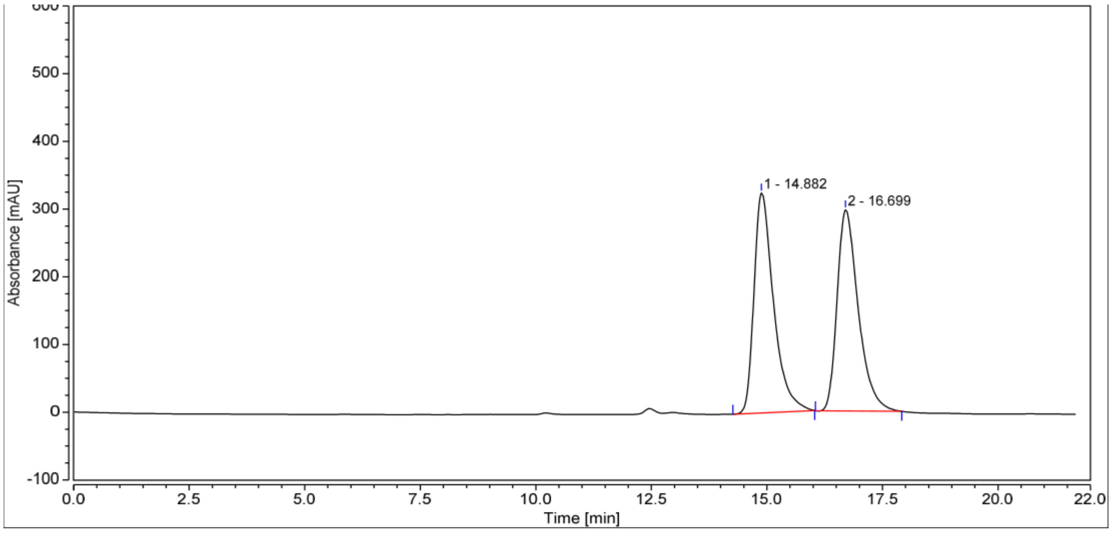

Integration Results

No.	Peak Name	Retention Time min	Area mAU*min	Height mAU	Relative Area %	Relative Height %	Amount n.a.
1		14.882	159.653	324.707	50.31	52.23	n.a.
2		16.699	157.662	296.998	49.69	47.77	n.a.
**Total:**			**317.314**	**621.704**	**100.00**	**100.00**	

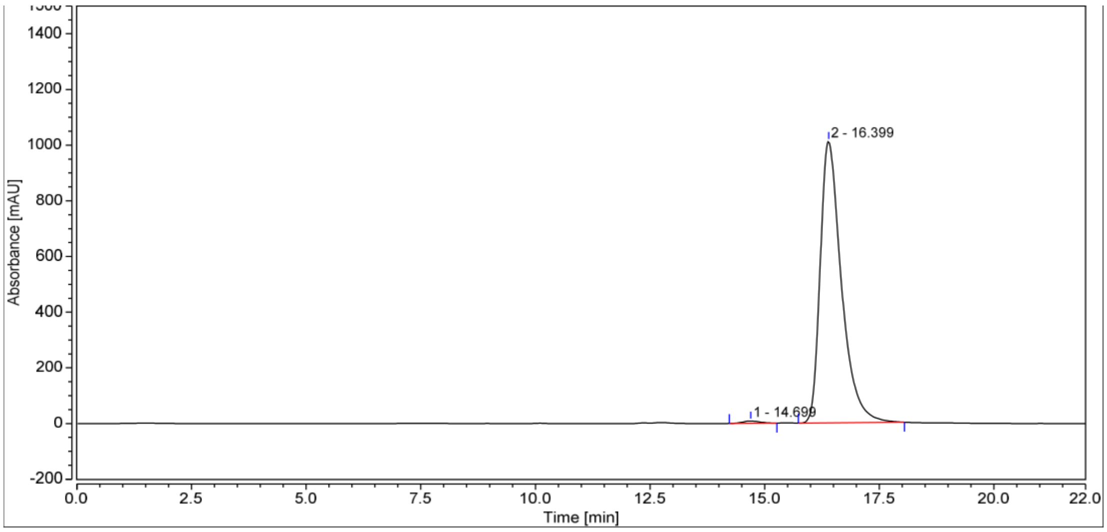

Integration Results							
No.	Peak Name	Retention Time min	Area mAU*min	Height mAU	Relative Area %	Relative Height %	Amount n.a.
1		14.699	3.539	8.153	0.65	0.80	n.a.
2		16.399	542.816	1011.345	99.35	99.20	n.a.
Total:			546.355	1019.499	100.00	100.00	

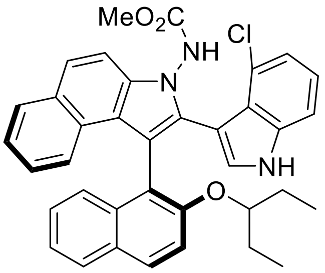

(*S*)-Methyl-2-(7-chloro-1H-indol-3-yl)-1-(2-(pentan-3-yloxy)naphthalen-1-yl)-3H-benzo[e]indol-3-yl)carbamate (15n)

Brown solid (87 mg, 72% yield), mp 149.0-150.0 ℃; $[\alpha]_D^{24}$ -37.6 (*c* 1.0, $CHCl_3$, dr = 1:2, 99% ee, 99% ee); IR (KBr): 3415, 2923, 1640, 1458, 1242, 806 cm^{-1}; ^1H NMR (400 MHz, $CDCl_3$) δ 8.01 (d, *J* = 39.5 Hz, 1H), 7.92 – 7.77 (m, 3H), 7.72 (dd, *J* = 18.4, 8.4 Hz, 4H), 7.40 (dd, *J* = 56.8, 5.5 Hz, 2H), 7.27 – 7.15 (m, 3H), 7.15 – 7.06 (m, 1H), 7.07 – 6.89 (m, 3H), 6.84 (s, 1H), 4.21 – 3.91 (m, 1H), 3.54 (dd, *J* = 38.0, 30.8 Hz, 3H), 1.24 (ddd, *J* = 13.4, 12.7, 6.9 Hz, 4H), 0.79 – 0.10 (m, 6H); ^{13}C NMR (100 MHz, $CDCl_3$) δ 155.2, 154.2*, 136.9, 136.4*, 134.8, 133.3*, 133.1, 131.3, 131.1*, 130.0, 129.5, 128.9, 128.6, 128.4, 128.3*, 128.3*, 127.6*, 127.3, 125.8*, 125.5*, 125.4*, 126.5, 126.2, 125.8*, 125.5, 125.4, 124.6*, 124.5, 124.1, 124.0*, 123.7, 123.5, 123.3, 123.1, 123.0, 122.9, 122.8*, 122.3, 122.3*, 120.8, 120.4, 120.2, 119.7, 116.1, 111.7, 111.2*, 110.3, 110.1*, 103.9*, 83.0, 80.6*, 53.1*, 52.9, 26.1, 25.9*, 25.4, 9.4, 90.9*, 8.8, 8.3*; *Peaks from the isomer; HRMS (ESI) calcd for $C_{37}H_{32}ClN_3O_3$ *m/z* $[M + Na]^+$:

624.2024; found: 624.2019; HPLC (Daicel Chiralpak IA, *i*-PrOH/hexane = 20/80, flow rate 0.6 mL/min, λ = 260 nm): t_1 (major) = 8.6 min, t_2 (minor) = 9.2 min, t_3 (major) = 9.8 min, t_4 (minor) = 10.4 min.

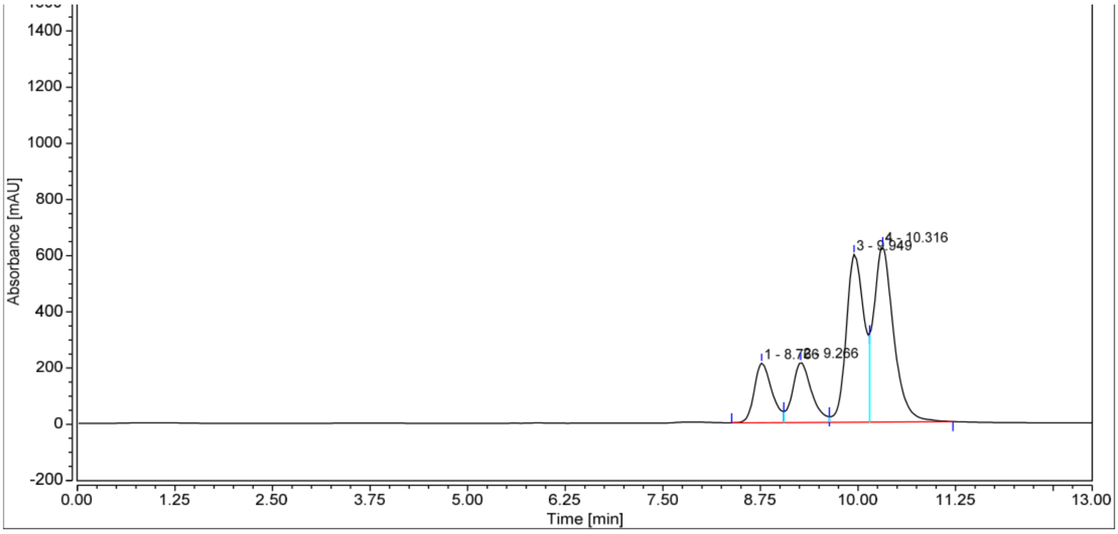

Integration Results							
No.	Peak Name	Retention Time<br>min	Area<br>mAU*min	Height<br>mAU	Relative Area<br>%	Relative Height<br>%	Amount<br>n.a.
1		8.766	55.291	211.386	12.04	12.84	n.a.
2		9.266	60.215	213.467	13.12	12.97	n.a.
3		9.949	159.022	596.707	34.64	36.25	n.a.
4		10.316	184.527	624.523	40.20	37.94	n.a.
**Total:**			**459.056**	**1646.083**	**100.00**	**100.00**	

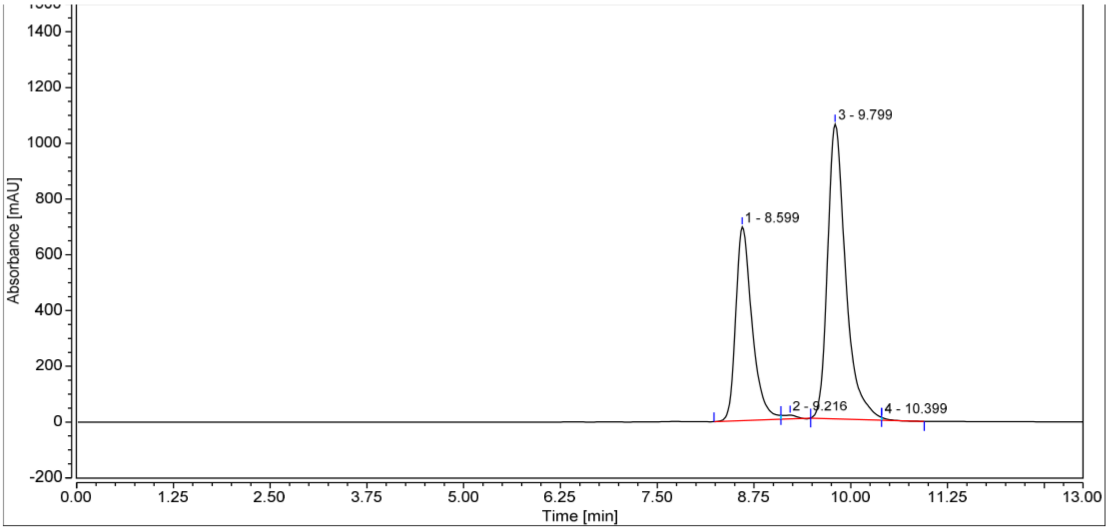

Integration Results							
No.	Peak Name	Retention Time<br>min	Area<br>mAU*min	Height<br>mAU	Relative Area<br>%	Relative Height<br>%	Amount<br>n.a.
1		8.599	169.885	695.584	36.83	39.17	n.a.
2		9.216	2.581	13.726	0.56	0.77	n.a.
3		9.799	288.284	1056.928	62.49	59.52	n.a.
4		10.399	0.578	9.620	0.13	0.54	n.a.
**Total:**			**461.329**	**1775.858**	**100.00**	**100.00**	

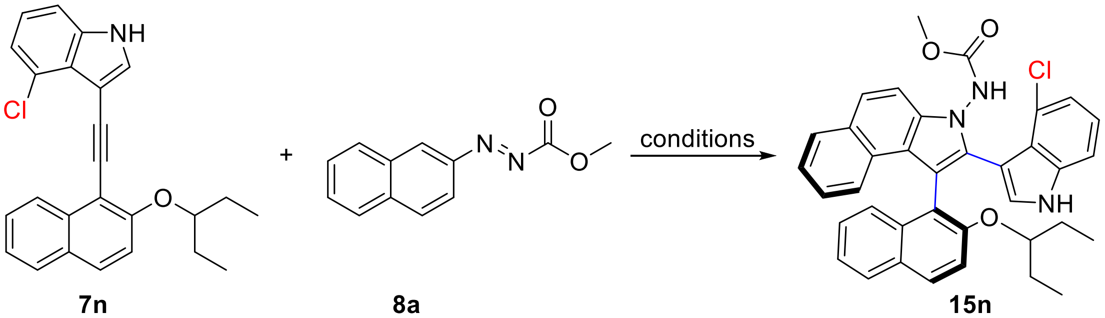

7n **8a** **15n**

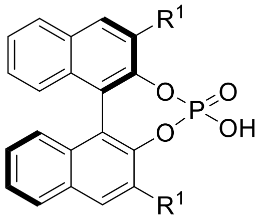

CPA 2: R^1 = 9-anthryl
CPA 3: R^1 = 9-phenanthryl
CPA 8: R^1 = 2,4,6-^{i}Pr$_3$C$_6$H$_2$
CPA 9: R^1 = 3,5-(CF$_3$)$_2$C$_6$H$_3$

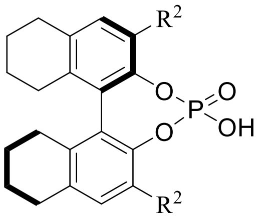

CPA 4: R^2 = 9-anthryl
CPA 10: R^2 = 2,4,6-^{i}Pr$_3$C$_6$H$_2$

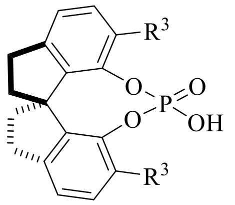

CPA 5: R^3 = 9-anthryl
CPA 6: R^3 = 9-phenanthryl
CPA 7: R^3 = 2,4,6-^{i}Pr$_3$C$_6$H$_2$
CPA 11: R^3 = 2,4,6-Me$_3$C$_6$H$_2$

Entry	Cat	Solvent	T (°C)	Time (h)	Yield (%)	dr	ee (%)
1	**CPA 6**	CHCl$_3$	-50	82	72	1:2	99, 99
2	**CPA 5**	CHCl$_3$	-50	82	26	7:2	93, 40
3	**CPA 4**	CHCl$_3$	-50	82	76	2:8	31, -90
4	**CPA 2**	CHCl$_3$	-50	82	95	3:7	-43, -75
5	**CPA 7**	CHCl$_3$	-50	82	NR	-	-,-
6	**CPA 8**	CHCl$_3$	-50	82	20	7:2	-81，-33
7	**CPA 9**	CHCl$_3$	-50	82	63	3:2	-67，-23
8	**CPA 10**	CHCl$_3$	-50	82	NR	-	-， -
9	**CPA 11**	CHCl$_3$	-50	82	53	8:1	91，-25

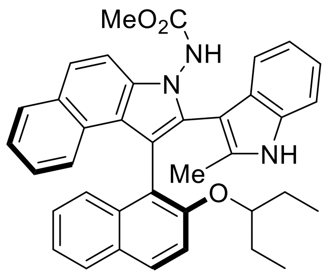

(*S*)-Methyl-2-(7-chloro-1H-indol-3-yl)-1-(2-(pentan-3-yloxy)naphthalen-1-yl)-3H-benzo[e]indol-3-yl)carbamate (15o)

Brown solid (96 mg, 82% yield), mp 151.0-152.0 ℃; $[\alpha]_D^{24}$ -49.2 (*c* 1.0, $CHCl_3$, dr = 7:3, < 5% ee); IR (KBr): 3320, 2860, 1648, 1342, 842 cm^{-1}; ^1H NMR (400 MHz, $CDCl_3$) δ 7.90 (d, *J* = 8.1 Hz, 1H), 7.88 – 7.57 (m, 5H), 7.46 (d, *J* = 8.0 Hz, 1H), 7.44 – 7.23 (m, 4H), 7.18 (d, *J* = 9.0 Hz, 2H), 7.12 – 6.94 (m, 3H), 6.83 (dd, *J* = 54.8, 46.4 Hz, 2H), 4.13 (dd, *J* = 94.3, 4.6 Hz, 1H), 3.55 (s, 3H), 1.57 (s, 3H), 1.32 – 1.07 (m, 4H), 0.65 (dt, *J* = 13.0, 6.8 Hz, 3H), 0.31 (dd, *J* = 21.1, 14.0 Hz, 3H); ^{13}C NMR (100 MHz, $CDCl_3$) δ 154.3, 135.0, 134.9, 134.8*, 134.0, 132.1, 130.1, 129.9*, 129.2, 128.9*, 128.8, 128.7, 128.7*, 128.5, 128.3, 128.2*, 127.6, 127.3, 126.1, 125.7, 125.2, 125.1*, 123.4, 123.2, 123.1*, 122.8, 120.9, 120.3, 119.7, 118.6, 116.8, 115.3, 112.5, 112.2, 110.3, 110.2, 102.5, 102.1, 81.3, 52.8, 25.9, 25.6, 12.2, 9.6, 8.4; *Peaks from the isomer; HRMS (ESI) calcd for $C_{38}H_{35}N_3O_3Na$ *m/z* $[M + Na]^+$: 604.2571; found: 604.2569; HPLC (Daicel Chiralpak IF, *i*-PrOH/hexane = 10/80, flow rate 0.8 mL/min, λ = 260 nm): t_1 = 12.7 min, t_2 =13.5 min, t_3 = 16.4 min, t_4 = 17.7 min.

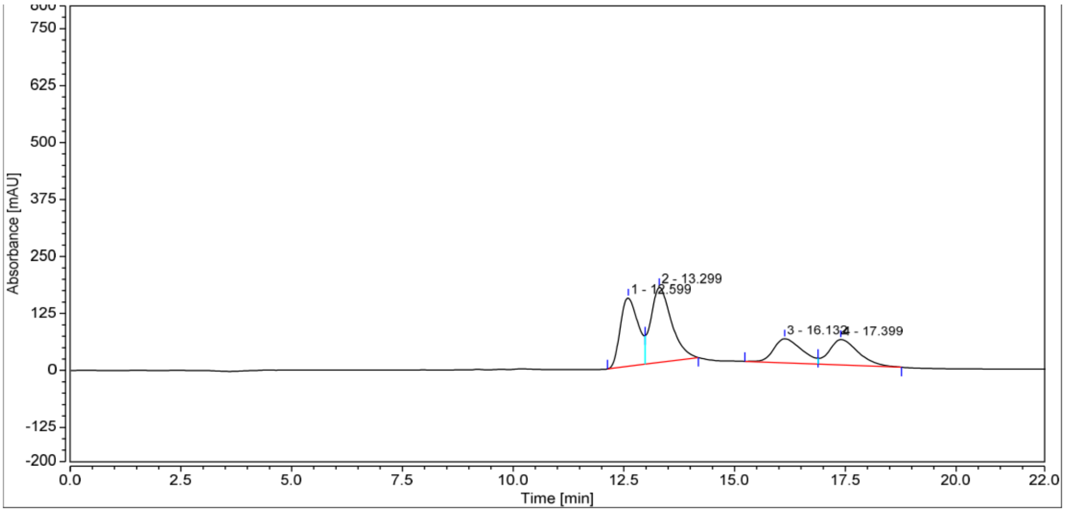

Integration Results							
No.	Peak Name	Retention Time min	Area mAU*min	Height mAU	Relative Area %	Relative Height %	Amount n.a.
1		12.599	70.376	150.010	29.13	35.42	n.a.
2		13.299	89.405	164.824	37.00	38.91	n.a.
3		16.132	39.210	52.882	16.23	12.49	n.a.
4		17.399	42.626	55.841	17.64	13.18	n.a.
**Total:**			**241.617**	**423.557**	**100.00**	**100.00**	

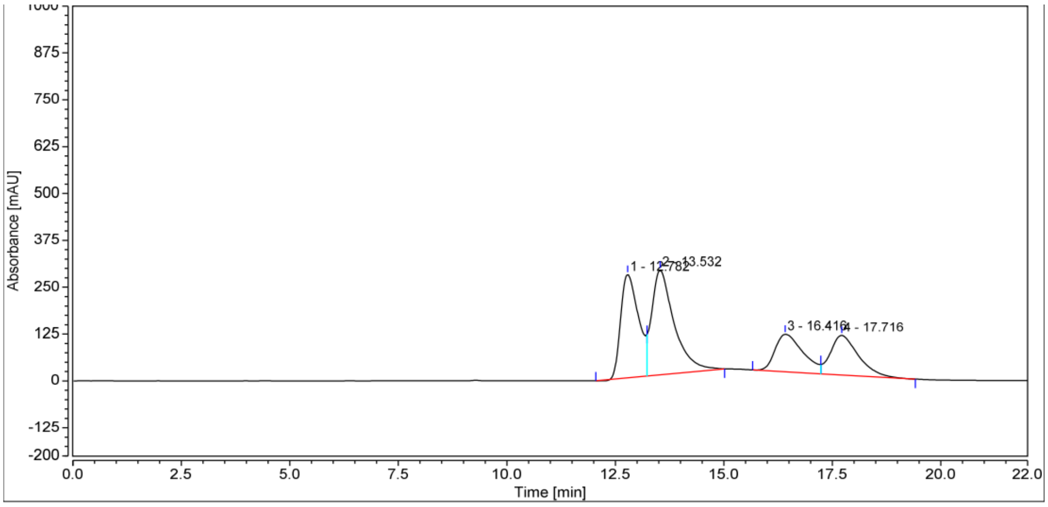

Integration Results							
No.	Peak Name	Retention Time min	Area mAU*min	Height mAU	Relative Area %	Relative Height %	Amount n.a.
1		12.782	131.974	275.923	29.11	36.27	n.a.
2		13.532	167.727	279.239	37.00	36.71	n.a.
3		16.416	74.866	99.952	16.52	13.14	n.a.
4		17.716	78.724	105.542	17.37	13.88	n.a.
**Total:**			**453.290**	**760.654**	**100.00**	**100.00**	

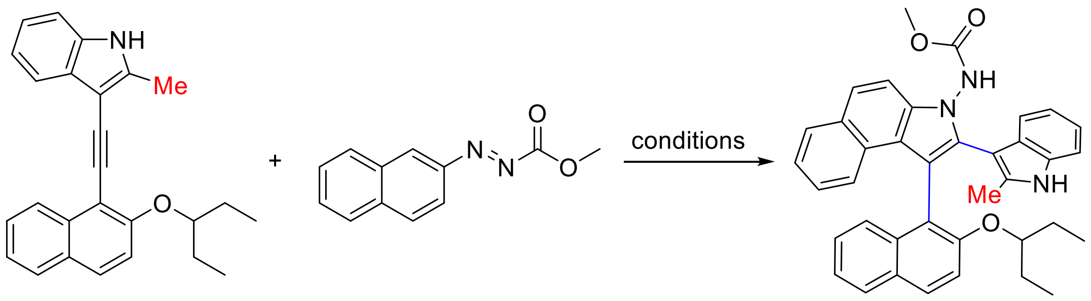

7o **8a** **15o**

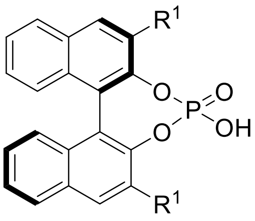

CPA 2: R^1 = 9-anthryl
CPA 3: R^1 = 9-phenanthryl
CPA 8: R^1 = 2,4,6-$^i Pr_3C_6H_2$
CPA 9: R^1 = 3,5-$(CF_3)_2C_6H_3$

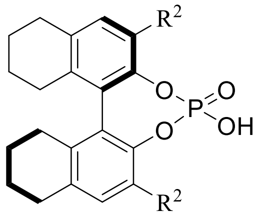

CPA 4: R^2 = 9-anthryl
CPA 10: R^2 = 2,4,6-$^i Pr_3C_6H_2$

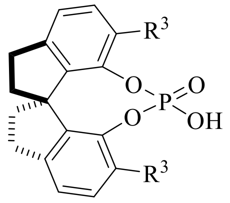

CPA 5: R^3 = 9-anthryl
CPA 6: R^3 = 9-phenanthryl
CPA 7: R^3 = 2,4,6-$^i Pr_3C_6H_2$
CPA 11: R^3 = 2,4,6-$Me_3C_6H_2$

Entry	Cat	Solvent	T (ºC)	Time (h)	Yield (%)	dr	ee (%)
1	**CPA 6**	$CHCl_3$	-50	82	82	7:3	<5, 0
2	**CPA 5**	$CHCl_3$	-50	82	57	7:3	0, 35
3	**CPA 4**	$CHCl_3$	-50	82	60	7:3	35, 20
4	**CPA 2**	$CHCl_3$	-50	82	43	7:3	25, 17
5	**CPA 7**	$CHCl_3$	-50	82	NR	-	-, -
6	**CPA 8**	$CHCl_3$	-50	82	55	7:3	-7,-11
7	**CPA 9**	$CHCl_3$	-50	82	95	7:3	9,11
8	**CPA 10**	$CHCl_3$	-50	82	41	7:3	-7,-9
9	**CPA 11**	$CHCl_3$	-50	82	74	7:3	<5,<5

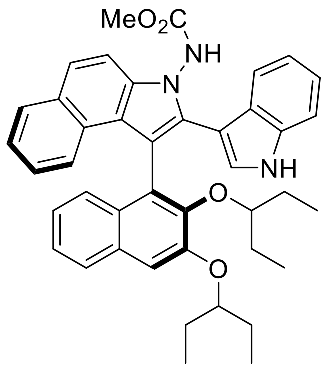

(*S*)-Methyl -(2,3-bis(pentan-3-yloxy)naphthalen-1-yl)-2-(1H-indol-3-yl)-3H-benzo [e]indol-3-yl)carbamate (15p)

Brown solid (104 mg, 80% yield), mp 137.0-138.0 ℃; $[\alpha]_D^{24}$ -37.6 (*c* 1.0, $CHCl_3$, 93% ee); IR (KBr): 3426, 2960, 1468, 1342, 842 cm^{-1}; ^1H NMR (400 MHz, $CDCl_3$) δ 8.12 (s, 1H), 7.89 (d, *J* = 8.0 Hz, 1H), 7.70 (dd, *J* = 26.4, 8.8 Hz, 3H), 7.52–7.38 (m, 3H), 7.28 (dt, *J* = 7.1, 5.9 Hz, 4H), 7.17 (s, 1H), 7.03 (dd, *J* = 13.9, 6.9 Hz, 5H), 4.32– 4.24 (m, 1H), 3.98–3.89 (m, 1H), 3.62 (s, 3H), 1.78–1.71 (m, 2H), 1.68–1.61 (m, 2H), 1.52–1.48(m, 2H), 1.22–1.10(m, 2H), 0.98–1.09 (m, 3H), 0.88–0.95 (m, 3H), 0.31–0.62 (m, 3H), 0.29–0.19 (m, 3H); ^{13}C NMR (100 MHz, $CDCl_3$) δ 151.7, 147.9, 135.6, 130.6, 130.3, 128.4, 128.2, 127.0, 126.7, 126.2, 125.5, 124.6, 123.9, 123.7, 123.5, 123.0, 121.9, 120.2, 118.9, 111.3, 110.8, 110.3, 109.6, 105.4, 84.6, 80.4, 53.0, 29.7, 25.5, 9.7, 9.3, 9.2; HRMS (ESI) calcd for $C_{42}H_{43}N_3O_4Na$ *m/z* $[M + Na]^+$: 676.3146; found: 676.3152; HPLC (Daicel Chiralpak IA, *i*-PrOH/hexane = 20/80, flow rate 0.8 mL/min, λ = 260 nm): t_1 (major) =12.2 min, t_2 (minor) =15.6 min.

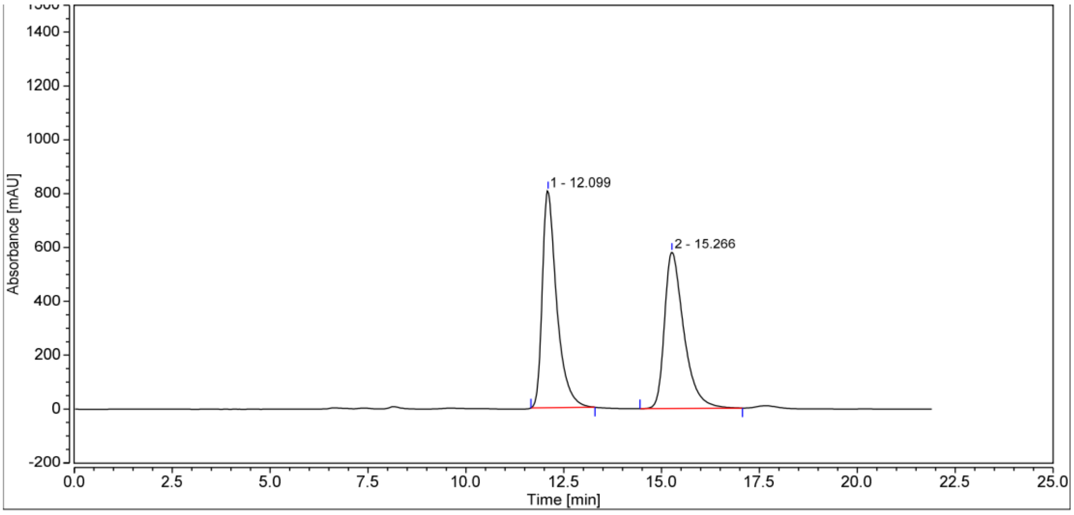

Integration Results							
No.	Peak Name	Retention Time min	Area mAU*min	Height mAU	Relative Area %	Relative Height %	Amount n.a.
1		12.099	344.085	805.684	50.85	58.12	n.a.
2		15.266	332.554	580.525	49.15	41.88	n.a.
**Total:**			**676.639**	**1386.209**	**100.00**	**100.00**	

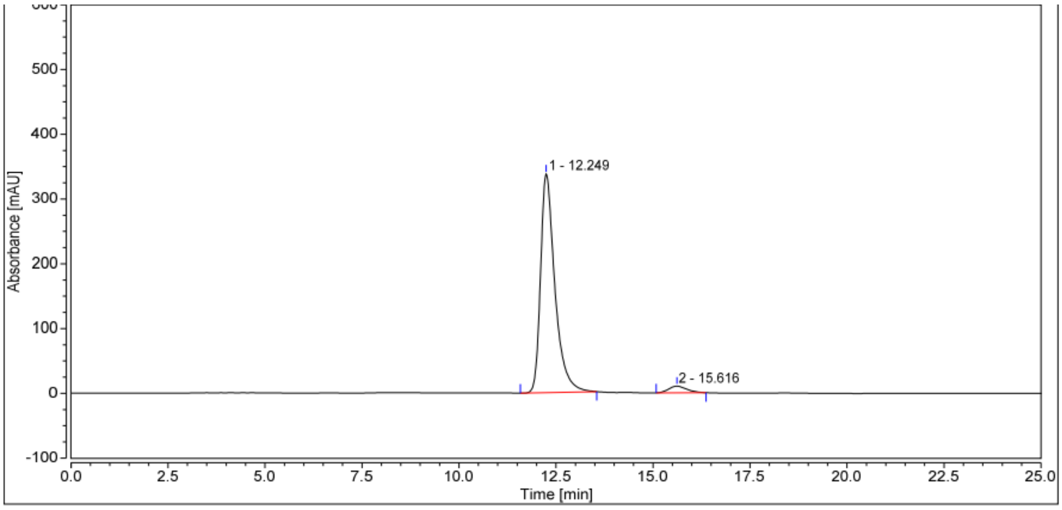

Integration Results							
No.	Peak Name	Retention Time min	Area mAU*min	Height mAU	Relative Area %	Relative Height %	Amount n.a.
1		12.249	144.657	337.609	96.34	97.06	n.a.
2		15.616	5.499	10.224	3.66	2.94	n.a.
**Total:**			**150.156**	**347.833**	**100.00**	**100.00**	

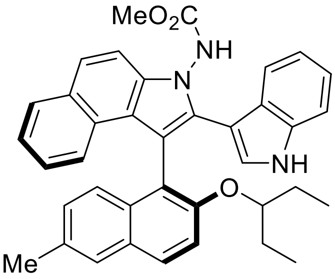

(*S*)-Methyl-(2-(1H-indol-3-yl)-1-(6-methyl-2-(pentan-3-yloxy)naphthalen-1-yl)-3H-benzo[e]indol-3-yl)carbamate (15q)

Brown solid (114 mg, 98% yield), mp 128.0-129.0 ℃; $[\alpha]_D^{23}$ -22.0 (*c* 1.0, $CHCl_3$, 97% ee); IR (KBr): 3411, 2974, 1730, 1380, 1256, 754 cm^{-1}; ^1H NMR (600 MHz, $CDCl_3$, 60 ℃) δ 7.87 (s, 1H), 7.83 (d, *J* = 8.0 Hz, 1H), 7.69 (dd, *J* = 8.9, 4.4 Hz, 2H), 7.63 (d, *J* = 8.8 Hz, 1H), 7.51 (s, 2H), 7.47 (d, *J* = 6.9 Hz, 1H), 7.29 (s, 1H), 7.26 – 7.22 (m, 1H), 7.22 – 7.13 (m, 4H), 7.07 (t, *J* = 7.5 Hz, 1H), 7.02 (d, *J* = 7.5 Hz, 1H), 6.98 (dd, *J* = 15.0, 6.9 Hz, 2H), 3.97 (s, 1H), 3.61 (s, 3H), 2.37 (s, 3H), 1.32 (s, 2H), 1.25 – 1.12 (m, 2H), 0.53 (s, 3H), 0.33 (t, *J* = 7.4 Hz, 3H); ^{13}C NMR (100 MHz, $CDCl_3$) δ 155.9, 154.7, 135.8, 135.5, 133.9, 131.8, 130.1, 128.8, 128.6, 128.5, 128.3, 127.4, 127.0, 125.9, 125.4, 124.8, 123.5, 123.3, 123.2, 122.9, 121.8, 121.1, 120.7, 120.0, 119.0, 117.5, 117.3, 111.2, 110.9, 110.3, 105.6, 81.5, 53.0, 25.7, 21.8, 9.2, 8.7; HRMS (ESI) calcd for $C_{38}H_{35}N_3O_3Na$ *m/z* $[M + Na]^+$: 604.2571; found: 604.2572; HPLC (Daicel Chiralpak IA, *i*-PrOH/hexane = 7/93, flow rate 0.8 mL/min, λ = 260 nm): t_1 (major) = 26.1 min, t_2 (minor) = 42.4 min.

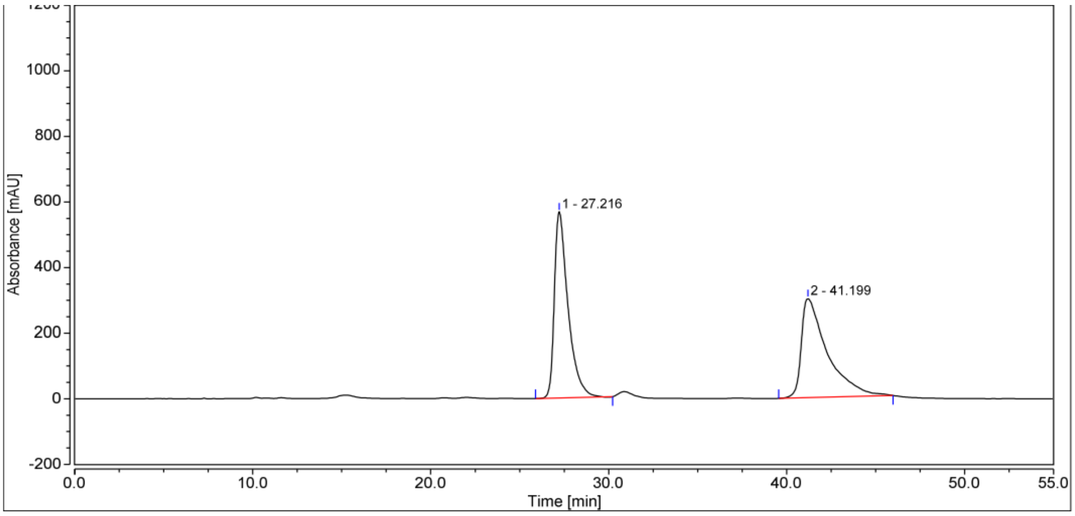

Integration Results

No.	Peak Name	Retention Time min	Area mAU*min	Height mAU	Relative Area %	Relative Height %	Amount n.a.
1		27.216	504.643	566.961	49.69	65.26	n.a.
2		41.199	511.035	301.776	50.31	34.74	n.a.
**Total:**			**1015.678**	**868.737**	**100.00**	**100.00**	

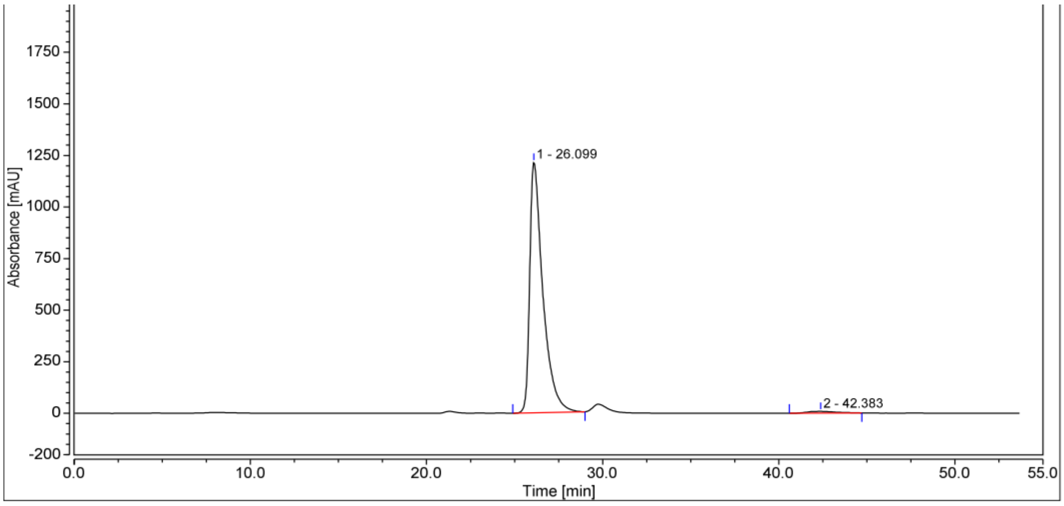

Integration Results							
No.	Peak Name	Retention Time min	Area mAU*min	Height mAU	Relative Area %	Relative Height %	Amount n.a.
1		26.099	1051.541	1213.909	98.53	99.32	n.a.
2		42.383	15.636	8.285	1.47	0.68	n.a.
**Total:**			**1067.178**	**1222.194**	**100.00**	**100.00**	

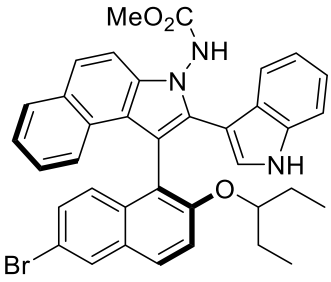

(*S*)-Methyl-(1-(6-bromo-2-(pentan-3-yloxy)naphthalen-1-yl)-2-(1H-indol-3-yl)-3H-benzo[e]indol-3-yl)carbamate (15r)

Brown solid (94 mg, 73% yield), mp 142.0-143.0 ℃; $[\alpha]_D^{23}$ -45.2 (*c* 1.0, $CHCl_3$, 98% ee); IR (KBr): 3420, 2966, 1459, 1240, 746 cm^{-1}; ^1H NMR (600 MHz, $CDCl_3$，60 °C) δ 7.92 (s, 1H), 7.88 (s, 1H), 7.83 (d, *J* = 8.1 Hz, 1H), 7.67 (t, *J* = 9.7 Hz, 2H), 7.62 (d, *J* = 8.8 Hz, 1H), 7.50 (s, 1H), 7.44 (d, *J* = 7.0 Hz, 1H), 7.32 (s, 1H), 7.23 (s, 1H), 7.23 – 7.21 (m, 1H), 7.21 – 7.11 (m, 3H), 7.07 (t, *J* = 7.6 Hz, 1H), 7.03 – 6.84 (m, 3H), 4.02 (s, 1H), 3.60 (s, 3H), 1.32 (s, 2H), 1.27 – 1.10 (m, 2H), 0.54 (s, 3H), 0.34 (t, *J* = 7.4 Hz, 3H); ^{13}C NMR (100 MHz, $CDCl_3$) δ 155.9, 154.9, 135.5, 134.0, 132.1, 130.6, 130.2, 129.5, 128.4, 128.4, 127.9, 127.7, 127.1, 125.5, 123.7, 123.1, 123.0, 122.0, 120.5, 120.1, 119.1, 117.3, 111.3, 110.3, 105.4, 81.5, 53.0, 26.3, 25.7, 9.2, 8.6; HRMS (ESI) calcd for $C_{37}H_{32}BrN_3O_3Na$ *m/z* $[M + Na]^+$: 668.1519; found: 668.1515;

HPLC (Daicel Chiralpak IA, *i*-PrOH/hexane = 20/80, flow rate 0.8 mL/min, λ = 230 nm): t_1 (major) = 8.3 min, t_2 (minor) = 9.6 min.

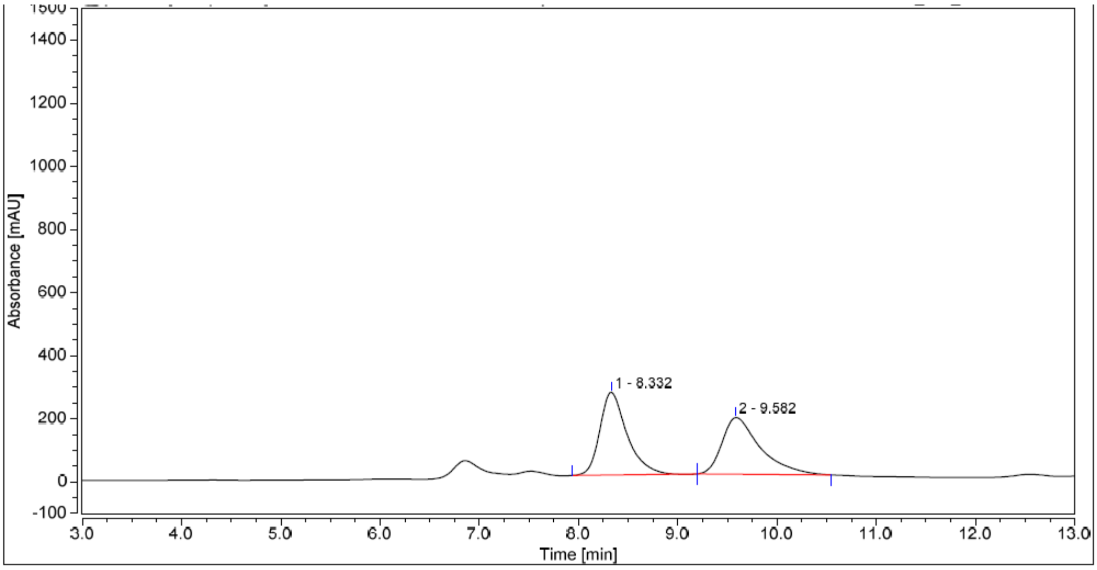

Integration Results							
No.	Peak Name	Retention Time min	Area mAU*min	Height mAU	Relative Area %	Relative Height %	Amount n.a.
1		8.332	82.928	262.090	50.93	59.34	n.a.
2		9.582	79.909	179.570	49.07	40.66	n.a.
**Total:**			**162.837**	**441.660**	**100.00**	**100.00**	

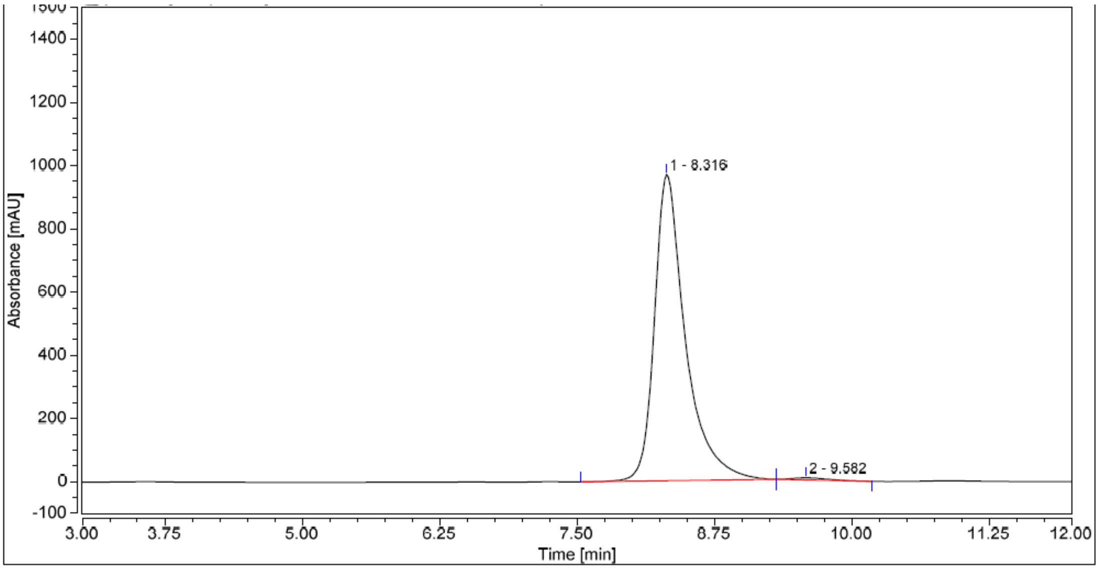

Integration Results							
No.	Peak Name	Retention Time min	Area mAU*min	Height mAU	Relative Area %	Relative Height %	Amount n.a.
1		8.316	315.411	968.206	99.25	99.31	n.a.
2		9.582	2.388	6.706	0.75	0.69	n.a.
**Total:**			**317.799**	**974.912**	**100.00**	**100.00**	

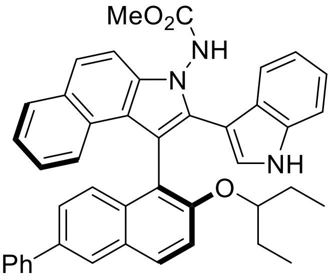

(*S*)-Methyl-(2-(1H-indol-3-yl)-1-(2-(pentan-3-yloxy)-6-phenylnaphthalen-1-yl)-3H-benzo[e]indol-3-yl)carbamate (15s)

Brown solid (126 mg, 98% yield), mp 150.0-151.0 ℃; $[\alpha]_D^{22}$ -42.8 (*c* 1.0, $CHCl_3$, 97% ee); IR (KBr): 3418, 2967, 1384, 1245, 748 cm^{-1}; ^1H NMR (600 MHz, $CDCl_3$, 60 °C) δ 7.95 (s, 1H), 7.91 (s, 1H), 7.84 (dd, *J* = 8.4, 6.0 Hz, 2H), 7.70 (d, *J* = 8.8 Hz, 2H), 7.64 (d, *J* = 8.8 Hz, 1H), 7.60 (d, *J* = 7.6 Hz, 2H), 7.49 (d, *J* = 7.5 Hz, 1H), 7.44 – 7.35 (m, 3H), 7.29 (dd, *J* = 16.2, 8.2 Hz, 3H), 7.26 – 7.22 (m, 2H), 7.21 (dd, *J* = 8.0, 1.1 Hz, 1H), 7.16 (d, *J* = 8.1 Hz, 1H), 7.07 (dd, *J* = 11.2, 4.0 Hz, 1H), 7.03 – 6.96 (m, 2H), 4.03 (s, 1H), 3.60 (s, 3H), 1.43 – 1.23 (m, 2H), 1.28 – 1.10 (m, 2H), 0.54 (s, 3H), 0.35 (t, *J* = 7.4 Hz, 3H); ^{13}C NMR (100 MHz, $CDCl_3$) δ 156.0, 154.8, 140.9, 136.0, 135.5, 134.9, 134.0, 132.1, 130.2, 129.3, 129.1, 128.7, 128.5, 128.4, 127.1, 127.0, 126.9, 126.4, 125.9, 125.5, 125.4, 123.6, 123.2, 123.0, 121.9, 120.7, 120.1, 119.1, 111.3, 110.8, 110.3, 105.5, 81.8, 53.0, 25.7, 9.2, 8.7; HRMS (ESI) calcd for $C_{43}H_{37}N_3O_3Na$ *m/z* $[M + Na]^+$: 666.2727; found: 666.2726; HPLC (Daicel Chiralpak IA, *i*-PrOH/hexane = 20/80, flow rate 0.8 mL/min, λ = 230 nm): t_1 (major) = 9.6 min, t_2 (minor) = 11.8 min.

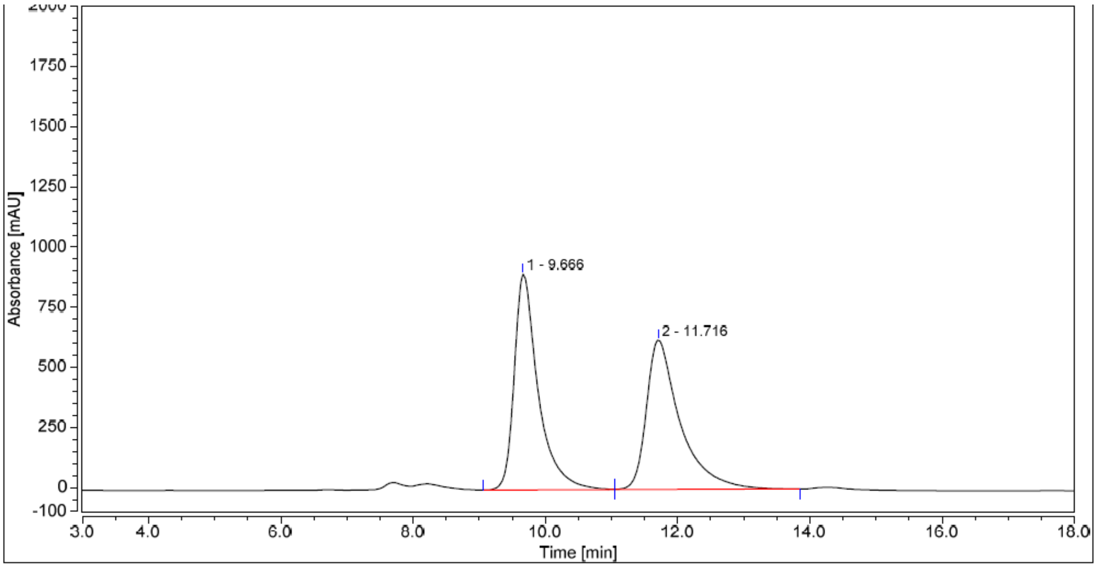

Integration Results							
No.	Peak Name	Retention Time min	Area mAU*min	Height mAU	Relative Area %	Relative Height %	Amount n.a.
1		9.666	377.268	897.097	50.70	59.08	n.a.
2		11.716	366.820	621.437	49.30	40.92	n.a.
**Total:**			**744.088**	**1518.534**	**100.00**	**100.00**	

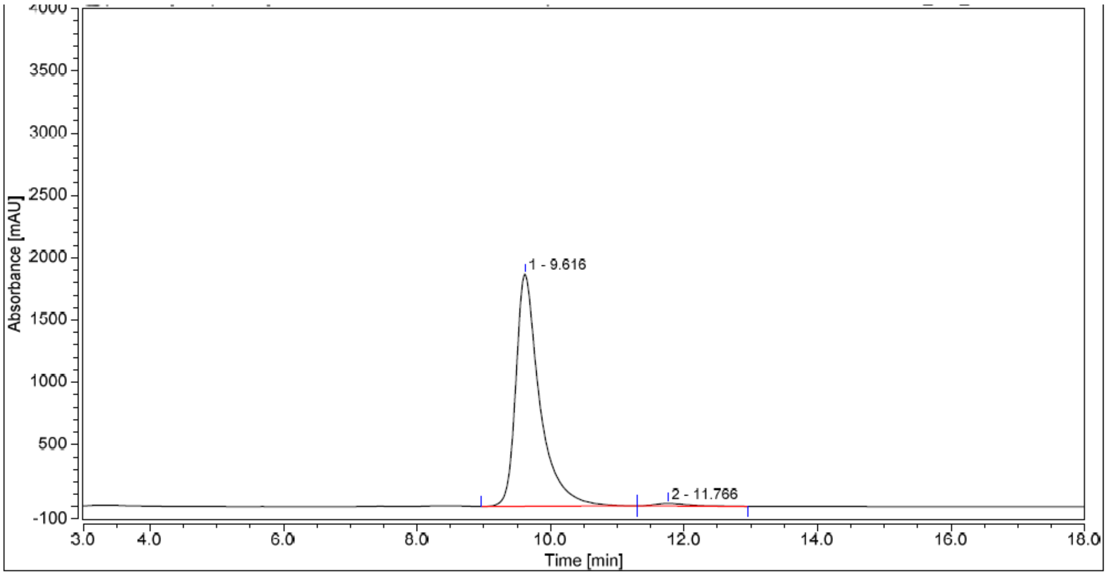

Integration Results							
No.	Peak Name	Retention Time min	Area mAU*min	Height mAU	Relative Area %	Relative Height %	Amount n.a.
1		9.616	772.369	1864.604	98.46	98.88	n.a.
2		11.766	12.065	21.142	1.54	1.12	n.a.
**Total:**			**784.434**	**1885.746**	**100.00**	**100.00**	

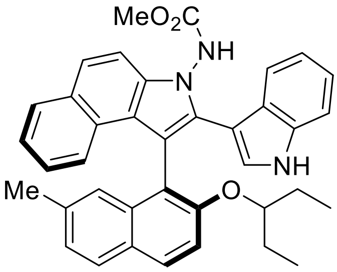

(*S*)-Methyl-(2-(1H-indol-3-yl)-1-(7-methyl-2-(pentan-3-yloxy)naphthalen-1-yl)-3H-benzo[e]indol-3-yl)carbamate (15t)

Brown solid (109 mg, 94% yield), mp 138.0-139.0 ℃; $[\alpha]_D^{24}$ -59.8 (*c* 1.0, $CHCl_3$, 86% ee); IR (KBr): 3411, 2974, 1730, 1380, 1157, 950, 754 cm^{-1}; ^1H NMR (400 MHz, $CDCl_3$) δ 7.82 (d, *J* = 7.9 Hz, 1H), 7.63 (m, 5H), 7.42 (s, 3H), 7.29 (d, *J* = 8.2 Hz, 1H), 7.24 – 7.11 (m, 3H), 7.02 – 6.82 (m, 5H), 3.96 (s, 1H), 3.55 (s, 3H), 2.08 (s, 3H), 1.22 (d, *J* = 30.6 Hz, 4H), 0.46 (s, 3H), 0.30 (s, 3H); ^{13}C NMR (100 MHz, $CDCl_3$) δ 156.0, 154.8, 135.8, 135.5, 134.0, 131.9, 130.2, 128.7, 128.5, 128.3, 127.4, 127.1, 125.9, 125.4, 124.8, 123.5, 123.3, 123.0, 121.8, 121.1, 120.8, 120.0, 119.1, 117.1, 111.2, 111.1, 110.3, 105.6, 81.5, 53.0, 25.7, 21.7, 9.2, 8.6; HRMS (ESI) calcd for $C_{38}H_{35}N_3O_3Na$ *m/z* $[M + Na]^+$: 604.2571; found: 604.2570; HPLC (Daicel Chiralpak IA, *i*-PrOH/hexane = 5/95, flow rate 0.8 mL/min, *λ* = 260 nm): t_1 (major) = 46.4 min, t_2 (minor) = 53.4 min.

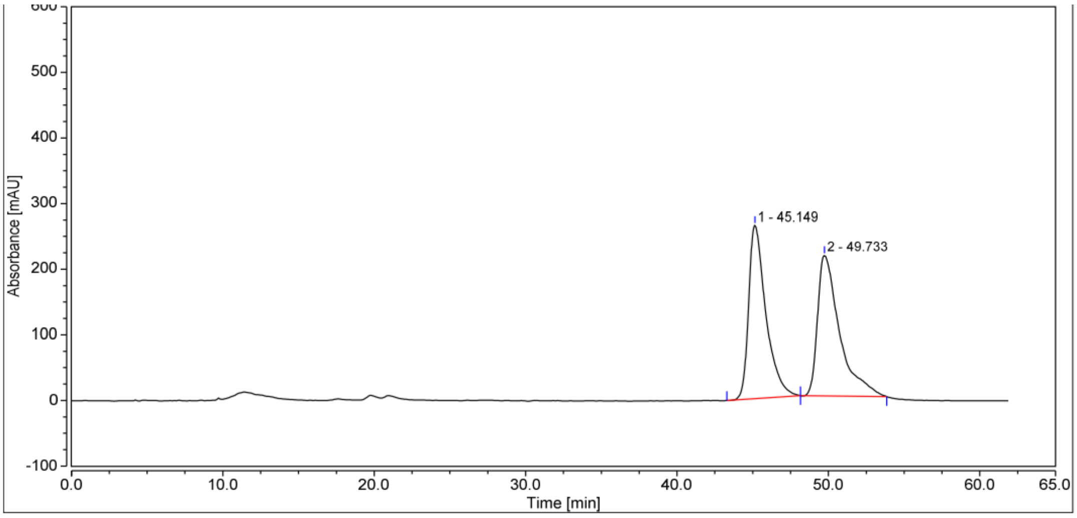

Integration Results							
No.	Peak Name	Retention Time min	Area mAU*min	Height mAU	Relative Area %	Relative Height %	Amount n.a.
1		45.149	348.613	264.163	49.15	55.24	n.a.
2		49.733	360.674	214.064	50.85	44.76	n.a.
**Total:**			**709.286**	**478.227**	**100.00**	**100.00**	

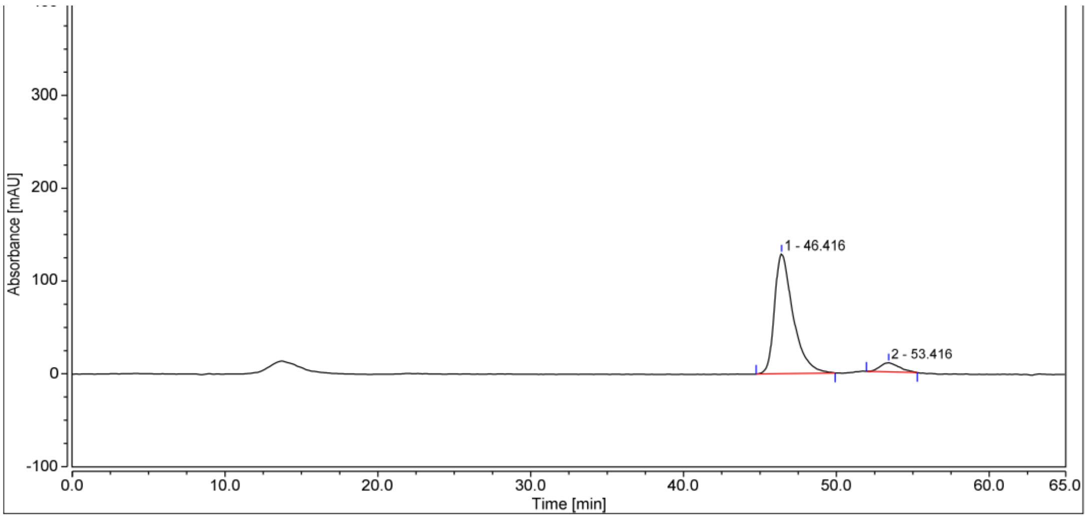

Integration Results							
No.	Peak Name	Retention Time min	Area mAU*min	Height mAU	Relative Area %	Relative Height %	Amount n.a.
1		46.416	182.754	129.036	92.86	93.01	n.a.
2		53.416	14.046	9.702	7.14	6.99	n.a.
**Total:**			**196.800**	**138.738**	**100.00**	**100.00**	

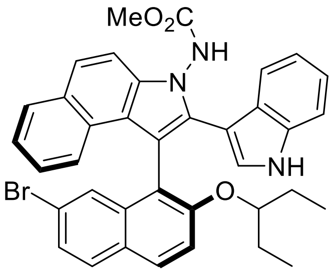

(*S*)-Methyl-(1-(7-bromo-2-(pentan-3-yloxy)naphthalen-1-yl)-2-(1H-indol-3-yl)-3H -benzo[e]indol-3-yl)carbamate (15u)

Brown solid (97 mg, 75% yield), mp 141.0-142.0 ℃; $[\alpha]_D^{22}$ -131.4 (*c* 1.0, $CHCl_3$, 99% ee); IR (KBr): 3411, 2966, 1458, 1244, 746 cm^{-1}; ^1H NMR (600 MHz, $CDCl_3$, 60 °C) δ 7.93 (s, 1H), 7.84 (t, *J* = 12.8 Hz, 2H), 7.72 (dd, *J* = 18.1, 8.9 Hz, 2H), 7.64 (d, *J* = 8.8 Hz, 1H), 7.57 (d, *J* = 8.5 Hz, 1H), 7.49 (d, *J* = 7.8 Hz, 1H), 7.29 – 7.21 (m, 5H), 7.19 (d, *J* = 8.1 Hz, 1H), 7.09 (t, *J* = 7.6 Hz, 1H), 7.02 (t, *J* = 7.0 Hz, 3H), 4.06 (s, 1H), 3.64 (s, 3H), 1.38 – 1.27 (m, 2H), 1.22 (ddd, *J* = 20.7, 13.3, 6.7 Hz, 2H), 0.55 (s, 3H), 0.36 (t, *J* = 7.4 Hz, 3H); ^{13}C NMR (100 MHz, $CDCl_3$) δ 156.6, 155.6, 136.5, 135.5, 134.0, 131.8, 130.2, 129.3, 128.8, 128.5, 128.4, 127.8, 127.2, 126.7, 125.5, 124.5, 123.8, 123.0, 122.0, 120.8, 120.2, 119.2, 117.0, 111.2, 110.3, 105.5, 81.2, 53.1, 25.7, 9.2, 8.6; HRMS (ESI) calcd for $C_{37}H_{32}BrN_3O_3Na$ *m/z* $[M + Na]^+$: 668.1519; found: 668.1514; HPLC (Daicel Chiralpak IA, *i*-PrOH/hexane = 20/80, flow rate 0.8 mL/min, λ = 230 nm): t_1 (major) = 7.6 min, t_2 (minor) = 8.7 min.

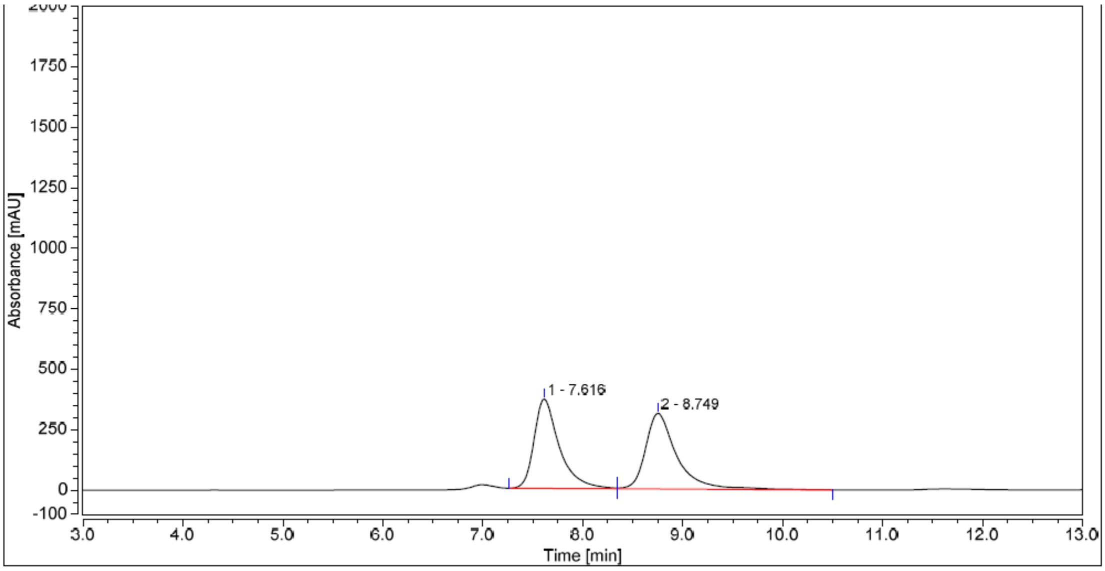

Integration Results

No.	Peak Name	Retention Time min	Area mAU*min	Height mAU	Relative Area %	Relative Height %	Amount n.a.
1		7.616	108.500	369.566	48.90	54.13	n.a.
2		8.749	113.378	313.119	51.10	45.87	n.a.
**Total:**			**221.878**	**682.685**	**100.00**	**100.00**	

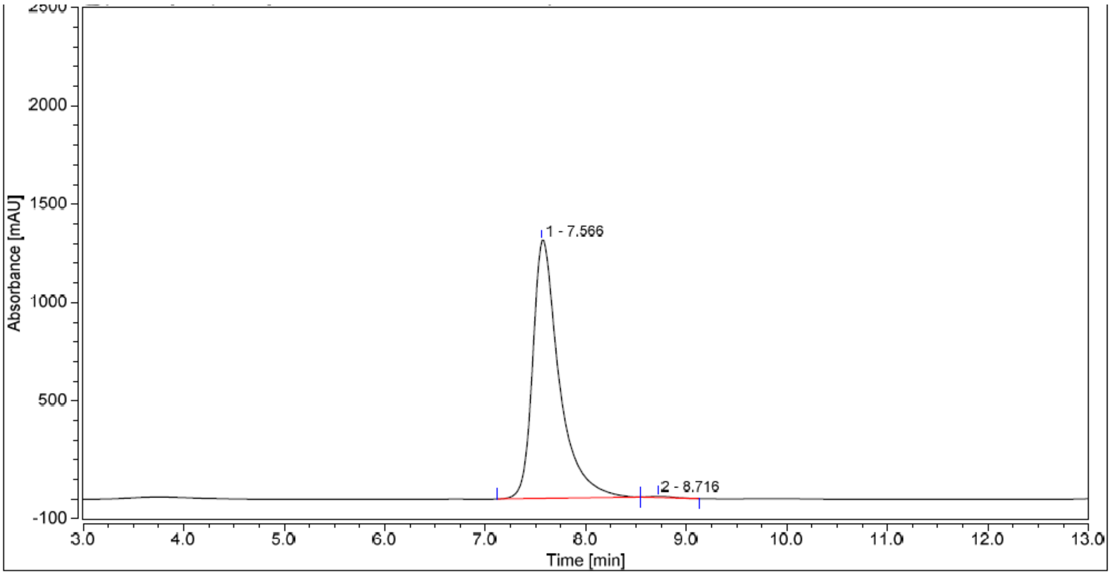

Integration Results

No.	Peak Name	Retention Time min	Area mAU*min	Height mAU	Relative Area %	Relative Height %	Amount n.a.
1		7.566	401.093	1312.334	99.62	99.54	n.a.
2		8.716	1.536	6.059	0.38	0.46	n.a.
**Total:**			**402.628**	**1318.393**	**100.00**	**100.00**	

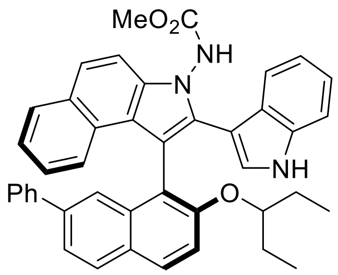

(*S*)-Methyl-(2-(1H-indol-3-yl)-1-(2-(pentan-3-yloxy)-7-phenylnaphthalen-1-yl)-3H-benzo[e]indol-3-yl)carbamate (15v)

Brown solid (125 mg, 97% yield), mp 143.0-144.0 ℃; $[\alpha]_D^{23}$ -94.8 (*c* 1.0, $CHCl_3$, 96% ee); IR (KBr): 3419, 2964, 1456, 1249, 743 cm^{-1}; ^1H NMR (600 MHz, $CDCl_3$) δ 7.95 (s, 1H), 7.79 (d, *J* = 7.7 Hz, 1H), 7.73 (d, *J* = 8.9 Hz, 3H), 7.61 (d, *J* = 8.2 Hz, 1H), 7.54 (d, *J* = 8.7 Hz, 1H), 7.48 (s, 3H), 7.34 (d, *J* = 8.1 Hz, 1H), 7.16 (m, 7H), 6.99 (t, *J* = 7.3 Hz, 1H), 6.94 (s, 1H), 6.89 (s, 1H), 6.74 (m, 2H), 4.00 (s, 1H), 3.46 (s, 3H), 1.22 (d, *J* = 54.8 Hz, 4H), 0.46 (s, 3H), 0.31 (s, 3H); ^{13}C NMR (100 MHz, $CDCl_3$) δ 156.1, 155.2, 141.3, 138.7, 135.9, 135.8, 135.5, 134.2, 131.9, 130.2, 128.6, 128.5, 128.4, 128.3, 128.2, 127.3, 127.0, 126.9, 125.4, 123.8, 123.6, 123.3, 123.0, 122.0, 120.8, 120.0, 119.1, 117.9, 111.3, 110.8, 110.3, 105.7, 81.3, 53.0, 25.7, 9.2, 8.7; HRMS (ESI) calcd for $C_{43}H_{37}N_3O_3Na$ *m/z* $[M + Na]^+$: 666.2727; found: 666.2725; HPLC (Daicel Chiralpak IA, *i*-PrOH/hexane = 10/80, flow rate 0.8 mL/min, *λ* = 230 nm): t_1 (major) = 17.6 min, t_2 (minor) = 20.9 min.

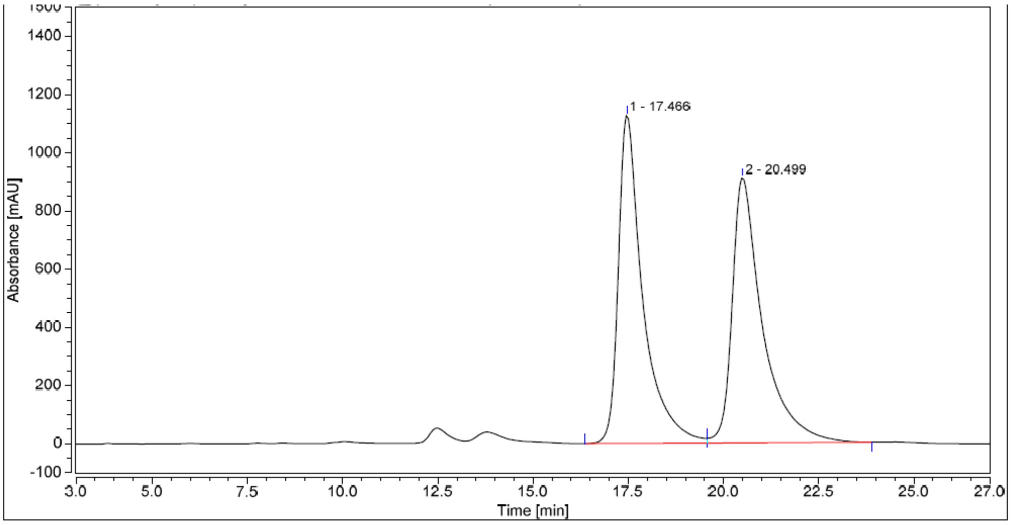

Integration Results							
No.	Peak Name	Retention Time min	Area mAU*min	Height mAU	Relative Area %	Relative Height %	Amount n.a.
1		17.466	841.127	1128.132	50.11	55.32	n.a.
2		20.499	837.356	910.998	49.89	44.68	n.a.
**Total:**			**1678.483**	**2039.130**	**100.00**	**100.00**	

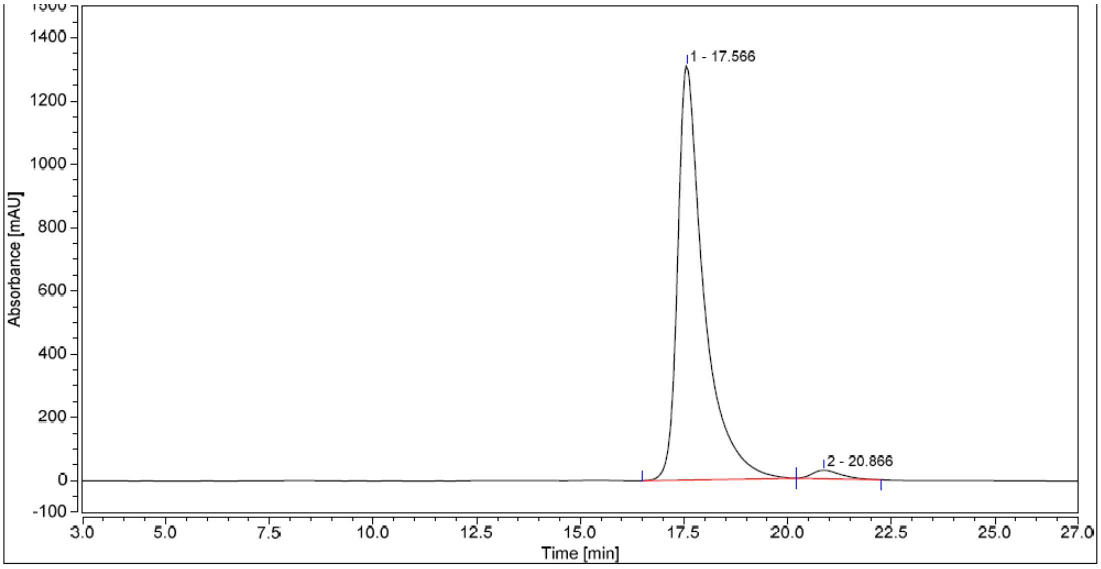

Integration Results							
No.	Peak Name	Retention Time min	Area mAU*min	Height mAU	Relative Area %	Relative Height %	Amount n.a.
1		17.566	962.171	1310.425	97.83	98.01	n.a.
2		20.866	21.319	26.663	2.17	1.99	n.a.
Total:			983.490	1337.088	100.00	100.00	

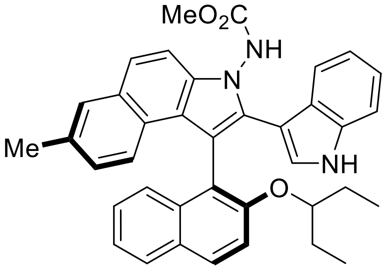

(*S*)-Methyl-(2-(1H-indol-3-yl)-7-methyl-1-(2-(pentan-3-yloxy)naphthalen-1-yl)-3 H-benzo[e]indol-3-yl)carbamate (15w)

Brown solid (110 mg, 95% yield), mp 140.0-141.0 ℃; $[\alpha]_D^{23}$ -11.1 (*c* 1.0, $CHCl_3$, 99% ee); IR (KBr): 3419, 2925, 1267, 756 cm^{-1}; ^1H NMR (600 MHz, $CDCl_3$, 60 °C) δ 7.89 (s, 1H), 7.76 (dd, *J* = 18.6, 8.5 Hz, 2H), 7.68 – 7.53 (m, 4H), 7.47 (d, *J* = 6.3 Hz, 1H), 7.28 (s, 1H), 7.24 (s, 1H), 7.20 (dd, *J* = 18.1, 8.5 Hz, 3H), 7.15 (s, 1H), 7.10 – 7.05 (m, 2H), 7.00 (t, *J* = 7.4 Hz, 1H), 6.81 (d, *J* = 8.5 Hz, 1H), 4.00 (s, 1H), 3.62 (s, 3H), 2.36 (s, 3H), 1.32 (s, 2H), 1.28 – 1.09 (m, 2H), 0.54 (s, 3H), 0.35 (t, *J* = 7.4 Hz, 3H); ^{13}C NMR (150 MHz, $CDCl_3$) δ 156.0, 154.8, 135.5, 133.6, 132.2, 131.9, 130.4, 129.2, 128.7, 127.6, 127.4, 127.0, 126.4, 126.3, 125.9, 125.6, 123.6, 123.0, 123.0, 121.8, 120.8, 120.0, 119.1, 111.2, 110.5, 110.3, 105.6, 82.0, 53.0, 25.7, 21.3, 9.2, 8.7; HRMS (ESI) calcd for $C_{38}H_{35}N_3O_3Na$ *m/z* $[M + Na]^+$: 604.2571; found: 604.2575;

HPLC (Daicel Chiralpak IA, *i*-PrOH/hexane = 7/93, flow rate 0.8 mL/min, λ = 260 nm): t_1 (major) = 27.3 min, t_2 (minor) = 32.5 min.

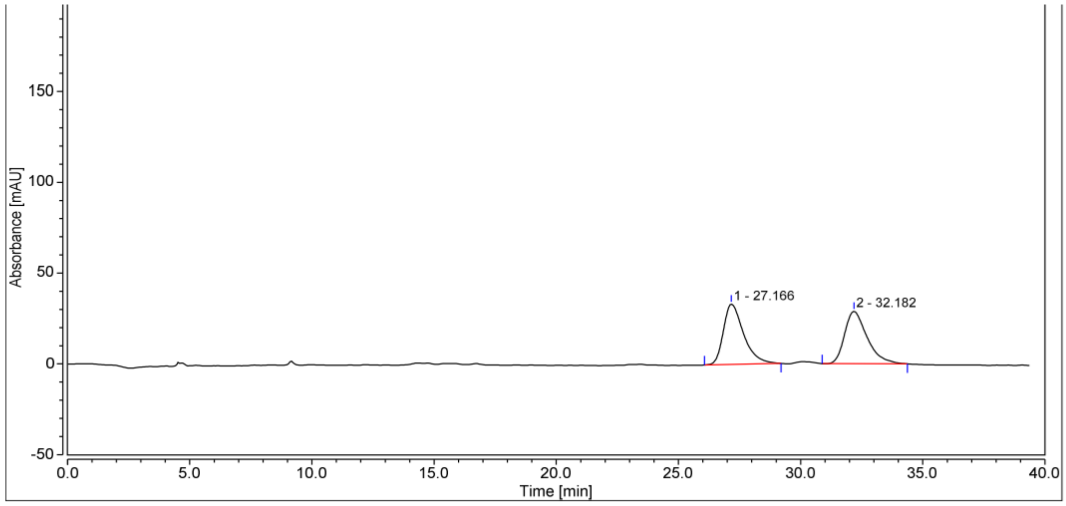

Integration Results

No.	Peak Name	Retention Time min	Area mAU*min	Height mAU	Relative Area %	Relative Height %	Amount n.a.
1		27.166	31.957	33.251	50.93	53.50	n.a.
2		32.182	30.791	28.901	49.07	46.50	n.a.
Total:			62.748	62.152	100.00	100.00	

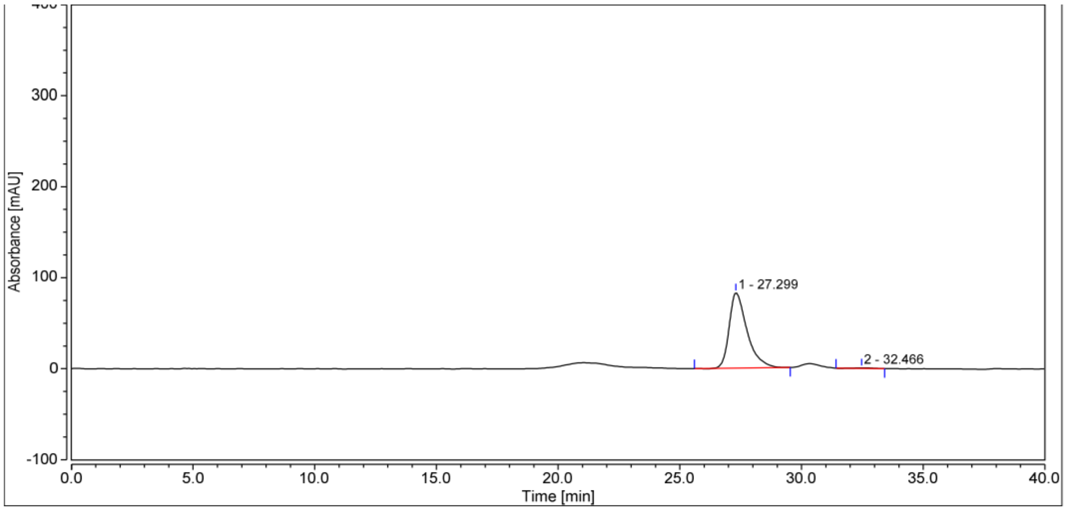

Integration Results

No.	Peak Name	Retention Time min	Area mAU*min	Height mAU	Relative Area %	Relative Height %	Amount n.a.
1		27.299	70.277	82.828	99.44	99.32	n.a.
2		32.466	0.395	0.563	0.56	0.68	n.a.
Total:			70.672	83.391	100.00	100.00	

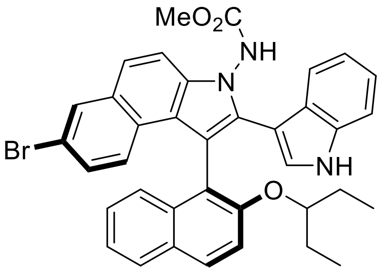

(*S*)-Methyl-(7-bromo-2-(1H-indol-3-yl)-1-(2-(pentan-3-yloxy)naphthalen-1-yl)-3H

-benzo[e]indol-3-yl)carbamate (15x)

Brown solid (120 mg, 93% yield), mp 138.0-139.0 ℃; $[\alpha]_D^{23}$ -25.6 (*c* 1.0, $CHCl_3$, 99% ee); IR (KBr): 3417, 2964, 1734, 1458, 1246, 742 cm^{-1}; ^1H NMR (600 MHz, $CDCl_3$, 60 ℃) δ 7.97 (s, 1H), 7.85 (s, 1H), 7.77 (d, *J* = 9.0 Hz, 1H), 7.73 (d, *J* = 8.1 Hz, 1H), 7.63 (d, *J* = 8.8 Hz, 1H), 7.57 (t, *J* = 11.8 Hz, 2H), 7.45 (d, *J* = 7.1 Hz, 1H), 7.33 (s, 1H), 7.22 (dd, *J* = 9.1, 7.0 Hz, 2H), 7.15 (s, 1H), 7.12 – 7.08 (m, 1H), 7.05 (dd, *J* = 15.3, 8.8 Hz, 3H), 6.99 (t, *J* = 7.4 Hz, 1H), 6.91 (s, 1H), 4.00 (s, 1H), 3.59 (s, 3H), 1.29 (d, *J* = 26.9 Hz, 2H), 1.30 – 1.11 (m, 2H), 0.53 (s, 3H), 0.35 (t, *J* = 7.4 Hz, 3H); ^{13}C NMR (100 MHz, $CDCl_3$) δ 155.9, 154.5, 135.5, 134.1, 132.6, 131.6, 130.3, 130.0, 129.0, 128.5, 128.1, 127.9, 127.7, 127.1, 126.9, 126.5, 125.6, 125.6, 124.9, 123.6, 122.5, 122.1, 120.6, 120.3, 119.1, 117.8, 116.6, 111.4, 111.3, 110.8, 105.5, 81.6, 53.1, 25.7, 9.3, 8.7; HRMS (ESI) calcd for $C_{37}H_{32}BrN_3O_3Na$ *m/z* $[M + Na]^+$: 668.1519; found: 668.1518; HPLC (Daicel Chiralpak IA, *i*-PrOH/hexane = 10/90, flow rate 0.8 mL/min, *λ* = 230 nm): t_1 (major) = 18.5 min, t_2 (minor) = 20.8 min.

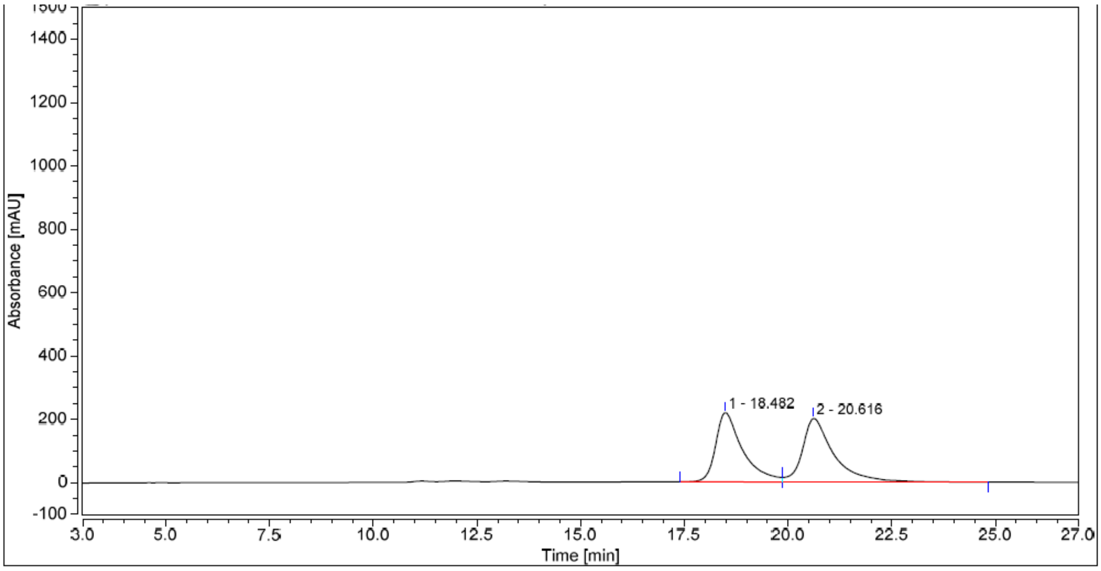

Integration Results							
No.	Peak Name	Retention Time min	Area mAU*min	Height mAU	Relative Area %	Relative Height %	Amount n.a.
1		18.482	164.207	218.659	48.36	52.14	n.a.
2		20.616	175.330	200.745	51.64	47.86	n.a.
**Total:**			**339.538**	**419.404**	**100.00**	**100.00**	

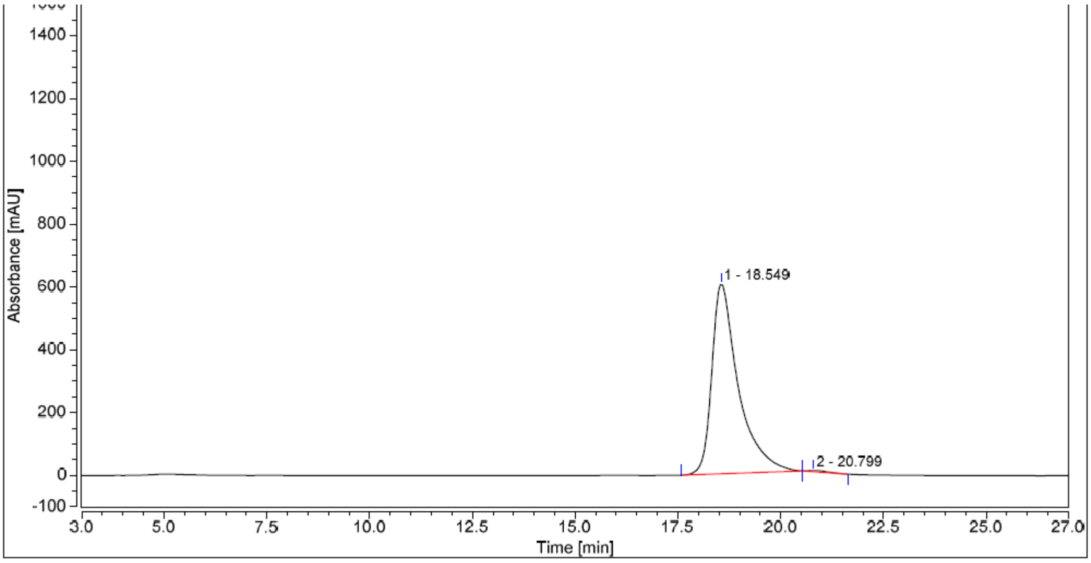

Integration Results							
No.	Peak Name	Retention Time min	Area mAU*min	Height mAU	Relative Area %	Relative Height %	Amount n.a.
1		18.549	450.171	604.282	99.55	99.41	n.a.
2		20.799	2.026	3.579	0.45	0.59	n.a.
Total:			452.197	607.861	100.00	100.00	

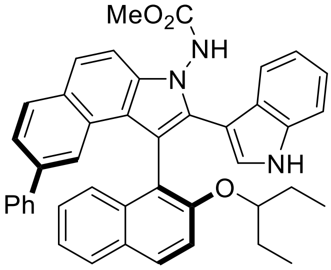

(*S*)-Methyl-(2-(1H-indol-3-yl)-1-(2-(pentan-3-yloxy)naphthalen-1-yl)-8-phenyl-3 H-benzo[e]indol-3-yl)carbamate (15y)

Brown solid (81 mg, 63% yield), mp 134.0-135.0 ℃; $[\alpha]_D^{23}$ +55.4 (*c* 1.0, $CHCl_3$, 94% ee); IR (KBr): 3419, 2964, 1733, 1458, 1244, 746 cm^{-1}; ^1H NMR (400 MHz, $CDCl_3$) δ 7.85 (d, *J* = 8.5 Hz, 1H), 7.80 (s, 1H), 7.76 (d, *J* = 9.1 Hz, 2H), 7.64 (d, *J* = 8.8 Hz, 1H), 7.57 (d, *J* = 8.8 Hz, 1H), 7.49 (d, *J* = 8.4 Hz, 2H), 7.44 (s, 2H), 7.24 (s, 1H), 7.19 – 7.03 (m, 6H), 6.94 (d, *J* = 15.0 Hz, 4H), 6.85 (d, *J* = 5.4 Hz, 2H), 3.91 (s, 1H), 3.53 (s, 3H), 1.12 (d, *J* = 5.6 Hz, 4H), 0.42 (s, 3H), 0.24 (s, 3H); ^{13}C NMR (100 MHz, $CDCl_3$) δ 155.9, 154.5, 140.9, 137.0, 135.6, 134.2, 132.3, 129.4, 129.3, 129.1, 128.8, 128.7, 128.6, 128.3, 127.6, 127.2, 126.7, 126.6, 126.53, 125.9, 125.7, 125.2, 124.1, 123.6, 123.1, 121.9, 121.7, 121.0, 120.2, 119.9, 119.3, 119.2, 118.0, 111.3, 110.9, 110.4, 105.9, 82.0, 53.1, 25.6, 9.3, 8.5; HRMS (ESI) calcd for $C_{43}H_{37}N_3O_3Na$ *m/z* [M + Na]$^+$: 666.2727; found: 666.2724; HPLC (Daicel Chiralpak IA, *i*-PrOH/hexane =

10/90, flow rate 0.8 mL/min, λ = 230 nm): t_1 (minor) = 21.8 min, t_2 (major) = 24.2 min.

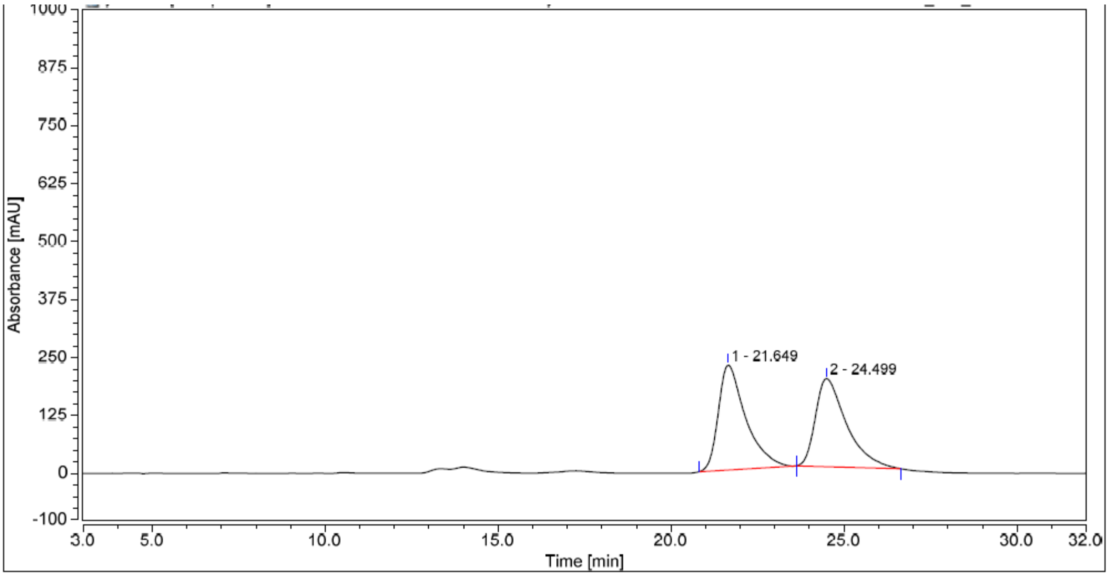

Integration Results							
No.	Peak Name	Retention Time min	Area mAU*min	Height mAU	Relative Area %	Relative Height %	Amount n.a.
1		21.649	207.470	226.594	51.14	54.36	n.a.
2		24.499	198.237	190.248	48.86	45.64	n.a.
**Total:**			**405.707**	**416.842**	**100.00**	**100.00**	

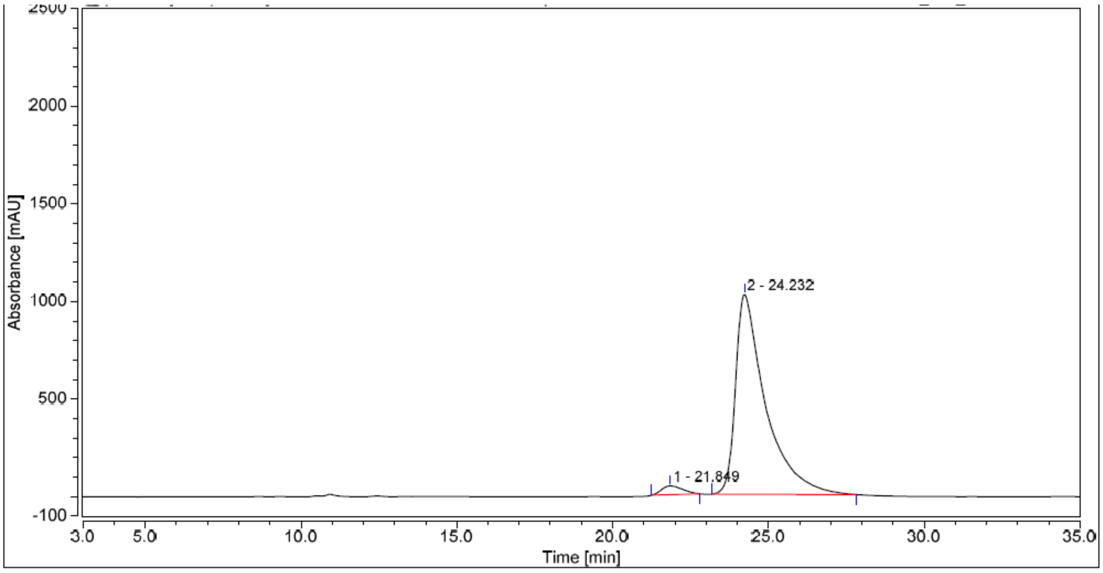

Integration Results							
No.	Peak Name	Retention Time min	Area mAU*min	Height mAU	Relative Area %	Relative Height %	Amount n.a.
1		21.849	35.263	45.444	3.00	4.25	n.a.
2		24.232	1142.039	1023.802	97.00	95.75	n.a.
**Total:**			**1177.302**	**1069.246**	**100.00**	**100.00**	

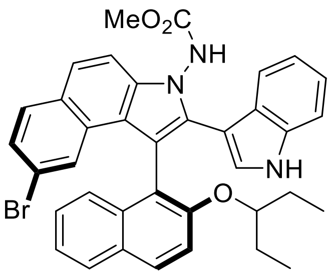

(*S*)-Methyl-(8-bromo-2-(1H-indol-3-yl)-1-(2-(pentan-3-yloxy)naphthalen-1-yl)-3H-benzo[e]indol-3-yl)carbamate (15z)

Brown solid (120 mg, 93% yield), mp 135.0-136.0 ℃; $[\alpha]_D^{23}$ -84.2 (*c* 1.0, $CHCl_3$, 92% ee); IR (KBr): 3419, 2968, 1730, 1458, 1247, 815, 746 cm^{-1}; ^1H NMR (600 MHz, $CDCl_3$, 60 °C) δ 7.90 (s, 1H), 7.82 (d, *J* = 9.0 Hz, 1H), 7.75 (d, *J* = 8.1 Hz, 1H), 7.68 (d, *J* = 8.6 Hz, 1H), 7.67 – 7.53 (m, 3H), 7.49 (d, *J* = 7.7 Hz, 1H), 7.36 (d, *J* = 1.6 Hz, 1H), 7.29 (dd, *J* = 8.6, 1.9 Hz, 2H), 7.25 (d, *J* = 9.0 Hz, 1H), 7.21 (d, *J* = 7.1 Hz, 1H), 7.17 (t, *J* = 8.3 Hz, 2H), 7.08 (t, *J* = 7.6 Hz, 1H), 7.02 (t, *J* = 7.5 Hz, 2H), 4.08 (s, 1H), 3.63 (s, 3H), 1.41 – 1.30 (m, 2H), 1.23 (dd, *J* = 13.8, 7.2 Hz, 2H), 0.57 (s, 3H), 0.41 (t, *J* = 7.4 Hz, 3H); ^{13}C NMR (100 MHz, $CDCl_3$) δ 156.0, 154.5, 135.5, 135.3, 134.4, 132.4, 129.8, 129.6, 129.2, 129.0, 128.5, 127.7, 126.9, 126.3, 126.1, 125.8, 125.5, 125.4, 123.5, 123.1, 121.9, 120.0, 119.7, 119.4, 119.0, 116.7, 111.2, 110.9, 110.7, 105.3, 81.1, 53.0, 25.7, 9.2, 8.7; HRMS (ESI) calcd for $C_{37}H_{32}BrN_3O_3Na$ *m/z* $[M + Na]^+$: 668.1519; found: 668.1516; HPLC (Daicel Chiralpak IB, *i*-PrOH/hexane = 7/93, flow rate 0.8 mL/min, *λ* = 260 nm): t_1 (minor) = 27.3 min, t_2 (major) = 30.4 min.

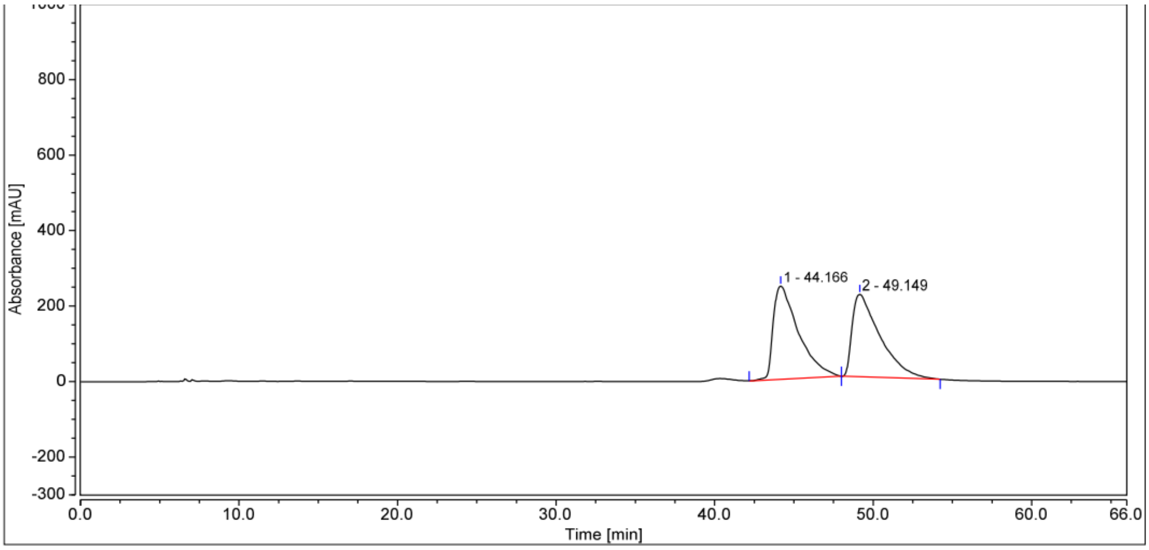

Integration Results							
No.	Peak Name	Retention Time min	Area mAU*min	Height mAU	Relative Area %	Relative Height %	Amount n.a.
1		44.166	457.013	246.979	50.67	53.10	n.a.
2		49.149	444.858	218.119	49.33	46.90	n.a.
**Total:**			**901.871**	**465.098**	**100.00**	**100.00**	

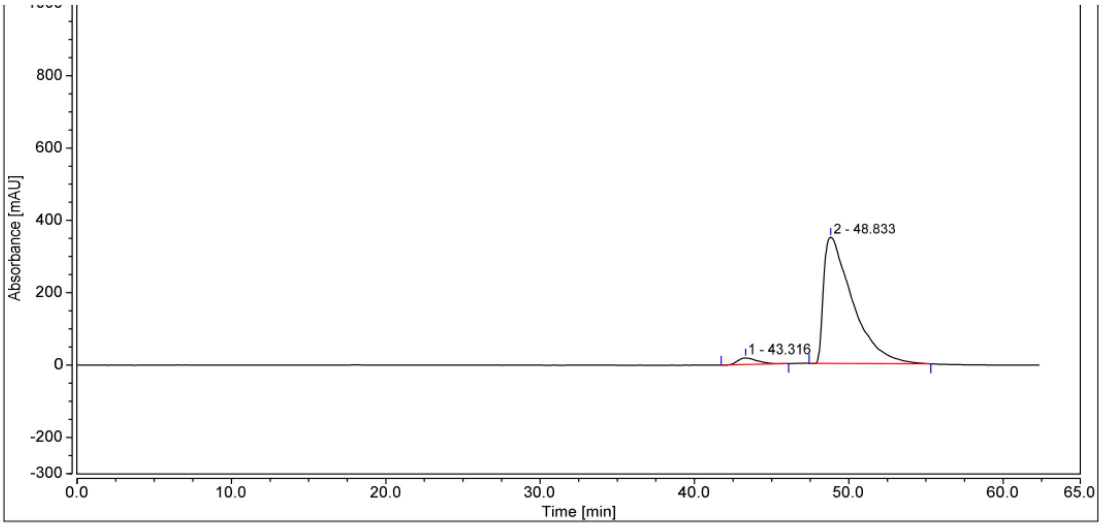

Integration Results							
No.	Peak Name	Retention Time min	Area mAU*min	Height mAU	Relative Area %	Relative Height %	Amount n.a.
1		43.316	26.727	17.899	3.40	4.88	n.a.
2		48.833	758.482	349.033	96.60	95.12	n.a.
**Total:**			**785.209**	**366.932**	**100.00**	**100.00**	

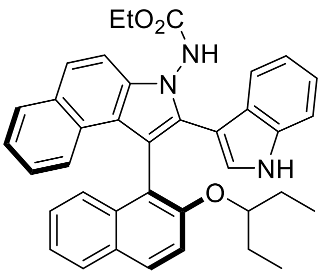

(*S*)-Ethyl-(2-(1H-indol-3-yl)-1-(2-(pentan-3-yloxy)naphthalen-1-yl)-3H-benzo[e]indol-3-yl)carbamate (15aa)

Brown solid (96 mg, 83% yield), mp 124.0-125.0 ℃; $[\alpha]_D^{23}$ -54.6 (*c* 1.0, $CHCl_3$, 97% ee); IR (KBr): 3419, 2966, 1722, 1458, 1240, 744 cm^{-1}; ^1H NMR (400 MHz, $CDCl_3$) δ 7.81 (d, *J* = 8.0 Hz, 1H), 7.74 (d, *J* = 8.9 Hz, 3H), 7.61 (dd, *J* = 21.5, 8.8 Hz, 3H), 7.44 (s, 2H), 7.24 – 7.19 (m, 4H), 7.17 (s, 1H), 7.06 (s, 1H), 6.95 (dd, *J* = 16.0, 7.7 Hz, 3H), 6.84 (s, 1H), 4.01 (s, 3H), 1.23 (d, *J* = 28.4 Hz, 4H), 1.02 (t, *J* = 6.9 Hz, 3H), 0.46 (s, 3H), 0.31 (t, *J* = 7.2 Hz, 3H); ^{13}C NMR (100 MHz, $CDCl_3$) δ 155.6, 154.5, 135.5, 133.9, 132.1, 130.2, 129.2, 128.8, 128.5, 128.3, 127.6, 127.0, 126.3, 125.9, 125.6, 125.4, 123.6, 123.5, 123.2, 122.9, 121.9, 121.8, 120.7, 120.0, 119.1, 118.4, 111.1, 110.7, 110.4, 105.5, 81.8, 62.2, 25.7, 14.2, 9.2, 8.7; HRMS (ESI) calcd for $C_{38}H_{35}N_3O_3Na$ *m/z* $[M + Na]^+$: 604.2571; found: 604.2570; HPLC (Daicel Chiralpak IA, *i*-PrOH/hexane = 10/90, flow rate 0.8 mL/min, λ = 230 nm): t_1 (major) = 16.4 min, t_2 (minor) = 21.6 min.

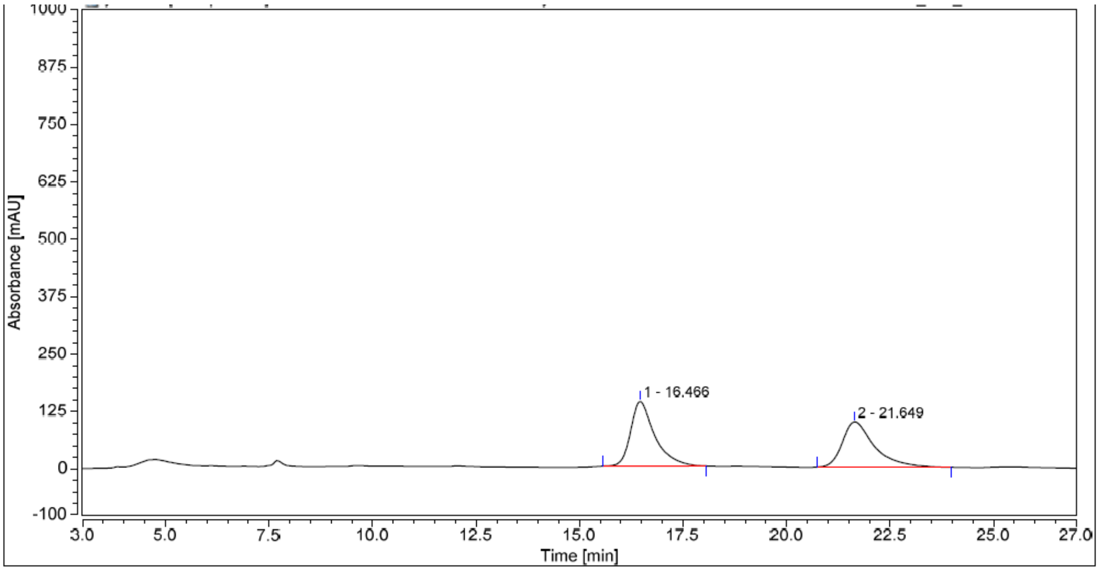

Integration Results

No.	Peak Name	Retention Time min	Area mAU*min	Height mAU	Relative Area %	Relative Height %	Amount n.a.
1		16.466	95.203	140.850	51.80	58.83	n.a.
2		21.649	88.588	98.560	48.20	41.17	n.a.
**Total:**			**183.791**	**239.410**	**100.00**	**100.00**	

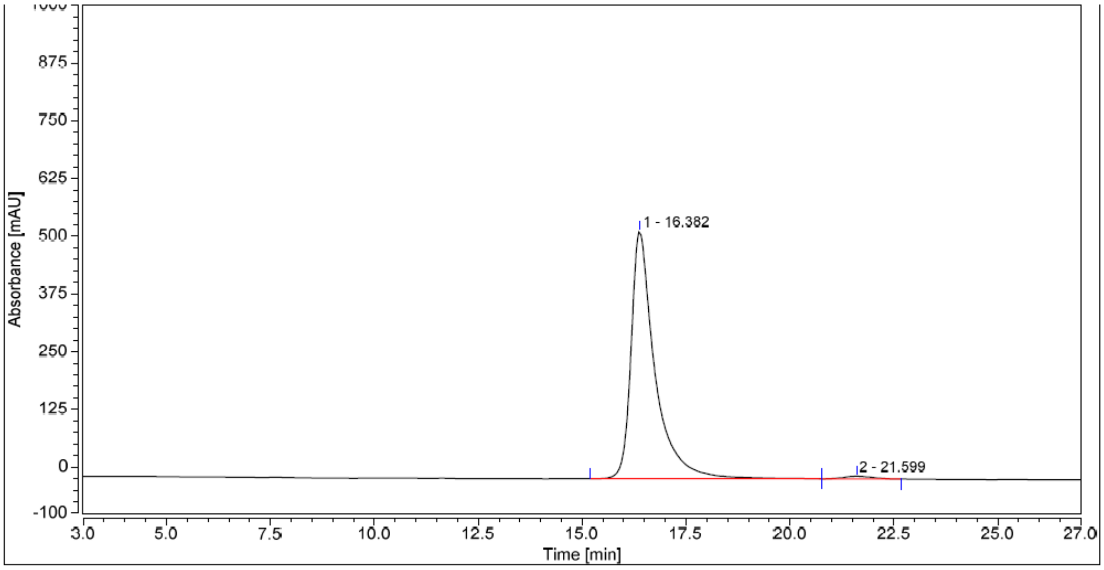

Integration Results

No.	Peak Name	Retention Time min	Area mAU*min	Height mAU	Relative Area %	Relative Height %	Amount n.a.
1		16.382	355.907	535.691	98.77	98.98	n.a.
2		21.599	4.415	5.537	1.23	1.02	n.a.
**Total:**			**360.322**	**541.228**	**100.00**	**100.00**	

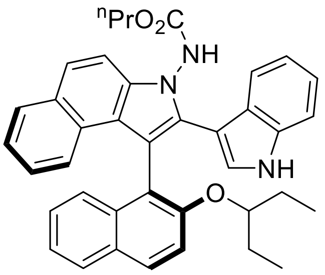

(*S*)-Propyl-(2-(1H-indol-3-yl)-1-(2-(pentan-3-yloxy)naphthalen-1-yl)-3H-benzo[e]indol-3-yl)carbamate (15ab)

Brown solid (89 mg, 75% yield), mp 121.0-122.0 ℃; $[\alpha]_D^{23}$ -54.0 (*c* 1.0, $CHCl_3$, 97% ee); IR (KBr): 3421, 2966, 1730, 1238, 744 cm^{-1}; ^{1}H NMR (400 MHz, $CDCl_3$) δ 7.83 (d, *J* = 8.0 Hz, 1H), 7.77 (d, *J* = 9.0 Hz, 2H), 7.67 (d, *J* = 8.8 Hz, 2H), 7.62 (d, *J* = 8.8 Hz, 1H), 7.41 (s, 2H), 7.28 – 7.16 (m, 5H), 7.11 (s, 1H), 7.04 – 6.85 (m, 5H), 3.96 (s, 3H), 1.45 (d, *J* = 6.7 Hz, 2H), 1.23 (d, *J* = 23.9 Hz, 4H), 0.69 (s, 3H), 0.47 (s, 3H), 0.31 (t, *J* = 7.4 Hz, 3H); ^{13}C NMR (100 MHz, $CDCl_3$) δ 155.8, 154.5, 135.5, 134.1, 132.2, 130.2, 129.1, 128.8, 128.5, 128.3, 127.6, 127.1, 126.3, 125.9, 125.6, 125.4, 123.6, 123.5, 123.2, 122.9, 121.9, 120.7, 120.1, 119.1, 118.1, 111.2, 110.7, 110.4, 105.7, 81.9, 67.7, 25.7, 22.0, 10.0, 9.2, 8.7; HRMS (ESI) calcd for $C_{39}H_{37}N_3O_3Na$ *m/z* $[M + Na]^+$: 618.2727; found: 618.2728; HPLC (Daicel Chiralpak IA, *i*-PrOH/hexane = 10/90, flow rate 0.8 mL/min, *λ* = 260 nm): t_1 (major) = 17.3 min, t_2 (minor) = 25.5 min.

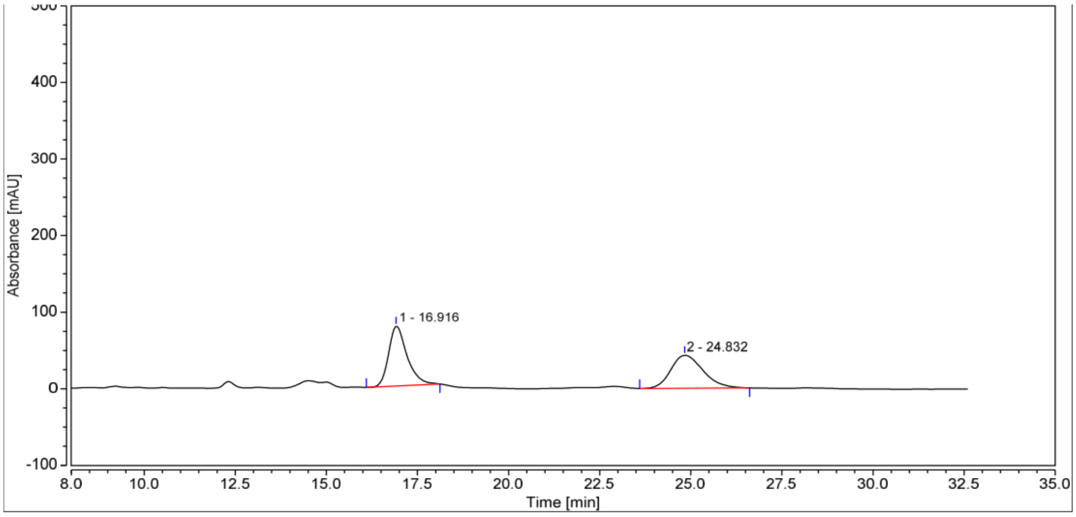

Integration Results							
No.	Peak Name	Retention Time min	Area mAU*min	Height mAU	Relative Area %	Relative Height %	Amount n.a.
1		16.916	45.814	78.147	50.90	64.47	n.a.
2		24.832	44.189	43.071	49.10	35.53	n.a.
**Total:**			**90.002**	**121.217**	**100.00**	**100.00**	

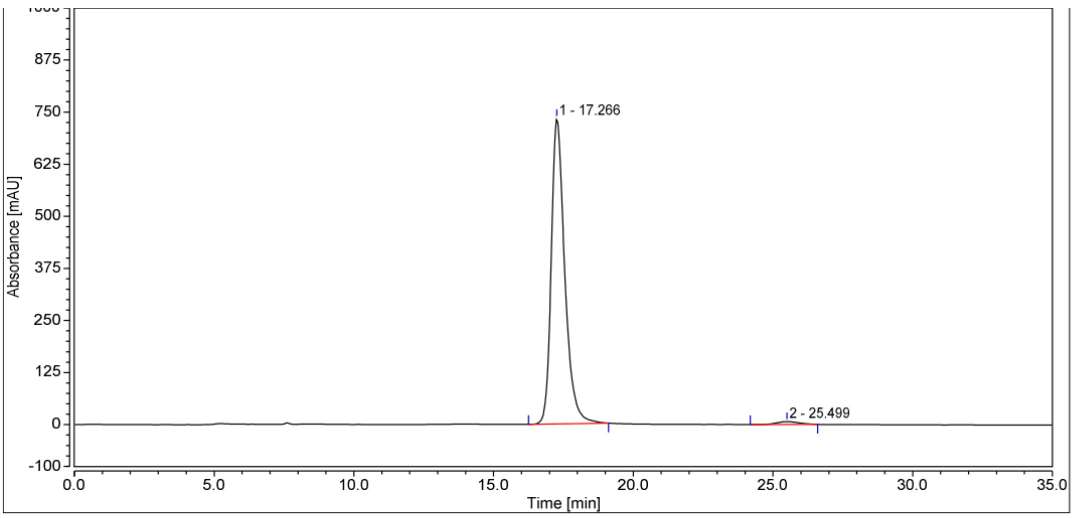

Integration Results							
No.	Peak Name	Retention Time min	Area mAU*min	Height mAU	Relative Area %	Relative Height %	Amount n.a.
1		17.266	410.749	733.317	98.49	99.07	n.a.
2		25.499	6.297	6.858	1.51	0.93	n.a.
Total:			417.046	740.175	100.00	100.00	

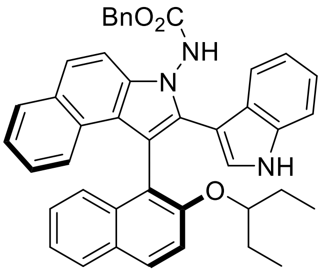

(*S*)-Benzyl-(2-(1H-indol-3-yl)-1-(2-(pentan-3-yloxy)naphthalen-1-yl)-3H-benzo[e]indol-3-yl)carbamate (15ac)

Brown solid (99 mg, 77% yield), mp 126.0-127.0 ℃; $[\alpha]_D^{24}$ -56.2 (*c* 1.0, $CHCl_3$, 96% ee); IR (KBr): 3423, 2927, 1728, 1265, 752 cm^{-1}; ^1H NMR (400 MHz, $CDCl_3$) δ 7.81 (d, *J* = 6.6 Hz, 1H), 7.73 (d, *J* = 9.0 Hz, 2H), 7.63 (d, *J* = 8.5 Hz, 2H), 7.55 (d, *J* = 18.1 Hz, 4H), 7.28 – 7.12 (m, 7H), 7.09 – 6.83 (m, 8H), 4.99 (s, 2H), 3.94 (s, 1H), 1.26 (s, 2H), 1.15 (s, 2H), 0.47 (s, 3H), 0.29 (s, 3H); ^{13}C NMR (100 MHz, $CDCl_3$) δ 155.3, 154.4, 135.4, 134.0, 132.1, 130.2, 129.1, 128.7, 128.5, 128.3, 128.1, 128.1, 127.8, 127.8, 127.6, 127.0, 126.3, 125.9, 125.7, 125.4, 123.5, 123.2, 123.0, 121.8, 120.7, 120.1, 119.1, 111.2, 110.8, 110.4, 105.5, 81.7, 67.5, 25.7, 9.2, 8.7; HRMS (ESI) calcd for $C_{43}H_{37}N_3O_3Na$ *m/z* $[M + Na]^+$: 666.2727; found: 666.2725; HPLC (Daicel Chiralpak IF, *i*-PrOH/hexane = 7/93, flow rate 0.8 mL/min, λ = 260 nm): t_1 (minor) = 23.4 min, t_2 (major) = 24.4 min.

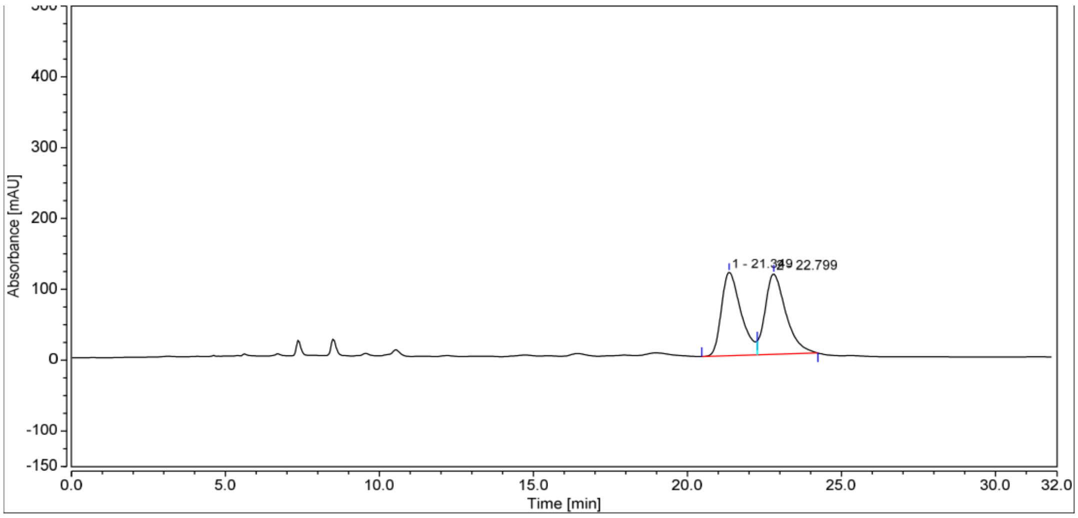

Integration Results							
No.	Peak Name	Retention Time min	Area mAU*min	Height mAU	Relative Area %	Relative Height %	Amount n.a.
1		21.349	87.052	117.668	49.46	50.95	n.a.
2		22.799	88.953	113.296	50.54	49.05	n.a.
**Total:**			**176.004**	**230.964**	**100.00**	**100.00**	

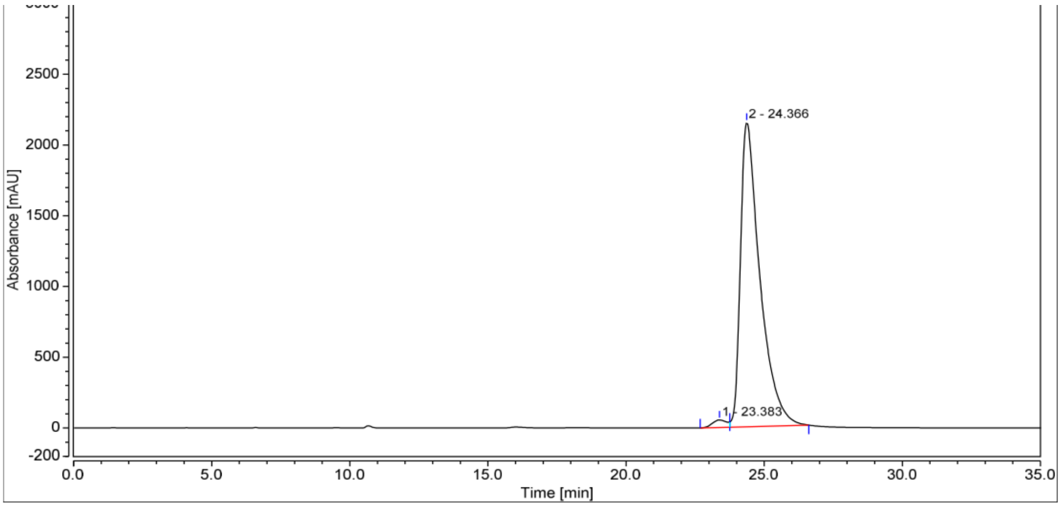

Integration Results							
No.	Peak Name	Retention Time min	Area mAU*min	Height mAU	Relative Area %	Relative Height %	Amount n.a.
1		23.383	31.517	52.649	1.76	2.39	n.a.
2		24.366	1758.721	2151.351	98.24	97.61	n.a.
**Total:**			**1790.238**	**2204.000**	**100.00**	**100.00**	

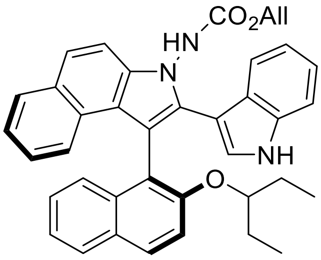

(*S*)-Allyl-(2-(1H-indol-3-yl)-1-(2-(pentan-3-yloxy)naphthalen-1-yl)-3H-benzo[e]indol-3-yl)carbamate (15ad)

Brown solid (53 mg, 52% yield), mp 114.0-115.0 ℃; $[\alpha]_D^{24}$ -36.6 (*c* 1.0, $CHCl_3$, 95% ee); IR (KBr): 3423, 2975, 1662, 1384, 1249, 950, 750 cm^{-1}; ^1H NMR (600 MHz, $CDCl_3$, 60 °C) δ 7.83 (d, *J* = 8.1 Hz, 2H), 7.78 (t, *J* = 7.4 Hz, 1H), 7.74 (d, *J* = 7.9 Hz, 1H), 7.68 (d, *J* = 8.8 Hz, 1H), 7.65 (t, *J* = 9.5 Hz, 2H), 7.48 (d, *J* = 7.1 Hz, 1H), 7.33 (s, 1H), 7.23 – 7.18 (m, 5H), 7.15 (d, *J* = 8.1 Hz, 2H), 7.06 (t, *J* = 7.6 Hz, 1H), 7.02 – 6.93 (m, 2H), 5.83 – 5.59 (m, 1H), 5.07 (dd, *J* = 13.9, 6.5 Hz, 2H), 4.51 (s, 2H), 4.00 (s, 1H), 1.28 (d, *J* = 22.9 Hz, 2H), 1.23 – 1.13 (m, 2H), 0.52 (s, 3H), 0.33 (t, *J* = 7.4 Hz, 3H); ^{13}C NMR (100 MHz, $CDCl_3$) δ 154.6, 154.4, 135.6, 131.9, 131.7, 130.2, 129.6, 129.1, 128.8, 128.5, 128.3, 127.7, 127.6, 127.2, 126.4, 126.31, 125.9, 125.6, 125.4, 123.7, 123.6, 123.2, 122.9, 121.9, 121.6, 120.2, 119.2, 118.0, 111.2, 110.3, 105.8, 81.7, 66.5, 29.6, 25.8, 9.2, 8.7; HRMS (ESI) calcd for $C_{39}H_{35}N_3O_3Na$ *m/z* [M + Na]$^+$: 616.2571; found: 616.2573; HPLC (Daicel Chiralpak IA, *i*-PrOH/hexane = 10/90, flow rate 0.8 mL/min, λ = 230 nm): t_1 (major) = 17.9 min, t_2 (minor) = 21.8 min.

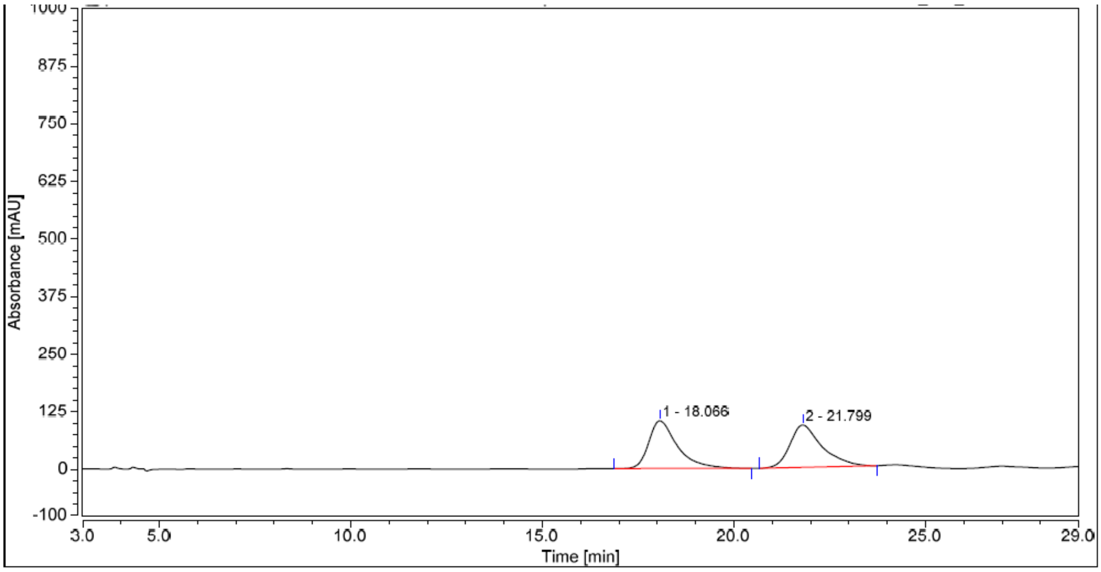

Integration Results							
No.	Peak Name	Retention Time min	Area mAU*min	Height mAU	Relative Area %	Relative Height %	Amount n.a.
1		18.066	89.976	103.634	50.16	52.97	n.a.
2		21.799	89.414	92.002	49.84	47.03	n.a.
**Total:**			**179.390**	**195.637**	**100.00**	**100.00**	

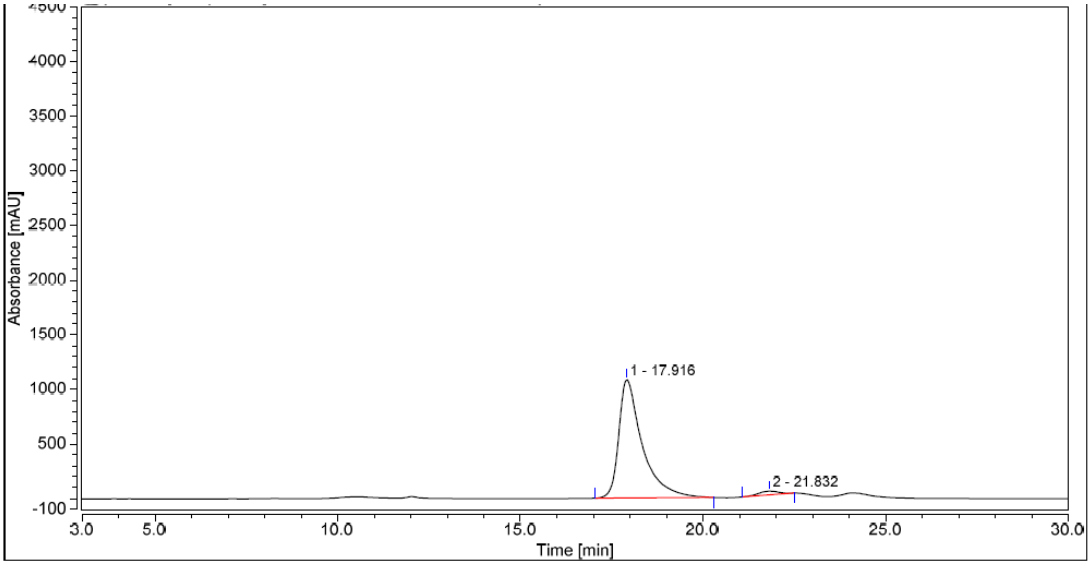

Integration Results							
No.	Peak Name	Retention Time min	Area mAU*min	Height mAU	Relative Area %	Relative Height %	Amount n.a.
1		17.916	807.504	1084.322	97.35	96.97	n.a.
2		21.832	22.011	33.894	2.65	3.03	n.a.
**Total:**			**829.515**	**1118.216**	**100.00**	**100.00**	

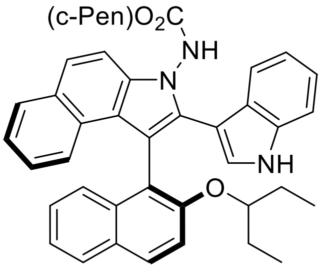

(*S*)-Cyclopentyl-(2-(1H-indol-3-yl)-1-(2-(pentan-3-yloxy)naphthalen-1-yl)-3H-benzo[e]indol-3-yl)carbamate (15ae)

Brown solid (61 mg, 60% yield), mp 128.0-129.0 ℃; $[\alpha]_D^{24}$ -29.6 (*c* 0.5, $CHCl_3$, 94% ee); IR (KBr): 3419, 2975, 1716, 1384, 1263, 754 cm^{-1}; ^1H NMR (600 MHz, $CDCl_3$, 60 °C) δ 7.87 (s, 1H), 7.83 (d, *J* = 8.0 Hz, 1H), 7.77 (dd, *J* = 15.2, 8.5 Hz, 2H), 7.66 (dd, *J* = 25.0, 8.7 Hz, 3H), 7.48 (d, *J* = 7.4 Hz, 1H), 7.23 – 7.19 (m, 6H), 7.17 (d, *J* = 8.0 Hz, 2H), 7.07 (t, *J* = 7.5 Hz, 1H), 7.00 (t, *J* = 7.3 Hz, 1H), 6.96 (t, *J* = 7.6 Hz, 1H), 5.07 (s, 1H), 3.99 (s, 1H), 1.69 (s, 2H), 1.44 (s, 7H), 1.22 (ddd, *J* = 19.6, 14.0, 7.3 Hz, 4H), 0.50 (s, 3H), 0.33 (t, *J* = 7.4 Hz, 3H); ^{13}C NMR (100 MHz, $CDCl_3$) δ 155.4, 154.5, 135.5, 134.0, 132.3, 130.1, 129.1, 128.7, 128.5, 128.3, 127.6, 127.0, 126.2, 125.8, 125.3, 123.5, 123.4, 123.2, 122.9, 121.8, 120.6, 120.1, 119.2, 118.0, 111.1, 110.5, 110.4, 105.7, 81.3, 79.3, 65.8, 32.5, 25.8, 23.3, 15.2, 9.2, 8.8; HRMS (ESI) calcd for $C_{41}H_{39}N_3O_3Na$ *m/z* $[M + Na]^+$: 644.2884; found: 644.2882; HPLC (Daicel

Chiralpak IA, *i*-PrOH/hexane = 10/90, flow rate 0.8 mL/min, λ = 230 nm): t_1 (major) = 16.9 min, t_2 (minor) = 19.3 min.

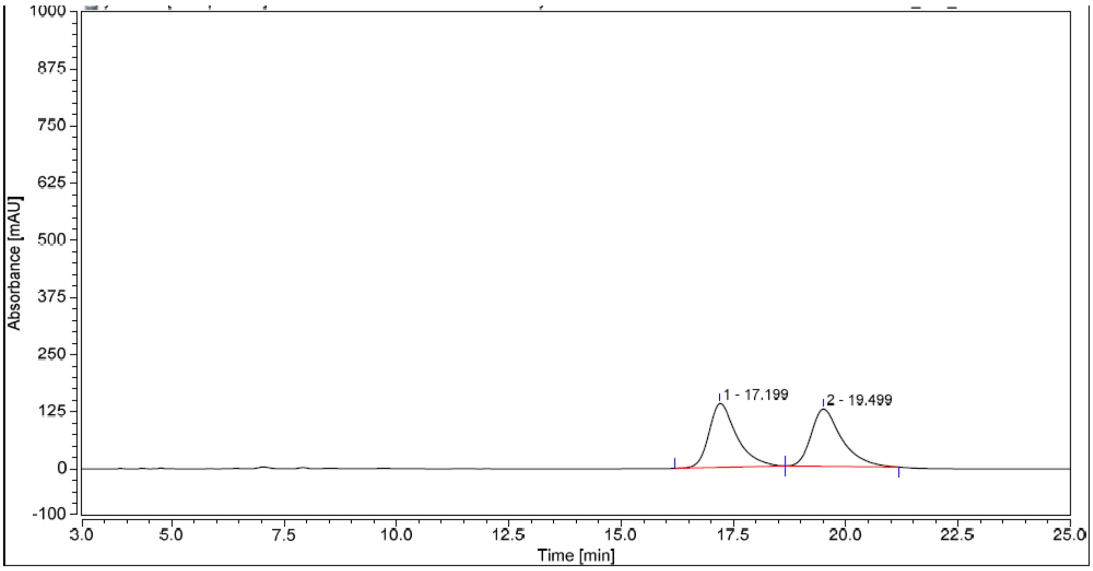

Integration Results							
No.	Peak Name	Retention Time min	Area mAU*min	Height mAU	Relative Area %	Relative Height %	Amount n.a.
1		17.199	100.246	139.683	50.25	52.70	n.a.
2		19.499	99.235	125.394	49.75	47.30	n.a.
**Total:**			**199.481**	**265.078**	**100.00**	**100.00**	

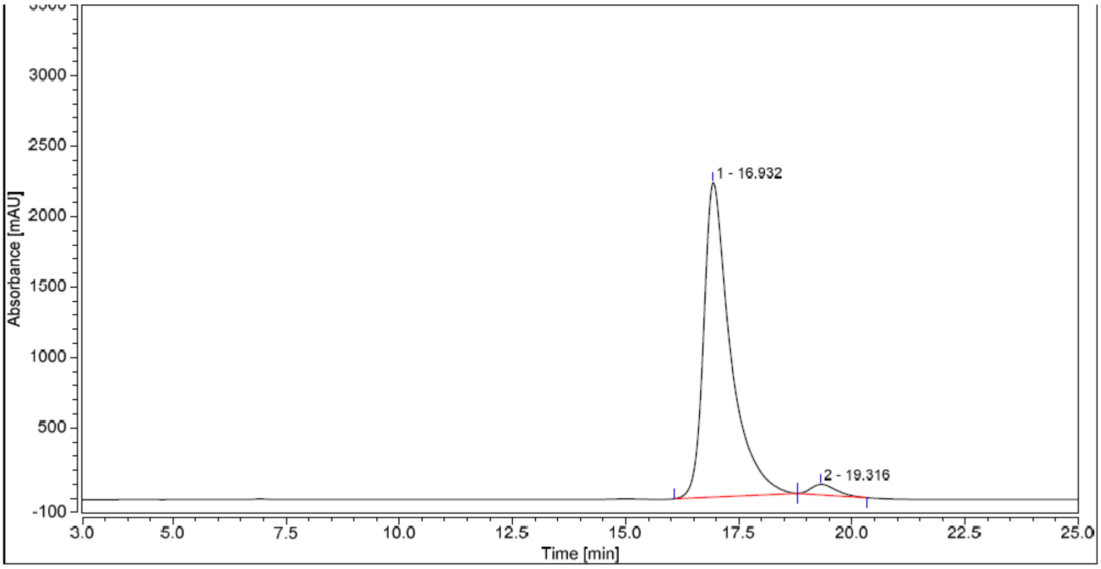

Integration Results							
No.	Peak Name	Retention Time min	Area mAU*min	Height mAU	Relative Area %	Relative Height %	Amount n.a.
1		16.932	1531.869	2232.054	96.98	96.75	n.a.
2		19.316	47.779	74.996	3.02	3.25	n.a.
**Total:**			**1579.648**	**2307.050**	**100.00**	**100.00**	

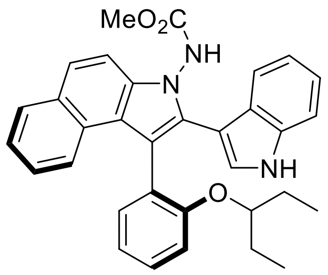

(*S*)-Methyl-(2-(1H-indol-3-yl)-1-(2-(pentan-3-yloxy)phenyl)-3H-benzo[e]indol-3-yl)carbamate (15af)

Brown solid (81 mg, 79% yield), mp 128.0-130.0 ℃; $[\alpha]_D^{24}$ +66.0 (*c* 1.0, $CHCl_3$, 80% ee); IR (KBr): 3425, 2975, 1384, 1153, 958, 811 cm^{-1}; ^1H NMR (400 MHz, $CDCl_3$) δ 8.10 (s, 1H), 7.87 (d, *J* = 8.0 Hz, 1H), 7.68 (dd, *J* = 13.0, 8.6 Hz, 2H), 7.54 (d, *J* = 8.7 Hz, 1H), 7.44 (s, 1H), 7.35 – 7.26 (m, 2H), 7.23 – 7.18 (m, 4H), 7.05 (dt, *J* = 14.3, 6.9 Hz, 3H), 6.89 (s, 1H), 6.80 (t, *J* = 7.2 Hz, 1H), 3.98 (s, 1H), 3.63 (s, 3H), 1.40 – 1.13 (m, 4H), 0.55 (s, 3H), 0.38 (s, 3H); ^{13}C NMR (100 MHz, $CDCl_3$) δ 157.1, 156.0, 135.6, 133.4, 130.7, 130.1, 128.7, 128.4, 128.3, 127.4, 127.1, 125.8, 125.3, 123.7, 123.5, 123.0, 122.0, 120.2, 120.0, 119.5, 114.5, 111.2, 110.2, 105.6, 79.9, 53.0, 25.5, 25.4, 9.3, 8.9; HRMS (ESI) calcd for $C_{33}H_{31}N_3O_3Na$ *m/z* $[M + Na]^+$: 540.2258; found: 540.2255; HPLC (Daicel Chiralpak IC, *i*-PrOH/hexane = 10/90, flow rate 0.8 mL/min, λ = 260 nm): t_1 (major) = 9.3 min, t_2 (minor) = 11.4 min.

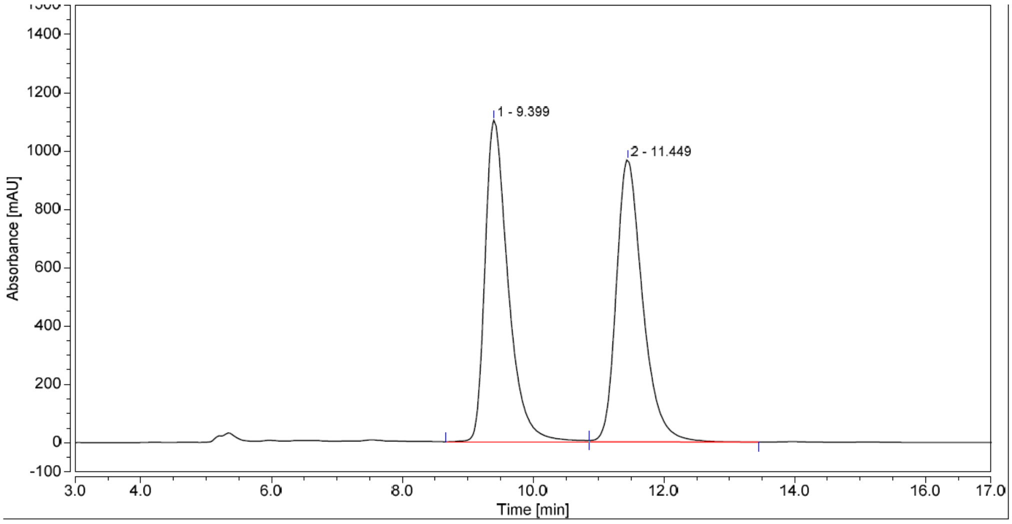

Integration Results							
No.	Peak Name	Retention Time min	Area mAU*min	Height mAU	Relative Area %	Relative Height %	Amount n.a.
1		9.399	452.299	1102.566	50.00	53.27	n.a.
2		11.449	452.287	967.262	50.00	46.73	n.a.
**Total:**			**904.586**	**2069.829**	**100.00**	**100.00**	

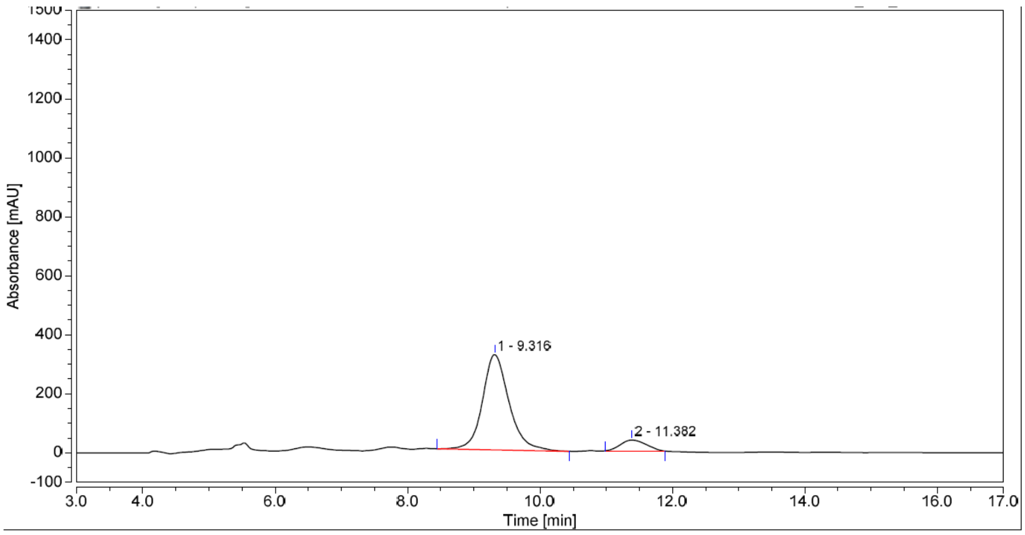

Integration Results							
No.	Peak Name	Retention Time min	Area mAU*min	Height mAU	Relative Area %	Relative Height %	Amount n.a.
1		9.316	149.835	323.366	90.08	89.76	n.a.
2		11.382	16.499	36.897	9.92	10.24	n.a.
**Total:**			**166.335**	**360.263**	**100.00**	**100.00**	

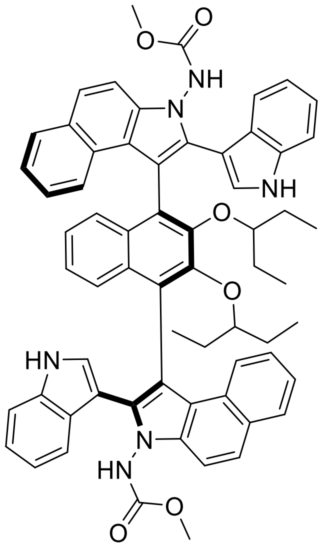

(*S*)-Dimethyl ((2,3-bis(pentan-3-yloxy)naphthalene-1,4-diyl)bis(2-(1H-indol-3-yl) -3H-benzo[e]indole-1,3-diyl))dicarbamate (15ag)

Brown solid (12.2 mg, 25% yield), mp 152.0-153.0 ℃; $[\alpha]_D^{24}$ -86.2 (*c* 1.0, $CHCl_3$, 94% ee); IR (KBr): 3416, 2961, 2268, 1540, 1340, 756 cm^{-1}; ^1H NMR (400 MHz, $CDCl_3$) δ 8.10 (s, 2H), 7.89 (d, *J* = 8.0 Hz, 2H), 7.78 – 7.59 (m, 6H), 7.42-7.38(m, 2H), 7.27 – 7.32 (m, 8H), 7.21 – 7.11 (m, 4H), 7.09 – 7.02 (m, 2H), 7.02 – 6.95 (m, 2H), 6.93 – 6.85 (m, 2H), 3.73 (s, 6H), 3.56 – 3.48 (m, 2H), 1.05 – 0.69 (m, 8H), 0.14-0.16 (m, 12H); ^{13}C NMR (100 MHz, $CDCl_3$) δ 150.7, 135.6, 132.9, 131.7, 130.4, 129.5, 129.1， 128.6， 128.4, 128.1, 127.2, 126.0, 125.6, 125.2, 125.1, 123.7, 123.7,

123.7, 123.0, 122.1, 120.2, 119.6, 111.6, 111.1, 110.4, 106.2, 84.4, 53.2, 24.8, 9.0; HRMS (ESI) calcd for $C_{64}H_{58}N_6O_6Na$ *m/z* [M + Na]$^+$: 1029.4310; found: 1029.4321. HPLC (Daicel Chiralpak IA, *i*-PrOH/hexane = 20/80, flow rate 0.8 mL/min, λ = 260 nm): t_1 (major) =21.1 min, t_2 (minor) =25.7 min.

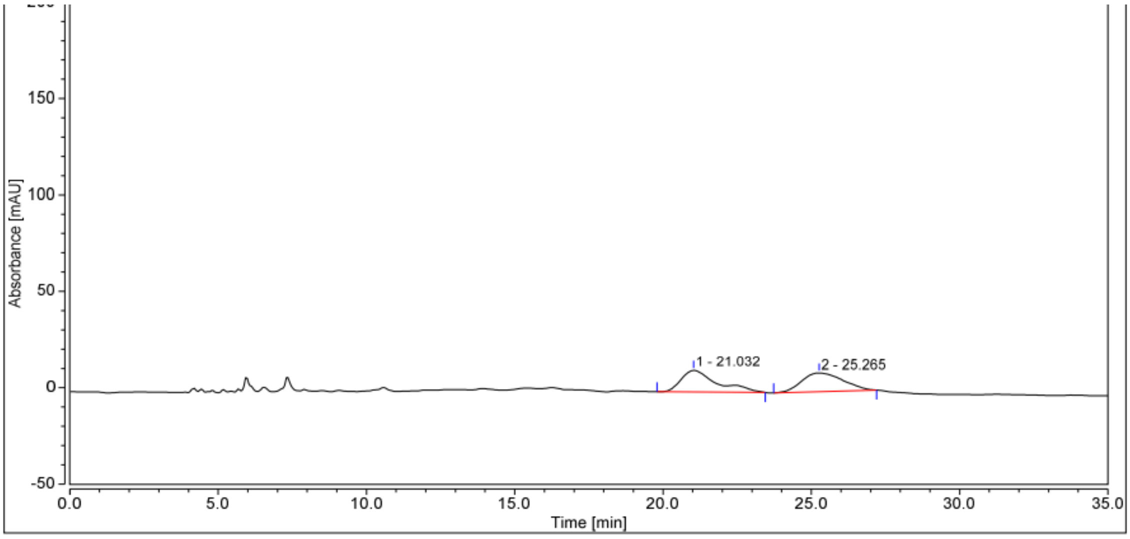

Integration Results

No.	Peak Name	Retention Time min	Area mAU*min	Height mAU	Relative Area %	Relative Height %	Amount n.a.
1		21.032	15.722	11.222	50.32	53.75	n.a.
2		25.265	15.522	9.656	49.68	46.25	n.a.
**Total:**			**31.244**	**20.878**	**100.00**	**100.00**	

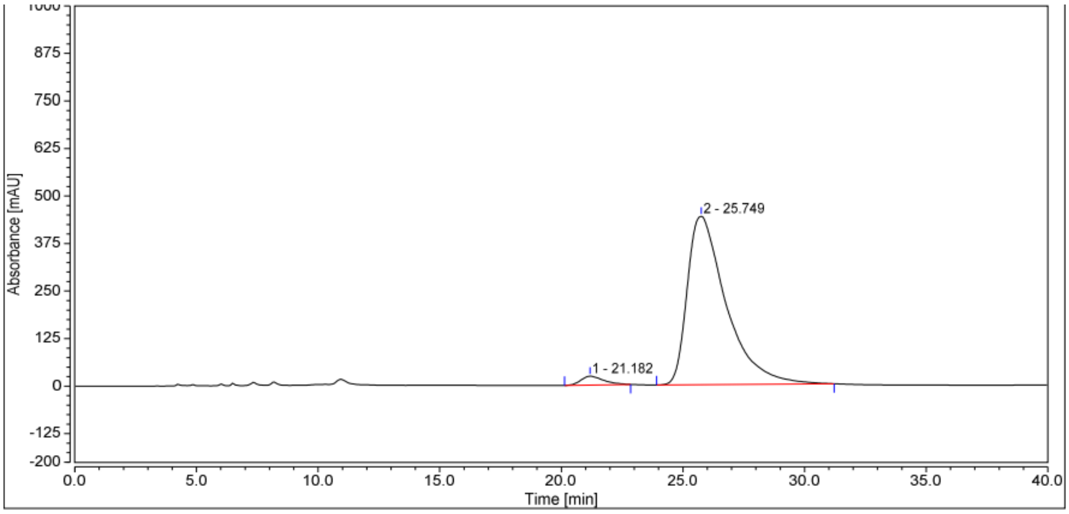

Integration Results

No.	Peak Name	Retention Time min	Area mAU*min	Height mAU	Relative Area %	Relative Height %	Amount n.a.
1		21.182	25.236	23.302	2.99	5.00	n.a.
2		25.749	819.939	442.966	97.01	95.00	n.a.
**Total:**			**845.175**	**466.268**	**100.00**	**100.00**	

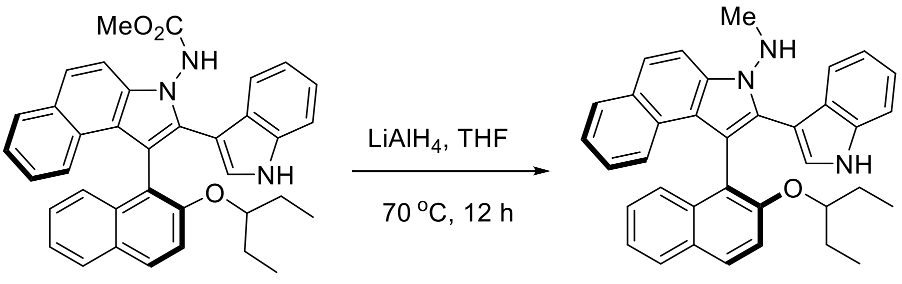

15a, (97% ee)   **17**, 65%, 96% ee

(*S*)-2-(1H-Indol-3-yl)-N-methyl-1-(2-(pentan-3-yloxy)naphthalen-1-yl)-3H-benzo[e]indol-3-amine (17)

Compound **15a** (113.0 mg, 0.2 mmol, 1.0 eq, 97% ee) was treated with $LiAlH_4$ (23.0 mg, 0.6 mmol, 3.0 eq) in THF (2.0 mL) at 70 °C for 12 h. After finished, the reaction was quenched with H_2O (5 mL). The solution was extracted with CH_2Cl_2 (3×5 mL). The combined extracts were washed with brine (8.0 mL), dried (Na_2SO_4), filtered and concentrated in vacuo. The crude product was purified by flash column chromatography (20% EtOAc in hexane) gave **17** (68.0 mg, 65% yield, 96% ee); White solid, mp 112.0-113.0 °C; $[\alpha]_D^{24}$ +95.6 (*c* 1.0, $CHCl_3$, 96% ee); IR (KBr): 3426, 2965, 2755, 1460, 1240, 752 cm^{-1}; ^1H NMR (400 MHz, $CDCl_3$) δ 7.90 (d, *J* = 8.8 Hz, 1H), 7.87 – 7.82 (m, 2H), 7.79 (d, *J* = 8.9 Hz, 1H), 7.75 (d, *J* = 8.0 Hz, 1H), 7.68 (d, *J* = 8.8 Hz, 1H), 7.54 (d, *J* = 7.9 Hz, 2H), 7.27 (d, *J* = 8.9 Hz, 1H), 7.22 – 7.16 (m, 3H), 7.09 (m, 5H), 6.95 (t, *J* = 7.6 Hz, 1H), 5.14 (s, 1H), 3.94 (p, *J* = 5.5 Hz, 1H), 2.56 (s, 3H), 1.39 – 1.30 (m, 2H), 1.19 (m, 2H), 0.53 (t, *J* = 7.4 Hz, 3H), 0.30 (t, *J* = 7.4 Hz, 3H); ^{13}C NMR (100 MHz, $CDCl_3$) δ 154.9, 135.7, 135.7, 132.6, 130.4, 129.8, 129.4, 128.6, 128.5, 128.3, 127.5, 126.9, 126.2, 126.0, 125.1, 124.8, 123.7, 123.3, 123.0, 122.6, 122.5, 122.2, 120.5, 120.2, 119.9, 118.8, 111.4, 109.9, 106.4, 82.5, 39.6, 25.8, 9.2, 8.2; HRMS (ESI) calcd for $C_{36}H_{33}N_3ONa$ *m/z* $[M + Na]^+$: 546.2516; found: 546.2518; HPLC (Daicel Chiralpak IA, *i*-PrOH/hexane = 10/90, flow rate 0.8 mL/min, λ = 260 nm): t_1 (major) = 14.0 min, t_2 (minor) = 15.2 min.

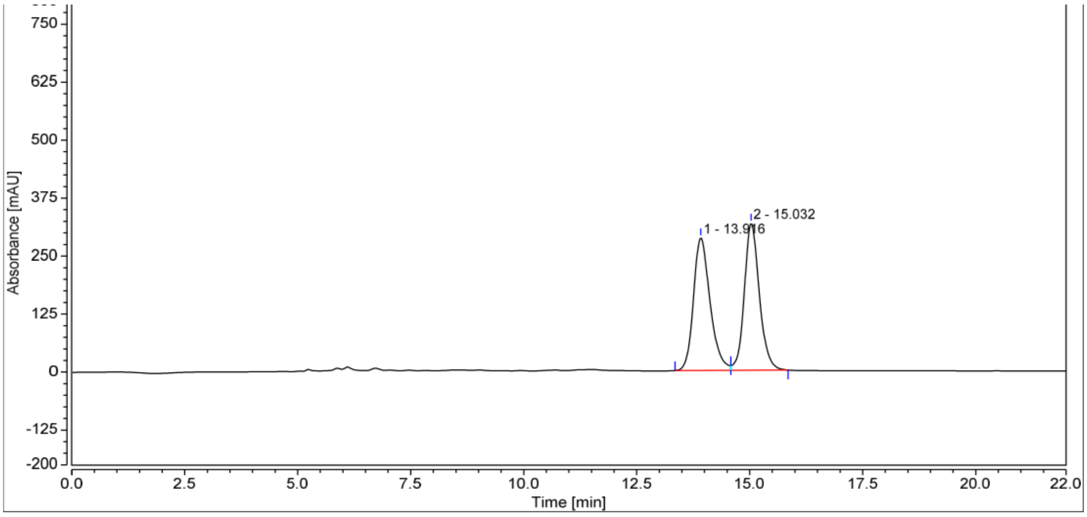

Integration Results							
No.	Peak Name	Retention Time min	Area mAU*min	Height mAU	Relative Area %	Relative Height %	Amount n.a.
1		13.916	122.180	286.239	49.95	47.42	n.a.
2		15.032	122.430	317.346	50.05	52.58	n.a.
**Total:**			**244.609**	**603.585**	**100.00**	**100.00**	

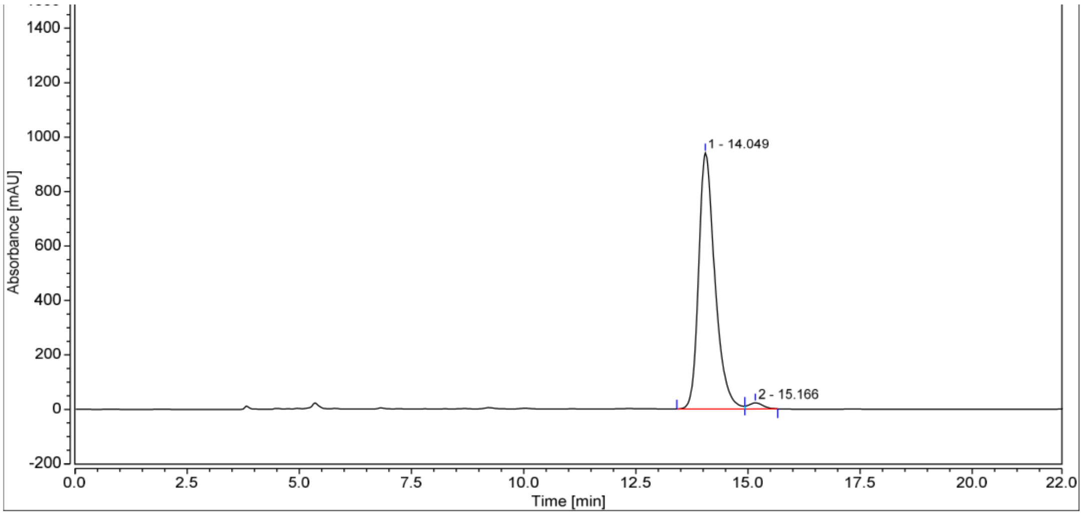

Integration Results							
No.	Peak Name	Retention Time min	Area mAU*min	Height mAU	Relative Area %	Relative Height %	Amount n.a.
1		14.049	397.223	940.832	97.96	97.66	n.a.
2		15.166	8.268	22.504	2.04	2.34	n.a.
**Total:**			**405.491**	**963.336**	**100.00**	**100.00**	

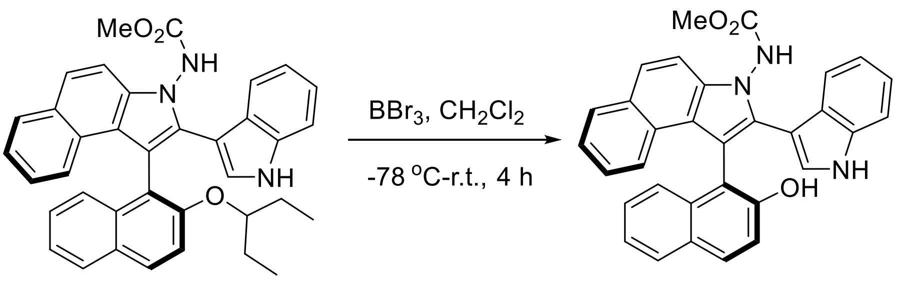

15a, (97% ee) **18**, 99%, 97% ee

(*S*)-Methyl-(1-(2-hydroxynaphthalen-1-yl)-2-(1H-indol-3-yl)-3H-benzo[e]indol-3-yl)carbamate (18)

To a mixture of **15a** (113.0 mg, 0.2 mmol, 1.0 eq, 97% ee) in CH_2Cl_2 (2 mL) at -78 ℃ was added BBr_3 (0.2mL 1.0 mol/L CH_2Cl_2) dropwise, and the mixture was stirred for 30 minutes at the room temperature. TLC showed complete consumption of the starting material. The reaction was quenched with 10 % $NaHCO_3$ solution and extracted with CH_2Cl_2 (3×5 mL). The combined organics were washed with brine and dried over Na_2SO_4. The solvent was removed under reduced pressure, the residue was purified by column chromatography on silica gel (20% EtOAc in hexane) to yield triflate **18** as a white solid (98.4 mg, 99% yield, 97% ee); mp 154.0-155.0 ℃; $[\alpha]_D^{24}$ -89.6 (*c* 1.0, $CHCl_3$, 97% ee); IR (KBr): 3418, 2955, 1460, 1242, 742 cm^{-1}; ^1H NMR (400 MHz, Acetone-d_6) δ 10.25 (s, 1H), 9.80 (s, 1H), 7.95 (d, *J* = 8.1 Hz, 1H), 7.87 – 7.75 (m, 7H), 7.44 (d, *J* = 8.2 Hz, 1H), 7.37 – 7.18 (m, 6H), 7.07 (dt, *J* = 11.6, 5.9 Hz, 2H), 7.01 – 6.88 (m, 1H), 3.69 (s, 3H); ^{13}C NMR (100 MHz, Acetone-d_6) δ 157.1, 154.2, 136.8, 136.1, 135.3, 134.6, 131.0, 129.9, 129.5, 129.4, 129.3, 128.6, 128.3, 126.9, 126.8, 126.1, 125.8, 123.9, 123.7, 123.4, 123.4, 122.2, 121.0, 120.7, 120.0, 118.7, 116.4, 112.2, 111.6 108.8, 105.7, 52.9; HRMS (ESI) calcd for $C_{32}H_{23}N_3O_3Na$ *m/z* [M + Na]$^+$: 520.1632; found: 520.1634; HPLC (Daicel Chiralpak IA, *i*-PrOH/hexane = 20/80, flow rate 0.8 mL/min, λ = 230 nm): t_1 (major) = 9.8 min, t_2 (minor) = 15.9 min.

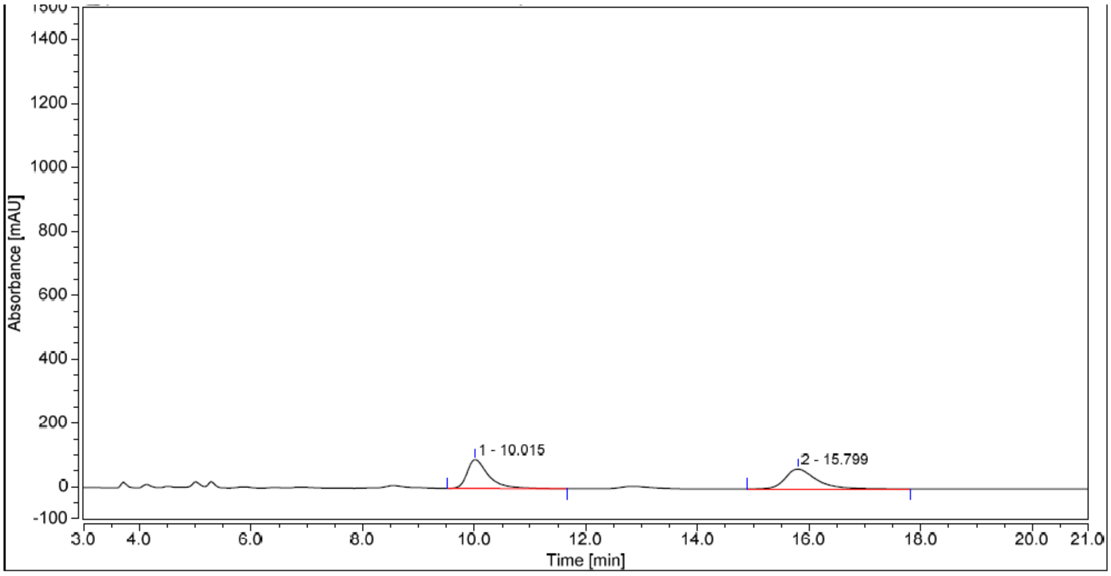

Integration Results

No.	Peak Name	Retention Time<br>min	Area<br>mAU*min	Height<br>mAU	Relative Area<br>%	Relative Height<br>%	Amount<br>n.a.
1		10.015	40.300	91.212	50.10	59.42	n.a.
2		15.799	40.145	62.294	49.90	40.58	n.a.
**Total:**			**80.445**	**153.506**	**100.00**	**100.00**	

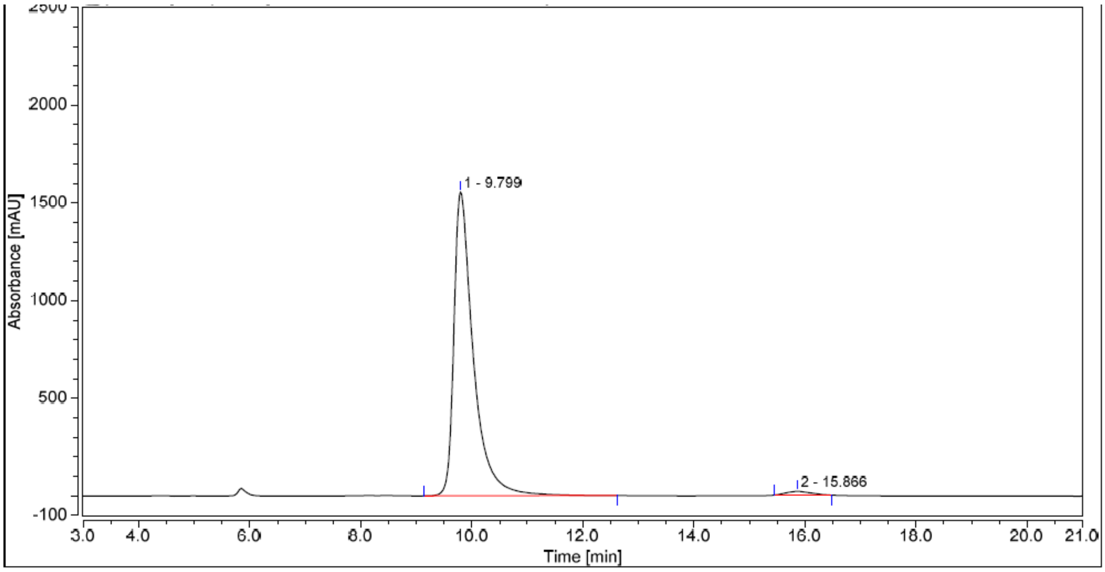

Integration Results

No.	Peak Name	Retention Time<br>min	Area<br>mAU*min	Height<br>mAU	Relative Area<br>%	Relative Height<br>%	Amount<br>n.a.
1		9.799	629.526	1553.855	98.47	98.80	n.a.
2		15.866	9.806	18.842	1.53	1.20	n.a.
**Total:**			**639.332**	**1572.697**	**100.00**	**100.00**	

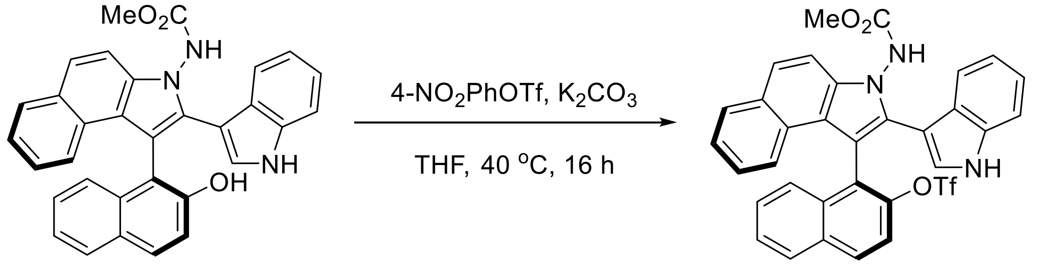

18, (97% ee) **19**, 95%, 97% ee

(*S*)-1-(2-(1H-Indol-3-yl)-3-((methoxycarbonyl)amino)-3H-benzo[e]indol-1-yl)naphthalen-2-yl trifluoromethanesulfonate (19)

The reaction was performed using **18** (98.4 mg, 0.2 mmol, 1.0eq, 97% ee), 4-nitrophenyl trifluoromethanesulfonate (65.0 mg, 0.24 mmol, 1.2 eq), and K_2CO_3 (41.5 mg, 0.3 mmol, 1.5 eq) in THF (2.0 mL) at 40 °C. After finished, the reaction was quenched with H_2O (5 mL). The solution was extracted with CH_2Cl_2 (3×5 mL), washed with brine (5.0 mL) and dried over anhydrous Na_2SO_4. The solvent was evaporated under reduced pressure. The crude product was purified by flash column chromatography (10% EtOAc in hexane) giving **19** as a white solid (119.5 mg, 95% yield, 97% ee); mp 251.0 - 252.0 °C; $[\alpha]_D^{24}$ +70.4 (*c* 0.5, acetone, 97% ee); IR (KBr): 3422, 2965, 1468, 1242, 732 cm^{-1}; ^1H NMR (400 MHz, Acetone-d_6) δ 10.37 (d, *J* = 20.8 Hz, 1H), 8.05 (t, *J* = 7.7 Hz, 3H), 7.94 (d, *J* = 8.1 Hz, 1H), 7.86 – 7.71 (m, 3H), 7.57 (s, 1H), 7.43 (d, *J* = 8.4 Hz, 3H), 7.33 (s, 1H), 7.27 (t, *J* = 7.5 Hz, 1H), 7.17 (s, 2H), 6.99 (q, *J* = 7.9 Hz, 2H), 6.83 (s, 1H), 3.66 (s, 3H); ^{13}C NMR (100 MHz, Acetone-d_6) δ 206.2, 157.0, 156.6, 146.5, 136.8, 135.6, 135.0, 133.3, 131.3, 131.0, 129.3, 129.1, 128.6, 128.5, 127.9, 127.8, 127.0, 125.9, 124.5, 124.0, 123.9, 122.3, 120.8, 120.6, 120.2, 120.0, 117.4, 112.2, 111.5, 107.3, 105.3, 52.9; HRMS (ESI) calcd for $C_{33}H_{22}F_3N_3O_5SNa$ *m/z* $[M + Na]^+$: 652.1124; found: 652.1125; HPLC (Daicel Chiralpak IB, *i*-PrOH/hexane = 10/90, flow rate 0.8 mL/min, λ = 230 nm): t_1 (major) = 29.2 min, t_2 (minor) = 36.5 min.

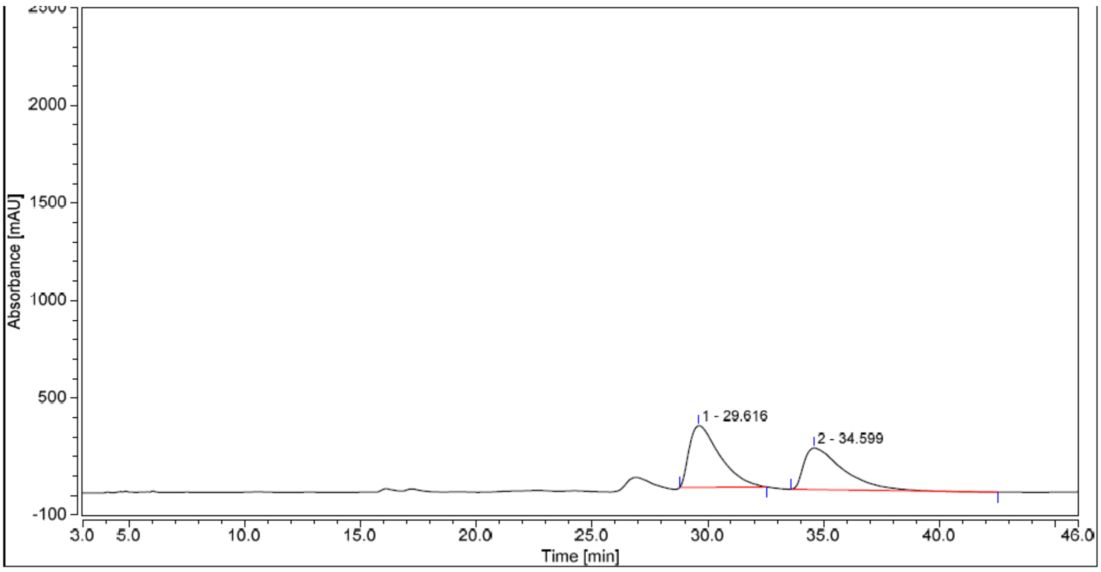

Integration Results							
No.	Peak Name	Retention Time<br>min	Area<br>mAU*min	Height<br>mAU	Relative Area<br>%	Relative Height<br>%	Amount<br>n.a.
1		29.616	487.155	316.831	53.82	59.75	n.a.
2		34.599	418.042	213.386	46.18	40.25	n.a.
**Total:**			**905.198**	**530.217**	**100.00**	**100.00**	

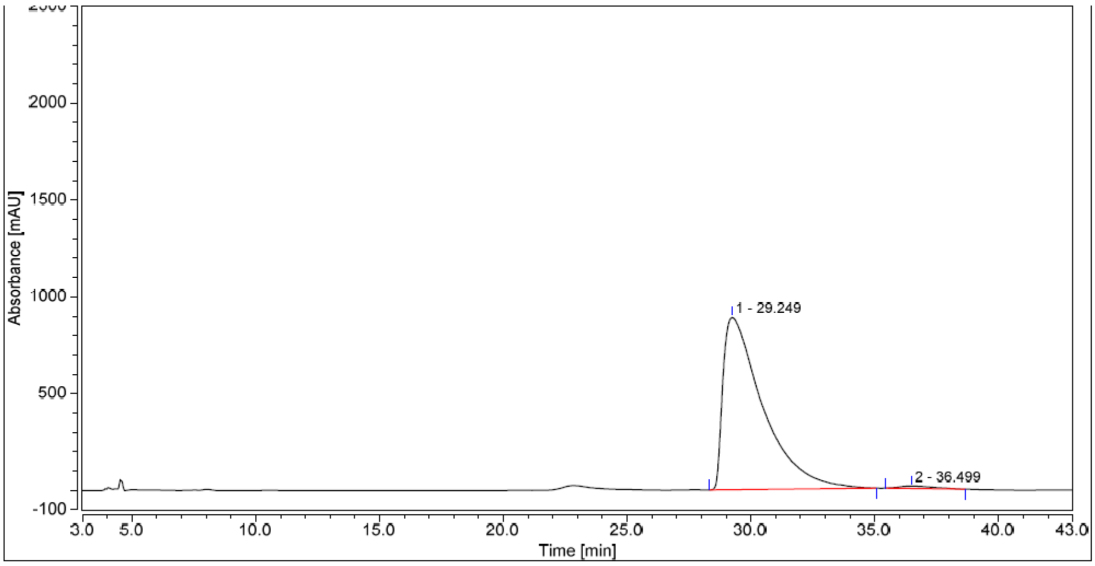

Integration Results							
No.	Peak Name	Retention Time<br>min	Area<br>mAU*min	Height<br>mAU	Relative Area<br>%	Relative Height<br>%	Amount<br>n.a.
1		29.249	1659.288	889.714	98.78	98.59	n.a.
2		36.499	20.500	12.691	1.22	1.41	n.a.
**Total:**			**1679.787**	**902.404**	**100.00**	**100.00**	

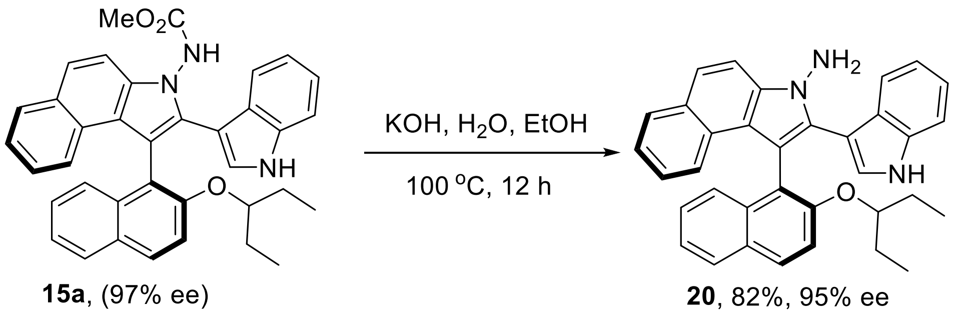

(*S*)-2-(1H-Indol-3-yl)-1-(2-(pentan-3-yloxy)naphthalen-1-yl)-3H-benzo[e]indol-3-amine (20)

A solution of KOH (112.2 mg, 2.0 mmol, 10.0 eq) in EtOH (2.0 mL) and H_2O was stirred at room temperature for 10 min at under N_2. Subsequently, **15a** (113.0 mg, 0.2 mmol, 1.0 eq, 95% ee) was added. The reaction was stirred at 100 °C for 12 h. The solution was extracted with CH_2Cl_2 (3×5 mL), washed with brine (8.0 mL) and dried over anhydrous Na_2SO_4. The solvent was evaporated under reduced pressure. The crude product was purified by flash column chromatography (30% EtOAc in hexane) gave **20** (83.5 mg, 82% yield, 95% ee); Blackish green solid, mp 134.0-135.0 ℃; $[\alpha]_D^{24}$ +100.4 (*c* 0.25, $CHCl_3$, 95% ee); IR (KBr): 3425, 2962, 1508, 1242, 722 cm^{-1}; ^1H NMR (400 MHz, $CDCl_3$) δ 7.93 (d, *J* = 8.8 Hz, 1H), 7.88 – 7.83 (m, 2H), 7.80 (d, *J* = 8.8 Hz, 1H), 7.76 (d, *J* = 8.0 Hz, 1H), 7.68 (d, *J* = 8.8 Hz, 1H), 7.58 (d, *J* = 8.4 Hz, 1H), 7.56 – 7.51 (m, 1H), 7.26 (d, *J* = 9.0 Hz, 2H), 7.23 – 7.15 (m, 3H), 7.15 – 7.08 (m, 3H), 7.00 – 6.93 (m, 2H), 4.77 (s, 2H), 4.04 – 3.77 (m, 1H), 1.36 – 1.24 (m, 2H), 1.20 – 1.12 (m, 2H), 0.51 (t, *J* = 7.4 Hz, 3H), 0.28 (t, *J* = 7.4 Hz, 3H); ^{13}C NMR (100 MHz, $CDCl_3$) δ 154.8, 135.8, 135.8, 134.3, 131.9, 129.8, 129.3, 128.6, 128.5, 128.3, 127.6, 127.1, 126.2, 126.1, 125.4, 125.1, 123.6, 123.1, 122.8, 122.6, 122.6, 122.5, 122.2, 120.6, 119.7, 119.2, 119.1, 111.5, 111.4, 109.1, 106.5, 82.1, 25.9, 9.3, 8.7; HRMS (ESI) calcd for $C_{35}H_{31}N_3ONa$ *m/z* $[M + Na]^+$: 532.2359; found: 532.2357; HPLC (Daicel Chiralpak IE, *i*-PrOH/hexane = 10/90, flow rate 0.8 mL/min, λ = 230 nm): t_1 (minor) = 7.6 min, t_2 (major) = 8.1 min.

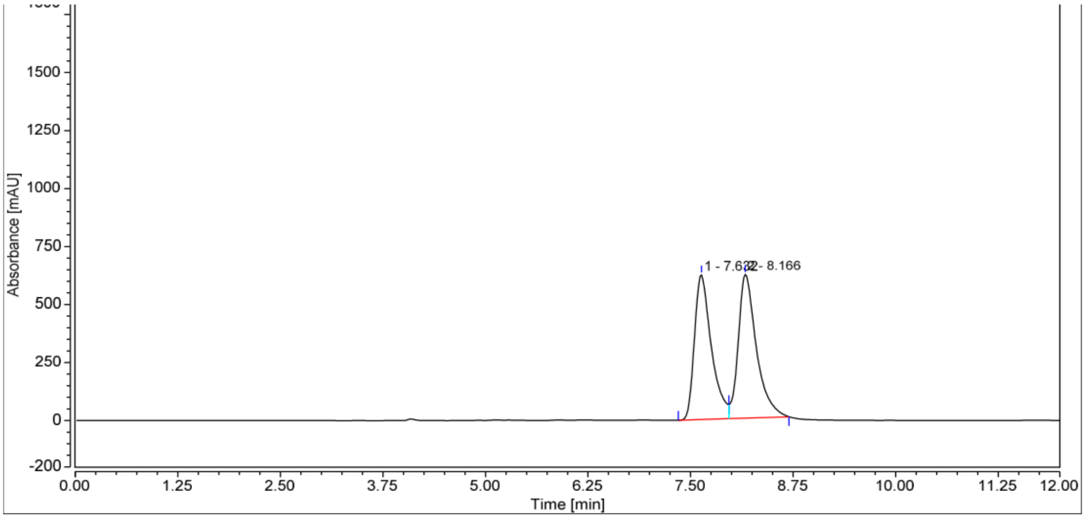

Integration Results							
No.	Peak Name	Retention Time<br>min	Area<br>mAU*min	Height<br>mAU	Relative Area<br>%	Relative Height<br>%	Amount<br>n.a.
1		7.632	148.880	623.241	48.42	50.15	n.a.
2		8.166	158.616	619.443	51.58	49.85	n.a.
**Total:**			**307.496**	**1242.684**	**100.00**	**100.00**	

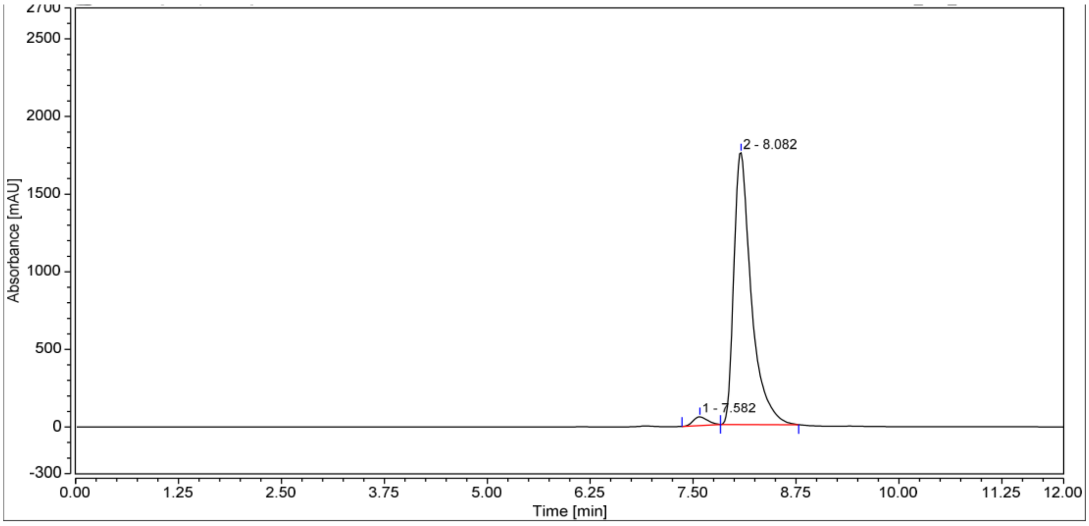

Integration Results							
No.	Peak Name	Retention Time<br>min	Area<br>mAU*min	Height<br>mAU	Relative Area<br>%	Relative Height<br>%	Amount<br>n.a.
1		7.582	11.276	55.212	2.55	3.06	n.a.
2		8.082	430.766	1749.853	97.45	96.94	n.a.
**Total:**			**442.042**	**1805.065**	**100.00**	**100.00**	

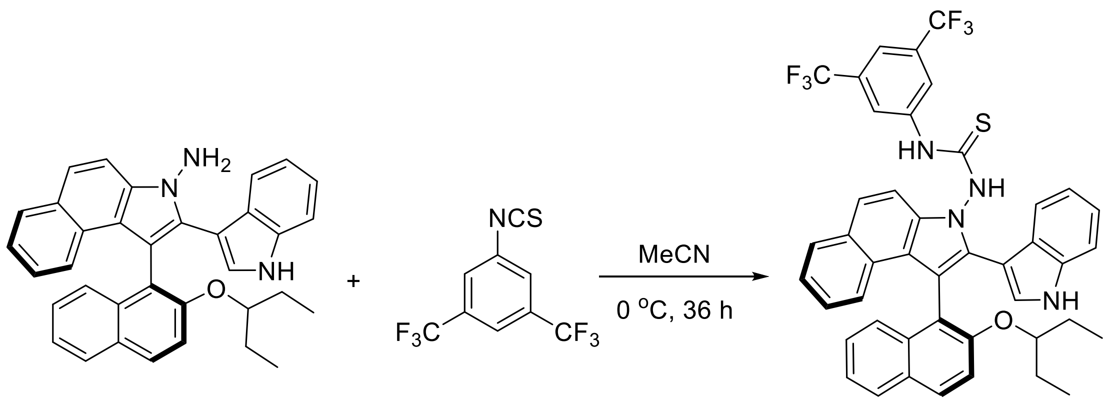

20, 95% ee **21**, 90%, 96% ee

(*S*)-1-(2-(1H-Indol-3-yl)-1-(2-(pentan-3-yloxy)naphthalen-1-yl)-3H-benzo[e]indol-3-yl)-3-(3,5-bis(trifluoromethyl)phenyl)thiourea (21)[8]

To the solution of **20** (20 mg, 0.04 mmol) in MeCN (1 mL) was added 3,5-Bis(trifluoromethyl)phenyl isothiocyanate (21.1 mg, 0.08 mmol, 2.0 eq.). Then, the reaction mixture was stirred at 25 °C for 36 h. After the completion of the reaction which was indicated by TLC, the reaction mixture was purified through flash column chromatography on silica gel (25% EtOAc in P.E.) to afford pure product **21** (26.7 mg, 90% yield, 96% ee); Brown solid, mp 151.0-152.0 ℃; $[\alpha]_D^{24}$ -62.7 (*c* 1.0, $CHCl_3$, 96% ee); IR (KBr): 3216, 2863, 1840, 1558, 1242 cm^{-1}; ^1H NMR (400 MHz, $CDCl_3$) δ 9.08 – 8.81 (m, 1H), 8.01 (d, *J* = 6.4 Hz, 1H), 7.91 – 7.79 (m, 3H), 7.79 – 7.72 (m, 2H), 7.71 – 7.56 (m, 3H), 7.55 – 7.40 (m, 3H), 7.37 – 7.27 (m, 3H), 7.25 – 7.18 (m, 2H), 7.17 – 7.09 (m, 2H), 7.04 (dt, *J* = 8.1, 7.3 Hz, 2H), 6.97 – 6.84 (m, 1H), 4.31 – 3.85 (m, 1H), 1.39 – 1.05 (m, 4H), 0.64 (t, *J* = 7.4 Hz, 2H), 0.42 (td, *J* = 7.3, 1.8 Hz, 1H), 0.29 (t, *J* = 7.4 Hz, 1H), 0.17 (td, *J* = 7.3, 2.1 Hz, 2H); ^{13}C NMR (100 MHz, $CDCl_3$) δ 182.2, 154.6, 138.7, 135.6, 134.3, 133.3, 133.0, 132.4, 132.0, 131.7, 130.9, 130.6, 129.7, 128.9, 128.6, 128.3, 127.7, 126.6, 126.6, 126.3, 125.3, 125.2, 125.1, 124.2, 124.0, 123.6, 123.3, 122.6, 121.4, 121.2, 120.7, 119.2, 118.8, 117.4, 115.5, 113.0, 111.5, 109.9, 104.7, 80.6, 25.8, 25.6, 9.5, 8.2; HRMS (ESI) calcd for $C_{44}H_{34}F_6N_4OS$ *m/z* $[M + Na]^+$: 803.2250; found: 803.2246; HPLC (Daicel Chiralpak IA, *i*-PrOH/hexane = 5/95, flow rate 0.6 mL/min, *λ* = 260 nm): t_1 (major) = 21.1 min, t_2 (minor) = 27.9 min.

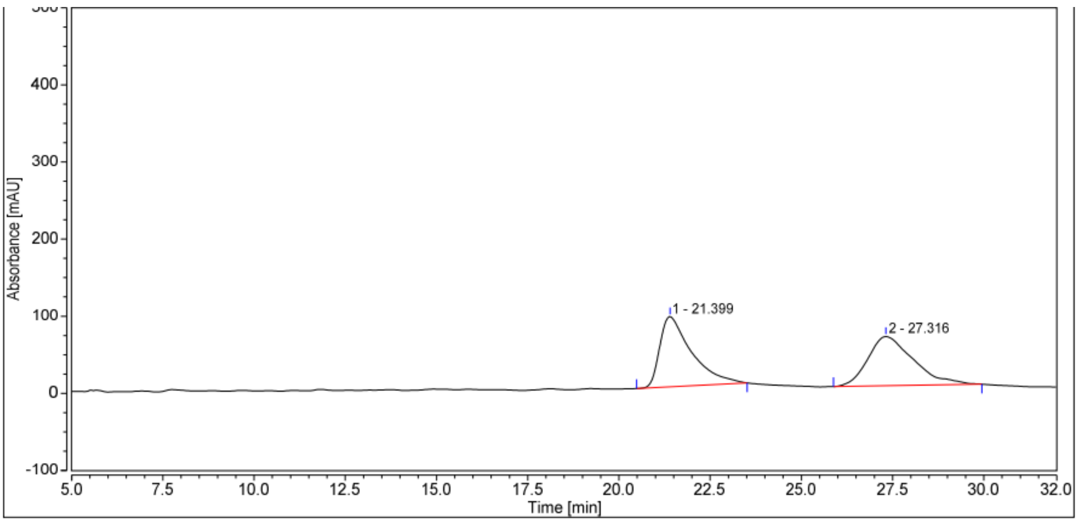

Integration Results

No.	Peak Name	Retention Time min	Area mAU*min	Height mAU	Relative Area %	Relative Height %	Amount n.a.
1		21.399	91.798	91.036	49.89	58.80	n.a.
2		27.316	92.217	63.800	50.11	41.20	n.a.
**Total:**			**184.015**	**154.836**	**100.00**	**100.00**	

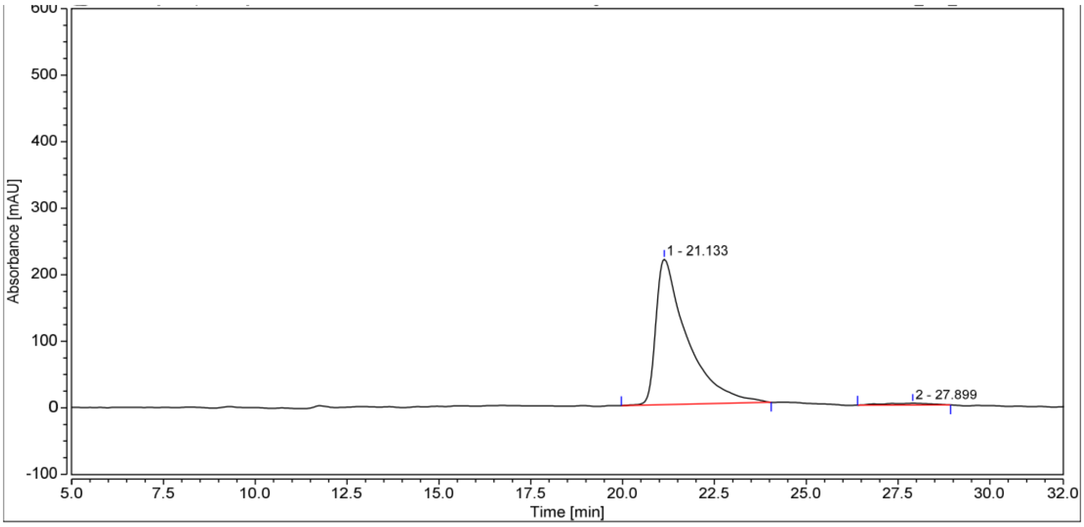

Integration Results

No.	Peak Name	Retention Time min	Area mAU*min	Height mAU	Relative Area %	Relative Height %	Amount n.a.
1		21.133	216.456	218.617	98.28	98.82	n.a.
2		27.899	3.788	2.608	1.72	1.18	n.a.
**Total:**			**220.244**	**221.226**	**100.00**	**100.00**	

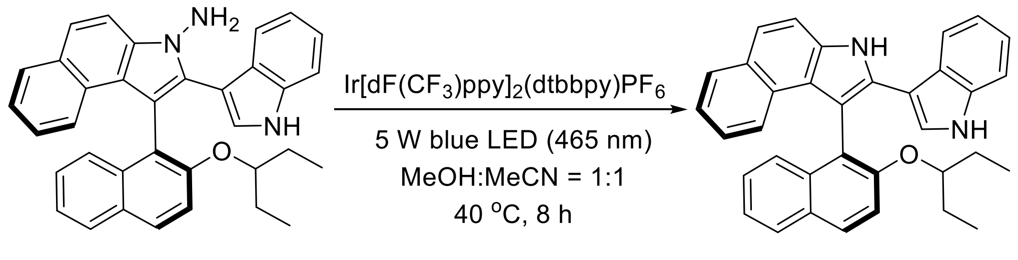

20, 98% ee

22, 35%, 98% ee

(*S*)-2-(1H-Indol-3-yl)-1-(2-(pentan-3-yloxy)naphthalen-1-yl)-3H-benzo[e]indole (22)[9]

The reaction vial with a stirring bar was charged with Ir[dF(CF_3)ppy]$_2$(dtbbpy)PF_6[dF(CF_3)ppy = 2-(2,4-difluorophenyl)-5-trifluoromethyl-pyridine, dtbbpy = 4,4'-di-tert-butyl-2,2'-bipyridine] (4.6 mg, 0.004 mmol), **20** (101.9 mg, 0.2 mmol) and methanol/acetonitrile (1:1, 2 mL). The solution was sealed with a Teflon-lined cap and heated to 40 ℃ under the irradiation of a 5 W blue LED at a wavelength of 465 nm for 8 h. When the reaction was complete, the solvent was removed under reduced pressure, and the residue was purified via silica gel chromatography (eluent:petroleum ether/ethyl acetate = 10:1) to afford **22** as a yellow solid (69.9 mg, 35% yield); mp 126.0-127.0 ℃; $[a]_D^{24}$ -32.8 (*c* 1.0, $CHCl_3$, 98% ee); IR (KBr): 3143, 2956, 1862, 1365, 852 cm^{-1}; ^{1}H NMR (400 MHz, $CDCl_3$) δ 8.96 (s, 1H), 8.00 – 7.84 (m, 4H), 7.82 (s, 1H), 7.72 – 7.57 (m, 3H), 7.39 – 7.27 (m, 3H), 7.24 – 7.12 (m, 5H), 7.05 – 6.95 (m, 1H), 6.69 (d, *J* = 2.6 Hz, 1H), 4.07 – 3.82 (m, 1H), 1.40 – 1.06 (m, 4H), 0.49 (t, *J* = 7.4 Hz, 3H), 0.33 (t, *J* = 7.4 Hz, 3H); ^{13}C NMR (100 MHz, $CDCl_3$) δ 154.6, 135.6, 135.5, 132.3, 130.3, 129.8, 129.7, 128.8, 128.5, 128.3, 127.7, 126.5, 126.0, 125.5, 125.1, 124.0, 123.5, 123.1, 123.0, 122.7, 122.3, 122.1, 120.4, 120.1, 118.9, 112.4, 111.4, 108.9, 108.3, 99.8, 82.8, 25.8, 8.9; HRMS (ESI) calcd for $C_{35}H_{30}N_2ONa$ *m/z* $[M + Na]^+$: 517.2250; found: 517.2246; HPLC (Daicel Chiralpak IA, *i*-PrOH/hexane = 10/90, flow rate 0.8 mL/min, λ = 260 nm): t_1 (major) = 36.2 min, t_2 (minor) = 49.5 min.

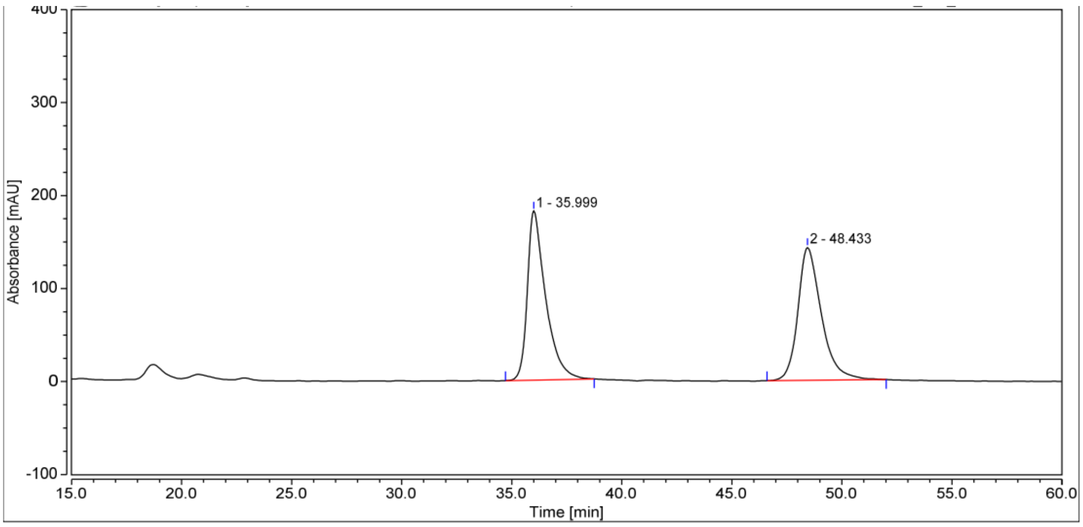

Integration Results							
No.	Peak Name	Retention Time min	Area mAU*min	Height mAU	Relative Area %	Relative Height %	Amount n.a.
1		35.999	175.563	181.894	49.87	56.03	n.a.
2		48.433	176.497	142.753	50.13	43.97	n.a.
**Total:**			**352.060**	**324.648**	**100.00**	**100.00**	

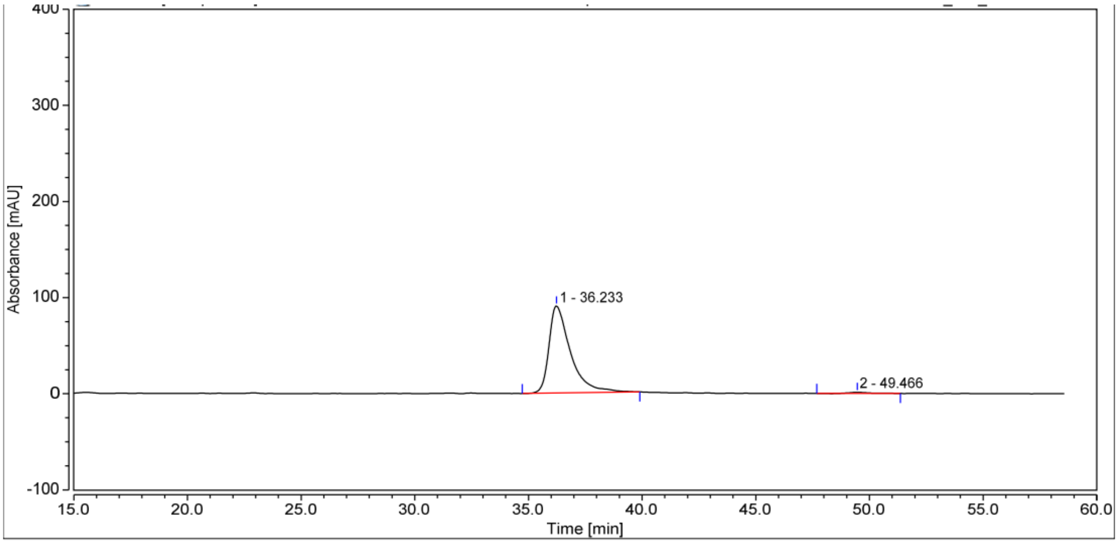

Integration Results							
No.	Peak Name	Retention Time min	Area mAU*min	Height mAU	Relative Area %	Relative Height %	Amount n.a.
1		36.233	99.527	90.635	98.90	98.92	n.a.
2		49.466	1.108	0.987	1.10	1.08	n.a.
**Total:**			**100.635**	**91.623**	**100.00**	**100.00**	

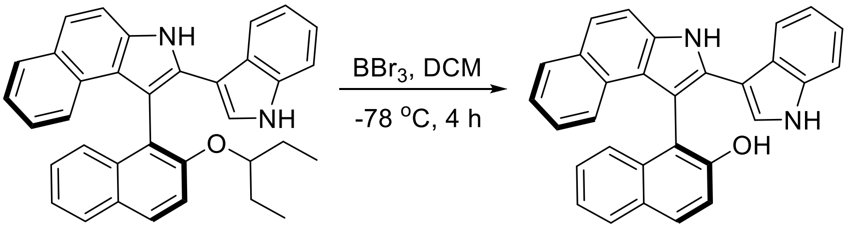

(*S*)-1-(2-(1H-Indol-3-yl)-3H-benzo[e]indol-1-yl)naphthalen-2-ol (23)

To a mixture of **22** (98.9 mg, 0.2 mmol, 1.0 eq, 98% ee) in CH_2Cl_2 (4 mL) at -78 ℃ was added BBr_3 (0.4mL, 1.0 mol/L CH_2Cl_2) dropwise, and the mixture was stirred for 30 minutes at the room temperature. After TLC showed complete consumption of the starting material, the reaction was quenched with H_2O and extracted with CH_2Cl_2 (3×5 mL). The combined organics were washed with brine and dried over Na_2SO_4. The solvent was removed under reduced pressure, the residue was purified by column chromatography on silica gel (30% EtOAc in hexane) to yield **23** as a white solid (78.9 mg, 93% yield); mp 135.0-136.0 ℃; $[a]_D^{24}$ -54.6 (*c* 1.0, $CHCl_3$, 98% ee); IR (KBr): 3420, 2968, 1648, 1365, 768 cm^{-1}; ^1H NMR (400 MHz, $CDCl_3$) δ 9.08 (s, 1H), 7.91 (dd, *J* = 13.4, 8.1 Hz, 4H), 7.78 – 7.59 (m, 3H), 7.53 (d, *J* = 8.4 Hz, 1H), 7.42 – 7.27 (m, 4H), 7.19 (d, *J* = 7.3 Hz, 4H), 7.07 (t, *J* = 7.6 Hz, 1H), 6.56 (s, 1H), 5.67 (s, 1H); ^{13}C NMR (100 MHz, $CDCl_3$) δ 152.1, 135.6, 134.5, 133.0, 131.6, 129.8, 129.1, 128.4, 128.3, 128.1, 126.8, 125.9, 125.2, 125.2, 123.6, 123.4, 123.4, 122.6, 122.5, 122.5, 120.7, 118.8, 117.1, 115.6, 112.4, 111.7, 107.2, 104.3; HRMS (ESI) calcd for $C_{30}H_{20}N_2ONa$ *m/z* $[M + Na]^+$: 447.1468; found: 447.1468; HPLC (Daicel Chiralpak IC, *i*-PrOH/hexane = 20/80, flow rate 0.8 mL/min, λ = 260 nm): t_1 (major) = 8.0 min, t_2 (minor) = 10.7 min.

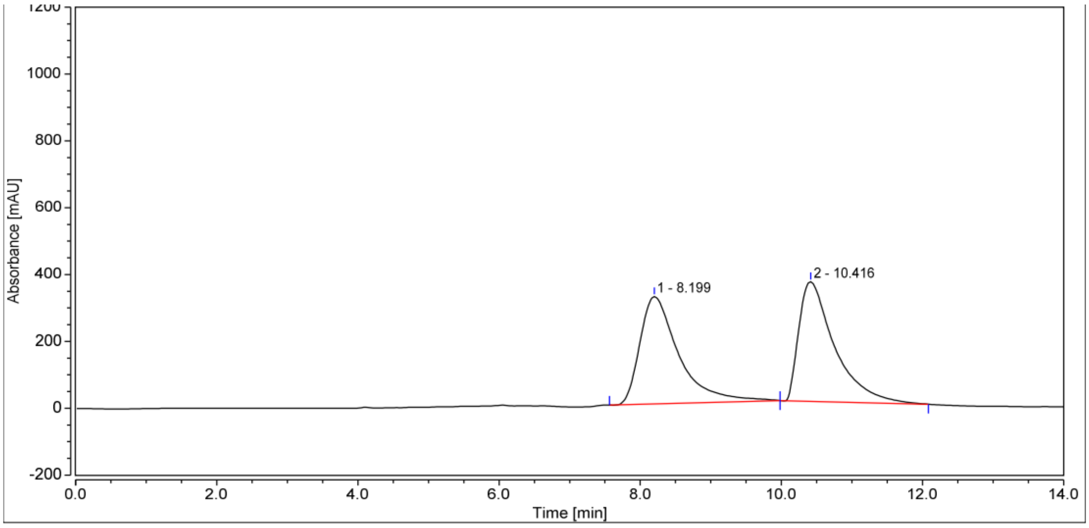

Integration Results							
No.	Peak Name	Retention Time min	Area mAU*min	Height mAU	Relative Area %	Relative Height %	Amount n.a.
1		8.199	205.185	321.060	49.45	47.29	n.a.
2		10.416	209.734	357.802	50.55	52.71	n.a.
**Total:**			**414.919**	**678.862**	**100.00**	**100.00**	

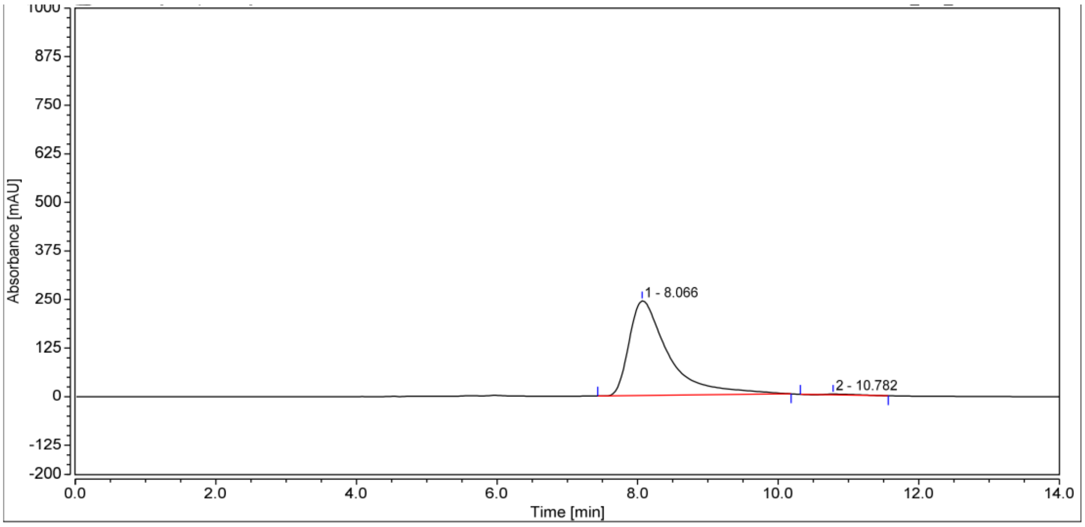

Integration Results							
No.	Peak Name	Retention Time min	Area mAU*min	Height mAU	Relative Area %	Relative Height %	Amount n.a.
1		8.066	158.613	243.811	99.40	99.21	n.a.
2		10.782	0.958	1.947	0.60	0.79	n.a.
**Total:**			**159.572**	**245.758**	**100.00**	**100.00**	

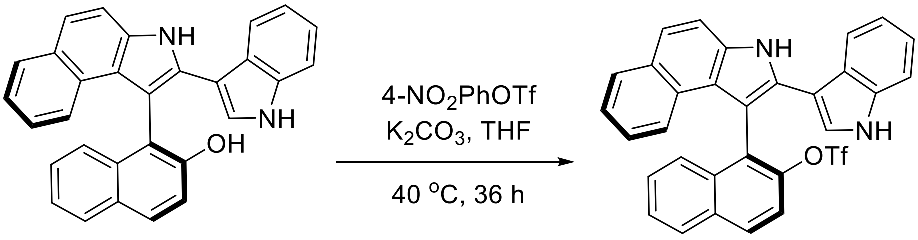

23, 93%, 98% ee　　**23'**, 84%, 93% ee

(*S*)-1-(2-(1H-Indol-3-yl)-3H-benzo[e]indol-1-yl)naphthalen-2-yl trifluoromethanesulfonate (23')

The reaction was performed using **23** (212.2 mg, 0.5 mmol, 1.0 eq, 98% ee), 4-nitrophenyl trifluoromethanesulfonate (270.9 mg, 1 mmol, 2.0 eq.), and K_2CO_3 (207.0 mg, 1.5 mmol, 3.0 eq.) in THF (10.0 mL) at 40 °C. After finished, the reaction was quenched with H_2O (10 mL). The solution was extracted with CH_2Cl_2 (3×15 mL), washed with brine (20.0 mL) and dried over anhydrous Na_2SO_4. The solvent was evaporated under reduced pressure. The crude product was purified by flash column chromatography (20% EtOAc in hexane) giving **23'** as a white solid (233.7 mg, 84% yield); mp 132.0-133.0 ℃; $[a]_D^{24}$ -47.2 (*c* 1.0, $CHCl_3$, 93% ee); IR (KBr): 3313, 2869, 1628, 1545, 852 cm^{-1}; ^1H NMR (400 MHz, $CDCl_3$) δ 8.99 (s, 1H), 8.00 (dd, *J* = 15.1, 8.6 Hz, 3H), 7.86 (d, *J* = 8.5 Hz, 2H), 7.78 (s, 1H), 7.66 (s, 1H), 7.58 – 7.43 (m, 3H), 7.35 – 7.25 (m, 3H), 7.17 (dt, *J* = 15.4, 7.9 Hz, 4H), 7.04 (s, 1H); ^{13}C NMR (100 MHz, $CDCl_3$) δ 146.1, 135.6, 134.6, 134.4, 132.5, 130.9, 130.0, 128.5, 128.5, 128.1, 128.0, 127.8, 127.4, 127.0, 125.4, 125.3, 123.5, 123.1, 122.9, 122.6, 120.6, 119.9, 119.7, 119.2, 116.6, 111.5, 108.6, 108.1, 105.5, 101.7; HRMS (ESI) calcd for $C_{31}H_{19}F_3N_2O_3SNa$ *m/z* $[M + Na]^+$: 579.0961; found: 579.0957; HPLC (Daicel Chiralpak IA, *i*-PrOH/hexane = 20/80, flow rate 0.8 mL/min, λ = 260 nm): t_1 (minor) = 16.9 min, t_2 (major) = 20.7 min.

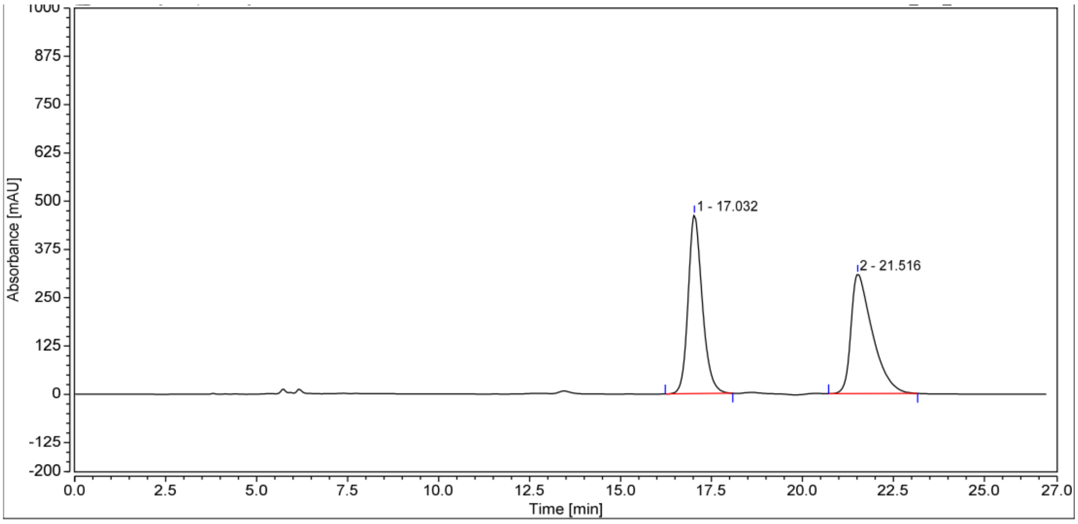

Integration Results

No.	Peak Name	Retention Time min	Area mAU*min	Height mAU	Relative Area %	Relative Height %	Amount n.a.
1		17.032	209.296	463.041	49.74	59.94	n.a.
2		21.516	211.499	309.453	50.26	40.06	n.a.
**Total:**			**420.795**	**772.494**	**100.00**	**100.00**	

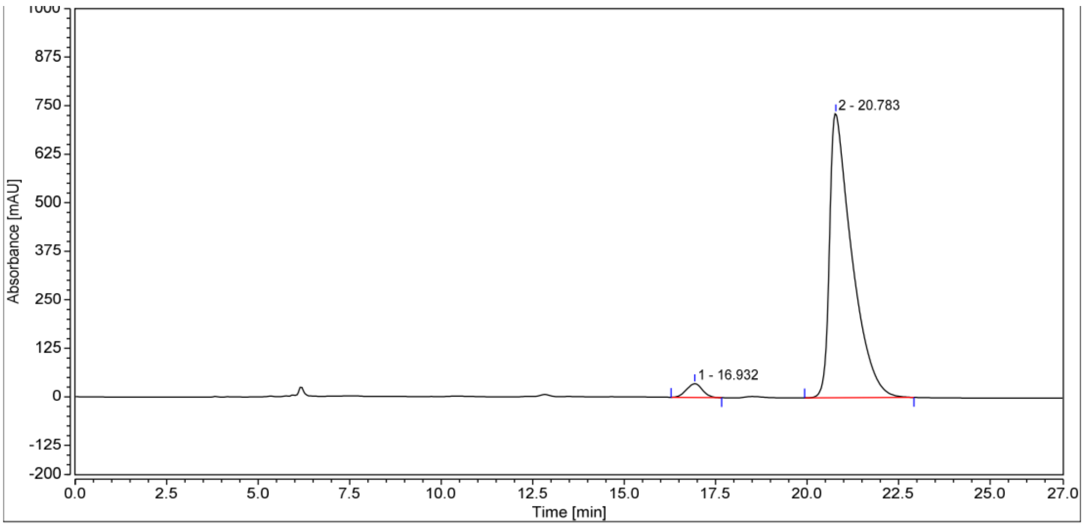

Integration Results							
No.	Peak Name	Retention Time min	Area mAU*min	Height mAU	Relative Area %	Relative Height %	Amount n.a.
1		16.932	18.031	35.555	3.37	4.63	n.a.
2		20.783	517.756	732.128	96.63	95.37	n.a.
**Total:**			**535.787**	**767.682**	**100.00**	**100.00**	

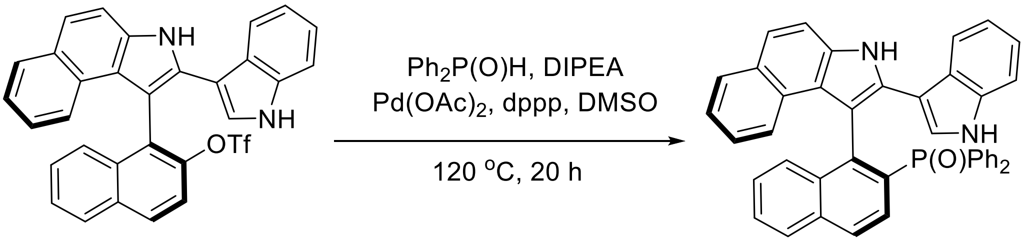

23', 93% ee

24, 26%, 98% ee

(*S*)-(1-(2-(1H-Indol-3-yl)-3H-benzo[e]indol-1-yl)naphthalen-2-yl)diphenylphosphine oxide (24)

A solution of $Pd(OAc)_2$ (8.1mg, 0.036 mmol, 0.2 eq.) and dppp (15.0 mg, 0.036 mmol, 0.2 eq.) in dry DMSO (3.0 mL) was stirred at room temperature for 0.5 h at under N_2. Subsequently, **23'** (100 mg, 0.18 mmol, 1.0 eq.), diphenylphosphine oxide (91 mg, 0.45 mmol, 2.5 eq.) and DIPEA (0.16 mL, 0.9 mmol, 5.0 eq.) were added. The reaction was stirred at 120 ºC for 20 h. The reaction was quenched with H_2O (10 mL). The solution was extracted with CH_2Cl_2 (3 ×10 mL), washed with H_2O (3 ×10 mL) and brine (10.0 mL) and dried over anhydrous Na_2SO_4. The solvent was evaporated under reduced pressure. The crude product was purified by flash column chromatography (30% EtOAc in hexane) gave **24** (28 mg, 26% yield, 99% ee), mp 156.0-157.0 ℃; $[a]_D^{24}$ -22.8 (*c* 1.0, $CHCl_3$, 99% ee); IR (KBr): 3343, 3056, 2962, 1865, 1352 cm^{-1}; ^1H NMR (400 MHz, $CDCl_3$) δ 9.13 (s, 1H), 8.73 (s, 1H), 8.06 – 7.90 (m,

3H), 7.80 – 7.60 (m, 4H), 7.53 (dd, J = 15.8, 7.9 Hz, 1H), 7.48 – 7.38 (m, 4H), 7.21 (t, J = 7.6 Hz, 3H), 7.09 (dt, J = 11.2, 7.5 Hz, 3H), 6.96 (ddd, J = 23.2, 11.4, 7.9 Hz, 6H), 6.74 (t, J = 7.2 Hz, 1H), 6.40 (td, J = 7.7, 2.8 Hz, 2H); ^{13}C NMR (100 MHz, $CDCl_3$) δ 152.3, 140.9, 135.7, 135.0, 134.1, 133.2, 131.4, 129.9, 129.8, 129.1, 128.7, 128.6, 128.2, 128.0, 127.8, 127.0, 126.9, 126.7, 126.1, 126.1, 125.9, 125.8, 125.4, 125.3, 125.1, 123.8, 123.6, 123.3, 122.7, 122.6, 122.4, 121.3, 121.0, 119.2, 118.7, 117.4, 117.3, 115.5, 111.8, 111.4, 105.0, 104.7; HRMS (ESI) calcd for $C_{42}H_{29}N_2OPNa$ *m/z* [M + Na]$^+$: 631.1910; found: 631.1913; HPLC (Daicel Chiralpak IA, *i*-PrOH/hexane = 40/60, flow rate 0.8 mL/min, λ = 260 nm): t_1 (major) = 15.8 min, t_2 (minor) = 25.4 min.

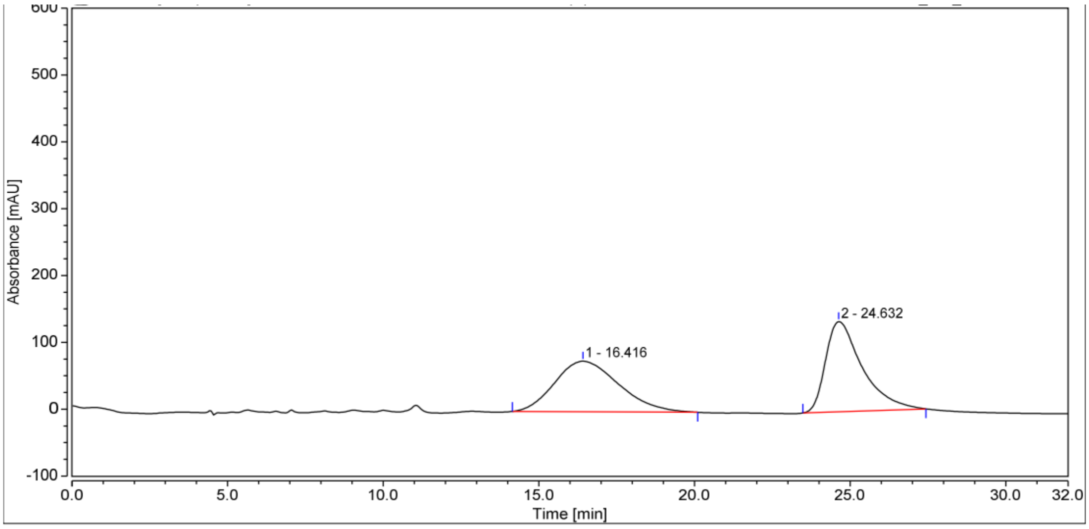

Integration Results							
No.	Peak Name	Retention Time min	Area mAU*min	Height mAU	Relative Area %	Relative Height %	Amount n.a.
1		16.416	180.962	75.632	49.47	35.92	n.a.
2		24.632	184.808	134.932	50.53	64.08	n.a.
Total:			365.770	210.565	100.00	100.00	

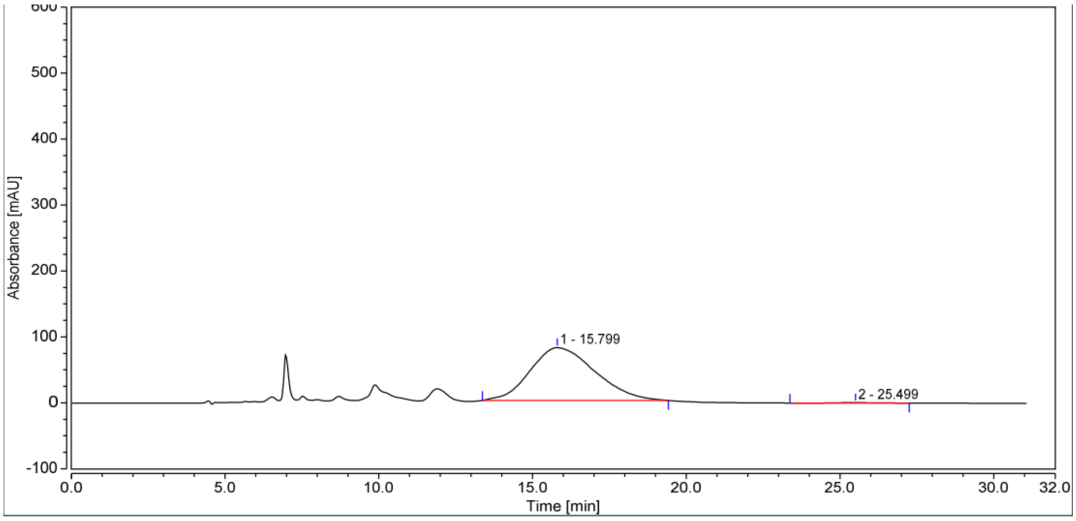

Integration Results							
No.	Peak Name	Retention Time min	Area mAU*min	Height mAU	Relative Area %	Relative Height %	Amount n.a.
1		15.799	196.186	80.022	99.58	99.30	n.a.
2		25.499	0.833	0.563	0.42	0.70	n.a.
Total:			197.019	80.585	100.00	100.00	

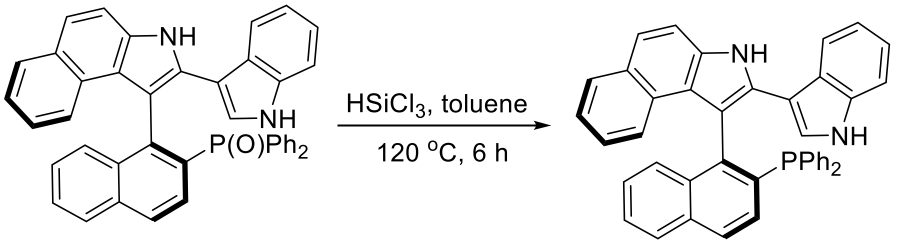

24, 98% ee **25**, 95%, 98% ee

(*S*)-1-(2-(Diphenylphosphaneyl)naphthalen-1-yl)-2-(1H-indol-3-yl)-3H-benzo[e]indole (25)

A dried pressure tube charged with **24** (28.0 mg, 0.046 mmol, 1.0 eq.) and $HSiCl_3$ (18.8 mg, 0.13 mmol, 3.0 eq.) in 1 mL toluene was stirred at 120 °C for 6 h under N_2. After removal of the solvent under reduced pressure, the residue was purified by column chromatography on silica gel (5% EtOAc in hexane) gave **25** (25.9 mg, 95% yield); mp 162.0-163.0 ℃; $[\alpha]_D^{24}$ -46.8 (*c* 1.0, $CHCl_3$, 98% ee); IR (KBr): 3243, 2866, 1962, 1465, 752 cm^{-1}; ^1H NMR (400 MHz, $CDCl_3$) δ 8.94 (s, 1H), 7.96 – 7.80 (m, 4H), 7.73 – 7.61 (m, 4H), 7.53 – 7.47 (m, 1H), 7.37 – 7.29 (m, 3H), 7.24 – 7.17 (m, 2H), 7.14 (dd, *J* = 11.4, 4.5 Hz, 2H), 7.07 – 7.02 (m, 3H), 7.01 – 6.95 (m, 1H), 6.95 – 6.89 (m, 2H), 6.80 – 6.67 (m, 5H), 6.14 (d, *J* = 2.6 Hz, 1H); ^{13}C NMR (150 MHz, $CDCl_3$) δ 142.8, 142.6, 139.2, 139.1, 135.7, 134.7, 133.7, 133.3, 133.2, 133.0, 132.3, 130.9, 130.0, 128.7, 128.5, 128.1, 128.0, 127.9, 127.6, 127.5, 127.4, 127.4, 127.3,

126.9, 125.6, 125.2, 123.2, 123.2, 123.0, 122.9, 122.3, 120.6, 120.4, 119.4, 118.8, 113.1, 113.0, 112.6, 112.2, 111.8, 111.3, 108.8; HRMS (ESI) calcd for $C_{42}H_{29}N_2PNa$ *m/z* $[M + Na]^+$: 615.1961; found: 615.1966; HPLC (Daicel Chiralpak IC, *i*-PrOH/hexane = 20/80, flow rate 0.8 mL/min, λ = 260 nm): t_1 (major) = 4.6 min, t_2 (minor) = 14.3 min.

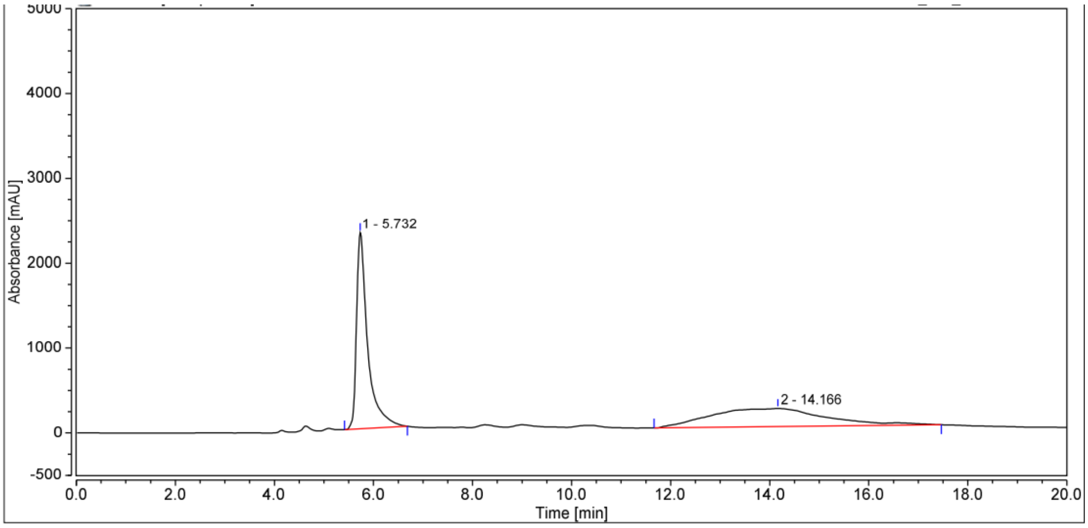

Integration Results							
No.	Peak Name	Retention Time min	Area mAU*min	Height mAU	Relative Area %	Relative Height %	Amount n.a.
1		5.732	603.561	2313.247	50.96	91.58	n.a.
2		14.166	580.793	212.603	49.04	8.42	n.a.
**Total:**			**1184.354**	**2525.850**	**100.00**	**100.00**	

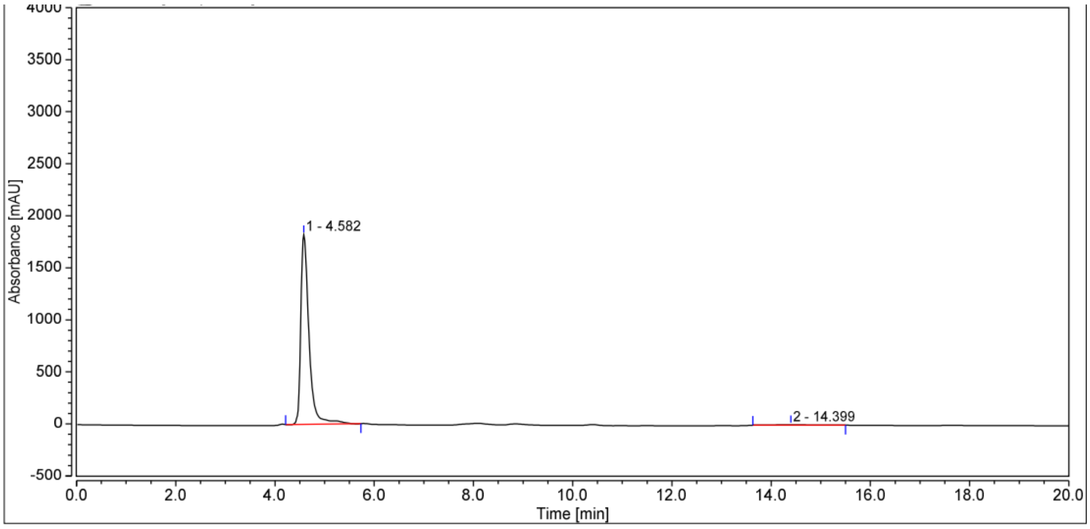

Integration Results							
No.	Peak Name	Retention Time min	Area mAU*min	Height mAU	Relative Area %	Relative Height %	Amount n.a.
1		4.582	349.695	1822.195	99.09	99.85	n.a.
2		14.399	3.198	2.675	0.91	0.15	n.a.
**Total:**			**352.893**	**1824.869**	**100.00**	**100.00**	

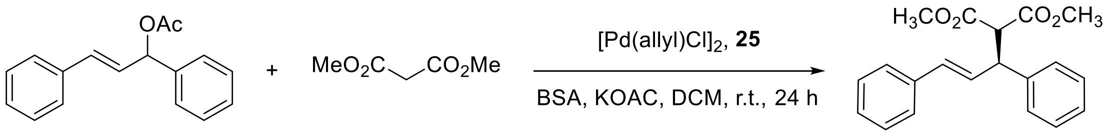

26 **27** **28**, 99%, 39% ee

(*S*,*E*)-Dimethyl-2-(1,3-diphenylallyl)malonate (28)[10]

In a Schlenk tube, a solution of 1,3-diphenylprop-2-en-1-yl acetate **26** (63 mg, 0.25 mmol, 1.0 eq.), [Pd(allyl)Cl]$_2$ (1.8 mg, 0.005 mmo, 0.02 eq.), and **25** (5.9 mg, 0.01 mmol, 0.04 eq.) in CH_2Cl_2 (2 mL) was stirred at room temperature for 40 min. Dimethyl malonate **27** (99 mg, 0.75mmol, 2.5 eq.), KOAc (2.0 mg, 0.02 mmol, 0.08 eq.), and BSA (152 mg, 0.75 mmol, 3.0 eq.) were then added. The reaction mixture was stirred for 24 h and then quenched with saturated aqueous NH_4Cl and extracted with CH_2Cl_2 (3 × 10 mL). The organic phase was washed with saturated aqueous $NaHCO_3$ and brine, dried over Na_2SO_4, and concentrated under a reduced pressure. The residue was purified by flash chromatography on silica gel with hexane–EtOAc (15:1) as eluent to produce **28** (80 mg, 99% yield, 39% ee); ^{1}H NMR (400 MHz, $CDCl_3$) δ 7.31 – 7.03 (m, 10H), 6.39 (d, *J* = 15.8 Hz, 1H), 6.24 (dd, *J* = 15.7, 8.6 Hz, 1H), 4.19 (dd, *J* = 10.7, 8.8 Hz, 1H), 3.88 (d, *J* = 10.9 Hz, 1H), 3.59 (s, 3H), 3.40 (s, 3H); ^{13}C NMR (100 MHz, $CDCl_3$) δ 168.1, 167.6, 140.0, 136.7, 131.7, 129.0, 128.6, 128.4, 127.7, 127.5, 127.0, 126.3, 57.5, 52.5, 52.3, 49.1; HRMS (ESI) calcd for $C_{20}H_{20}O_4Na$ *m/z* [M + Na]$^+$: 347.1254, found: 347.1249; HPLC (Daicel Chiralpak IA, *i*-PrOH/hexane =20/90, flow rate 0.8 mL/min, λ = 230 nm): t_1 (minor) = 7.8 min, t_2 (major) = 9.3 min.

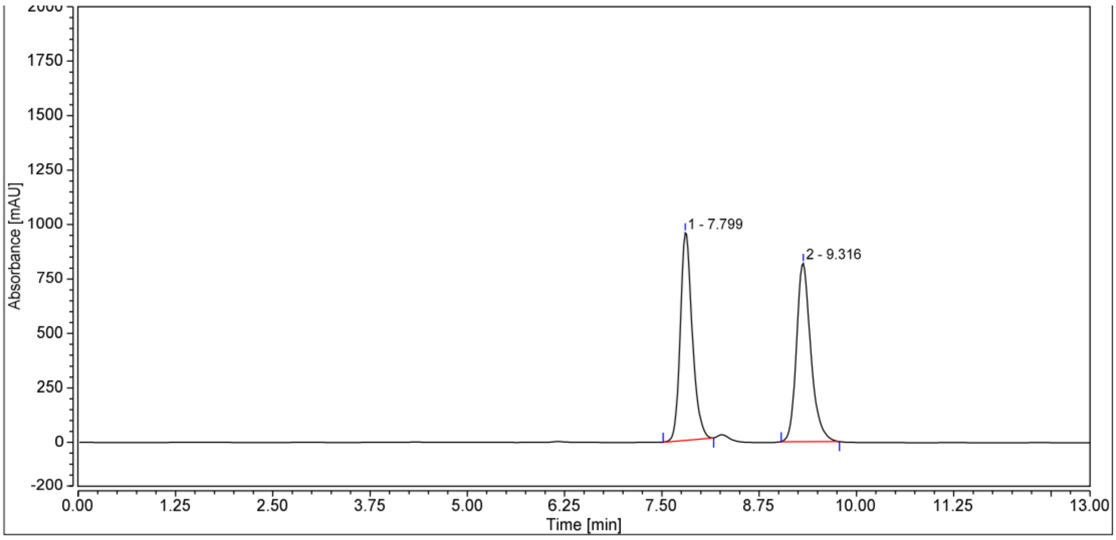

Integration Results

No.	Peak Name	Retention Time<br>min	Area<br>mAU*min	Height<br>mAU	Relative Area<br>%	Relative Height<br>%	Amount<br>n.a.
1		7.799	170.294	952.926	49.24	53.80	n.a.
2		9.316	175.529	818.219	50.76	46.20	n.a.
**Total:**			**345.823**	**1771.146**	**100.00**	**100.00**	

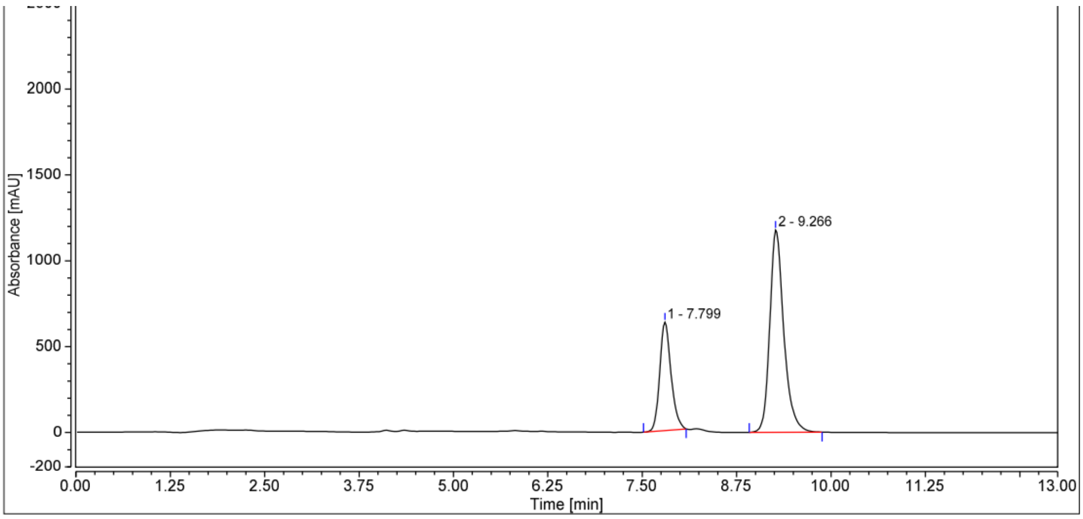

Integration Results

No.	Peak Name	Retention Time<br>min	Area<br>mAU*min	Height<br>mAU	Relative Area<br>%	Relative Height<br>%	Amount<br>n.a.
1		7.799	110.004	631.699	30.19	34.92	n.a.
2		9.266	254.413	1177.243	69.81	65.08	n.a.
**Total:**			**364.416**	**1808.942**	**100.00**	**100.00**	

Enantiomerisation barrier determination for 15a and 15af

The enantiomerisation barrier, corresponding to barrier to rotation for the following atropisomers, was obtained by kinetic of racemisation of an enantiomer. The slope of the first-order kinetic line gives the racemisation constant ($k_{racemisation} = 2 \times k_{enantiomerisation}$). Eyring equation gives the enantiomerisation barrier ($\Delta G^{\neq}_{enantiomerisation}$) from enantiomerisation constant ($k_{enantiomerisation}$), R = 8.31451 $J.K^{-1}$ mol^{-1}, h = 6.62608 $\times 10^{-34}$ J.s and k_B =1.38066 $\times 10^{-23}$ $J.K^{-1}$.

About 5 mg of enantio-enriched **15a** was heated in 3 mL of *o*-dibromobenzene. Samples of 5 μL of this solution were injected on Daicel Chiralpak IA (*i*-PrOH/hexane = 20/80, flow rate 0.8 mL/min, λ = 230 nm) to monitor the percentage decrease of the second eluted enantiomer over time.

Solvent : *o*-dibromobenzene

Temperature = 200 ℃

Time (h)	% second eluted enantiomer (%t)	ln ((%t-50)/(%t_0-50))
0	97	0
6	95.28	-0.0373
12	93.02	-0.0885
18	91.68	-0.1201
24	90.26	-0.1548
30	89.16	-0.1825
36	87.76	-0.2189
42	86.08	-0.2644
48	85.16	-0.2902

$K_{racemisation} = 1.6667 \times 10^{-6}\ s^{-1}$

$k_{enantiomerisation} = 8.3333 \times 10^{-7}\ s^{-1}$

$\Delta G^{\neq}$ enantiomerisation = 172.7 $kJ.mol^{-1}$

Half-life time $t_{1/2}$ (200 ^{o}C)= 4.8 days

$t_{1/2}$ (25 ^{o}C) = 3.3 $\times 10^{9}$ years

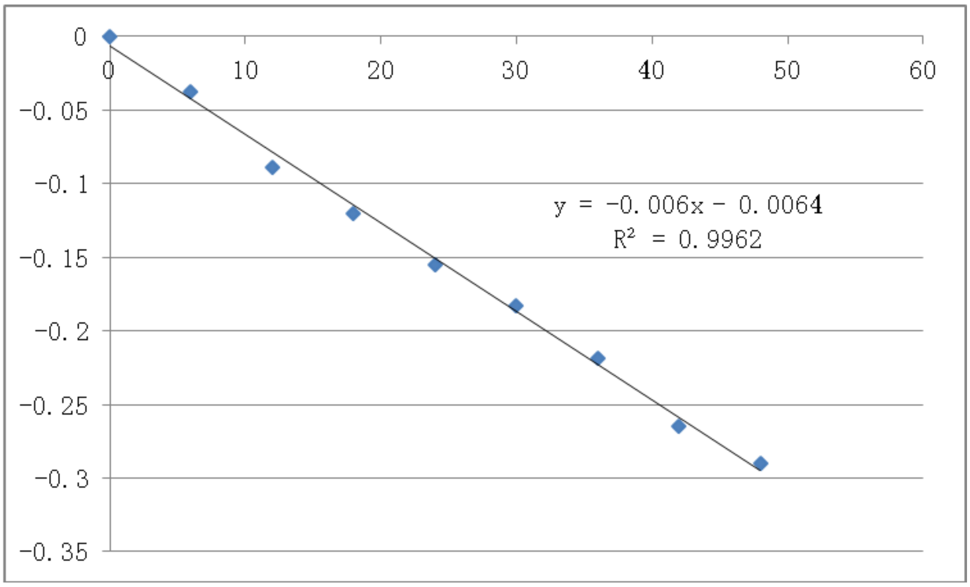

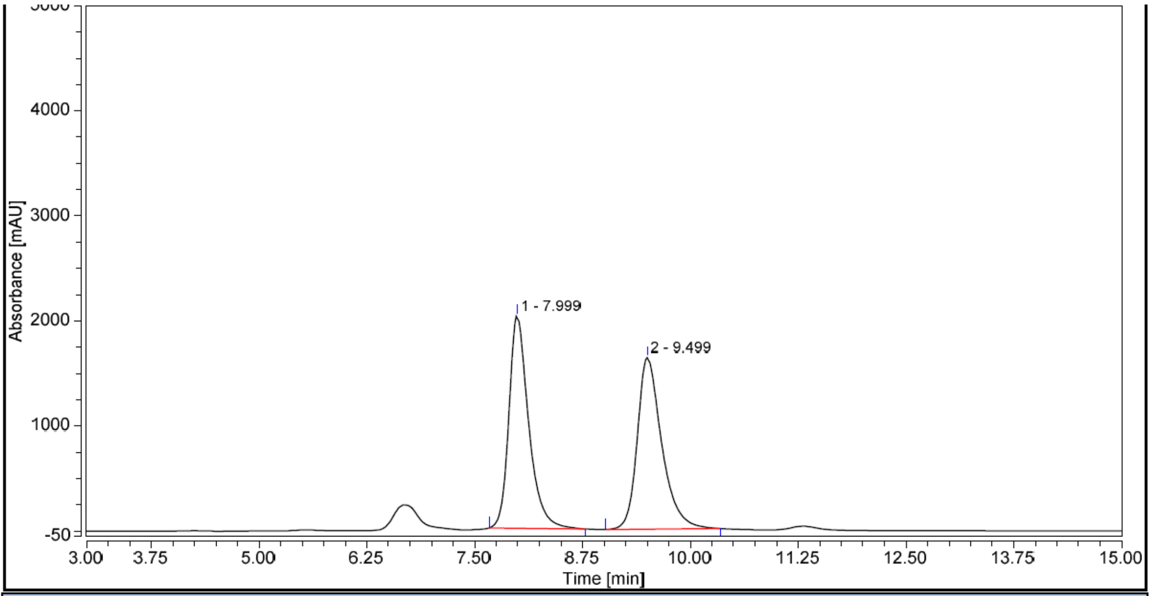

Integration Results

No.	Peak Name	Retention Time min	Area mAU*min	Height mAU	Relative Area %	Relative Height %	Amount n.a.
1		7.999	537.748	2023.573	50.02	55.33	n.a.
2		9.499	537.307	1633.690	49.98	44.67	n.a.
**Total:**			**1075.055**	**3657.262**	**100.00**	**100.00**	

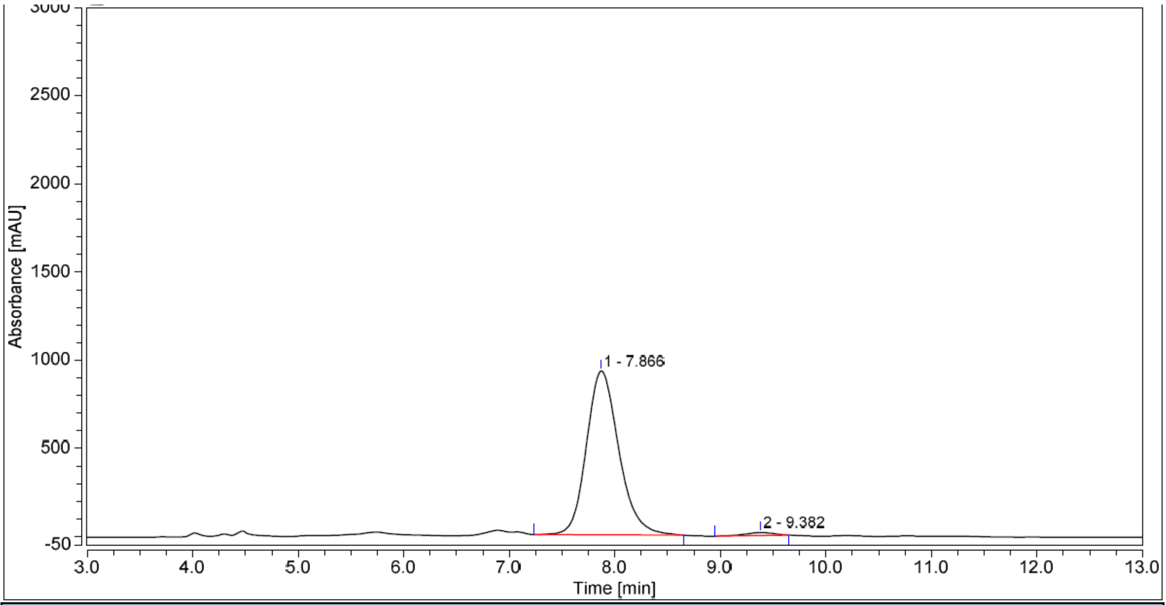

Integration Results

No.	Peak Name	Retention Time min	Area mAU*min	Height mAU	Relative Area %	Relative Height %	Amount n.a.
1		7.866	331.173	929.544	98.50	98.29	n.a.
2		9.382	5.027	16.181	1.50	1.71	n.a.
**Total:**			**336.201**	**945.725**	**100.00**	**100.00**	

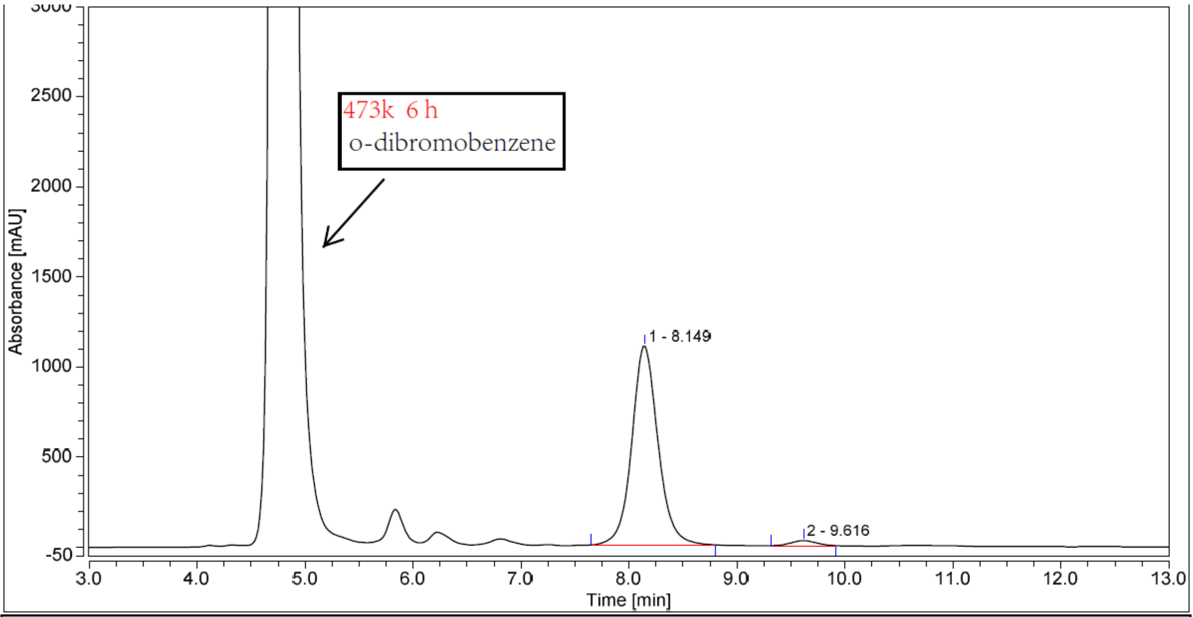

Integration Results

No.	Peak Name	Retention Time min	Area mAU*min	Height mAU	Relative Area %	Relative Height %	Amount n.a.
1		8.149	310.387	1101.733	97.64	97.53	n.a.
2		9.616	7.490	27.936	2.36	2.47	n.a.
**Total:**			**317.877**	**1129.669**	**100.00**	**100.00**	

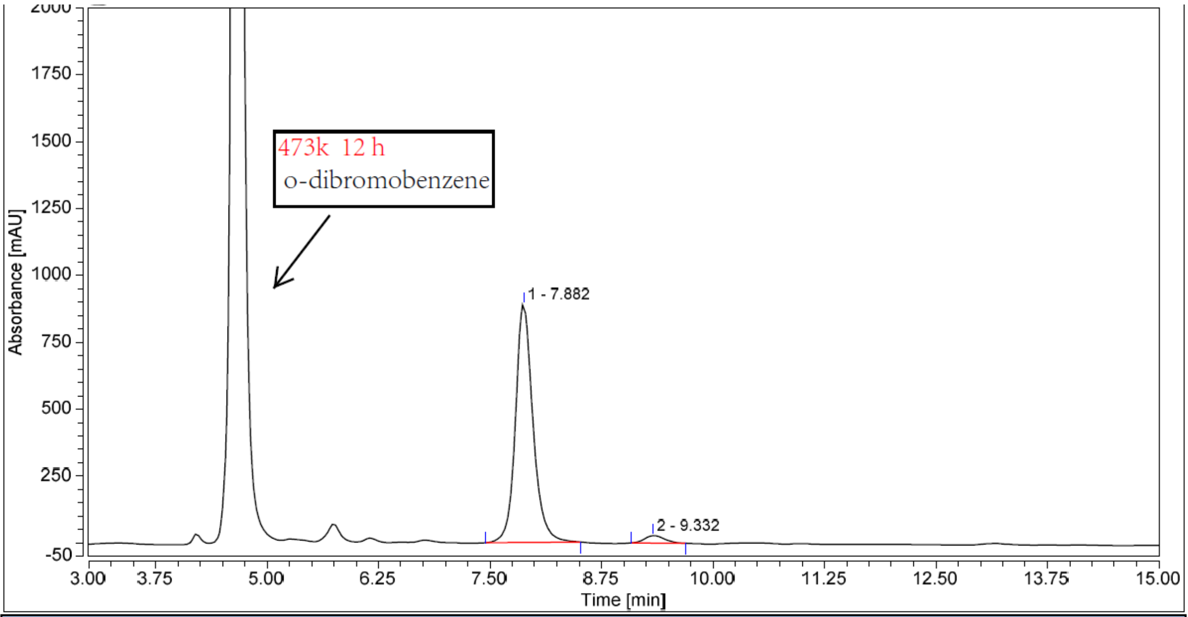

Integration Results

No.	Peak Name	Retention Time min	Area mAU*min	Height mAU	Relative Area %	Relative Height %	Amount n.a.
1		7.882	203.913	890.201	96.51	96.93	n.a.
2		9.332	7.383	28.227	3.49	3.07	n.a.
**Total:**			**211.296**	**918.428**	**100.00**	**100.00**	

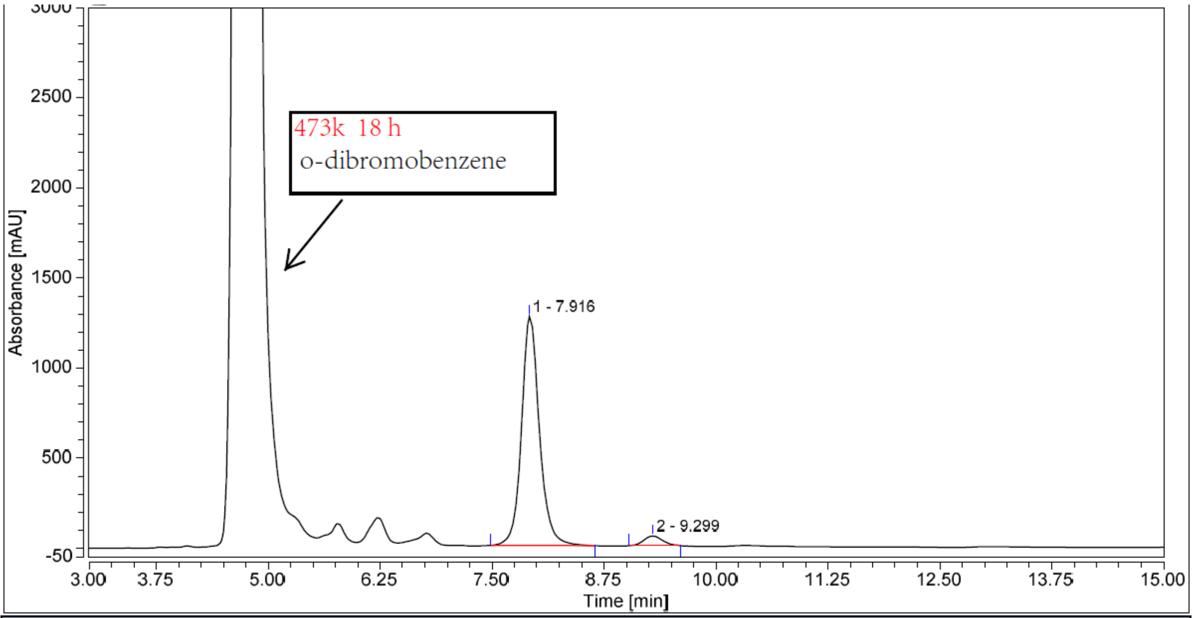

Integration Results

No.	Peak Name	Retention Time min	Area mAU*min	Height mAU	Relative Area %	Relative Height %	Amount n.a.
1		7.916	295.386	1271.493	95.84	95.95	n.a.
2		9.299	12.809	53.605	4.16	4.05	n.a.
**Total:**			**308.194**	**1325.098**	**100.00**	**100.00**	

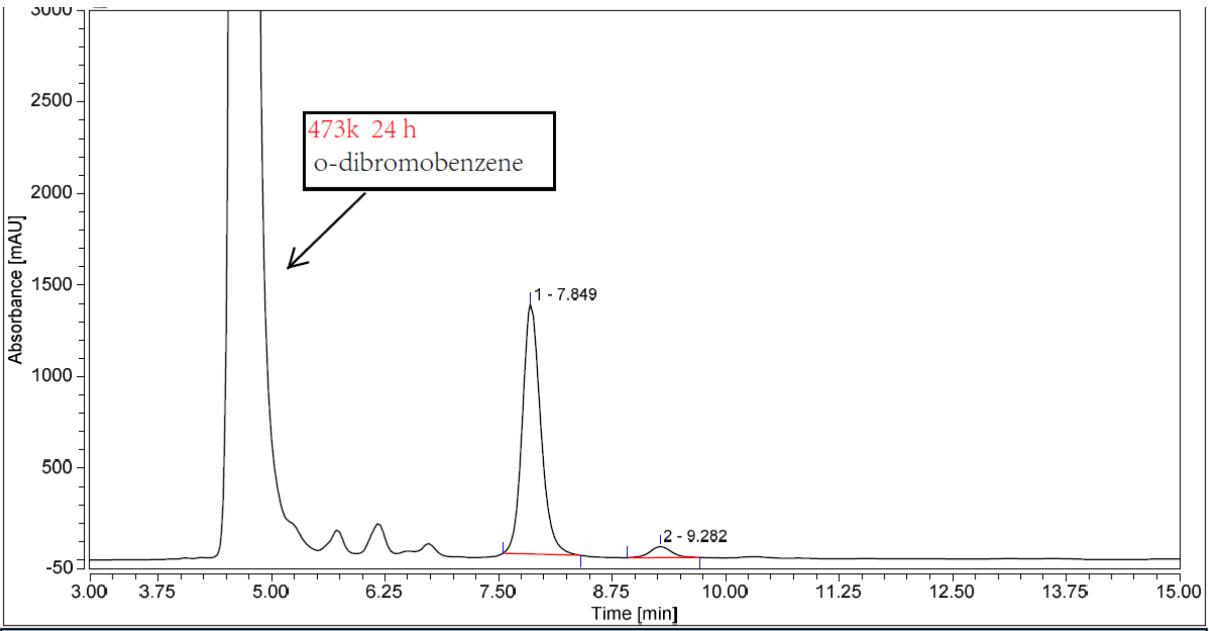

Integration Results

No.	Peak Name	Retention Time min	Area mAU*min	Height mAU	Relative Area %	Relative Height %	Amount n.a.
1		7.849	320.668	1362.696	95.13	95.76	n.a.
2		9.282	16.402	60.355	4.87	4.24	n.a.
**Total:**			**337.070**	**1423.052**	**100.00**	**100.00**	

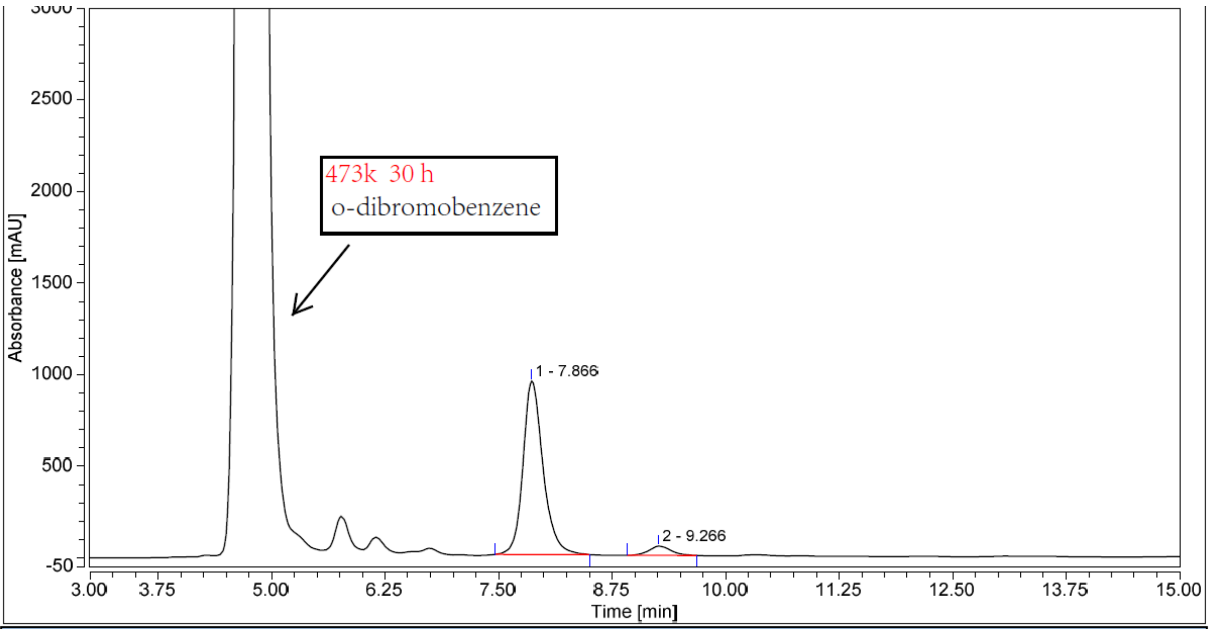

Integration Results

No.	Peak Name	Retention Time min	Area mAU*min	Height mAU	Relative Area %	Relative Height %	Amount n.a.
1		7.866	242.471	945.974	94.58	95.00	n.a.
2		9.266	13.907	49.782	5.42	5.00	n.a.
**Total:**			**256.378**	**995.756**	**100.00**	**100.00**	

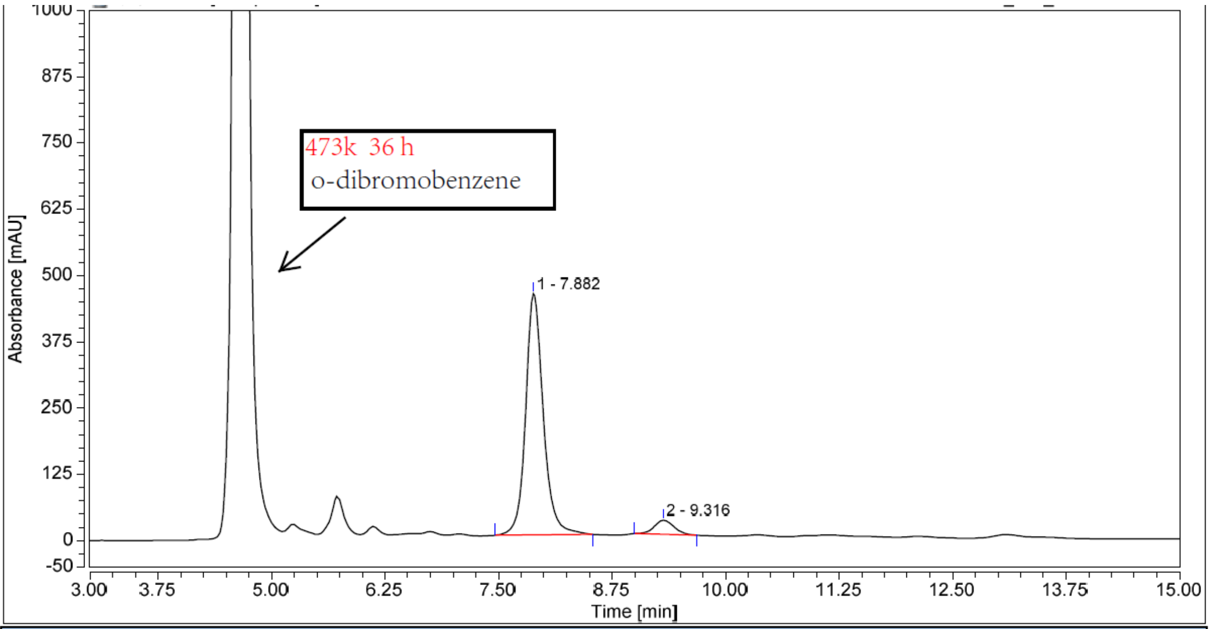

Integration Results

No.	Peak Name	Retention Time min	Area mAU*min	Height mAU	Relative Area %	Relative Height %	Amount n.a.
1		7.882	102.749	454.717	93.88	94.49	n.a.
2		9.316	6.701	26.519	6.12	5.51	n.a.
**Total:**			**109.450**	**481.236**	**100.00**	**100.00**	

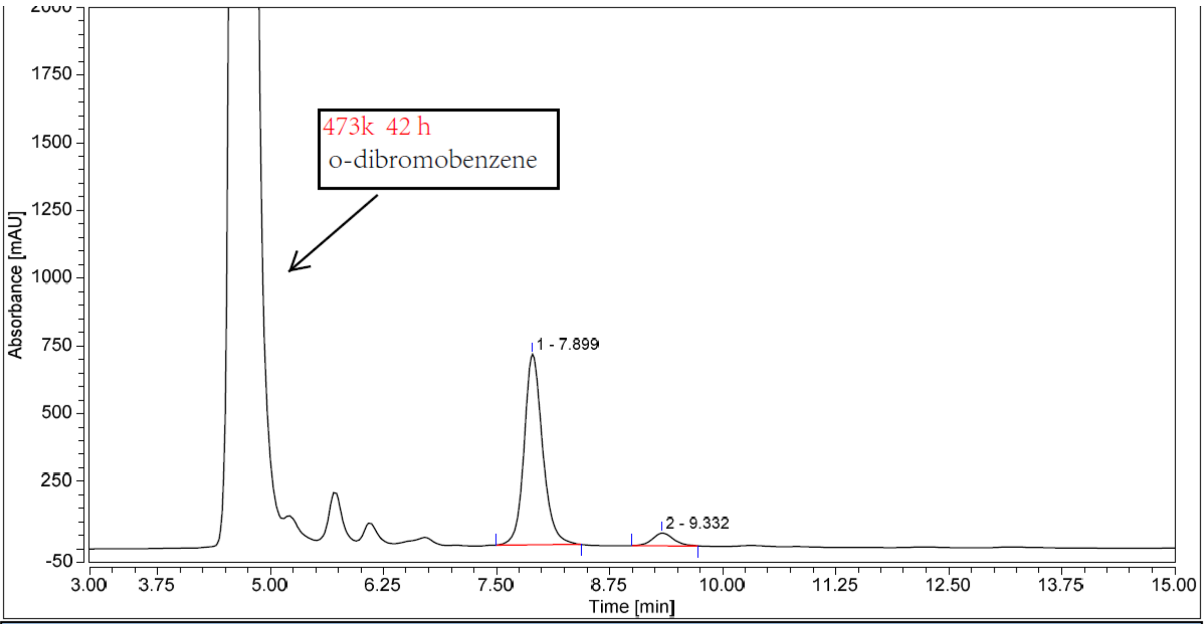

Integration Results

No.	Peak Name	Retention Time min	Area mAU*min	Height mAU	Relative Area %	Relative Height %	Amount n.a.
1		7.899	166.542	703.816	93.04	93.80	n.a.
2		9.332	12.464	46.523	6.96	6.20	n.a.
**Total:**			**179.006**	**750.339**	**100.00**	**100.00**	

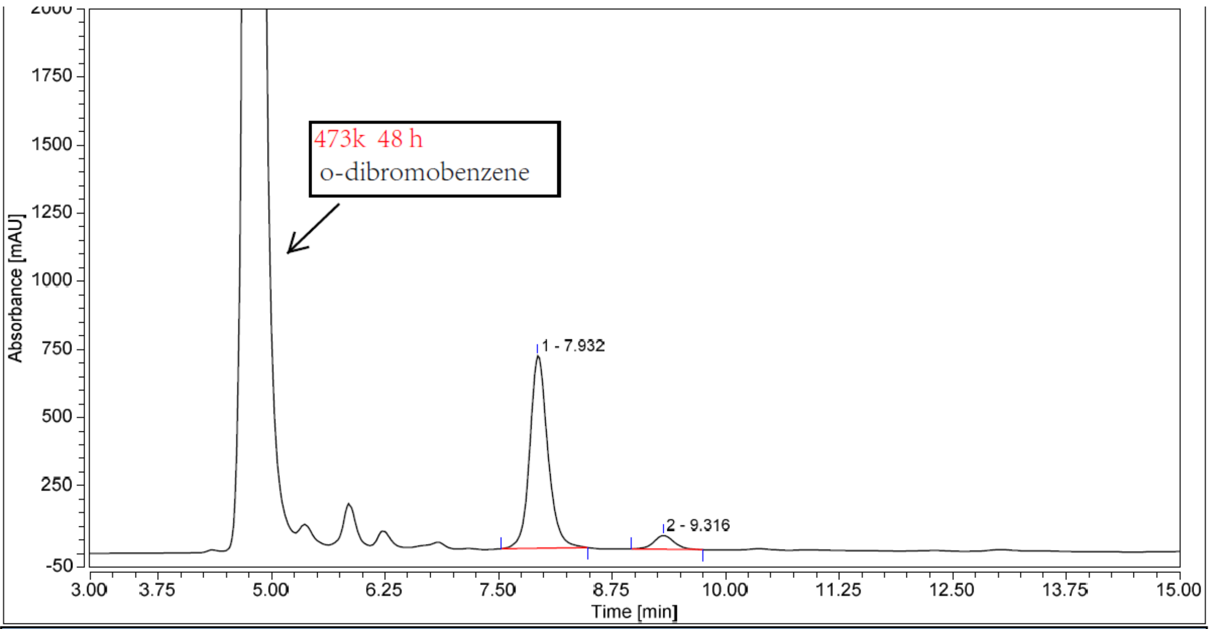

Integration Results							
No.	Peak Name	Retention Time min	Area mAU*min	Height mAU	Relative Area %	Relative Height %	Amount n.a.
1		7.932	163.042	706.369	92.58	93.35	n.a.
2		9.316	13.062	50.286	7.42	6.65	n.a.
**Total:**			**176.104**	**756.655**	**100.00**	**100.00**	

About 10 mg of enantio-enriched **15af** was dissolved in 5 mL of dichloromethane. Samples of 5 μL of this solution were injected on Daicel Chiralpak IC (*i*-PrOH/hexane = 10/80, flow rate 0.8 mL/min, λ = 230 nm) to monitor the percentage decrease of the second eluted enantiomer over time.

Solvent : dichloromethane.

Temperature = 25 ℃

Time (min)	% second eluted enantiomer (%t)	ln ((%t-50)/(%t$_0$-50))
0	71.5	0
30	70.44	-0.0505
60	68.6	-0.1449
90	67.82	-0.1877
120	66.8	-0.2467
150	65.34	-0.3376
180	64.1	-0.4219
210	62.98	-0.5046

$K_{racemisation} = 4.0 \times 10^{-5}\ s^{-1}$

$k_{enantiomerisation} = 2.0 \times 10^{-5}\ s^{-1}$

$\Delta G^{\neq}$ enantiomerisation = 99.78 kJ.mol^{-1}

Half-life time $t_{1/2}$ (25 °C)= 4.8 hours

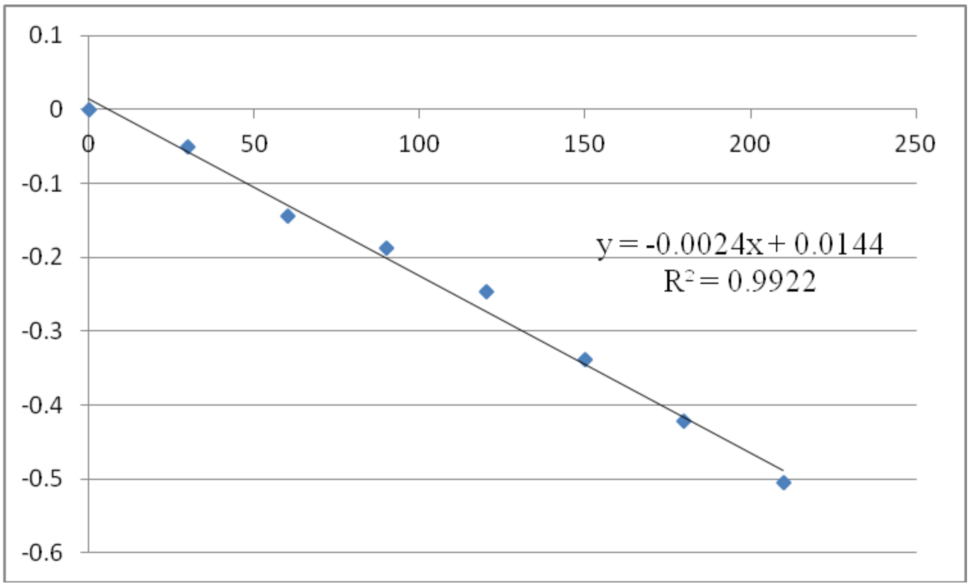

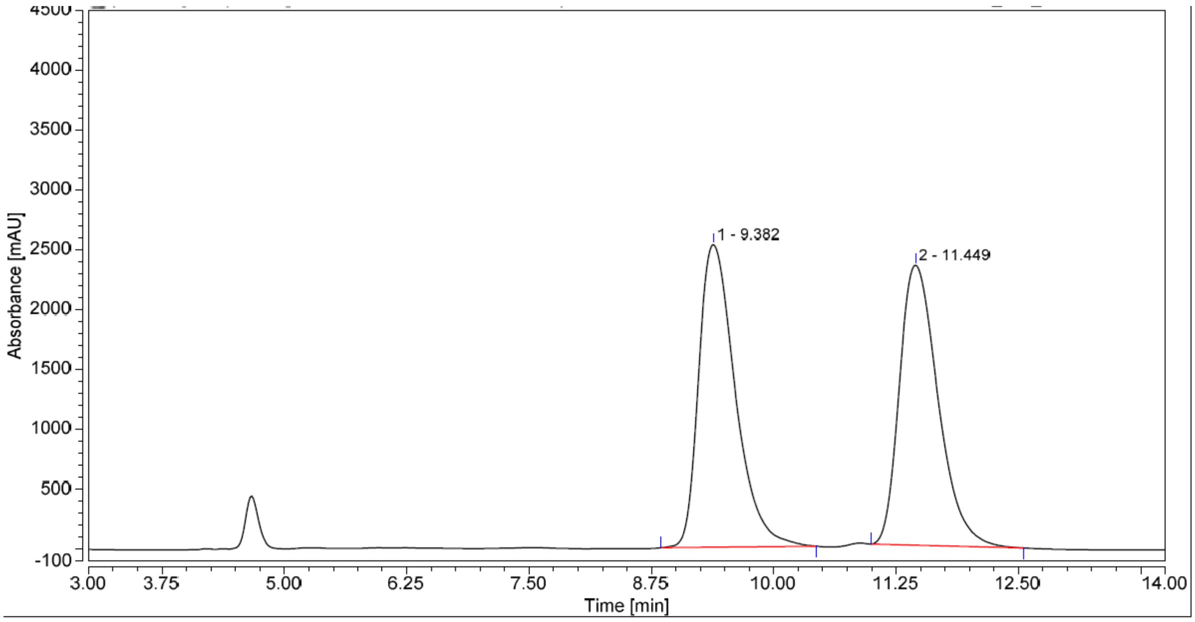

Integration Results							
No.	Peak Name	Retention Time min	Area mAU*min	Height mAU	Relative Area %	Relative Height %	Amount n.a.
1		9.382	1094.875	2527.889	50.59	51.94	n.a.
2		11.449	1069.265	2339.128	49.41	48.06	n.a.
**Total:**			**2164.140**	**4867.017**	**100.00**	**100.00**	

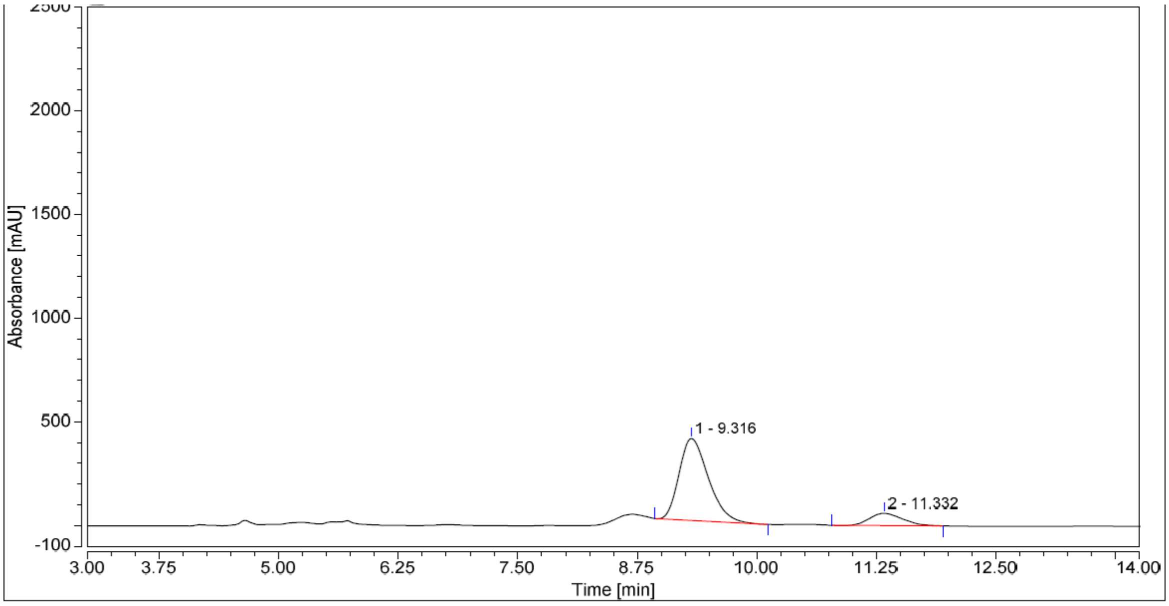

Integration Results

No.	Peak Name	Retention Time min	Area mAU*min	Height mAU	Relative Area %	Relative Height %	Amount n.a.
1		9.316	140.335	395.422	85.73	87.03	n.a.
2		11.332	23.362	58.906	14.27	12.97	n.a.
**Total:**			**163.697**	**454.328**	**100.00**	**100.00**	

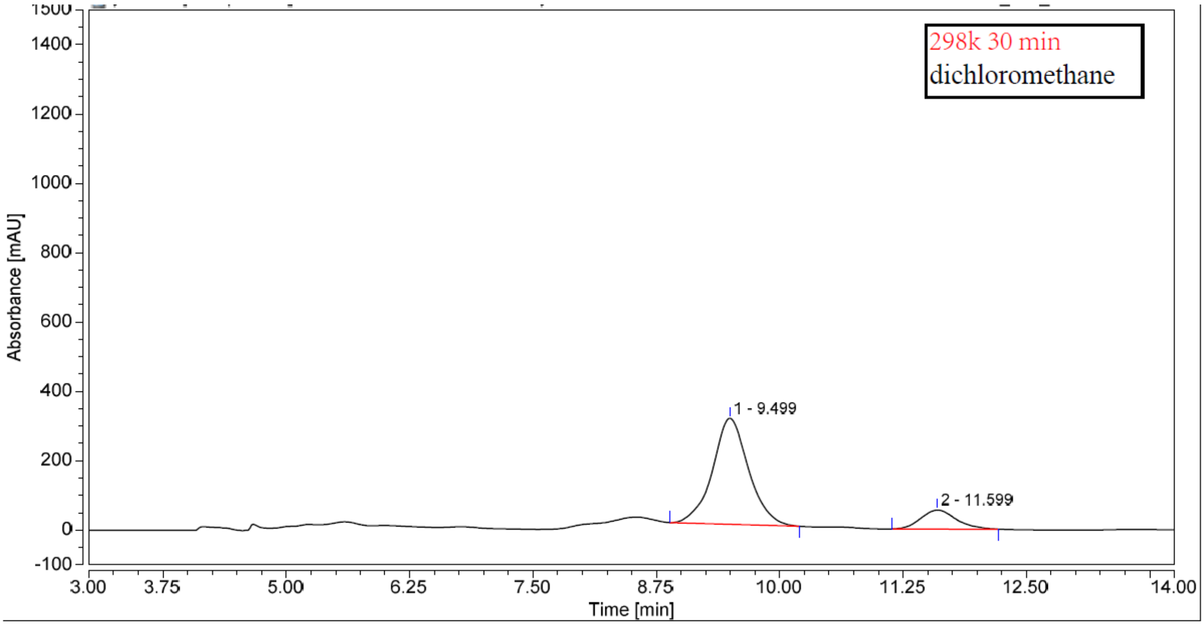

Integration Results

No.	Peak Name	Retention Time min	Area mAU*min	Height mAU	Relative Area %	Relative Height %	Amount n.a.
1		9.499	129.931	305.586	85.22	84.90	n.a.
2		11.599	22.535	54.334	14.78	15.10	n.a.
**Total:**			**152.466**	**359.920**	**100.00**	**100.00**	

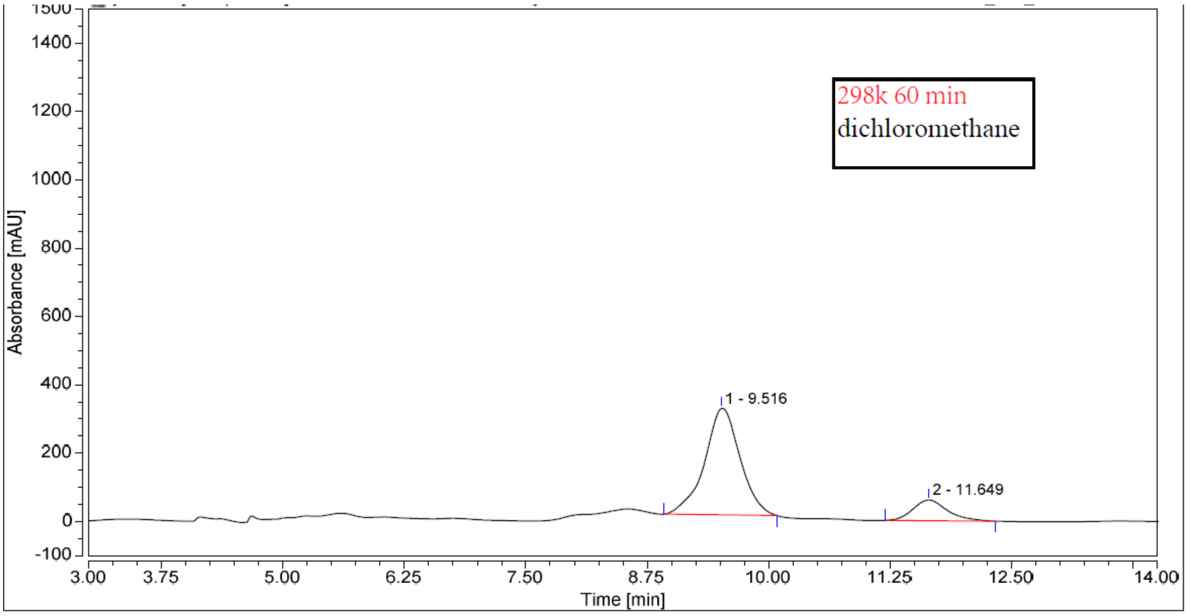

Integration Results

No.	Peak Name	Retention Time min	Area mAU*min	Height mAU	Relative Area %	Relative Height %	Amount n.a.
1		9.516	132.396	311.941	84.30	83.79	n.a.
2		11.649	24.651	60.347	15.70	16.21	n.a.
**Total:**			**157.047**	**372.289**	**100.00**	**100.00**	

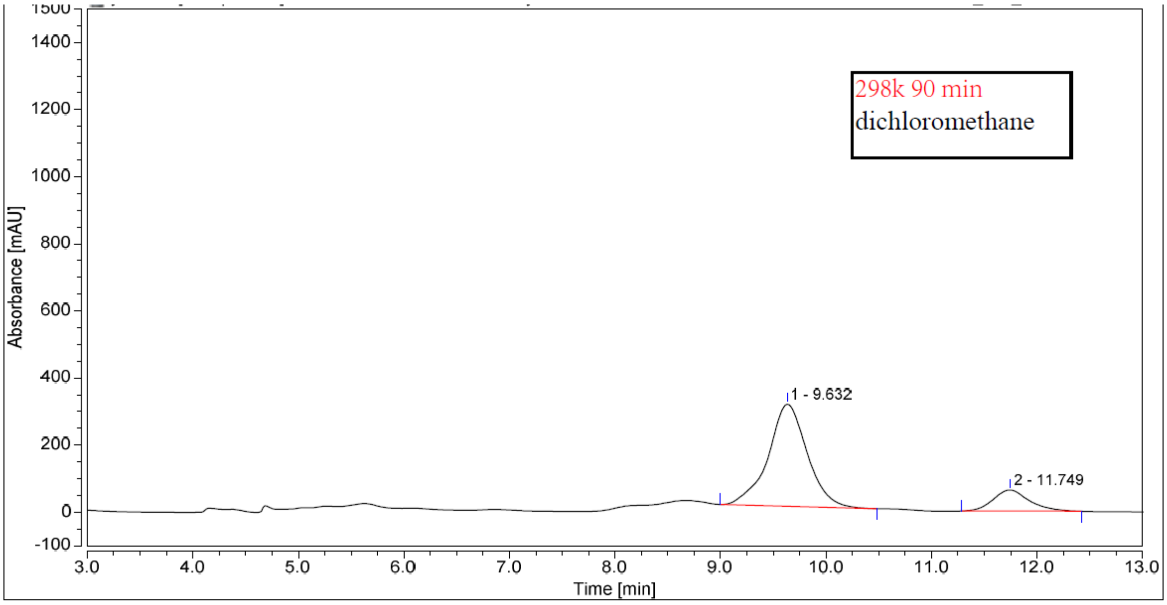

Integration Results

No.	Peak Name	Retention Time min	Area mAU*min	Height mAU	Relative Area %	Relative Height %	Amount n.a.
1		9.632	130.496	304.304	83.91	82.81	n.a.
2		11.749	25.019	63.188	16.09	17.19	n.a.
**Total:**			**155.515**	**367.492**	**100.00**	**100.00**	

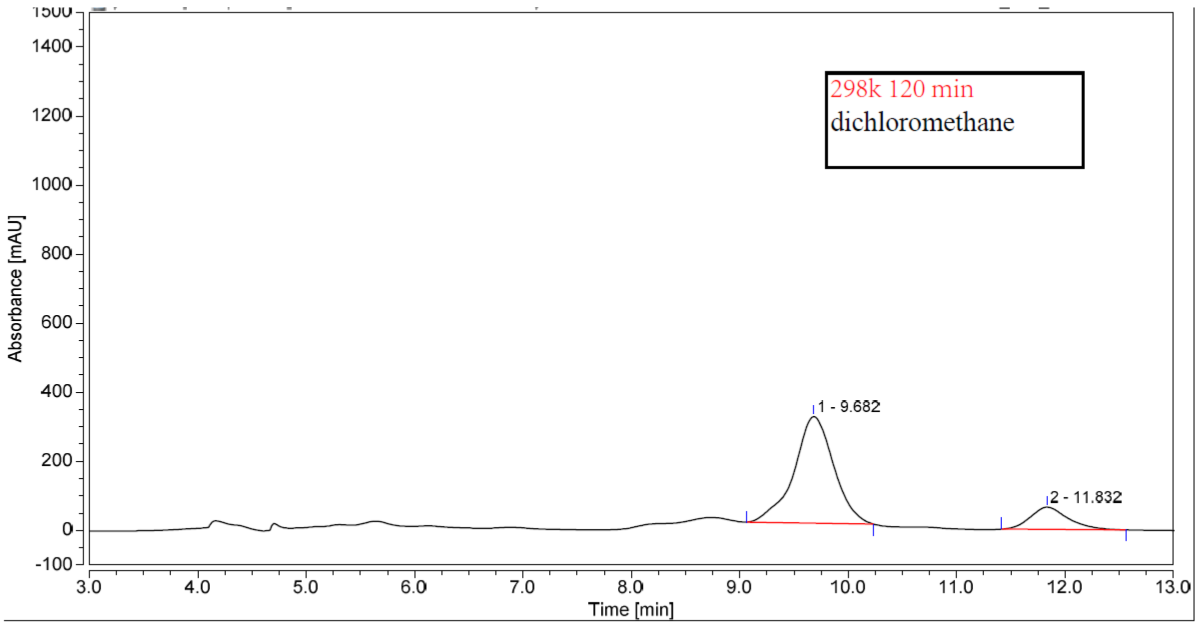

Integration Results							
No.	Peak Name	Retention Time min	Area mAU*min	Height mAU	Relative Area %	Relative Height %	Amount n.a.
1		9.682	131.082	308.678	83.40	82.73	n.a.
2		11.832	26.091	64.458	16.60	17.27	n.a.
**Total:**			**157.172**	**373.136**	**100.00**	**100.00**	

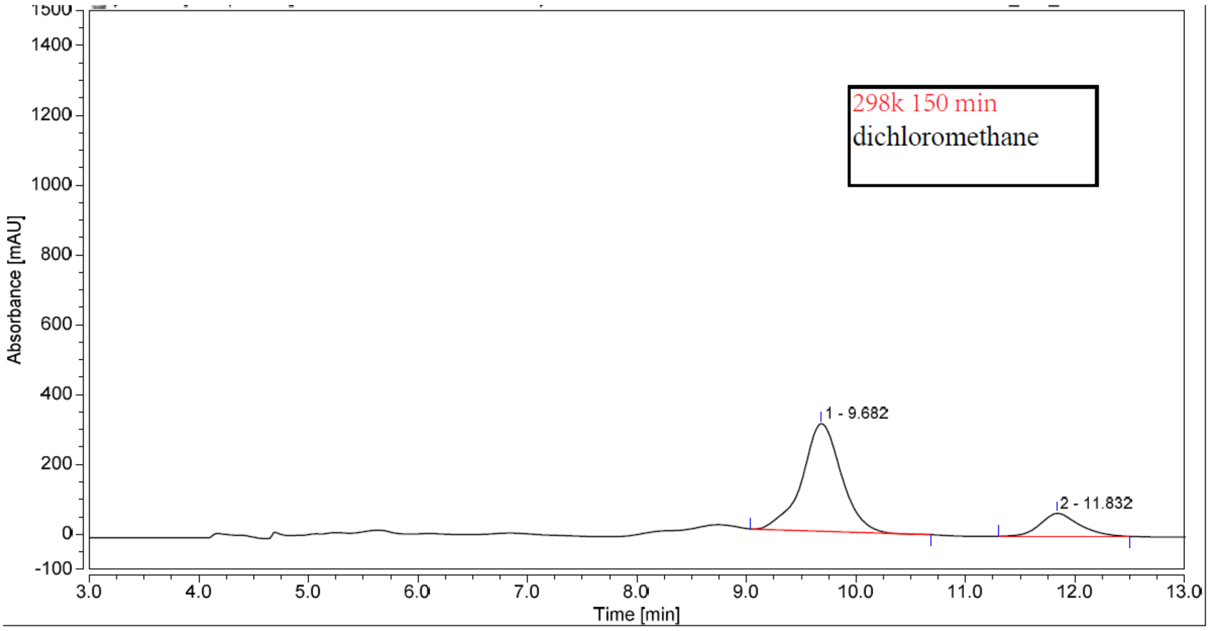

Integration Results							
No.	Peak Name	Retention Time min	Area mAU*min	Height mAU	Relative Area %	Relative Height %	Amount n.a.
1		9.682	128.121	308.264	82.67	82.51	n.a.
2		11.832	26.851	65.331	17.33	17.49	n.a.
**Total:**			**154.972**	**373.595**	**100.00**	**100.00**	

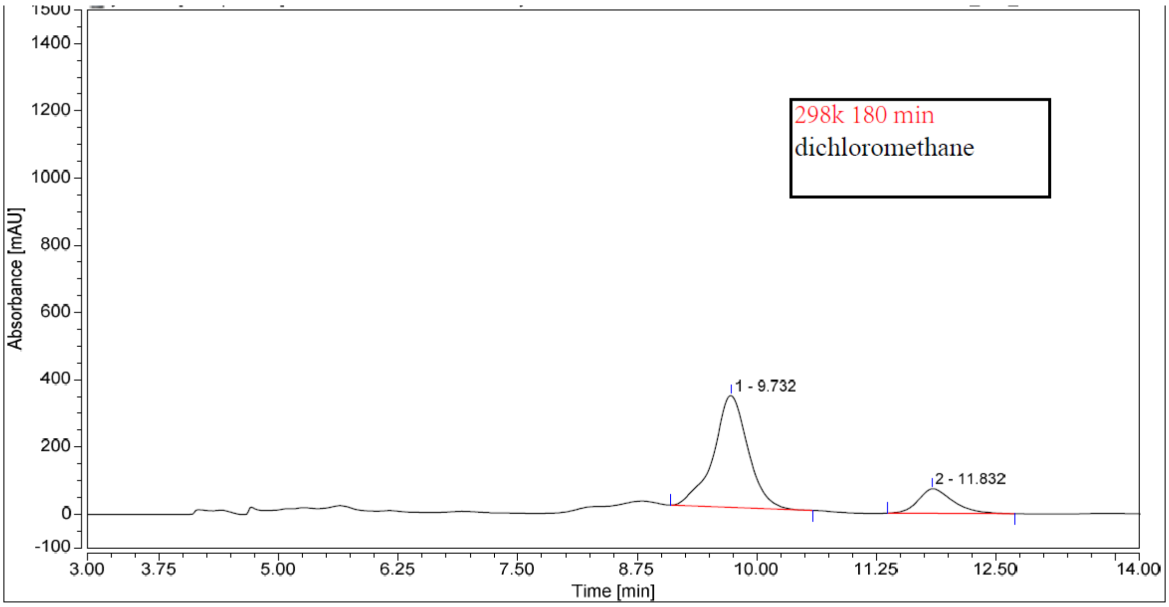

Integration Results							
No.	Peak Name	Retention Time min	Area mAU*min	Height mAU	Relative Area %	Relative Height %	Amount n.a.
1		9.732	139.546	332.351	82.05	81.92	n.a.
2		11.832	30.524	73.348	17.95	18.08	n.a.
**Total:**			**170.070**	**405.699**	**100.00**	**100.00**	

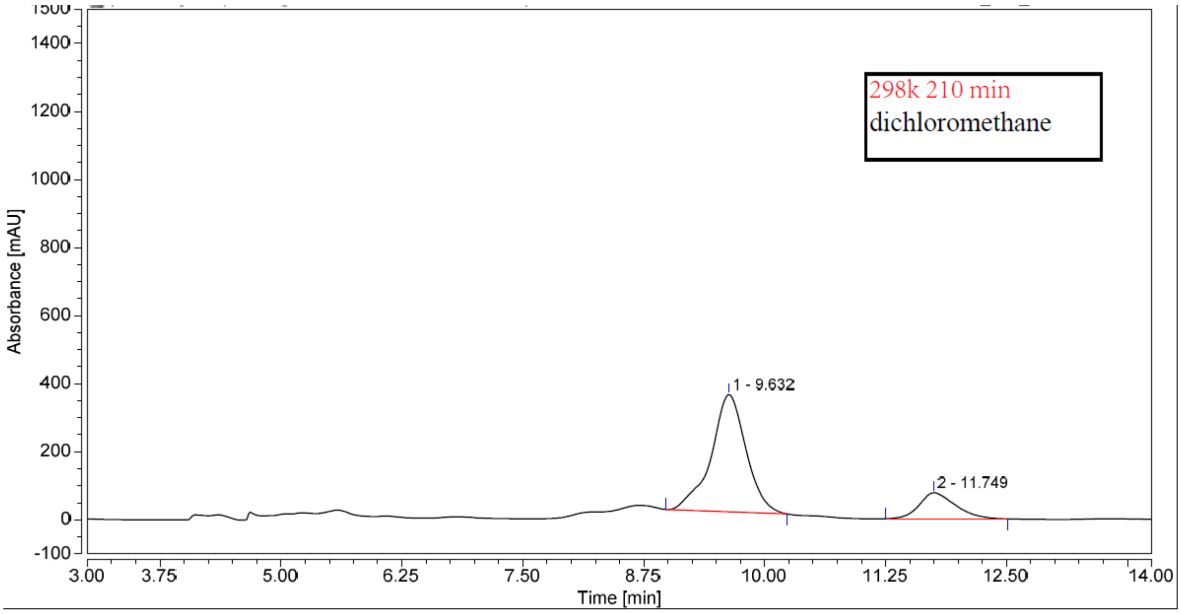

Integration Results							
No.	Peak Name	Retention Time min	Area mAU*min	Height mAU	Relative Area %	Relative Height %	Amount n.a.
1		9.632	145.134	344.666	81.49	81.82	n.a.
2		11.749	32.968	76.607	18.51	18.18	n.a.
**Total:**			**178.102**	**421.273**	**100.00**	**100.00**	

^{1}H and ^{13}C NMR spectra

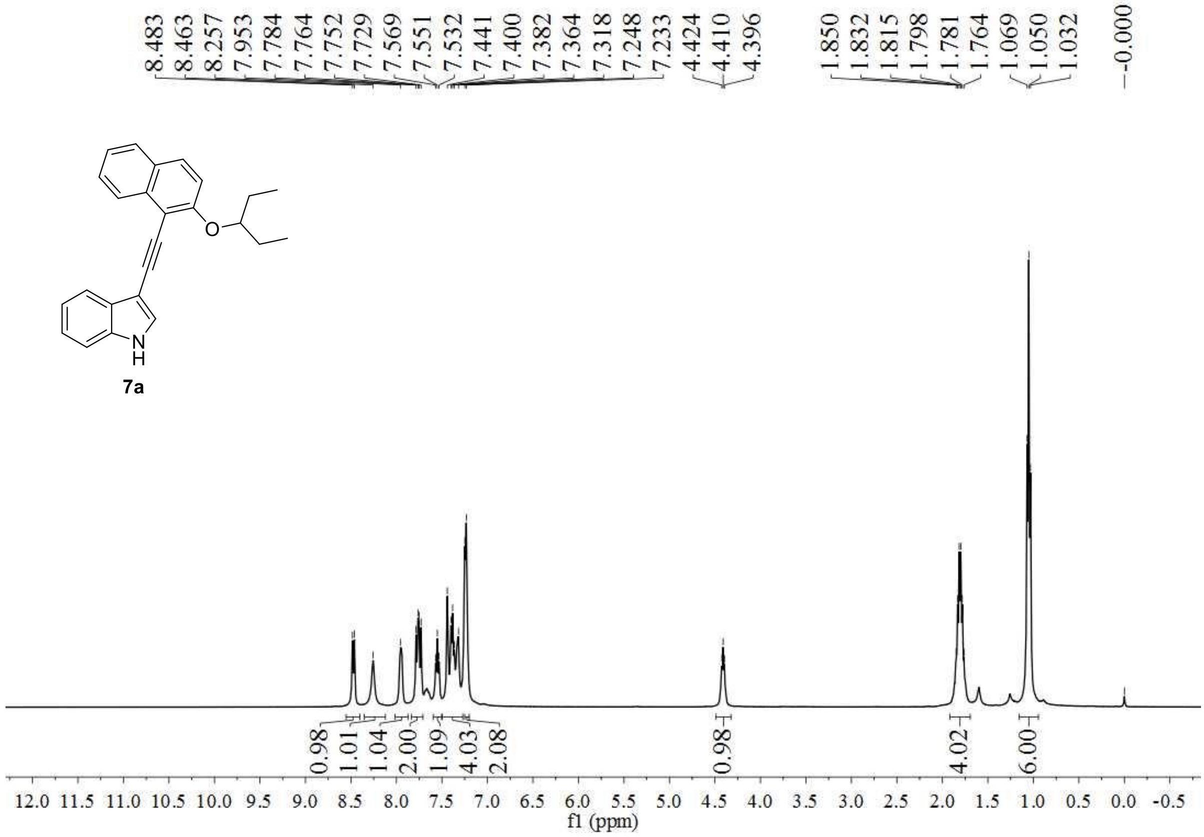

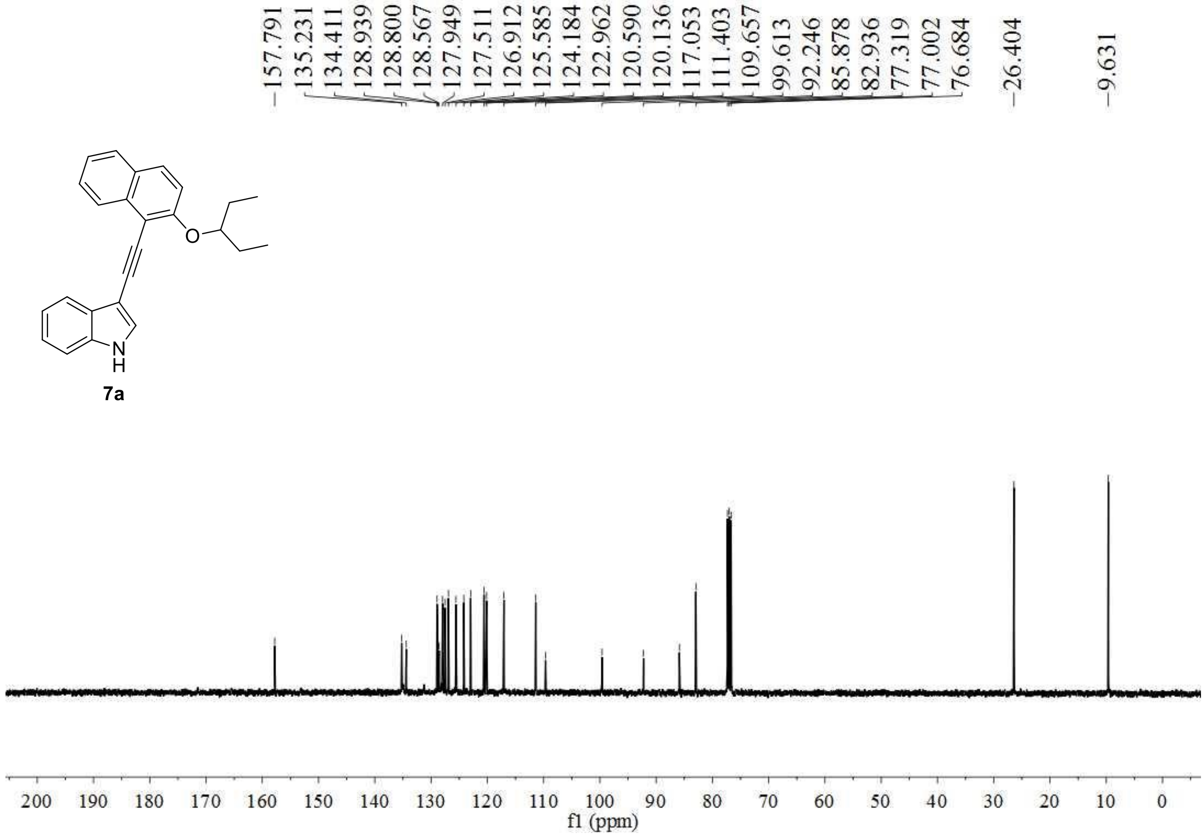

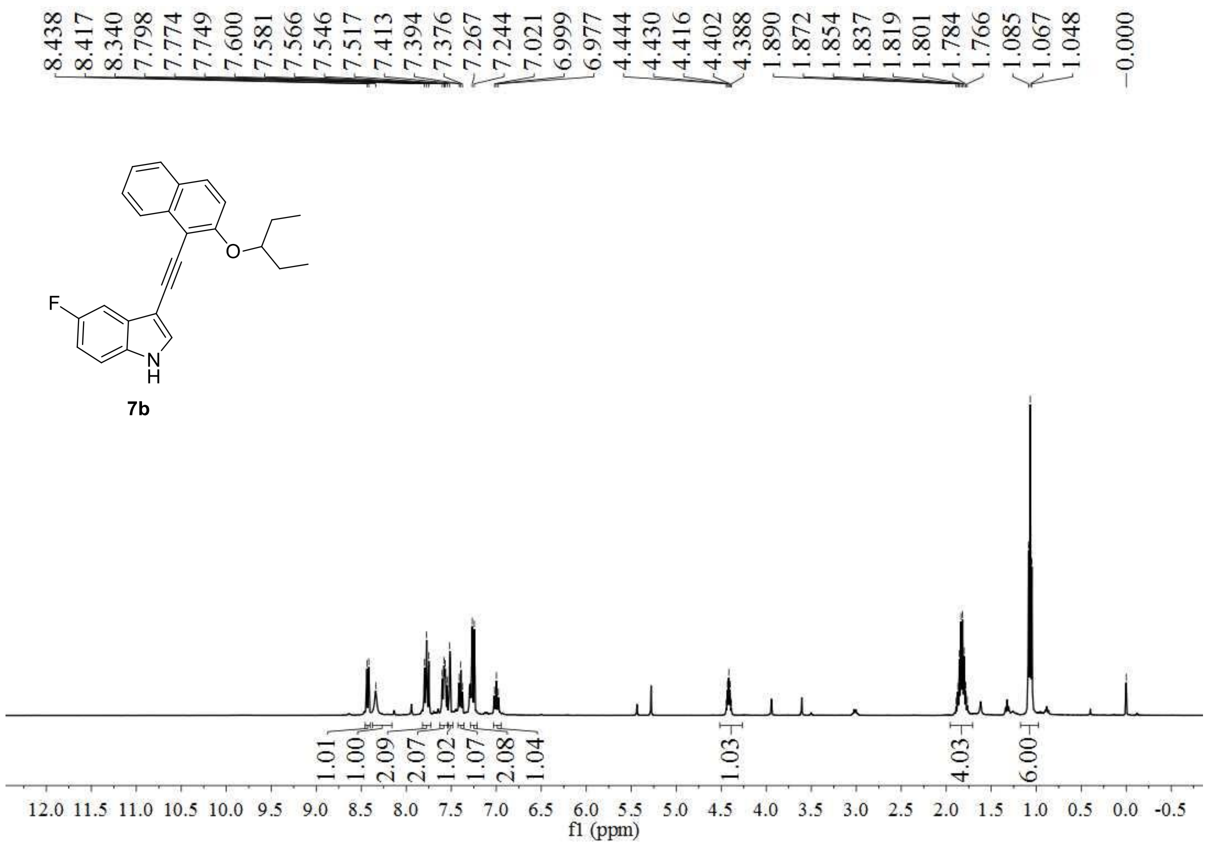
8.438
8.417
8.340
7.798
7.774
7.749
7.600
7.581
7.566
7.546
7.517
7.413
7.394
7.376
7.267
7.244
7.021
6.999
6.977
4.444
4.430
4.416
4.402
4.388
1.890
1.872
1.854
1.837
1.819
1.801
1.784
1.766
1.085
1.067
1.048
-0.000
F
O
N
H
7b
1.01
1.00
2.09
2.07
1.02
1.07
2.08
1.04
1.03
4.03
6.00
12.0 11.5 11.0 10.5 10.0 9.5 9.0 8.5 8.0 7.5 7.0 6.5 6.0 5.5 5.0 4.5 4.0 3.5 3.0 2.5 2.0 1.5 1.0 0.5 0.0 -0.5
f1 (ppm)

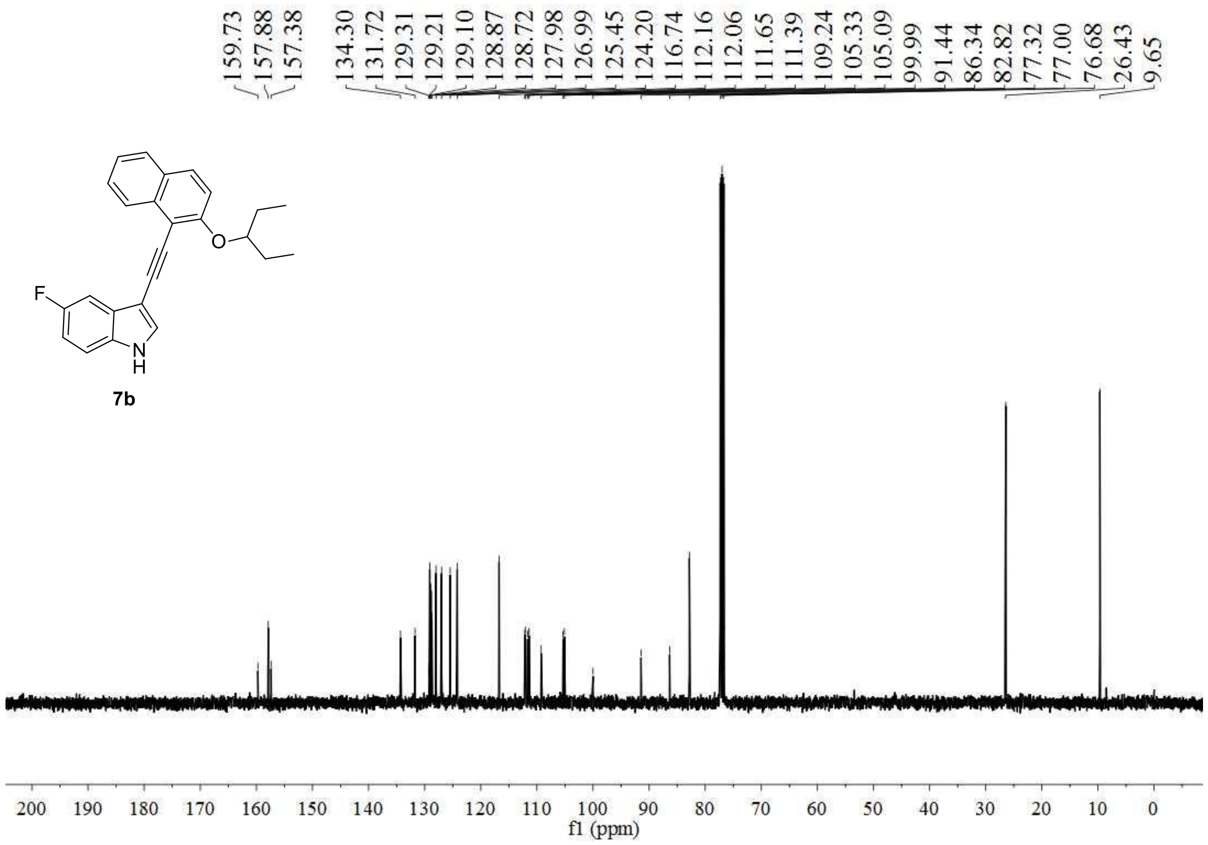
159.73
157.88
157.38
134.30
131.72
129.31
129.21
129.10
128.87
128.72
127.98
126.99
125.45
124.20
116.74
112.16
112.06
111.65
111.39
109.24
105.33
105.09
99.99
91.44
86.34
82.82
77.32
77.00
76.68
26.43
9.65
F
O
N
H
7b
200 190 180 170 160 150 140 130 120 110 100 90 80 70 60 50 40 30 20 10 0
f1 (ppm)

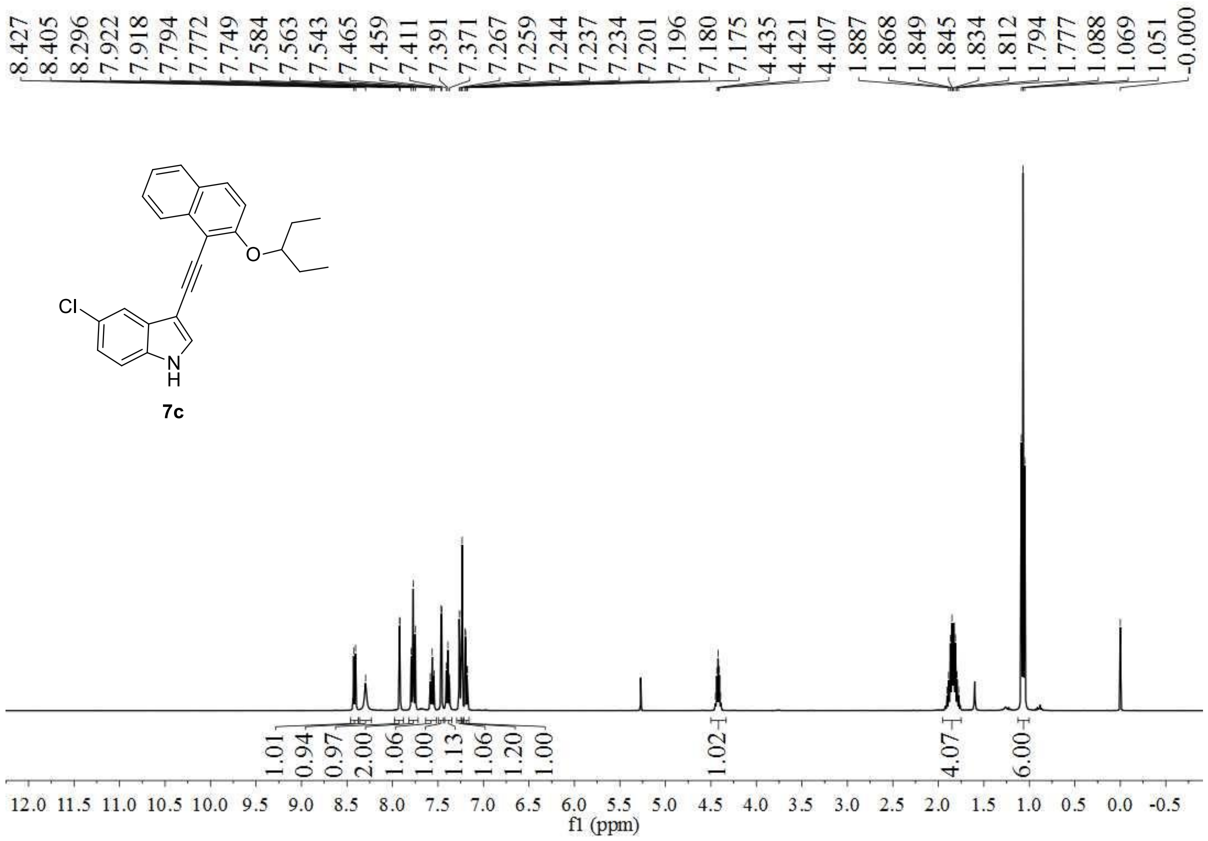
8.427 8.405 8.296 7.922 7.918 7.794 7.772 7.749 7.584 7.563 7.543 7.465 7.459 7.411 7.391 7.371 7.267 7.259 7.244 7.237 7.234 7.201 7.196 7.180 7.175 4.435 4.421 4.407 1.887 1.868 1.849 1.845 1.834 1.812 1.794 1.777 1.088 1.069 1.051 -0.000
Cl
O
N
H
7c
1.01 0.94 0.97 2.00 1.06 1.00 1.13 1.06 1.20 1.00
1.02
4.07
6.00
12.0 11.5 11.0 10.5 10.0 9.5 9.0 8.5 8.0 7.5 7.0 6.5 6.0 5.5 5.0 4.5 4.0 3.5 3.0 2.5 2.0 1.5 1.0 0.5 0.0 -0.5
f1 (ppm)

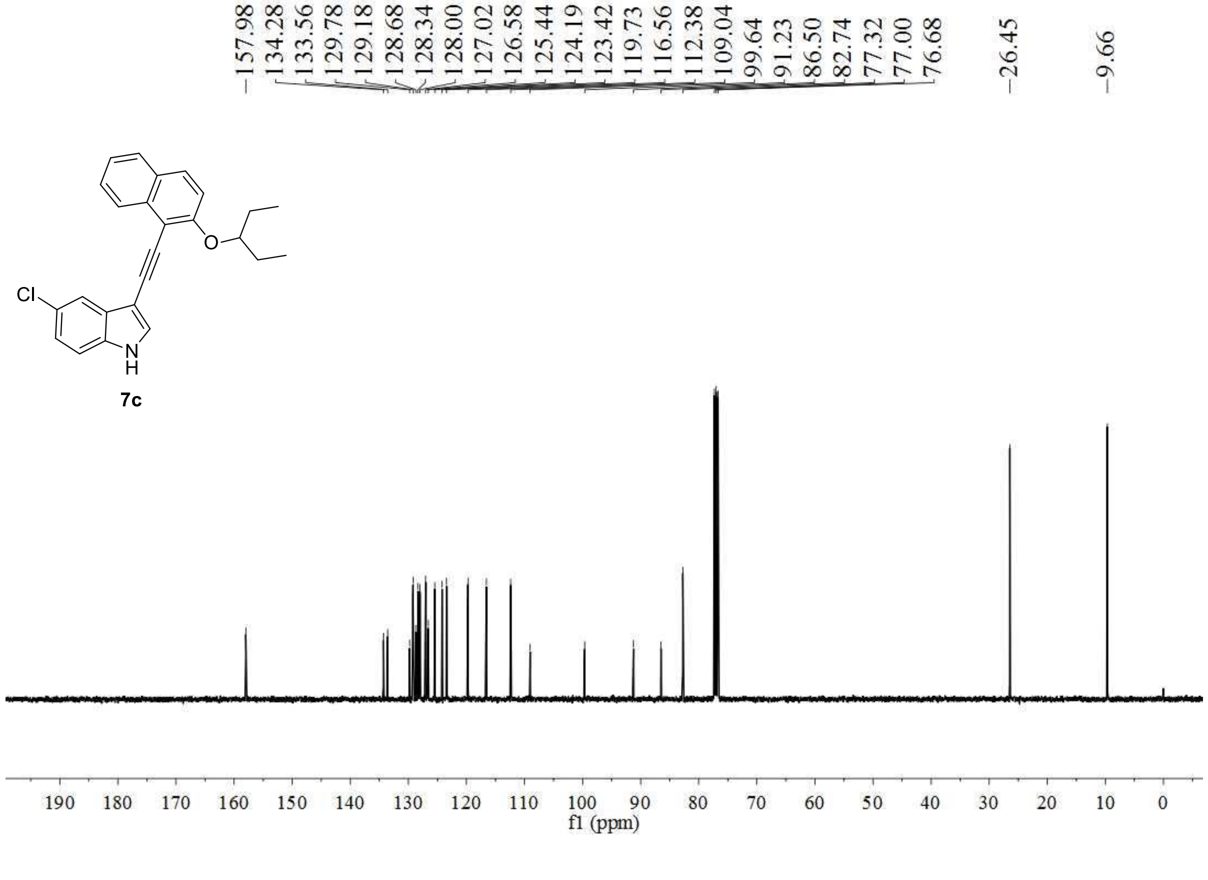
157.98 134.28 133.56 129.78 129.18 128.68 128.34 128.00 127.02 126.58 125.44 124.19 123.42 119.73 116.56 112.38 109.04 99.64 91.23 86.50 82.74 77.32 77.00 76.68 26.45 9.66
Cl
O
N
H
7c
190 180 170 160 150 140 130 120 110 100 90 80 70 60 50 40 30 20 10 0
f1 (ppm)

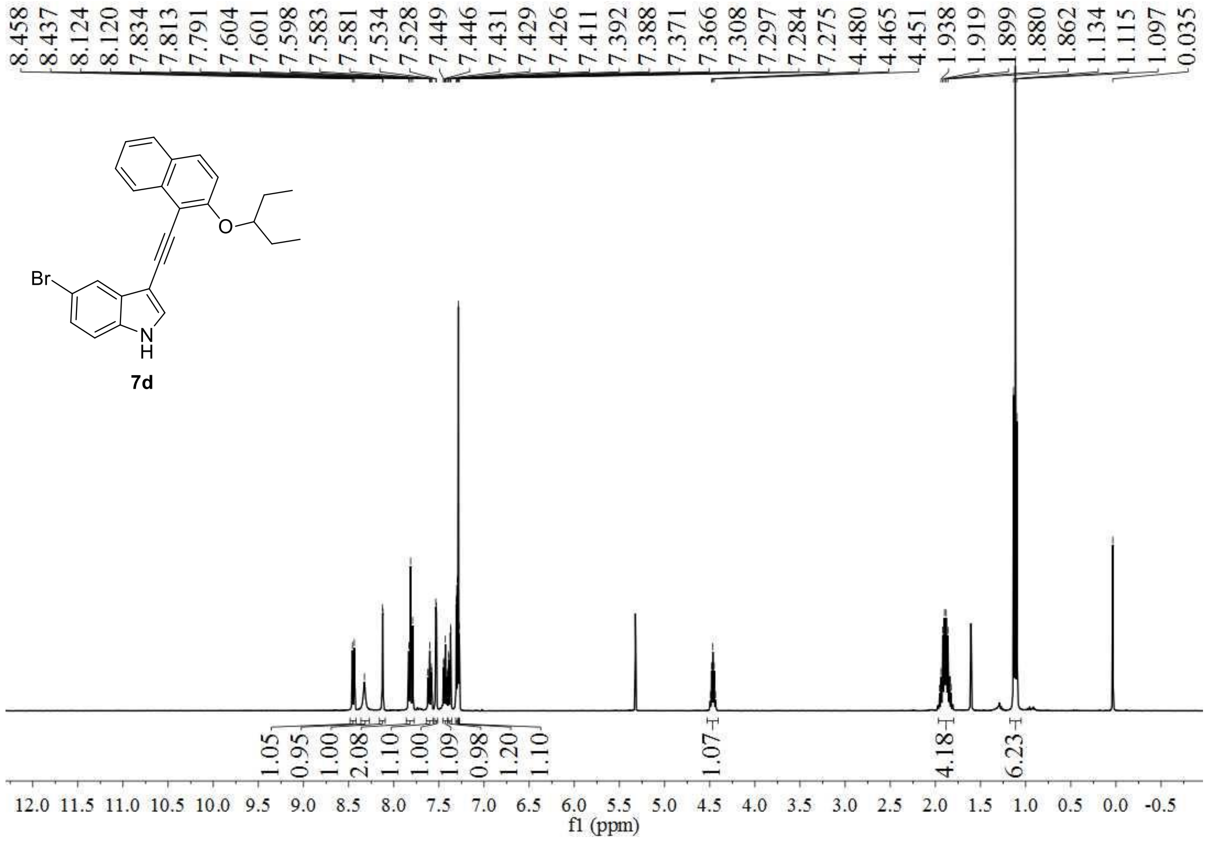
8.458 8.437 8.124 8.120 7.834 7.813 7.791 7.604 7.601 7.598 7.583 7.581 7.534 7.528 7.449 7.446 7.431 7.429 7.426 7.411 7.392 7.388 7.371 7.366 7.308 7.297 7.284 7.275 4.480 4.465 4.451 1.938 1.919 1.899 1.880 1.862 1.134 1.115 1.097 0.035
Br
O
N
H
7d
1.05 0.95 1.00 2.08 1.10 1.00 1.09 0.98 1.20 1.10
1.07
4.18
6.23
12.0 11.5 11.0 10.5 10.0 9.5 9.0 8.5 8.0 7.5 7.0 6.5 6.0 5.5 5.0 4.5 4.0 3.5 3.0 2.5 2.0 1.5 1.0 0.5 0.0 -0.5
f1 (ppm)

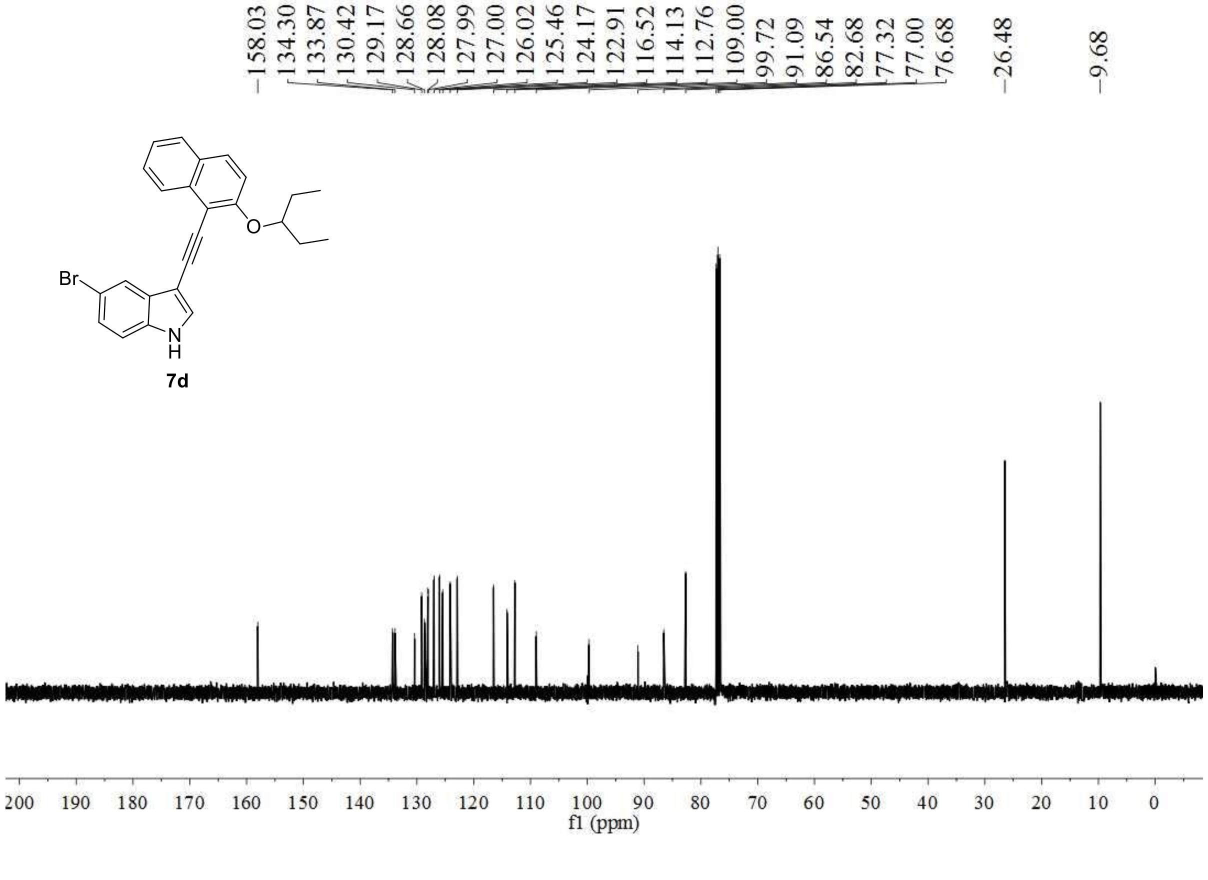
158.03 134.30 133.87 130.42 129.17 128.66 128.08 127.99 127.00 126.02 125.46 124.17 122.91 116.52 114.13 112.76 109.00 99.72 91.09 86.54 82.68 77.32 77.00 76.68
26.48
9.68
Br
O
N
H
7d
200 190 180 170 160 150 140 130 120 110 100 90 80 70 60 50 40 30 20 10 0
f1 (ppm)

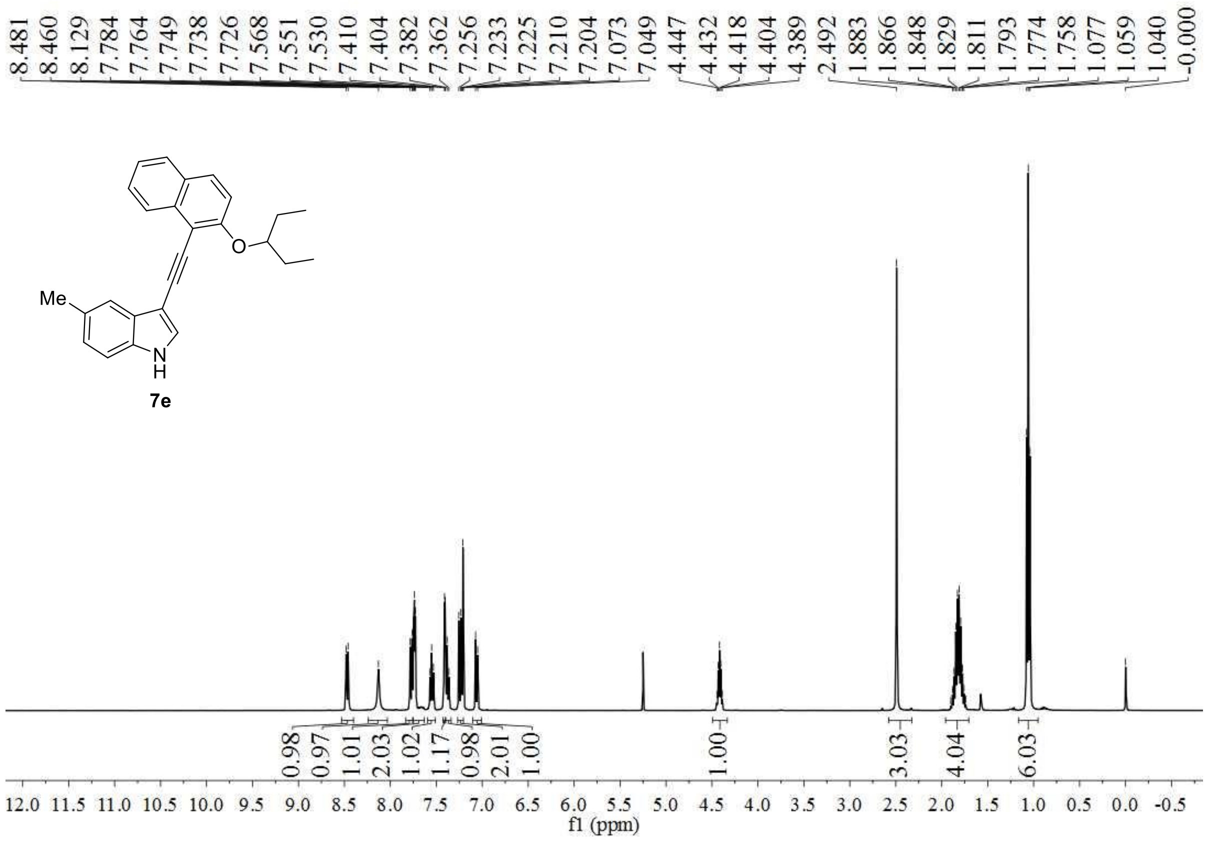
8.481 8.460 8.129 7.784 7.764 7.749 7.738 7.726 7.568 7.551 7.530 7.410 7.404 7.382 7.362 7.256 7.233 7.225 7.210 7.204 7.073 7.049 4.447 4.432 4.418 4.404 4.389 2.492 1.883 1.866 1.848 1.829 1.811 1.793 1.774 1.758 1.077 1.059 1.040 -0.000
Me
O
N
H
7e
0.98 0.97 1.01 2.03 1.02 1.17 0.98 2.01 1.00 1.00 3.03 4.04 6.03
12.0 11.5 11.0 10.5 10.0 9.5 9.0 8.5 8.0 7.5 7.0 6.5 6.0 5.5 5.0 4.5 4.0 3.5 3.0 2.5 2.0 1.5 1.0 0.5 0.0 -0.5
f1 (ppm)

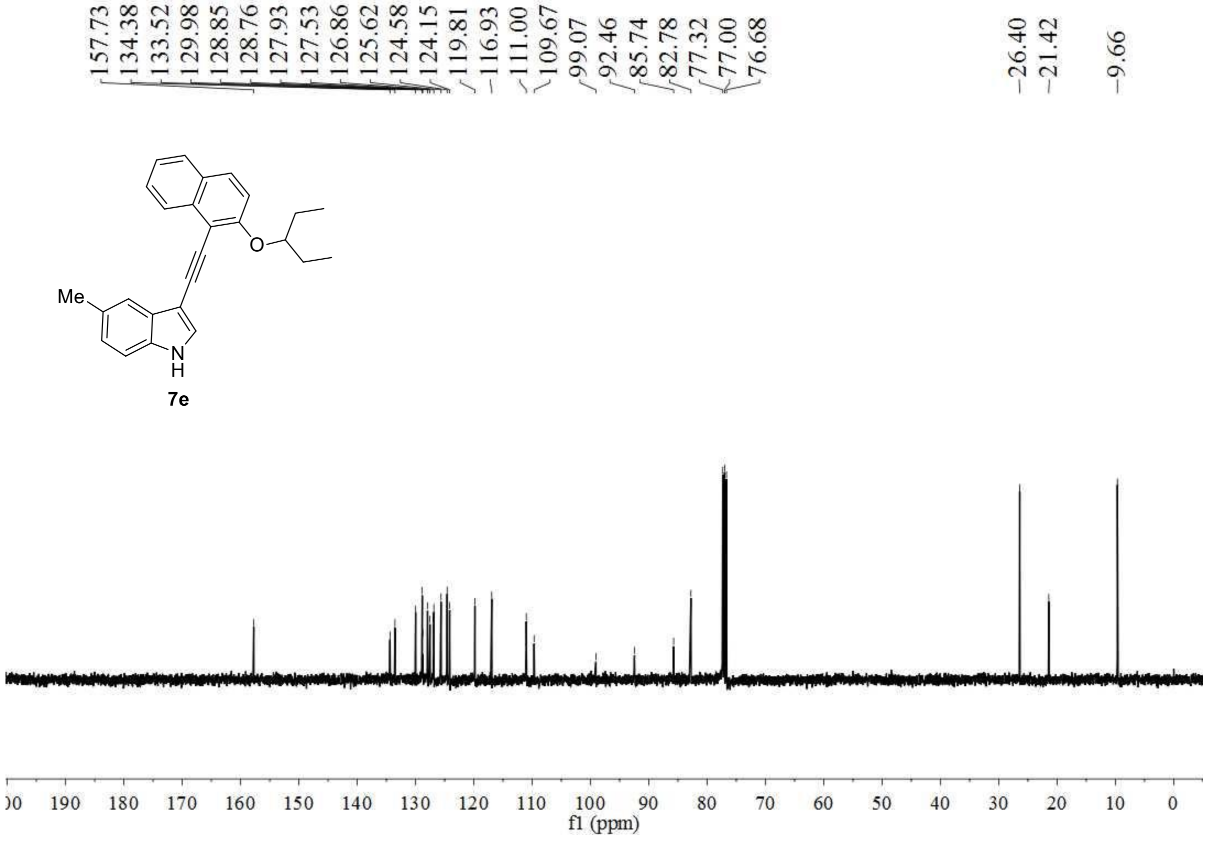
157.73 134.38 133.52 129.98 128.85 128.76 127.93 127.53 126.86 125.62 124.58 124.15 119.81 116.93 111.00 109.67 99.07 92.46 85.74 82.78 77.32 77.00 76.68
26.40 21.42
9.66
Me
O
N
H
7e
190 180 170 160 150 140 130 120 110 100 90 80 70 60 50 40 30 20 10 0
f1 (ppm)

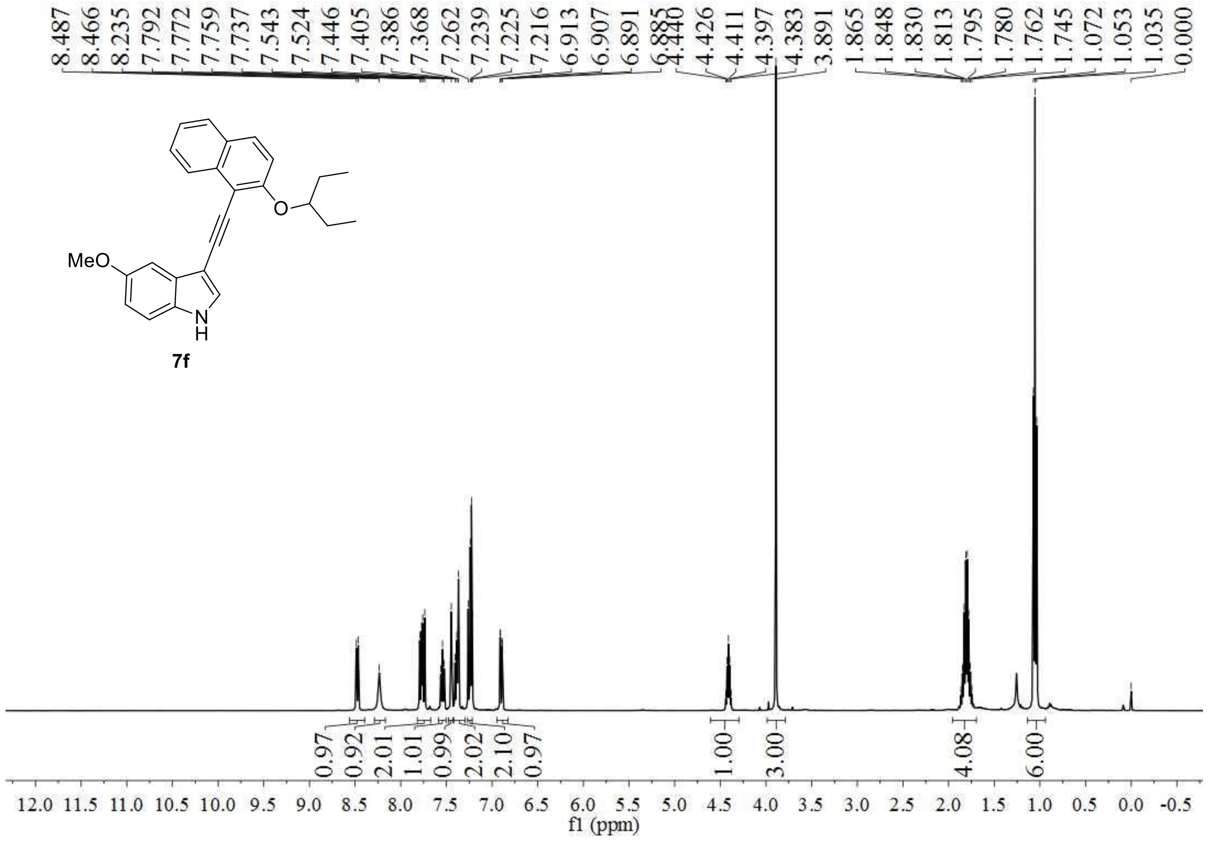

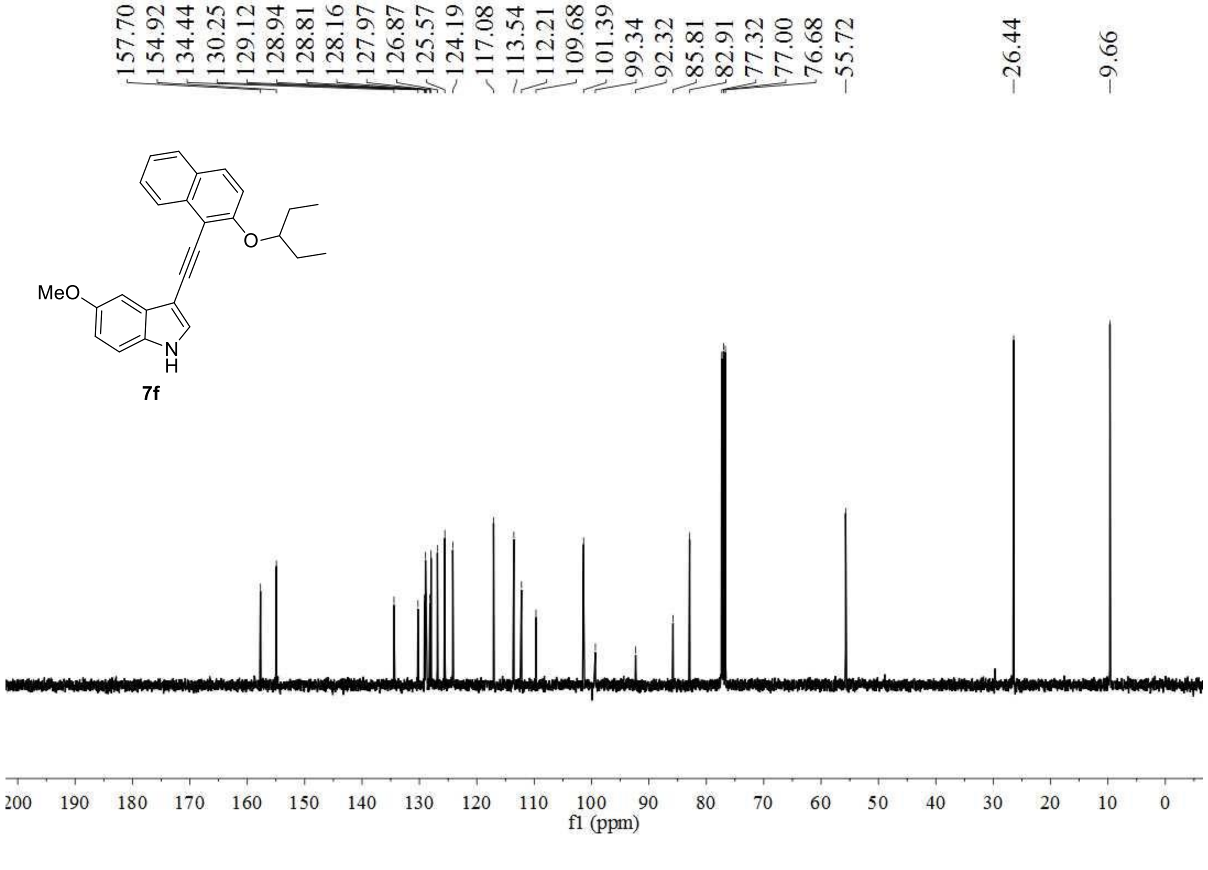

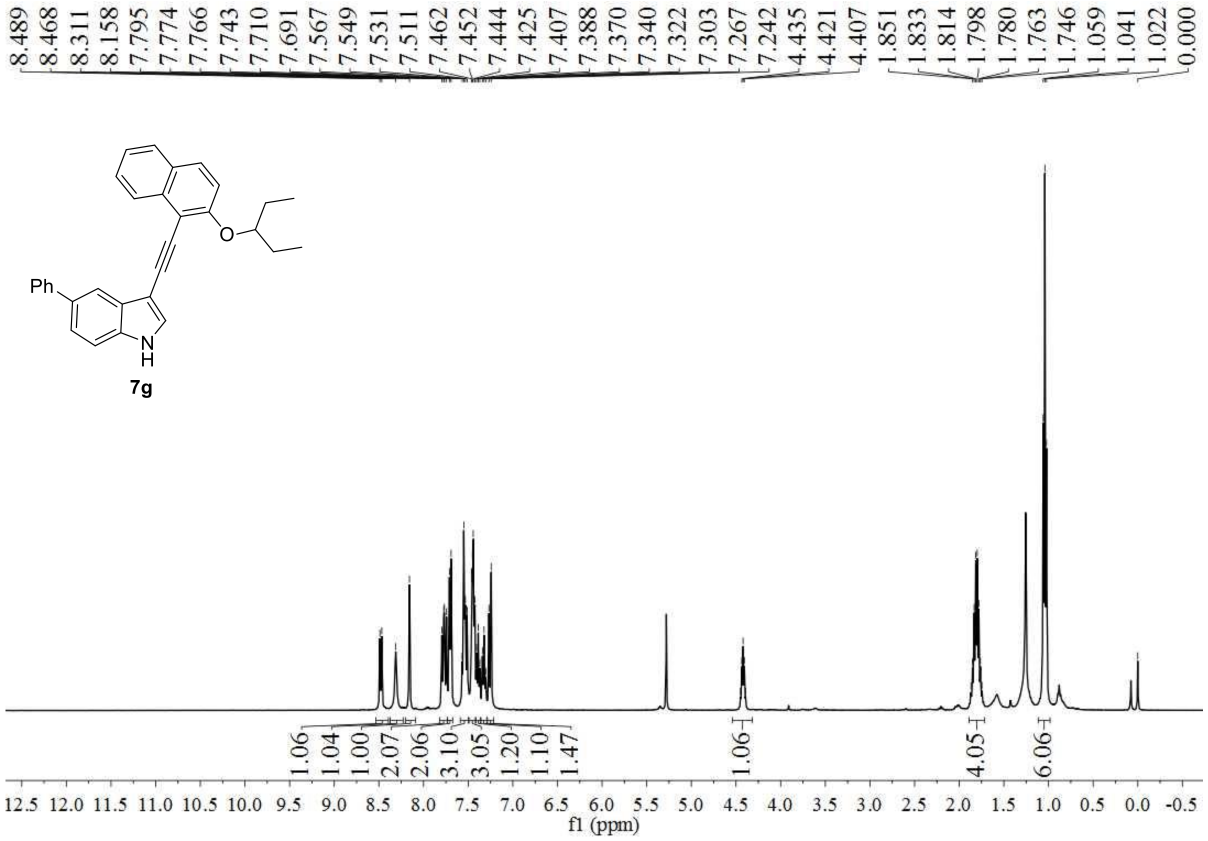
8.489 8.468 8.311 8.158 7.795 7.774 7.766 7.743 7.710 7.691 7.567 7.549 7.531 7.511 7.462 7.452 7.444 7.425 7.407 7.388 7.370 7.340 7.322 7.303 7.267 7.242 4.435 4.421 4.407 1.851 1.833 1.814 1.798 1.780 1.763 1.746 1.059 1.041 1.022 0.000
Ph
O
N
H
7g
1.06 1.04 1.00 2.07 2.06 3.10 3.05 1.20 1.10 1.47
1.06
4.05
6.06
f1 (ppm)

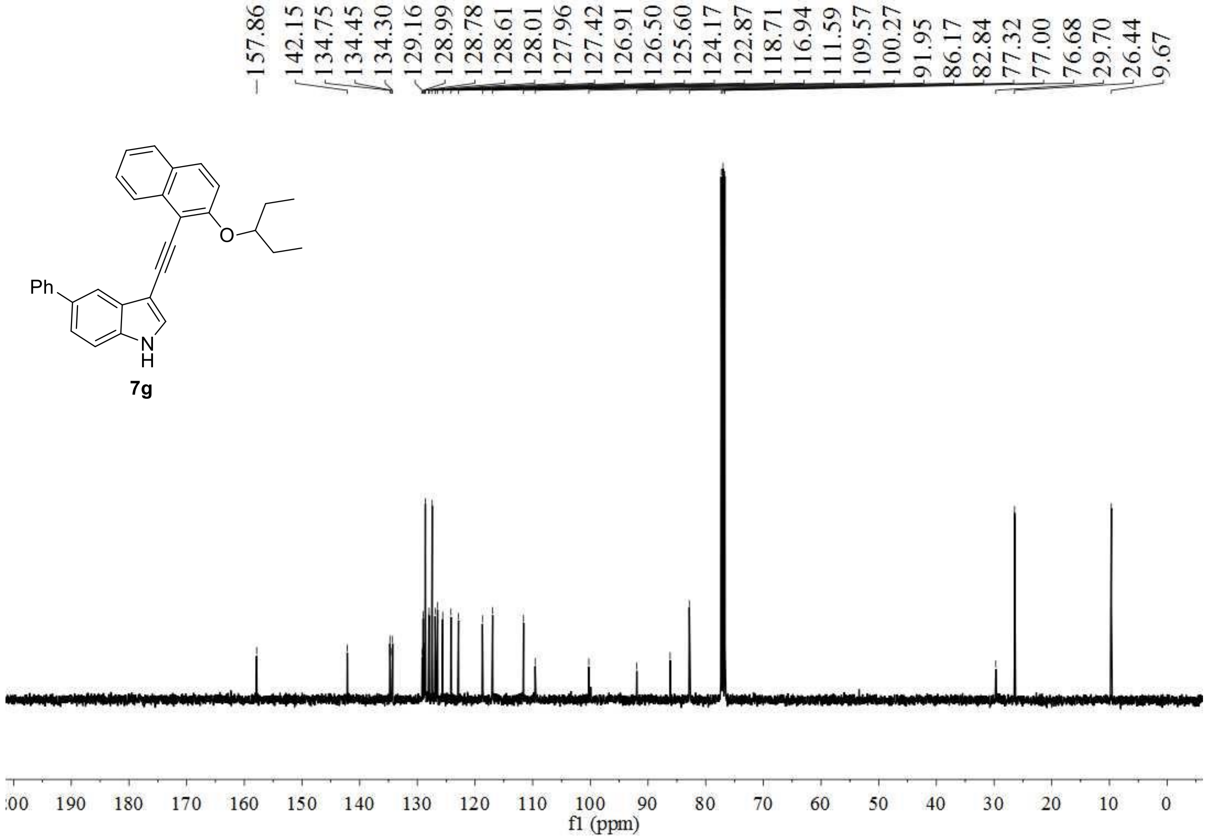
157.86 142.15 134.75 134.45 134.30 129.16 128.99 128.78 128.61 128.01 127.96 127.42 126.91 126.50 125.60 124.17 122.87 118.71 116.94 111.59 109.57 100.27 91.95 86.17 82.84 77.32 77.00 76.68 29.70 26.44 9.67
Ph
O
N
H
7g
f1 (ppm)

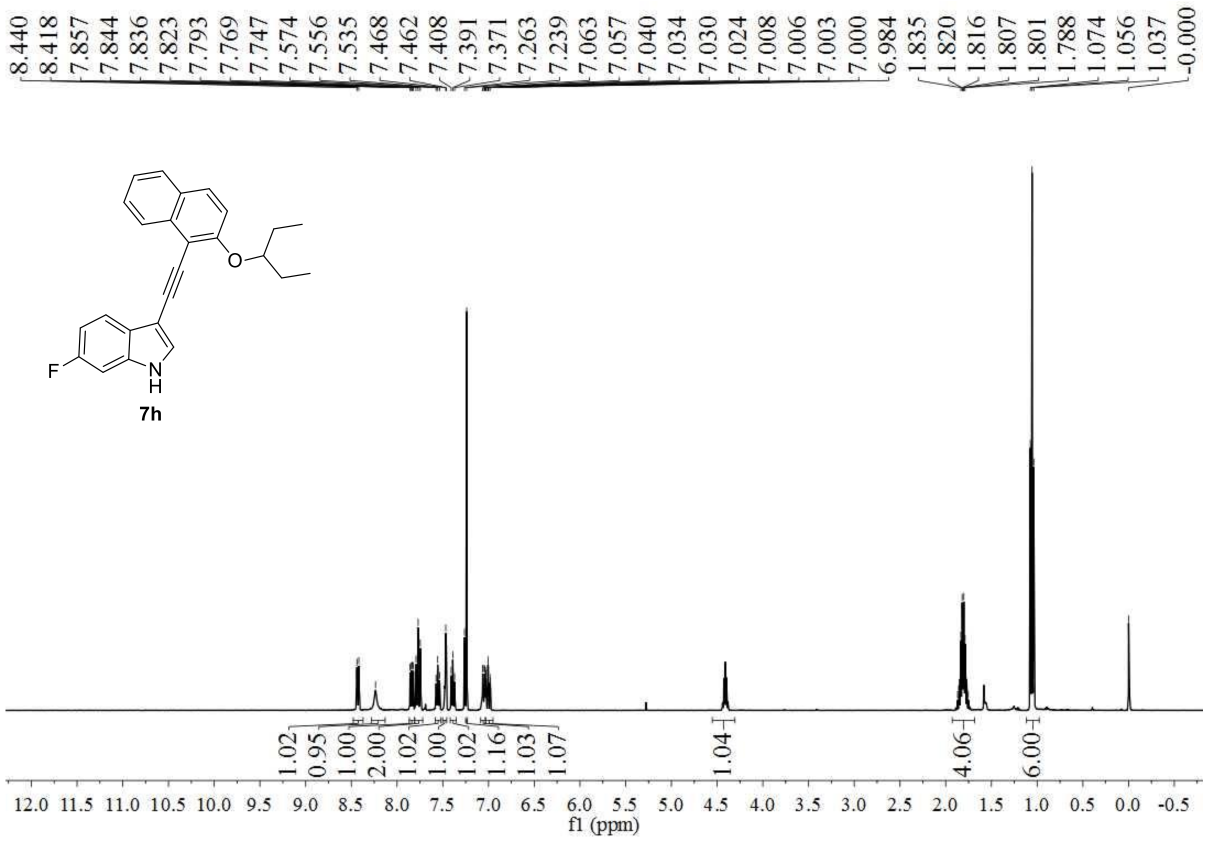
8.440 8.418 7.857 7.844 7.836 7.823 7.793 7.769 7.747 7.574 7.556 7.535 7.468 7.462 7.408 7.391 7.371 7.263 7.239 7.063 7.057 7.040 7.034 7.030 7.024 7.008 7.006 7.003 7.000 6.984 1.835 1.820 1.816 1.807 1.801 1.788 1.074 1.056 1.037 -0.000
F
N
H
O
7h
1.02 0.95 1.00 2.00 1.02 1.00 1.02 1.16 1.03 1.07 1.04 4.06 6.00
12.0 11.5 11.0 10.5 10.0 9.5 9.0 8.5 8.0 7.5 7.0 6.5 6.0 5.5 5.0 4.5 4.0 3.5 3.0 2.5 2.0 1.5 1.0 0.5 0.0 -0.5
f1 (ppm)

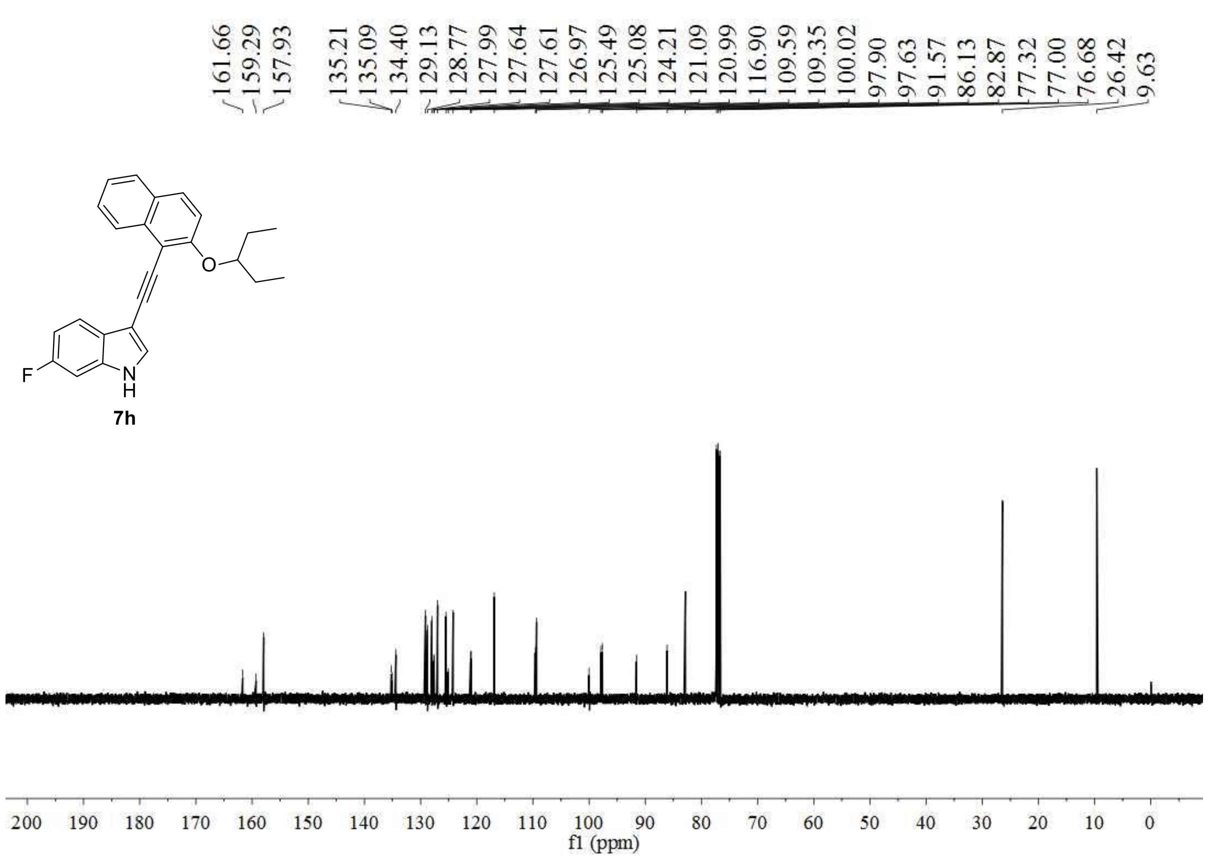
161.66 159.29 157.93 135.21 135.09 134.40 129.13 128.77 127.99 127.64 127.61 126.97 125.49 125.08 124.21 121.09 120.99 116.90 109.59 109.35 100.02 97.90 97.63 91.57 86.13 82.87 77.32 77.00 76.68 26.42 9.63
F
N
H
O
7h
200 190 180 170 160 150 140 130 120 110 100 90 80 70 60 50 40 30 20 10 0
f1 (ppm)

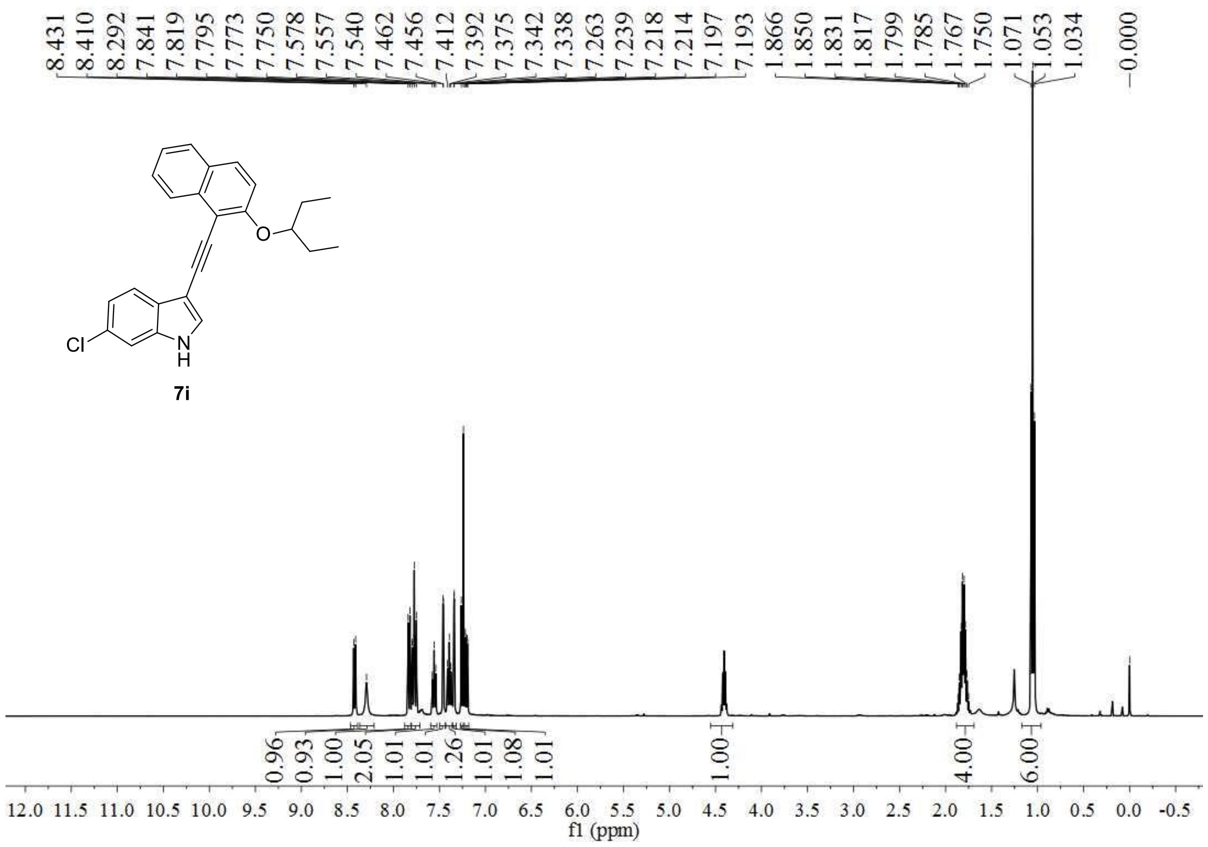
8.431
8.410
8.292
7.841
7.819
7.795
7.773
7.750
7.578
7.557
7.540
7.462
7.456
7.412
7.392
7.375
7.342
7.338
7.263
7.239
7.218
7.214
7.197
7.193
1.866
1.850
1.831
1.817
1.799
1.785
1.767
1.750
1.071
1.053
1.034
0.000
Cl
N
H
O
7i
0.96
0.93
1.00
2.05
1.01
1.01
1.26
1.01
1.08
1.01
1.00
4.00
6.00
12.0 11.5 11.0 10.5 10.0 9.5 9.0 8.5 8.0 7.5 7.0 6.5 6.0 5.5 5.0 4.5 4.0 3.5 3.0 2.5 2.0 1.5 1.0 0.5 0.0 -0.5
f1 (ppm)

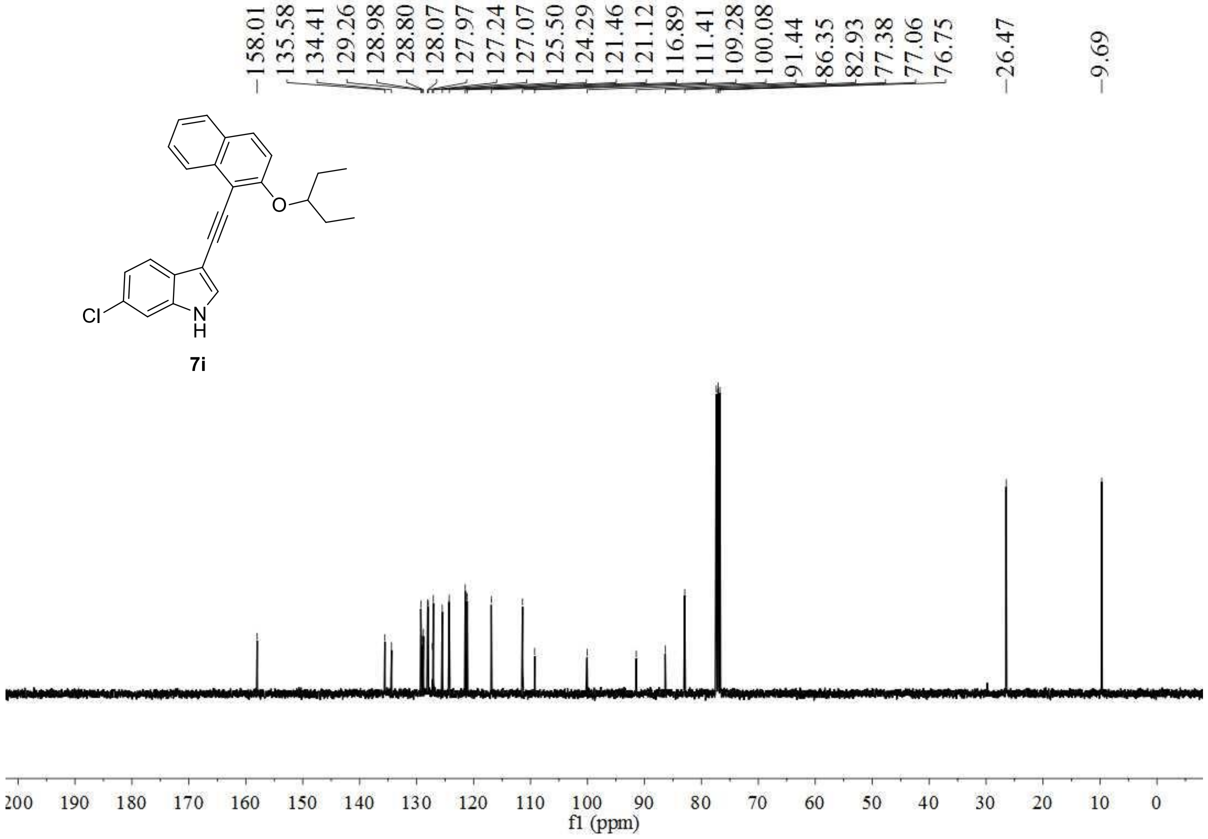
158.01
135.58
134.41
129.26
128.98
128.80
128.07
127.97
127.24
127.07
125.50
124.29
121.46
121.12
116.89
111.41
109.28
100.08
91.44
86.35
82.93
77.38
77.06
76.75
26.47
9.69
Cl
N
H
O
7i
200 190 180 170 160 150 140 130 120 110 100 90 80 70 60 50 40 30 20 10 0
f1 (ppm)

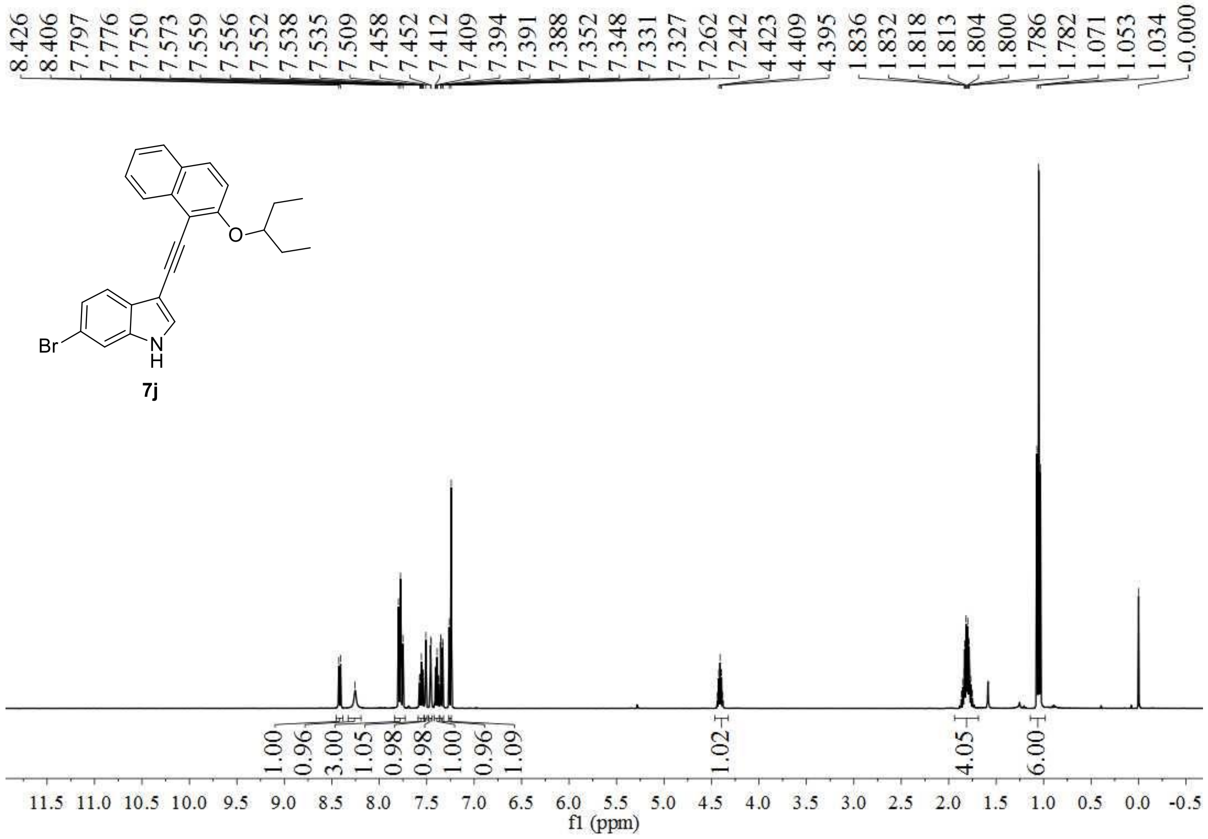

7j

8.426
8.406
7.797
7.776
7.750
7.573
7.559
7.556
7.552
7.538
7.535
7.509
7.458
7.452
7.412
7.409
7.394
7.391
7.388
7.352
7.348
7.331
7.327
7.262
7.242
4.423
4.409
4.395
1.836
1.832
1.818
1.813
1.804
1.800
1.786
1.782
1.071
1.053
1.034
-0.000
1.00
0.96
3.00
1.05
0.98
0.98
1.00
0.96
1.09
1.02
4.05
6.00
11.5 11.0 10.5 10.0 9.5 9.0 8.5 8.0 7.5 7.0 6.5 6.0 5.5 5.0 4.5 4.0 3.5 3.0 2.5 2.0 1.5 1.0 0.5 0.0 -0.5
f1 (ppm)

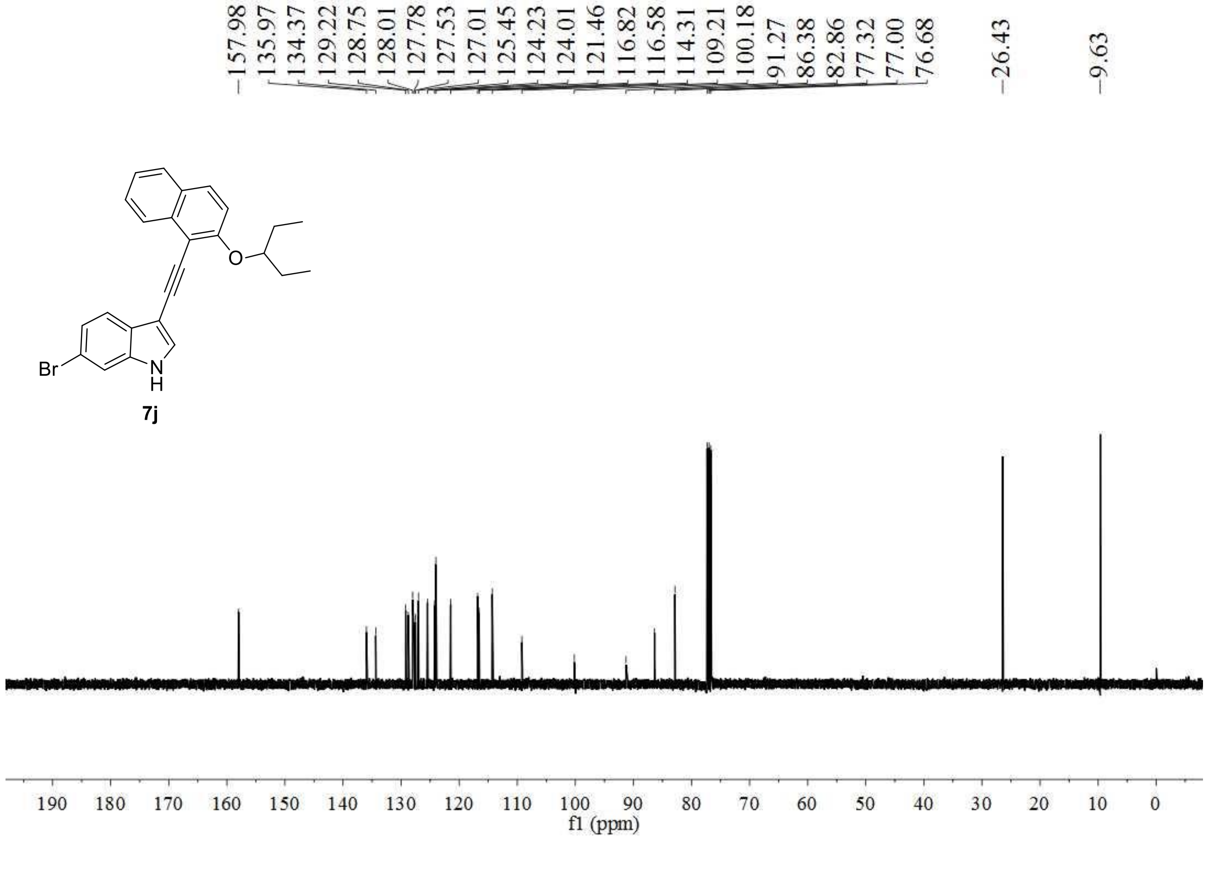

7j

157.98
135.97
134.37
129.22
128.75
128.01
127.78
127.53
127.01
125.45
124.23
124.01
121.46
116.82
116.58
114.31
109.21
100.18
91.27
86.38
82.86
77.32
77.00
76.68
26.43
9.63
190 180 170 160 150 140 130 120 110 100 90 80 70 60 50 40 30 20 10 0
f1 (ppm)

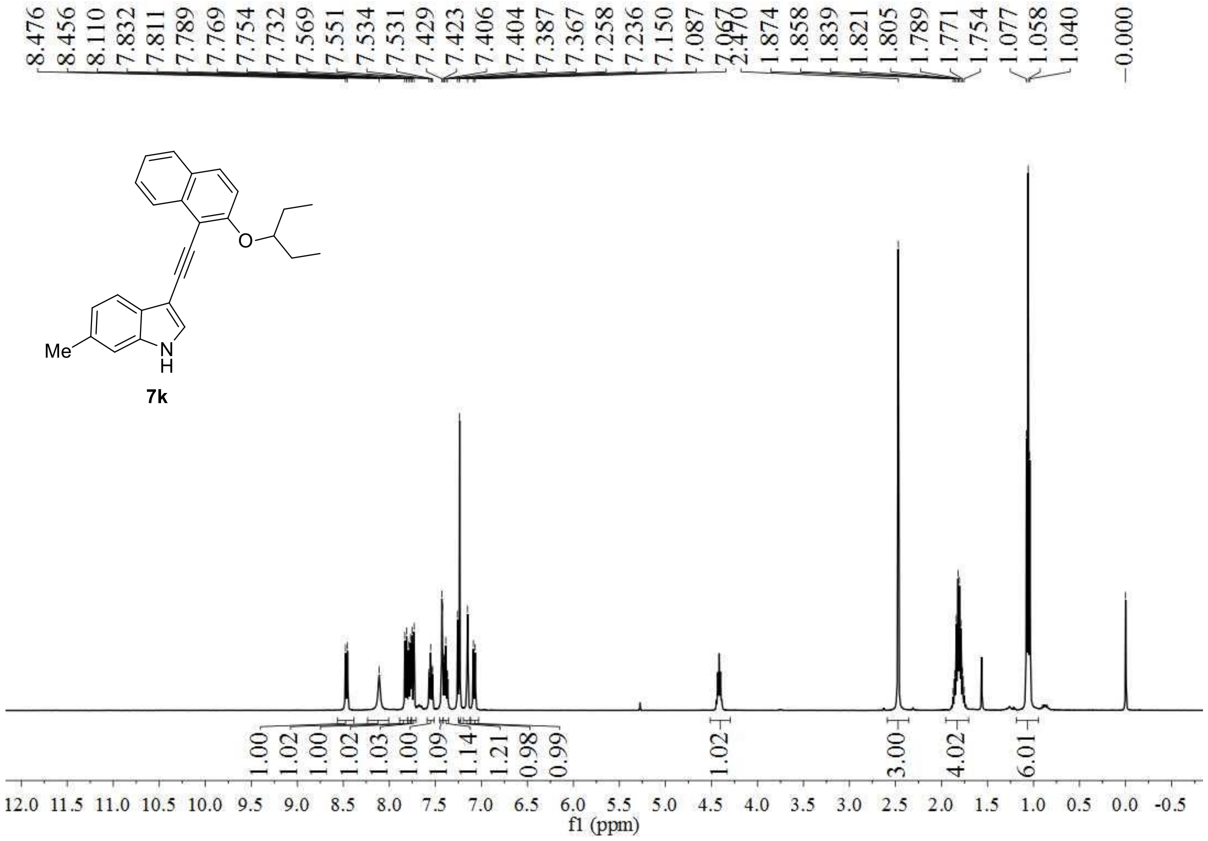
8.476
8.456
8.110
7.832
7.811
7.789
7.769
7.754
7.732
7.569
7.551
7.534
7.531
7.429
7.423
7.406
7.404
7.387
7.367
7.258
7.236
7.150
7.087
7.067
2.470
1.874
1.858
1.839
1.821
1.805
1.789
1.771
1.754
1.077
1.058
1.040
0.000
O
Me
N
H
7k
1.00
1.02
1.00
1.02
1.03
1.00
1.09
1.14
1.21
0.98
0.99
1.02
3.00
4.02
6.01
12.0 11.5 11.0 10.5 10.0 9.5 9.0 8.5 8.0 7.5 7.0 6.5 6.0 5.5 5.0 4.5 4.0 3.5 3.0 2.5 2.0 1.5 1.0 0.5 0.0 -0.5
f1 (ppm)

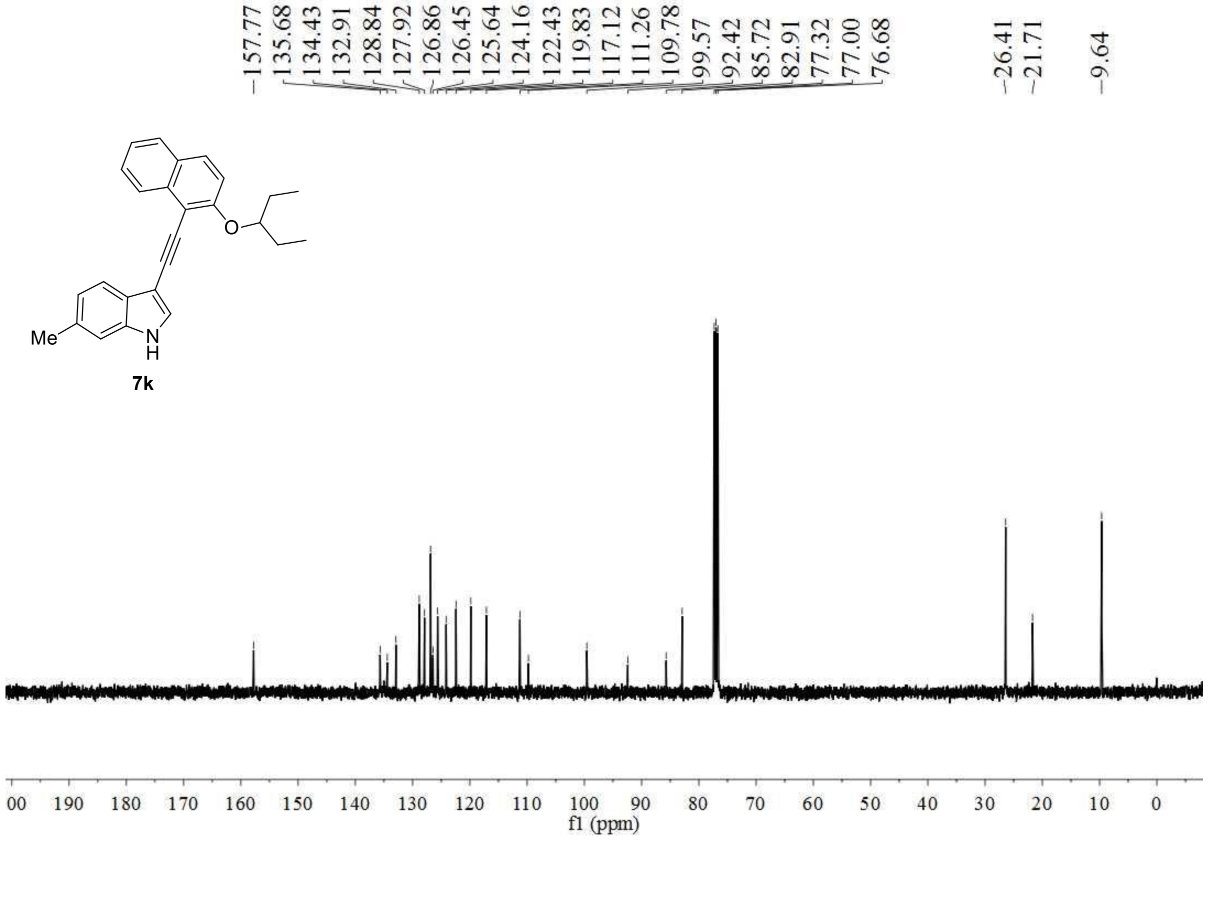
157.77
135.68
134.43
132.91
128.84
127.92
126.86
126.45
125.64
124.16
122.43
119.83
117.12
111.26
109.78
99.57
92.42
85.72
82.91
77.32
77.00
76.68
26.41
21.71
9.64
O
Me
N
H
7k
00 190 180 170 160 150 140 130 120 110 100 90 80 70 60 50 40 30 20 10 0
f1 (ppm)

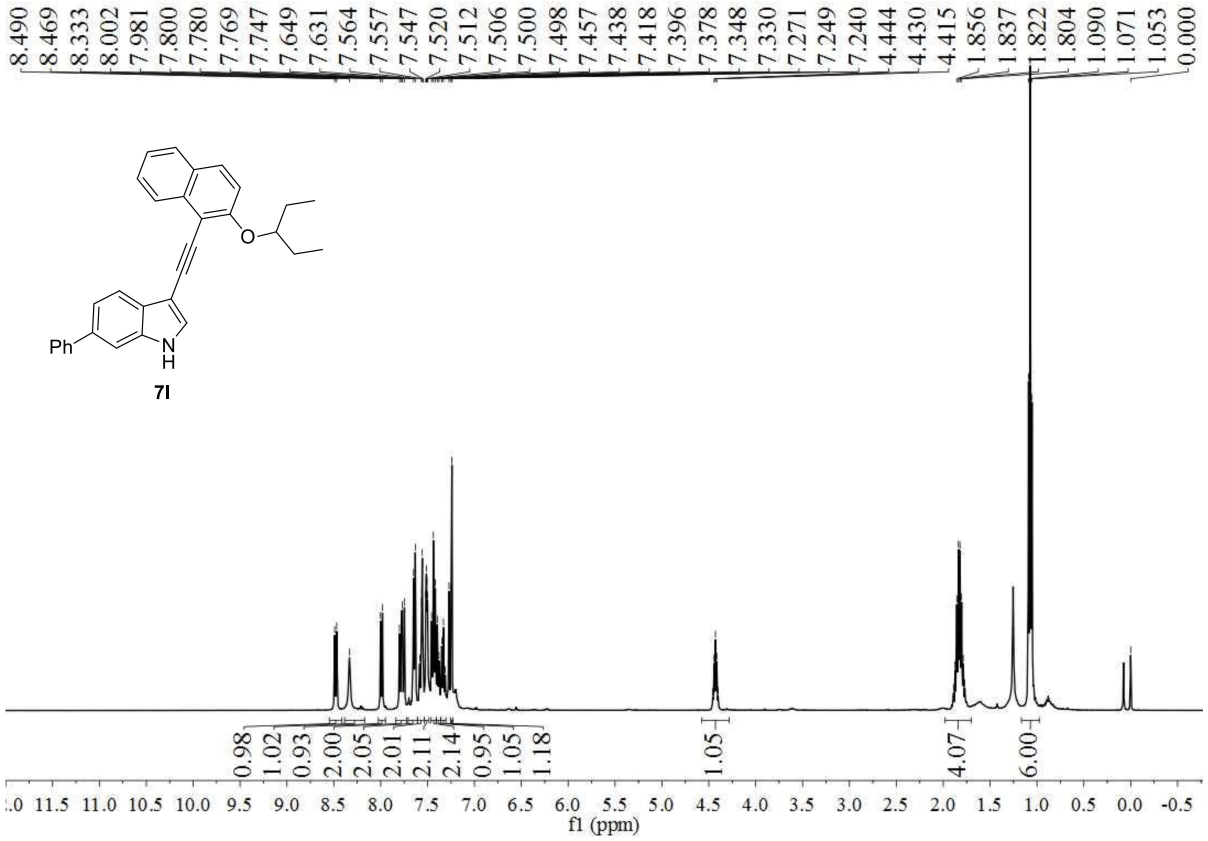
8.490
8.469
8.333
8.002
7.981
7.800
7.780
7.769
7.747
7.649
7.631
7.564
7.557
7.547
7.520
7.512
7.506
7.500
7.498
7.457
7.438
7.418
7.396
7.378
7.348
7.330
7.271
7.249
7.240
4.444
4.430
4.415
1.856
1.837
1.822
1.804
1.090
1.071
1.053
0.000
O
Ph
N
H
7l
0.98
1.02
0.93
2.00
2.05
2.01
2.11
2.14
0.95
1.05
1.18
1.05
4.07
6.00
f1 (ppm)

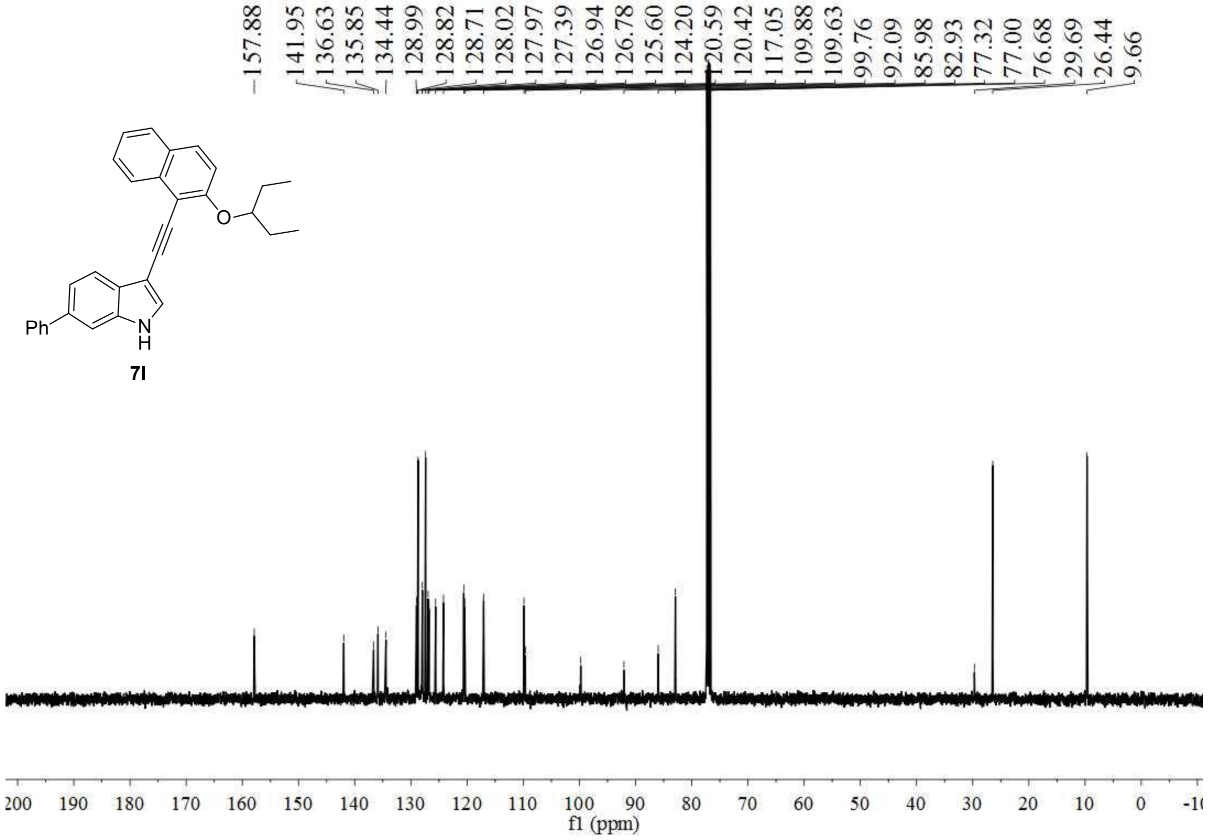
157.88
141.95
136.63
135.85
134.44
128.99
128.82
128.71
128.02
127.97
127.39
126.94
126.78
125.60
124.20
120.59
120.42
117.05
109.88
109.63
99.76
92.09
85.98
82.93
77.32
77.00
76.68
29.69
26.44
9.66
O
Ph
N
H
7l
f1 (ppm)

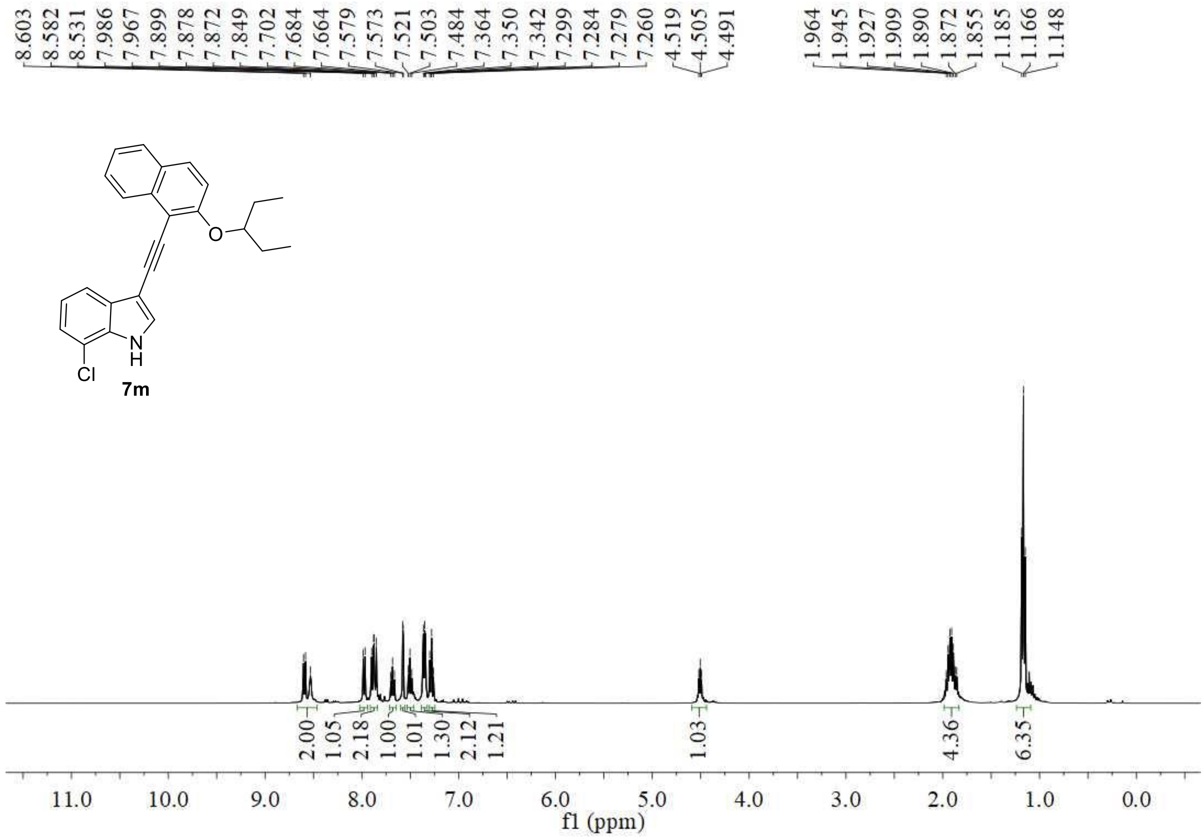
8.603
8.582
8.531
7.986
7.967
7.899
7.878
7.872
7.849
7.702
7.684
7.664
7.579
7.573
7.521
7.503
7.484
7.364
7.350
7.342
7.299
7.284
7.279
7.260
4.519
4.505
4.491
1.964
1.945
1.927
1.909
1.890
1.872
1.855
1.185
1.166
1.148
O
N
H
Cl
7m
2.00
1.05
2.18
1.00
1.01
1.30
2.12
1.21
1.03
4.36
6.35
11.0
10.0
9.0
8.0
7.0
6.0
5.0
4.0
3.0
2.0
1.0
0.0
f1 (ppm)

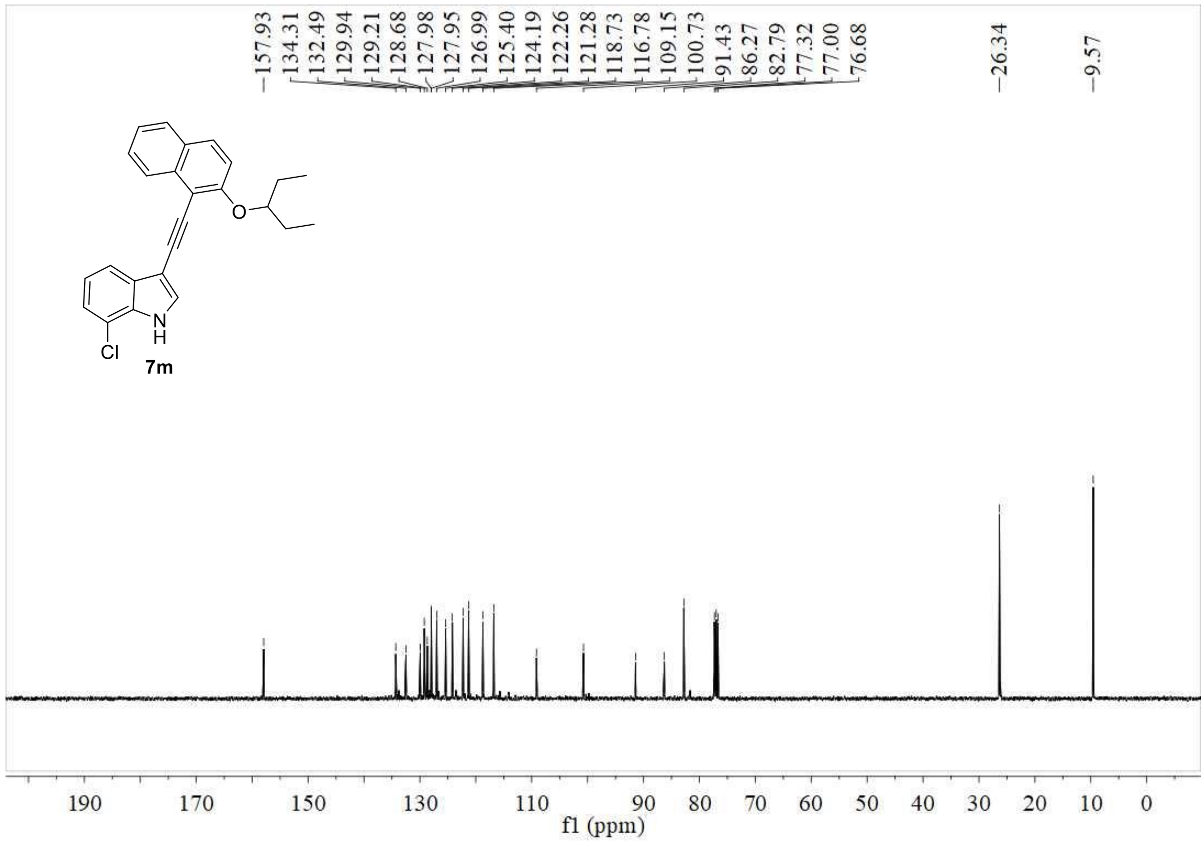
157.93
134.31
132.49
129.94
129.21
128.68
127.98
127.95
126.99
125.40
124.19
122.26
121.28
118.73
116.78
109.15
100.73
91.43
86.27
82.79
77.32
77.00
76.68
26.34
9.57
O
N
H
Cl
7m
190
170
150
130
110
90
80
70
60
50
40
30
20
10
0
f1 (ppm)

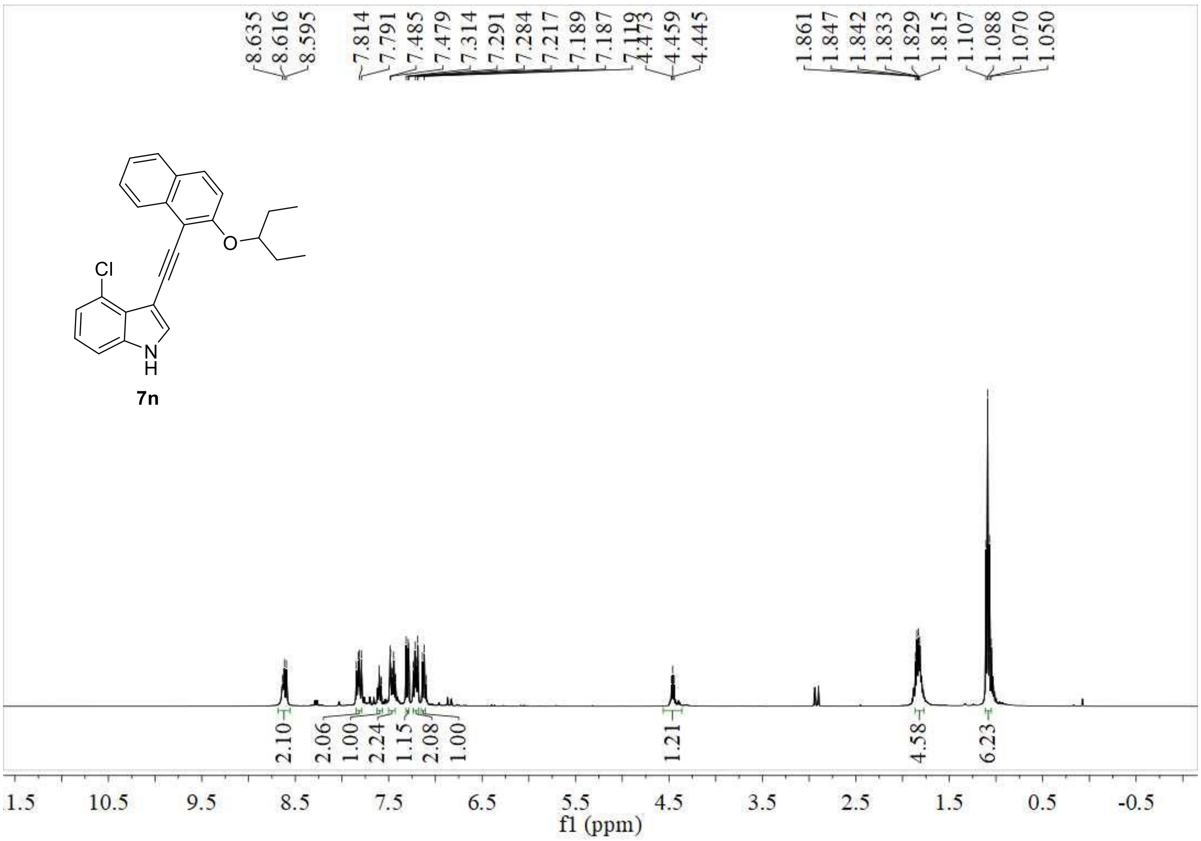
8.635
8.616
8.595
7.814
7.791
7.485
7.479
7.314
7.291
7.284
7.217
7.189
7.187
7.119
4.473
4.459
4.445
1.861
1.847
1.842
1.833
1.829
1.815
1.107
1.088
1.070
1.050
Cl
O
N
H
7n
2.10
2.06
1.00
2.24
1.15
2.08
1.00
1.21
4.58
6.23
f1 (ppm)

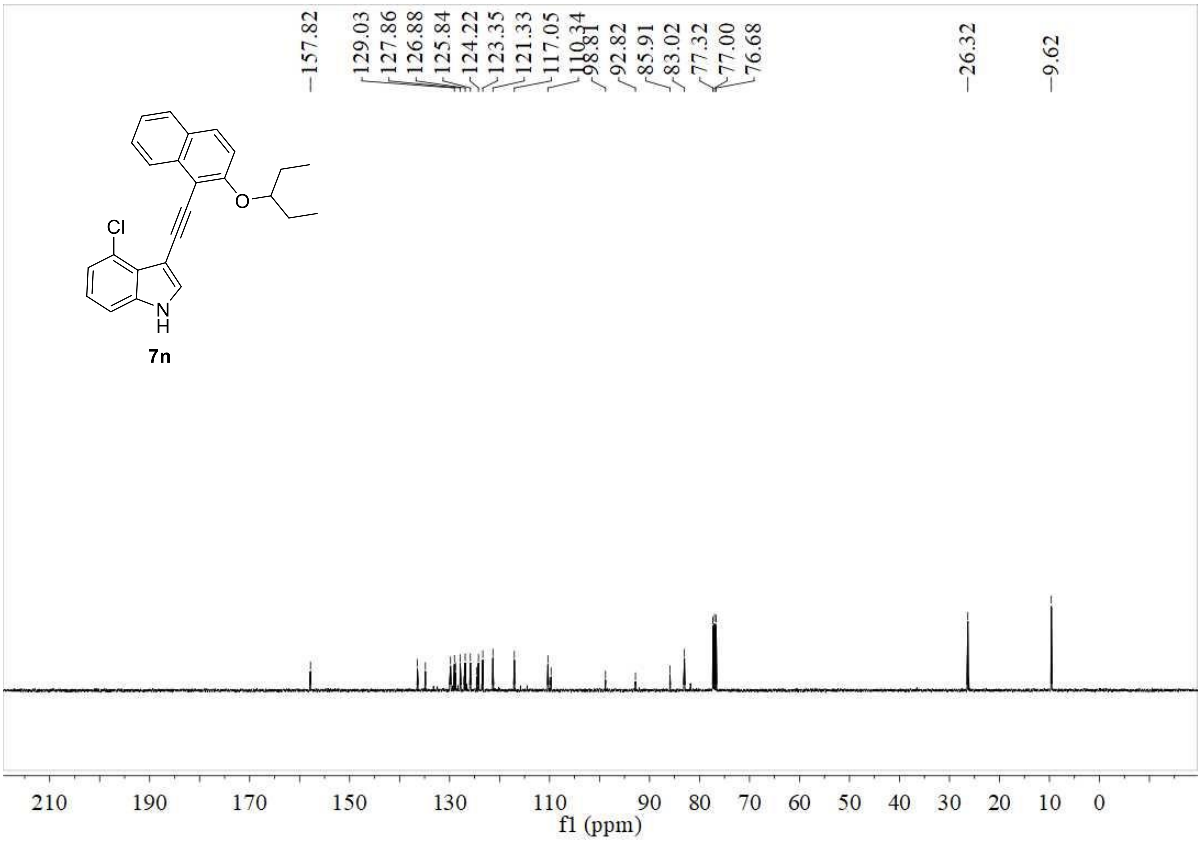
157.82
129.03
127.86
126.88
125.84
124.22
123.35
121.33
117.05
110.34
98.81
92.82
85.91
83.02
77.32
77.00
76.68
26.32
9.62
Cl
O
N
H
7n
f1 (ppm)

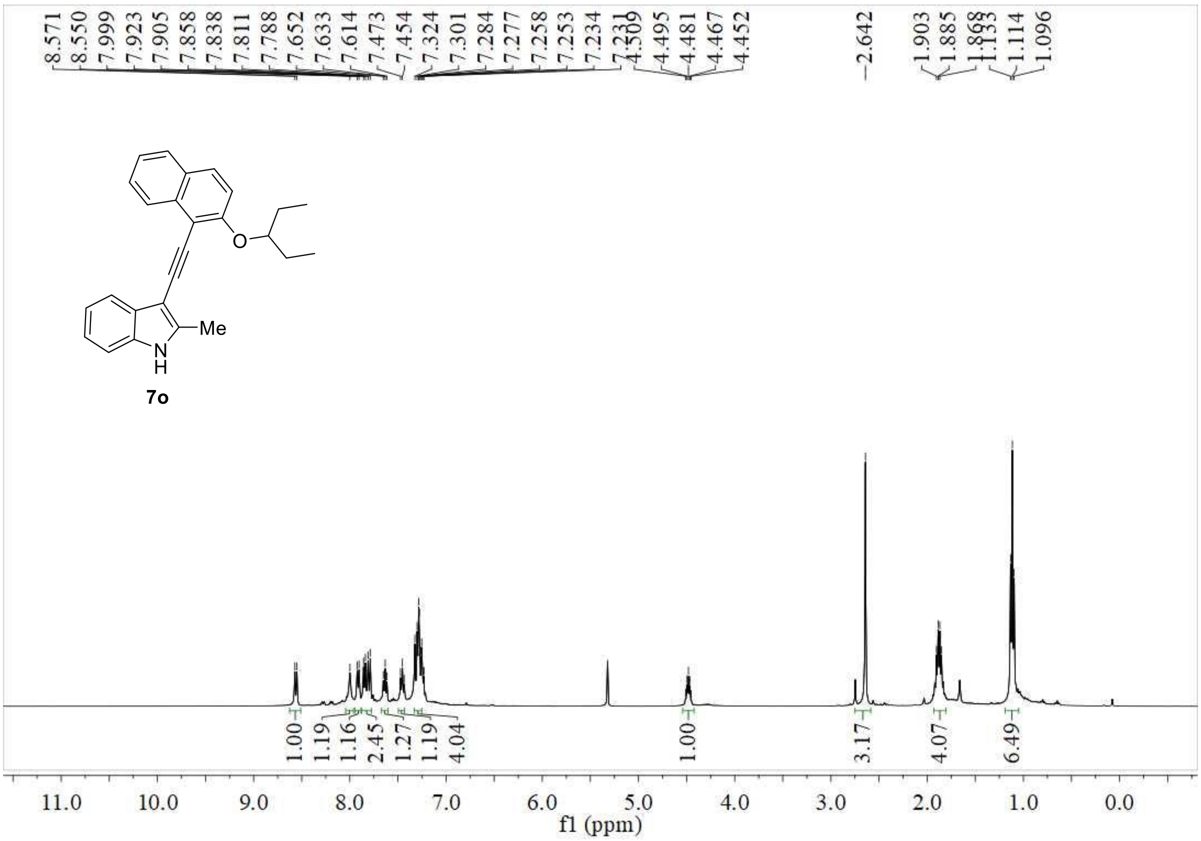

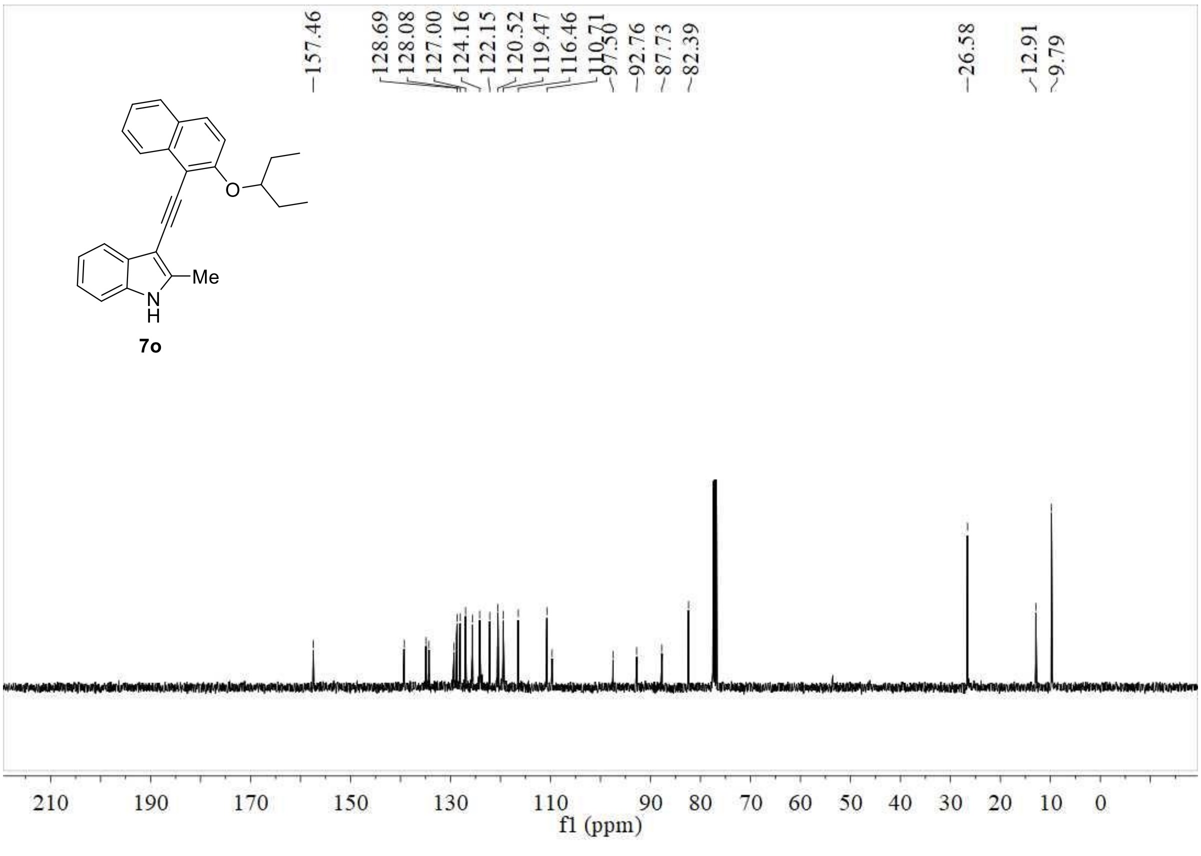

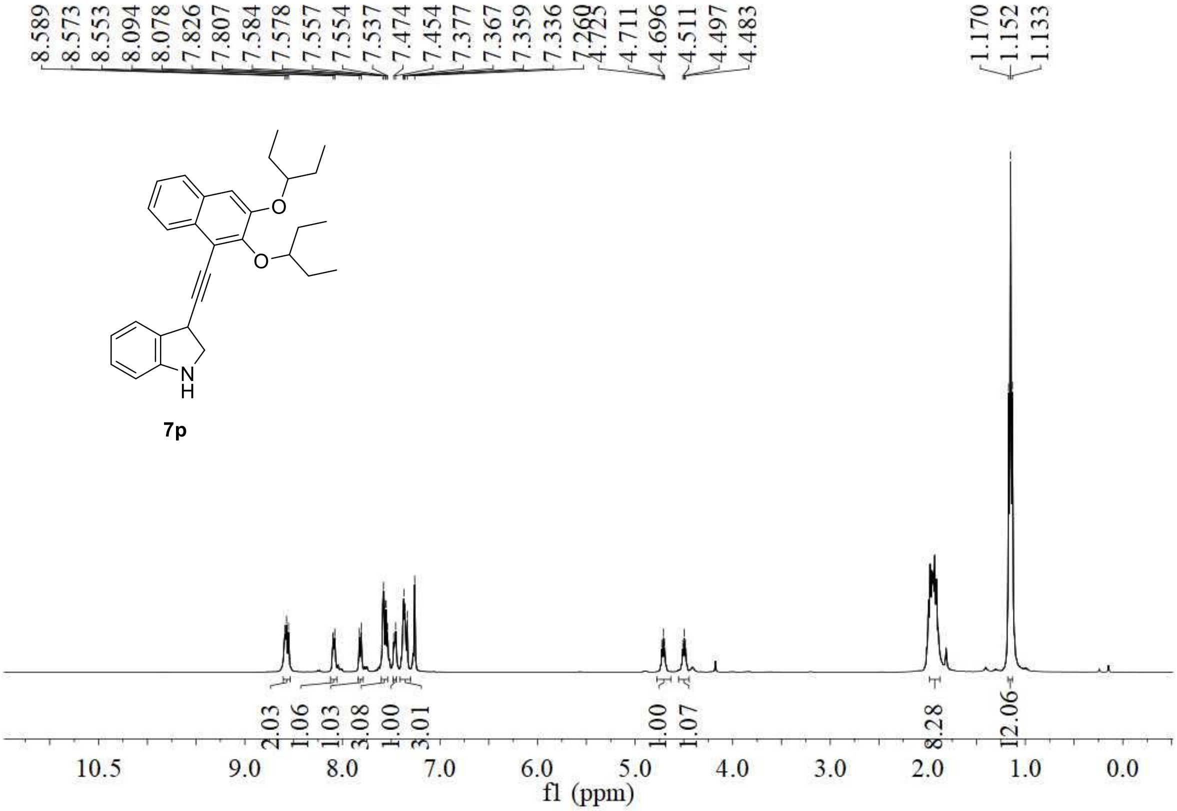
8.589
8.573
8.553
8.094
8.078
7.826
7.807
7.584
7.578
7.557
7.554
7.537
7.474
7.454
7.377
7.367
7.359
7.336
7.260
4.725
4.711
4.696
4.511
4.497
4.483
1.170
1.152
1.133
O
O
N
H
7p
2.03
1.06
1.03
3.08
1.00
3.01
1.00
1.07
8.28
12.06
10.5
9.0
8.0
7.0
6.0
5.0
4.0
3.0
2.0
1.0
0.0
f1 (ppm)

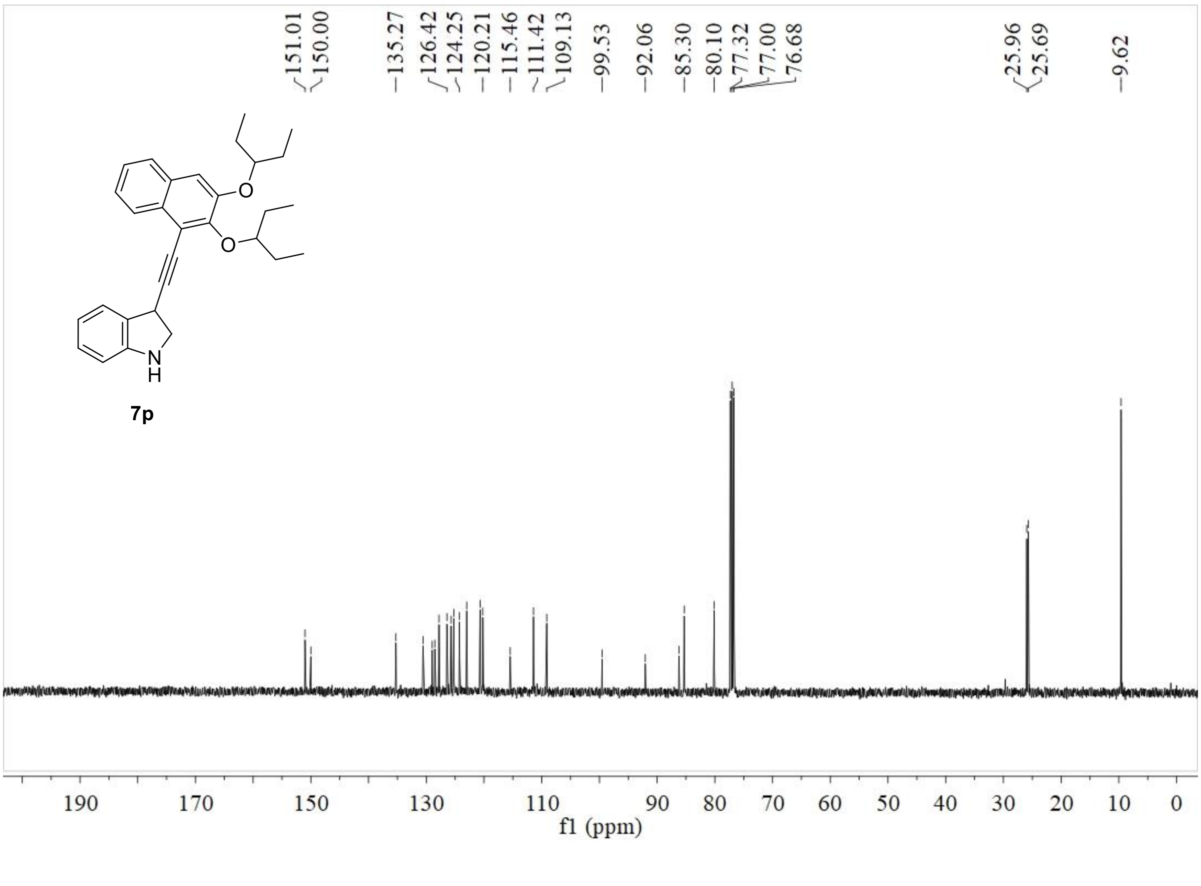
151.01
150.00
135.27
126.42
124.25
120.21
115.46
111.42
109.13
99.53
92.06
85.30
80.10
77.32
77.00
76.68
25.96
25.69
9.62
O
O
N
H
7p
190
170
150
130
110
90
80
70
60
50
40
30
20
10
0
f1 (ppm)

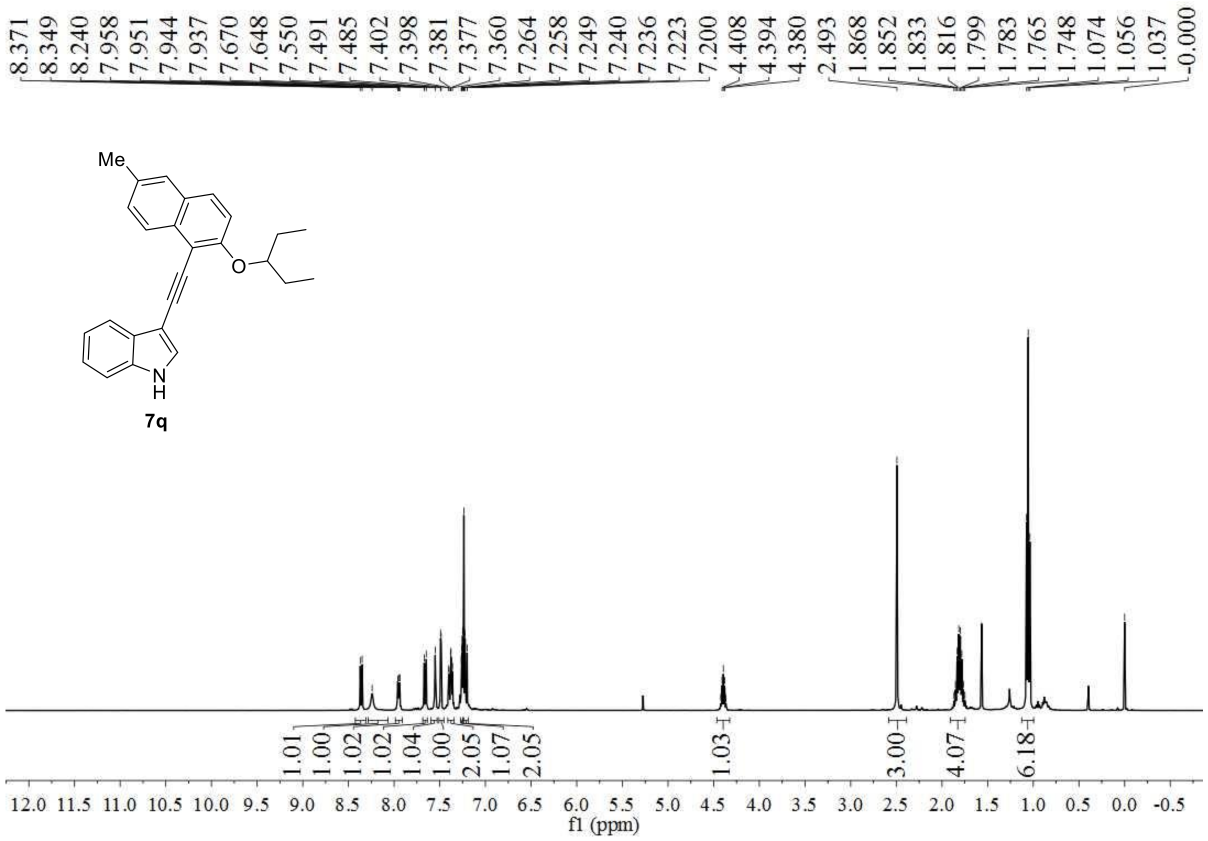
8.371 8.349 8.240 7.958 7.951 7.944 7.937 7.670 7.648 7.550 7.491 7.485 7.402 7.398 7.381 7.377 7.360 7.264 7.258 7.249 7.240 7.236 7.223 7.200 4.408 4.394 4.380 2.493 1.868 1.852 1.833 1.816 1.799 1.783 1.765 1.748 1.074 1.056 1.037 -0.000
Me
O
N
H
7q
1.01 1.00 1.02 1.02 1.04 1.00 2.05 1.07 2.05 1.03 3.00 4.07 6.18
12.0 11.5 11.0 10.5 10.0 9.5 9.0 8.5 8.0 7.5 7.0 6.5 6.0 5.5 5.0 4.5 4.0 3.5 3.0 2.5 2.0 1.5 1.0 0.5 0.0 -0.5
f1 (ppm)

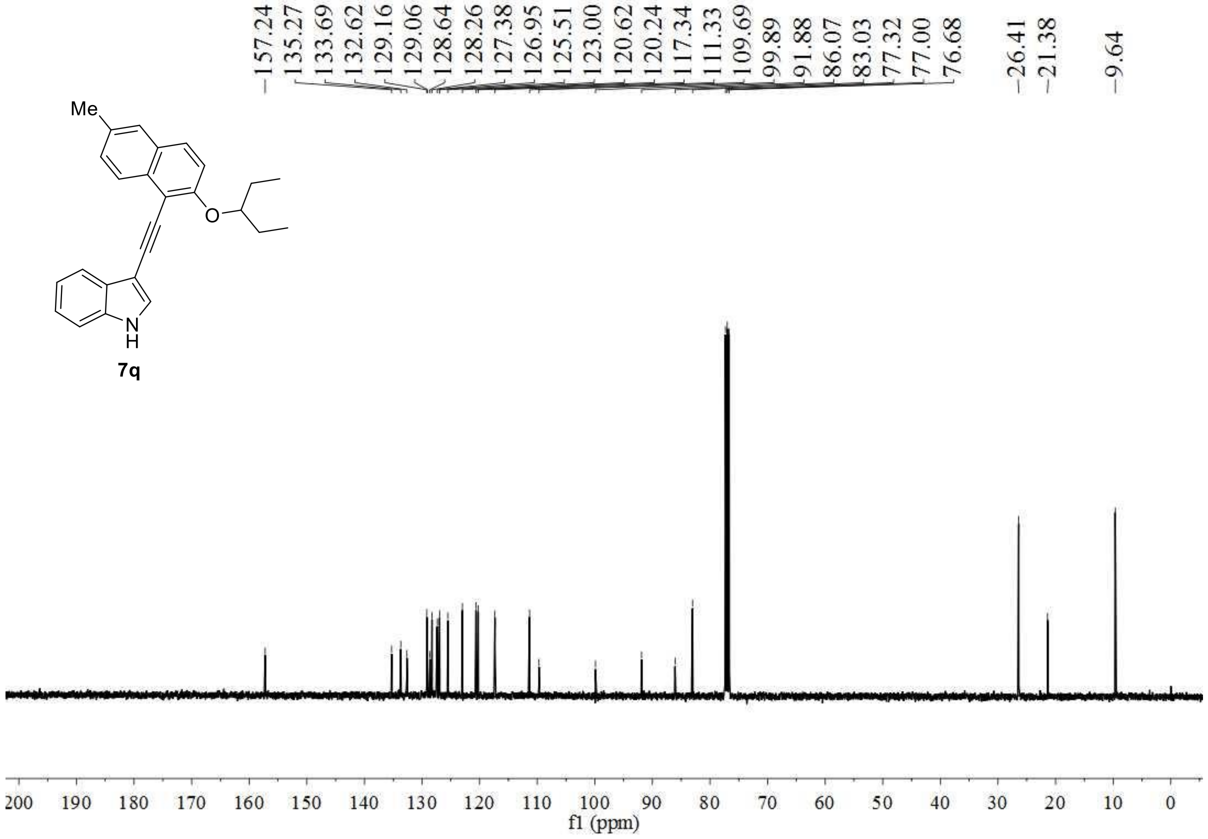
157.24 135.27 133.69 132.62 129.16 129.06 128.64 128.26 127.38 126.95 125.51 123.00 120.62 120.24 117.34 111.33 109.69 99.89 91.88 86.07 83.03 77.32 77.00 76.68 26.41 21.38 9.64
Me
O
N
H
7q
200 190 180 170 160 150 140 130 120 110 100 90 80 70 60 50 40 30 20 10 0
f1 (ppm)

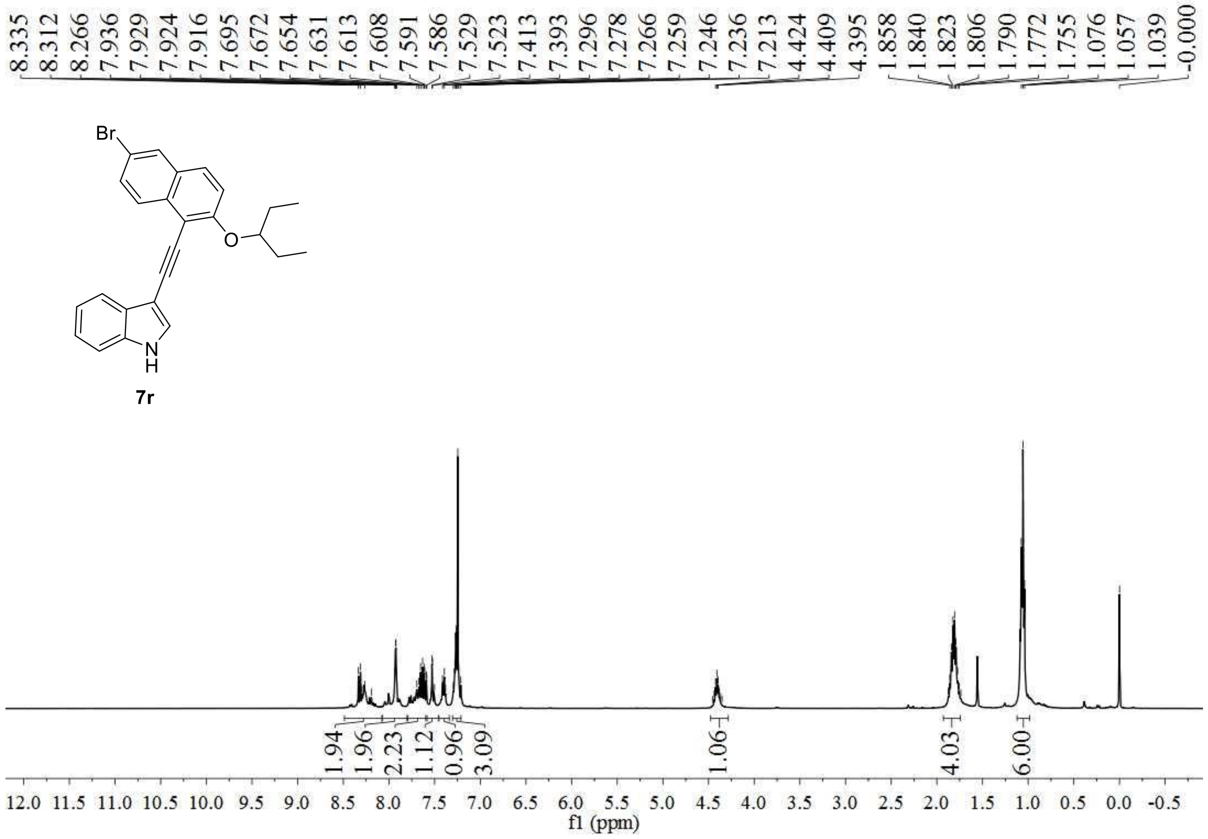
8.335 8.312 8.266 7.936 7.929 7.924 7.916 7.695 7.672 7.654 7.631 7.613 7.608 7.591 7.586 7.529 7.523 7.413 7.393 7.296 7.278 7.266 7.259 7.246 7.236 7.213 4.424 4.409 4.395 1.858 1.840 1.823 1.806 1.790 1.772 1.755 1.076 1.057 1.039 -0.000
Br
O
N
H
7r
1.94 1.96 2.23 1.12 0.96 3.09 1.06 4.03 6.00
12.0 11.5 11.0 10.5 10.0 9.5 9.0 8.5 8.0 7.5 7.0 6.5 6.0 5.5 5.0 4.5 4.0 3.5 3.0 2.5 2.0 1.5 1.0 0.5 0.0 -0.5
f1 (ppm)

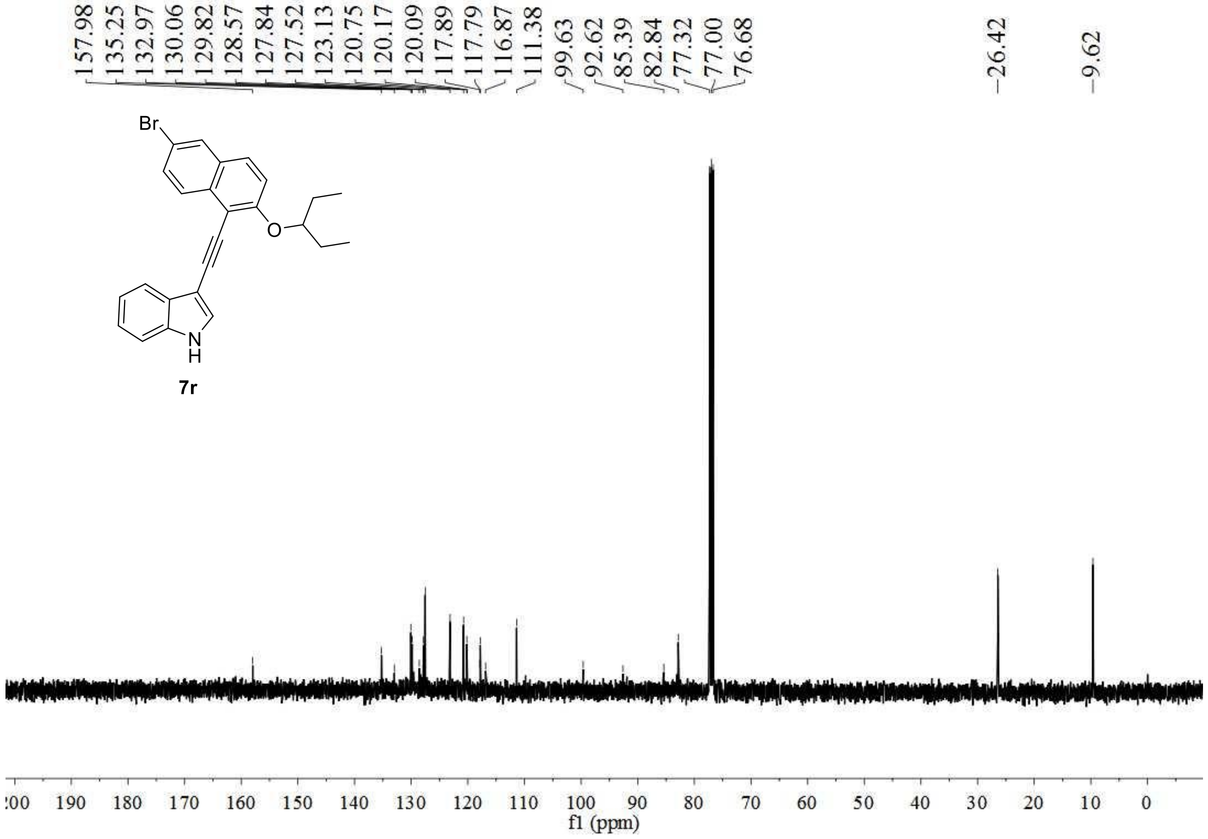
157.98 135.25 132.97 130.06 129.82 128.57 127.84 127.52 123.13 120.75 120.17 120.09 117.89 117.79 116.87 111.38 99.63 92.62 85.39 82.84 77.32 77.00 76.68 26.42 9.62
Br
O
N
H
7r
200 190 180 170 160 150 140 130 120 110 100 90 80 70 60 50 40 30 20 10 0
f1 (ppm)

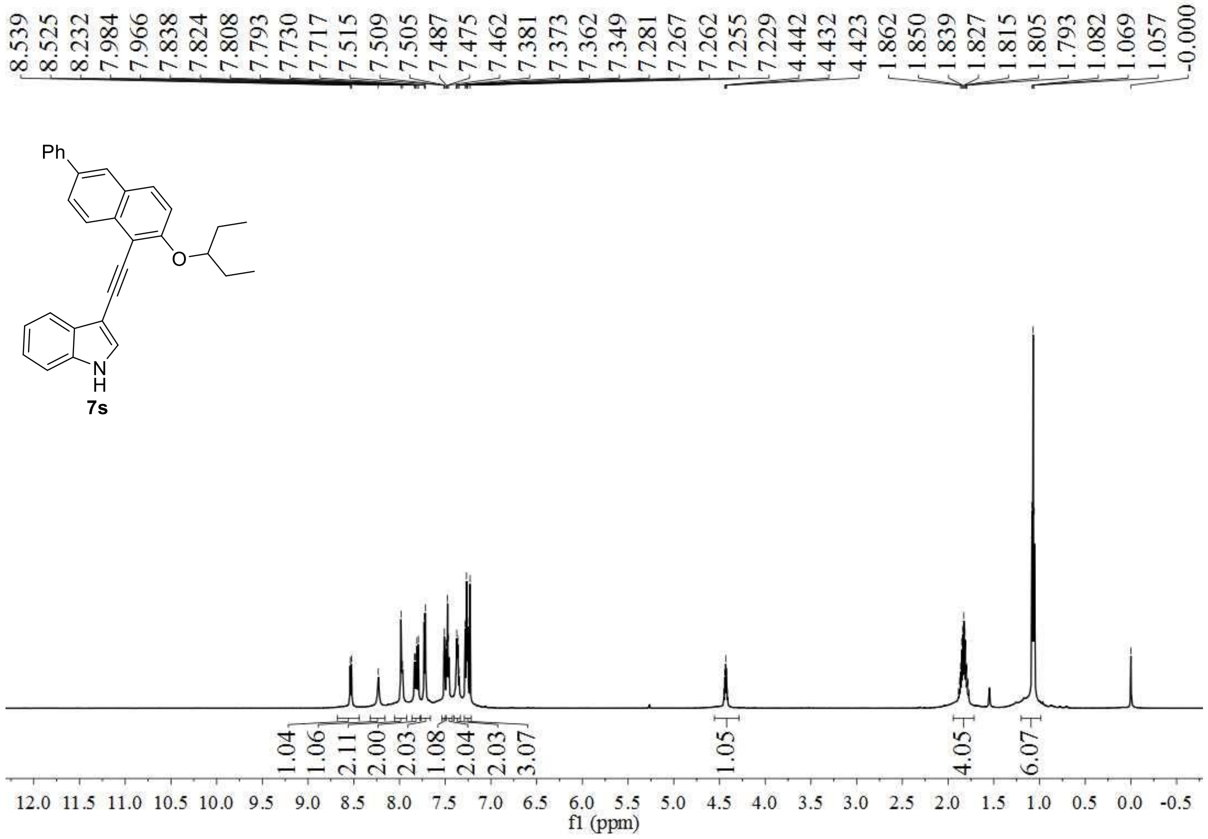
8.539 8.525 8.232 7.984 7.966 7.838 7.824 7.808 7.793 7.730 7.717 7.515 7.509 7.505 7.487 7.475 7.462 7.381 7.373 7.362 7.349 7.281 7.267 7.262 7.255 7.229 4.442 4.432 4.423 1.862 1.850 1.839 1.827 1.815 1.805 1.793 1.082 1.069 1.057 -0.000
Ph
O
N
H
7s
1.04 1.06 2.11 2.00 2.03 1.08 2.04 2.03 3.07 1.05 4.05 6.07
12.0 11.5 11.0 10.5 10.0 9.5 9.0 8.5 8.0 7.5 7.0 6.5 6.0 5.5 5.0 4.5 4.0 3.5 3.0 2.5 2.0 1.5 1.0 0.5 0.0 -0.5
f1 (ppm)

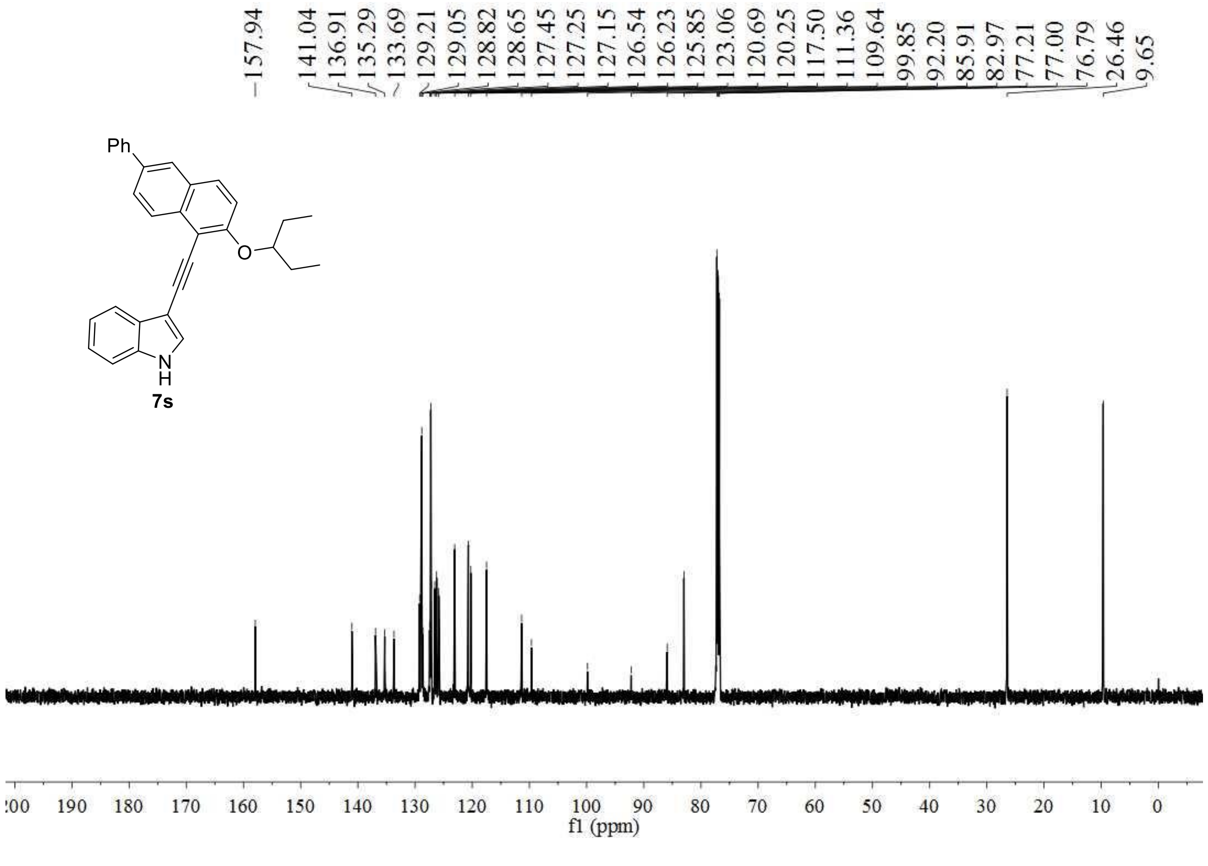
157.94 141.04 136.91 135.29 133.69 129.21 129.05 128.82 128.65 127.45 127.25 127.15 126.54 126.23 125.85 123.06 120.69 120.25 117.50 111.36 109.64 99.85 92.20 85.91 82.97 77.21 77.00 76.79 26.46 9.65
Ph
O
N
H
7s
200 190 180 170 160 150 140 130 120 110 100 90 80 70 60 50 40 30 20 10 0
f1 (ppm)

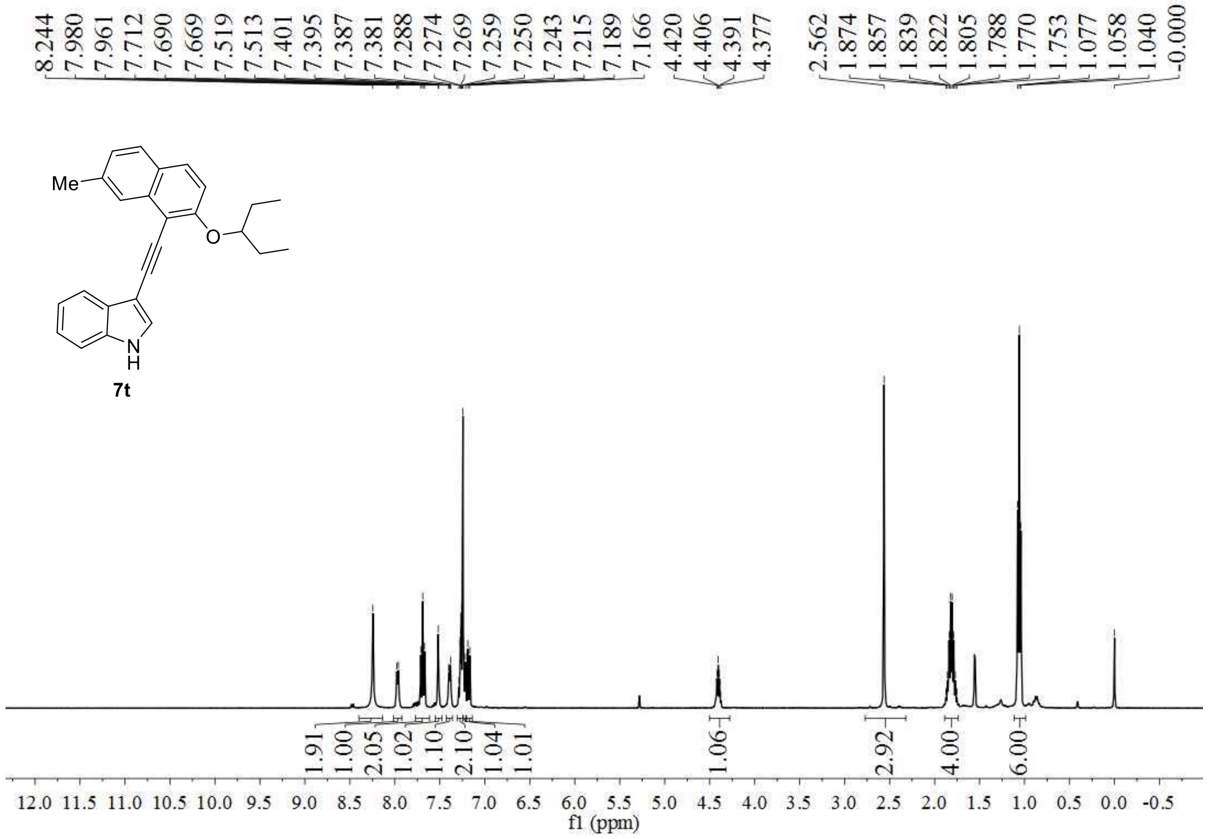
8.244 7.980 7.961 7.712 7.690 7.669 7.519 7.513 7.401 7.395 7.387 7.381 7.288 7.274 7.269 7.259 7.250 7.243 7.215 7.189 7.166 4.420 4.406 4.391 4.377 2.562 1.874 1.857 1.839 1.822 1.805 1.788 1.770 1.753 1.077 1.058 1.040 -0.000
Me
O
N
H
7t
1.91 1.00 2.05 1.02 1.10 2.10 1.04 1.01 1.06 2.92 4.00 6.00
12.0 11.5 11.0 10.5 10.0 9.5 9.0 8.5 8.0 7.5 7.0 6.5 6.0 5.5 5.0 4.5 4.0 3.5 3.0 2.5 2.0 1.5 1.0 0.5 0.0 -0.5
f1 (ppm)

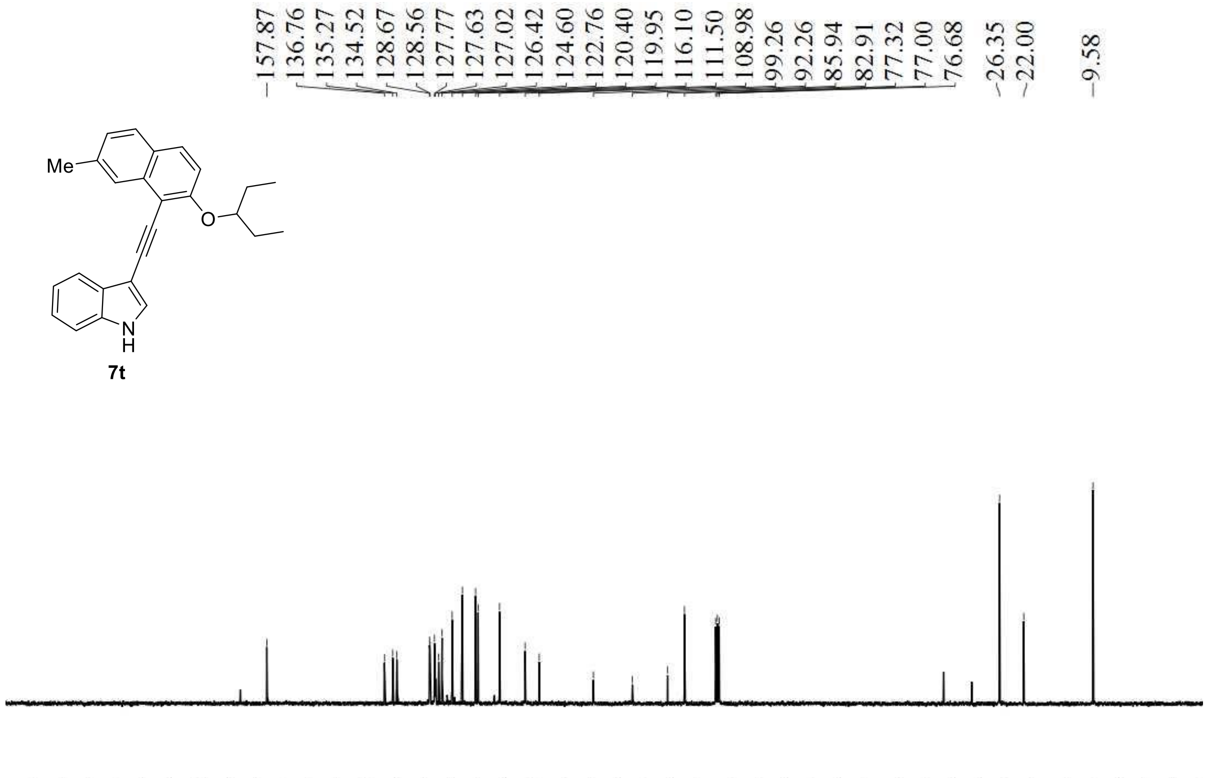
157.87 136.76 135.27 134.52 128.67 128.56 127.77 127.63 127.02 126.42 124.60 122.76 120.40 119.95 116.10 111.50 108.98 99.26 92.26 85.94 82.91 77.32 77.00 76.68 26.35 22.00 9.58
Me
O
N
H
7t
200 190 180 170 160 150 140 130 120 110 100 90 80 70 60 50 40 30 20 10 0 -1
f1 (ppm)

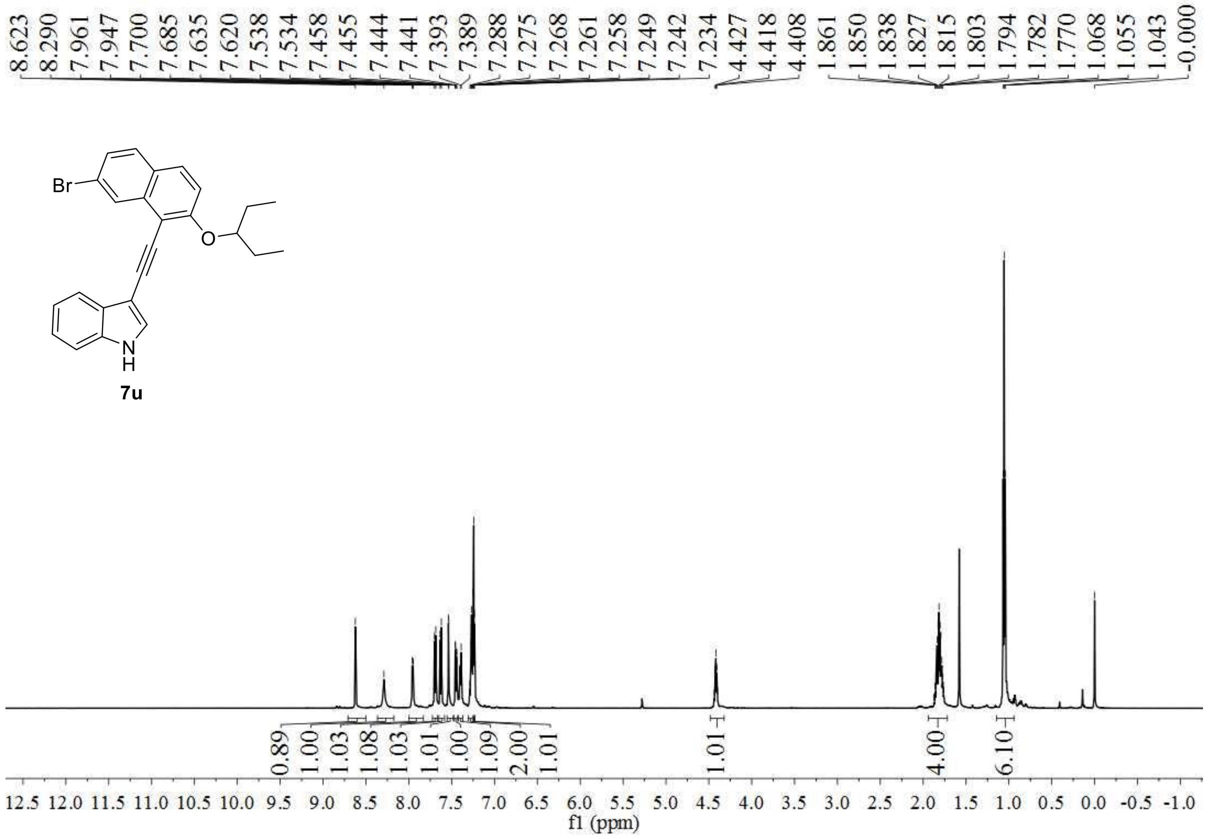
8.623
8.290
7.961
7.947
7.700
7.685
7.635
7.620
7.538
7.534
7.458
7.455
7.444
7.441
7.393
7.389
7.288
7.275
7.268
7.261
7.258
7.249
7.242
7.234
4.427
4.418
4.408
1.861
1.850
1.838
1.827
1.815
1.803
1.794
1.782
1.770
1.068
1.055
1.043
-0.000
Br
O
N
H
7u
0.89
1.00
1.03
1.08
1.03
1.01
1.00
1.09
2.00
1.01
1.01
4.00
6.10
12.5 12.0 11.5 11.0 10.5 10.0 9.5 9.0 8.5 8.0 7.5 7.0 6.5 6.0 5.5 5.0 4.5 4.0 3.5 3.0 2.5 2.0 1.5 1.0 0.5 0.0 -0.5 -1.0
f1 (ppm)

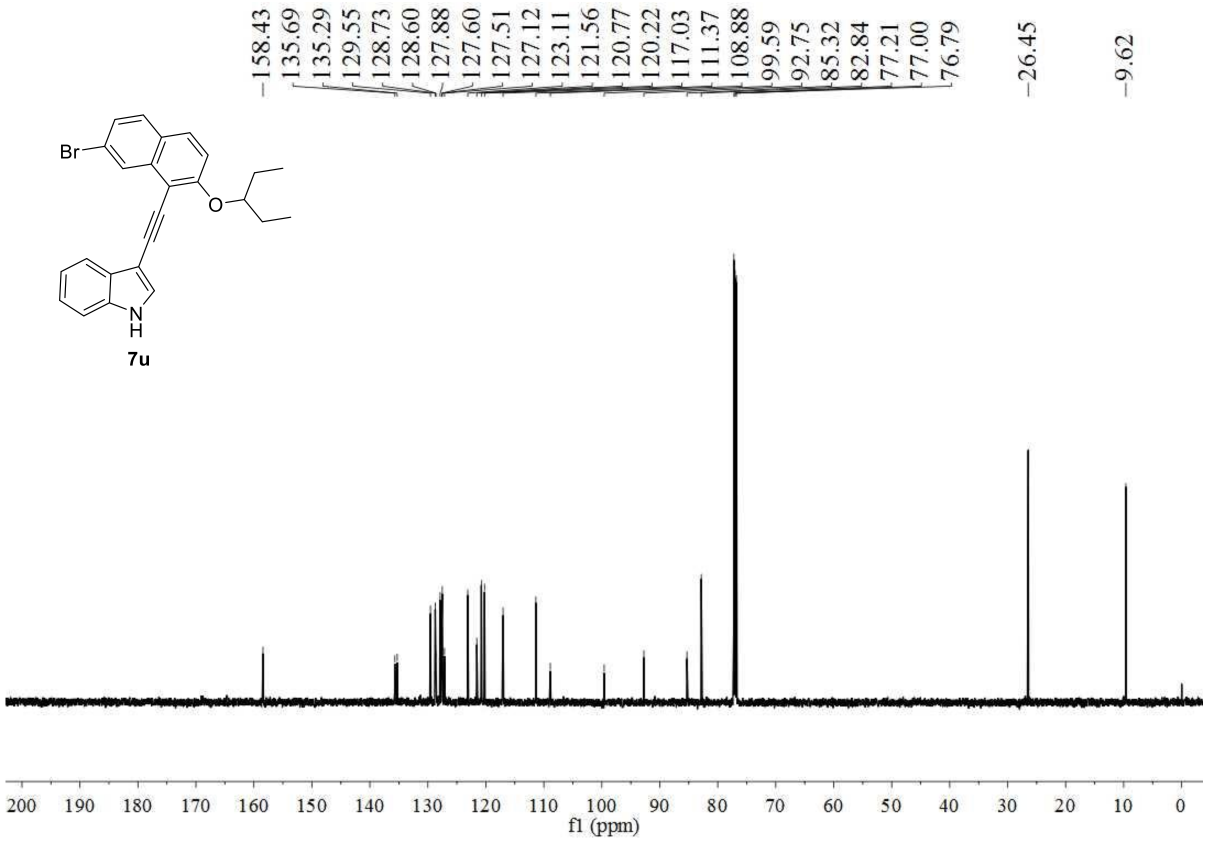
158.43
135.69
135.29
129.55
128.73
128.60
127.88
127.60
127.51
127.12
123.11
121.56
120.77
120.22
117.03
111.37
108.88
99.59
92.75
85.32
82.84
77.21
77.00
76.79
26.45
9.62
Br
O
N
H
7u
200 190 180 170 160 150 140 130 120 110 100 90 80 70 60 50 40 30 20 10 0
f1 (ppm)

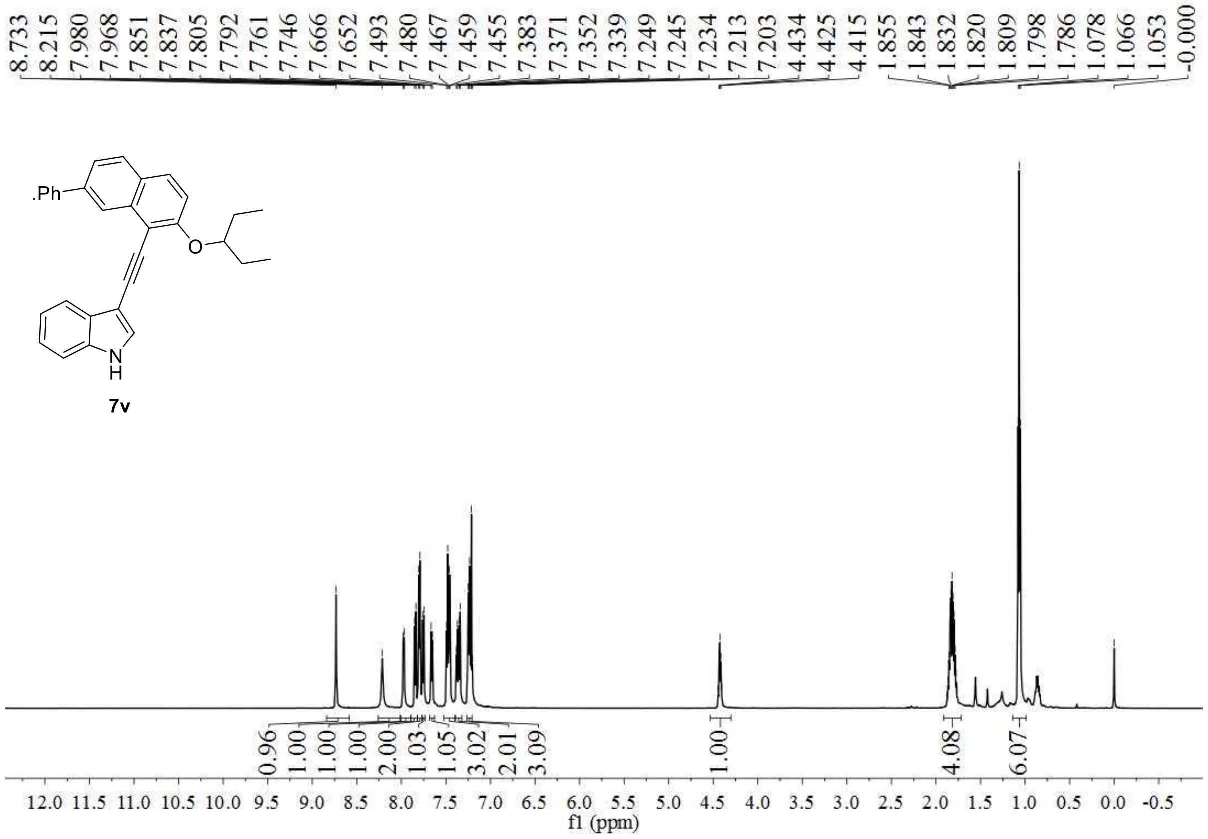
8.733 8.215 7.980 7.968 7.851 7.837 7.805 7.792 7.761 7.746 7.666 7.652 7.493 7.480 7.467 7.459 7.455 7.383 7.371 7.352 7.339 7.249 7.245 7.234 7.213 7.203 4.434 4.425 4.415 1.855 1.843 1.832 1.820 1.809 1.798 1.786 1.078 1.066 1.053 -0.000
.Ph
O
N
H
7v
0.96 1.00 1.00 1.00 2.00 1.03 1.05 3.02 2.01 3.09
1.00
4.08
6.07
12.0 11.5 11.0 10.5 10.0 9.5 9.0 8.5 8.0 7.5 7.0 6.5 6.0 5.5 5.0 4.5 4.0 3.5 3.0 2.5 2.0 1.5 1.0 0.5 0.0 -0.5
f1 (ppm)

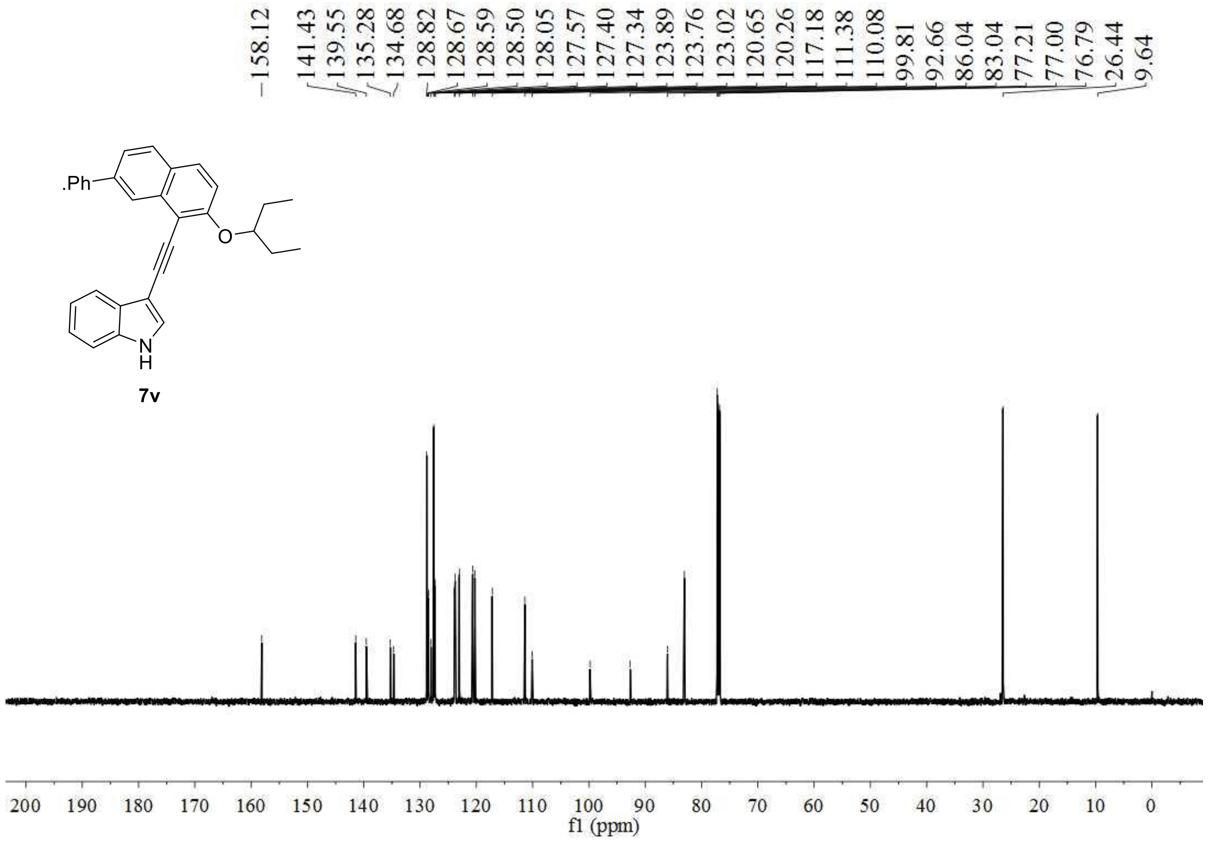
158.12 141.43 139.55 135.28 134.68 128.82 128.67 128.59 128.50 128.05 127.57 127.40 127.34 123.89 123.76 123.02 120.65 120.26 117.18 111.38 110.08 99.81 92.66 86.04 83.04 77.21 77.00 76.79 26.44 9.64
.Ph
O
N
H
7v
200 190 180 170 160 150 140 130 120 110 100 90 80 70 60 50 40 30 20 10 0
f1 (ppm)

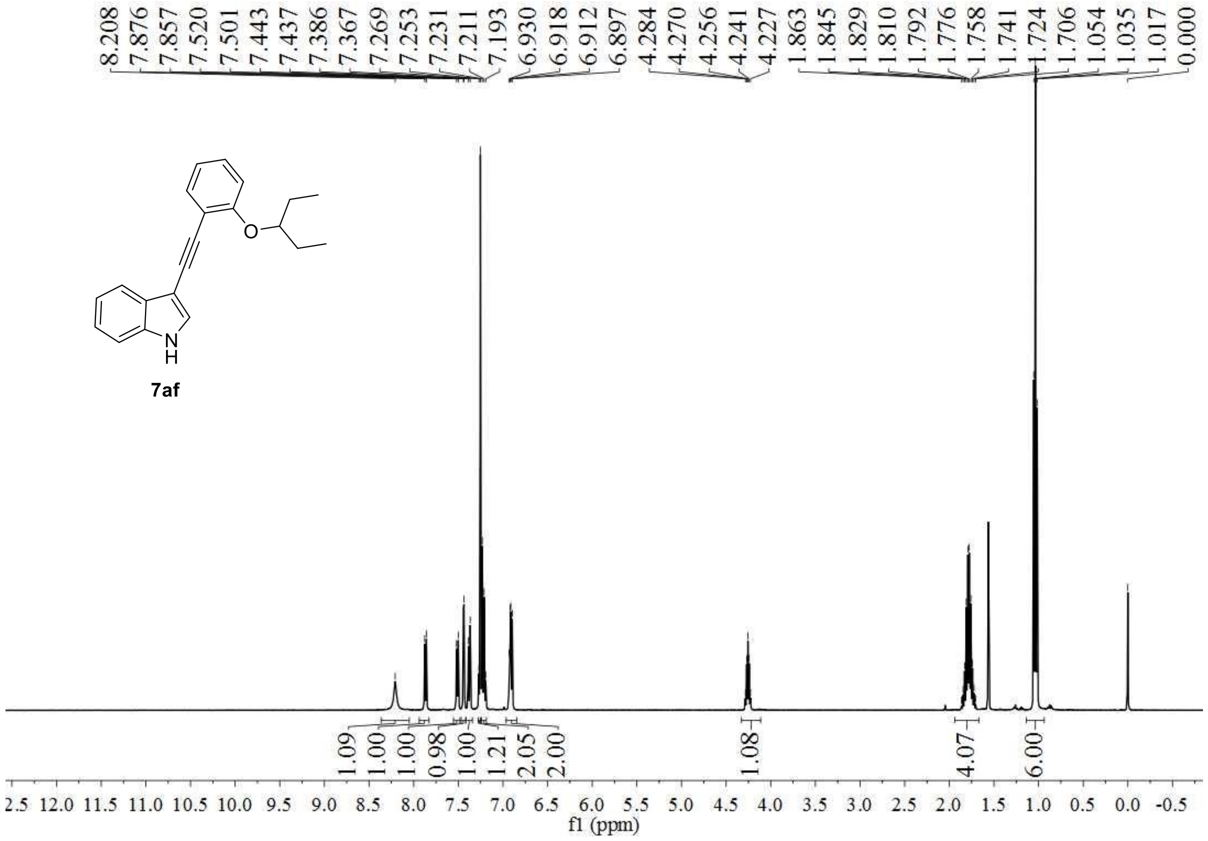
8.208 7.876 7.857 7.520 7.501 7.443 7.437 7.386 7.367 7.269 7.253 7.231 7.211 7.193 6.930 6.918 6.912 6.897 4.284 4.270 4.256 4.241 4.227 1.863 1.845 1.829 1.810 1.792 1.776 1.758 1.741 1.724 1.706 1.054 1.035 1.017 0.000
O
N
H
7af
1.09 1.00 1.00 0.98 1.00 1.21 2.05 2.00 1.08 4.07 6.00
2.5 12.0 11.5 11.0 10.5 10.0 9.5 9.0 8.5 8.0 7.5 7.0 6.5 6.0 5.5 5.0 4.5 4.0 3.5 3.0 2.5 2.0 1.5 1.0 0.5 0.0 -0.5
f1 (ppm)

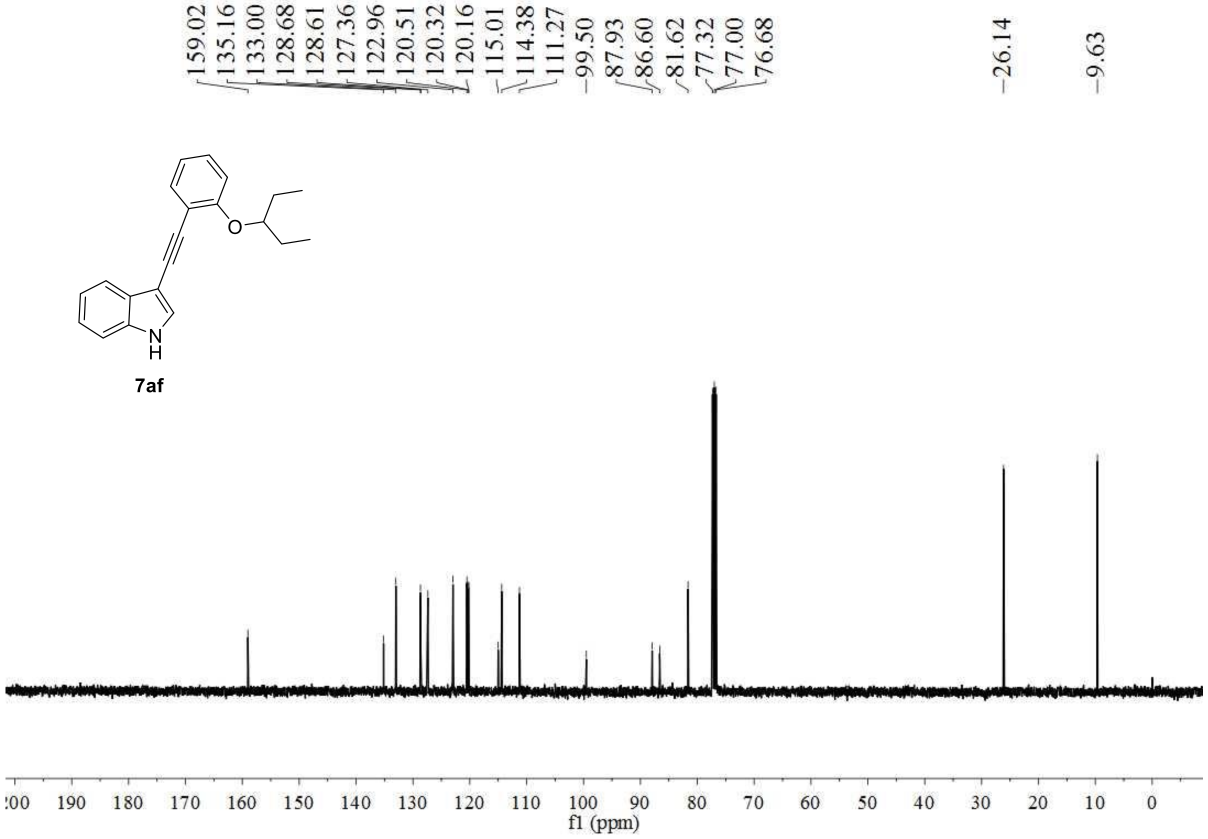
159.02 135.16 133.00 128.68 128.61 127.36 122.96 120.51 120.32 120.16 115.01 114.38 111.27 99.50 87.93 86.60 81.62 77.32 77.00 76.68 26.14 9.63
O
N
H
7af
200 190 180 170 160 150 140 130 120 110 100 90 80 70 60 50 40 30 20 10 0
f1 (ppm)

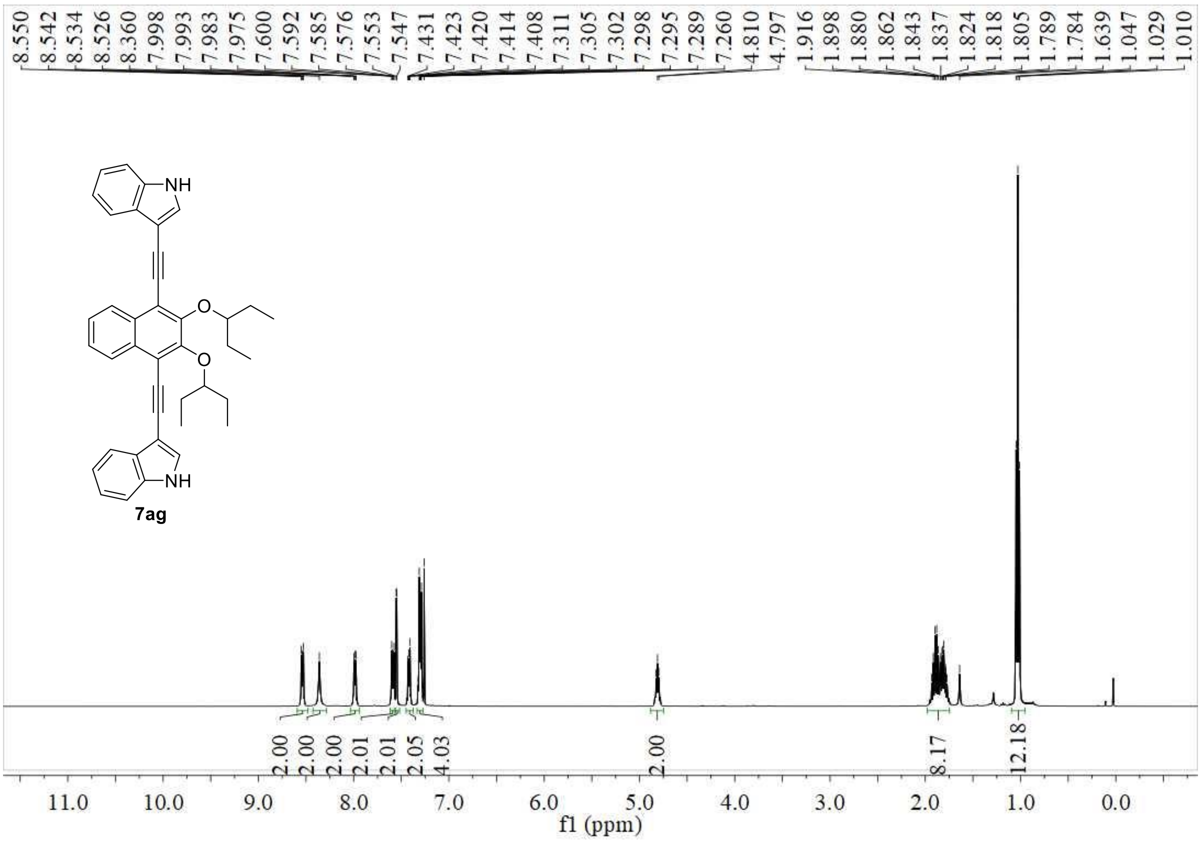
8.550 8.542 8.534 8.526 8.360 7.998 7.993 7.983 7.975 7.600 7.592 7.585 7.576 7.553 7.547 7.431 7.423 7.420 7.414 7.408 7.311 7.305 7.302 7.298 7.295 7.289 7.260 4.810 4.797 1.916 1.898 1.880 1.862 1.843 1.837 1.824 1.818 1.805 1.789 1.784 1.639 1.047 1.029 1.010
NH
O
O
NH
7ag
2.00 2.00 2.00 2.01 2.01 2.05 4.03
2.00
8.17
12.18
11.0 10.0 9.0 8.0 7.0 6.0 5.0 4.0 3.0 2.0 1.0 0.0
f1 (ppm)

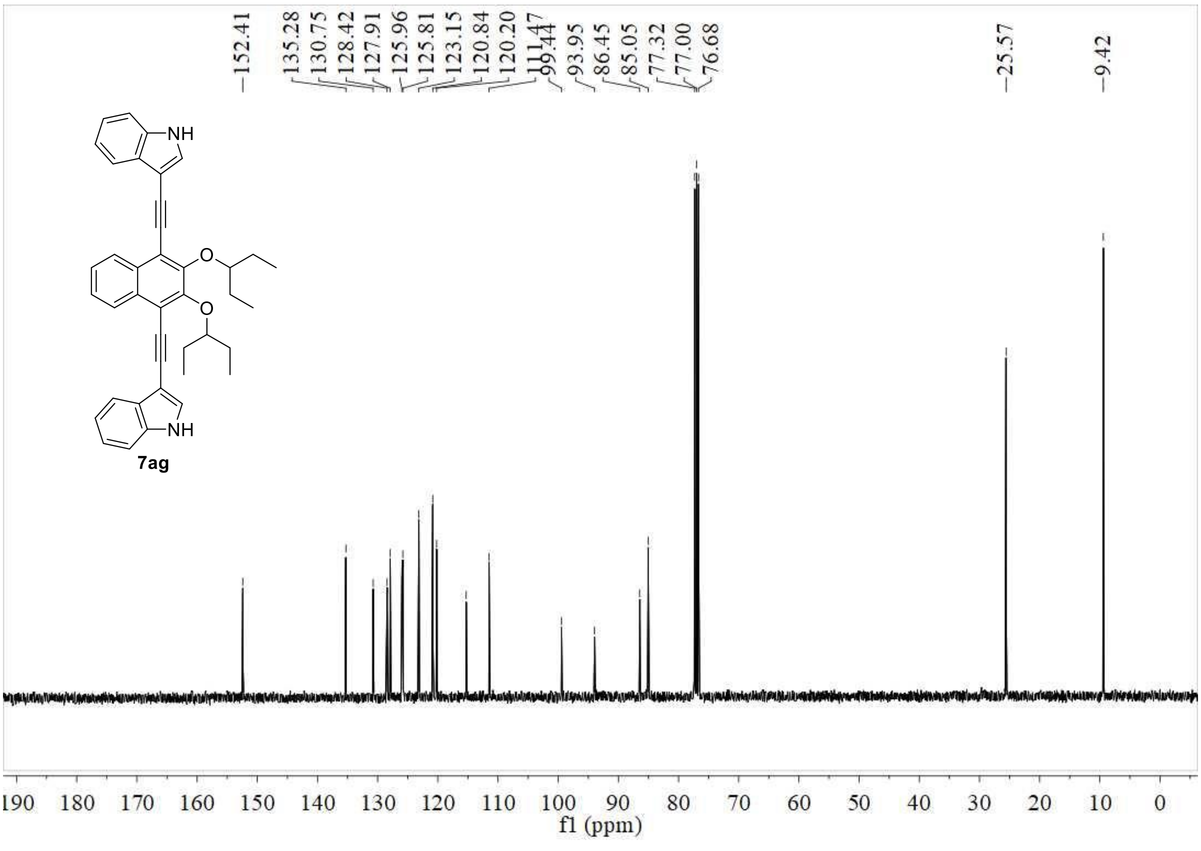
152.41
135.28 130.75 128.42 127.91 125.96 125.81 123.15 120.84 120.20 111.47 99.44 93.95 86.45 85.05 77.32 77.00 76.68
25.57
9.42
NH
O
O
NH
7ag
190 180 170 160 150 140 130 120 110 100 90 80 70 60 50 40 30 20 10 0
f1 (ppm)

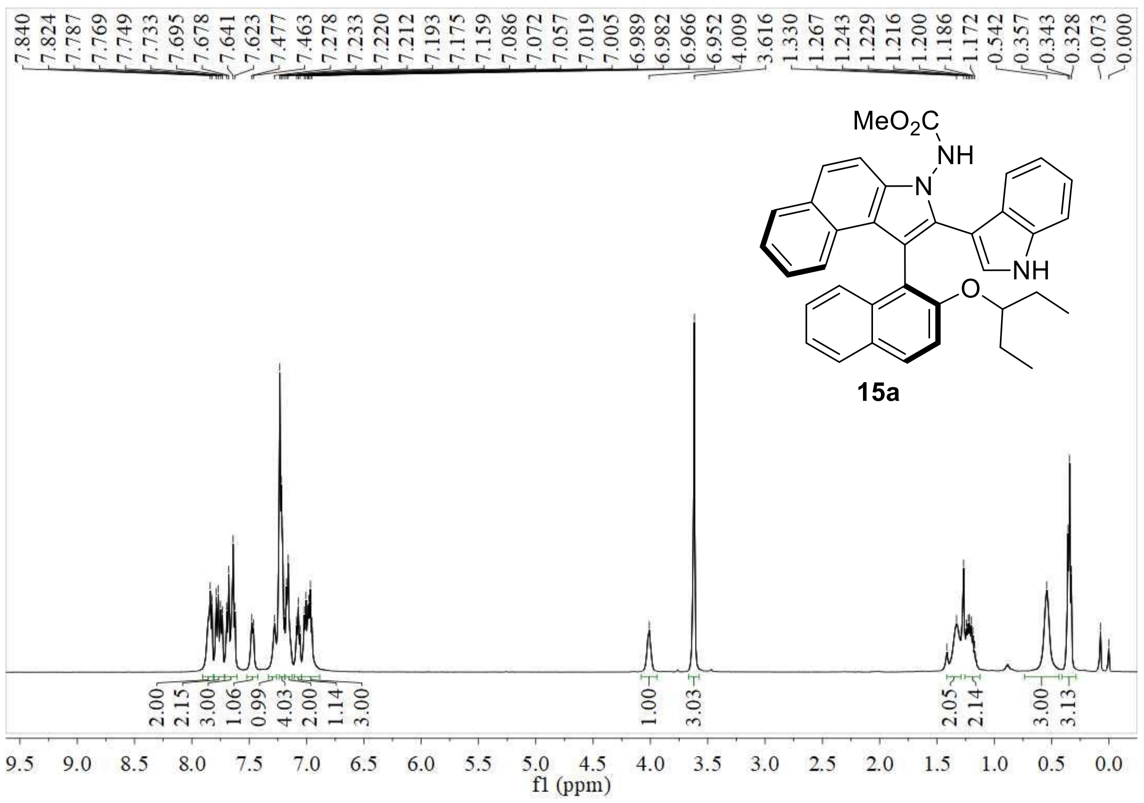

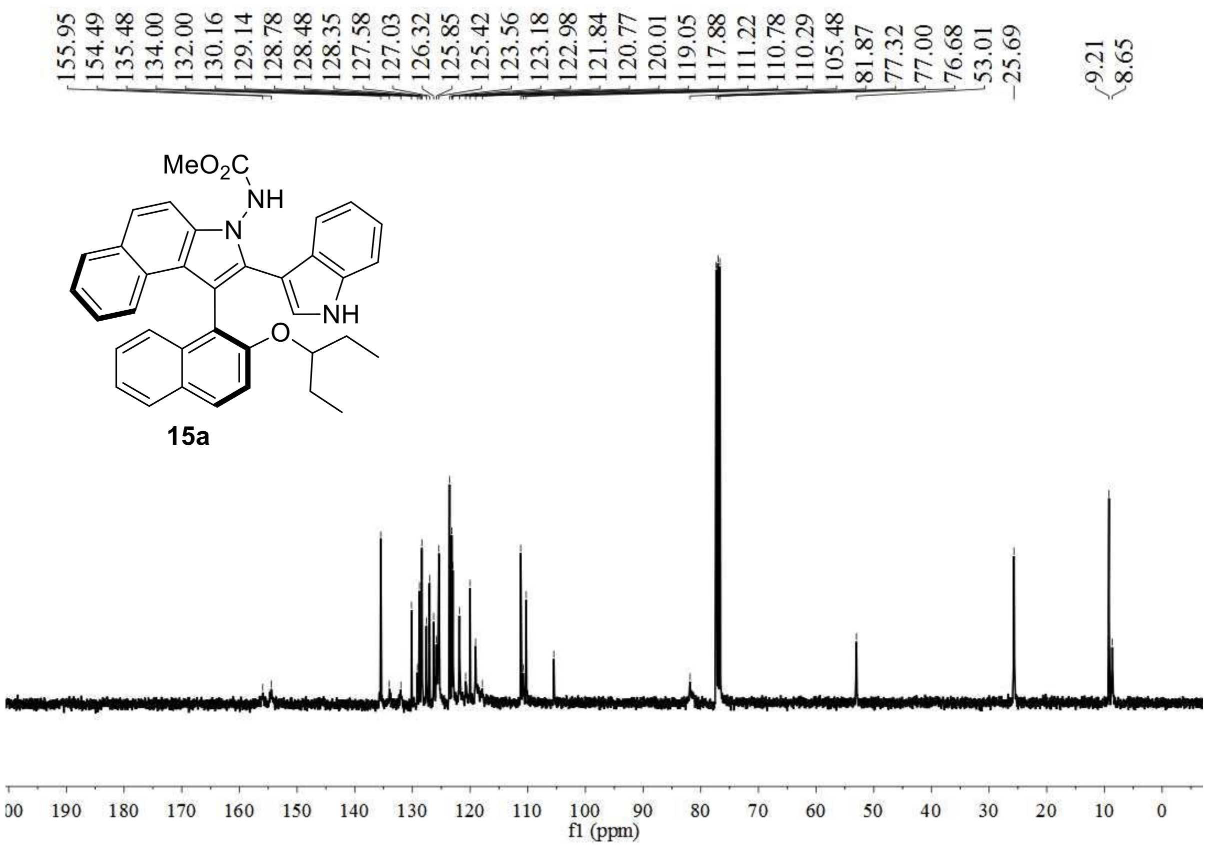

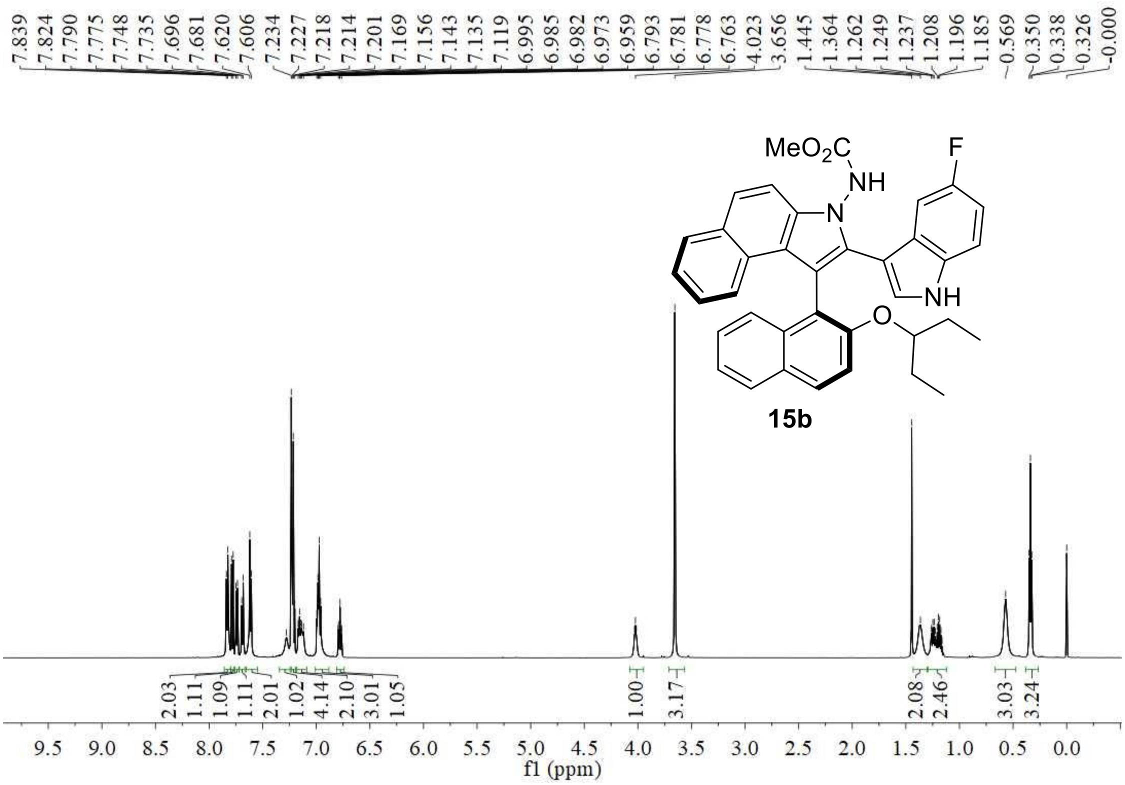
7.839 7.824 7.790 7.775 7.748 7.735 7.696 7.681 7.620 7.606 7.234 7.227 7.218 7.214 7.201 7.169 7.156 7.143 7.135 7.119 6.995 6.985 6.982 6.973 6.959 6.793 6.781 6.778 6.763 4.023 3.656 1.445 1.364 1.262 1.249 1.237 1.208 1.196 1.185 0.569 0.350 0.338 0.326 -0.000
MeO2C
NH
N
F
NH
O
15b
2.03 1.11 1.09 1.11 2.01 1.02 4.14 2.10 3.01 1.05 1.00 3.17 2.08 2.46 3.03 3.24
9.5 9.0 8.5 8.0 7.5 7.0 6.5 6.0 5.5 5.0 4.5 4.0 3.5 3.0 2.5 2.0 1.5 1.0 0.5 0.0
f1 (ppm)

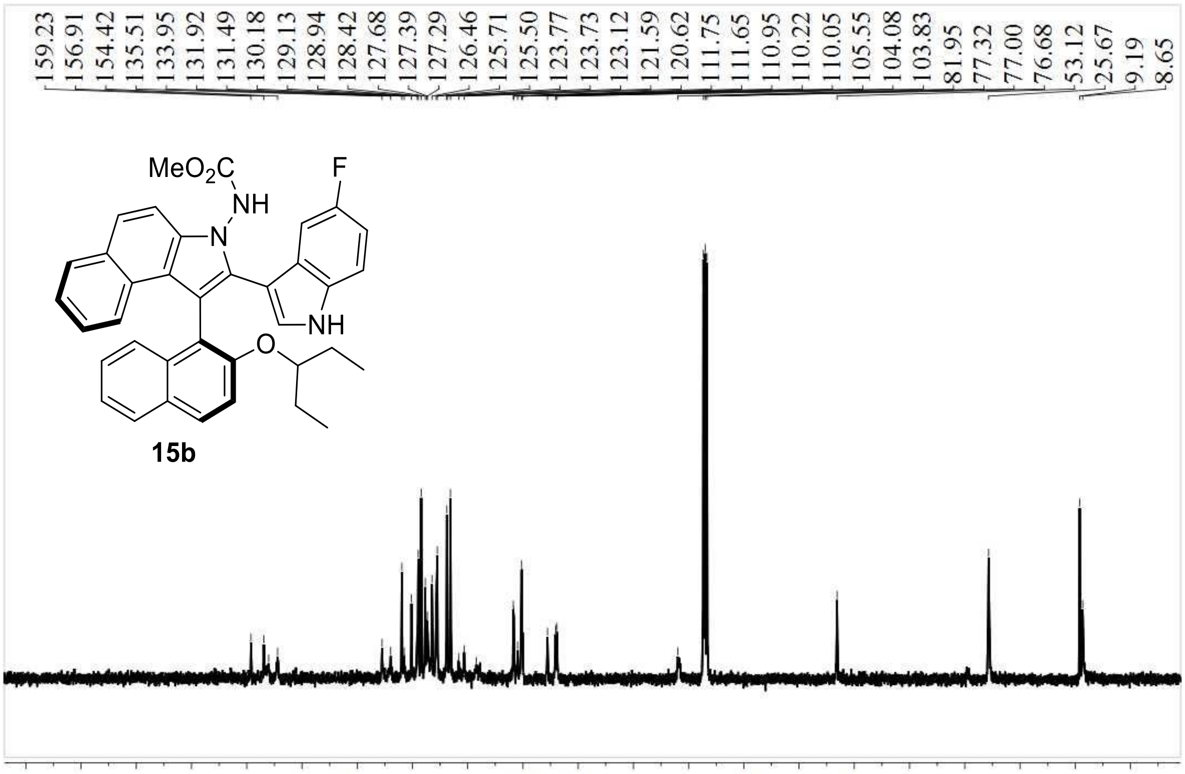
159.23 156.91 154.42 135.51 133.95 131.92 131.49 130.18 129.13 128.94 128.42 127.68 127.39 127.29 126.46 125.71 125.50 123.77 123.73 123.12 121.59 120.62 111.75 111.65 110.95 110.22 110.05 105.55 104.08 103.83 81.95 77.32 77.00 76.68 53.12 25.67 9.19 8.65
MeO2C
NH
N
F
NH
O
15b
200 190 180 170 160 150 140 130 120 110 100 90 80 70 60 50 40 30 20 10 0
f1 (ppm)

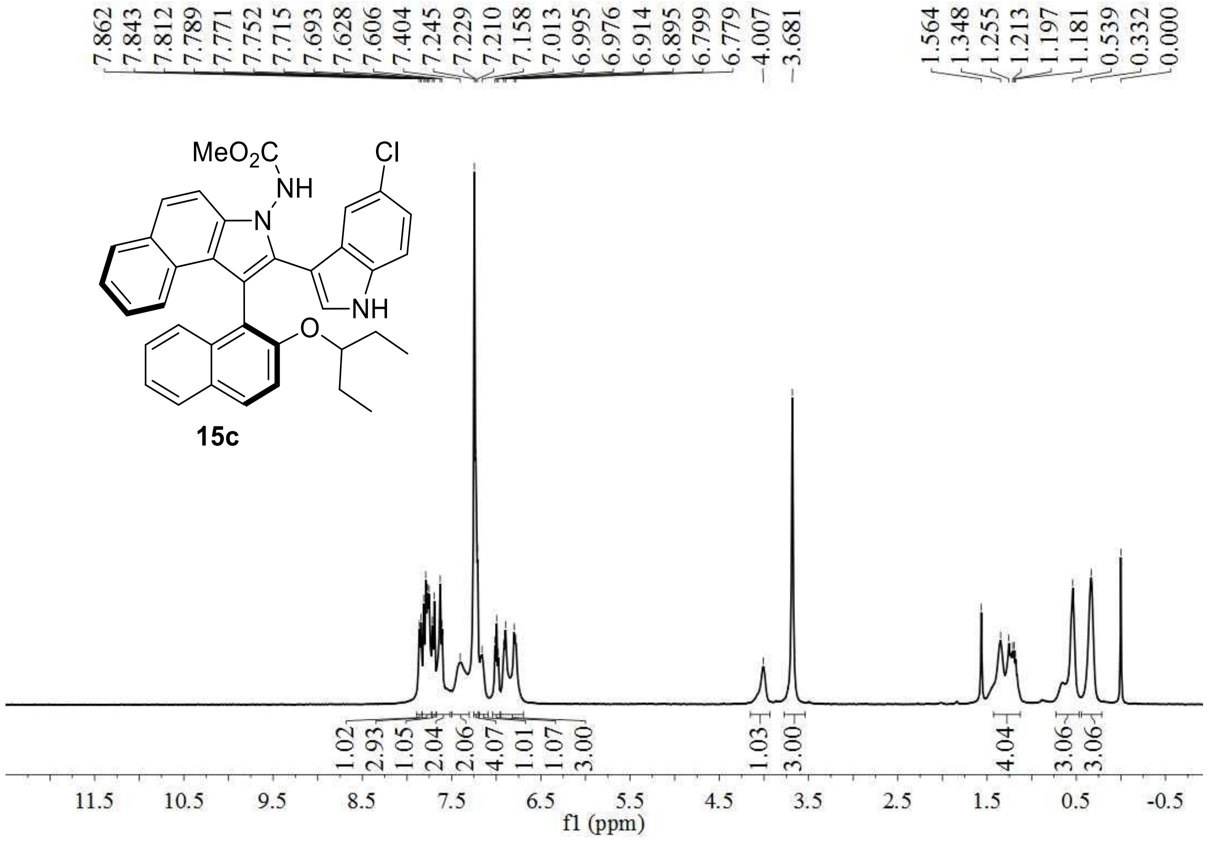
7.862
7.843
7.812
7.789
7.771
7.752
7.715
7.693
7.628
7.606
7.404
7.245
7.229
7.210
7.158
7.013
6.995
6.976
6.914
6.895
6.799
6.779
4.007
3.681
1.564
1.348
1.255
1.213
1.197
1.181
0.539
0.332
0.000
MeO2C
NH
N
Cl
NH
O
15c
1.02
2.93
1.05
2.04
2.06
4.07
1.01
1.07
3.00
1.03
3.00
4.04
3.06
3.06
11.5 10.5 9.5 8.5 7.5 6.5 5.5 4.5 3.5 2.5 1.5 0.5 -0.5
f1 (ppm)

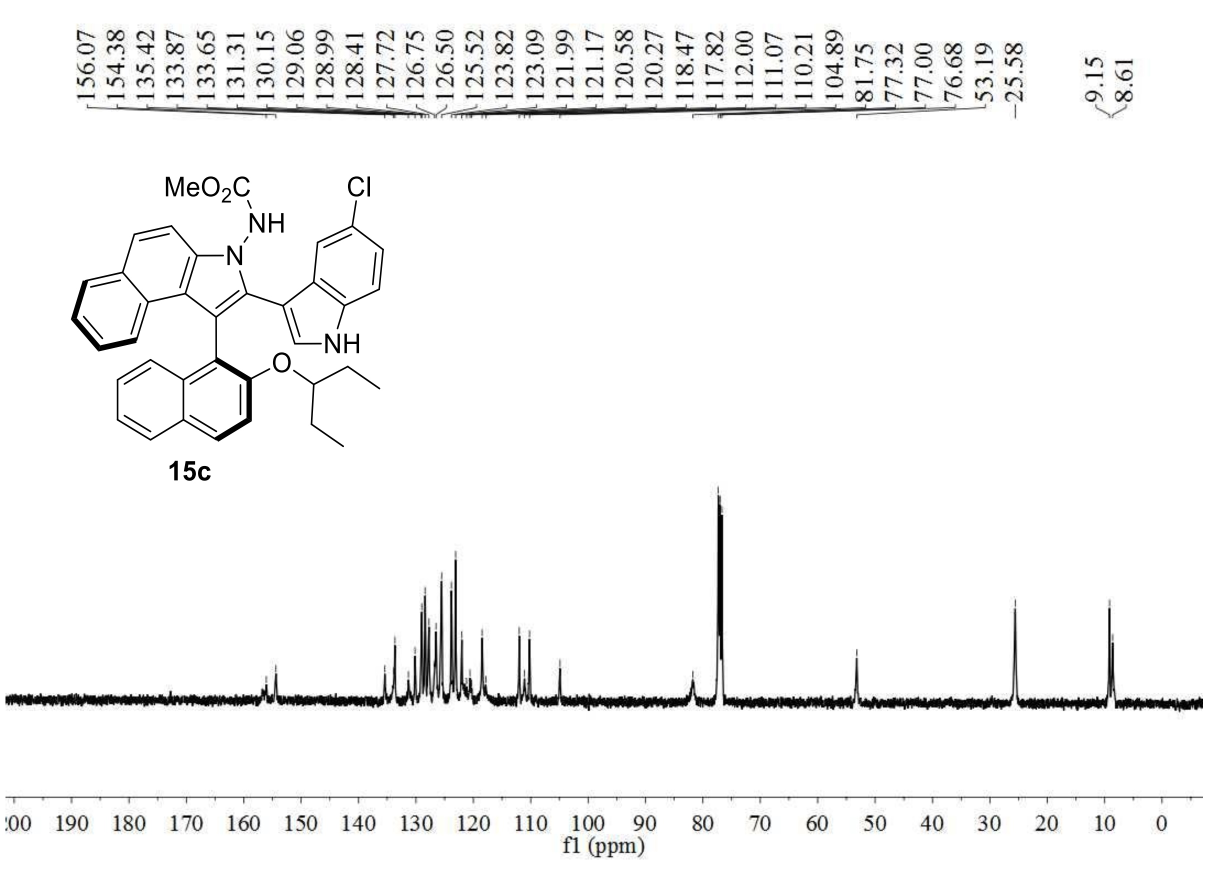
156.07
154.38
135.42
133.87
133.65
131.31
130.15
129.06
128.99
128.41
127.72
126.75
126.50
125.52
123.82
123.09
121.99
121.17
120.58
120.27
118.47
117.82
112.00
111.07
110.21
104.89
81.75
77.32
77.00
76.68
53.19
25.58
9.15
8.61
MeO2C
NH
N
Cl
NH
O
15c
190 180 170 160 150 140 130 120 110 100 90 80 70 60 50 40 30 20 10 0
f1 (ppm)

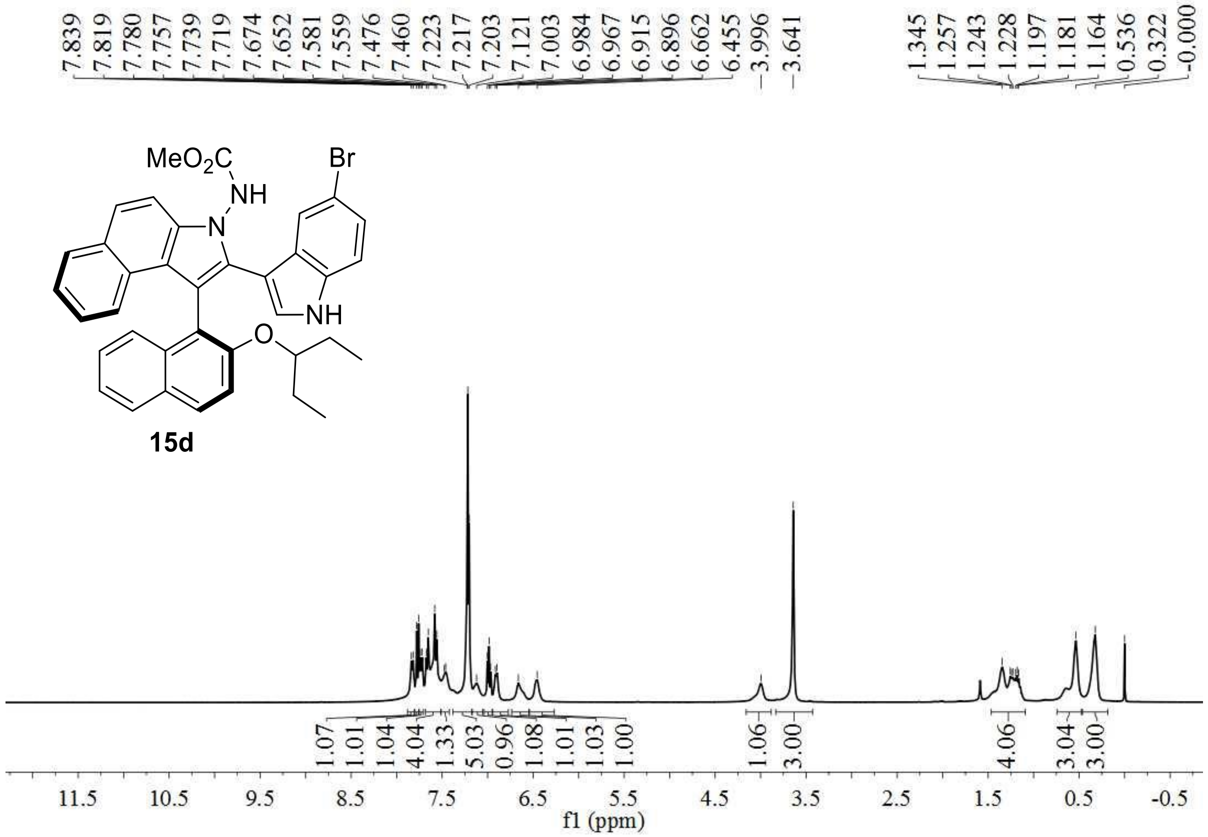
7.839 7.819 7.780 7.757 7.739 7.719 7.674 7.652 7.581 7.559 7.476 7.460 7.223 7.217 7.203 7.121 7.003 6.984 6.967 6.915 6.896 6.662 6.455 3.996 3.641
1.345 1.257 1.243 1.228 1.197 1.181 1.164 0.536 0.322 -0.000
MeO2C
NH
N
Br
NH
O
15d
1.07 1.01 1.04 4.04 1.33 5.03 0.96 1.08 1.01 1.03 1.00 1.06 3.00 4.06 3.04 3.00
11.5 10.5 9.5 8.5 7.5 6.5 5.5 4.5 3.5 2.5 1.5 0.5 -0.5
f1 (ppm)

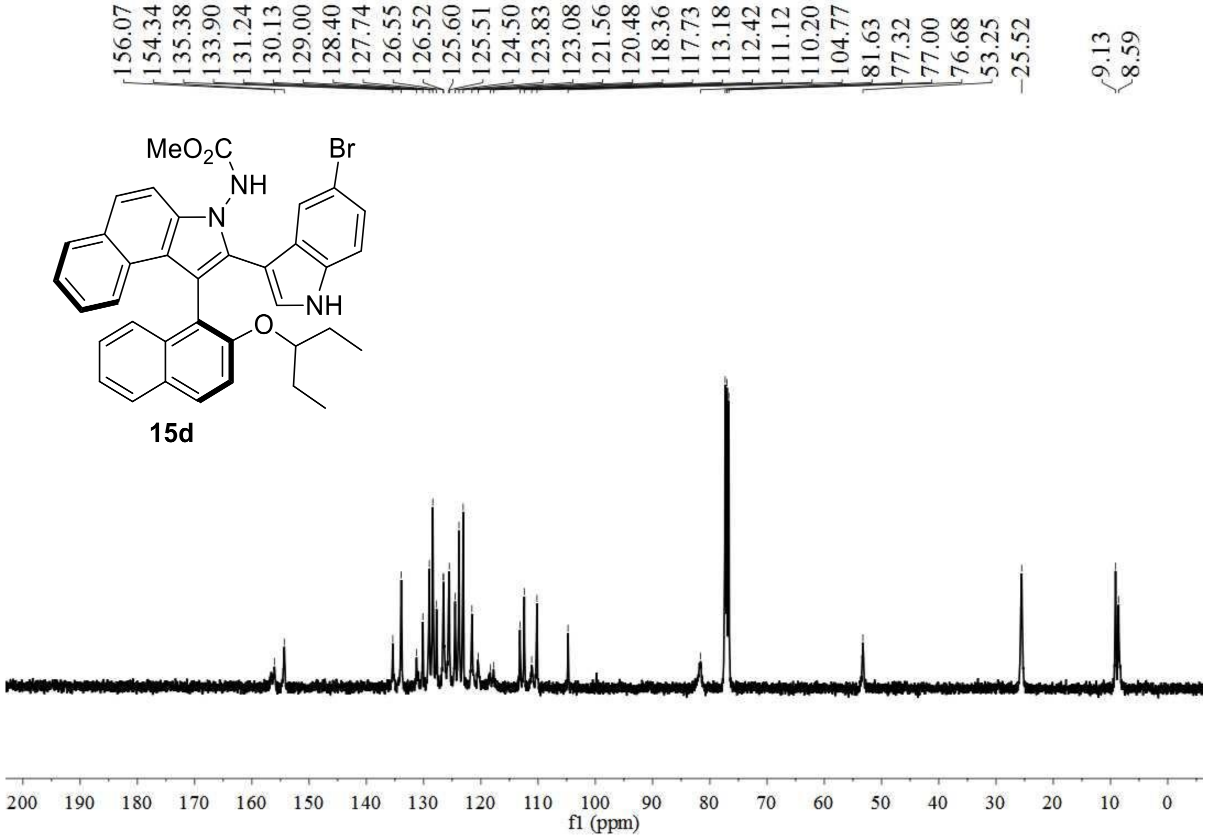
156.07 154.34 135.38 133.90 131.24 130.13 129.00 128.40 127.74 126.55 126.52 125.60 125.51 124.50 123.83 123.08 121.56 120.48 118.36 117.73 113.18 112.42 111.12 110.20 104.77 81.63 77.32 77.00 76.68 53.25 25.52
9.13 8.59
MeO2C
NH
N
Br
NH
O
15d
200 190 180 170 160 150 140 130 120 110 100 90 80 70 60 50 40 30 20 10 0
f1 (ppm)

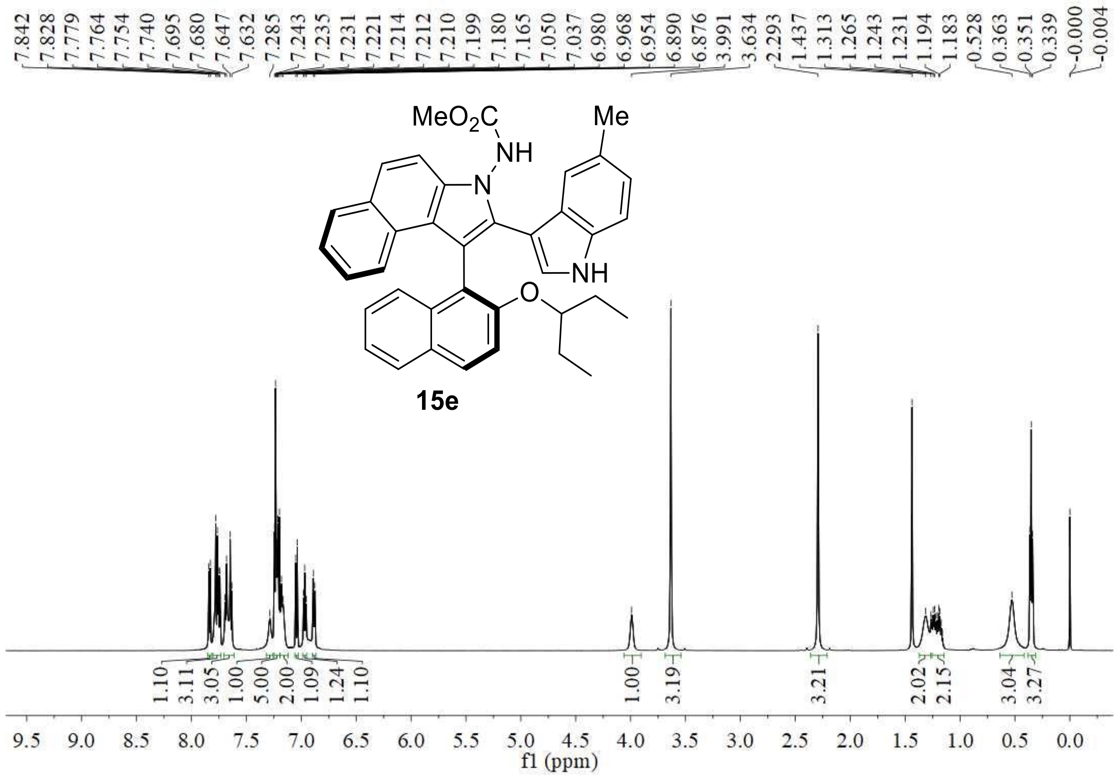
7.842 7.828 7.779 7.764 7.754 7.740 7.695 7.680 7.647 7.632 7.285 7.243 7.235 7.231 7.221 7.214 7.212 7.210 7.199 7.180 7.165 7.050 7.037 6.980 6.968 6.954 6.890 6.876 3.991 3.634 2.293 1.437 1.313 1.265 1.243 1.231 1.194 1.183 0.528 0.363 0.351 0.339 -0.000 -0.004
MeO2C
NH
N
Me
NH
O
15e
1.10 3.11 3.05 1.00 5.00 2.00 1.09 1.24 1.10 1.00 3.19 3.21 2.02 2.15 3.04 3.27
f1 (ppm)

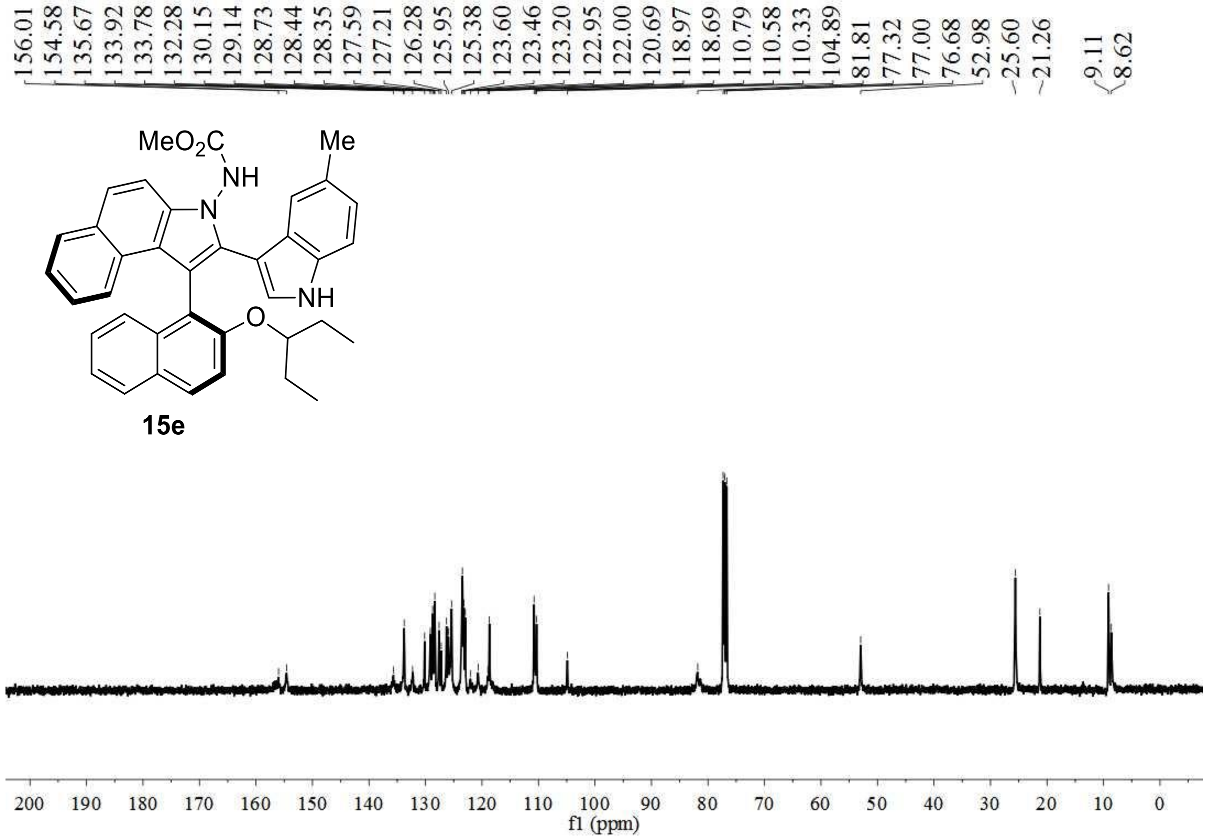
156.01 154.58 135.67 133.92 133.78 132.28 130.15 129.14 128.73 128.44 128.35 127.59 127.21 126.28 125.95 125.38 123.60 123.46 123.20 122.95 122.00 120.69 118.97 118.69 110.79 110.58 110.33 104.89 81.81 77.32 77.00 76.68 52.98 25.60 21.26 9.11 8.62
MeO2C
NH
N
Me
NH
O
15e
f1 (ppm)

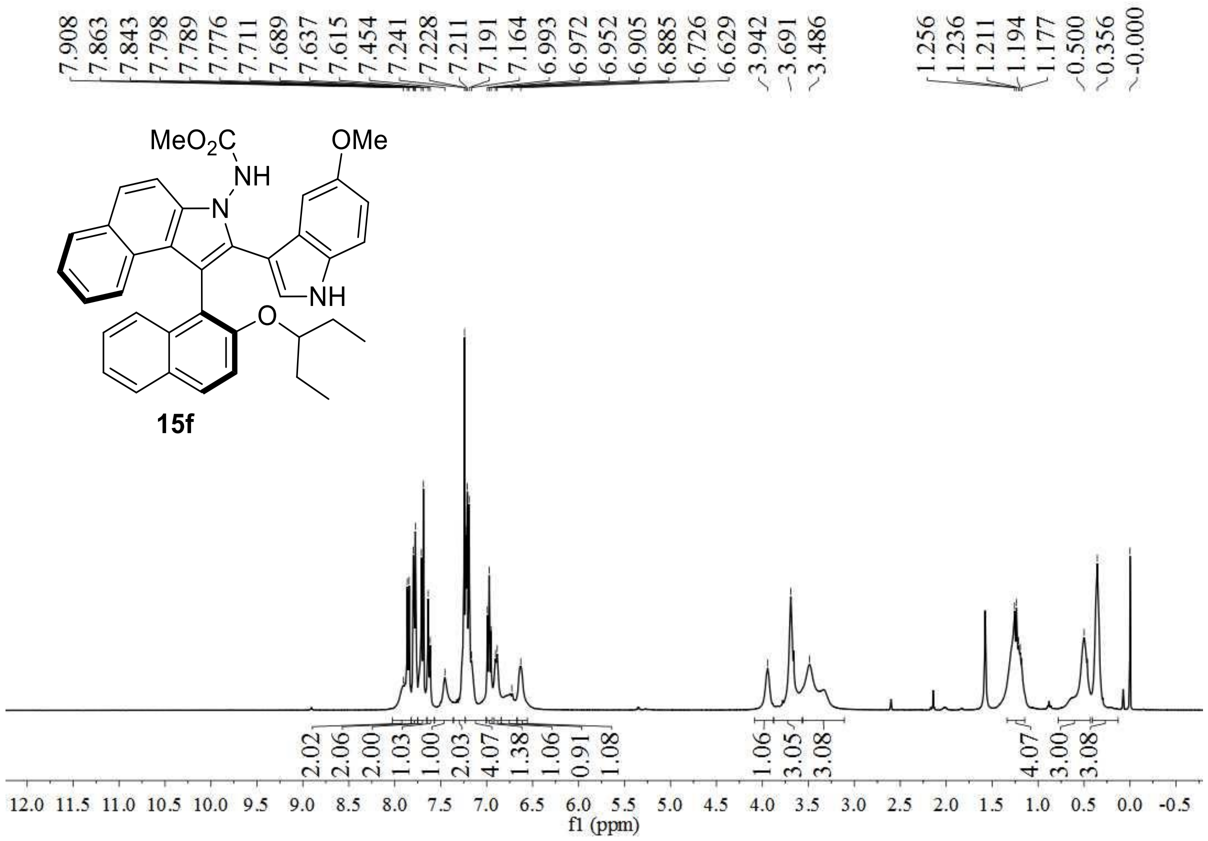

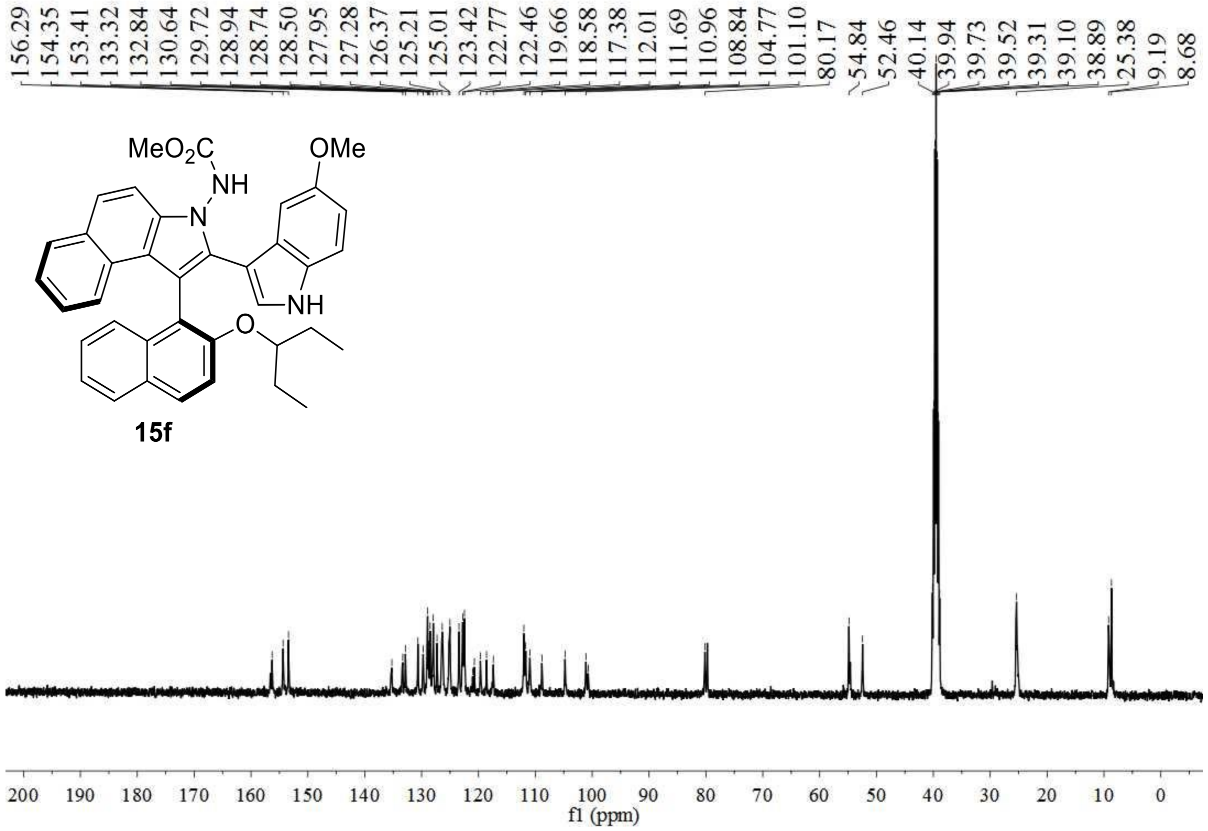

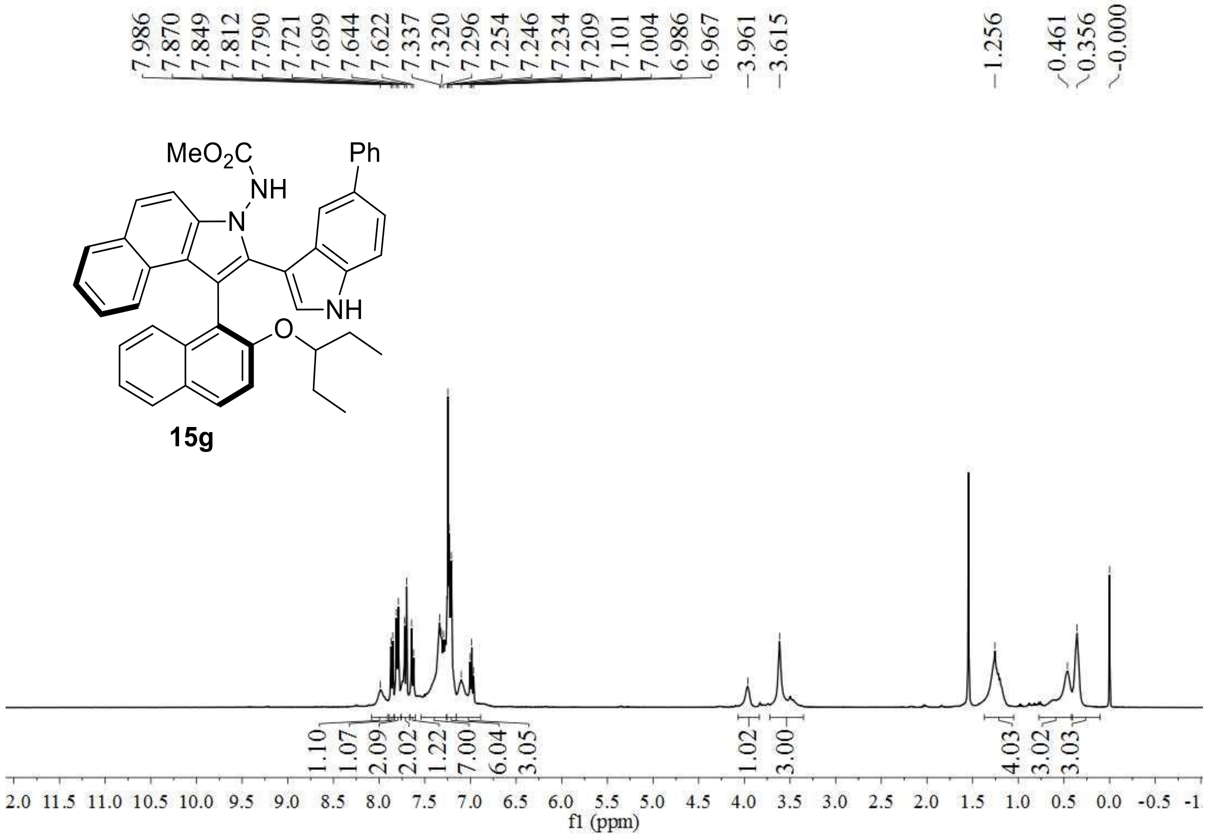
7.986
7.870
7.849
7.812
7.790
7.721
7.699
7.644
7.622
7.337
7.320
7.296
7.254
7.246
7.234
7.209
7.101
7.004
6.986
6.967
3.961
3.615
1.256
0.461
0.356
-0.000
MeO2C
NH
N
Ph
NH
O
15g
1.10
1.07
2.09
2.02
1.22
7.00
6.04
3.05
1.02
3.00
4.03
3.02
3.03
f1 (ppm)

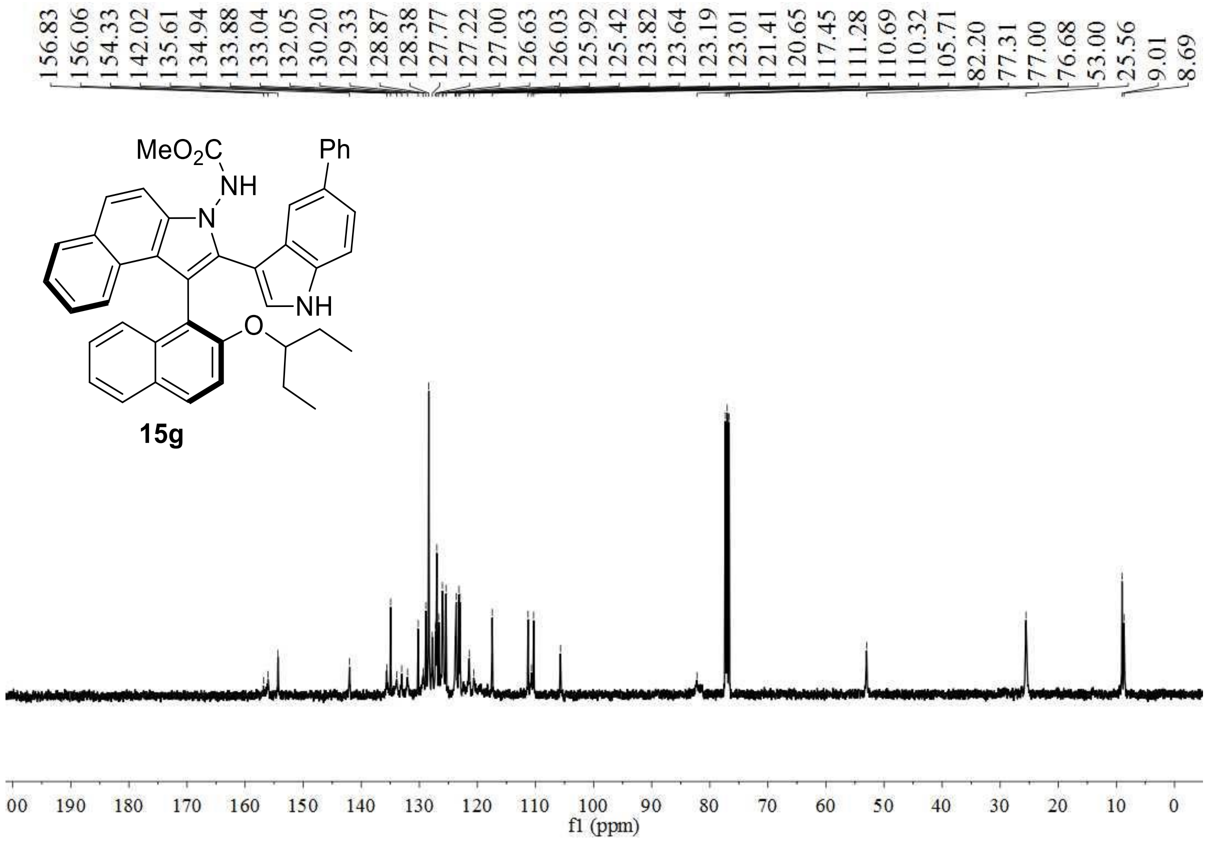
156.83
156.06
154.33
142.02
135.61
134.94
133.88
133.04
132.05
130.20
129.33
128.87
128.38
127.77
127.22
127.00
126.63
126.03
125.92
125.42
123.82
123.64
123.19
123.01
121.41
120.65
117.45
111.28
110.69
110.32
105.71
82.20
77.31
77.00
76.68
53.00
25.56
9.01
8.69
MeO2C
NH
N
Ph
NH
O
15g
f1 (ppm)

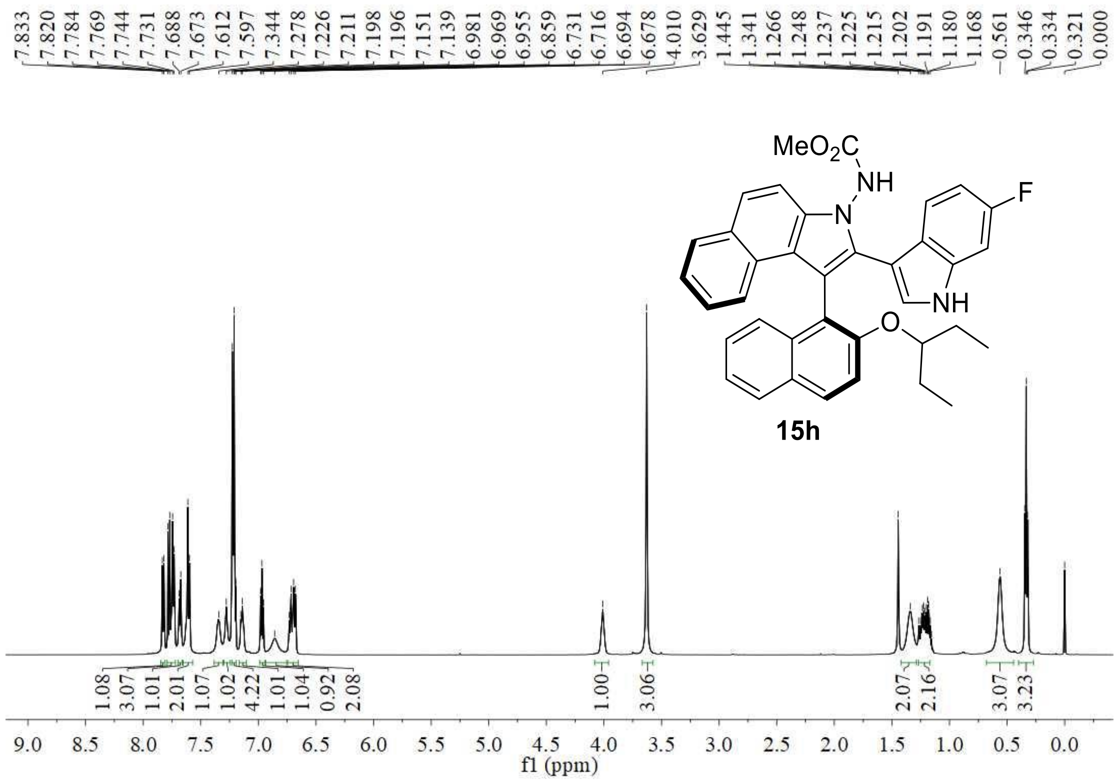

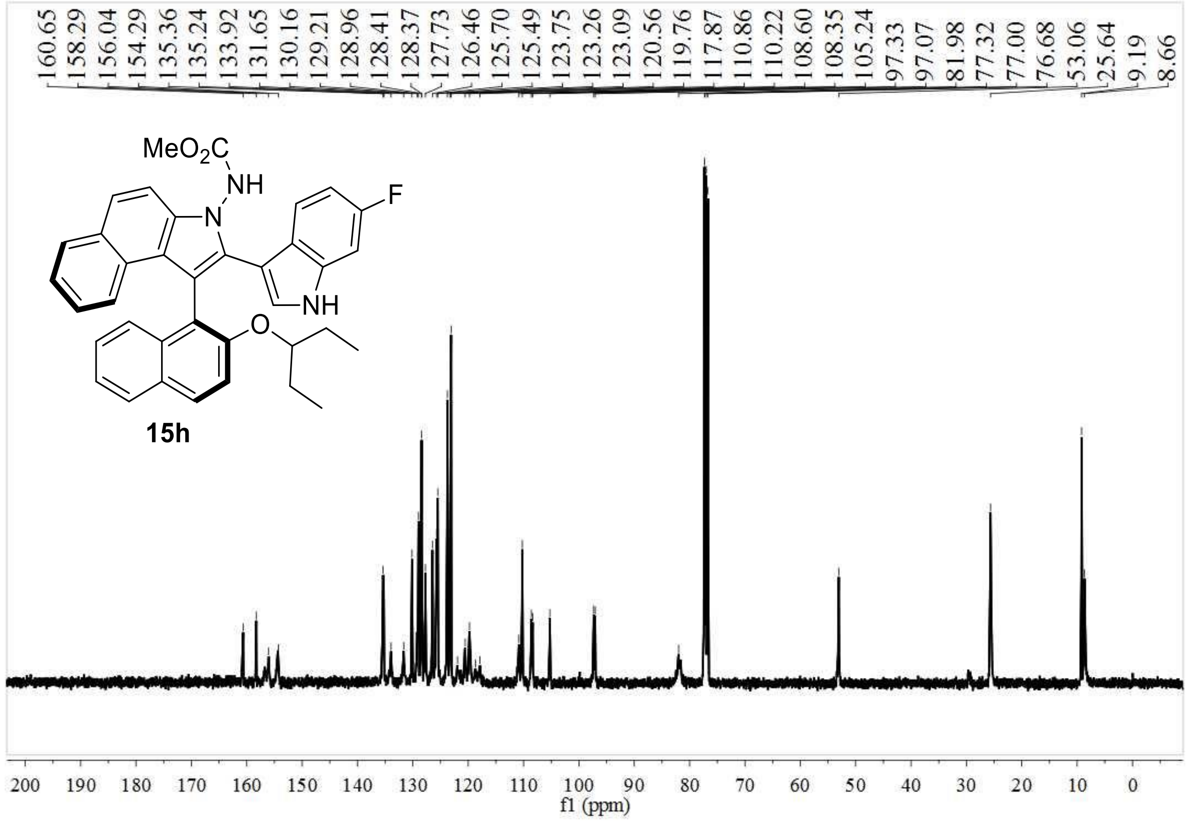

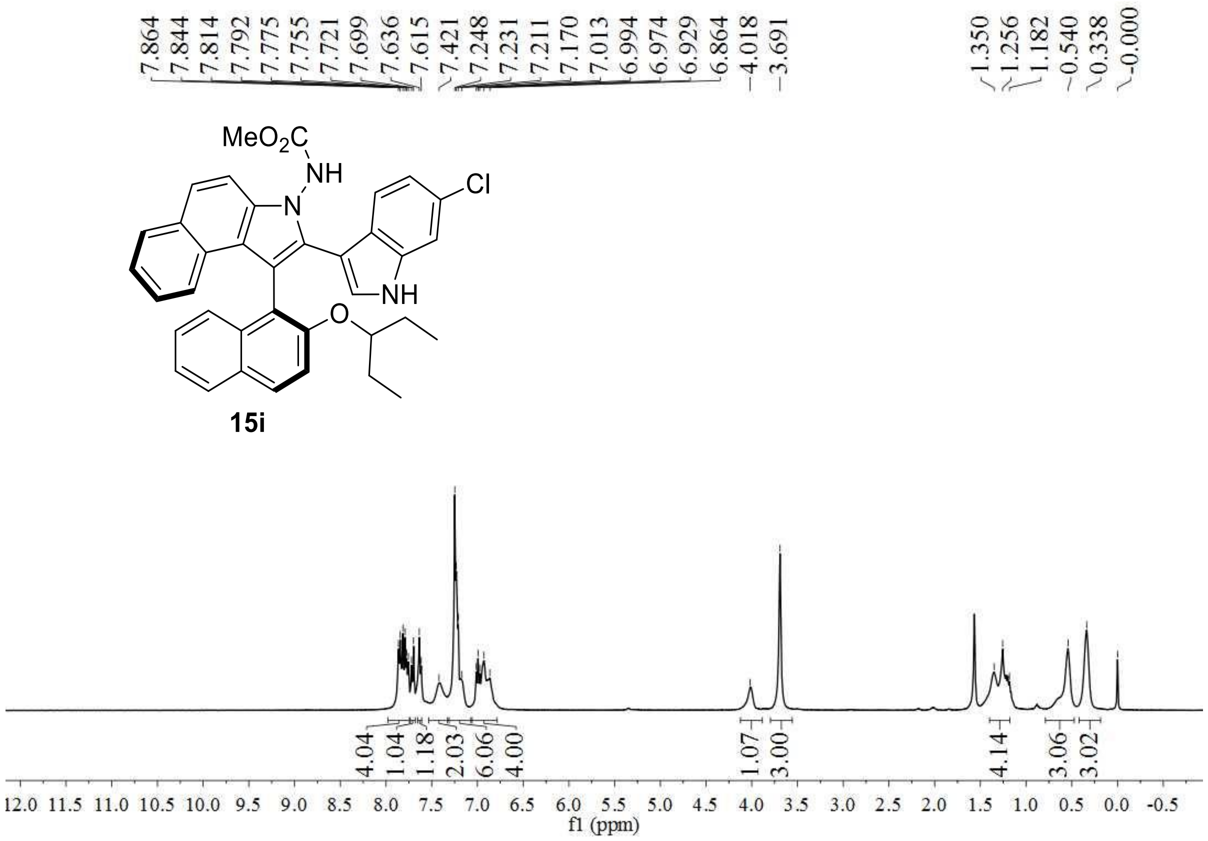

7.864
7.844
7.814
7.792
7.775
7.755
7.721
7.699
7.636
7.615
7.421
7.248
7.231
7.211
7.170
7.013
6.994
6.974
6.929
6.864
4.018
3.691
1.350
1.256
1.182
0.540
0.338
-0.000
MeO2C
NH
N
Cl
NH
O
15i
4.04
1.04
1.18
2.03
6.06
4.00
1.07
3.00
4.14
3.06
3.02
12.0 11.5 11.0 10.5 10.0 9.5 9.0 8.5 8.0 7.5 7.0 6.5 6.0 5.5 5.0 4.5 4.0 3.5 3.0 2.5 2.0 1.5 1.0 0.5 0.0 -0.5
f1 (ppm)

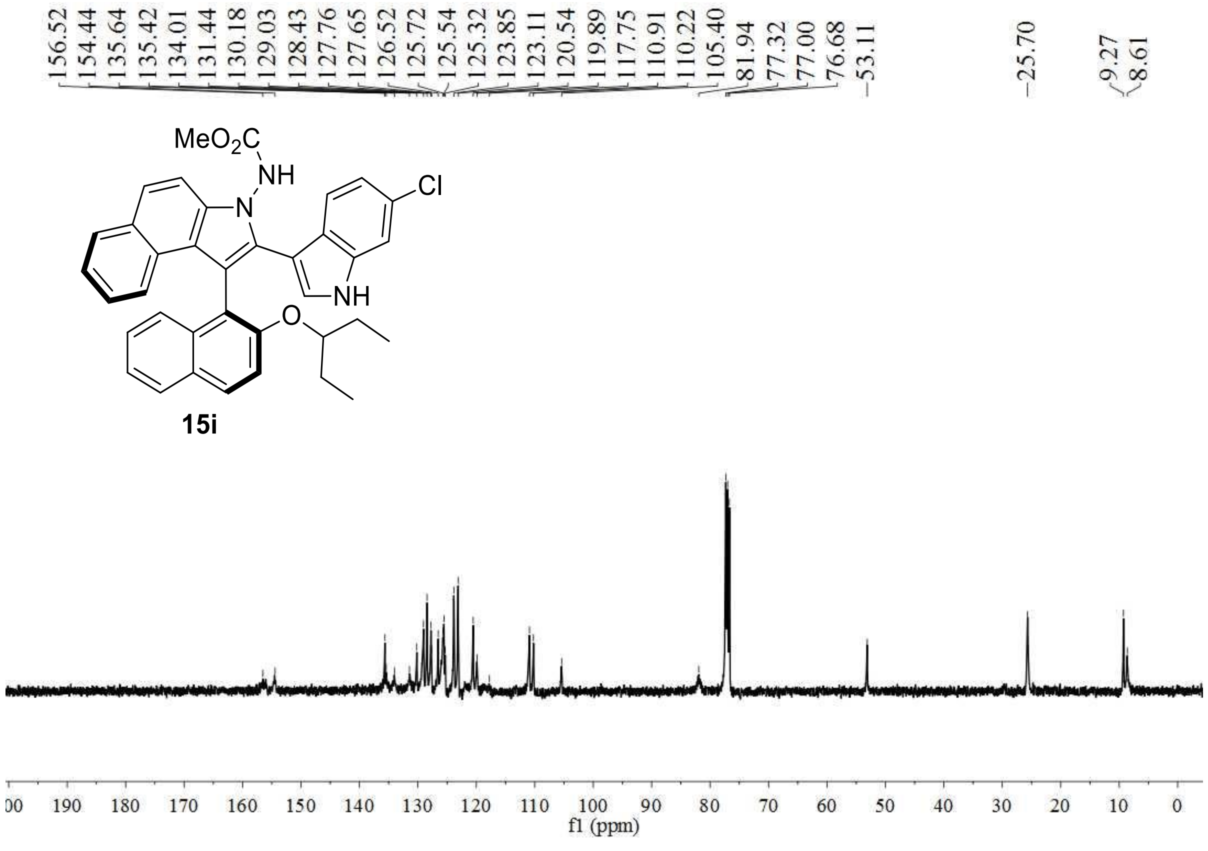

156.52
154.44
135.64
135.42
134.01
131.44
130.18
129.03
128.43
127.76
127.65
126.52
125.72
125.54
125.32
123.85
123.11
120.54
119.89
117.75
110.91
110.22
105.40
81.94
77.32
77.00
76.68
53.11
25.70
9.27
8.61
MeO2C
NH
N
Cl
NH
O
15i
190 180 170 160 150 140 130 120 110 100 90 80 70 60 50 40 30 20 10 0
f1 (ppm)

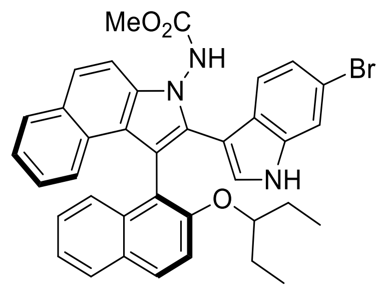
MeO2C
NH
N
Br
NH
O

15j

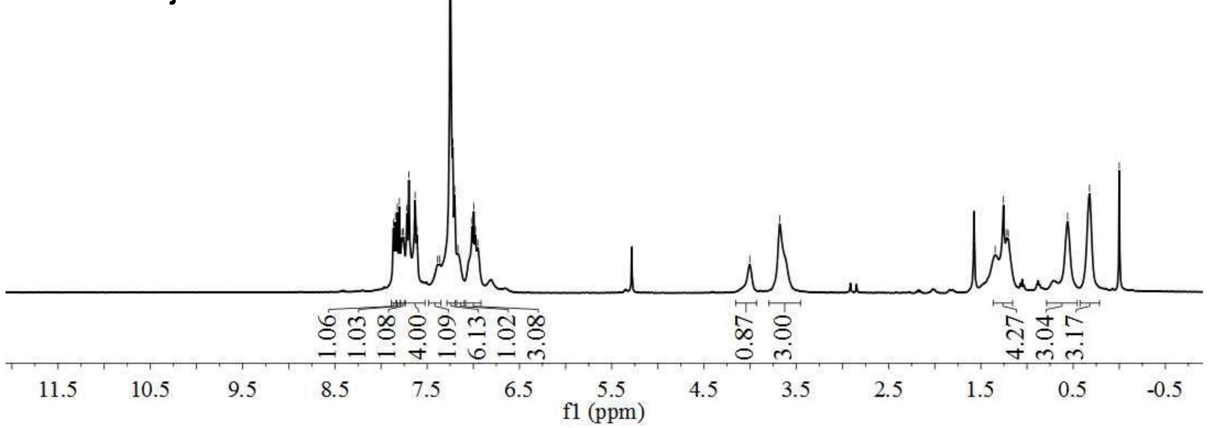
7.865
7.845
7.824
7.801
7.774
7.758
7.720
7.698
7.631
7.610
7.389
7.363
7.249
7.223
7.202
7.167
7.014
6.995
6.976
6.951
4.004
3.679
1.345
1.255
1.218
1.203
0.561
0.323
0.000
1.06
1.03
1.08
4.00
1.09
6.13
1.02
3.08
0.87
3.00
4.27
3.04
3.17
11.5
10.5
9.5
8.5
7.5
6.5
5.5
4.5
3.5
2.5
1.5
0.5
-0.5
f1 (ppm)

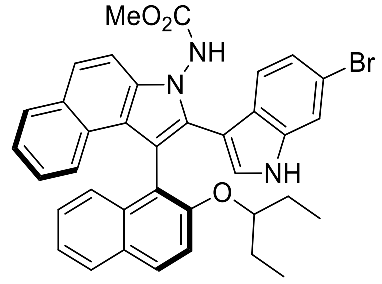
MeO2C
NH
N
Br
NH
O

15j

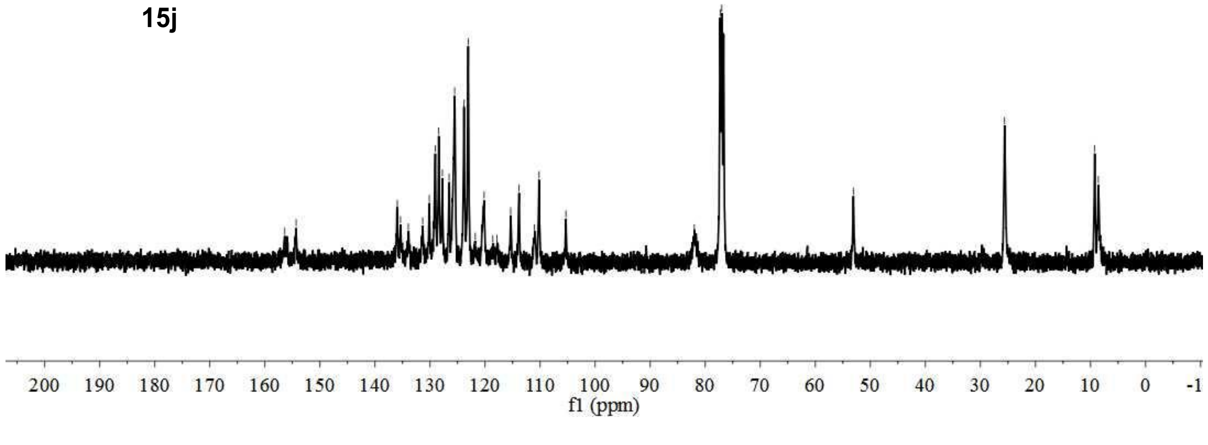
156.40
154.33
135.96
135.37
133.95
131.34
130.14
129.05
128.42
127.76
126.54
125.53
123.82
123.07
121.78
120.38
120.17
118.59
117.85
115.35
113.82
111.03
110.20
105.33
81.99
77.29
77.00
76.65
53.10
25.62
9.24
8.59
200
190
180
170
160
150
140
130
120
110
100
90
80
70
60
50
40
30
20
10
0
-1
f1 (ppm)

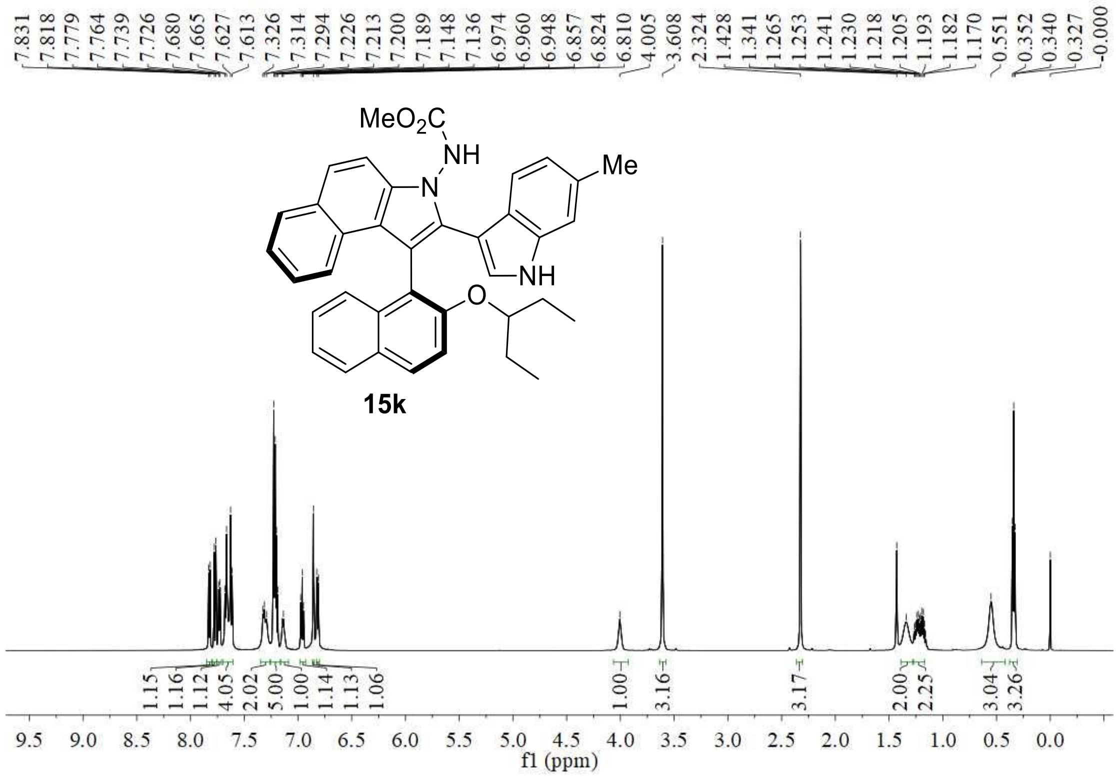
7.831 7.818 7.779 7.764 7.739 7.726 7.680 7.665 7.627 7.613 7.326 7.314 7.294 7.226 7.213 7.200 7.189 7.148 7.136 6.974 6.960 6.948 6.857 6.824 6.810 4.005 3.608 2.324 1.428 1.341 1.265 1.253 1.241 1.230 1.218 1.205 1.193 1.182 1.170 0.551 0.352 0.340 0.327 -0.000
MeO2C
NH
N
Me
NH
O
15k
1.15 1.16 1.12 4.05 2.02 5.00 1.00 1.14 1.13 1.06 1.00 3.16 3.17 2.00 2.25 3.04 3.26
9.5 9.0 8.5 8.0 7.5 7.0 6.5 6.0 5.5 5.0 4.5 4.0 3.5 3.0 2.5 2.0 1.5 1.0 0.5 0.0
f1 (ppm)

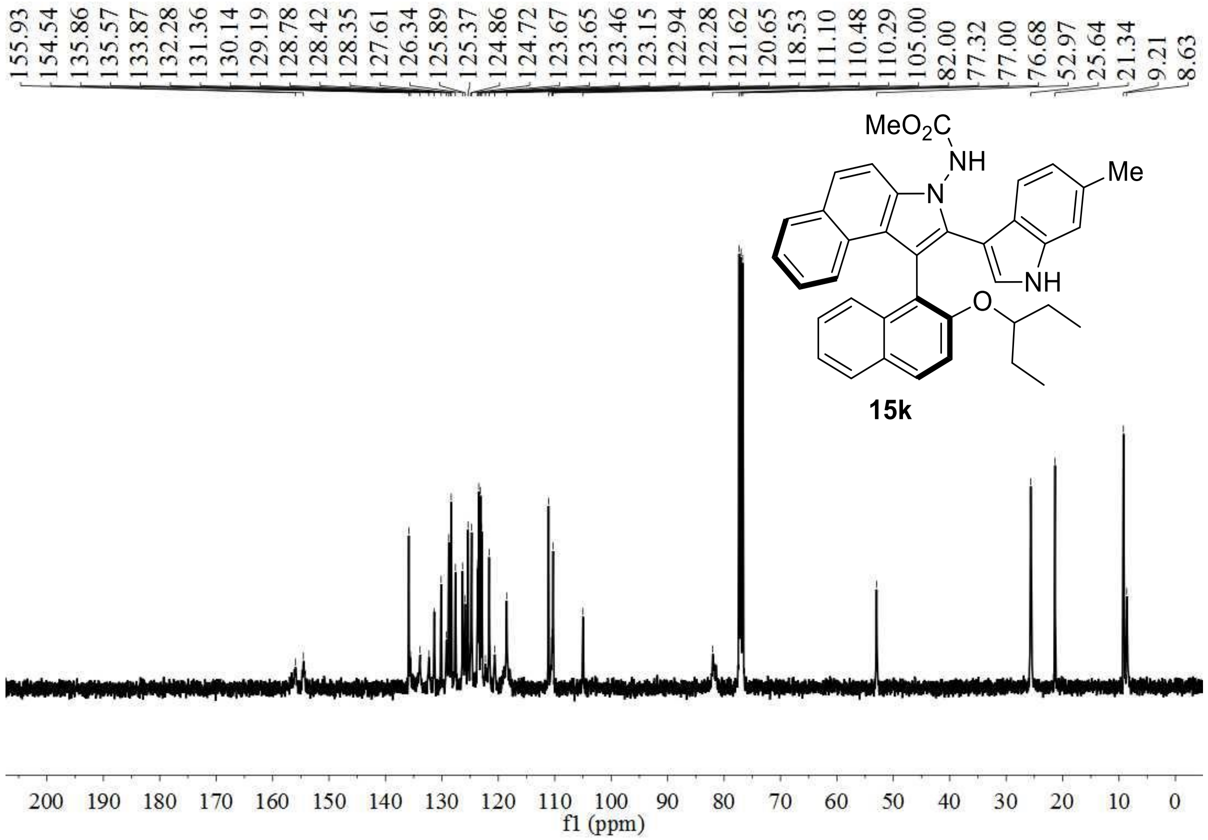
155.93 154.54 135.86 135.57 133.87 132.28 131.36 130.14 129.19 128.78 128.42 128.35 127.61 126.34 125.89 125.37 124.86 124.72 123.67 123.65 123.46 123.15 122.94 122.28 121.62 120.65 118.53 111.10 110.48 110.29 105.00 82.00 77.32 77.00 76.68 52.97 25.64 21.34 9.21 8.63
MeO2C
NH
N
Me
NH
O
15k
200 190 180 170 160 150 140 130 120 110 100 90 80 70 60 50 40 30 20 10 0
f1 (ppm)

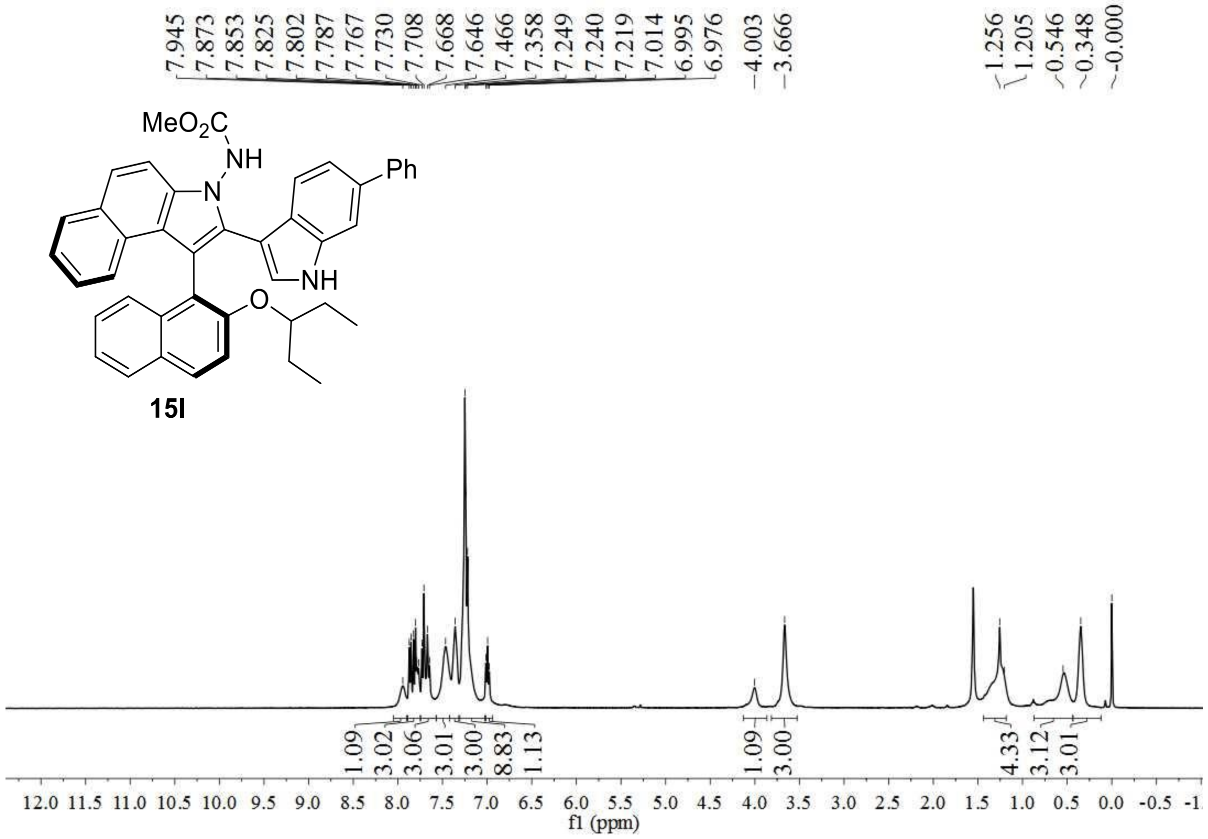
15l

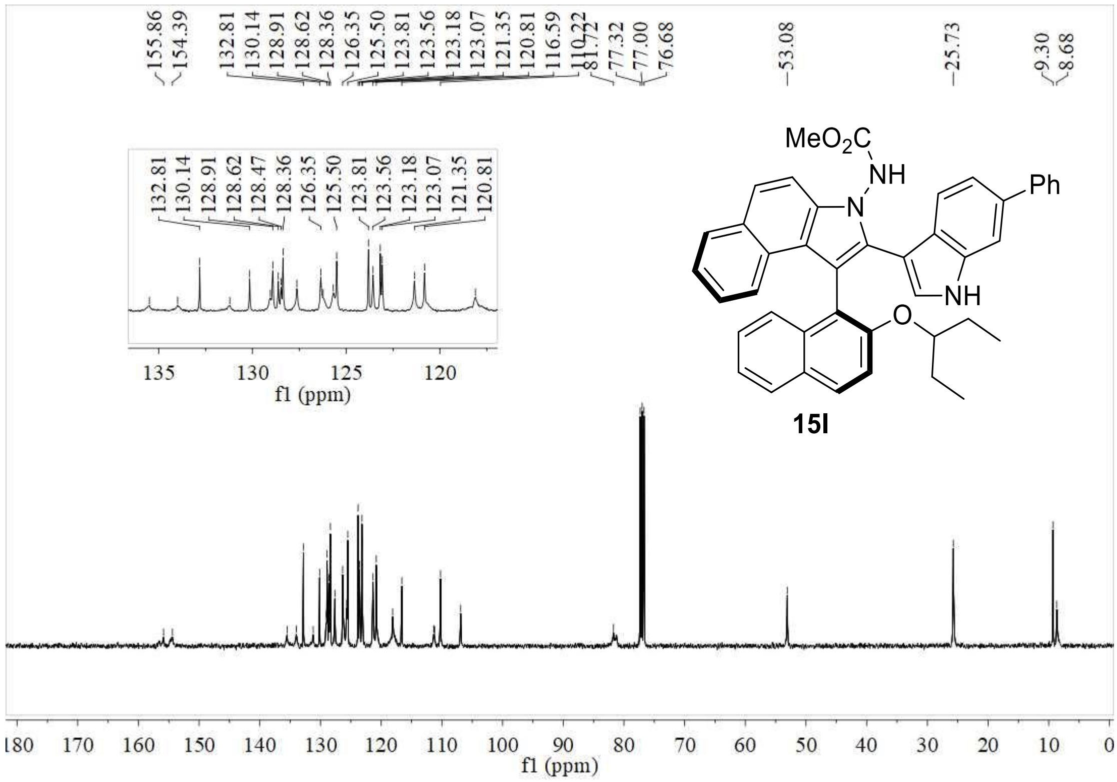
15l

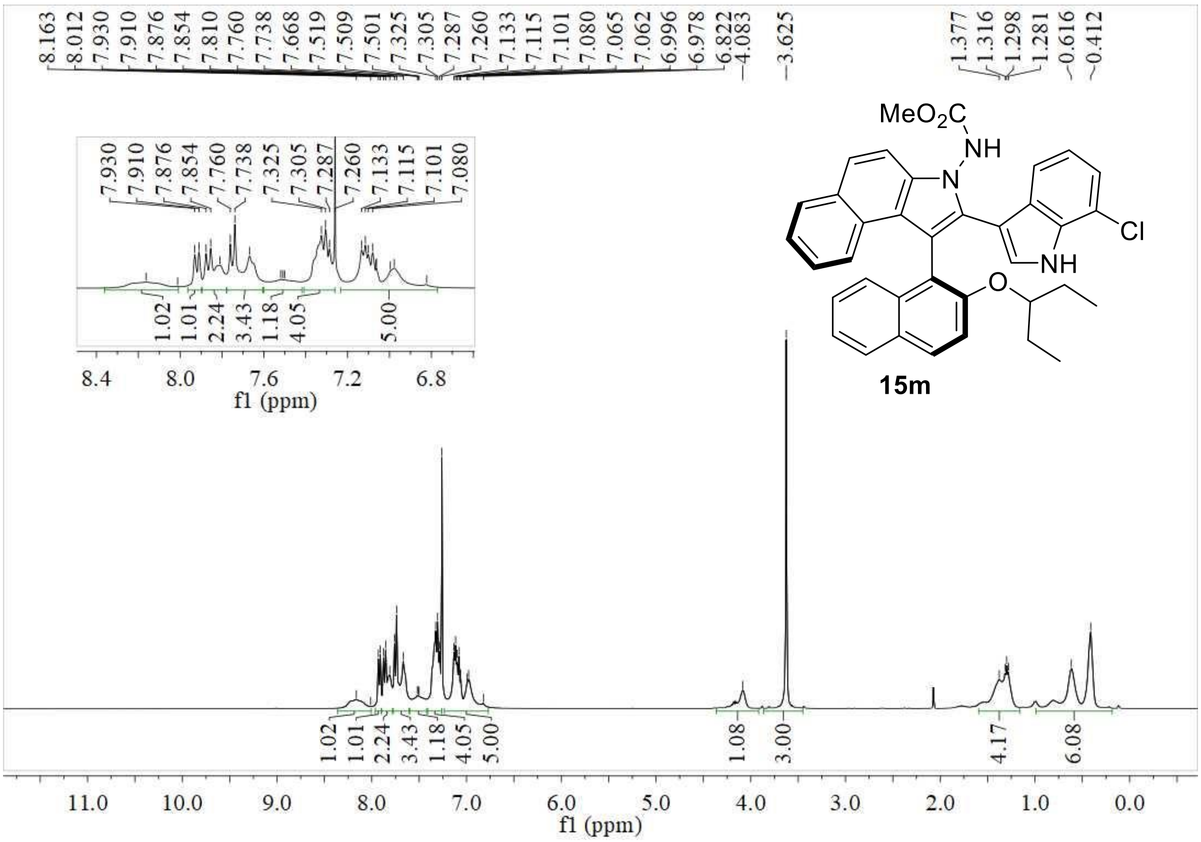

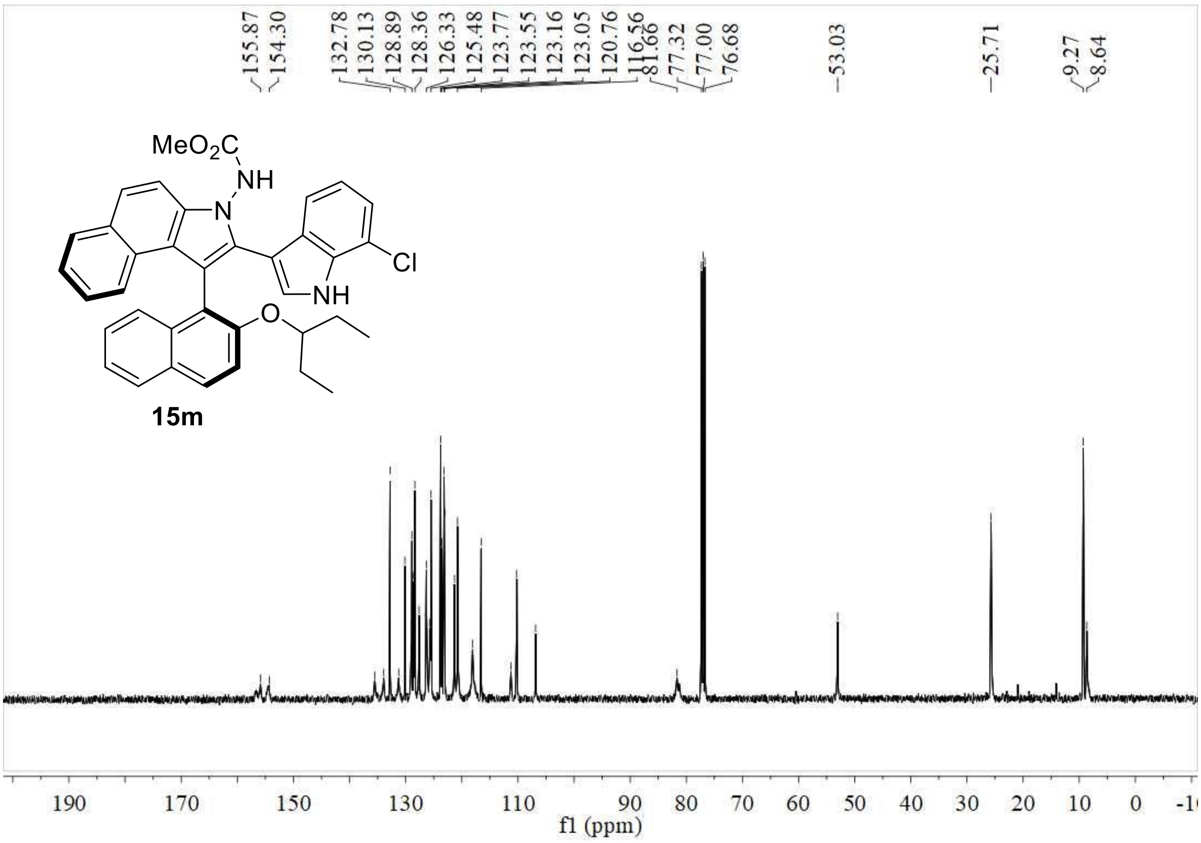

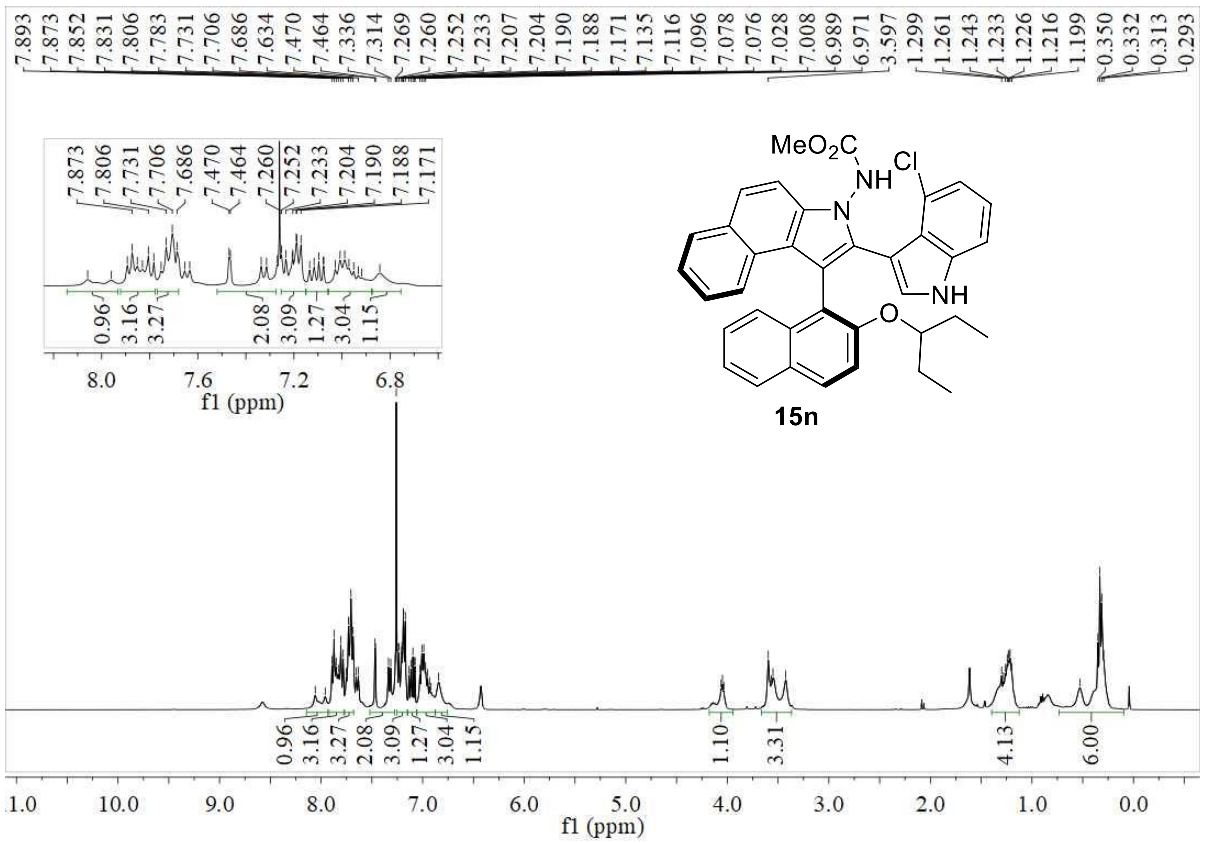
MeO2C
NH
Cl
N
NH
O
15n
f1 (ppm)

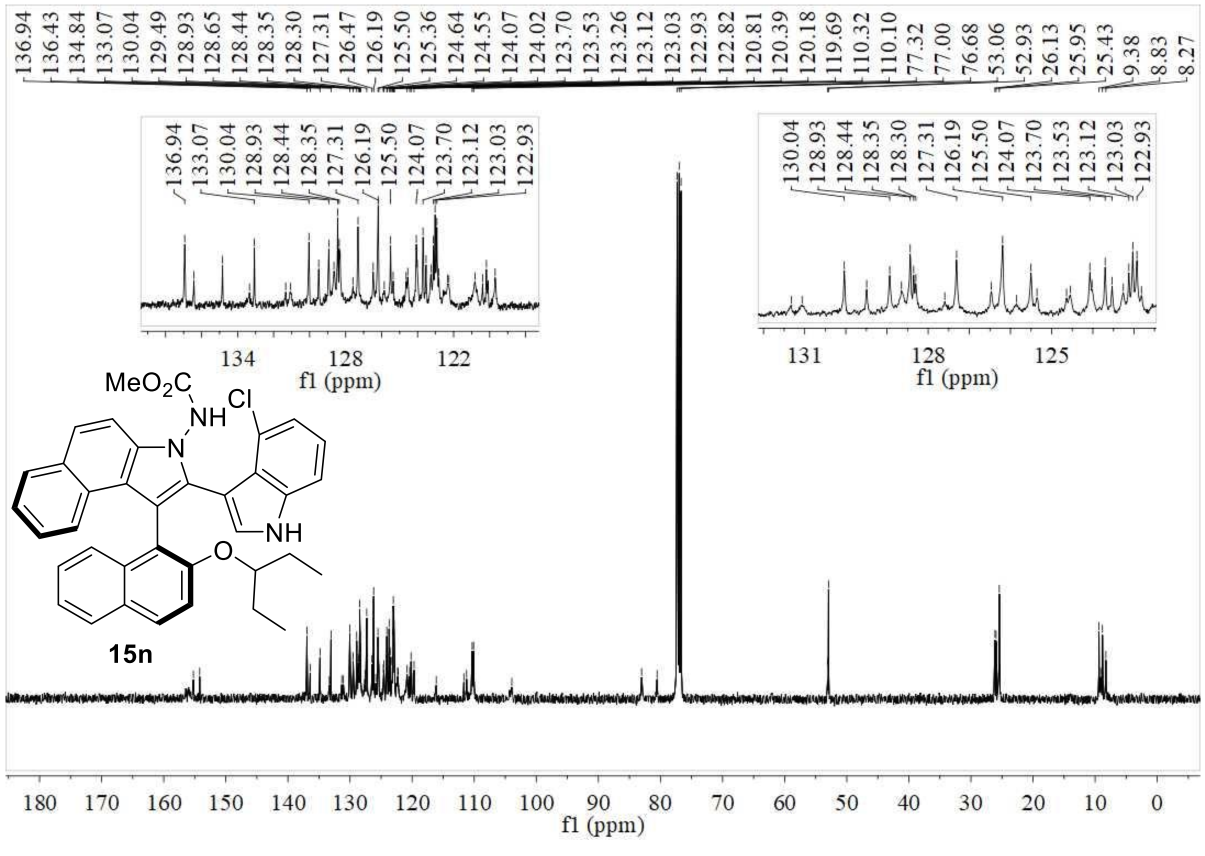
MeO2C
NH
Cl
N
NH
O
15n
f1 (ppm)

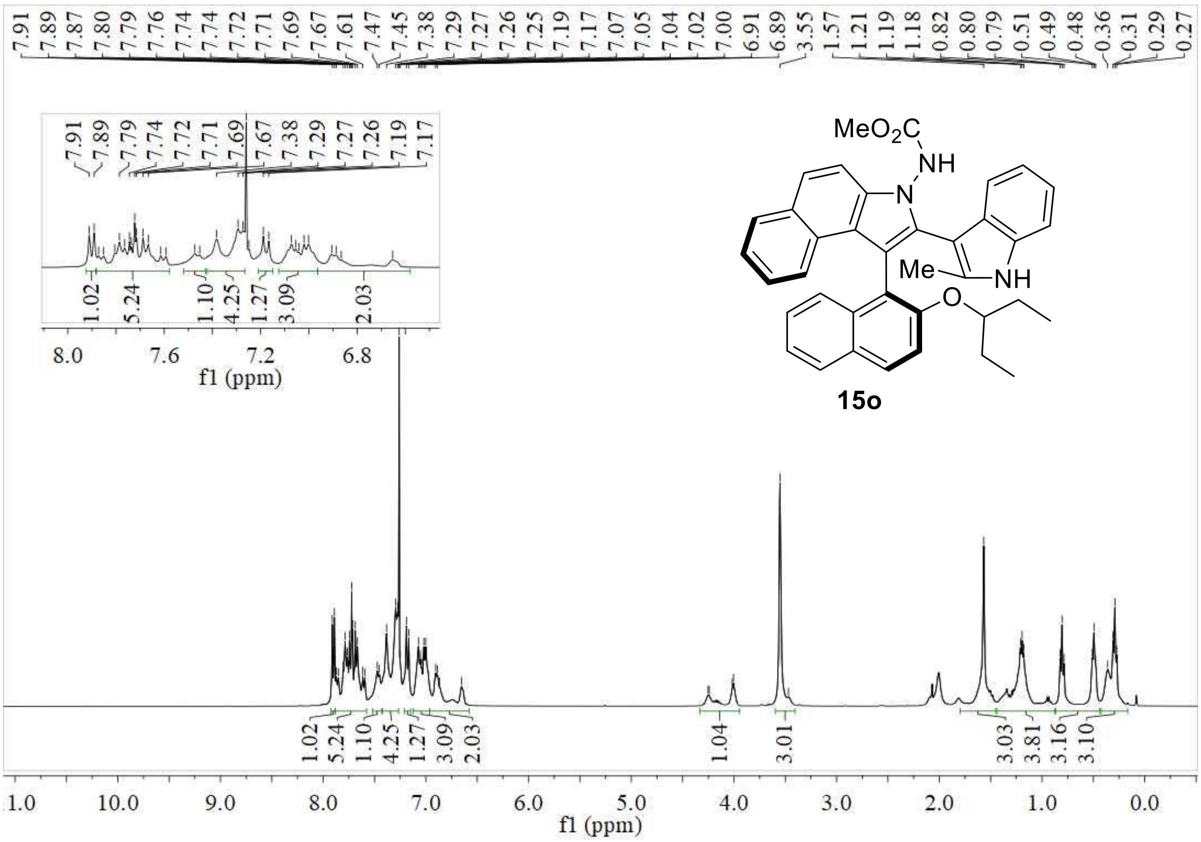
7.91 7.89 7.87 7.80 7.79 7.76 7.74 7.74 7.72 7.71 7.69 7.67 7.61 7.47 7.45 7.38 7.29 7.27 7.26 7.25 7.19 7.17 7.07 7.05 7.04 7.02 7.00 6.91 6.89 3.55 1.57 1.21 1.19 1.18 0.82 0.80 0.79 0.51 0.49 0.48 0.36 0.31 0.29 0.27
7.91 7.89 7.79 7.74 7.72 7.71 7.69 7.67 7.38 7.29 7.27 7.26 7.19 7.17
1.02 5.24 1.10 4.25 1.27 3.09 2.03
8.0 7.6 7.2 6.8
f1 (ppm)
MeO2C
NH
N
Me
NH
O
15o
1.02 5.24 1.10 4.25 1.27 3.09 2.03 1.04 3.01 3.03 3.81 3.16 3.10
1.0 10.0 9.0 8.0 7.0 6.0 5.0 4.0 3.0 2.0 1.0 0.0
f1 (ppm)

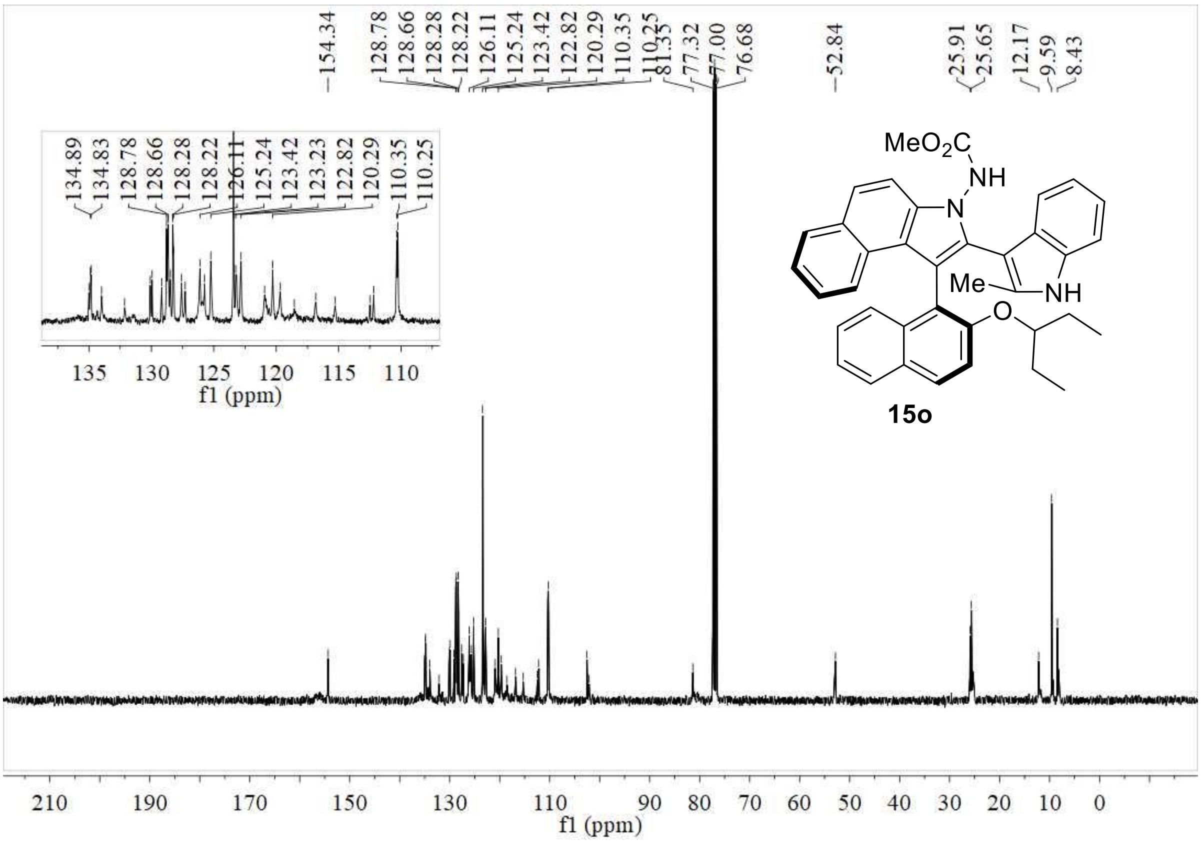
154.34 128.78 128.66 128.28 128.22 126.11 125.24 123.42 122.82 120.29 110.35 110.25 81.35 77.32 77.00 76.68 52.84 25.91 25.65 12.17 9.59 8.43
134.89 134.83 128.78 128.66 128.28 128.22 126.11 125.24 123.42 123.23 122.82 120.29 110.35 110.25
135 130 125 120 115 110
f1 (ppm)
MeO2C
NH
N
Me
NH
O
15o
210 190 170 150 130 110 90 80 70 60 50 40 30 20 10 0
f1 (ppm)

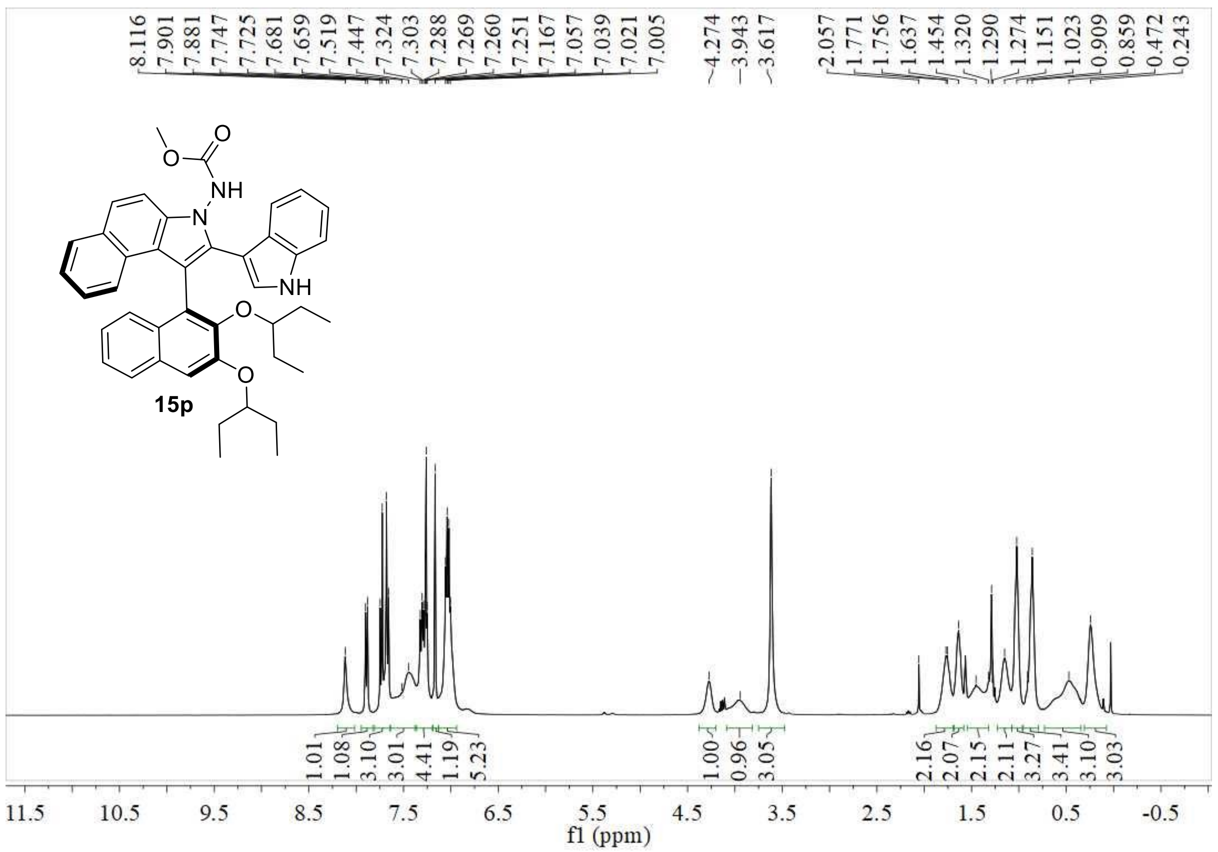

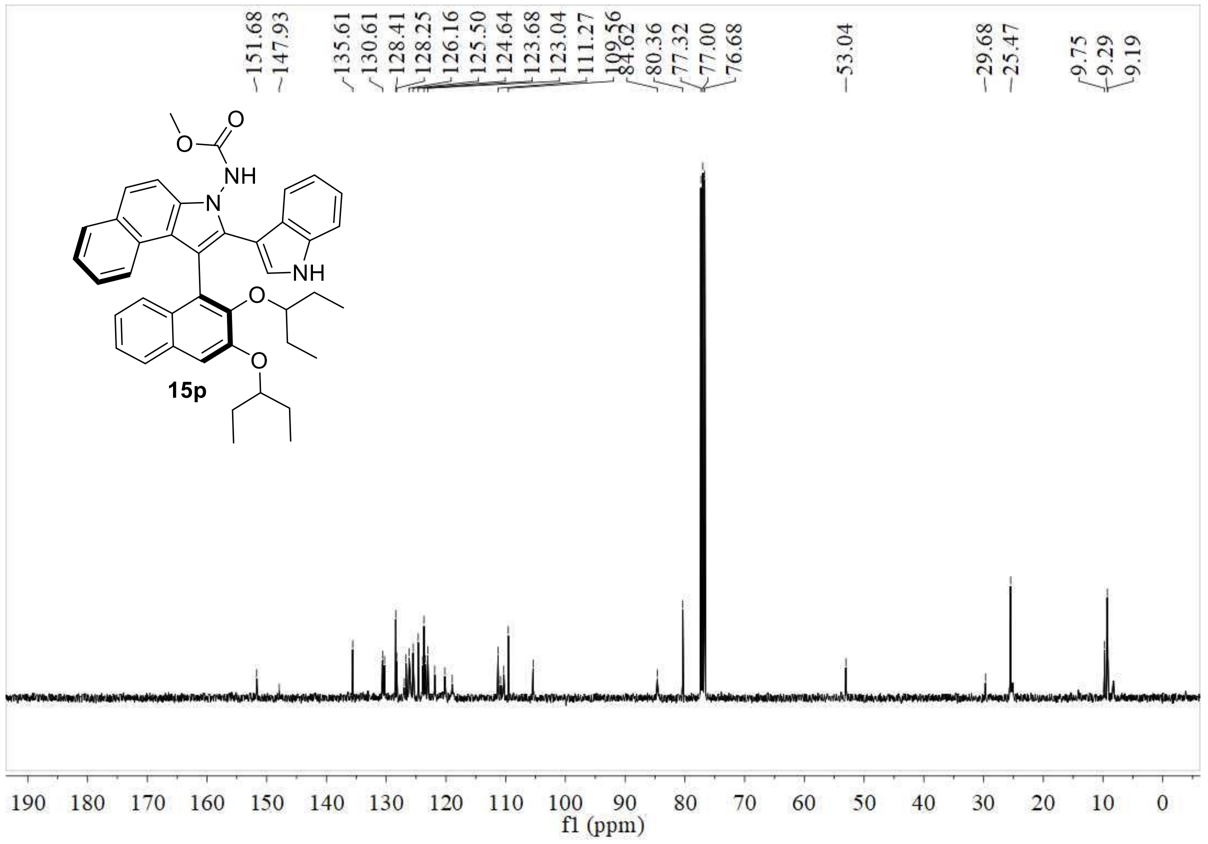

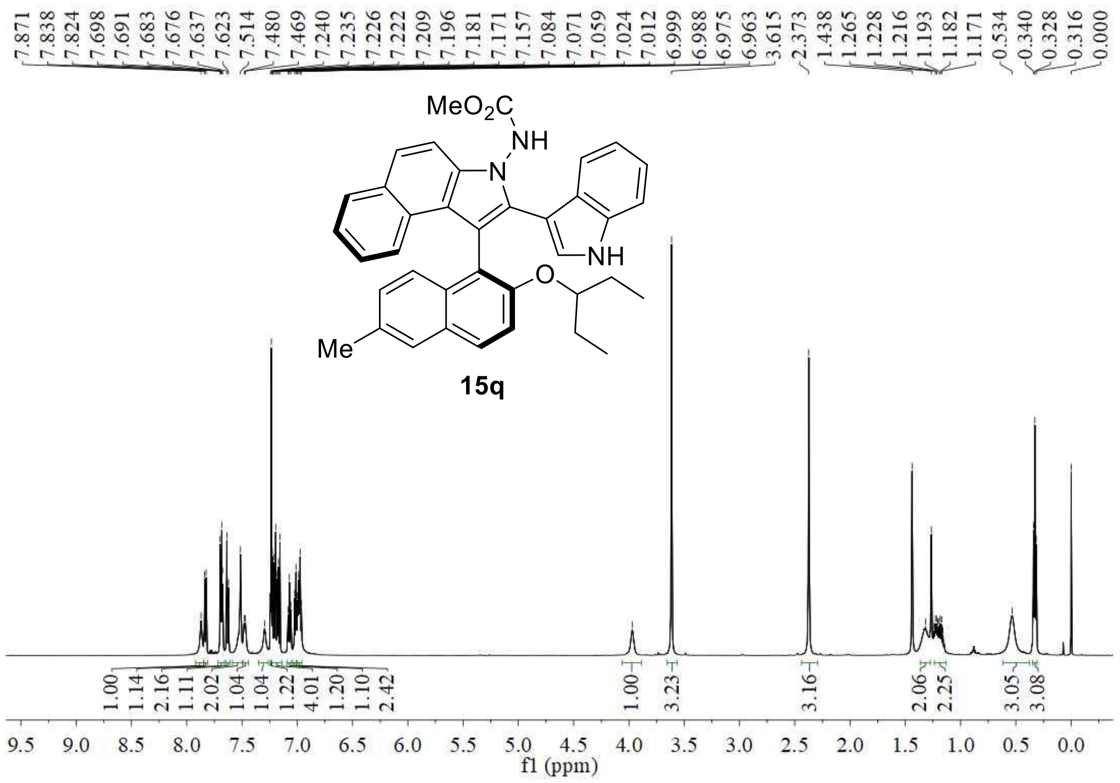
7.871 7.838 7.824 7.698 7.691 7.683 7.676 7.637 7.623 7.514 7.480 7.469 7.240 7.235 7.226 7.222 7.209 7.196 7.181 7.171 7.157 7.084 7.071 7.059 7.024 7.012 6.999 6.988 6.975 6.963 3.615 2.373 1.438 1.265 1.228 1.216 1.193 1.182 1.171 0.534 0.340 0.328 0.316 0.000
MeO2C
NH
N
NH
O
Me
15q
1.00 1.14 2.16 1.11 2.02 1.04 1.04 1.22 4.01 1.20 1.10 2.42
1.00 3.23 3.16 2.06 2.25 3.05 3.08
9.5 9.0 8.5 8.0 7.5 7.0 6.5 6.0 5.5 5.0 4.5 4.0 3.5 3.0 2.5 2.0 1.5 1.0 0.5 0.0
f1 (ppm)

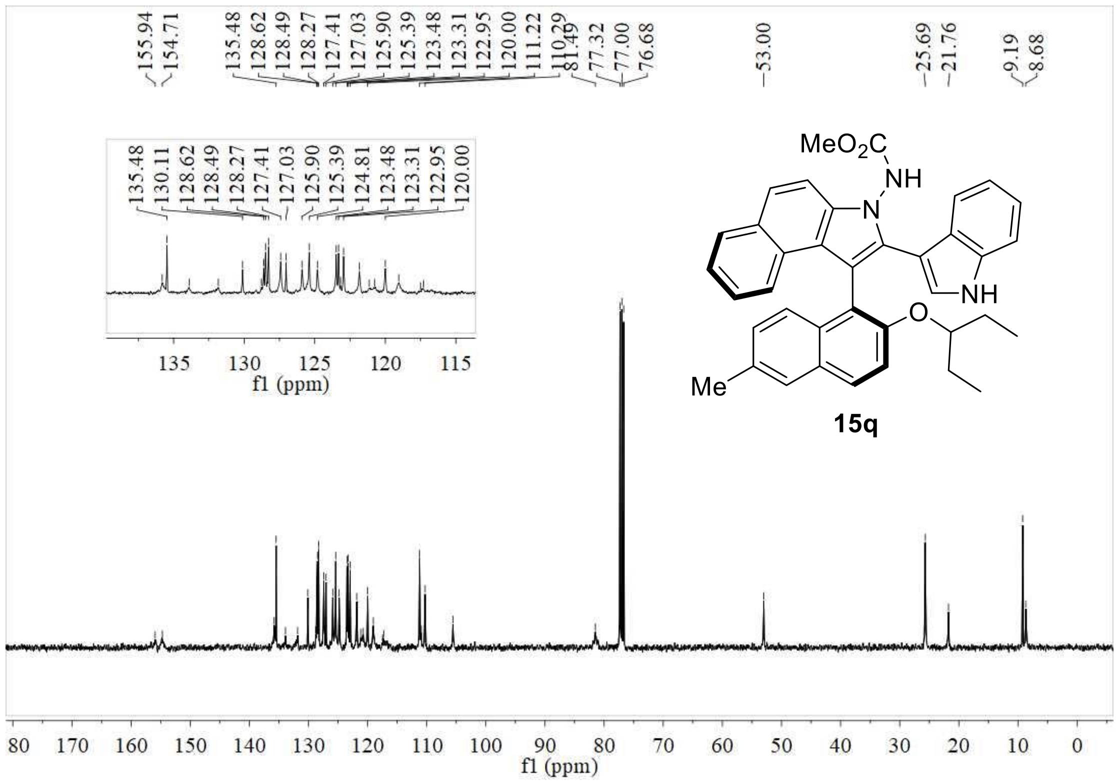
155.94 154.71 135.48 128.62 128.49 128.27 127.41 127.03 125.90 125.39 123.48 123.31 122.95 120.00 111.22 110.29 81.49 77.32 77.00 76.68 53.00 25.69 21.76 9.19 8.68
135.48 130.11 128.62 128.49 128.27 127.41 127.03 125.90 125.39 124.81 123.48 123.31 122.95 120.00
135 130 125 120 115
f1 (ppm)
MeO2C
NH
N
NH
O
Me
15q
80 170 160 150 140 130 120 110 100 90 80 70 60 50 40 30 20 10 0
f1 (ppm)

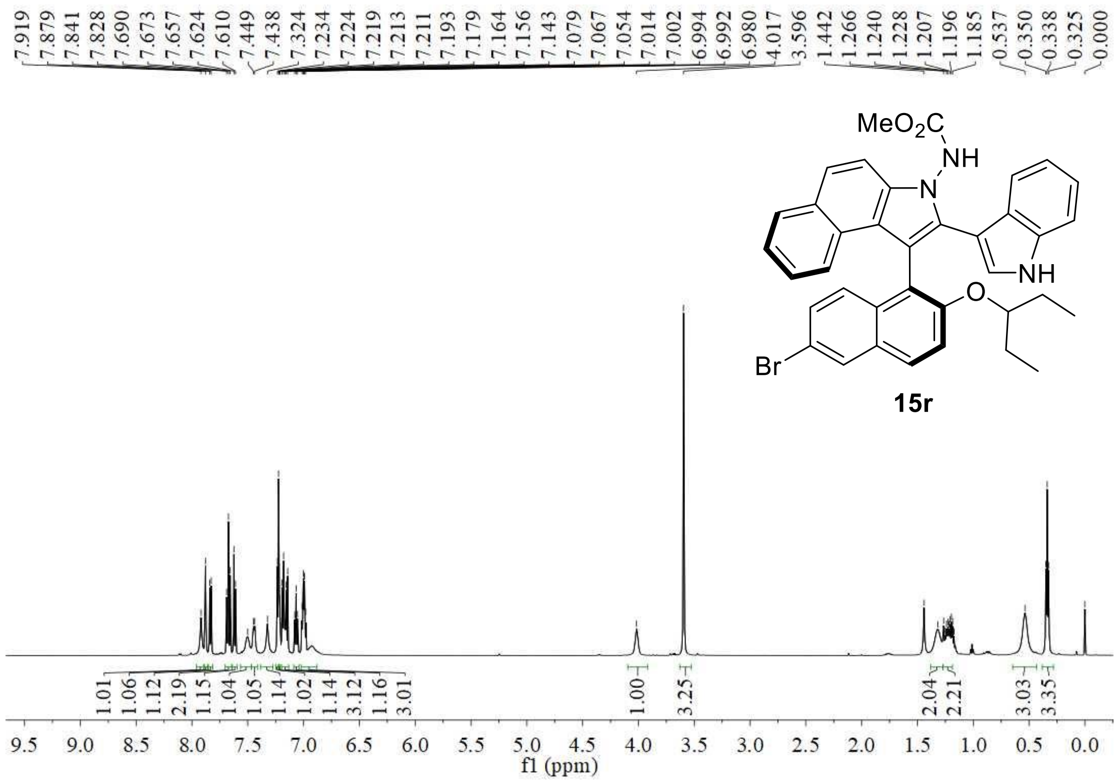
7.919
7.879
7.841
7.828
7.690
7.673
7.657
7.624
7.610
7.449
7.438
7.324
7.234
7.224
7.219
7.213
7.211
7.193
7.179
7.164
7.156
7.143
7.079
7.067
7.054
7.014
7.002
6.994
6.992
6.980
4.017
3.596
1.442
1.266
1.240
1.228
1.207
1.196
1.185
0.537
0.350
0.338
0.325
0.000
MeO2C
NH
N
NH
O
Br
15r
1.01
1.06
1.12
2.19
1.15
1.04
1.05
1.14
1.02
1.14
3.12
1.16
3.01
1.00
3.25
2.04
2.21
3.03
3.35
f1 (ppm)

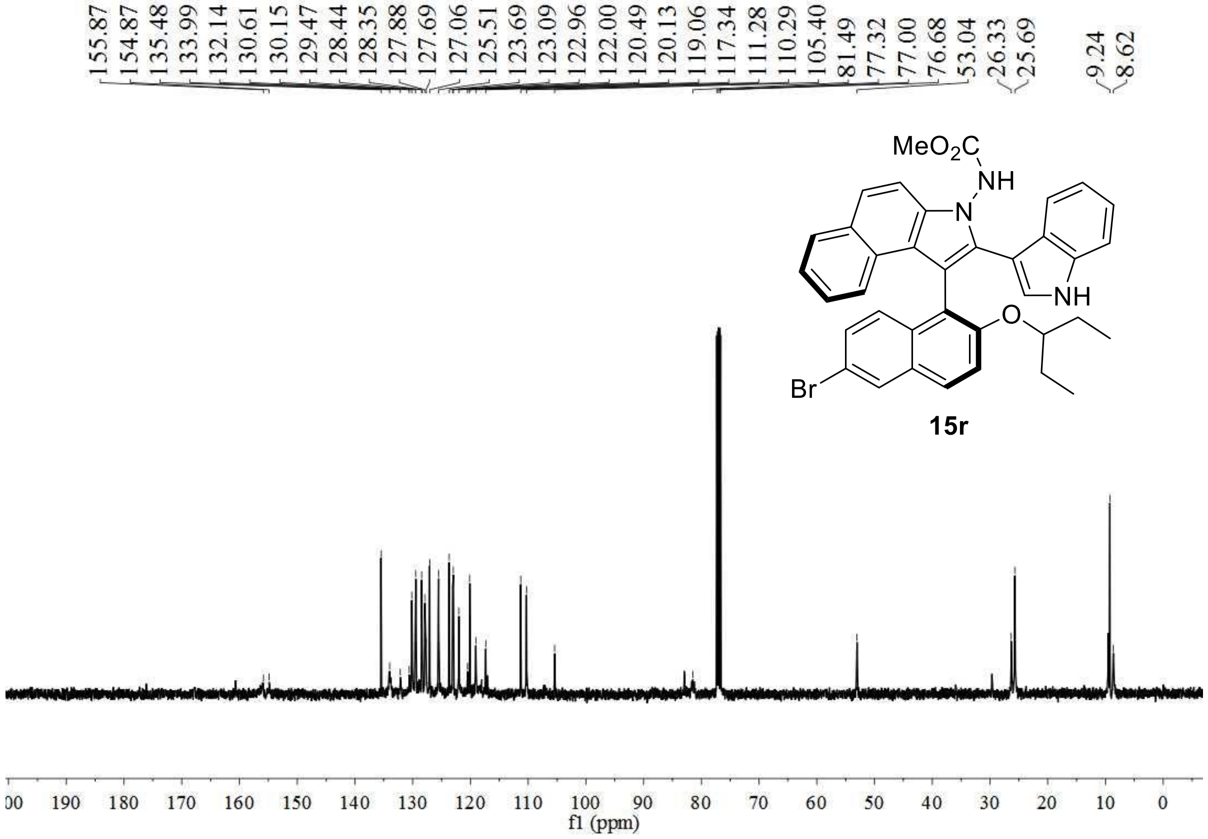
155.87
154.87
135.48
133.99
132.14
130.61
130.15
129.47
128.44
128.35
127.88
127.69
127.06
125.51
123.69
123.09
122.96
122.00
120.49
120.13
119.06
117.34
111.28
110.29
105.40
81.49
77.32
77.00
76.68
53.04
26.33
25.69
9.24
8.62
MeO2C
NH
N
NH
O
Br
15r
f1 (ppm)

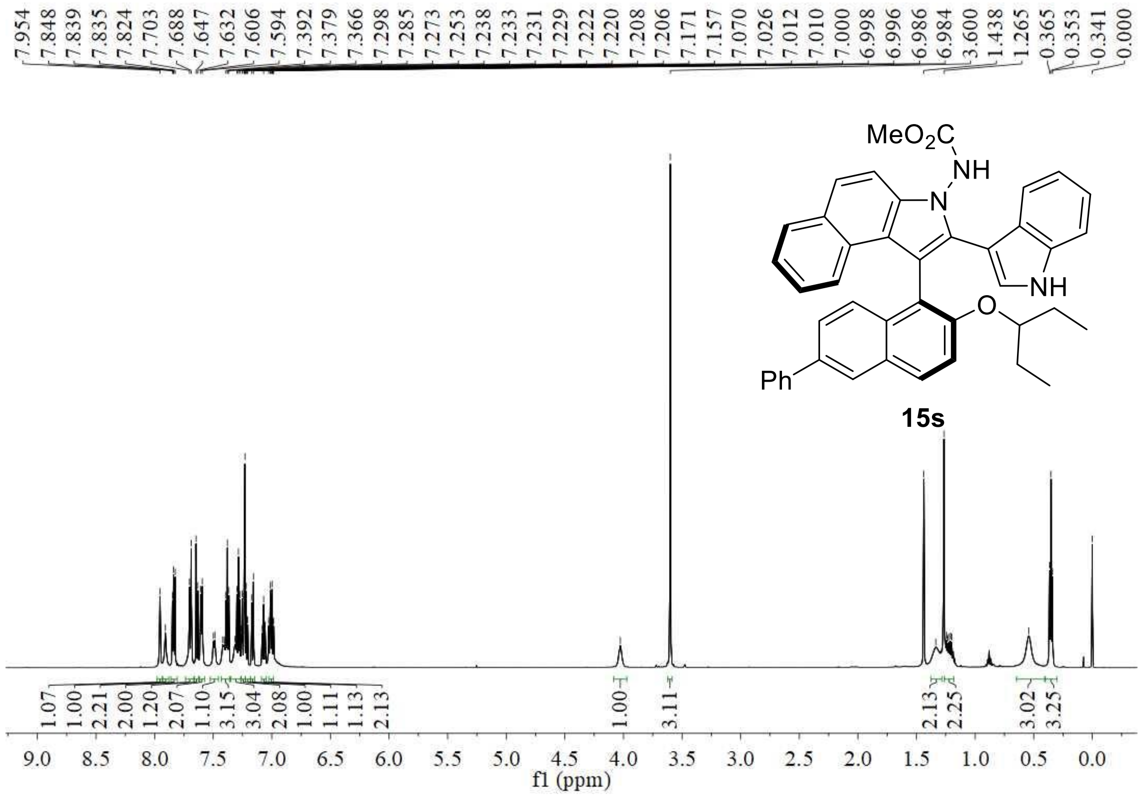
7.954 7.848 7.839 7.835 7.824 7.703 7.688 7.647 7.632 7.606 7.594 7.392 7.379 7.366 7.298 7.285 7.273 7.253 7.238 7.233 7.231 7.229 7.222 7.220 7.208 7.206 7.171 7.157 7.070 7.026 7.012 7.010 7.000 6.998 6.996 6.986 6.984 3.600 1.438 1.265 0.365 0.353 0.341 0.000
MeO2C
NH
N
NH
O
Ph
15s
1.07 1.00 2.21 2.00 1.20 2.07 1.10 3.15 3.04 2.08 1.00 1.11 1.13 2.13
1.00
3.11
2.13 2.25
3.02 3.25
9.0 8.5 8.0 7.5 7.0 6.5 6.0 5.5 5.0 4.5 4.0 3.5 3.0 2.5 2.0 1.5 1.0 0.5 0.0
f1 (ppm)

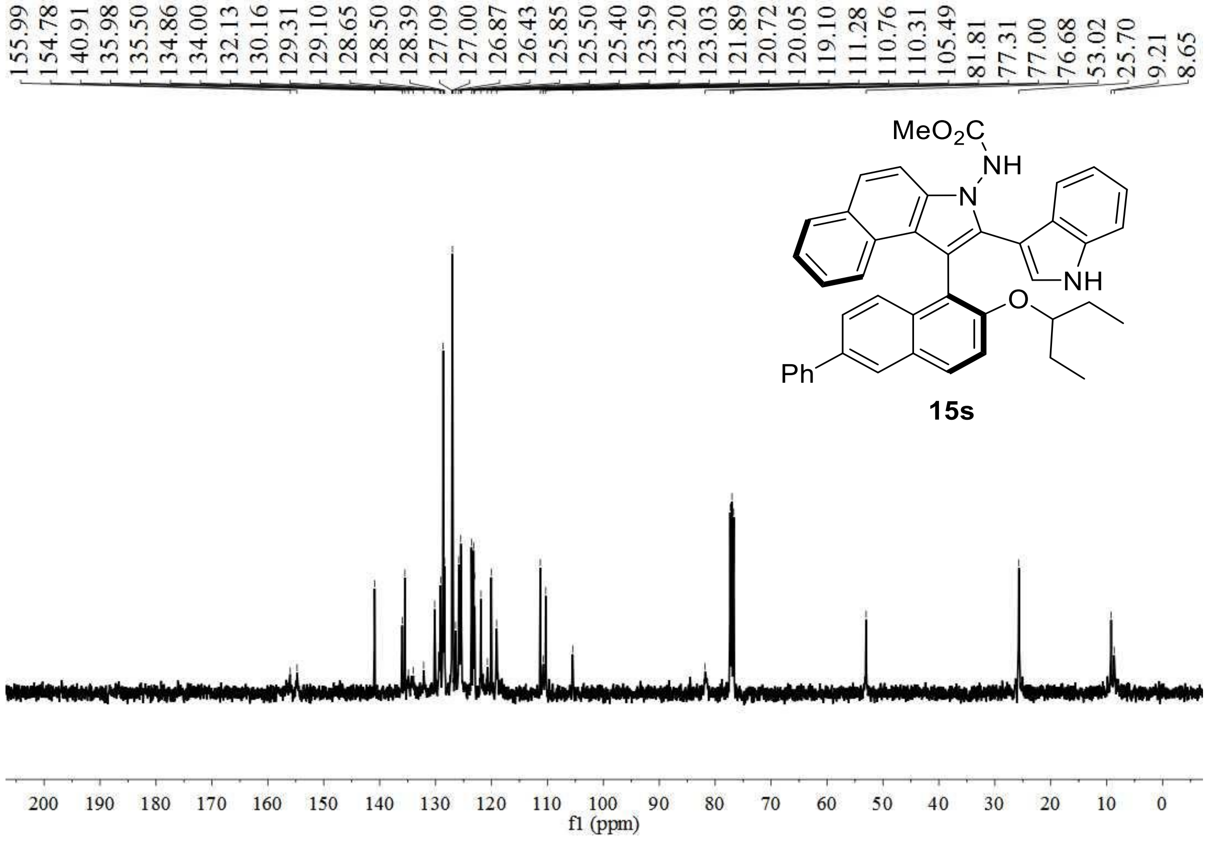
155.99 154.78 140.91 135.98 135.50 134.86 134.00 132.13 130.16 129.31 129.10 128.65 128.50 128.39 127.09 127.00 126.87 126.43 125.85 125.50 125.40 123.59 123.20 123.03 121.89 120.72 120.05 119.10 111.28 110.76 110.31 105.49 81.81 77.31 77.00 76.68 53.02 25.70 9.21 8.65
MeO2C
NH
N
NH
O
Ph
15s
200 190 180 170 160 150 140 130 120 110 100 90 80 70 60 50 40 30 20 10 0
f1 (ppm)

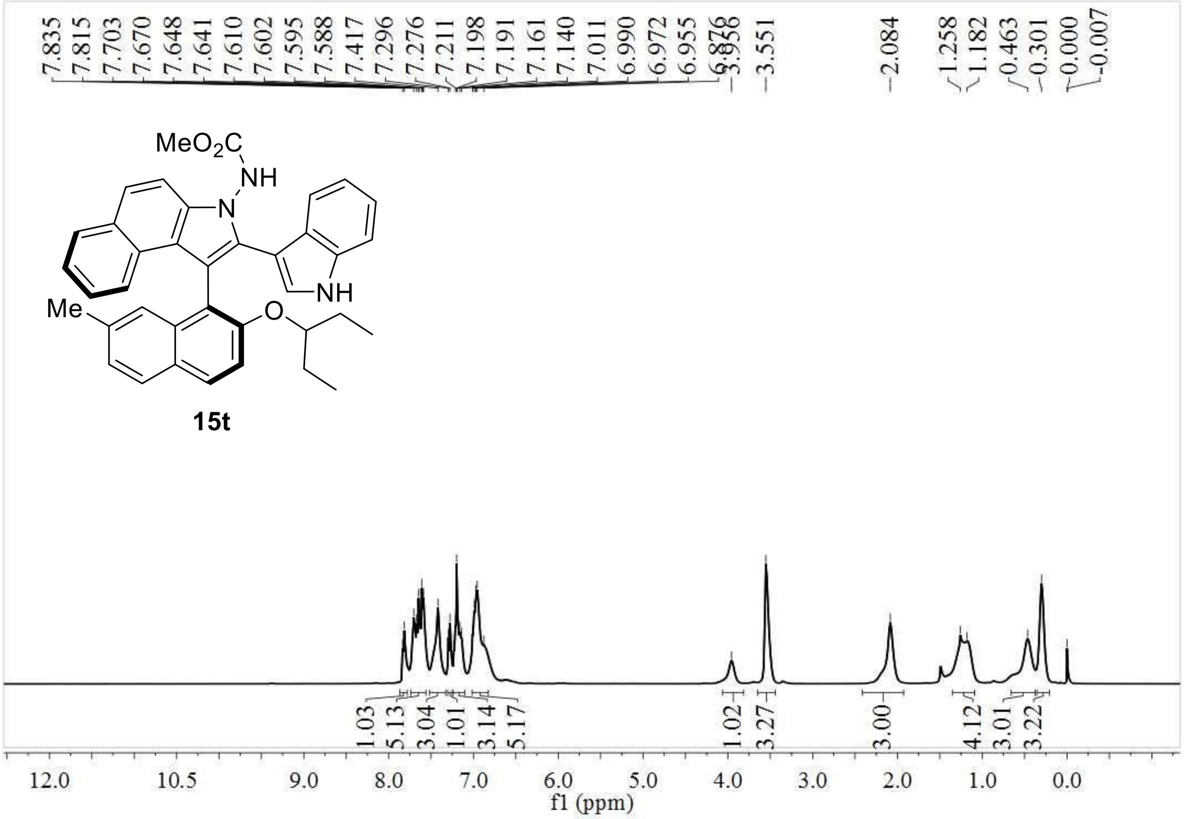
7.835
7.815
7.703
7.670
7.648
7.641
7.610
7.602
7.595
7.588
7.417
7.296
7.276
7.211
7.198
7.191
7.161
7.140
7.011
6.990
6.972
6.955
6.876
3.956
3.551
2.084
1.258
1.182
0.463
0.301
0.000
-0.007
MeO2C
NH
N
NH
Me
O
15t
1.03
5.13
3.04
1.01
3.14
5.17
1.02
3.27
3.00
4.12
3.01
3.22
12.0 10.5 9.0 8.0 7.0 6.0 5.0 4.0 3.0 2.0 1.0 0.0
f1 (ppm)

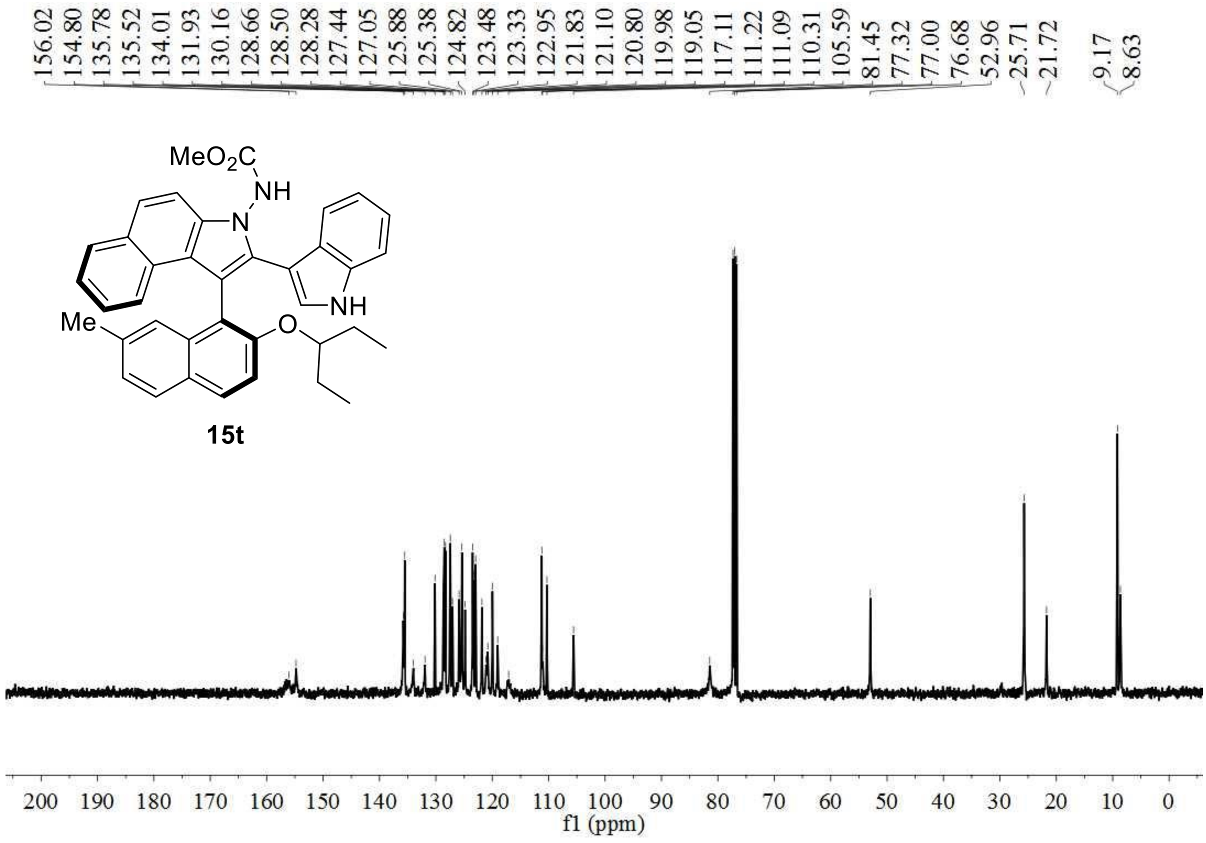
156.02
154.80
135.78
135.52
134.01
131.93
130.16
128.66
128.50
128.28
127.44
127.05
125.88
125.38
124.82
123.48
123.33
122.95
121.83
121.10
120.80
119.98
119.05
117.11
111.22
111.09
110.31
105.59
81.45
77.32
77.00
76.68
52.96
25.71
21.72
9.17
8.63
MeO2C
NH
N
NH
Me
O
15t
200 190 180 170 160 150 140 130 120 110 100 90 80 70 60 50 40 30 20 10 0
f1 (ppm)

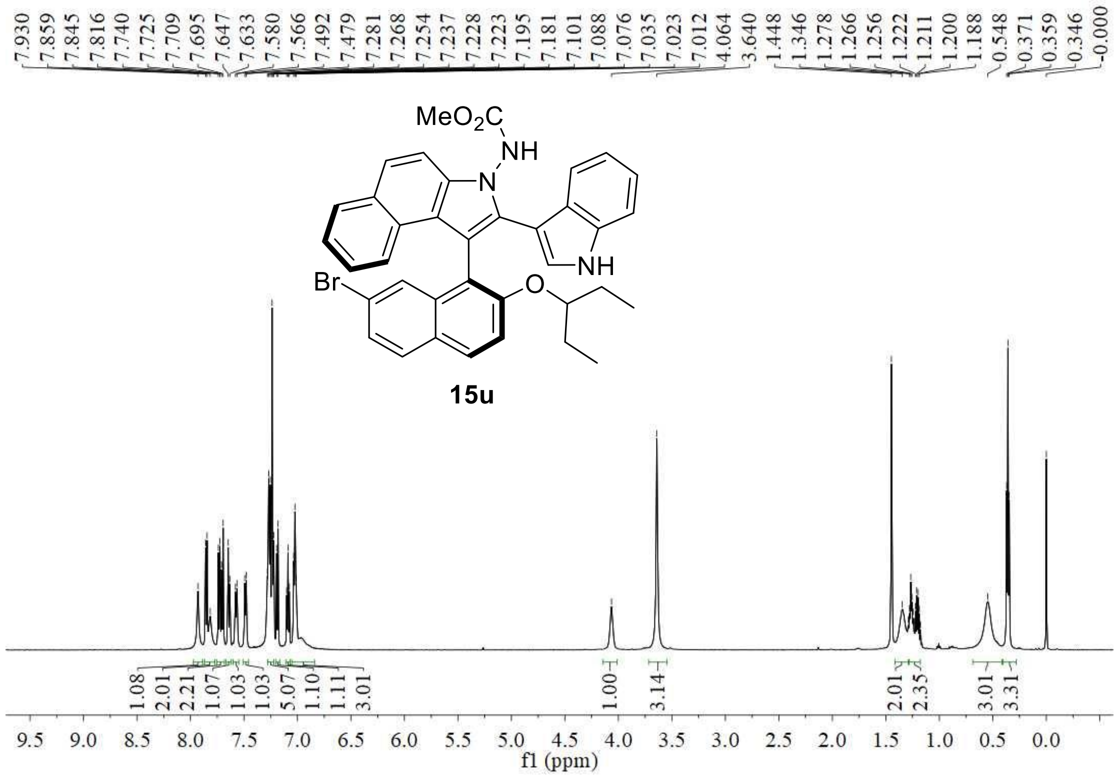
7.930
7.859
7.845
7.816
7.740
7.725
7.709
7.695
7.647
7.633
7.580
7.566
7.492
7.479
7.281
7.268
7.254
7.237
7.228
7.223
7.195
7.181
7.101
7.088
7.076
7.035
7.023
7.012
4.064
3.640
1.448
1.346
1.278
1.266
1.256
1.222
1.211
1.200
1.188
0.548
0.371
0.359
0.346
-0.000
MeO2C
NH
N
NH
Br
O
15u
1.08
2.01
2.21
1.07
1.03
1.03
5.07
1.10
1.11
3.01
1.00
3.14
2.01
2.35
3.01
3.31
9.5 9.0 8.5 8.0 7.5 7.0 6.5 6.0 5.5 5.0 4.5 4.0 3.5 3.0 2.5 2.0 1.5 1.0 0.5 0.0
f1 (ppm)

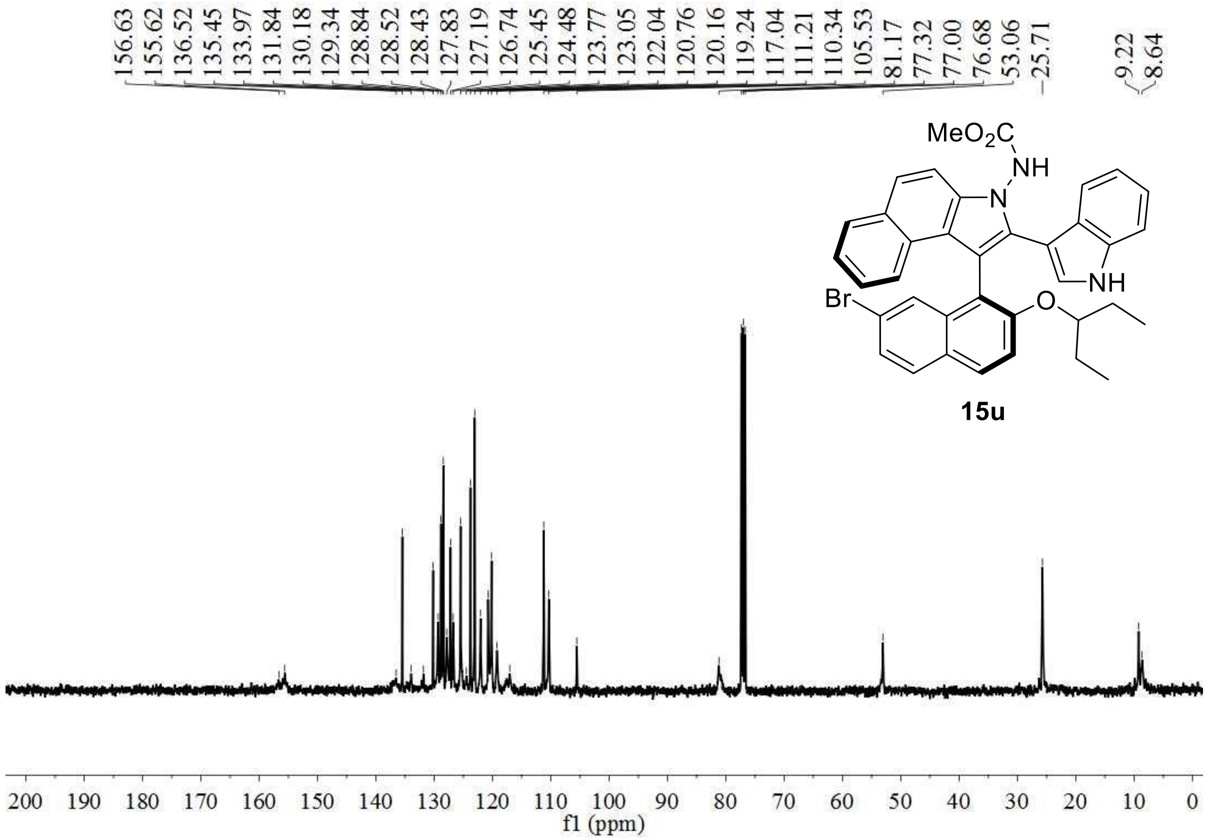
156.63
155.62
136.52
135.45
133.97
131.84
130.18
129.34
128.84
128.52
128.43
127.83
127.19
126.74
125.45
124.48
123.77
123.05
122.04
120.76
120.16
119.24
117.04
111.21
110.34
105.53
81.17
77.32
77.00
76.68
53.06
25.71
9.22
8.64
MeO2C
NH
N
NH
Br
O
15u
200 190 180 170 160 150 140 130 120 110 100 90 80 70 60 50 40 30 20 10 0
f1 (ppm)

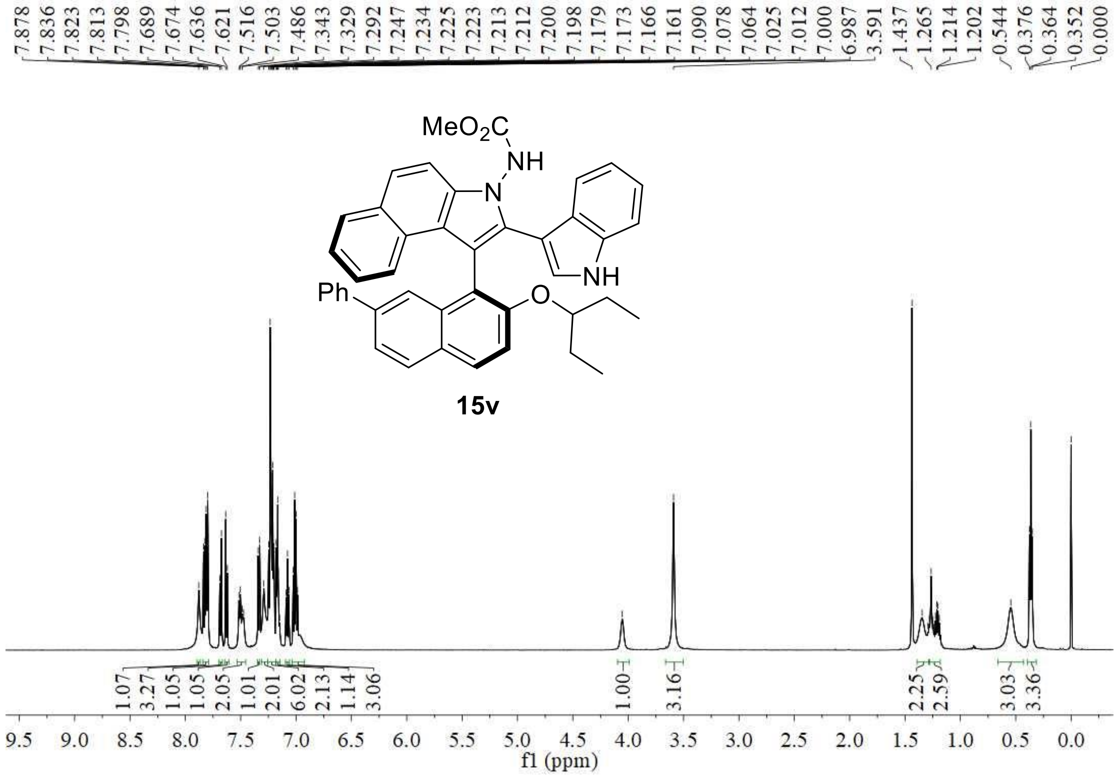
7.878 7.836 7.823 7.813 7.798 7.689 7.674 7.636 7.621 7.516 7.503 7.486 7.343 7.329 7.292 7.247 7.234 7.225 7.223 7.213 7.212 7.200 7.198 7.179 7.173 7.166 7.161 7.090 7.078 7.064 7.025 7.012 7.000 6.987 3.591 1.437 1.265 1.214 1.202 0.544 0.376 0.364 0.352 0.000
MeO2C
NH
N
NH
Ph
O
15v
1.07 3.27 1.05 1.05 2.05 1.01 2.01 6.02 2.13 1.14 3.06 1.00 3.16 2.25 2.59 3.03 3.36
9.5 9.0 8.5 8.0 7.5 7.0 6.5 6.0 5.5 5.0 4.5 4.0 3.5 3.0 2.5 2.0 1.5 1.0 0.5 0.0
f1 (ppm)

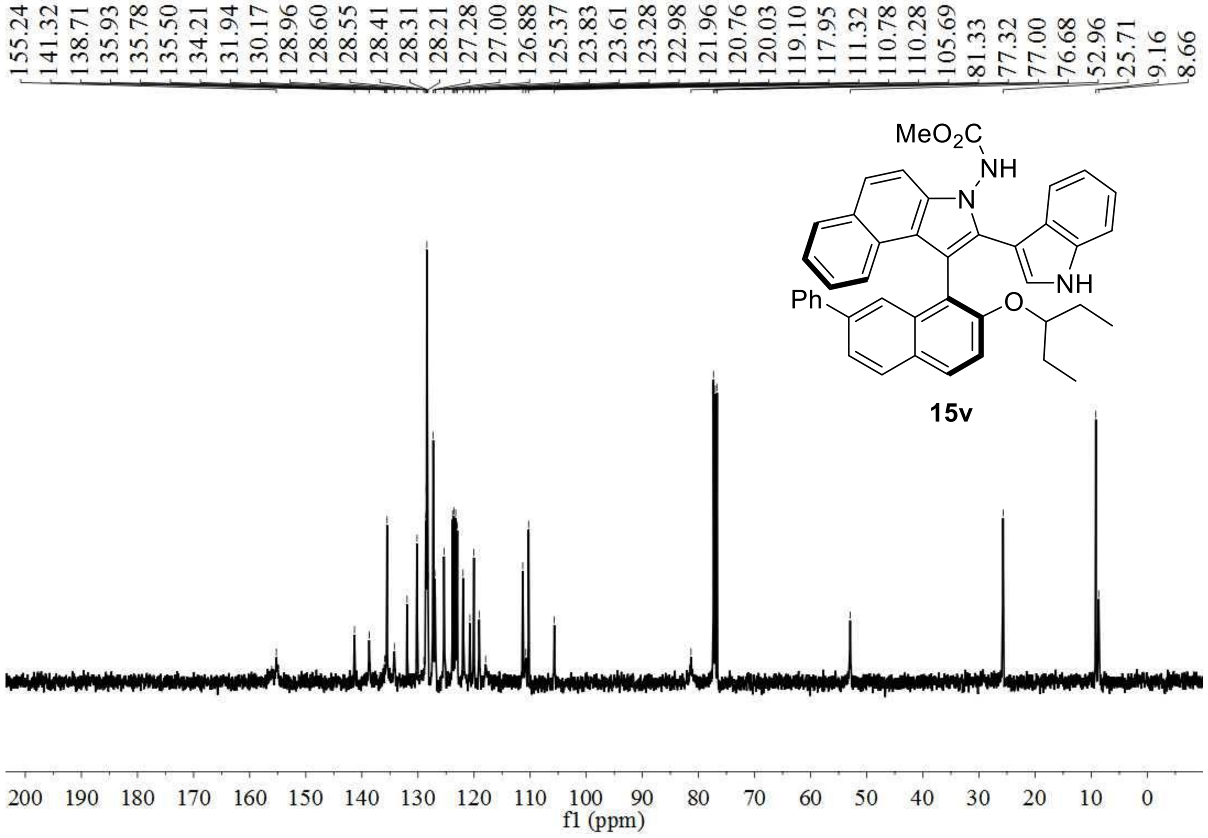
155.24 141.32 138.71 135.93 135.78 135.50 134.21 131.94 130.17 128.96 128.60 128.55 128.41 128.31 128.21 127.28 127.00 126.88 125.37 123.83 123.61 123.28 122.98 121.96 120.76 120.03 119.10 117.95 111.32 110.78 110.28 105.69 81.33 77.32 77.00 76.68 52.96 25.71 9.16 8.66
MeO2C
NH
N
NH
Ph
O
15v
200 190 180 170 160 150 140 130 120 110 100 90 80 70 60 50 40 30 20 10 0
f1 (ppm)

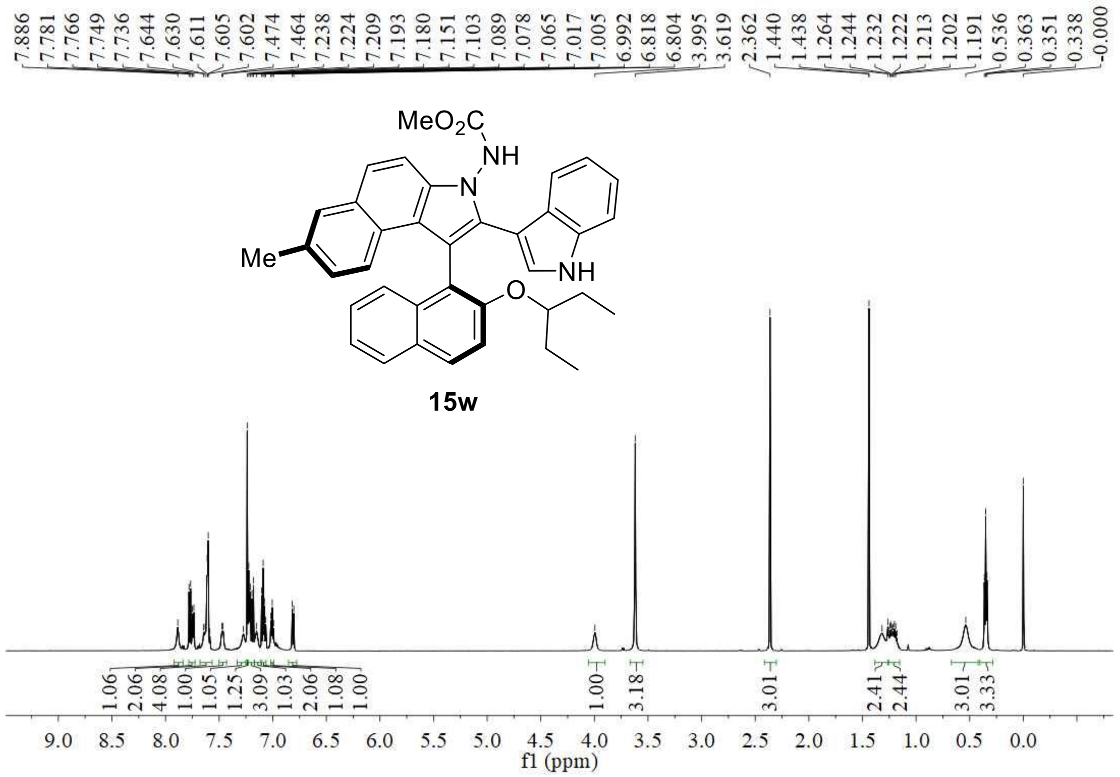
7.886
7.781
7.766
7.749
7.736
7.644
7.630
7.611
7.605
7.602
7.474
7.464
7.238
7.224
7.209
7.193
7.180
7.151
7.103
7.089
7.078
7.065
7.017
7.005
6.992
6.818
6.804
3.995
3.619
2.362
1.440
1.438
1.264
1.244
1.232
1.222
1.213
1.202
1.191
0.536
0.363
0.351
0.338
-0.000
MeO2C
NH
N
Me
NH
O
1.06
2.06
4.08
1.00
1.05
1.25
3.09
1.03
2.06
1.08
1.00
1.00
3.18
3.01
2.41
2.44
3.01
3.33
f1 (ppm)

15w

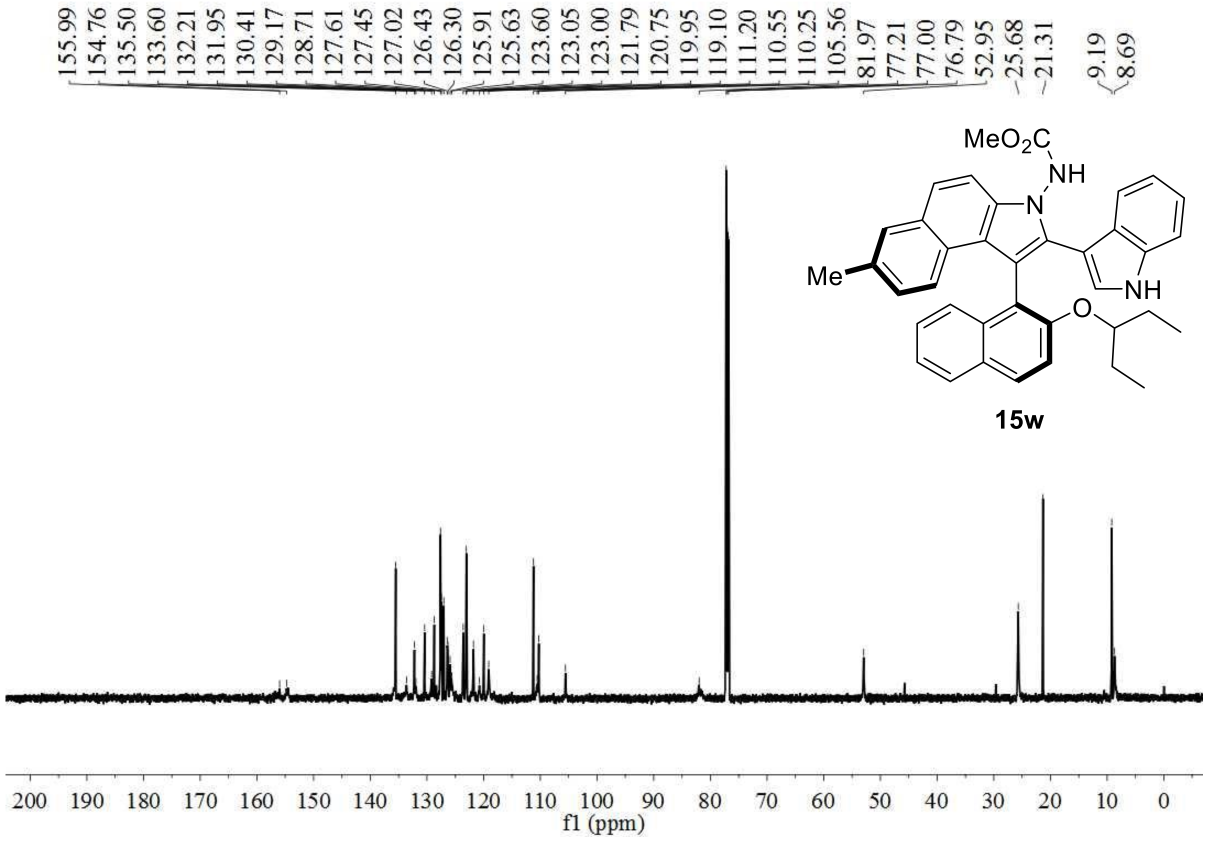
155.99
154.76
135.50
133.60
132.21
131.95
130.41
129.17
128.71
127.61
127.45
127.02
126.43
126.30
125.91
125.63
123.60
123.05
123.00
121.79
120.75
119.95
119.10
111.20
110.55
110.25
105.56
81.97
77.21
77.00
76.79
52.95
25.68
21.31
9.19
8.69
MeO2C
NH
N
Me
NH
O
f1 (ppm)

15w

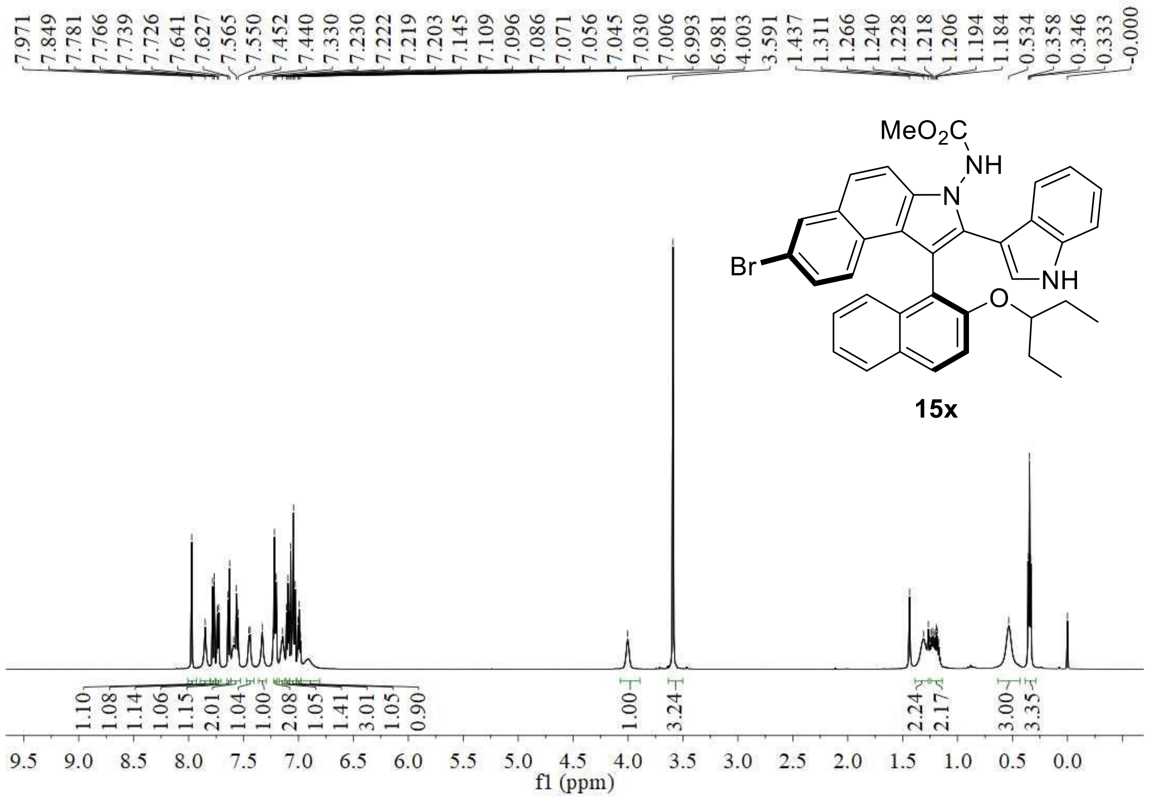
MeO2C
NH
N
Br
NH
O
15x
f1 (ppm)

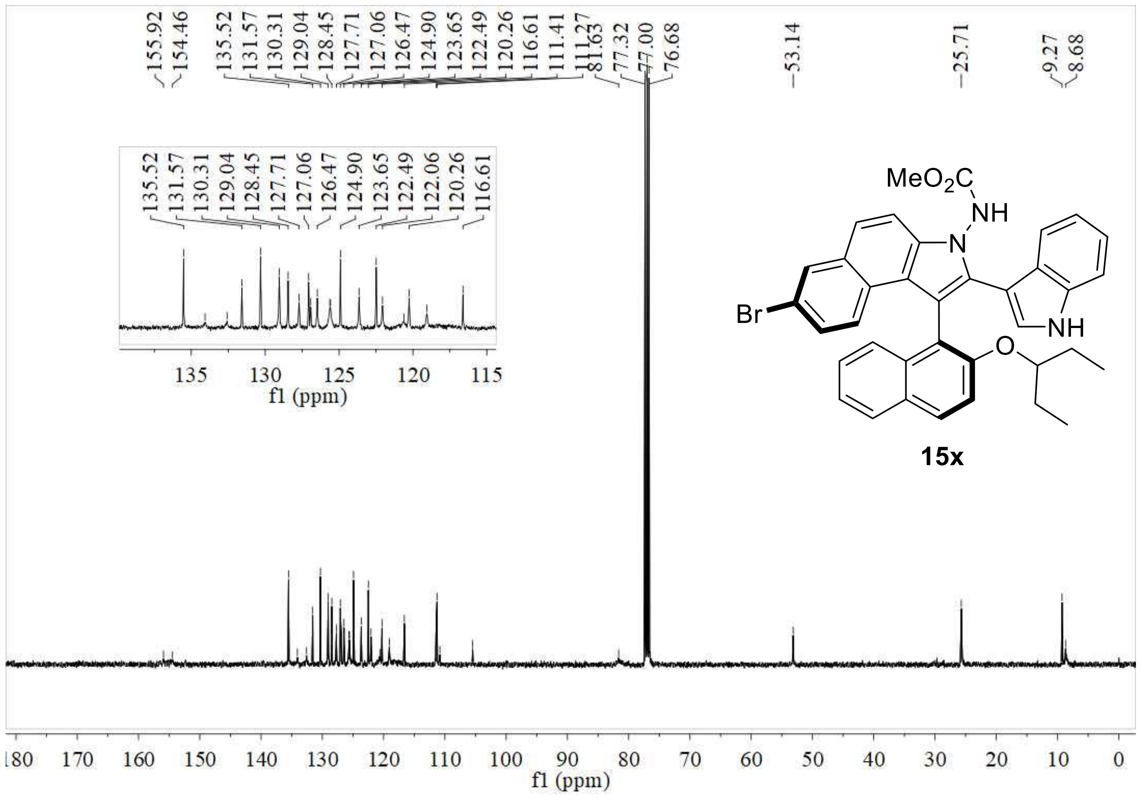
MeO2C
NH
N
Br
NH
O
15x
f1 (ppm)
f1 (ppm)

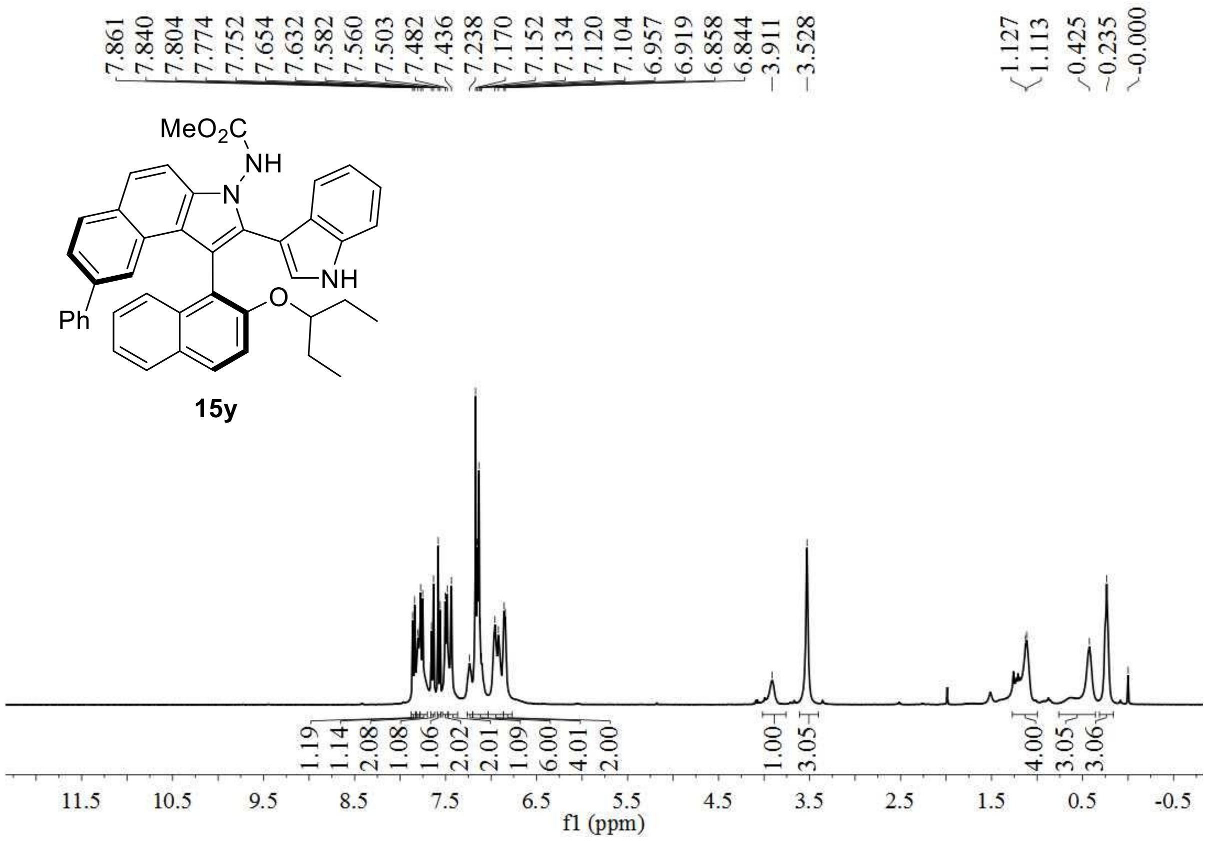
7.861
7.840
7.804
7.774
7.752
7.654
7.632
7.582
7.560
7.503
7.482
7.436
7.238
7.170
7.152
7.134
7.120
7.104
6.957
6.919
6.858
6.844
3.911
3.528
1.127
1.113
0.425
0.235
-0.000
MeO2C
NH
N
NH
O
Ph
15y
1.19
1.14
2.08
1.08
1.06
2.02
2.01
1.09
6.00
4.01
2.00
1.00
3.05
4.00
3.05
3.06
f1 (ppm)

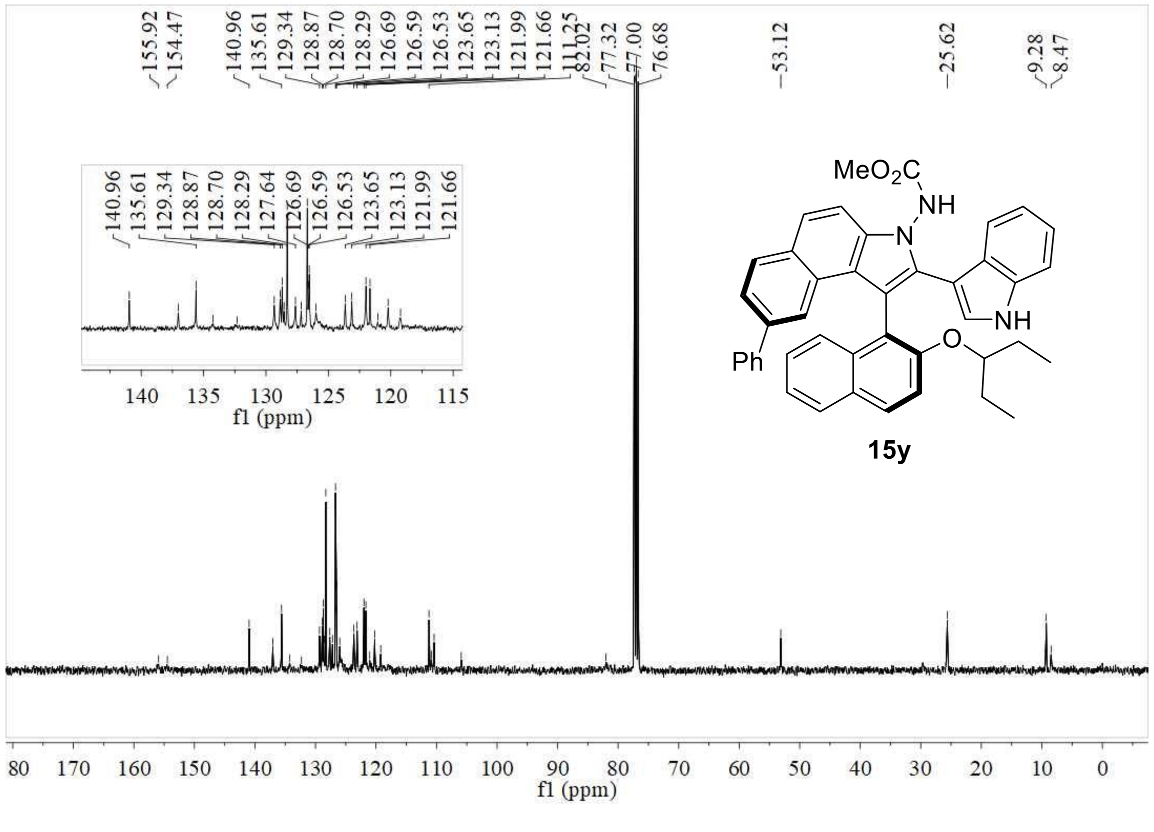
155.92
154.47
140.96
135.61
129.34
128.87
128.70
128.29
126.69
126.59
126.53
123.65
123.13
121.99
121.66
111.25
82.02
77.32
77.00
76.68
53.12
25.62
9.28
8.47
140.96
135.61
129.34
128.87
128.70
128.29
127.64
126.69
126.59
126.53
123.65
123.13
121.99
121.66
f1 (ppm)
MeO2C
NH
N
NH
O
Ph
15y
f1 (ppm)

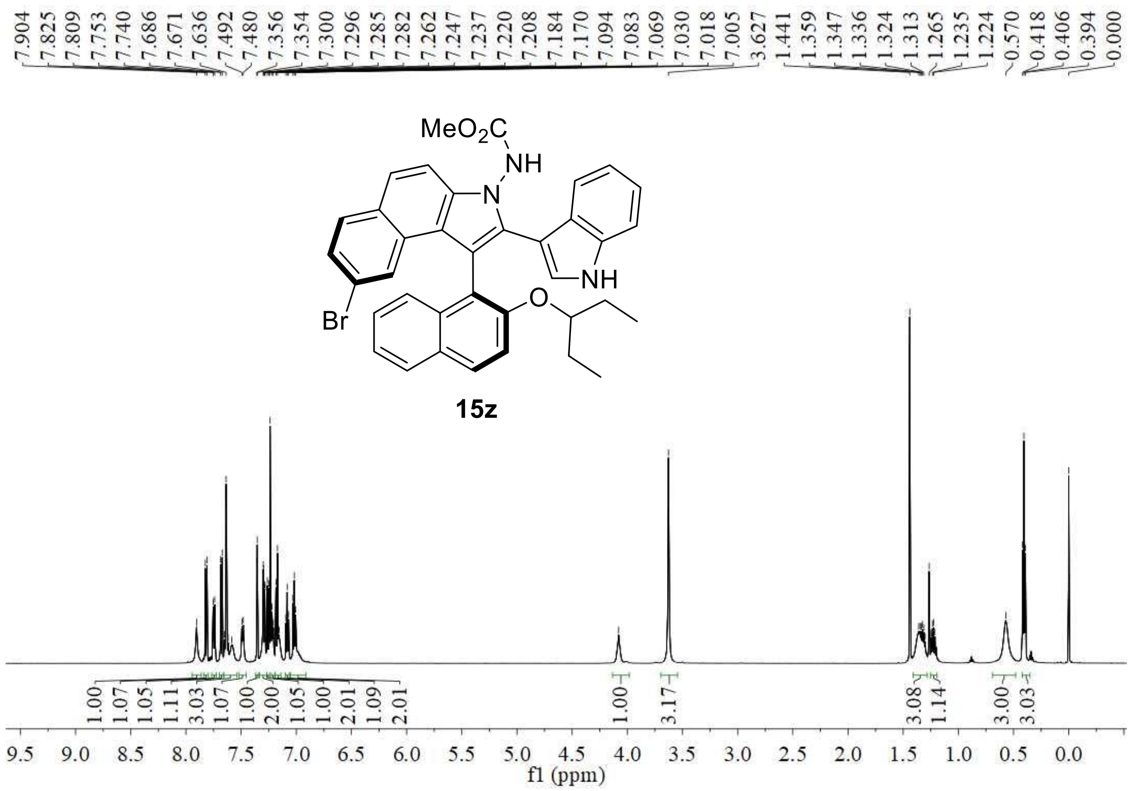
7.904 7.825 7.809 7.753 7.740 7.686 7.671 7.636 7.492 7.480 7.356 7.354 7.300 7.296 7.285 7.282 7.262 7.247 7.237 7.220 7.208 7.184 7.170 7.094 7.083 7.069 7.030 7.018 7.005 3.627 1.441 1.359 1.347 1.336 1.324 1.313 1.265 1.235 1.224 0.570 0.418 0.406 0.394 0.000
MeO2C
NH
N
NH
Br
O
15z
1.00 1.07 1.05 1.11 3.03 1.07 1.00 2.00 1.05 1.00 2.01 1.09 2.01
1.00
3.17
3.08 1.14
3.00 3.03
9.5 9.0 8.5 8.0 7.5 7.0 6.5 6.0 5.5 5.0 4.5 4.0 3.5 3.0 2.5 2.0 1.5 1.0 0.5 0.0
f1 (ppm)

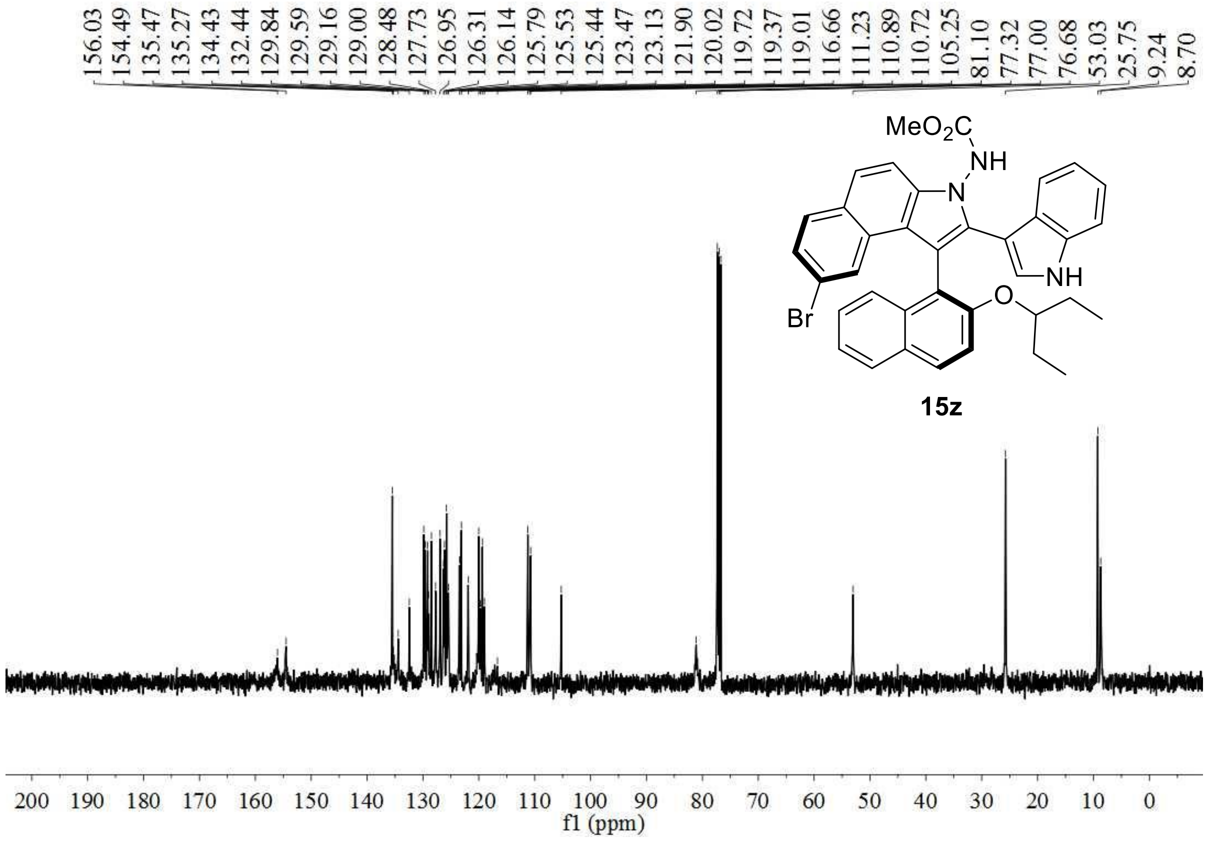
156.03 154.49 135.47 135.27 134.43 132.44 129.84 129.59 129.16 129.00 128.48 127.73 126.95 126.31 126.14 125.79 125.53 125.44 123.47 123.13 121.90 120.02 119.72 119.37 119.01 116.66 111.23 110.89 110.72 105.25 81.10 77.32 77.00 76.68 53.03 25.75 9.24 8.70
MeO2C
NH
N
NH
Br
O
15z
200 190 180 170 160 150 140 130 120 110 100 90 80 70 60 50 40 30 20 10 0
f1 (ppm)

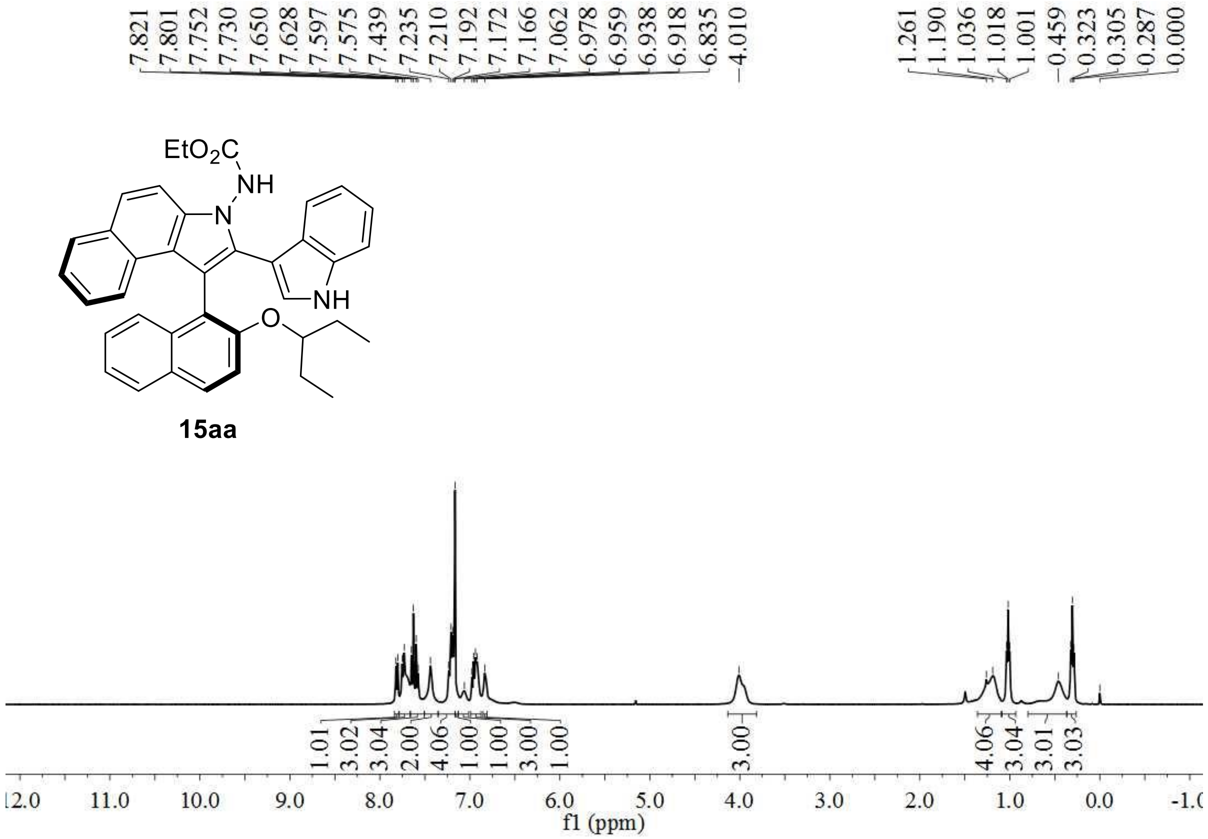
7.821
7.801
7.752
7.730
7.650
7.628
7.597
7.575
7.439
7.235
7.210
7.192
7.172
7.166
7.062
6.978
6.959
6.938
6.918
6.835
4.010
1.261
1.190
1.036
1.018
1.001
0.459
0.323
0.305
0.287
0.000
EtO2C
NH
N
NH
O
15aa
1.01
3.02
3.04
2.00
4.06
1.00
1.00
3.00
1.00
3.00
4.06
3.04
3.01
3.03
f1 (ppm)

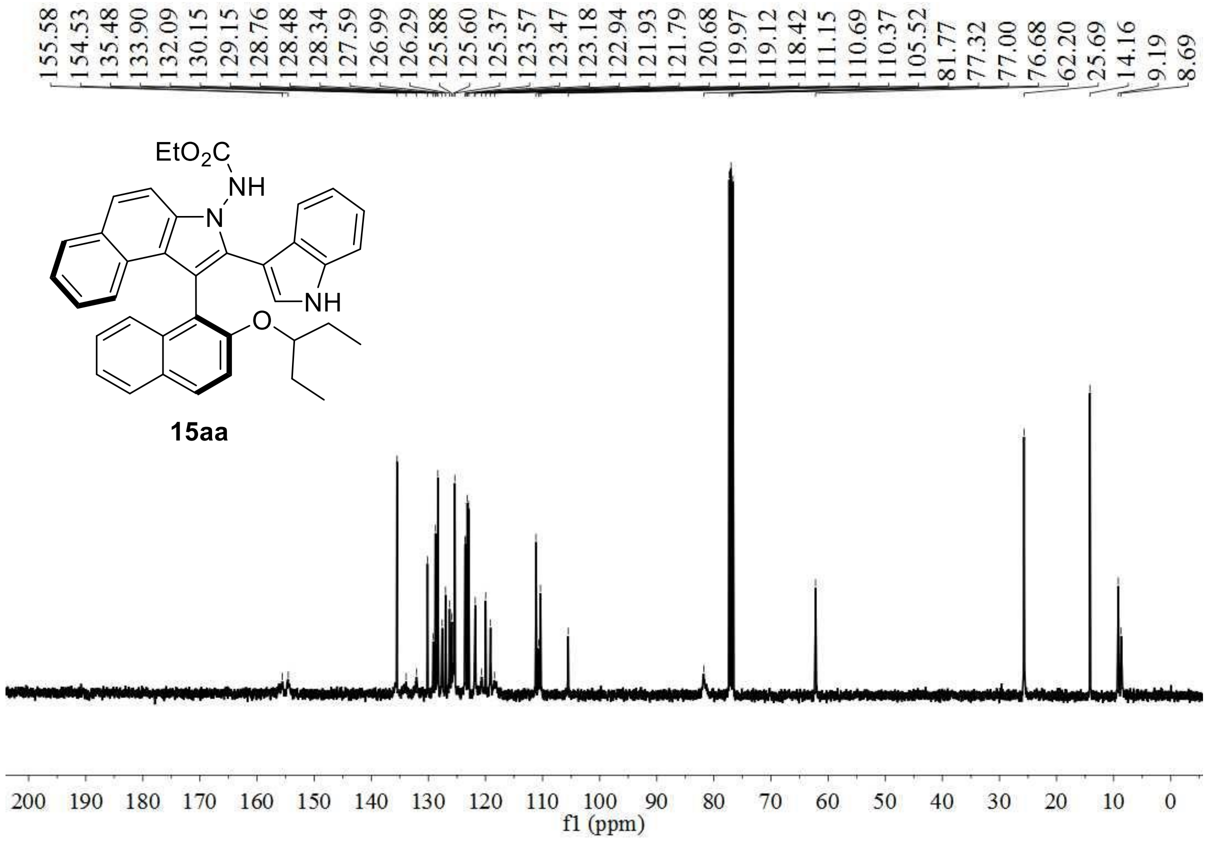
155.58
154.53
135.48
133.90
132.09
130.15
129.15
128.76
128.48
128.34
127.59
126.99
126.29
125.88
125.60
125.37
123.57
123.47
123.18
122.94
121.93
121.79
120.68
119.97
119.12
118.42
111.15
110.69
110.37
105.52
81.77
77.32
77.00
76.68
62.20
25.69
14.16
9.19
8.69
EtO2C
NH
N
NH
O
15aa
f1 (ppm)

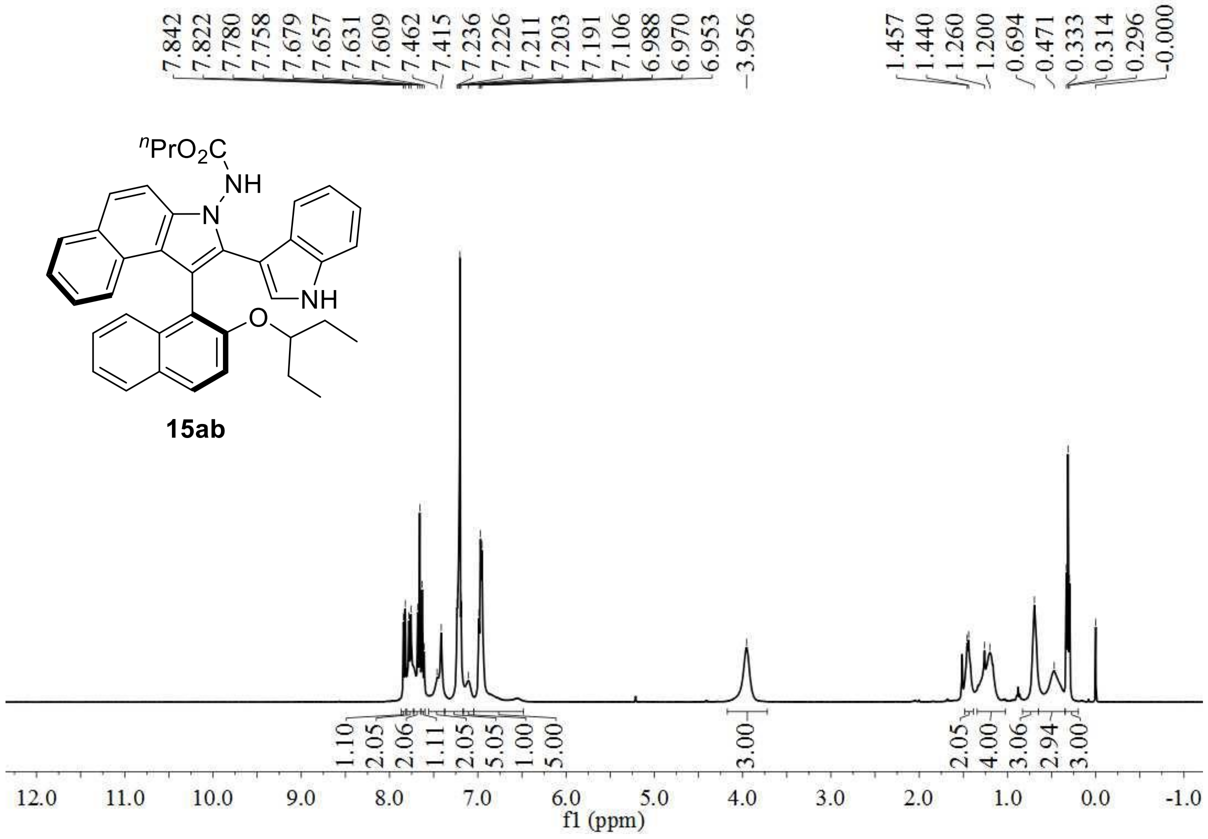
7.842
7.822
7.780
7.758
7.679
7.657
7.631
7.609
7.462
7.415
7.236
7.226
7.211
7.203
7.191
7.106
6.988
6.970
6.953
3.956
1.457
1.440
1.260
1.200
0.694
0.471
0.333
0.314
0.296
-0.000
ⁿPrO₂C
NH
N
NH
O
15ab
1.10
2.05
2.06
1.11
2.05
5.05
1.00
5.00
3.00
2.05
4.00
3.06
2.94
3.00
12.0 11.0 10.0 9.0 8.0 7.0 6.0 5.0 4.0 3.0 2.0 1.0 0.0 -1.0
f1 (ppm)

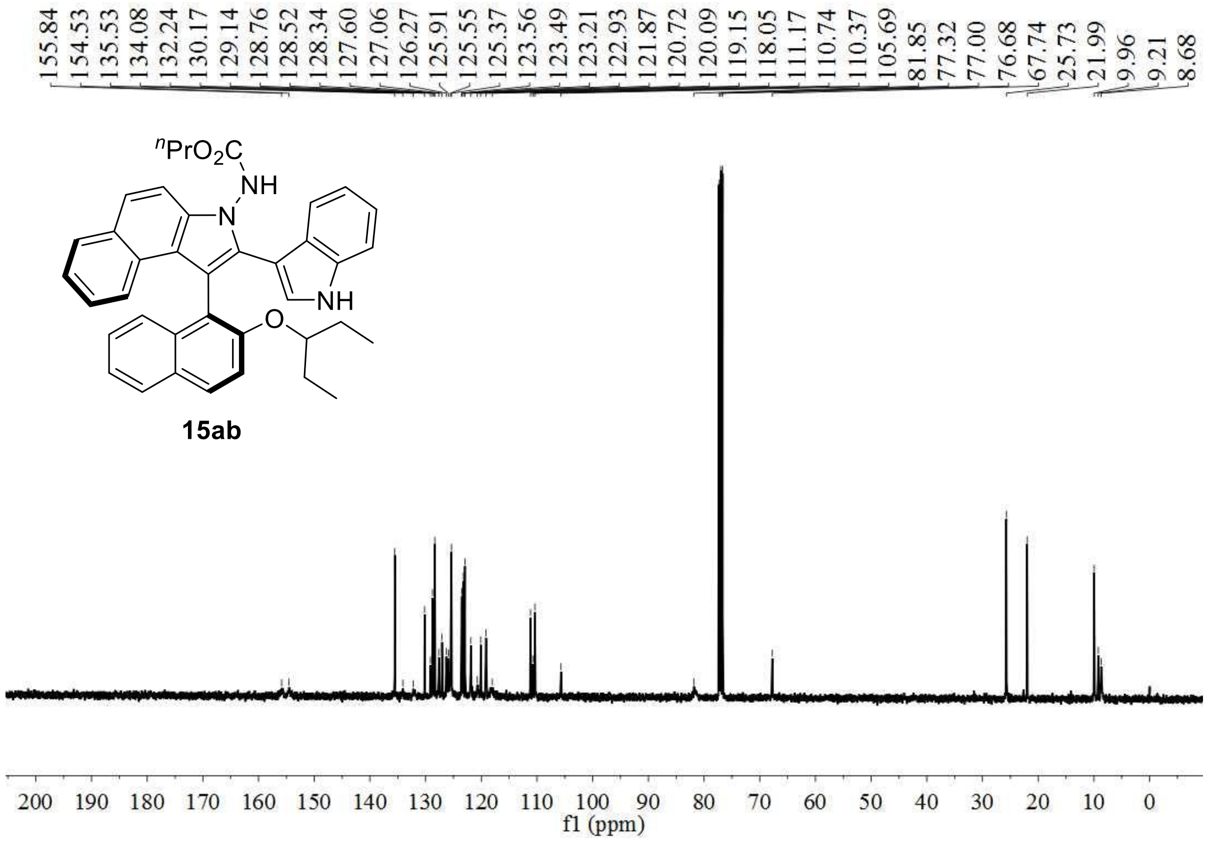
155.84
154.53
135.53
134.08
132.24
130.17
129.14
128.76
128.52
128.34
127.60
127.06
126.27
125.91
125.55
125.37
123.56
123.49
123.21
122.93
121.87
120.72
120.09
119.15
118.05
111.17
110.74
110.37
105.69
81.85
77.32
77.00
76.68
67.74
25.73
21.99
9.96
9.21
8.68
ⁿPrO₂C
NH
N
NH
O
15ab
200 190 180 170 160 150 140 130 120 110 100 90 80 70 60 50 40 30 20 10 0
f1 (ppm)

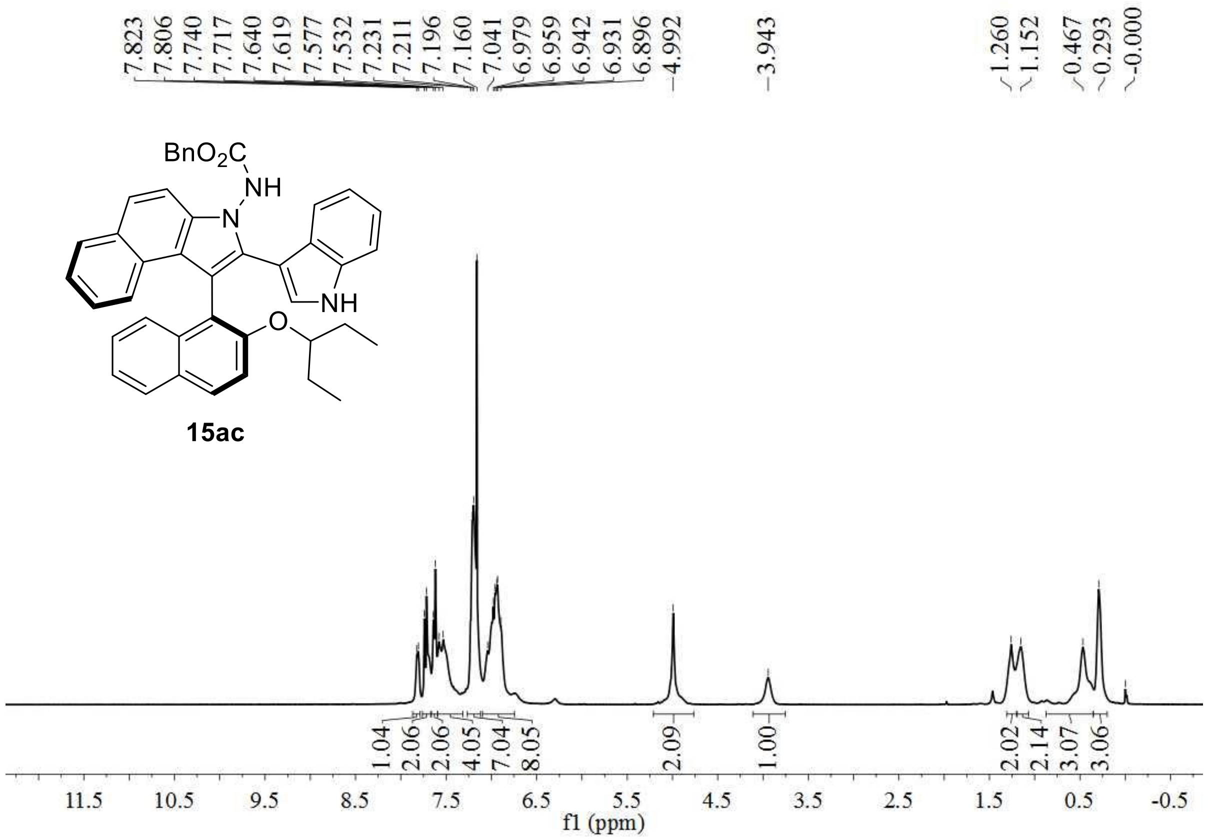
7.823
7.806
7.740
7.717
7.640
7.619
7.577
7.532
7.231
7.211
7.196
7.160
7.041
6.979
6.959
6.942
6.931
6.896
4.992
3.943
1.260
1.152
0.467
0.293
-0.000
BnO2C
NH
N
NH
O
15ac
1.04
2.06
2.06
4.05
7.04
8.05
2.09
1.00
2.02
2.14
3.07
3.06
11.5 10.5 9.5 8.5 7.5 6.5 5.5 4.5 3.5 2.5 1.5 0.5 -0.5
f1 (ppm)

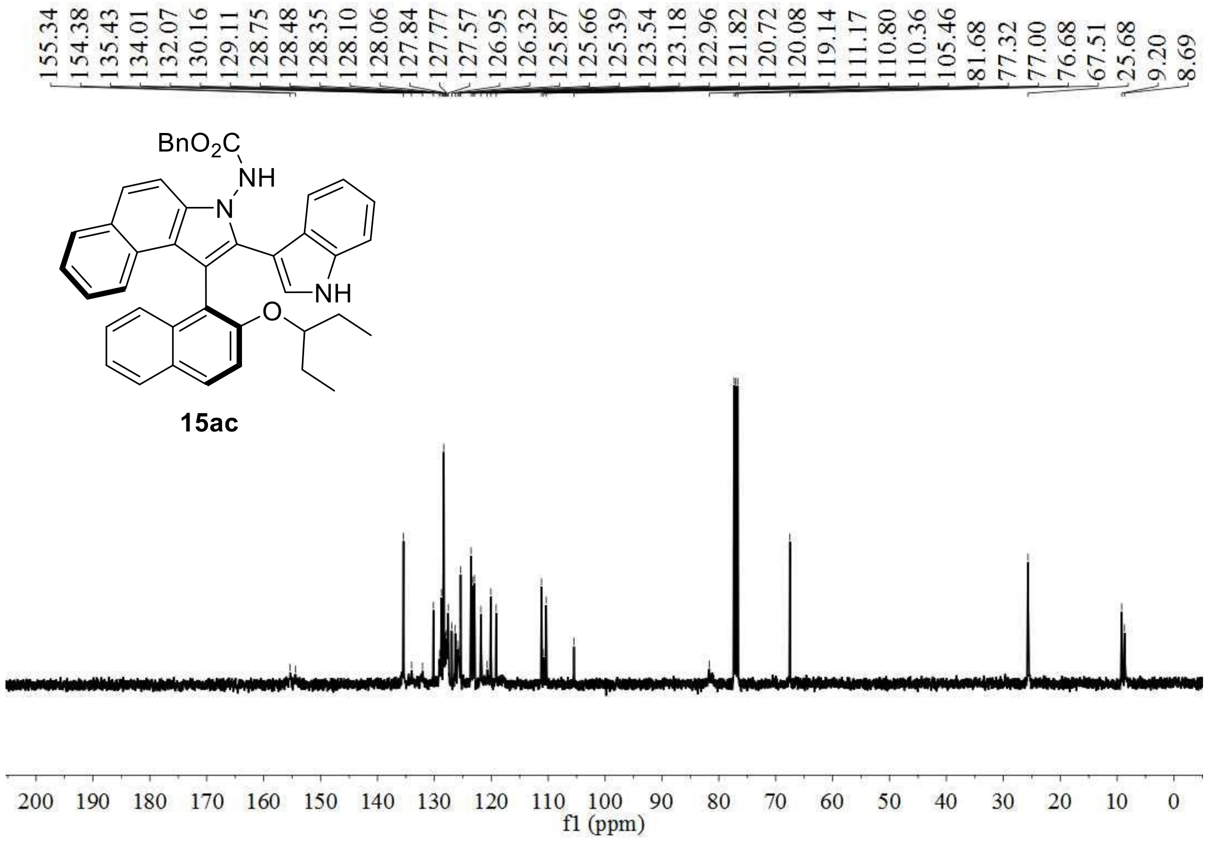
155.34
154.38
135.43
134.01
132.07
130.16
129.11
128.75
128.48
128.35
128.10
128.06
127.84
127.77
127.57
126.95
126.32
125.87
125.66
125.39
123.54
123.18
122.96
121.82
120.72
120.08
119.14
111.17
110.80
110.36
105.46
81.68
77.32
77.00
76.68
67.51
25.68
9.20
8.69
BnO2C
NH
N
NH
O
15ac
200 190 180 170 160 150 140 130 120 110 100 90 80 70 60 50 40 30 20 10 0
f1 (ppm)

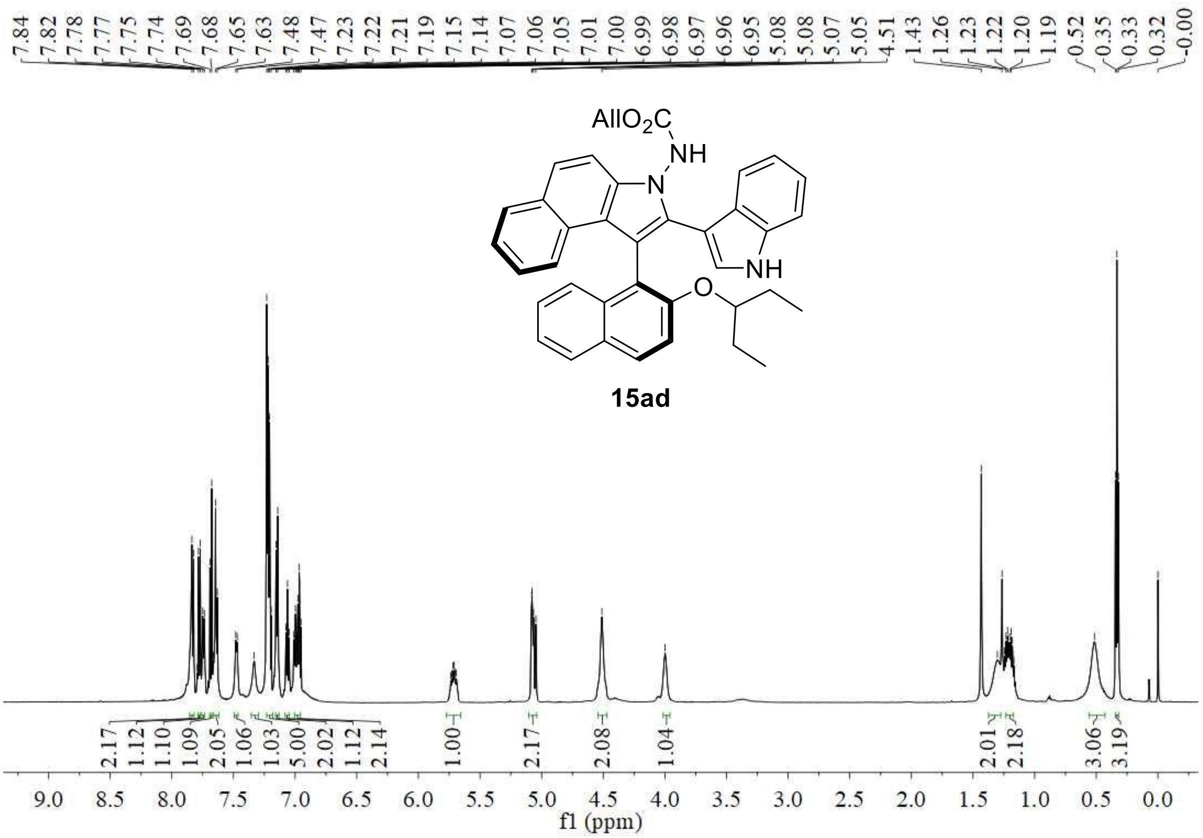
7.84 7.82 7.78 7.77 7.75 7.74 7.69 7.68 7.65 7.63 7.48 7.47 7.23 7.22 7.21 7.19 7.15 7.14 7.07 7.06 7.05 7.01 7.00 6.99 6.98 6.97 6.96 6.95 5.08 5.08 5.07 5.05 4.51 1.43 1.26 1.23 1.22 1.20 1.19 0.52 0.35 0.33 0.32 -0.00
AllO2C
NH
N
NH
O
15ad
2.17 1.12 1.10 1.09 2.05 1.06 1.03 5.00 2.02 1.12 2.14 1.00 2.17 2.08 1.04 2.01 2.18 3.06 3.19
f1 (ppm)

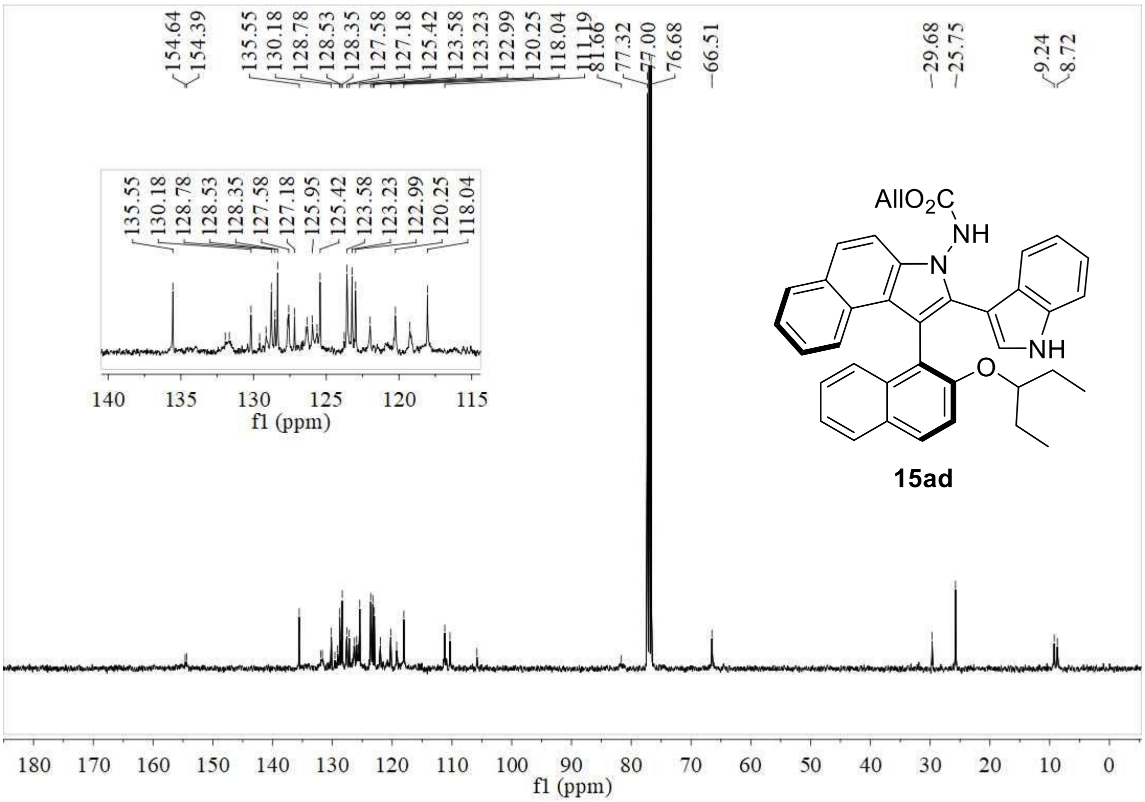
154.64 154.39 135.55 130.18 128.78 128.53 128.35 127.58 127.18 125.42 123.58 123.23 122.99 120.25 118.04 111.19 81.66 77.32 77.00 76.68 66.51 29.68 25.75 9.24 8.72
135.55 130.18 128.78 128.53 128.35 127.58 127.18 125.95 125.42 123.58 123.23 122.99 120.25 118.04
f1 (ppm)
AllO2C
NH
N
NH
O
15ad
f1 (ppm)

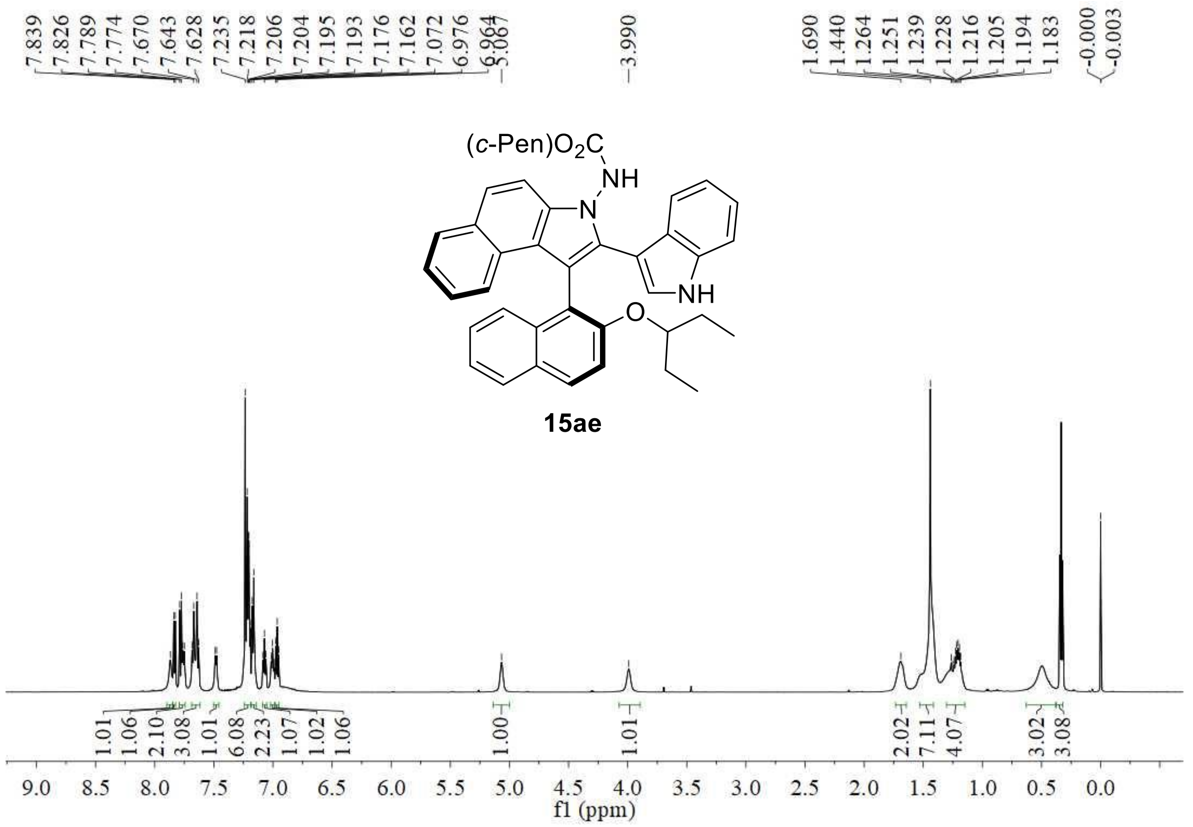
7.839
7.826
7.789
7.774
7.670
7.643
7.628
7.235
7.218
7.206
7.204
7.195
7.193
7.176
7.162
7.072
6.976
6.964
5.067
3.990
1.690
1.440
1.264
1.251
1.239
1.228
1.216
1.205
1.194
1.183
-0.000
-0.003
(c-Pen)O2C
NH
N
NH
O
15ae
1.01
1.06
2.10
3.08
1.01
6.08
2.23
1.07
1.02
1.06
1.00
1.01
2.02
7.11
4.07
3.02
3.08
9.0 8.5 8.0 7.5 7.0 6.5 6.0 5.5 5.0 4.5 4.0 3.5 3.0 2.5 2.0 1.5 1.0 0.5 0.0
f1 (ppm)

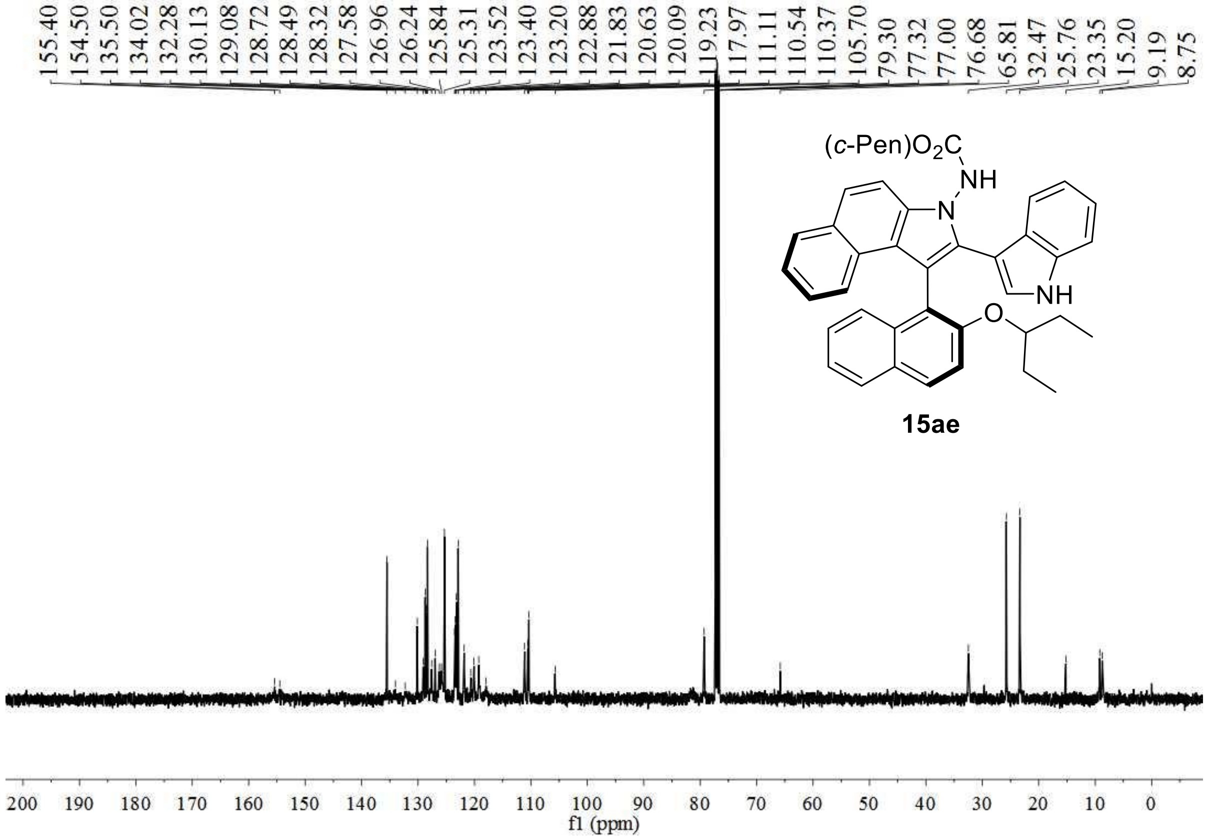
155.40
154.50
135.50
134.02
132.28
130.13
129.08
128.72
128.49
128.32
127.58
126.96
126.24
125.84
125.31
123.52
123.40
123.20
122.88
121.83
120.63
120.09
119.23
117.97
111.11
110.54
110.37
105.70
79.30
77.32
77.00
76.68
65.81
32.47
25.76
23.35
15.20
9.19
8.75
(c-Pen)O2C
NH
N
NH
O
15ae
200 190 180 170 160 150 140 130 120 110 100 90 80 70 60 50 40 30 20 10 0
f1 (ppm)

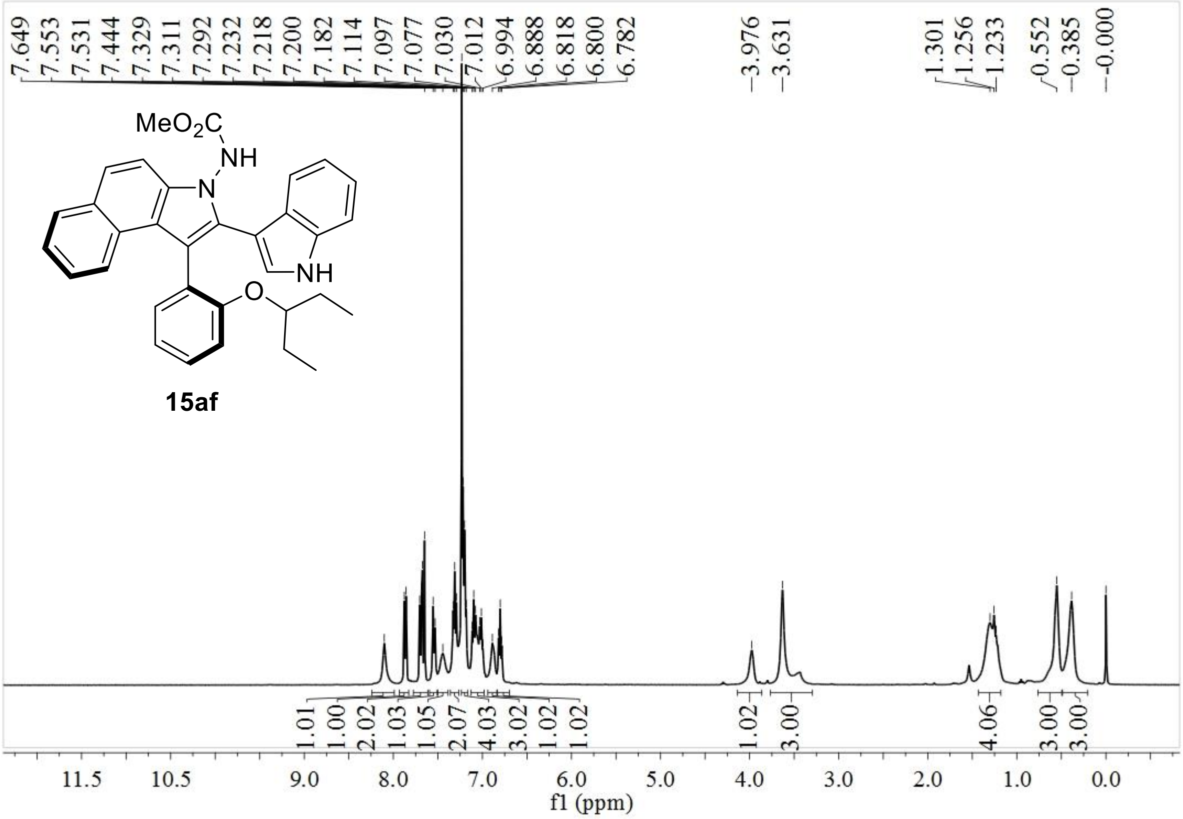
7.649
7.553
7.531
7.444
7.329
7.311
7.292
7.232
7.218
7.200
7.182
7.114
7.097
7.077
7.030
7.012
6.994
6.888
6.818
6.800
6.782
3.976
3.631
1.301
1.256
1.233
0.552
0.385
-0.000
MeO2C
NH
N
NH
O
15af
1.01
1.00
2.02
1.03
1.05
2.07
4.03
3.02
1.02
1.02
1.02
3.00
4.06
3.00
3.00
f1 (ppm)

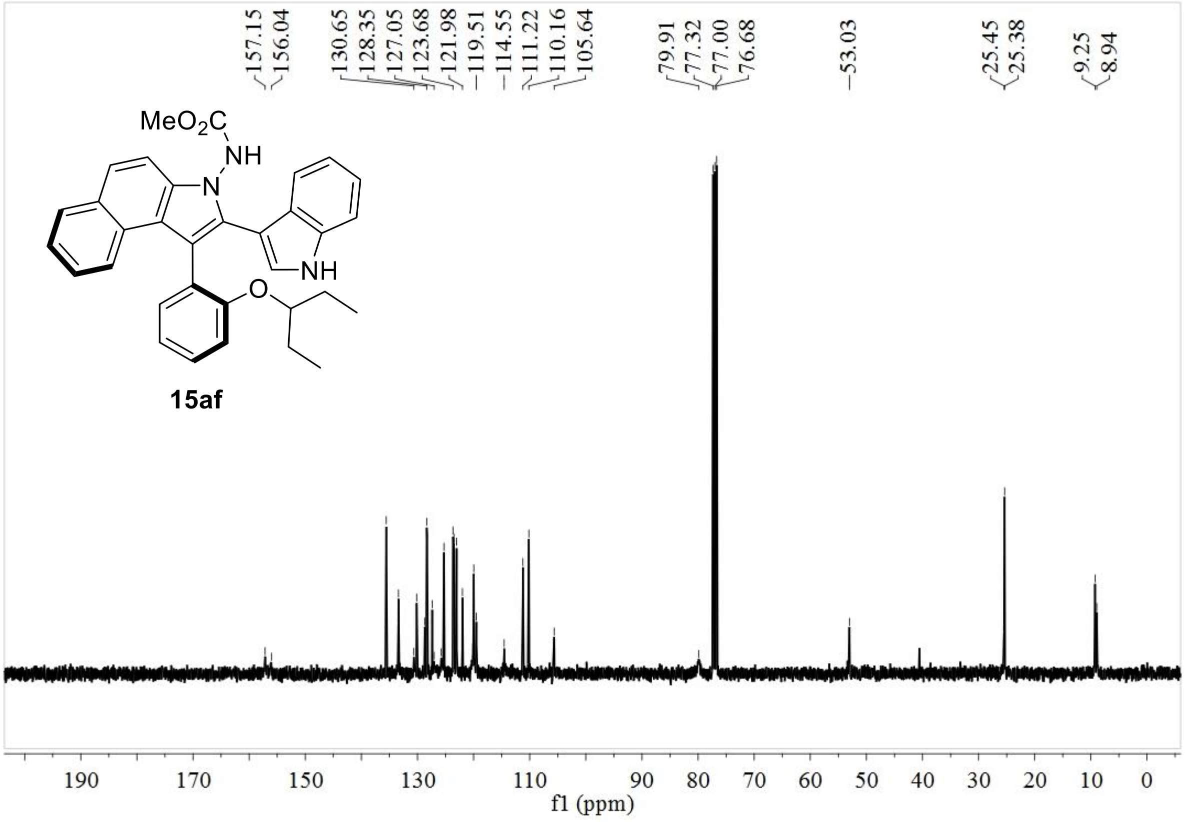
157.15
156.04
130.65
128.35
127.05
123.68
121.98
119.51
114.55
111.22
110.16
105.64
79.91
77.32
77.00
76.68
53.03
25.45
25.38
9.25
8.94
MeO2C
NH
N
NH
O
15af
f1 (ppm)

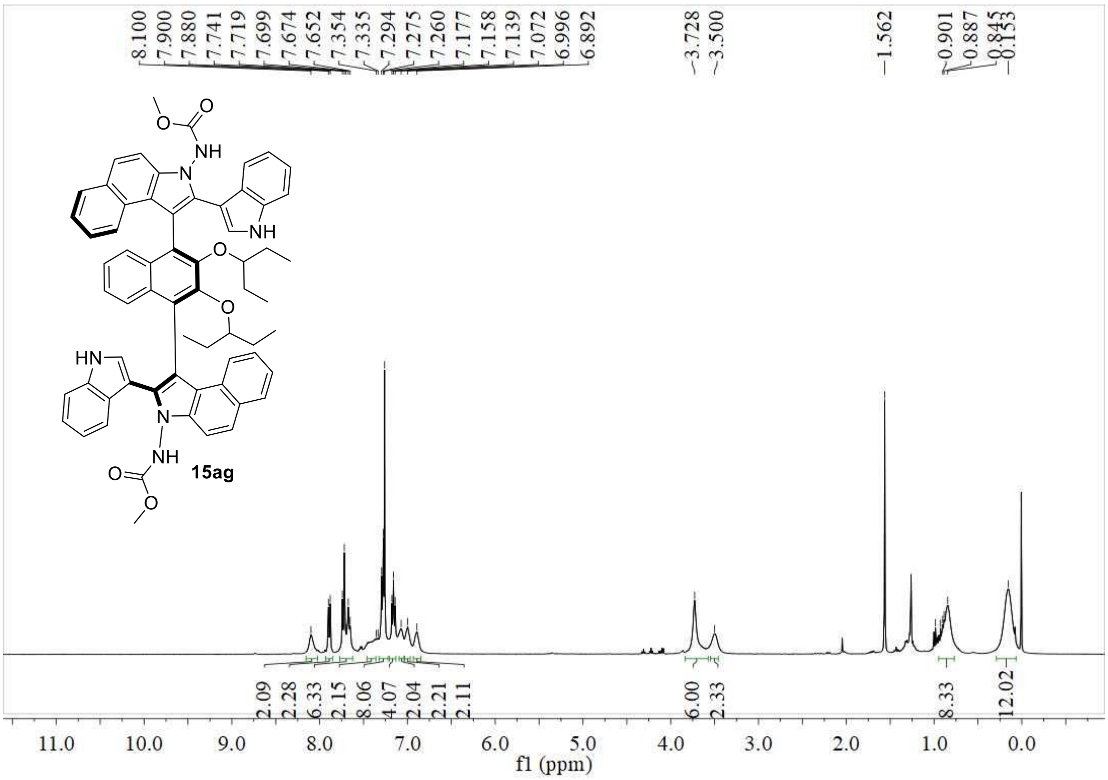

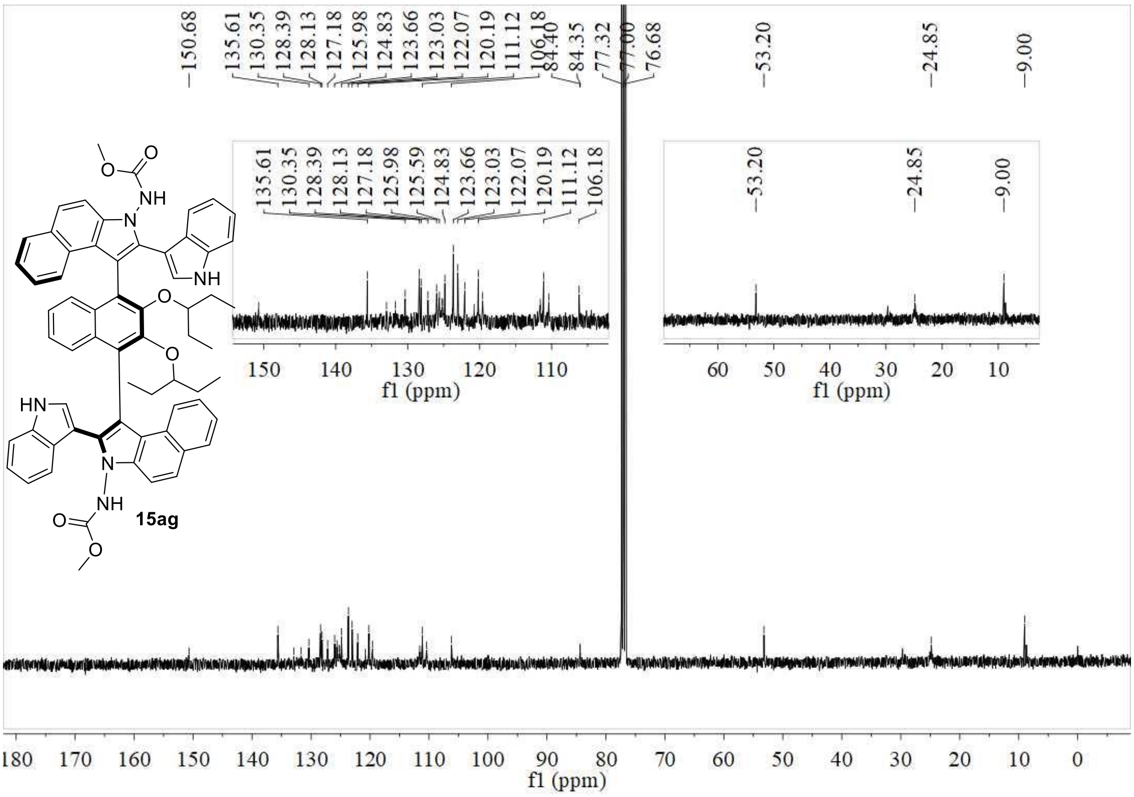

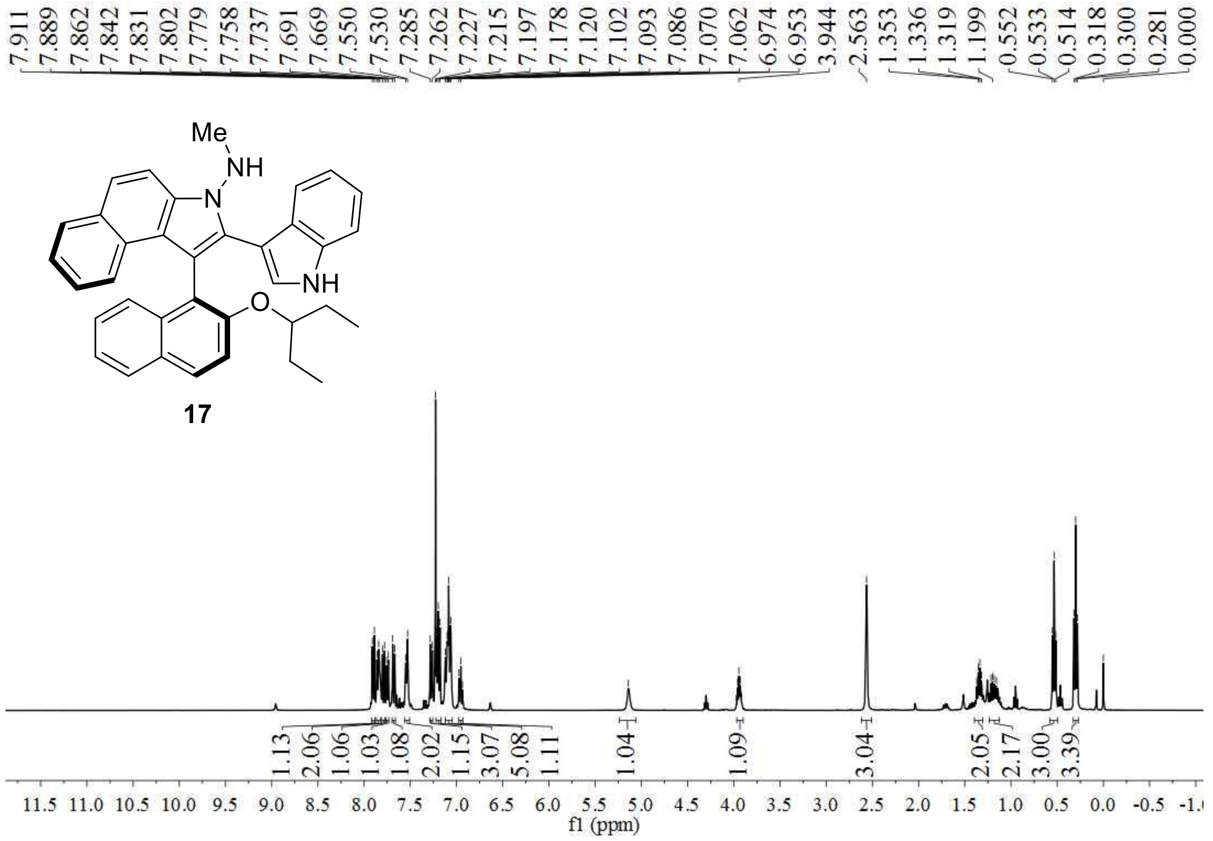
7.911 7.889 7.862 7.842 7.831 7.802 7.779 7.758 7.737 7.691 7.669 7.550 7.530 7.285 7.262 7.227 7.215 7.197 7.178 7.120 7.102 7.093 7.086 7.070 7.062 6.974 6.953 3.944 2.563 1.353 1.336 1.319 1.199 0.552 0.533 0.514 0.318 0.300 0.281 0.000
Me
NH
N
NH
O
17
1.13 2.06 1.06 1.03 1.08 2.02 1.15 3.07 5.08 1.11 1.04 1.09 3.04 2.05 2.17 3.00 3.39
f1 (ppm)

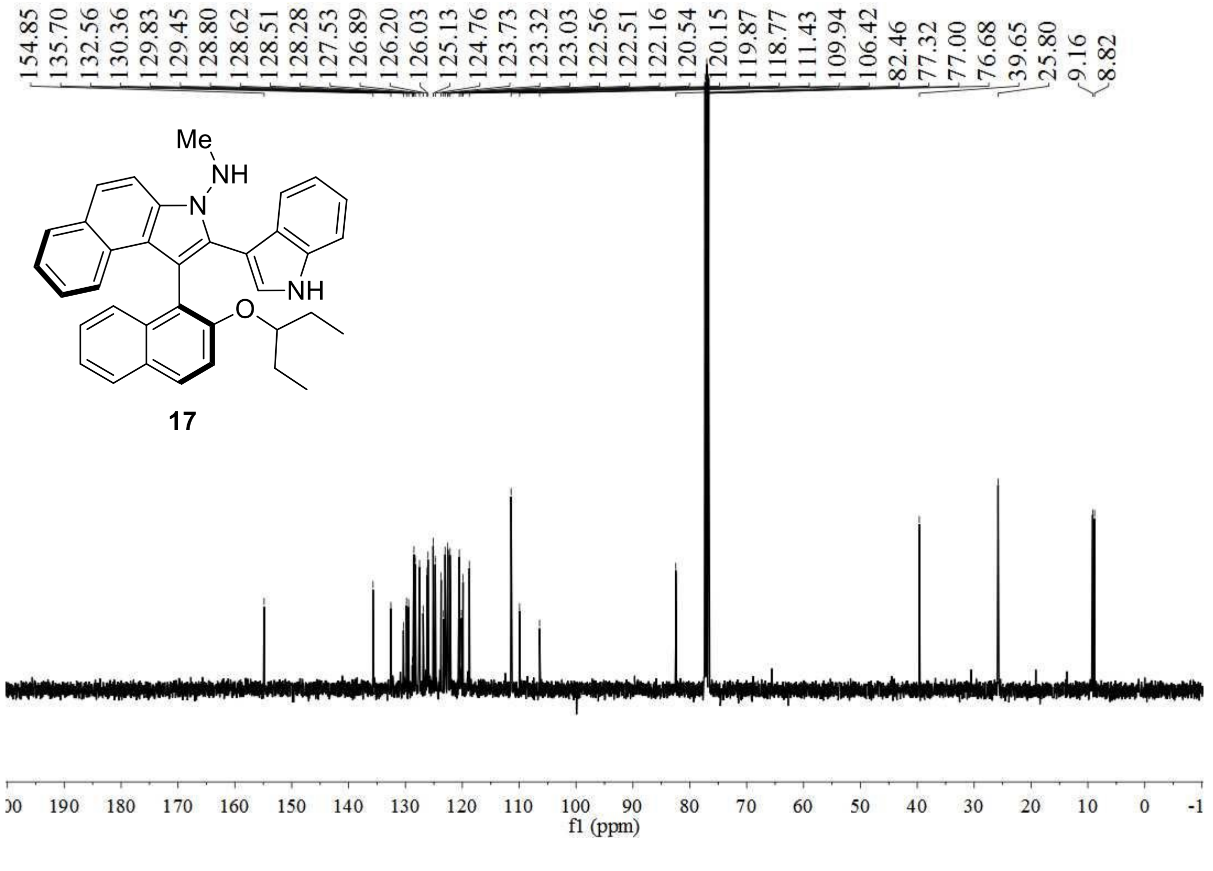
154.85 135.70 132.56 130.36 129.83 129.45 128.80 128.62 128.51 128.28 127.53 126.89 126.20 126.03 125.13 124.76 123.73 123.32 123.03 122.56 122.51 122.16 120.54 120.15 119.87 118.77 111.43 109.94 106.42 82.46 77.32 77.00 76.68 39.65 25.80 9.16 8.82
Me
NH
N
NH
O
17
f1 (ppm)

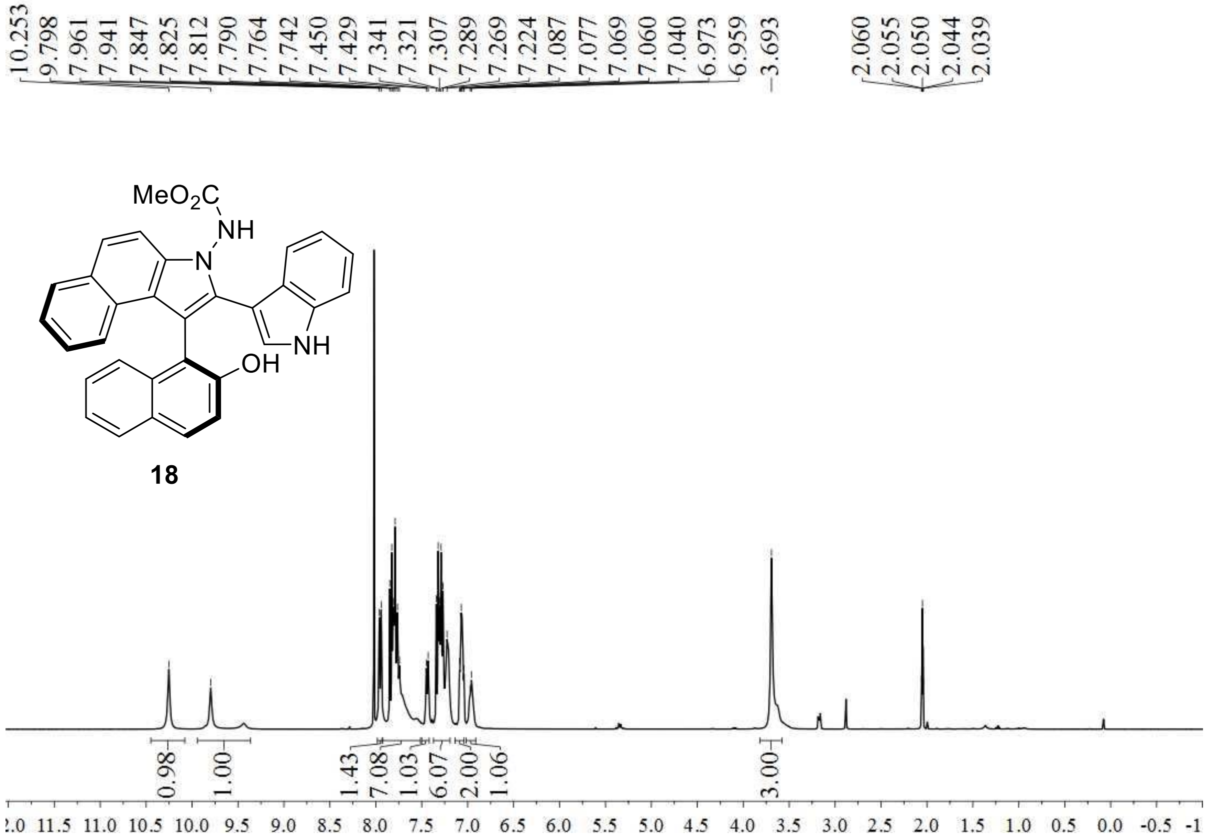
10.253
9.798
7.961
7.941
7.847
7.825
7.812
7.790
7.764
7.742
7.450
7.429
7.341
7.321
7.307
7.289
7.269
7.224
7.087
7.077
7.069
7.060
7.040
6.973
6.959
3.693
2.060
2.055
2.050
2.044
2.039
MeO2C
NH
N
NH
OH
18
0.98
1.00
1.43
7.08
1.03
6.07
2.00
1.06
3.00

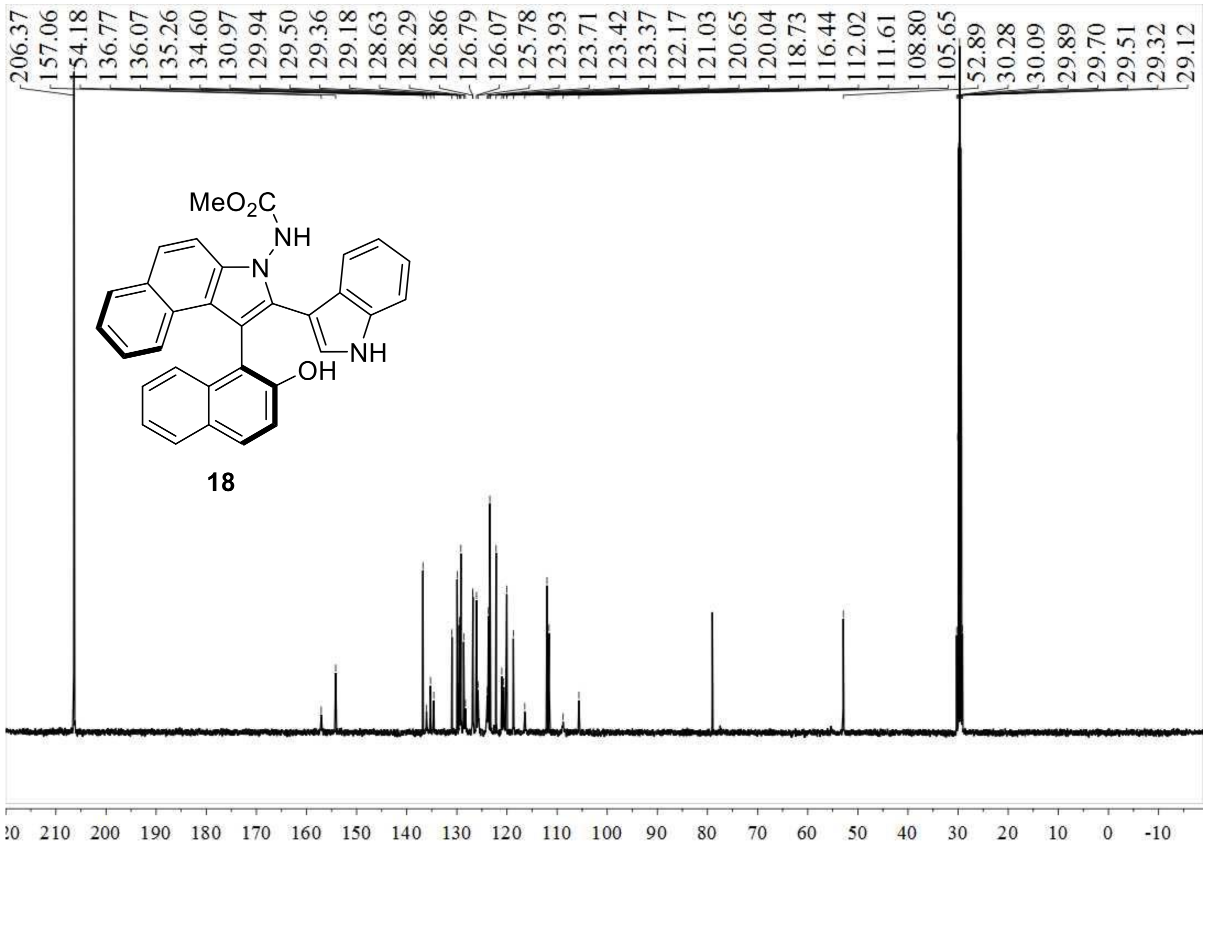
206.37
157.06
154.18
136.77
136.07
135.26
134.60
130.97
129.94
129.50
129.36
129.18
128.63
128.29
126.86
126.79
126.07
125.78
123.93
123.71
123.42
123.37
122.17
121.03
120.65
120.04
118.73
116.44
112.02
111.61
108.80
105.65
52.89
30.28
30.09
29.89
29.70
29.51
29.32
29.12
MeO2C
NH
N
NH
OH
18

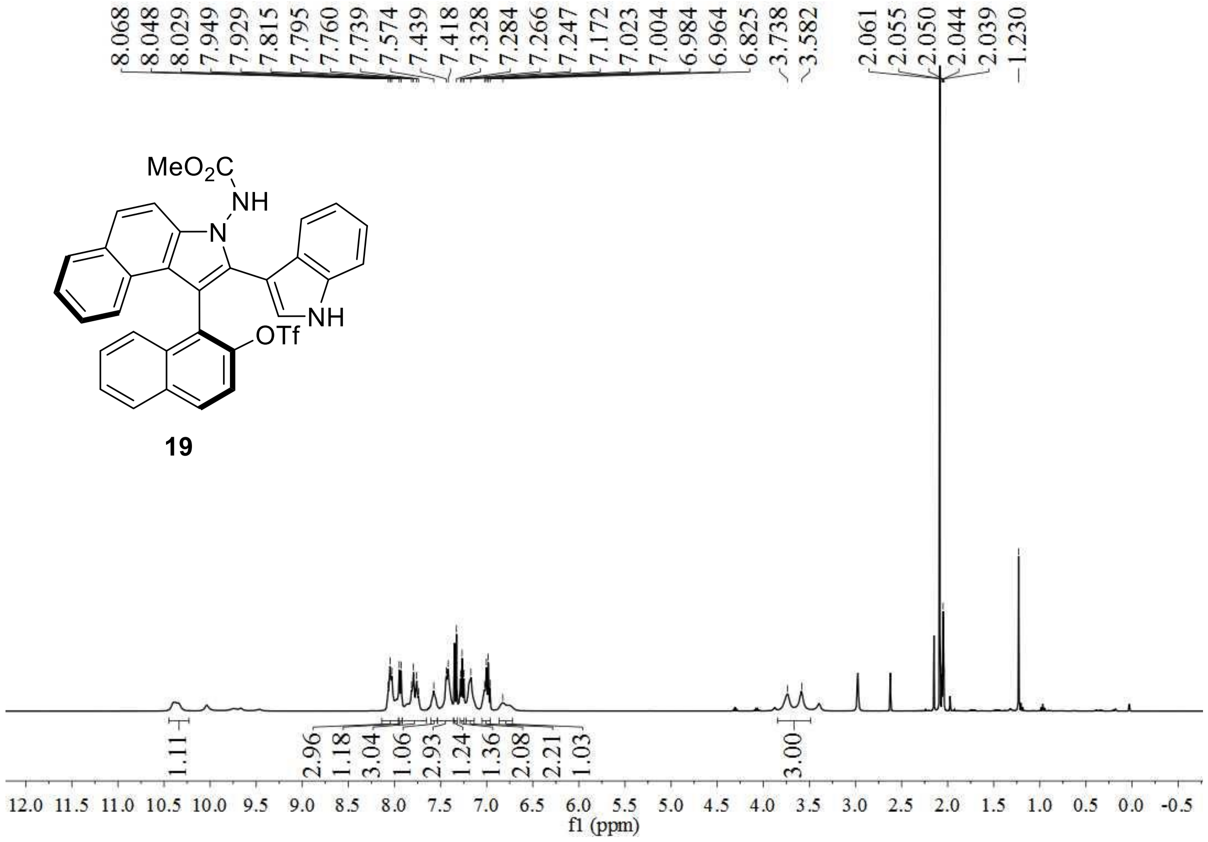
8.068
8.048
8.029
7.949
7.929
7.815
7.795
7.760
7.739
7.574
7.439
7.418
7.328
7.284
7.266
7.247
7.172
7.023
7.004
6.984
6.964
6.825
3.738
3.582
2.061
2.055
2.050
2.044
2.039
1.230
MeO2C
NH
N
OTf
NH
19
1.11
2.96
1.18
3.04
1.06
2.93
1.24
1.36
2.08
2.21
1.03
3.00
12.0 11.5 11.0 10.5 10.0 9.5 9.0 8.5 8.0 7.5 7.0 6.5 6.0 5.5 5.0 4.5 4.0 3.5 3.0 2.5 2.0 1.5 1.0 0.5 0.0 -0.5
f1 (ppm)

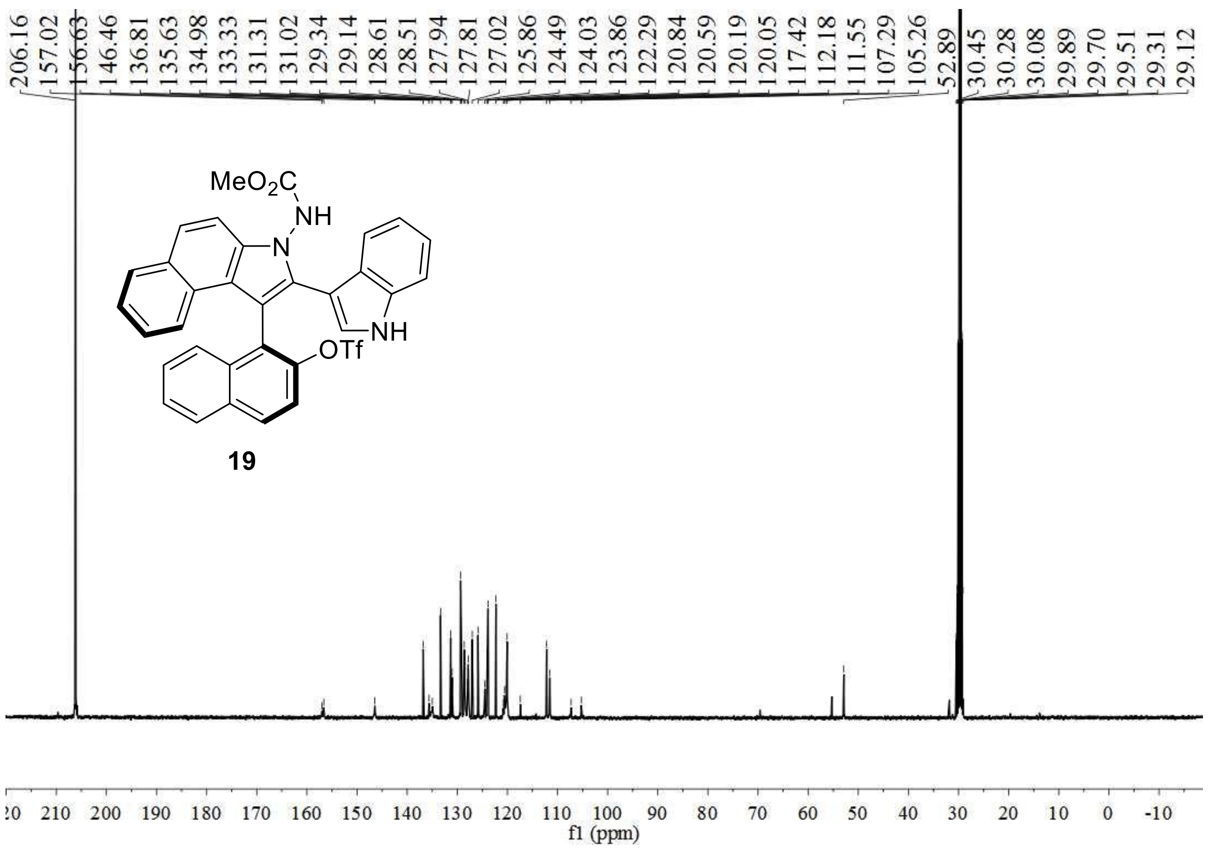
206.16
157.02
156.63
146.46
136.81
135.63
134.98
133.33
131.31
131.02
129.34
129.14
128.61
128.51
127.94
127.81
127.02
125.86
124.49
124.03
123.86
122.29
120.84
120.59
120.19
120.05
117.42
112.18
111.55
107.29
105.26
52.89
30.45
30.28
30.08
29.89
29.70
29.51
29.31
29.12
MeO2C
NH
N
OTf
NH
19
20 210 200 190 180 170 160 150 140 130 120 110 100 90 80 70 60 50 40 30 20 10 0 -10
f1 (ppm)

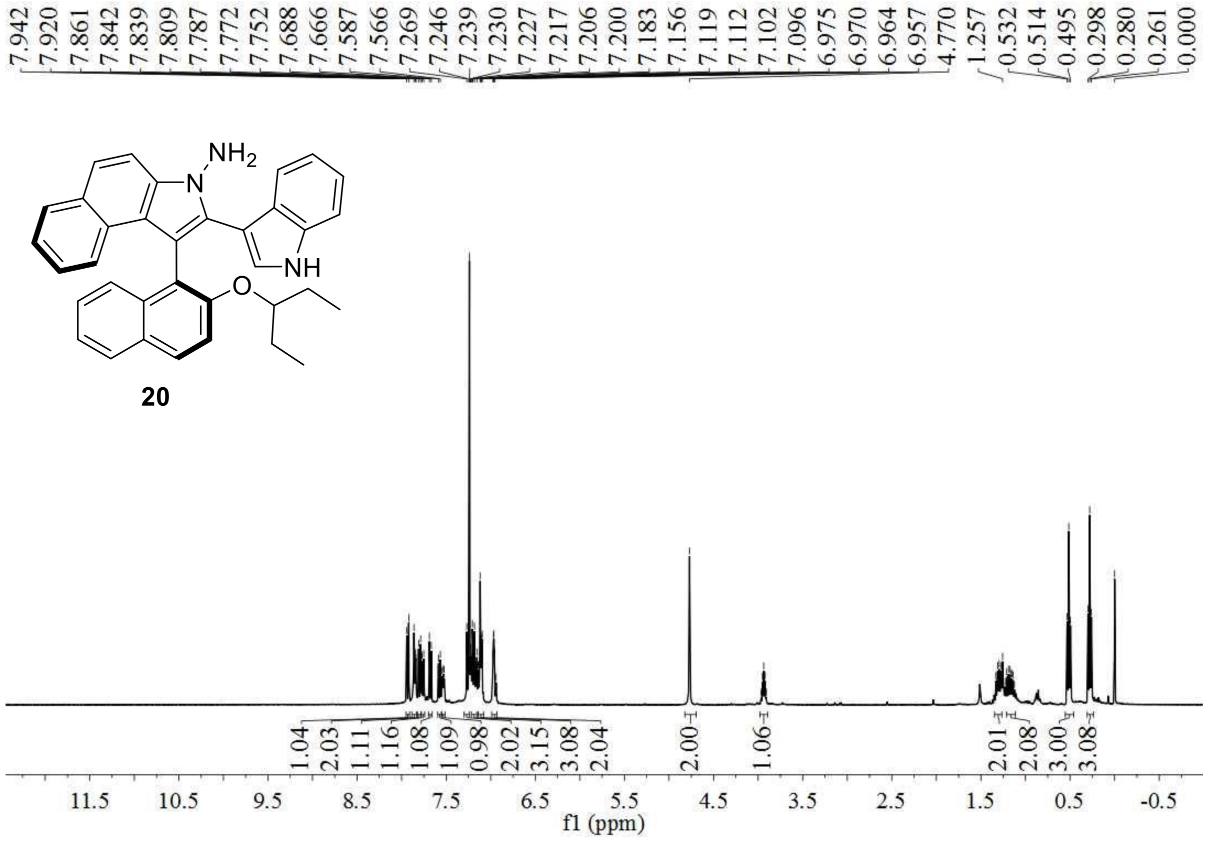
7.942 7.920 7.861 7.842 7.839 7.809 7.787 7.772 7.752 7.688 7.666 7.587 7.566 7.269 7.246 7.239 7.230 7.227 7.217 7.206 7.200 7.183 7.156 7.119 7.112 7.102 7.096 6.975 6.970 6.964 6.957 4.770 1.257 0.532 0.514 0.495 0.298 0.280 0.261 0.000
NH_2
N
NH
O
20
1.04 2.03 1.11 1.16 1.08 1.09 0.98 2.02 3.15 3.08 2.04 2.00 1.06 2.01 2.08 3.00 3.08
11.5 10.5 9.5 8.5 7.5 6.5 5.5 4.5 3.5 2.5 1.5 0.5 -0.5
f1 (ppm)

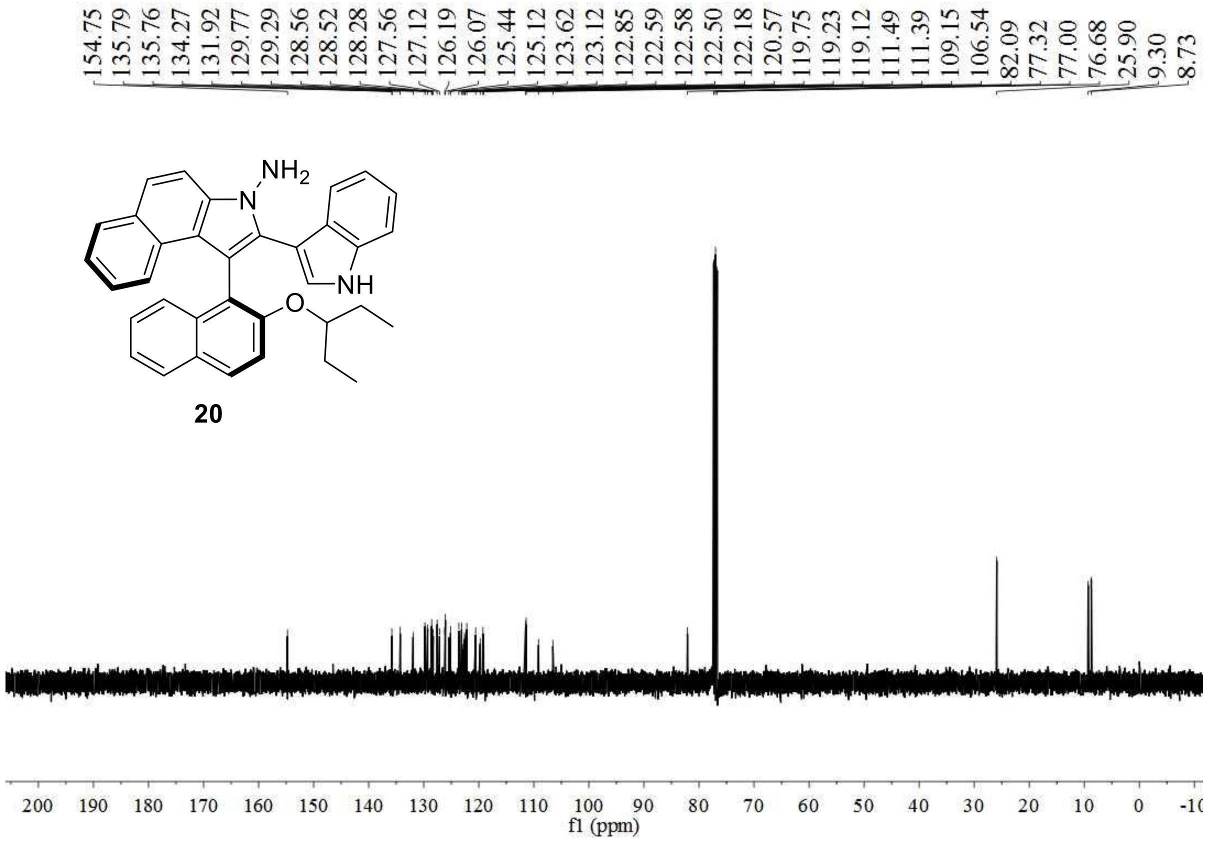
154.75 135.79 135.76 134.27 131.92 129.77 129.29 128.56 128.52 128.28 127.56 127.12 126.19 126.07 125.44 125.12 123.62 123.12 122.85 122.59 122.58 122.50 122.18 120.57 119.75 119.23 119.12 111.49 111.39 109.15 106.54 82.09 77.32 77.00 76.68 25.90 9.30 8.73
NH_2
N
NH
O
20
200 190 180 170 160 150 140 130 120 110 100 90 80 70 60 50 40 30 20 10 0
f1 (ppm)

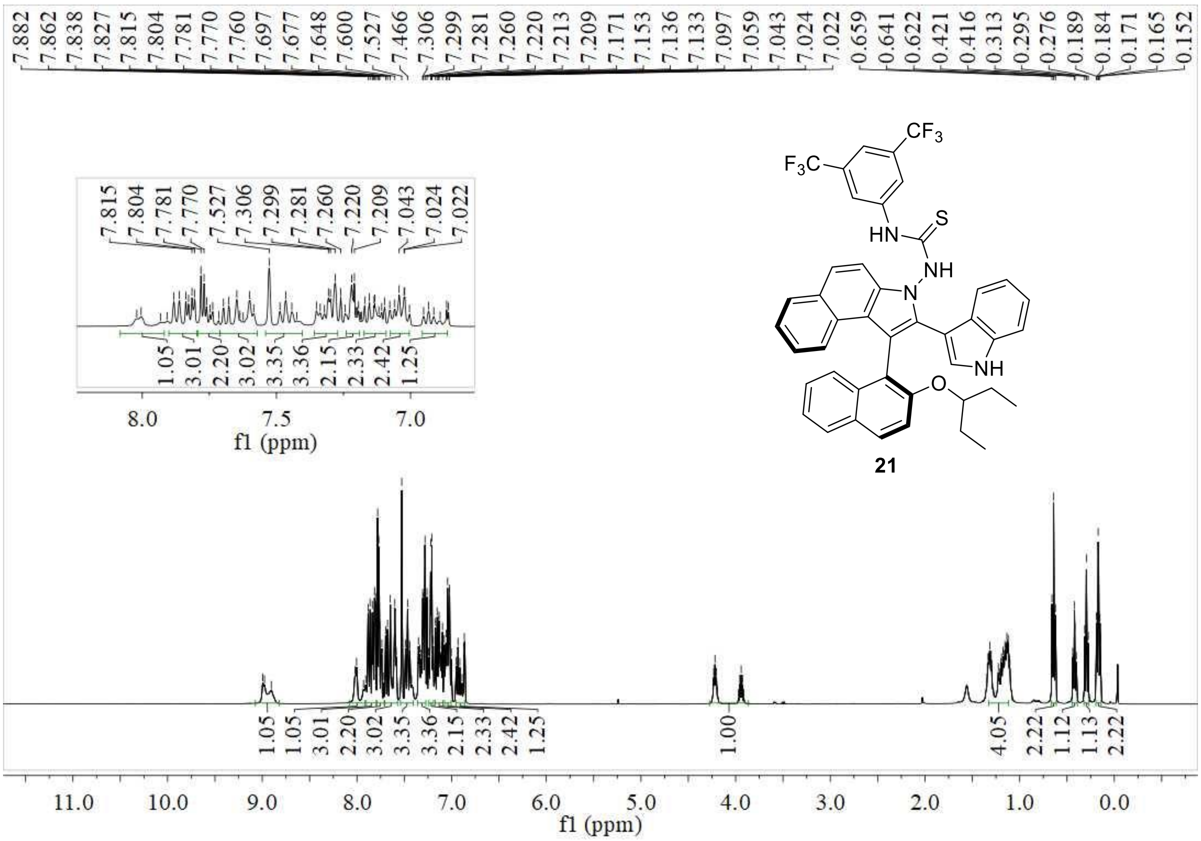

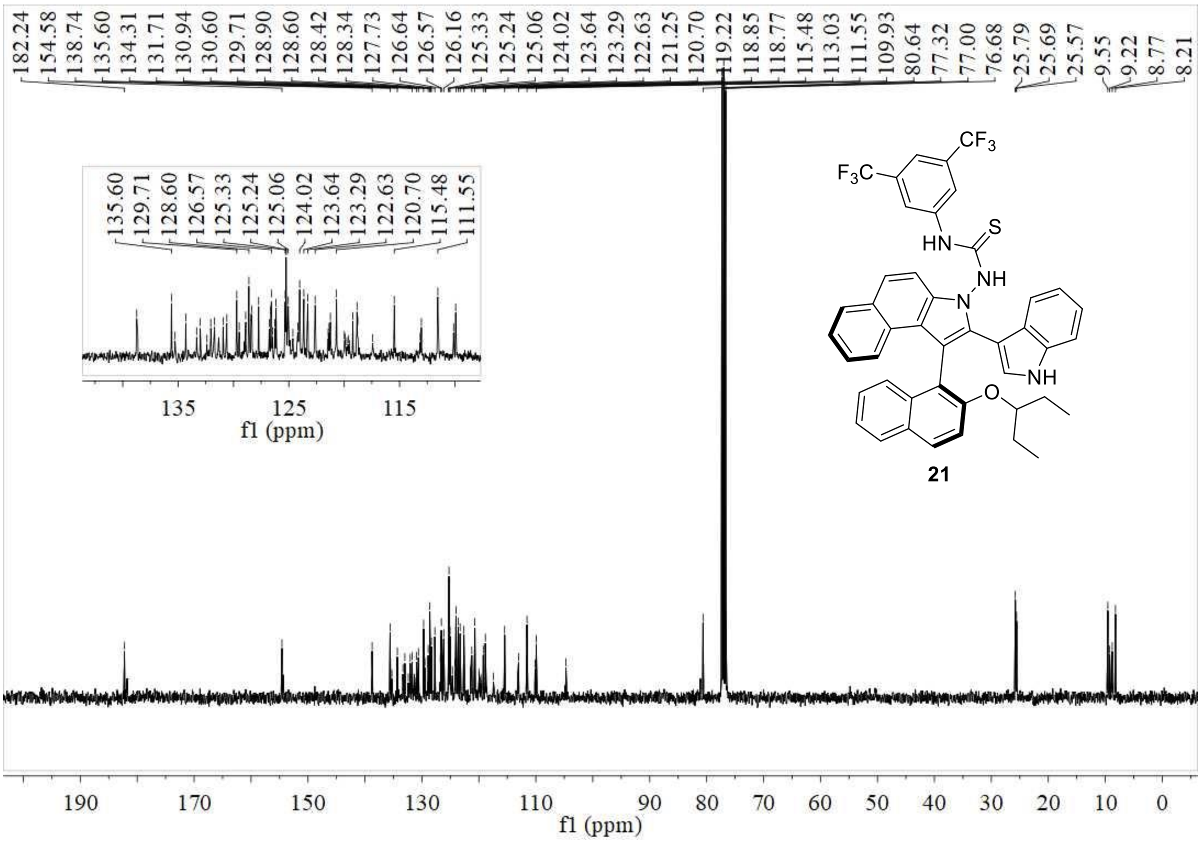

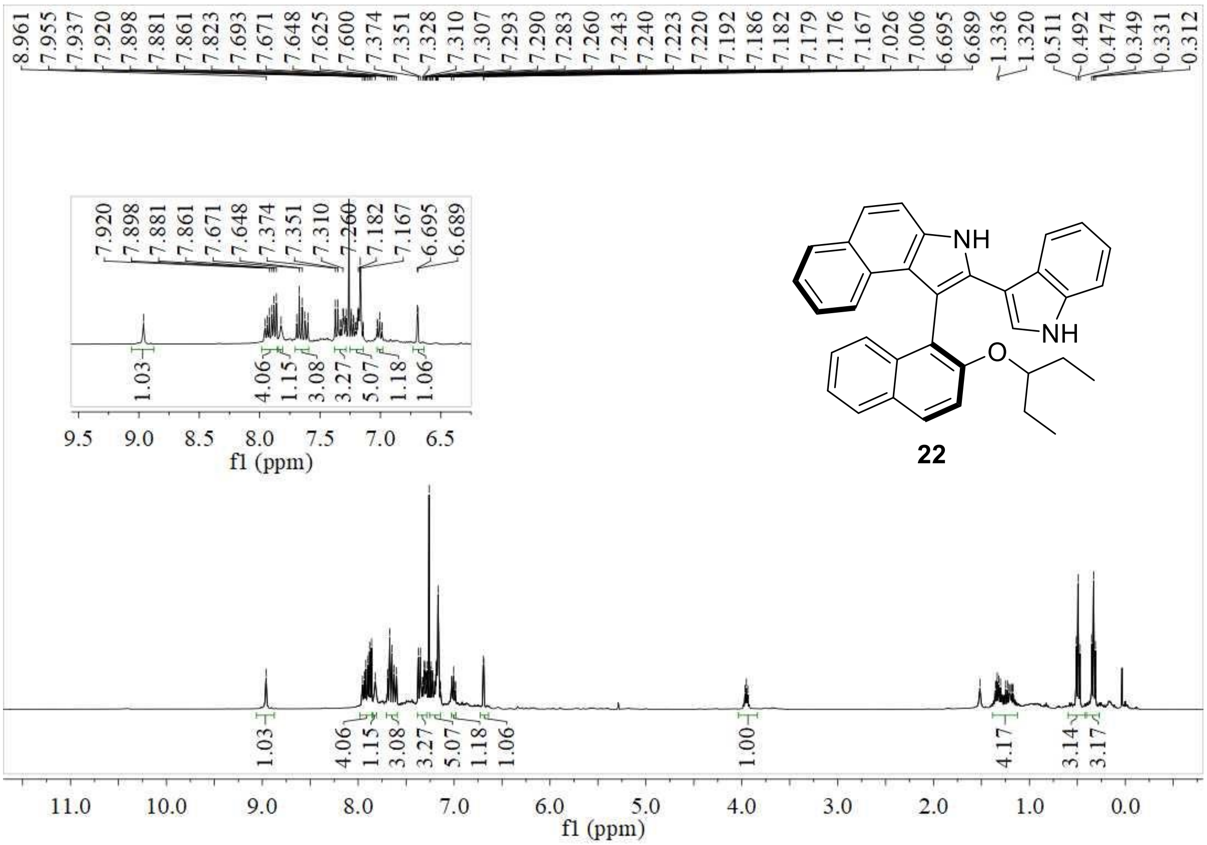
8.961 7.955 7.937 7.920 7.898 7.881 7.861 7.823 7.693 7.671 7.648 7.625 7.600 7.374 7.351 7.328 7.310 7.307 7.293 7.290 7.283 7.260 7.243 7.240 7.223 7.220 7.192 7.186 7.182 7.179 7.176 7.167 7.026 7.006 6.695 6.689 1.336 1.320 0.511 0.492 0.474 0.349 0.331 0.312
7.920 7.898 7.881 7.861 7.671 7.648 7.374 7.351 7.310 7.260 7.182 7.167 6.695 6.689
1.03 4.06 1.15 3.08 3.27 5.07 1.18 1.06
9.5 9.0 8.5 8.0 7.5 7.0 6.5
f1 (ppm)
NH
NH
O
22
1.03 4.06 1.15 3.08 3.27 5.07 1.18 1.06 1.00 4.17 3.14 3.17
11.0 10.0 9.0 8.0 7.0 6.0 5.0 4.0 3.0 2.0 1.0 0.0
f1 (ppm)

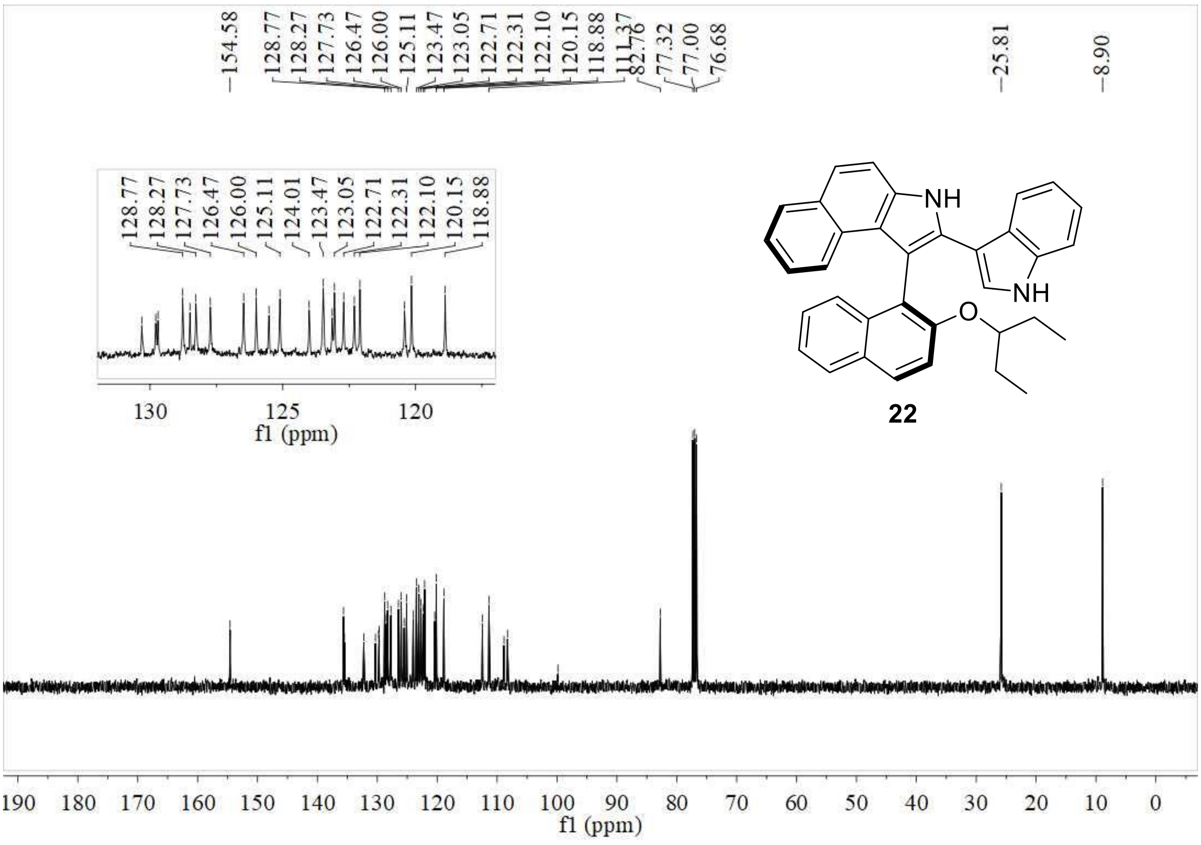
154.58 128.77 128.27 127.73 126.47 126.00 125.11 123.47 123.05 122.71 122.31 122.10 120.15 118.88 111.37 82.76 77.32 77.00 76.68 25.81 8.90
128.77 128.27 127.73 126.47 126.00 125.11 124.01 123.47 123.05 122.71 122.31 122.10 120.15 118.88
130 125 120
f1 (ppm)
NH
NH
O
22
190 180 170 160 150 140 130 120 110 100 90 80 70 60 50 40 30 20 10 0
f1 (ppm)

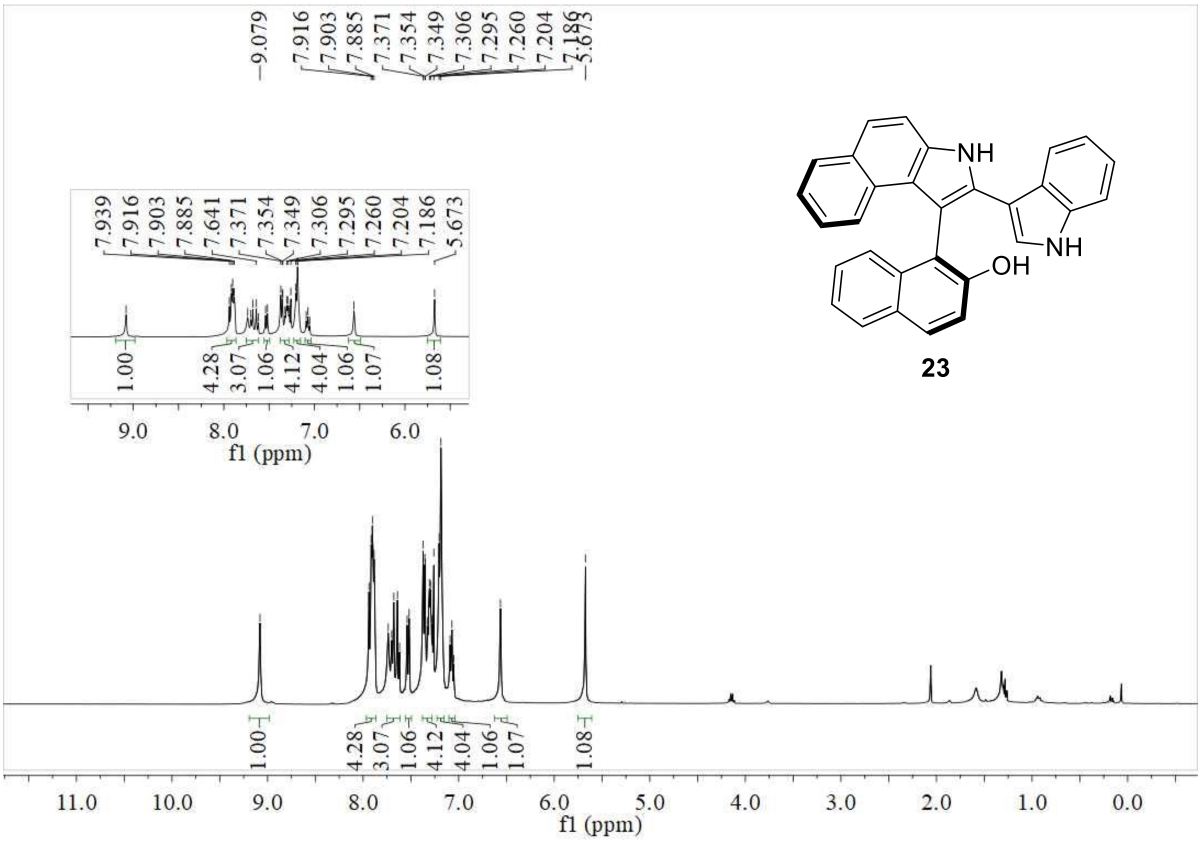
9.079
7.916
7.903
7.885
7.371
7.354
7.349
7.306
7.295
7.260
7.204
7.186
5.673
7.939
7.641
1.00
4.28
3.07
1.06
4.12
4.04
1.06
1.07
1.08
f1 (ppm)
NH
NH
OH
23

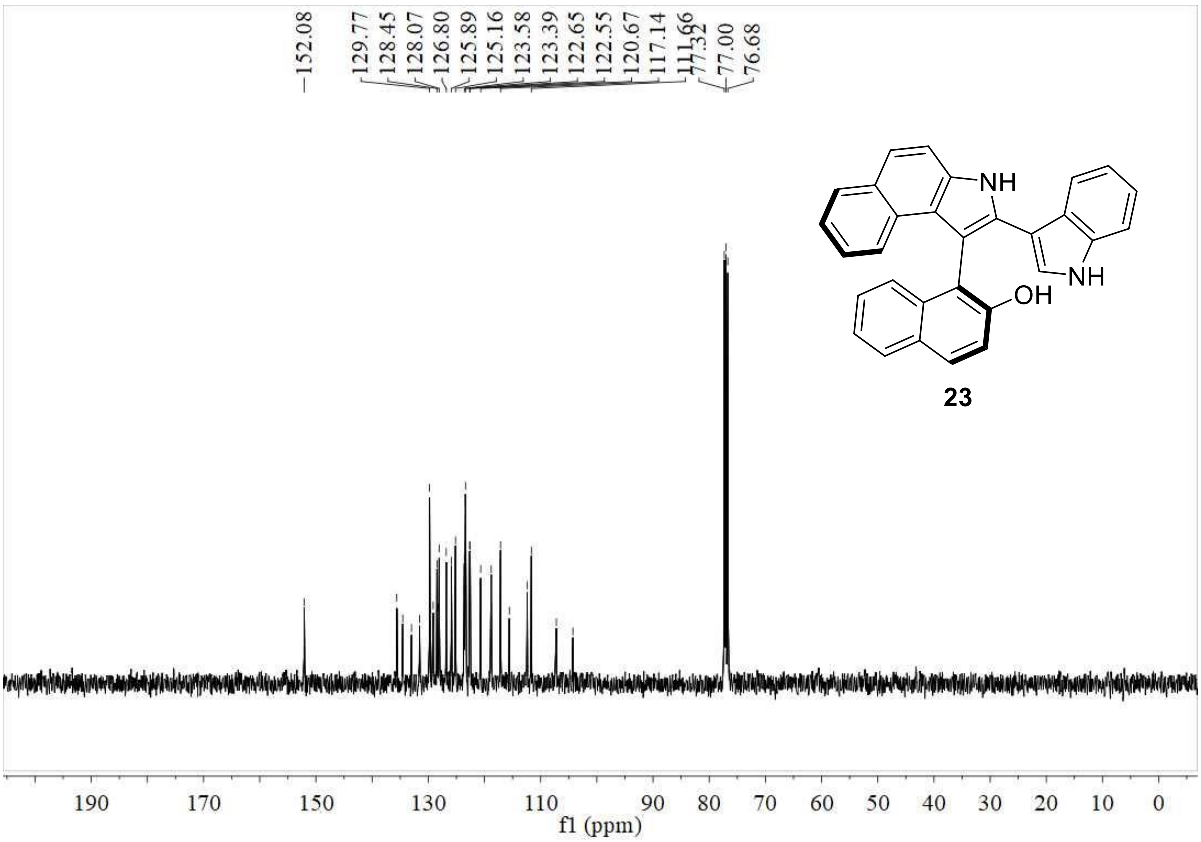
152.08
129.77
128.45
128.07
126.80
125.89
125.16
123.58
123.39
122.65
122.55
120.67
117.14
111.66
77.32
77.00
76.68
f1 (ppm)
NH
NH
OH
23

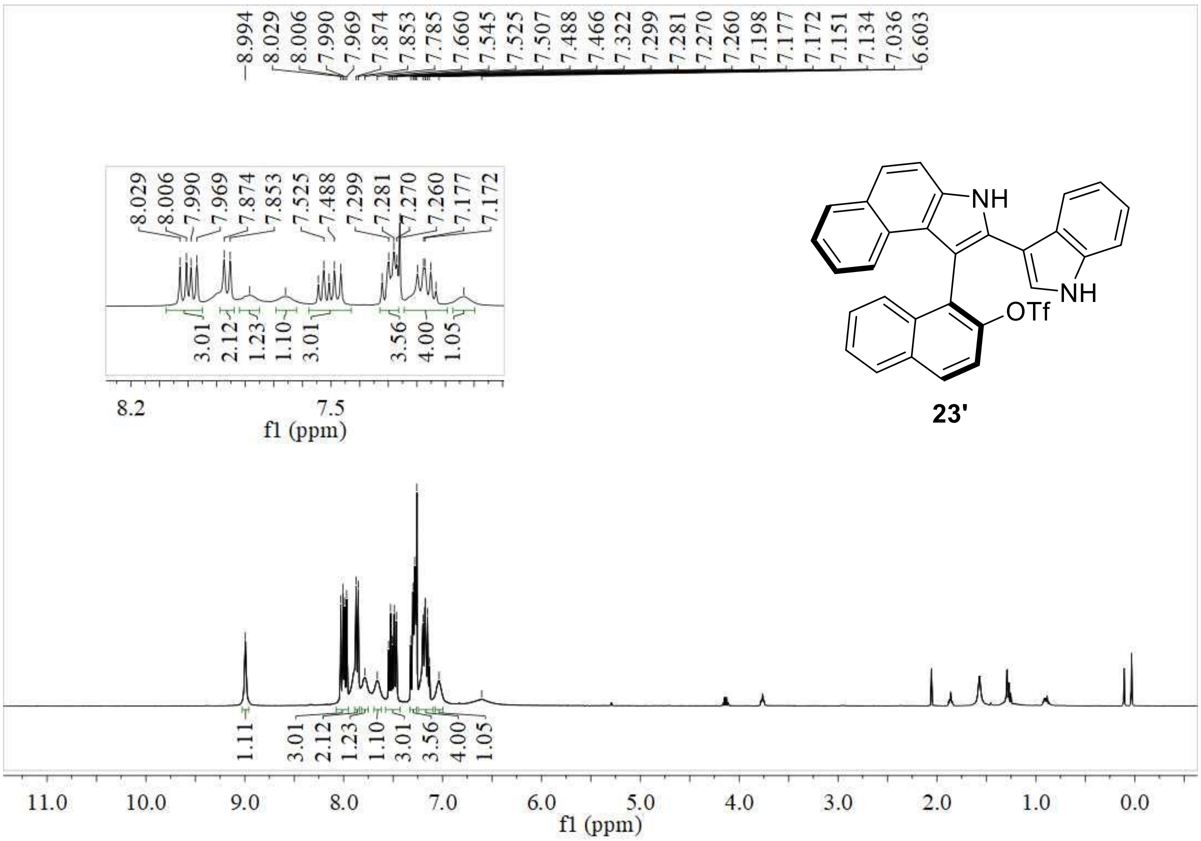
8.994
8.029
8.006
7.990
7.969
7.874
7.853
7.785
7.660
7.545
7.525
7.507
7.488
7.466
7.322
7.299
7.281
7.270
7.260
7.198
7.177
7.172
7.151
7.134
7.036
6.603
NH
NH
OTf
23'
1.11
3.01
2.12
1.23
1.10
3.01
3.56
4.00
1.05
f1 (ppm)

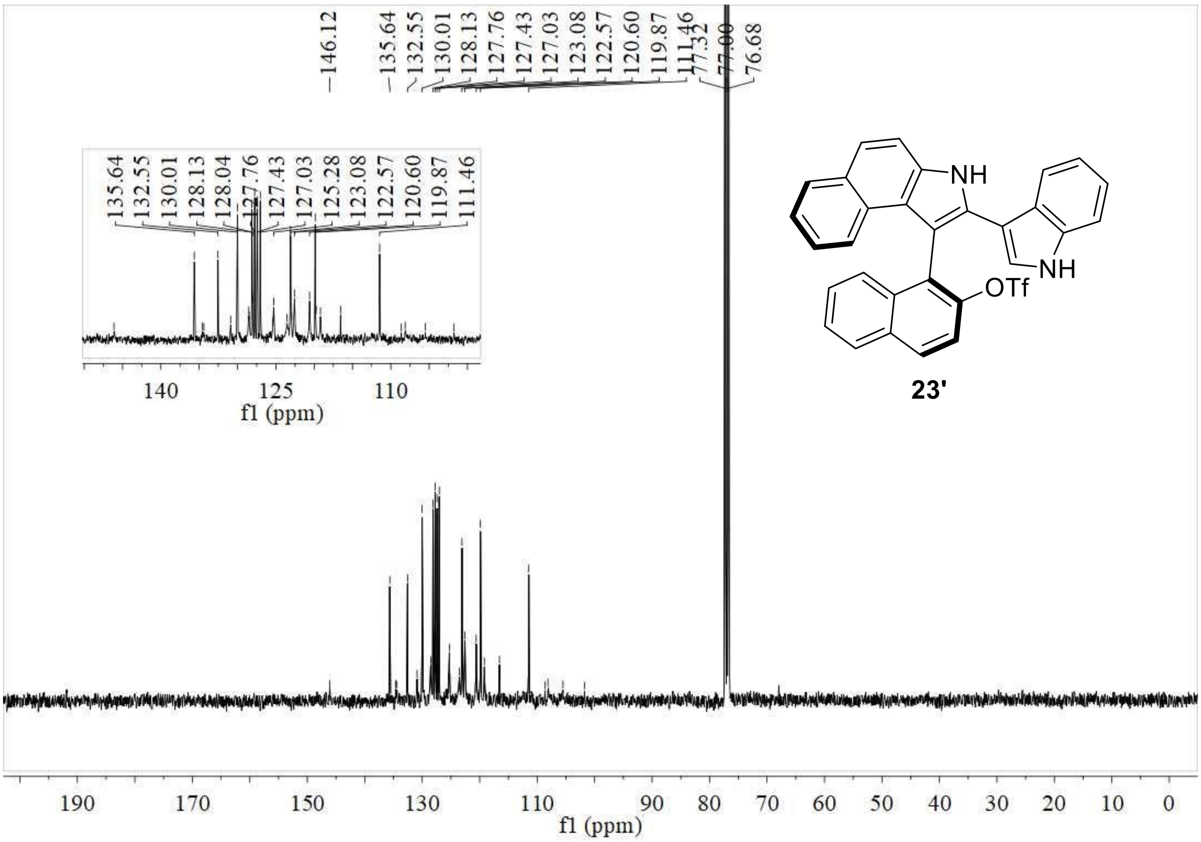
146.12
135.64
132.55
130.01
128.13
127.76
127.43
127.03
123.08
122.57
120.60
119.87
111.46
77.32
77.00
76.68
128.04
125.28
NH
NH
OTf
23'
f1 (ppm)

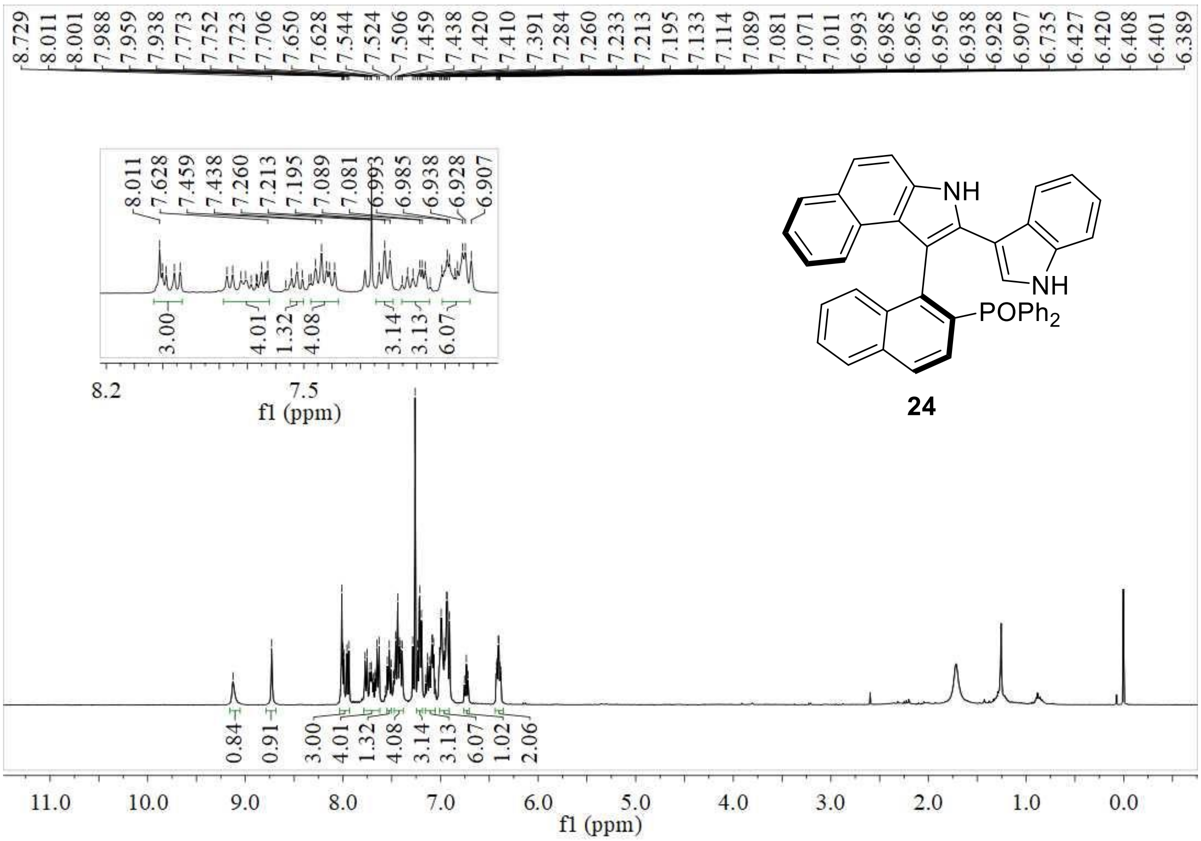
8.729 8.011 8.001 7.988 7.959 7.938 7.773 7.752 7.723 7.706 7.650 7.628 7.544 7.524 7.506 7.459 7.438 7.420 7.410 7.391 7.284 7.260 7.233 7.213 7.195 7.133 7.114 7.089 7.081 7.071 7.011 6.993 6.985 6.965 6.956 6.938 6.928 6.907 6.735 6.427 6.420 6.408 6.401 6.389
8.011 7.628 7.459 7.438 7.260 7.213 7.195 7.089 7.081 6.993 6.985 6.938 6.928 6.907
3.00 4.01 1.32 4.08 3.14 3.13 6.07
8.2
7.5
f1 (ppm)
NH
NH
POPh2
24
0.84 0.91 3.00 4.01 1.32 4.08 3.14 3.13 6.07 1.02 2.06
11.0 10.0 9.0 8.0 7.0 6.0 5.0 4.0 3.0 2.0 1.0 0.0
f1 (ppm)

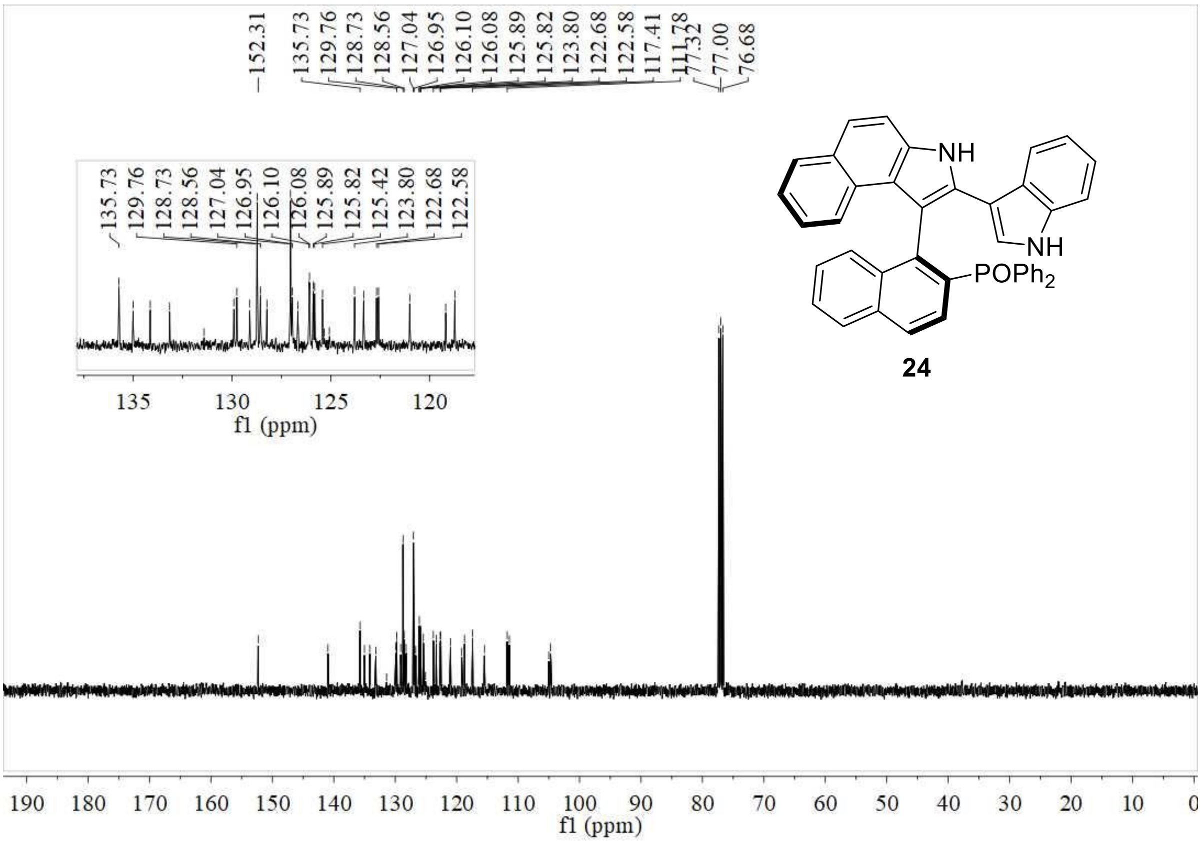
152.31 135.73 129.76 128.73 128.56 127.04 126.95 126.10 126.08 125.89 125.82 123.80 122.68 122.58 117.41 111.78 77.32 77.00 76.68
135.73 129.76 128.73 128.56 127.04 126.95 126.10 126.08 125.89 125.82 125.42 123.80 122.68 122.58
135 130 125 120
f1 (ppm)
NH
NH
POPh2
24
190 180 170 160 150 140 130 120 110 100 90 80 70 60 50 40 30 20 10 0
f1 (ppm)

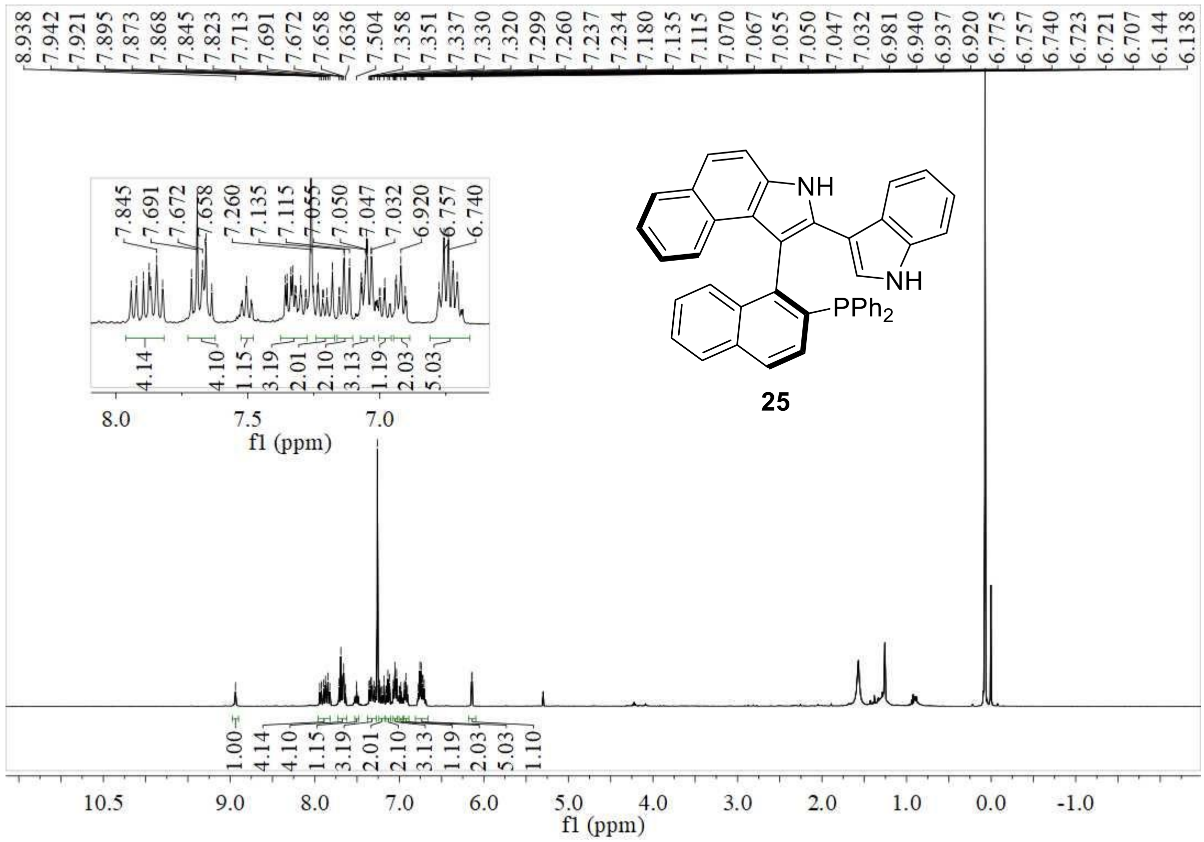
8.938 7.942 7.921 7.895 7.873 7.868 7.845 7.823 7.713 7.691 7.672 7.658 7.636 7.504 7.358 7.351 7.337 7.330 7.320 7.299 7.260 7.237 7.234 7.180 7.135 7.115 7.070 7.067 7.055 7.050 7.047 7.032 6.981 6.940 6.937 6.920 6.775 6.757 6.740 6.723 6.721 6.707 6.144 6.138
NH
NH
PPh2
25
1.00 4.14 4.10 1.15 3.19 2.01 2.10 3.13 1.19 2.03 5.03 1.10
f1 (ppm)

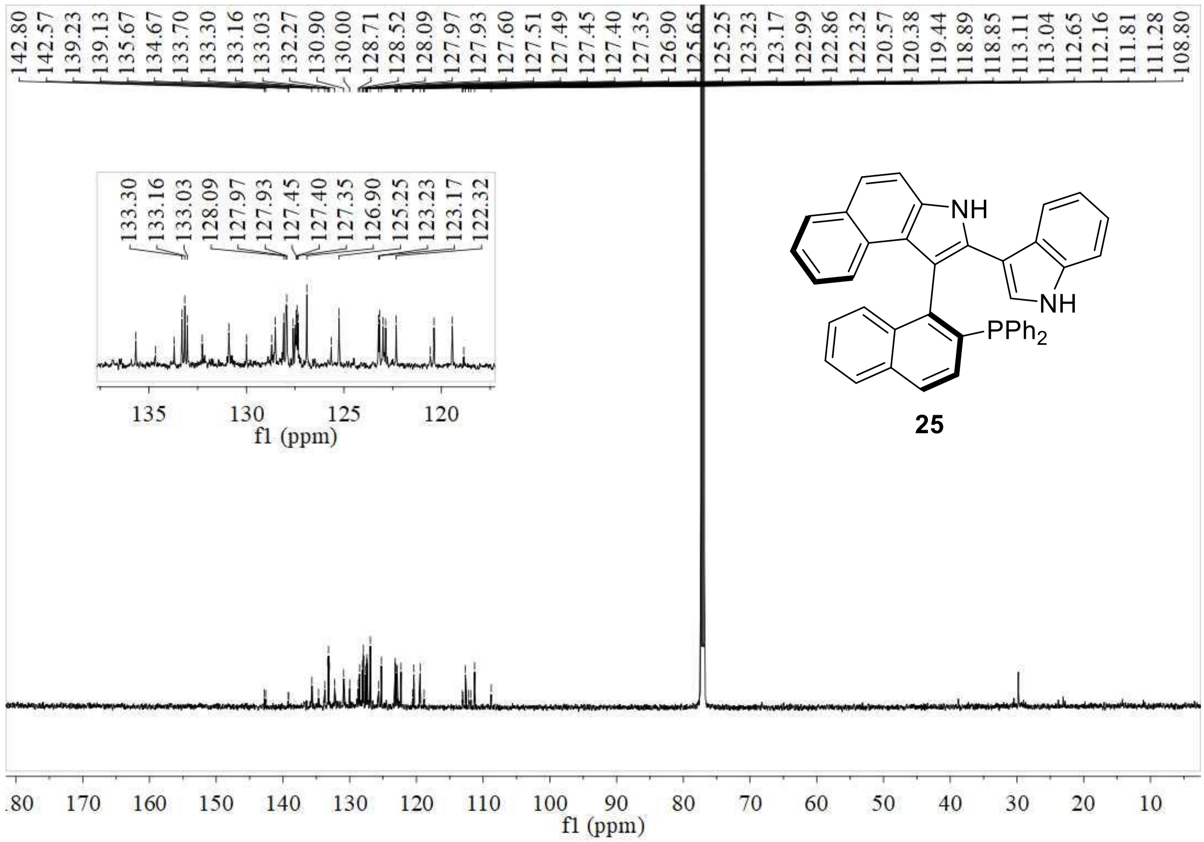
142.80 142.57 139.23 139.13 135.67 134.67 133.70 133.30 133.16 133.03 132.27 130.90 130.00 128.71 128.52 128.09 127.97 127.93 127.60 127.51 127.49 127.45 127.40 127.35 126.90 125.65 125.25 123.23 123.17 122.99 122.86 122.32 120.57 120.38 119.44 118.89 118.85 113.11 113.04 112.65 112.16 111.81 111.28 108.80
NH
NH
PPh2
25
f1 (ppm)

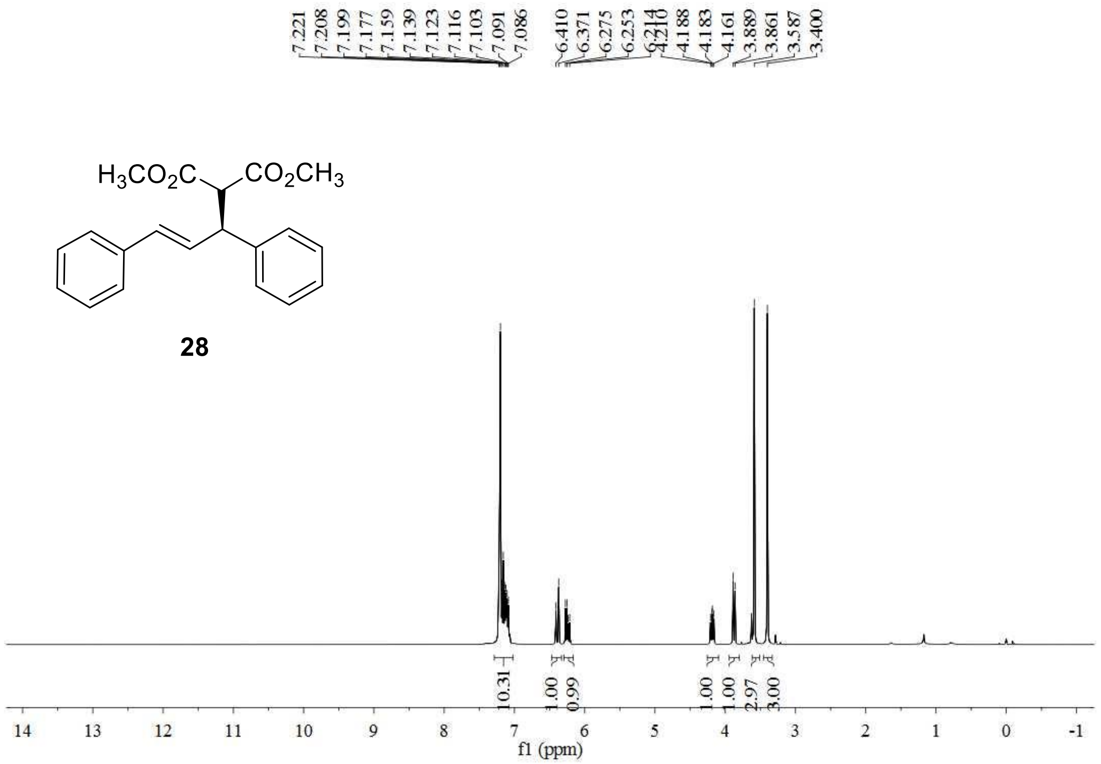
7.221 7.208 7.199 7.177 7.159 7.139 7.123 7.116 7.103 7.091 7.086
6.410 6.371 6.275 6.253 6.214 4.210 4.188 4.183 4.161 3.889 3.861 3.587 3.400
H_3CO_2C
CO_2CH_3
28
10.31 1.00 0.99 1.00 1.00 2.97 3.00
14 13 12 11 10 9 8 7 6 5 4 3 2 1 0 -1
f1 (ppm)

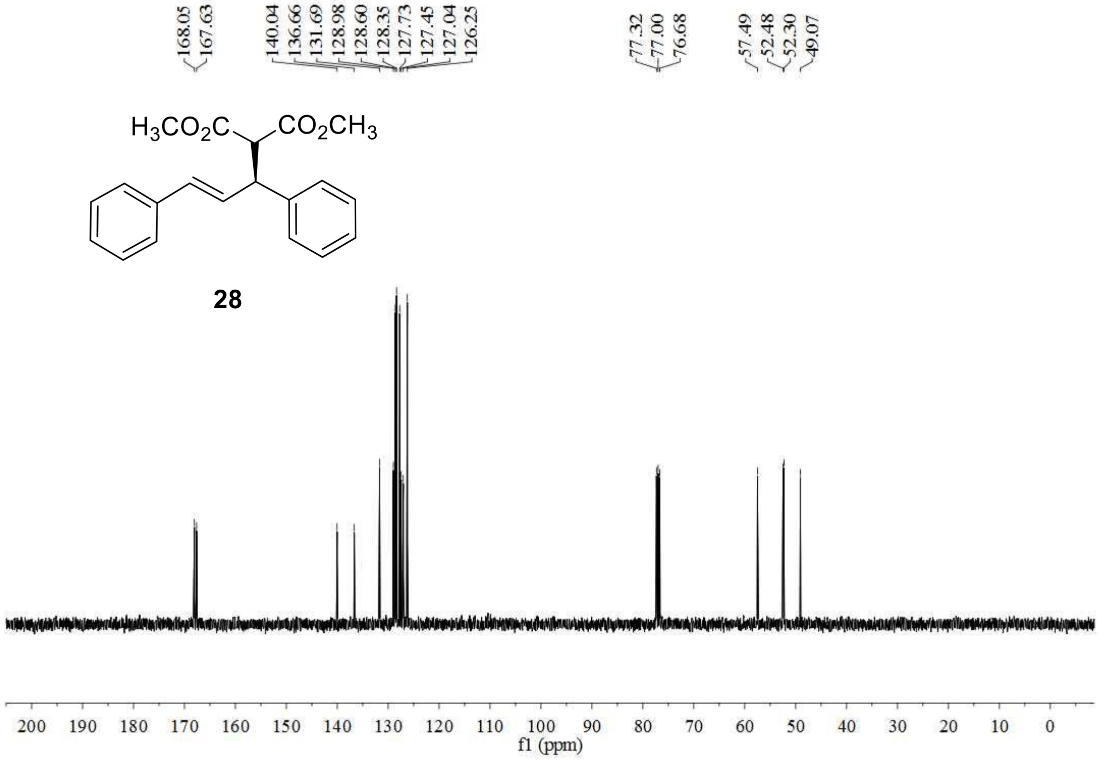
168.05 167.63
140.04 136.66 131.69 128.98 128.60 128.35 127.73 127.45 127.04 126.25
77.32 77.00 76.68
57.49 52.48 52.30 49.07
H_3CO_2C
CO_2CH_3
28
200 190 180 170 160 150 140 130 120 110 100 90 80 70 60 50 40 30 20 10 0
f1 (ppm)

CD spectra

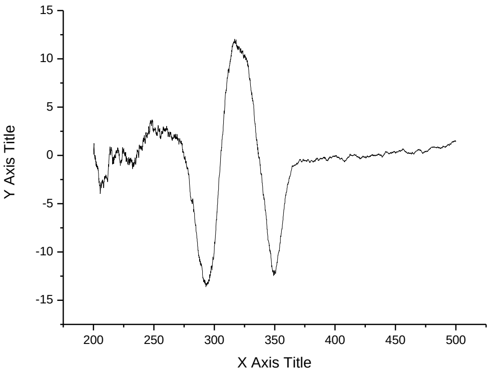

15a

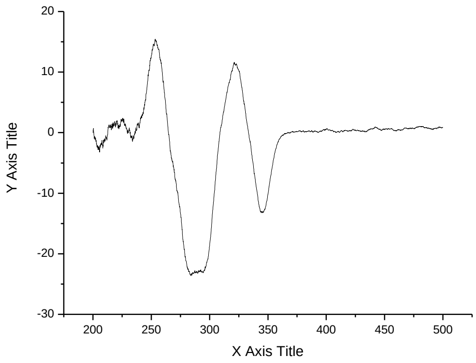

15d

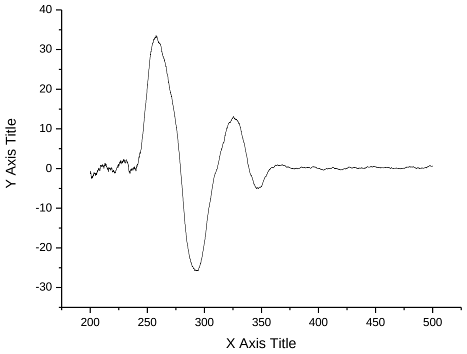

15f

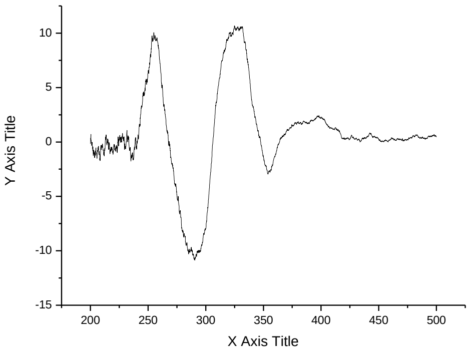

15r

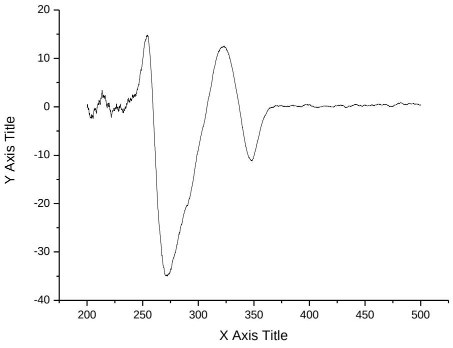

15u

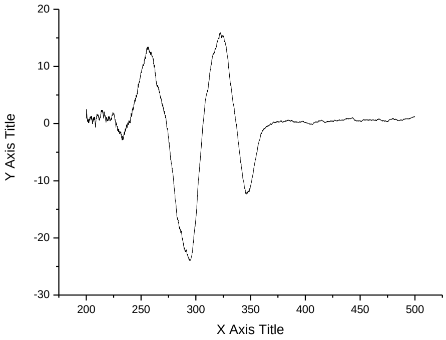

15w

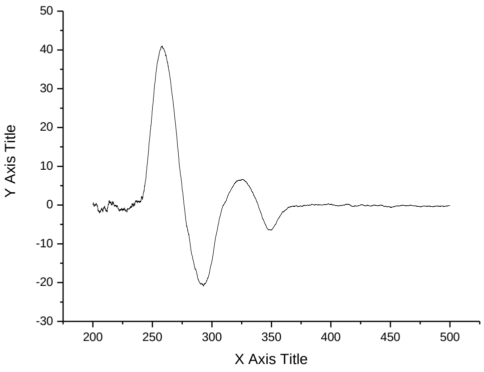

15x

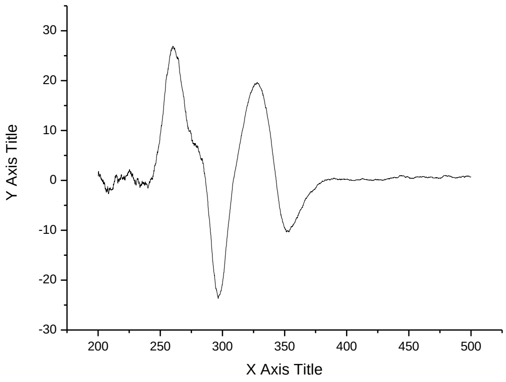

15y

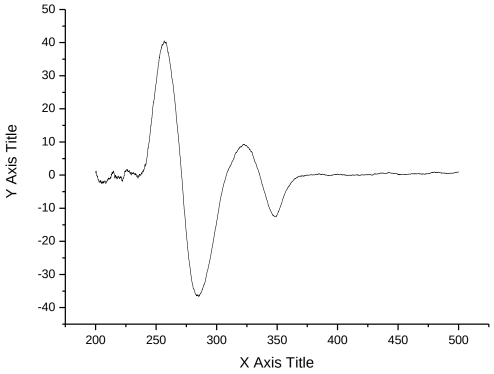

15z

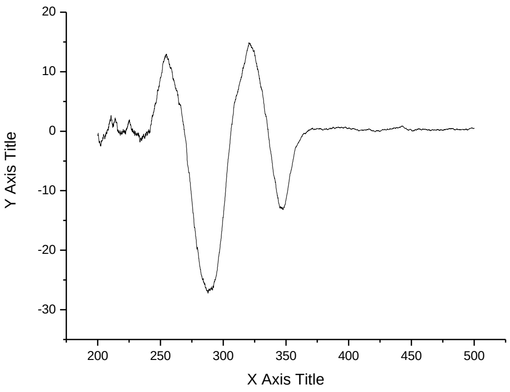

15ac

Fig. 1. X-ray structure of 15x

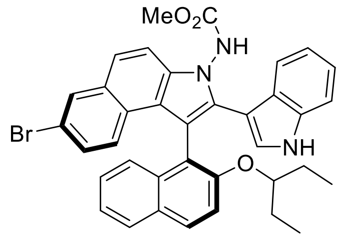

15x
CCDC 2005062

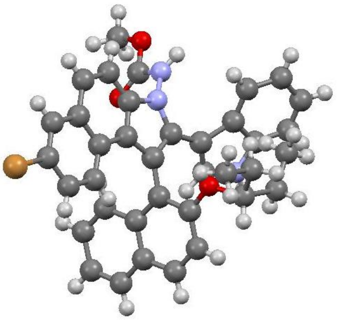

Bond precision:	C-C = 0.0131 A		Wavelength=0.71073
Cell:	a = 27.390(5)	b = 9.6268(17)	c = 15.237(3)
	alpha = 90	beta = 102.798(5)	gamma = 90
Temperature:	150 K		

Calculated	Reported	
Volume	3917.9 (13)	3917.8 (12)
Space group	C 2	C 1 2 1
Hall group	C 2y	C 2y
Moiety formula	C37 H32 Br N3 O3 [+solvent]	C37 H32 Br N3 O3
Sum formula	C37 H32 Br N3 O3 [+solvent]	C41 H40 Br N3 O5
Mr	646.56	734.67
Dx,g cm-3	1.096	1.246
Z	4	4
Mu (mm-1)	1.082	1.094
F000	1336.0	1528.0
F000'	1335.34	
h,k,lmax	32,11,18	32,11,18
Nref	7225 [3845]	7056
Tmin,Tmax	0.843,0.877	0.559,0.745
Tmin'	0.830	

Correction method= # Reported T Limits: Tmin=0.559 Tmax=0.745
AbsCorr = MULTI-SCAN

Data completeness= 1.84/0.98 Theta(max)= 25.403

R(reflections)= 0.0637(4846) wR2(reflections)= 0.1790(7056)

S = 1.009 Npar= 400

Computational Studies

Density functional theory (DFT) calculations were conducted using Gaussian 09 program.[11] All the intermediates and transition states were optimized by employing

the range separated functional ωB97X-D[12] and the 6-31G(d) basis set.[13-15] Normal vibrational mode analysis at the same level of theory confirmed that the optimized structures are minima (zero imaginary frequency) or saddle points (one imaginary frequency). Based on optimized geometries, free energies at 223K with solvent effects in chloroform were computed with the same functional and the 6-311G (d,p) basis set[16,17] using the SMD model.[18] DFT-optimized structures are illustrated using CYLView.[19] The free energy profiles (in Kcal/mol) of the most favorable pathway for the phosphoric acid catalyzed cycloaddition of alkynylindoles with azonaphthalenes are shown in Figure S2.

Fig. 2. DFT-calculations

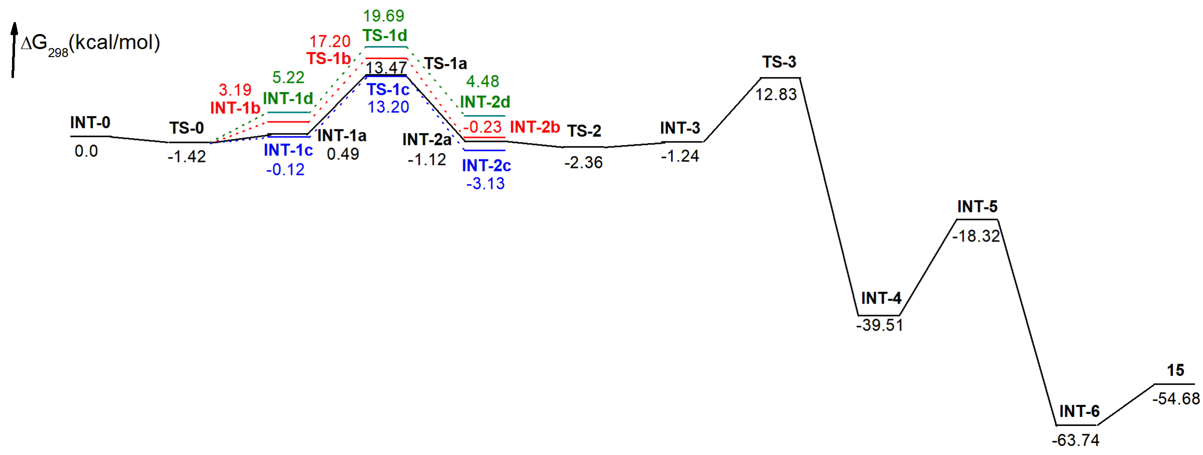

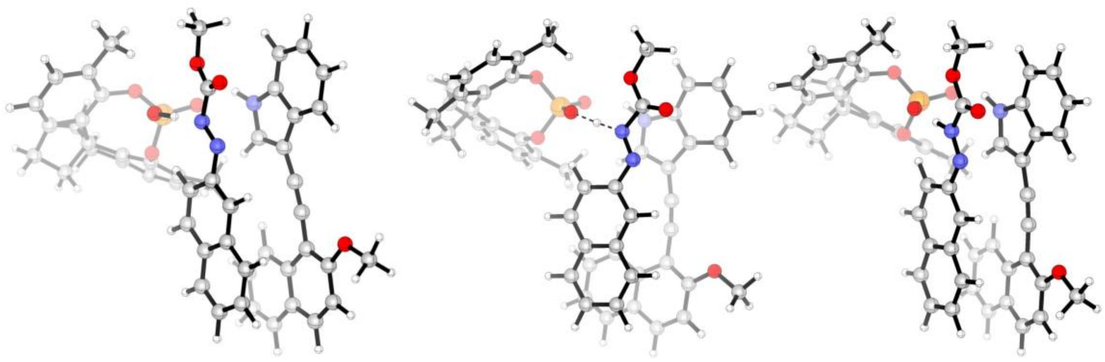

INT-0 **TS-0** **INT-1a**

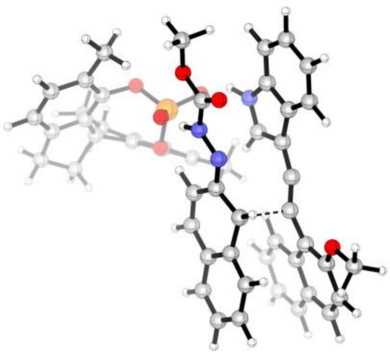

TS-1a

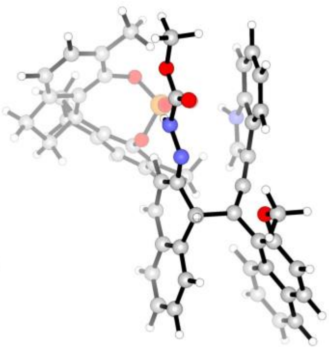

INT-2a

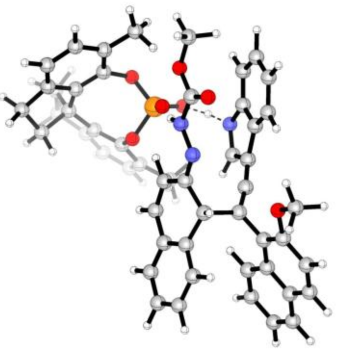

TS-2

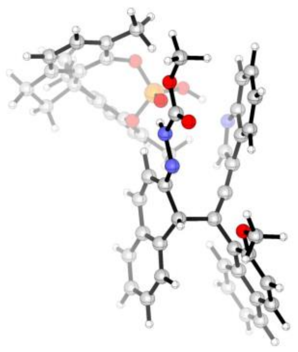

INT-3

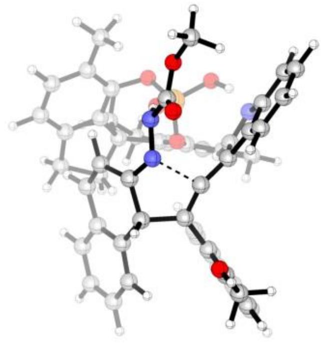

TS-3

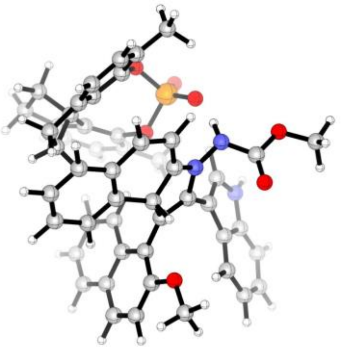

INT-4

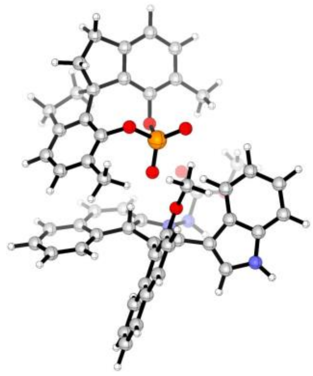

INT-5

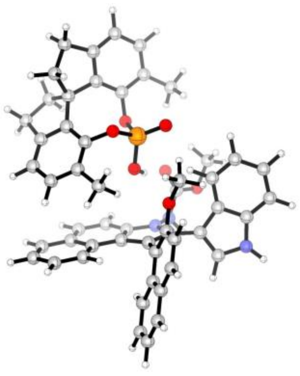

INT-6

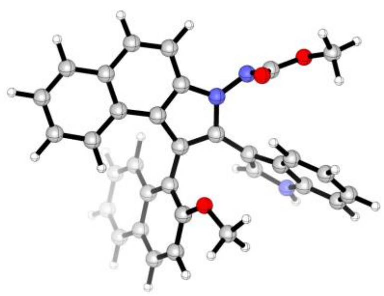

15

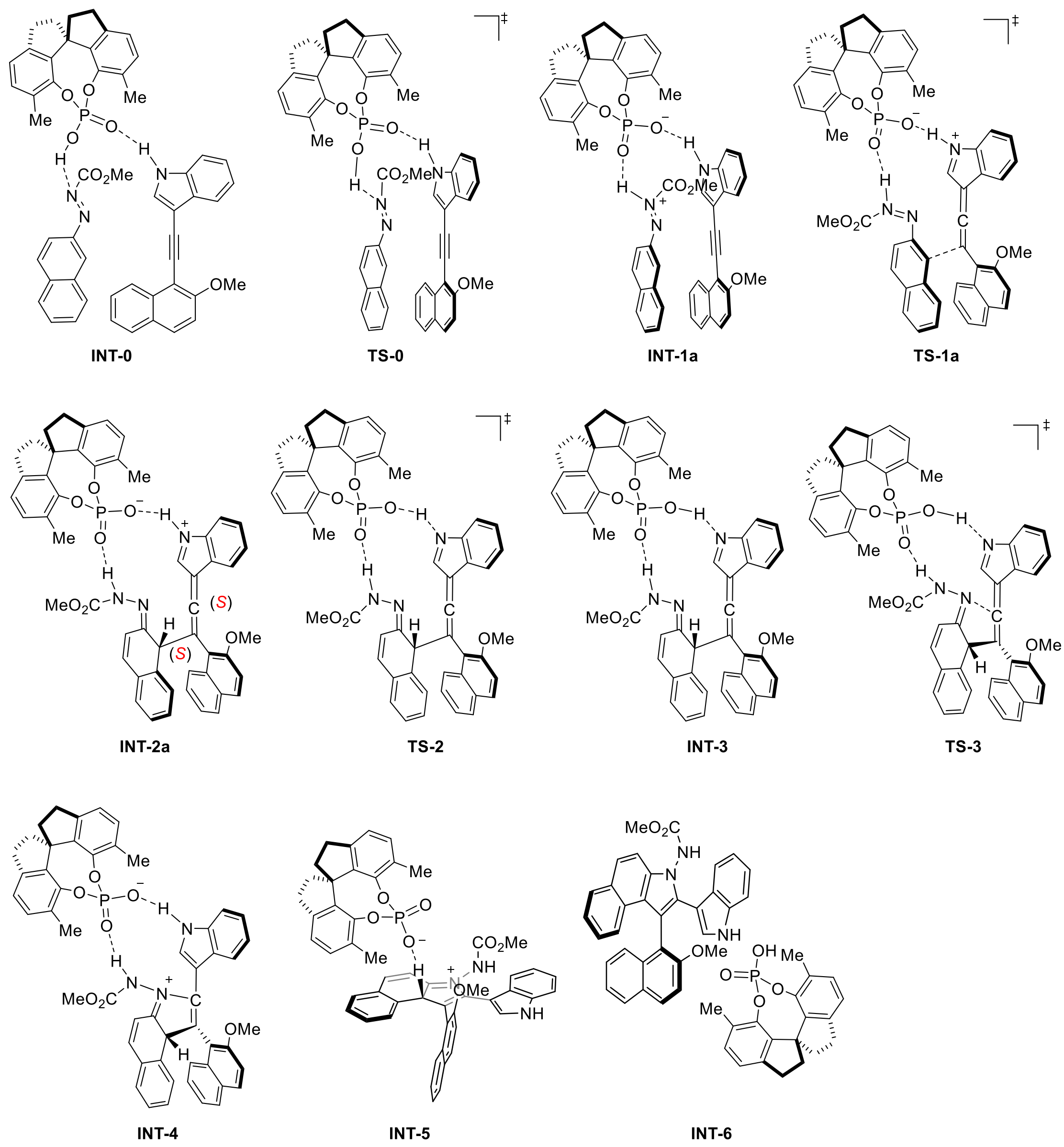

INT-0
TS-0
INT-1a
TS-1a
INT-2a
TS-2
INT-3
TS-3
INT-4
INT-5
INT-6

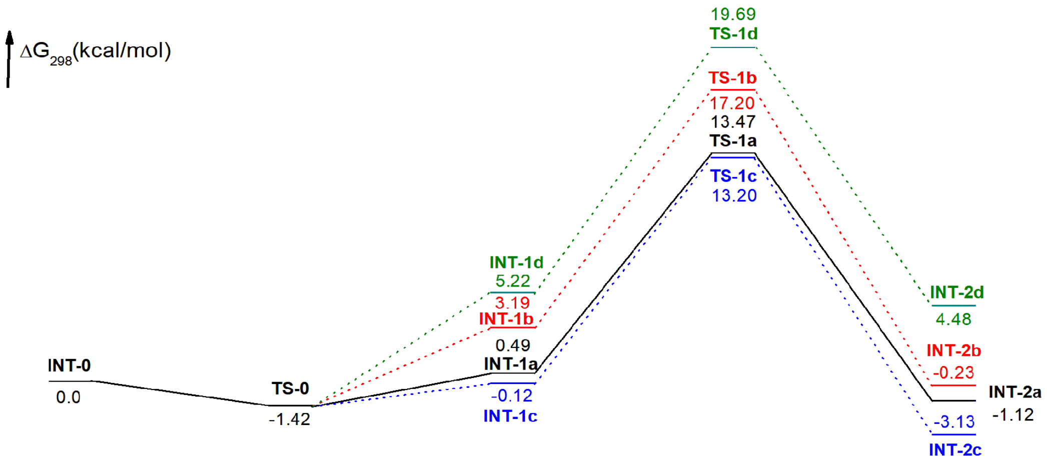

ΔG298(kcal/mol)
INT-0
0.0
TS-0
-1.42
INT-1d
5.22
3.19
INT-1b
0.49
INT-1a
-0.12
INT-1c
19.69
TS-1d
TS-1b
17.20
13.47
TS-1a
TS-1c
13.20
INT-2d
4.48
INT-2b
-0.23
INT-2a
-1.12
-3.13
INT-2c

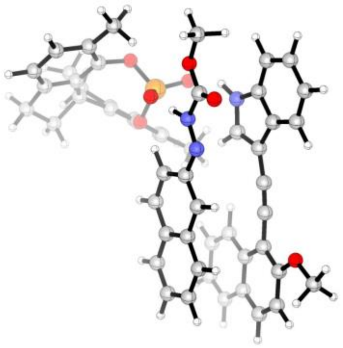
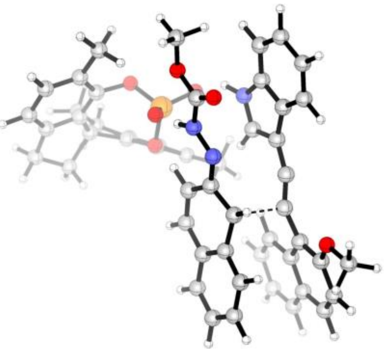
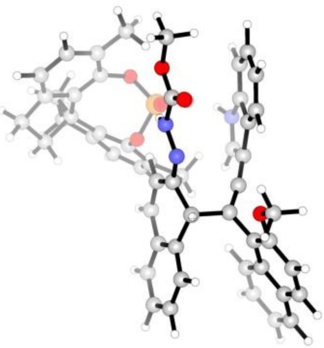

INT-1a **TS-1a (ΔΔG = 12.98)** **INT-2a**

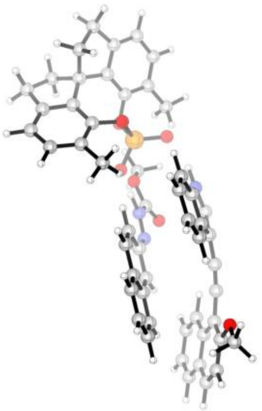
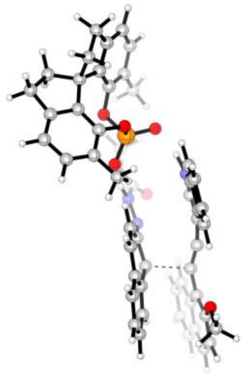
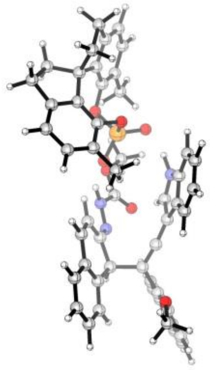

INT-1b **TS-1b (ΔΔG = 14.01)** **INT-2b**

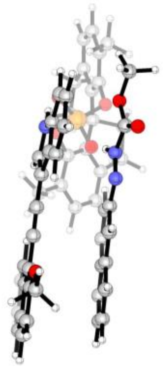
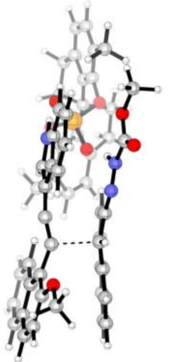
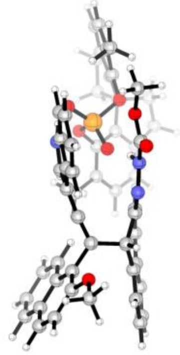

INT-1c **TS-1c (ΔΔG = 13.32)** **INT-2c**

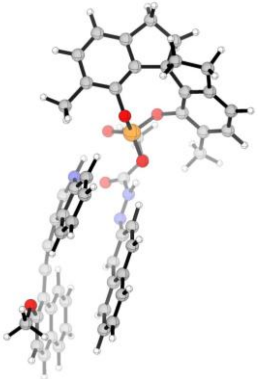
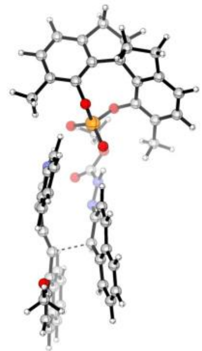
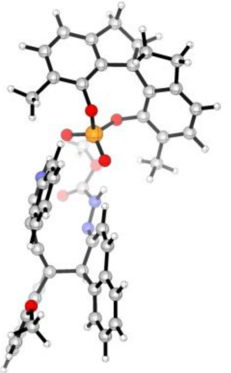

INT-1d **TS-1d (ΔΔG = 14.47)** **INT-2d**

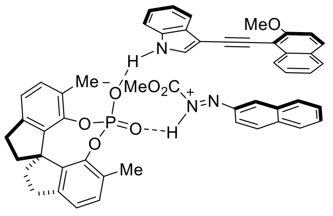

INT-1a

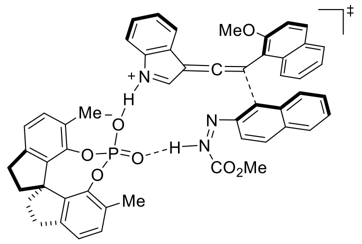

TS-1a

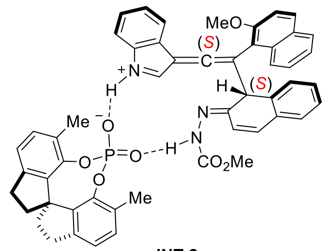

INT-2a

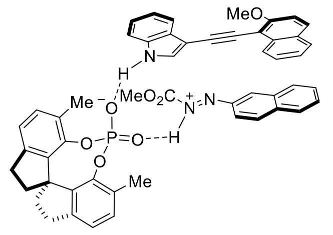

INT-1b

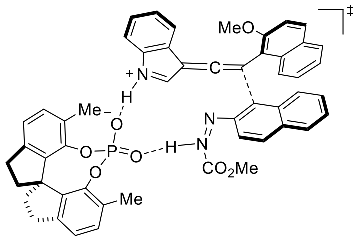

TS-1b

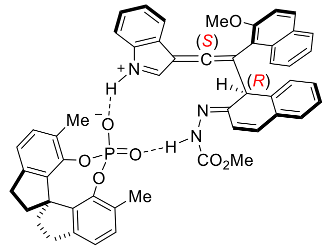

INT-2b

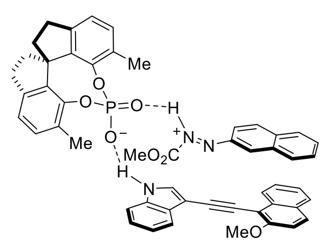

INT-1c

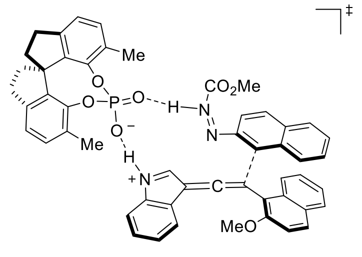

TS-1c

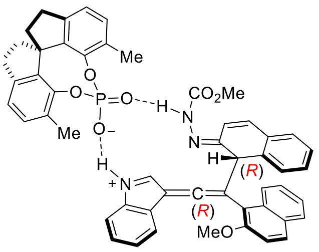

INT-2c

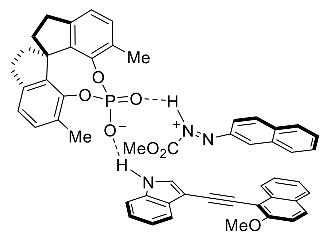

INT-1d

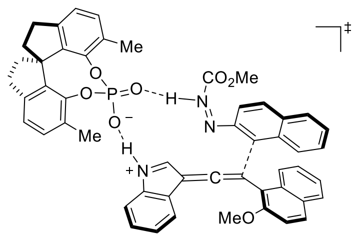

TS-1d

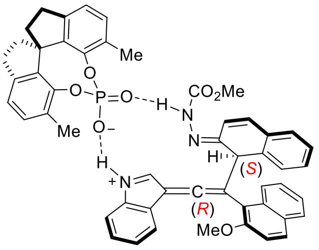

INT-2d

Cartesian coordinates of all optimized structures

Compound: **INT-0**

C	2.25681400	3.36845400	-1.52930200
C	3.21646700	4.37368500	-1.36171400
C	2.82716400	5.69410600	-1.49819500
C	1.49662900	6.03240400	-1.81169400
C	0.52965400	5.05717100	-1.98585900
C	0.92240200	3.72574900	-1.82748800

N	0.18714100	2.56516500	-1.91813300
C	1.00338400	1.50090000	-1.70088200
C	2.29086600	1.92744000	-1.46117000
C	3.36431000	1.05371300	-1.18180100
C	4.20572900	0.21743500	-0.93121900
H	4.24364800	4.11182800	-1.12627000
H	3.55744100	6.48640000	-1.36507300
H	1.22610600	7.07820200	-1.92507000
H	-0.49500300	5.31095600	-2.24077800
C	2.78543000	-3.78223900	-1.19437500
C	3.24169600	-2.49185200	-1.13349800
C	4.57726000	-2.21053400	-0.74119100
C	5.43476300	-3.29741000	-0.42129100
C	4.93346200	-4.62361500	-0.49810500
C	3.63811900	-4.86462900	-0.87203800
H	1.75728000	-3.97639300	-1.48711500
H	2.58320000	-1.66264700	-1.37030900
C	5.07159900	-0.87140200	-0.64204200
C	6.76244800	-3.02396400	-0.01831900
H	5.59951700	-5.44687900	-0.25089100
H	3.26368000	-5.88257200	-0.92617400
C	7.22884500	-1.73684300	0.07619700
C	6.38003200	-0.64936500	-0.22794800
H	7.42197500	-3.85411300	0.22115700
H	8.25138400	-1.56211700	0.39026100
C	4.07394300	-3.28810900	2.65939300
C	2.78253600	-3.06559700	2.25113200
C	2.31421200	-1.74868700	2.03772800
C	3.20319200	-0.66087300	2.25233200
C	4.53464000	-0.92035400	2.66380300
C	4.95957200	-2.20766600	2.86871200
H	0.30558500	-2.31206100	1.44066700
H	4.42641400	-4.30445500	2.80468600
H	2.11179700	-3.89975800	2.06766300
C	0.98081900	-1.47733500	1.60958300
C	2.73057400	0.65490200	2.04095700
H	5.21235300	-0.08221200	2.79953800
H	5.98313500	-2.40294300	3.17113800
C	1.42742500	0.88913800	1.65359600
C	0.53706100	-0.20109500	1.42650100
H	-0.48808700	-0.02902600	1.12926400
O	6.76018300	0.64753500	-0.12549600
C	8.08177900	0.93582400	0.26826700
H	8.81455700	0.52328200	-0.43680000

H	8.16044500	2.02349300	0.26994700
H	8.29656700	0.55842700	1.27677900
N	1.11624900	2.24774900	1.53951700
N	-0.04491200	2.55418200	1.18845300
C	-0.23710700	3.98748000	1.19179700
O	0.45813200	4.80857400	1.71906000
O	-1.38567300	4.22182800	0.55202100
C	-1.78497700	5.59223800	0.52438700
H	-1.99318100	5.94350800	1.53822100
H	-0.99687200	6.20495500	0.08213300
H	-2.68901600	5.62002900	-0.08306100
C	-4.74345100	-3.27223000	-1.95238800
C	-2.96720000	-1.91364200	-1.08655100
C	-2.07992000	-2.41127400	-2.04651700
C	-2.56457500	-3.36457700	-2.94340600
C	-3.89300200	-3.78176900	-2.92112000
H	-1.88983000	-3.75819000	-3.69905700
H	-4.25697100	-4.48794000	-3.66218300
O	-4.40901500	0.74596800	-0.70560800
C	-6.09138700	-1.02459300	2.03668000
C	-5.48222300	-0.79170800	0.79979100
C	-5.15343700	0.50776800	0.45208600
C	-5.51681900	1.60108300	1.24542200
C	-6.18779100	1.33591400	2.43934800
C	-6.45291400	0.03283300	2.85618700
H	-6.92901500	-0.14763100	3.81596500
O	-2.53032100	-0.92477800	-0.20445400
P	-2.80932600	0.61919700	-0.61163200
O	-2.17378100	1.06121300	-1.87069000
O	-2.41982000	1.33977600	0.72510700
C	-5.35612400	-2.09659600	0.03422200
C	-4.27368900	-2.36825600	-0.99543900
H	-6.47496900	2.17144300	3.07227300
C	-6.22374500	-2.50881800	2.28544300
H	-5.93421000	-2.78857200	3.30321200
H	-7.25888600	-2.84505800	2.14198100
C	-5.28853500	-3.10484900	1.21766500
H	-4.26106300	-3.13092200	1.59717300
H	-5.56193400	-4.12039300	0.91565600
C	-6.21970100	-3.52442000	-1.75818200
H	-6.77261900	-3.51674500	-2.70266400
H	-6.39665000	-4.50140300	-1.28968100
C	-6.62659500	-2.37262500	-0.82267900
H	-6.85157000	-1.47754600	-1.41287100

H	-7.50252900	-2.59837100	-0.20722200
H	-0.81223000	2.45234800	-2.05695900
H	-1.50856200	1.76538100	0.77827500
C	-5.16112700	3.00506500	0.83279500
H	-5.38351200	3.17442800	-0.22524600
H	-5.72211900	3.73281700	1.42592600
H	-4.09169300	3.19166200	0.97649400
C	-0.66931000	-1.89584000	-2.14496400
H	-0.66482700	-0.92562300	-2.65220400
H	-0.04269100	-2.58324500	-2.72081300
H	-0.21649200	-1.75766100	-1.15709200
H	3.38810600	1.50650100	2.18289100
H	0.60906000	0.49703000	-1.70552900

Compound: **TS-0**

C	2.12826600	3.36649800	-1.53944800
C	3.02760900	4.42488500	-1.36403900
C	2.56689700	5.71993500	-1.51759100
C	1.22458300	5.98080000	-1.85465000
C	0.31574400	4.95218000	-2.03544100
C	0.78077100	3.64611800	-1.86164100
N	0.10961300	2.44605200	-1.95324800
C	0.97873100	1.43226600	-1.72060100
C	2.23931900	1.92991400	-1.45902400
C	3.34585500	1.10925200	-1.15742700
C	4.20652500	0.29798100	-0.88668600
H	4.06392500	4.22371100	-1.11002100
H	3.24931100	6.55297800	-1.37980700
H	0.89870700	7.00912700	-1.98233500
H	-0.71834500	5.14523000	-2.30468000
C	2.89837800	-3.72170700	-1.32748400
C	3.32007500	-2.42340400	-1.20931600
C	4.63132400	-2.12297500	-0.75462500
C	5.50186600	-3.19856200	-0.43271400
C	5.03684300	-4.53314800	-0.56839000
C	3.76374500	-4.79284600	-1.00197800
H	1.88929700	-3.92978400	-1.67194600
H	2.65190300	-1.60399300	-1.45233800
C	5.09001200	-0.77597500	-0.59753800
C	6.80688100	-2.90606200	0.02842100
H	5.71280200	-5.34775500	-0.31954300
H	3.41730100	-5.81709100	-1.10226600
C	7.23921600	-1.61229600	0.17722400
C	6.37593800	-0.53578100	-0.12670600

H	7.47705500	-3.72762400	0.26794600
H	8.24509500	-1.42398700	0.53413200
C	4.01746300	-3.36743800	2.60752800
C	2.73245600	-3.15687800	2.16897700
C	2.25659300	-1.84543400	1.95179800
C	3.13386200	-0.75124600	2.18754700
C	4.45788800	-0.99759600	2.63049500
C	4.88830400	-2.28162900	2.84480800
H	0.26090100	-2.42480100	1.32059300
H	4.37518000	-4.38116800	2.75805900
H	2.07459000	-3.99711500	1.96866800
C	0.92542300	-1.58472100	1.50259500
C	2.65853100	0.55737700	1.96126300
H	5.12364400	-0.15316500	2.78384100
H	5.90480100	-2.46939200	3.17369400
C	1.35014800	0.78350800	1.55723400
C	0.46719600	-0.31615700	1.31815600
H	-0.55764200	-0.15306400	1.01187200
O	6.71854300	0.76560200	0.03126500
C	8.02557900	1.07479100	0.45895200
H	8.78082700	0.69706100	-0.24155600
H	8.07798800	2.16353700	0.48913500
H	8.22939100	0.67734000	1.46192700
N	1.05812200	2.12757100	1.44788800
N	-0.08970300	2.51216100	1.10831300
C	-0.24522800	3.94953400	1.13612800
O	0.53904200	4.72564800	1.59825300
O	-1.43235700	4.22122700	0.60819000
C	-1.79485400	5.60406400	0.61862000
H	-1.87743900	5.96146600	1.64780800
H	-1.04421400	6.18970700	0.08450600
H	-2.76026000	5.65141000	0.11701500
C	-4.86026400	-3.17636200	-1.97759100
C	-2.99717700	-1.89366400	-1.17268700
C	-2.18723200	-2.38064800	-2.20518100
C	-2.75065600	-3.28470700	-3.10612500
C	-4.08705800	-3.66803400	-3.01755800
H	-2.13470500	-3.66543700	-3.91688400
H	-4.51610300	-4.33372800	-3.76146200
O	-4.35287100	0.77044300	-0.59861800
C	-5.87463100	-1.07179700	2.18536300
C	-5.34887800	-0.79898700	0.91886100
C	-5.00463400	0.50465300	0.59921200
C	-5.27638500	1.56773200	1.46858200

C	-5.86491600	1.26831500	2.69706600
C	-6.14183500	-0.04422200	3.07576900
H	-6.55296600	-0.25501000	4.05928900
O	-2.47981500	-0.95534000	-0.29134000
P	-2.73678900	0.62879600	-0.61840500
O	-2.24560100	1.03535000	-1.96146900
O	-2.18369500	1.29387800	0.64264300
C	-5.31223800	-2.07054200	0.09039300
C	-4.30645400	-2.32394100	-1.01831100
H	-6.07782600	2.08220200	3.38554700
C	-6.03640400	-2.56162600	2.37712000
H	-5.68804200	-2.89715700	3.35921900
H	-7.08888800	-2.86200300	2.29010000
C	-5.19497200	-3.13301200	1.22165200
H	-4.14499100	-3.20267900	1.52709500
H	-5.51769600	-4.12669400	0.89579700
C	-6.32828000	-3.39800700	-1.69917400
H	-6.93953400	-3.33506900	-2.60504500
H	-6.50406100	-4.38938300	-1.26100800
C	-6.64205200	-2.27734500	-0.69193800
H	-6.87800200	-1.35200200	-1.22868100
H	-7.48331800	-2.50705700	-0.03089500
H	-0.88557000	2.26569600	-2.10605800
H	-1.10337600	1.86192400	0.79893800
C	-4.90940000	2.97770100	1.08886700
H	-5.24526400	3.21164200	0.07353200
H	-5.36498300	3.69360500	1.77948100
H	-3.82283200	3.10978200	1.11131300
C	-0.76991000	-1.89898800	-2.36286800
H	-0.76892100	-0.89187100	-2.79246000
H	-0.20432600	-2.55841000	-3.02804500
H	-0.25230800	-1.85017400	-1.39772200
H	3.30621100	1.41475500	2.11129600
H	0.64095000	0.40694300	-1.72943300

Compound: **INT-1a**

C	2.09427900	3.38106000	-1.54324000
C	2.98905300	4.44765200	-1.39720000
C	2.51945500	5.73654700	-1.57371100
C	1.17252500	5.98312900	-1.90259300
C	0.26698000	4.94628600	-2.05244500
C	0.74175600	3.64677700	-1.85859500
N	0.07441900	2.44089700	-1.92031200
C	0.94910800	1.43906300	-1.68075700

C	2.21216500	1.94654200	-1.43490600
C	3.31299400	1.12246400	-1.13095800
C	4.15123300	0.28807100	-0.85526600
H	4.02867100	4.25837700	-1.14796700
H	3.19842700	6.57590100	-1.45972100
H	0.83995100	7.00688600	-2.04871600
H	-0.77166100	5.12785900	-2.31149800
C	2.71986900	-3.68243100	-1.35772700
C	3.18162300	-2.39943600	-1.22235300
C	4.50019300	-2.14564900	-0.76017700
C	5.33610600	-3.25206900	-0.45163700
C	4.83050600	-4.56975700	-0.60591100
C	3.55062400	-4.78398500	-1.04435900
H	1.70565000	-3.85376000	-1.70698600
H	2.53919100	-1.55814300	-1.45970400
C	5.00125400	-0.81573800	-0.58188000
C	6.64942500	-3.00684300	0.01452100
H	5.48068400	-5.40806000	-0.36743500
H	3.17248800	-5.79540300	-1.15868900
C	7.12213200	-1.72996100	0.18381800
C	6.29253500	-0.62261400	-0.10327900
H	7.29346200	-3.85247900	0.24155800
H	8.13296700	-1.57879600	0.54424400
C	3.82664800	-3.45012400	2.59174000
C	2.55054000	-3.16993200	2.16143400
C	2.14541400	-1.83636200	1.94856800
C	3.08534500	-0.79210600	2.17699900
C	4.39672400	-1.10947500	2.61279900
C	4.75714800	-2.41540000	2.82600300
H	0.11238600	-2.30627800	1.33489800
H	4.12854100	-4.48219900	2.73983000
H	1.84668200	-3.97331200	1.96698500
C	0.82316800	-1.50407100	1.51183800
C	2.68508700	0.53809400	1.94675600
H	5.10754500	-0.30222900	2.76330800
H	5.76286800	-2.65891500	3.15088800
C	1.37878800	0.83569300	1.56001300
C	0.43035800	-0.21528500	1.33313400
H	-0.58745200	0.00916900	1.03443300
O	6.67254500	0.66462600	0.07971500
C	7.99105700	0.92857400	0.50445000
H	8.73026700	0.54052400	-0.20717200
H	8.07521000	2.01462500	0.55219200
H	8.18871600	0.50860900	1.49937100

N	1.16230300	2.18007600	1.45370100
N	0.02949900	2.64271600	1.13629000
C	-0.09095700	4.07821400	1.16199400
O	0.75932600	4.82425000	1.54852700
O	-1.30718100	4.37352500	0.73267700
C	-1.63050600	5.76720200	0.74119500
H	-1.61781600	6.14730600	1.76531000
H	-0.91117900	6.31822700	0.13268500
H	-2.63226000	5.82991500	0.31965200
C	-4.75898800	-3.24314500	-1.91523500
C	-2.91915500	-1.88910800	-1.16836500
C	-2.11313600	-2.38681200	-2.20029500
C	-2.66340600	-3.32880500	-3.06934400
C	-3.98811700	-3.74398600	-2.95233800
H	-2.04827500	-3.71484300	-3.87843000
H	-4.41013500	-4.44010500	-3.67212800
O	-4.33555000	0.74149700	-0.65826400
C	-5.77746000	-1.04718700	2.19903500
C	-5.27397500	-0.79902600	0.91870700
C	-4.96319700	0.50132200	0.55299700
C	-5.24782500	1.58176300	1.39782100
C	-5.81421600	1.30589600	2.64203600
C	-6.05762900	-0.00066200	3.06291400
H	-6.45190100	-0.19152800	4.05747500
O	-2.41117900	-0.92205200	-0.32023600
P	-2.70494600	0.66365700	-0.67012800
O	-2.25149900	1.02587900	-2.04565000
O	-2.14928300	1.37677800	0.53963000
C	-5.21441700	-2.09233500	0.12698300
C	-4.21511500	-2.35077000	-0.98674000
H	-6.03591500	2.13496200	3.30959400
C	-5.90033400	-2.53451900	2.43553500
H	-5.53328800	-2.83330600	3.42286000
H	-6.94586400	-2.86348500	2.36909900
C	-5.05782600	-3.11829600	1.28683800
H	-4.00285900	-3.15211500	1.58141300
H	-5.35863200	-4.12917100	0.99406200
C	-6.21738500	-3.49444300	-1.61223200
H	-6.84078400	-3.47210100	-2.51185300
H	-6.36280800	-4.47758200	-1.14495900
C	-6.54758800	-2.35471500	-0.63231400
H	-6.81287200	-1.45073900	-1.19124900
H	-7.37509400	-2.58686500	0.04525600
H	-0.91989500	2.23716300	-2.08829700

H	-0.84908900	2.07372200	0.84790300
C	-4.91107300	2.98696700	0.97523500
H	-5.37043600	3.71425700	1.65171400
H	-3.82600900	3.13331200	0.98009100
H	-5.26265600	3.18643600	-0.04211900
C	-0.71104500	-1.87364100	-2.38873100
H	-0.74407900	-0.86211800	-2.80642000
H	-0.14700300	-2.51354300	-3.07437300
H	-0.17240100	-1.82426600	-1.43448300
H	3.37970200	1.35928300	2.08822300
H	0.61571800	0.41232100	-1.67251300

Compound: **INT-1b**

C	1.81861600	-0.87072400	2.27153300
C	2.42605900	-2.13079900	2.30923600
C	1.61423500	-3.24510900	2.42753100
C	0.21127800	-3.12027600	2.51268400
C	-0.40928300	-1.88304500	2.48523100
C	0.41738400	-0.76260200	2.35971900
N	0.08557400	0.57033400	2.26025400
C	1.21945200	1.30202000	2.08900700
C	2.32040200	0.46826100	2.08677900
C	3.65612000	0.75979400	1.74340700
C	4.79684200	0.81449900	1.33335000
H	3.50624300	-2.21576000	2.22411900
H	2.06035500	-4.23549600	2.45566600
H	-0.39696300	-4.01584000	2.60311700
H	-1.48805400	-1.77421400	2.53836800
C	5.89317000	3.77255500	-1.54640500
C	5.54900600	2.85881300	-0.58387700
C	6.42978900	1.79605400	-0.24697000
C	7.67225200	1.69518700	-0.93120800
C	8.00236000	2.66414800	-1.91546700
C	7.13497100	3.67979100	-2.21902700
H	5.20695900	4.57701200	-1.79323800
H	4.59744800	2.93416300	-0.06591400
C	6.08080100	0.81035500	0.72789900
C	8.53478600	0.61613900	-0.62266700
H	8.95747200	2.58143300	-2.42812800
H	7.39475900	4.41391700	-2.97541600
C	8.19060300	-0.32974900	0.31032200
C	6.95325400	-0.24310000	0.98884200
H	9.48661800	0.53842700	-1.14137300
H	8.87302500	-1.14531500	0.51970500
C	4.89664000	-3.86343700	-1.15205300

C	3.53291200	-3.75314800	-0.99973300
C	2.90493400	-2.49266400	-1.07438300
C	3.71502600	-1.34135200	-1.29587400
C	5.11540800	-1.48457700	-1.47490800
C	5.69490300	-2.72526000	-1.40045400
H	0.88849900	-3.23081900	-0.75273200
H	5.36676700	-4.84039300	-1.08720800
H	2.92567600	-4.63373500	-0.81221700
C	1.49032600	-2.34385900	-0.92285000
C	3.09935800	-0.07713400	-1.29900800
H	5.71672400	-0.60002800	-1.66062400
H	6.76743500	-2.83154000	-1.52962800
C	1.71954600	0.03819100	-1.12103200
C	0.89669300	-1.12325000	-0.94964800
H	-0.17654300	-1.02297300	-0.82716200
O	6.52874900	-1.16162800	1.88578400
C	7.31014100	-2.31485800	2.09985800
H	8.29120300	-2.06884800	2.52517000
H	6.75302100	-2.92012200	2.81542600
H	7.44485100	-2.88559100	1.17199500
N	1.30398500	1.33559600	-1.10617700
N	0.08026000	1.62268600	-0.97418300
C	-0.22881100	3.02840800	-0.96481400
O	0.58067400	3.90576700	-0.86352400
O	-1.53829100	3.12997400	-1.08400600
C	-2.06110600	4.46096800	-1.12045200
H	-3.12356900	4.33850600	-1.31788700
H	-1.89984600	4.95210300	-0.15858700
H	-1.57565700	5.03256600	-1.91420400
C	-7.51434700	0.80121200	1.06916600
C	-5.28642500	1.51192100	0.50023900
C	-5.38310000	2.58451100	1.39772400
C	-6.57334500	2.73689900	2.11036400
C	-7.63473700	1.84692900	1.96965600
H	-6.65347500	3.55910600	2.81708200
H	-8.53303600	1.96398400	2.56972900
O	-3.82215500	-1.11116100	0.65556000
C	-5.67739500	-1.89345500	-2.41190200
C	-5.38358300	-1.37068200	-1.14904500
C	-4.21488400	-1.75262800	-0.50912600
C	-3.40372800	-2.76629700	-1.03592300
C	-3.75961400	-3.31808400	-2.26612500
C	-4.87071200	-2.86751500	-2.97755200
H	-5.09546200	-3.27514600	-3.95950400

O	-4.10922900	1.29773900	-0.20156200
P	-3.00099500	0.29570900	0.47448500
O	-2.61103100	0.75796500	1.83975100
O	-1.94738300	0.10235400	-0.59810500
C	-6.52788900	-0.49341000	-0.67943900
C	-6.35500800	0.64856400	0.30394800
H	-3.13019700	-4.09659900	-2.69029200
C	-6.90815800	-1.22632800	-2.98220600
H	-6.77658200	-0.93377100	-4.02904700
H	-7.77710300	-1.89642500	-2.94394200
C	-7.09323800	-0.01623300	-2.04672800
H	-6.48606300	0.82417100	-2.40078200
H	-8.13116500	0.32361200	-1.97357900
C	-8.48908700	-0.31469200	0.77938600
H	-8.95490200	-0.71222600	1.68677400
H	-9.30097800	0.02917700	0.12455300
C	-7.59679300	-1.34537900	0.06849800
H	-7.08643900	-1.96675600	0.81251900
H	-8.14621500	-2.01040900	-0.60497300
H	-0.89249500	0.88432400	2.21574700
H	-0.77546300	0.93511600	-0.81676500
C	-2.19617700	-3.24363700	-0.27696200
H	-2.48931600	-3.74615100	0.65215300
H	-1.61516600	-3.95208200	-0.87600000
H	-1.55059600	-2.41187400	0.01133300
C	-4.24609800	3.55289400	1.58772600
H	-3.29240700	3.02622700	1.66144600
H	-4.39024600	4.13790800	2.50069100
H	-4.19287100	4.25723300	0.74950400
H	3.68615700	0.82661500	-1.43004400
H	1.18062600	2.37348700	1.94882200

Compound: **INT-1c**

C	-1.64377300	3.51272800	-1.06708800
C	-2.53402300	4.53488400	-0.71895100
C	-2.03741300	5.81396700	-0.54086900
C	-0.66895100	6.09566900	-0.71701900
C	0.23009400	5.10230700	-1.06605500
C	-0.27151800	3.80765900	-1.22266700
N	0.38346600	2.63620100	-1.53606500
C	-0.51742700	1.62644300	-1.59642100
C	-1.78696100	2.09724300	-1.31624000
C	-2.95369600	1.30934200	-1.26136200
C	-3.93510600	0.59903900	-1.18408800

H	-3.59075500	4.31745000	-0.59303400
H	-2.71168600	6.61894300	-0.26489000
H	-0.31448700	7.11351300	-0.58372000
H	1.28347200	5.31423800	-1.22295400
C	-3.32175900	-3.59970600	-1.58913300
C	-3.51051700	-2.24657900	-1.48528000
C	-4.80517000	-1.71112800	-1.25395900
C	-5.90361800	-2.60591100	-1.14674900
C	-5.67535500	-4.00243700	-1.26292800
C	-4.41393900	-4.49229000	-1.47453000
H	-2.32411500	-3.99342800	-1.76190400
H	-2.67004700	-1.56454400	-1.56453600
C	-5.02688200	-0.30541900	-1.10874600
C	-7.19465400	-2.07824400	-0.91104100
H	-6.52370400	-4.67758000	-1.18011800
H	-4.24898700	-5.56193100	-1.56270800
C	-7.39986200	-0.72879000	-0.77084000
C	-6.31117200	0.16729700	-0.85597200
H	-8.03756900	-2.76072400	-0.83958800
H	-8.40186800	-0.35836600	-0.58837900
C	-5.13913800	-3.03374100	2.10276900
C	-3.78274200	-3.04539900	1.87908100
C	-3.06041700	-1.83610000	1.80879100
C	-3.76228800	-0.60903400	1.97787300
C	-5.16345600	-0.62606000	2.19845000
C	-5.83782200	-1.81763000	2.26569300
H	-1.12565700	-2.74711800	1.42665300
H	-5.68551300	-3.97101300	2.13744200
H	-3.25771100	-3.98387000	1.73072800
C	-1.65171400	-1.80686900	1.56744500
C	-3.03743500	0.59498000	1.91893600
H	-5.68843100	0.31930000	2.30023300
H	-6.91098700	-1.83078300	2.42226800
C	-1.65825700	0.59387400	1.71181000
C	-0.95444200	-0.64038300	1.51703700
H	0.11484600	-0.64870200	1.33328000
O	-6.42957700	1.50612000	-0.68428900
C	-7.71474400	2.05380200	-0.49453500
H	-8.37535900	1.83939500	-1.34372600
H	-7.56936500	3.13171900	-0.41729100
H	-8.18028200	1.68667100	0.42962600
N	-1.12393200	1.84974300	1.78106100
N	0.10021800	2.05634800	1.55085800
C	0.56673600	3.39703700	1.79839000

O	-0.06808300	4.24232600	2.35647800
O	1.80868200	3.46300400	1.34859300
C	2.46687400	4.71649300	1.55955700
H	2.67884200	4.84733600	2.62344200
H	1.83529400	5.53262200	1.20497200
H	3.39033300	4.65423000	0.98753800
H	1.40017300	2.47477700	-1.59053200
H	0.79676100	1.34044500	1.10546300
H	-3.53554100	1.55022400	2.04894700
H	-0.19664500	0.61975300	-1.81475500
C	3.63039700	-3.76742000	0.83025200
C	2.84447000	-2.07338400	-0.68491300
C	1.58053800	-2.67690900	-0.73024800
C	1.37713200	-3.83349700	0.02435900
C	2.37935100	-4.36573400	0.83366800
H	0.40116200	-4.31426700	-0.01178500
H	2.18163100	-5.23747000	1.45166900
O	4.08853800	0.39537100	0.62946000
C	7.18137900	-0.99642200	-0.75836300
C	5.91515200	-0.92563200	-0.17130900
C	5.37811000	0.31526300	0.13468600
C	6.11611400	1.49114300	-0.04660600
C	7.39959400	1.38209400	-0.58136500
C	7.93262700	0.15085400	-0.95886400
H	8.91745200	0.09693300	-1.41494900
O	3.04702000	-0.87369200	-1.34048900
P	2.85648500	0.48777100	-0.44228700
O	1.61973100	0.31823700	0.41211700
O	2.97625200	1.64844700	-1.37160500
C	5.36611200	-2.32189600	0.05719200
C	3.88496900	-2.65282000	0.02705700
H	7.98051500	2.28833400	-0.73349600
C	7.50999400	-2.42386900	-1.12667000
H	7.96826100	-2.50763500	-2.11728900
H	8.21280900	-2.86682300	-0.40834100
C	6.13187700	-3.10403600	-1.05127700
H	5.60184400	-2.96965800	-2.00057000
H	6.18457500	-4.17747900	-0.84412600
C	4.86136200	-4.12993500	1.62884200
H	4.63081100	-4.34397300	2.67753700
H	5.35033900	-5.02360200	1.21920400
C	5.74580500	-2.88007800	1.45820400
H	5.48281900	-2.13246000	2.21455300
H	6.81646300	-3.08869100	1.54767700

C	5.51054000	2.82391500	0.30285900
H	5.10815300	2.81269600	1.32196200
H	6.25634100	3.62109500	0.22977500
H	4.67989300	3.04367700	-0.37617400
C	0.46990800	-2.08129000	-1.55344500
H	-0.11481400	-1.37125800	-0.95907800
H	-0.21038000	-2.86149300	-1.90980800
H	0.86578700	-1.54596100	-2.42053600

Compound: **INT-1d**

C	-1.68131800	-1.45094300	1.52309800
C	-2.25014900	-2.66312800	1.11803100
C	-1.40472600	-3.67185600	0.69342900
C	-0.00874100	-3.47893600	0.65554600
C	0.57316400	-2.28555600	1.04816500
C	-0.28382300	-1.27373400	1.49441300
N	-0.01713600	0.01717300	1.91466700
C	-1.19124200	0.66132400	2.16340300
C	-2.25041300	-0.19351300	1.93722700
C	-3.62797400	0.08608900	1.84095800
C	-4.80529500	0.16157400	1.55466200
H	-3.33057600	-2.78061800	1.12281900
H	-1.81823800	-4.62391700	0.37223600
H	0.62936000	-4.28289000	0.30021700
H	1.64276400	-2.12873200	0.97053300
C	-6.67400000	3.86910200	0.52081500
C	-6.07766300	2.69314800	0.89629500
C	-6.75989300	1.45667500	0.74749500
C	-8.07053700	1.46087300	0.19627200
C	-8.66190600	2.69666000	-0.17668100
C	-7.98179500	3.87519600	-0.01996800
H	-6.13937900	4.80646700	0.64174100
H	-5.07662200	2.68883900	1.31692800
C	-6.15131500	0.21429800	1.10790200
C	-8.74118000	0.22648800	0.02301000
H	-9.66638900	2.68928900	-0.59240000
H	-8.44136000	4.81523600	-0.30929700
C	-8.14996500	-0.96277300	0.36803000
C	-6.84310500	-0.97809000	0.90721900
H	-9.74490500	0.22705300	-0.39369100
H	-8.69128000	-1.88995400	0.22019700
C	-5.11220300	-3.02058000	-2.79310500
C	-3.73861500	-3.00840100	-2.70694400
C	-3.06051000	-1.86134600	-2.24290900

C	-3.82981300	-0.72614800	-1.85551000
C	-5.24374100	-0.75842800	-1.96910900
C	-5.87231500	-1.88741700	-2.42892800
H	-1.06646200	-2.69598500	-2.42438600
H	-5.62040000	-3.91115300	-3.15145600
H	-3.16083300	-3.88268500	-2.99243800
C	-1.63550900	-1.81685200	-2.14001900
C	-3.15654000	0.40018000	-1.34810400
H	-5.81687600	0.11677300	-1.67878000
H	-6.95471200	-1.91079500	-2.50668600
C	-1.76588100	0.41043600	-1.25471400
C	-0.98689500	-0.72022800	-1.66683600
H	0.09428300	-0.72457500	-1.56651700
O	-6.18466500	-2.11387100	1.23131200
C	-6.78448700	-3.35630700	0.94157400
H	-7.71067400	-3.50289000	1.51120800
H	-6.05766600	-4.11159200	1.24179800
H	-6.99069100	-3.46089500	-0.13107700
N	-1.28182100	1.57122600	-0.71793000
N	-0.03592500	1.75271900	-0.62629400
C	0.39872700	2.96241200	0.03910900
O	-0.24470100	3.52220900	0.88006700
O	1.57790000	3.27167500	-0.44662400
C	2.35662900	4.19902700	0.32604900
H	1.82721400	5.14902600	0.41744900
H	3.28642800	4.31236500	-0.22715100
H	2.55049300	3.75143900	1.30160500
H	0.90566500	0.46820600	1.89650500
H	0.74593400	1.02997700	-0.82978500
H	-3.70861300	1.27225200	-1.01326400
H	-1.20025900	1.70552500	2.43993500
C	6.39115300	-0.61243600	-2.60688100
C	4.78517400	0.87931300	-1.61421900
C	4.30039000	1.22538400	-2.88281000
C	4.89619100	0.63303400	-3.99675000
C	5.91920400	-0.30381500	-3.87201600
H	4.52280000	0.89164100	-4.98450900
H	6.33254600	-0.78646600	-4.75351300
O	3.77785000	-0.87791300	0.64629800
C	7.07937100	-0.16448000	2.08879700
C	6.07957500	-0.42931000	1.14820400
C	4.79959700	-0.74385500	1.58083000
C	4.52227300	-0.91563000	2.94402800
C	5.55955000	-0.70478300	3.85349000

C	6.82877500	-0.30431600	3.44363400
H	7.60392900	-0.10044900	4.17739400
O	4.17530200	1.39992200	-0.47935600
P	2.99399100	0.49497500	0.20830600
O	1.98592900	0.06560100	-0.83621800
O	2.49650100	1.24606100	1.40715400
C	6.66365000	-0.37177300	-0.24983100
C	5.85943700	0.01319200	-1.47548200
H	5.35243500	-0.82822000	4.91340700
C	8.34589900	0.28705300	1.40069400
H	8.80591600	1.14933300	1.89410000
H	9.09731500	-0.51358700	1.38734100
C	7.84847900	0.61019200	-0.01885800
H	7.46260000	1.63505000	-0.05243300
H	8.62334400	0.51685400	-0.78613500
C	7.45086000	-1.61210700	-2.20748800
H	7.35605400	-2.56030200	-2.74647600
H	8.45805900	-1.22560500	-2.41245200
C	7.20596600	-1.76065700	-0.69552700
H	6.42801600	-2.51120300	-0.51762700
H	8.09717600	-2.05996000	-0.13527500
C	3.14837400	-1.29840600	3.42207900
H	3.15731800	-1.49726400	4.49738100
H	2.43479900	-0.49305400	3.23091700
H	2.78094400	-2.19607700	2.91609100
C	3.15490900	2.18656800	-3.04049400
H	3.03583600	2.47540400	-4.08883300
H	3.30735900	3.09213600	-2.44680300
H	2.22393000	1.72958900	-2.69521600

Compound: **TS-1a**

C	2.04984000	3.30467400	-1.69091100
C	2.93390200	4.37657800	-1.58433500
C	2.43885900	5.65728700	-1.77790100
C	1.08468200	5.87910300	-2.07278900
C	0.18764300	4.82540900	-2.17964200
C	0.69469600	3.54576800	-1.98266600
N	0.02766400	2.31876000	-2.00571000
C	0.88013300	1.34281700	-1.75323800
C	2.17352800	1.86437000	-1.52829600
C	3.17426600	1.05711100	-1.07952900
C	3.83031700	0.23024900	-0.41211200
H	3.97853500	4.20863800	-1.34401600
H	3.10761700	6.50749400	-1.69087300

H	0.73158200	6.89538300	-2.21856300
H	-0.86306900	4.98612800	-2.39660600
C	2.63438100	-3.75042800	-1.38773100
C	3.02588900	-2.46123100	-1.14215100
C	4.35975500	-2.16813100	-0.75338500
C	5.28610500	-3.23931800	-0.64056600
C	4.85090900	-4.56436600	-0.90443000
C	3.55429100	-4.81826900	-1.26489200
H	1.60660400	-3.95376800	-1.67383300
H	2.30974100	-1.65039200	-1.22215300
C	4.79184000	-0.84302300	-0.44523400
C	6.61860100	-2.95239000	-0.25959700
H	5.56813900	-5.37608800	-0.81270500
H	3.22946300	-5.83530500	-1.46152500
C	7.02681200	-1.67314400	0.01753200
C	6.09963900	-0.60823200	-0.05199100
H	7.33063600	-3.77009400	-0.18605100
H	8.05452100	-1.48974100	0.30785800
C	3.81506900	-3.49008200	2.83612100
C	2.55434300	-3.16258200	2.36622700
C	2.25555600	-1.84919800	1.98090900
C	3.26652700	-0.86574900	2.06149600
C	4.52978100	-1.20776700	2.56650800
C	4.80364400	-2.50705900	2.95419100
H	0.17183600	-2.24124600	1.44391600
H	4.03504800	-4.51456700	3.12022900
H	1.78508700	-3.92527000	2.28576100
C	0.93163800	-1.46876500	1.52657100
C	2.99754700	0.45980400	1.53852800
H	5.29426600	-0.43890400	2.63286500
H	5.78691800	-2.76563900	3.33354400
C	1.61543700	0.85289600	1.37966200
C	0.60403800	-0.18481400	1.27397000
H	-0.41769000	0.07558500	1.01466300
O	6.39599900	0.67000600	0.29049200
C	7.71030300	0.97292900	0.70815500
H	8.44146700	0.76689700	-0.08277500
H	7.71192500	2.04042700	0.92941300
H	7.98439100	0.41605800	1.61317600
N	1.43882800	2.15655700	1.31168300
N	0.24355200	2.67634000	1.15434400
C	0.15694400	4.07269300	1.16540100
O	1.05435800	4.83772900	1.40113500
O	-1.10868600	4.40627600	0.88234500

C	-1.37918900	5.80410400	0.91024400
H	-1.20387400	6.20871600	1.91035100
H	-0.74392100	6.32924000	0.19314800
H	-2.43035000	5.89954300	0.63966600
C	-4.88495400	-3.17477500	-1.85936000
C	-2.98583500	-1.89295700	-1.13223000
C	-2.21058600	-2.42888000	-2.16867100
C	-2.80457200	-3.35072800	-3.03011900
C	-4.14427200	-3.71046600	-2.90123000
H	-2.21174400	-3.76563200	-3.84174500
H	-4.60050400	-4.39154400	-3.61452800
O	-4.29510000	0.78661700	-0.61676900
C	-5.76885000	-0.93626900	2.26352100
C	-5.27318200	-0.70897600	0.97656200
C	-4.91143100	0.57670900	0.60563500
C	-5.13218900	1.66784800	1.45592400
C	-5.69183500	1.41547500	2.70836100
C	-5.98897800	0.12097700	3.13176500
H	-6.37610800	-0.05298400	4.13224800
O	-2.42930400	-0.94553900	-0.29598700
P	-2.66700800	0.65113200	-0.64354200
O	-2.24017600	0.96805500	-2.04799500
O	-2.04480800	1.36346200	0.51950400
C	-5.27559200	-2.00335500	0.18454700
C	-4.29769800	-2.30262500	-0.93805500
H	-5.86355100	2.25259200	3.38055600
C	-5.95245300	-2.41710100	2.50127300
H	-5.58715300	-2.73212600	3.48418500
H	-7.01170700	-2.70161300	2.44633300
C	-5.14826500	-3.03506200	1.34304100
H	-4.09233900	-3.11197100	1.62551900
H	-5.49371000	-4.03283100	1.05429700
C	-6.34975300	-3.36620100	-1.54306100
H	-6.97962000	-3.32029200	-2.43733100
H	-6.53039000	-4.34194200	-1.07262900
C	-6.62488500	-2.21266400	-0.56218700
H	-6.85852700	-1.29936700	-1.12011800
H	-7.45481000	-2.41051200	0.12328800
H	-0.98725100	2.08866400	-2.11195200
H	-0.62632800	2.13365700	0.95344800
C	-4.73528700	3.05697000	1.03225100
H	-3.64478300	3.15587700	1.03337800
H	-5.08237800	3.27177200	0.01639400
H	-5.16005000	3.80320100	1.71079200

C	-0.78989300	-1.97412200	-2.36896800
H	-0.24350100	-2.66255100	-3.02127400
H	-0.25909700	-1.90125000	-1.41166500
H	-0.78688700	-0.98204700	-2.83288300
H	3.68800300	1.25900800	1.78662800
H	0.54683300	0.31713500	-1.68799200

Compound: **TS-1b**

C	1.37075100	-0.87008800	2.49193900
C	1.76690200	-2.18177900	2.74990500
C	0.78757400	-3.09677500	3.10930700
C	-0.56342300	-2.72076600	3.20436500
C	-0.97708700	-1.42436200	2.93237800
C	0.01687900	-0.51690900	2.57860500
N	-0.10880500	0.82736800	2.22810800
C	1.08483300	1.32722400	1.92227700
C	2.06600900	0.32433900	2.05194500
C	3.33015300	0.40006000	1.53785400
C	4.34126700	0.38357500	0.81852300
H	2.81053400	-2.46845800	2.66006300
H	1.06642000	-4.12505800	3.31944900
H	-1.30334700	-3.46495400	3.48254800
H	-2.02034200	-1.12888400	2.95618300
C	6.12313700	3.81389600	-1.01625300
C	5.54428500	2.74129000	-0.38715600
C	6.32227600	1.60298500	-0.04401900
C	7.70765600	1.59781000	-0.36149300
C	8.27567100	2.72760600	-1.00518400
C	7.50266500	3.81183700	-1.32830500
H	5.51476700	4.67533100	-1.27377700
H	4.48469400	2.75875400	-0.14604900
C	5.75224100	0.45926200	0.59192100
C	8.47848200	0.45641600	-0.02990900
H	9.33721600	2.71613700	-1.23885700
H	7.94461200	4.67126000	-1.82257900
C	7.92034000	-0.63577600	0.58267900
C	6.53985100	-0.64593000	0.89378900
H	9.53830400	0.45241700	-0.27040400
H	8.54105500	-1.49096500	0.82214100
C	4.91472400	-3.91573100	-1.73713400
C	3.59064300	-3.72707100	-1.37140000
C	3.07134700	-2.43648300	-1.21165600
C	3.93150200	-1.32761800	-1.39433100
C	5.26135900	-1.53259300	-1.79819500

C	5.74877500	-2.81674000	-1.97044700
H	1.03061400	-3.07774000	-0.77382200
H	5.29971800	-4.92310900	-1.86574500
H	2.93723700	-4.58261800	-1.22403800
C	1.66589100	-2.21124700	-0.93107500
C	3.41734000	-0.00383700	-1.13613600
H	5.90129100	-0.67428300	-1.97904800
H	6.77584800	-2.96832600	-2.28796200
C	1.98864800	0.18109800	-1.06422900
C	1.12797600	-0.97547000	-0.91405700
H	0.06151200	-0.83017900	-0.76466300
O	5.91155300	-1.69231100	1.47183600
C	6.61150100	-2.91046400	1.61391000
H	7.43608600	-2.82111200	2.33180700
H	5.88207500	-3.62661200	1.99277500
H	6.99336600	-3.26327600	0.64879100
N	1.62770600	1.44705100	-1.08824700
N	0.36918000	1.81107800	-1.12058600
C	0.14270800	3.19038800	-1.01316200
O	0.96592200	3.99948100	-0.66050500
O	-1.11870600	3.44570800	-1.34816400
C	-1.49775900	4.81987500	-1.26457500
H	-0.76698200	5.44822800	-1.77736300
H	-2.46944200	4.88151700	-1.75055000
H	-1.57634500	5.12721100	-0.21891600
C	-7.40785200	0.87930500	0.93237000
C	-5.25972300	1.57211500	0.09984400
C	-5.38819400	2.80513700	0.75298100
C	-6.54920100	3.04217600	1.48811000
C	-7.55180700	2.08121200	1.60581800
H	-6.65317500	3.99283100	2.00528200
H	-8.42555700	2.27235100	2.22308300
O	-3.64504800	-0.92122100	0.64740000
C	-5.60029500	-2.28964500	-2.14118000
C	-5.27050700	-1.55221300	-1.00019500
C	-4.04071600	-1.74992700	-0.38886400
C	-3.18662500	-2.77583600	-0.81678200
C	-3.57082500	-3.53512000	-1.92191500
C	-4.75617400	-3.28313900	-2.60934100
H	-5.00813900	-3.85489500	-3.49848600
O	-4.09465700	1.28177500	-0.59076600
P	-2.91544300	0.49796000	0.23547000
O	-2.63029000	1.19523900	1.53552500
O	-1.81110700	0.23472700	-0.74963500

C	-6.44078600	-0.67389800	-0.60201400
C	-6.28141200	0.63660900	0.14102100
H	-2.90666200	-4.32328200	-2.26844900
C	-6.91242000	-1.81003200	-2.71721900
H	-6.87379200	-1.69446600	-3.80531300
H	-7.72372000	-2.51707000	-2.49828600
C	-7.12347600	-0.47254000	-1.98497500
H	-6.59997400	0.32895700	-2.51757000
H	-8.17586100	-0.18583700	-1.89325300
C	-8.31973700	-0.32525400	0.93058500
H	-8.69962600	-0.56586800	1.92884300
H	-9.19317500	-0.16046900	0.28549900
C	-7.40549900	-1.42499000	0.36187900
H	-6.81330700	-1.86807700	1.17014700
H	-7.95055600	-2.23209900	-0.13770600
H	-1.05358900	1.23143500	2.03573800
H	-0.46392500	1.16988800	-1.09072100
C	-1.90729700	-3.06441700	-0.08145900
H	-1.27676400	-3.74745100	-0.65970300
H	-1.34861800	-2.14958100	0.12305400
H	-2.10893600	-3.53304500	0.88825900
C	-4.29393300	3.83132400	0.65510100
H	-4.56510400	4.74345600	1.19504700
H	-4.11072500	4.09114900	-0.39232100
H	-3.36185700	3.43437400	1.06747600
H	3.98372500	0.85224000	-1.48747000
H	1.21237600	2.34177900	1.56768200

Compound: **TS-1c**

C	-1.60514800	3.52824400	-1.17208700
C	-2.44599900	4.61130000	-0.92693700
C	-1.87728900	5.87292800	-0.82886700
C	-0.49455300	6.06381100	-0.97492000
C	0.35766700	4.99637900	-1.22095000
C	-0.22312200	3.73642300	-1.30857300
N	0.39065600	2.49821800	-1.50778700
C	-0.51993700	1.54043300	-1.50694200
C	-1.80606500	2.09139200	-1.29347000
C	-2.91584700	1.32761200	-1.09762700
C	-3.79162900	0.56459900	-0.64182700
H	-3.51439500	4.46376600	-0.80557500
H	-2.51129200	6.73081500	-0.62883600
H	-0.08415600	7.06532300	-0.89224000
H	1.42876300	5.12877900	-1.33334200

C	-3.27778500	-3.55014200	-1.67853400
C	-3.43266300	-2.21501800	-1.41548800
C	-4.72617600	-1.65796800	-1.23524300
C	-5.85751600	-2.50899100	-1.35383200
C	-5.66345800	-3.88752600	-1.63032300
C	-4.40318800	-4.40086800	-1.78399300
H	-2.28035100	-3.96109400	-1.80374000
H	-2.56311400	-1.57295700	-1.32149300
C	-4.92419300	-0.28201200	-0.91335100
C	-7.14907000	-1.95591700	-1.18400300
H	-6.53633100	-4.52969500	-1.71637800
H	-4.26348600	-5.45707900	-1.99256200
C	-7.32857100	-0.62826700	-0.89257700
C	-6.20566100	0.21507100	-0.73126900
H	-8.01513600	-2.60416000	-1.28751100
H	-8.33168100	-0.23805500	-0.76767400
C	-5.18227300	-3.17703800	2.29631500
C	-3.81918700	-3.14205400	2.05783400
C	-3.16772600	-1.92444900	1.81920400
C	-3.92620200	-0.73261900	1.81194100
C	-5.30293200	-0.78281900	2.08078800
C	-5.92721400	-1.99230300	2.32401900
H	-1.17554600	-2.78301900	1.56490000
H	-5.67530600	-4.12906400	2.46745400
H	-3.24206900	-4.06225500	2.04439800
C	-1.73828600	-1.85331600	1.59320400
C	-3.27536700	0.50711600	1.44731000
H	-5.87197000	0.14255600	2.07940700
H	-6.99396800	-2.02419000	2.52045400
C	-1.83402600	0.56795700	1.51498700
C	-1.08653000	-0.67794600	1.46953200
H	-0.00932000	-0.66129000	1.34201100
O	-6.29132700	1.51580300	-0.35683600
C	-7.56946200	2.07824900	-0.14844700
H	-8.17254000	2.05816400	-1.06414400
H	-7.39329800	3.11410200	0.14245500
H	-8.11003100	1.56432800	0.65651600
N	-1.36207900	1.79425700	1.60466200
N	-0.06619900	2.02618700	1.62212600
C	0.32869600	3.35046900	1.83070200
O	-0.38072500	4.25144800	2.19334300
O	1.64250100	3.43325000	1.57933900
C	2.21560500	4.71549800	1.82101500
H	1.66938200	5.48646500	1.27323400

H	3.24412500	4.64430800	1.47012100
H	2.19081700	4.94805700	2.88897700
H	1.42196100	2.29171300	-1.52041500
H	0.64338600	1.34086700	1.28075300
H	-3.79530300	1.43850000	1.64529800
H	-0.23912800	0.50099800	-1.59303600
C	3.82123700	-3.80751200	0.77798100
C	2.94699100	-2.12479300	-0.70149700
C	1.71165900	-2.78401400	-0.74706100
C	1.56769100	-3.96351300	-0.01468700
C	2.60066600	-4.46639100	0.77346500
H	0.61166500	-4.48274700	-0.04533300
H	2.44843900	-5.35867100	1.37484200
O	4.01835500	0.37563100	0.67851100
C	7.21072000	-0.78278200	-0.70053900
C	5.93501000	-0.80945900	-0.13022600
C	5.31856500	0.38576800	0.20730000
C	5.98745500	1.60932300	0.08069500
C	7.28236000	1.59683000	-0.43691300
C	7.89338300	0.41404900	-0.85017600
H	8.88556600	0.43566400	-1.29293900
O	3.09484400	-0.90757000	-1.33735400
P	2.80368200	0.42291500	-0.41439100
O	1.54507300	0.21266400	0.37875000
O	2.91069800	1.59767200	-1.34213300
C	5.47383300	-2.24462200	0.04874300
C	4.01604600	-2.66414800	-0.00004300
H	7.81011700	2.54100200	-0.54643700
C	7.63063500	-2.17429100	-1.11219800
H	8.10237500	-2.19592700	-2.09988100
H	8.35302400	-2.59731800	-0.40128900
C	6.29668500	-2.94091600	-1.07492700
H	5.76825000	-2.80861200	-2.02543700
H	6.41446000	-4.01525700	-0.90151100
C	5.07021400	-4.12221800	1.56848200
H	4.85206900	-4.38040800	2.60984200
H	5.61094400	-4.97306000	1.13316400
C	5.87971000	-2.81834900	1.43665500
H	5.56876500	-2.10889500	2.21131100
H	6.96051600	-2.96512600	1.52741100
C	5.29785300	2.88783000	0.47436500
H	4.45873100	3.08418600	-0.20096400
H	4.88888900	2.81081600	1.48779500
H	5.99367000	3.73163600	0.43923500

C	0.56096900	-2.22087200	-1.53859300
H	-0.04425900	-3.02512700	-1.97012100
H	0.91769500	-1.58425900	-2.35255900
H	-0.08759800	-1.61820300	-0.89274900

Compound: **TS-1d**

C	-1.44348400	-1.00153600	2.31394800
C	-1.92820700	-2.30016500	2.45604400
C	-1.00550900	-3.33321300	2.51422700
C	0.37252500	-3.07916500	2.41214100
C	0.86749200	-1.79172400	2.26122800
C	-0.06596700	-0.75924300	2.23058200
N	0.11902700	0.61374900	2.02174600
C	-1.05948000	1.22187400	1.92733200
C	-2.09275700	0.27359700	2.07731900
C	-3.36854100	0.42150900	1.61968600
C	-4.37918000	0.39703200	0.89421400
H	-2.99804500	-2.48384900	2.48874100
H	-1.34992000	-4.35731700	2.62364600
H	1.06973700	-3.91087000	2.43215800
H	1.92515900	-1.60852600	2.11829300
C	-6.24764400	3.92447700	-0.65411900
C	-5.64199800	2.81812400	-0.11546300
C	-6.39912700	1.65244700	0.17635900
C	-7.79375800	1.65485400	-0.09791300
C	-8.39022600	2.81897700	-0.64781500
C	-7.63622000	3.92948500	-0.92304400
H	-5.65535500	4.80773400	-0.87251600
H	-4.57700200	2.82686100	0.09942100
C	-5.79935300	0.47533400	0.71533900
C	-8.54496700	0.48656800	0.18137300
H	-9.45851300	2.81271200	-0.84862100
H	-8.09999200	4.81514700	-1.34577000
C	-7.95814400	-0.63853800	0.69943400
C	-6.56743900	-0.65608200	0.96132300
H	-9.61216600	0.48855200	-0.02393200
H	-8.56301600	-1.51497500	0.89925000
C	-5.40838800	-3.60629200	-2.04885500
C	-4.07920300	-3.60261000	-1.65604900
C	-3.42295800	-2.39961400	-1.36434100
C	-4.14999300	-1.18752600	-1.43971400
C	-5.48766000	-1.20498600	-1.86888600
C	-6.11120600	-2.40293300	-2.17331200
H	-1.47967700	-3.31113500	-0.96451200

H	-5.89947400	-4.54648200	-2.28246200
H	-3.52559500	-4.53549000	-1.59239300
C	-2.00721500	-2.36625900	-1.05544700
C	-3.49331500	0.04597600	-1.06377400
H	-6.02785400	-0.26823500	-1.96734500
H	-7.14390000	-2.40508400	-2.50786500
C	-2.05152200	0.05156800	-0.99476100
C	-1.33103600	-1.20687600	-0.92767300
H	-0.26080600	-1.21199000	-0.73322800
O	-5.91073700	-1.73623400	1.43680000
C	-6.59221400	-2.97196300	1.50197500
H	-7.38663100	-2.95495800	2.25821900
H	-5.84031400	-3.70778400	1.78786300
H	-7.00951800	-3.24573500	0.52645000
N	-1.52509500	1.26028700	-0.96315200
N	-0.22809900	1.43182900	-1.04249400
C	0.24869400	2.73536100	-0.80298300
O	-0.35787300	3.58353000	-0.19510100
O	1.45132400	2.84753300	-1.34020000
C	2.24457000	3.95227100	-0.89340700
H	1.74772500	4.89461100	-1.13275100
H	3.18685000	3.85310200	-1.42878000
H	2.41416700	3.85369800	0.17938900
H	1.03466300	1.05230100	1.77511700
H	0.47941500	0.66583400	-1.03469100
H	-3.94886800	0.98294300	-1.36764600
H	-1.13525300	2.27216600	1.67602700
C	6.09629600	-1.52958200	-2.48150700
C	4.43299400	0.12109600	-1.92348000
C	3.76216500	-0.12585400	-3.12864100
C	4.30452500	-1.07612000	-3.99664300
C	5.45039900	-1.79859100	-3.67760800
H	3.78851100	-1.27445600	-4.93304100
H	5.82279600	-2.56524600	-4.35187700
O	3.87717400	-0.64783000	0.94893100
C	7.20372000	0.80463400	1.52306900
C	6.15233700	0.11804300	0.90760700
C	4.96906100	-0.09089100	1.60292200
C	4.86173700	0.27084500	2.95395800
C	5.95282800	0.89698500	3.55697500
C	7.11449700	1.19392100	2.84881500
H	7.93010100	1.72764800	3.32925300
O	3.91198600	1.02067300	-1.00213500
P	2.89938900	0.40015400	0.12826700

O	1.82205500	-0.42822000	-0.51239900
O	2.49842800	1.53017600	1.03951000
C	6.58260100	-0.33996800	-0.47274000
C	5.61612100	-0.53900500	-1.62214500
H	5.87217800	1.18813500	4.60123700
C	8.32771000	1.04115500	0.54181300
H	8.72371900	2.06037800	0.59760400
H	9.16820400	0.35971600	0.72931200
C	7.65271600	0.73803200	-0.80708700
H	7.14060300	1.63367800	-1.17570100
H	8.35125800	0.40630000	-1.58165600
C	7.31218300	-2.20019900	-1.88706300
H	7.28523700	-3.28942200	-1.99496400
H	8.23454600	-1.85223500	-2.37109700
C	7.24684800	-1.74656600	-0.41819300
H	6.59199400	-2.41810700	0.14789400
H	8.22197100	-1.72913500	0.07858900
C	3.60443400	0.00512200	3.73583700
H	3.71594100	0.34127900	4.77059100
H	2.75766600	0.53520100	3.29288500
H	3.36301600	-1.06281300	3.75468200
C	2.47262800	0.56703800	-3.47417900
H	2.33986800	0.61297800	-4.55961400
H	2.43521900	1.57912700	-3.06881800
H	1.62660700	0.01839300	-3.04738100

Compound: **INT-2a**

C	1.53481900	2.50117600	-1.87036600
C	2.07066700	3.69402200	-1.40730600
C	1.34889800	4.85972400	-1.64679200
C	0.11961600	4.83138500	-2.31708200
C	-0.43407300	3.63679700	-2.76711000
C	0.30404100	2.48893300	-2.53363500
N	-0.04080400	1.15192500	-2.81720000
C	0.86816000	0.34197200	-2.35423500
C	1.92152600	1.09371600	-1.71952500
C	2.78833500	0.56179400	-0.86861500
C	3.51686800	0.13116600	0.11324000
H	2.99390900	3.70434400	-0.83845700
H	1.73228800	5.80462700	-1.27663300
H	-0.42420100	5.75811600	-2.47044000
H	-1.40471900	3.59621700	-3.24841300
C	4.86763000	-3.75367400	-1.27049400
C	4.43158900	-2.51990700	-0.86825100

C	5.35938000	-1.50279300	-0.51329200
C	6.74991900	-1.79528500	-0.58882100
C	7.16824200	-3.08122600	-1.02245300
C	6.25103100	-4.04126100	-1.35491000
H	4.14377800	-4.52249900	-1.52303600
H	3.36855100	-2.31759800	-0.79200200
C	4.95241400	-0.21906100	-0.05693000
C	7.67950800	-0.79650500	-0.21441100
H	8.23343900	-3.29036400	-1.07966200
H	6.57886100	-5.02377800	-1.67978400
C	7.27299900	0.43695400	0.22489000
C	5.89126900	0.73083500	0.30689900
H	8.74130100	-1.02024000	-0.27499900
H	8.01094600	1.17961000	0.50435800
C	3.19272000	-4.13833700	2.53394000
C	2.00858100	-3.64372300	1.99977600
C	1.86730800	-2.28267000	1.71598200
C	2.94486000	-1.41498100	1.96629600
C	4.11570900	-1.91331300	2.52830000
C	4.24462500	-3.27012300	2.81077300
H	-0.19228800	-2.43919400	0.97305300
H	3.29086600	-5.19898700	2.74436300
H	1.17557000	-4.31440300	1.80457000
C	0.60831200	-1.74052800	1.20538900
C	2.84691900	0.02511800	1.51278500
H	4.94396500	-1.23755100	2.72395700
H	5.16860000	-3.64829700	3.23683700
C	1.42353700	0.54645600	1.42187500
C	0.37856600	-0.42047200	1.09317000
H	-0.59466200	-0.07298500	0.76488000
O	5.39766700	1.91759900	0.73594900
C	6.29035700	2.90186100	1.21601800
H	6.96372300	3.25276000	0.42452700
H	5.66265200	3.72790000	1.55014000
H	6.88110800	2.52954900	2.06179000
N	1.31575300	1.82509700	1.51591900
N	0.12999200	2.42210900	1.27110200
C	0.10028000	3.79697200	1.32718400
O	0.99180100	4.53254800	1.68081400
O	-1.12162100	4.20807000	0.93128100
C	-1.31680300	5.61253700	0.95469700
H	-1.19313500	6.00706600	1.96684100
H	-0.60875800	6.11460500	0.28958100
H	-2.33855600	5.76875200	0.60738500

C	-5.27421800	-3.42709600	-0.98357100
C	-3.21261500	-2.19542300	-0.90449500
C	-2.63382000	-3.07183400	-1.83083800
C	-3.40631500	-4.13123400	-2.30641100
C	-4.73100300	-4.30366300	-1.90993200
H	-2.96880100	-4.81396500	-3.03072300
H	-5.32887800	-5.10720500	-2.33140100
O	-4.25891000	0.63209100	-1.06149600
C	-5.37704200	-0.02658900	2.36837500
C	-5.07615000	-0.23129000	1.01902700
C	-4.69646100	0.84898600	0.23716600
C	-4.71011300	2.15454500	0.74346900
C	-5.07869400	2.33171600	2.07721800
C	-5.38764900	1.25214500	2.90264200
H	-5.62056100	1.41377500	3.95174200
O	-2.48210000	-1.11301000	-0.45367300
P	-2.66360900	0.33210800	-1.22923600
O	-2.42709200	0.17195500	-2.71284300
O	-1.81426600	1.28639200	-0.45537400
C	-5.28026500	-1.69140700	0.65826600
C	-4.50332900	-2.39689600	-0.43829200
H	-5.08538600	3.33914700	2.48580700
C	-5.61525600	-1.34878900	3.05959100
H	-5.12312200	-1.40585700	4.03588500
H	-6.68625000	-1.52270700	3.22894800
C	-5.04042000	-2.35450300	2.04566500
H	-3.96049000	-2.45811900	2.19989700
H	-5.48842300	-3.35080500	2.11574900
C	-6.68304400	-3.37183800	-0.44084300
H	-7.43771500	-3.51698200	-1.22049600
H	-6.84754500	-4.15138000	0.31493800
C	-6.73705100	-1.96629800	0.18461500
H	-6.99821700	-1.22926500	-0.58258300
H	-7.46370900	-1.87789200	0.99819300
H	-1.03912700	0.80794000	-3.01577700
H	-0.64343100	1.95233800	0.78248400
C	-4.29368500	3.31467800	-0.12045700
H	-4.72746000	3.23570300	-1.12213500
H	-4.61787700	4.26032000	0.32589600
H	-3.20431800	3.32975000	-0.22801300
C	-1.23160700	-2.83758500	-2.32691400
H	-1.22768900	-1.99788600	-3.03058400
H	-0.84335200	-3.72468400	-2.83596300
H	-0.55765400	-2.58630800	-1.49952400

H	3.43210600	0.68697200	2.15910100
H	0.74592800	-0.73234800	-2.38537900

Compound: **INT-2b**

C	1.08813700	-0.51857100	2.65260300
C	1.36119000	-1.72536800	3.28294700
C	0.37938500	-2.25690700	4.11523500
C	-0.84885700	-1.60949600	4.30221500
C	-1.13764200	-0.40622800	3.66498300
C	-0.13991300	0.11685900	2.85939200
N	-0.16424100	1.31365300	2.11276200
C	0.96254900	1.47472400	1.47629800
C	1.82621900	0.34063100	1.72329100
C	2.88219600	0.11011100	0.95464400
C	3.81832900	0.00373500	0.06639900
H	2.30907100	-2.23046200	3.12709900
H	0.56433400	-3.19816500	4.62317700
H	-1.59632000	-2.06179600	4.94585200
H	-2.09691600	0.08915100	3.76232100
C	5.51479500	3.81567900	-0.90852800
C	4.95734900	2.61115800	-0.56368400
C	5.75736500	1.59968200	0.03734700
C	7.13493900	1.86197200	0.27675200
C	7.67590000	3.12005300	-0.09452800
C	6.88537400	4.07663900	-0.67538400
H	4.88770000	4.57772100	-1.36074400
H	3.89947000	2.44103300	-0.75034500
C	5.22762900	0.33116300	0.41047400
C	7.92493600	0.85601600	0.88563500
H	8.72923100	3.31165000	0.09413300
H	7.30615700	5.03776200	-0.95444700
C	7.39269200	-0.35069800	1.25796500
C	6.02055900	-0.61758200	1.02802100
H	8.97802700	1.05561800	1.06593000
H	8.02141000	-1.09491300	1.73316000
C	4.50342600	-4.50707300	-2.01835800
C	3.17096900	-4.12739200	-1.91782300
C	2.81943900	-2.78419900	-1.74998200
C	3.83274900	-1.81670900	-1.66374200
C	5.16470500	-2.19990800	-1.79667400
C	5.50329100	-3.53857900	-1.97057100
H	0.66318600	-3.15546000	-1.74238100
H	4.76102900	-5.55321500	-2.15342000
H	2.38476300	-4.87523500	-1.98387000

C	1.41704900	-2.37222700	-1.72143200
C	3.46694200	-0.37253700	-1.39457700
H	5.94436500	-1.44368600	-1.75952900
H	6.54607100	-3.82319100	-2.07659100
C	2.01252200	-0.01400400	-1.67617200
C	1.02584000	-1.08653300	-1.72163600
H	-0.03179700	-0.84192400	-1.71737300
O	5.41978000	-1.78080700	1.38223200
C	6.22941600	-2.91741800	1.59886100
H	6.78917600	-2.84396500	2.53950800
H	5.54389300	-3.76369700	1.65306200
H	6.92465100	-3.07400700	0.76554900
N	1.81469100	1.25501500	-1.72317500
N	0.57324500	1.77579100	-1.86809900
C	0.45142600	3.10433000	-1.52117800
O	1.32316500	3.80427600	-1.04349800
O	-0.80130500	3.50900800	-1.76622800
C	-1.11114900	4.82085800	-1.30568500
H	-2.11312200	5.03055500	-1.67630700
H	-1.10443300	4.85178200	-0.21258400
H	-0.39443000	5.54692700	-1.69475700
C	-7.23410600	0.73612500	0.63152600
C	-5.14603200	1.48978500	-0.28795500
C	-5.45971500	2.81545500	0.03590400
C	-6.68277800	3.06614400	0.65747300
C	-7.56410600	2.03583000	0.98123500
H	-6.93440200	4.09087700	0.91938100
H	-8.49084500	2.25183900	1.50616500
O	-3.27050000	-0.55666800	0.88937600
C	-4.87161100	-2.81167000	-1.52053300
C	-4.70541100	-1.78585800	-0.58466300
C	-3.49652700	-1.65982600	0.08252100
C	-2.48752600	-2.62235900	-0.06745600
C	-2.70833300	-3.66891600	-0.96239400
C	-3.87890600	-3.75787900	-1.71475400
H	-4.00451400	-4.55462100	-2.44305300
O	-3.91821500	1.20674300	-0.86091000
P	-2.69064200	0.78575600	0.12960600
O	-2.51898600	1.80778900	1.23062000
O	-1.53336400	0.41871300	-0.74316800
C	-5.99630500	-1.00082500	-0.44562700
C	-6.04269100	0.46107200	-0.04546000
H	-1.93016500	-4.41826200	-1.08998200
C	-6.19941700	-2.66758900	-2.22731900

H	-6.11563200	-2.81424100	-3.30902500
H	-6.92682500	-3.40322100	-1.85891300
C	-6.61659500	-1.23345500	-1.85329300
H	-6.16641700	-0.52156000	-2.55370100
H	-7.69987900	-1.07712900	-1.86287600
C	-7.99031300	-0.54342100	0.90275800
H	-8.39637100	-0.58439300	1.91875000
H	-8.83777800	-0.65672800	0.21357100
C	-6.91703800	-1.61690500	0.64837800
H	-6.32344000	-1.76911800	1.55664700
H	-7.32969400	-2.58568700	0.34964300
H	-1.10374600	1.76178700	1.84461500
H	-0.28068800	1.21279200	-1.73365200
C	-1.21197700	-2.51939600	0.72167800
H	-1.41523200	-2.52707400	1.79658300
H	-0.54370100	-3.35599300	0.49663800
H	-0.68686500	-1.58830400	0.49440000
C	-4.48084600	3.91625000	-0.26767200
H	-3.59149500	3.81288300	0.36161900
H	-4.93085400	4.89822900	-0.09364800
H	-4.15088200	3.86046000	-1.31047200
H	4.10219300	0.28122300	-2.00144900
H	1.15375000	2.30528700	0.80466800

Compound: **INT-2c**

C	-1.62433100	3.38475900	-0.96780400
C	-2.41511000	4.43388300	-0.52288500
C	-1.86079500	5.70987600	-0.52147500
C	-0.54737800	5.93291200	-0.94984200
C	0.26066900	4.88500300	-1.38261900
C	-0.30631100	3.62111300	-1.37693200
N	0.28462300	2.38888200	-1.72062200
C	-0.56499000	1.41502900	-1.54488000
C	-1.81817700	1.93312100	-1.05738800
C	-2.74964800	1.15926700	-0.51463400
C	-3.59163800	0.38342000	0.09843600
H	-3.42468100	4.25454500	-0.16909200
H	-2.45287300	6.54781800	-0.16883500
H	-0.14533900	6.94091900	-0.93249800
H	1.28758700	5.04238400	-1.69443400
C	-3.33385800	-3.11618800	-2.40516800
C	-3.40767900	-1.99654000	-1.61985800
C	-4.64397200	-1.31593400	-1.44426500
C	-5.80195100	-1.83223200	-2.08961100

C	-5.68840900	-2.99134700	-2.90122600
C	-4.48279100	-3.62039800	-3.05928600
H	-2.38196800	-3.62449900	-2.52675700
H	-2.51996200	-1.63243900	-1.11229200
C	-4.77046600	-0.15397600	-0.63394000
C	-7.03991300	-1.17470200	-1.89780800
H	-6.57972500	-3.37222100	-3.39324400
H	-4.40410200	-4.50694300	-3.68068900
C	-7.14909400	-0.06405600	-1.10160500
C	-6.00010300	0.45075700	-0.45669700
H	-7.92396500	-1.56771400	-2.39281500
H	-8.11422900	0.41123300	-0.97238400
C	-4.77231600	-3.96606500	2.21787000
C	-3.42037600	-3.73643400	1.99513200
C	-2.93486700	-2.43584200	1.83417200
C	-3.83171600	-1.35744100	1.89042300
C	-5.18026700	-1.59351700	2.13639000
C	-5.65435000	-2.89175700	2.29848700
H	-0.84524500	-3.03061800	1.54864400
H	-5.13727600	-4.98173200	2.33646100
H	-2.72544300	-4.57105100	1.94921900
C	-1.50836800	-2.17467000	1.65225400
C	-3.33051300	0.03505400	1.59416900
H	-5.86532300	-0.75125300	2.18833400
H	-6.71038100	-3.06390700	2.48232100
C	-1.84344400	0.23159000	1.83745500
C	-0.98157500	-0.93728500	1.67197300
H	0.09024200	-0.80898200	1.57210100
O	-6.02041900	1.52365900	0.37563100
C	-7.24965500	2.17737500	0.61365800
H	-7.66775600	2.59550700	-0.31001200
H	-7.02368400	2.98869600	1.30589400
H	-7.98191100	1.50292400	1.07430400
N	-1.50329300	1.46109400	2.00671500
N	-0.19715100	1.81375700	1.94322600
C	0.08326100	3.15817400	2.05810600
O	-0.66297800	4.02594000	2.44259500
O	1.36447400	3.35616000	1.67770900
C	1.82495300	4.69216900	1.83100000
H	1.85701400	4.97007600	2.88830600
H	1.17249900	5.38896800	1.29956400
H	2.82859100	4.70603700	1.40606600
H	1.32758700	2.17524000	-1.82445600
H	0.49679000	1.22751900	1.45571700

H	-3.89297300	0.77764200	2.16943500
H	-0.28545000	0.38146300	-1.68622400
C	3.98366000	-3.64229100	0.97058700
C	2.95299900	-2.15035500	-0.60911200
C	1.76483400	-2.89272400	-0.55888200
C	1.72617300	-4.01631800	0.26736800
C	2.81509600	-4.38253000	1.05696300
H	0.81101500	-4.60346800	0.30798000
H	2.74352200	-5.23227500	1.73044000
O	3.85850300	0.51990700	0.52667600
C	7.11252100	-0.50444600	-0.81397200
C	5.84962500	-0.58111700	-0.22031700
C	5.15399300	0.58805900	0.04497800
C	5.73426900	1.84403600	-0.16837700
C	7.02102200	1.88684400	-0.70433100
C	7.70729000	0.72445400	-1.05275700
H	8.68991800	0.78567700	-1.51281600
O	2.99800200	-0.99033200	-1.35886700
P	2.64709400	0.40457000	-0.56098000
O	1.38805300	0.23554600	0.23521200
O	2.72363400	1.48370600	-1.61043800
C	5.49934700	-2.02826500	0.07290000
C	4.07556900	-2.55375900	0.09854500
H	7.48127700	2.85588600	-0.88078300
C	7.62346800	-1.88851900	-1.13946800
H	8.07573000	-1.94561100	-2.13483400
H	8.38882400	-2.20864800	-0.41987100
C	6.34963100	-2.74428000	-1.01708600
H	5.79568000	-2.72000900	-1.96194700
H	6.54910300	-3.79176900	-0.76991300
C	5.27174400	-3.80488700	1.74351800
H	5.09956900	-3.99659400	2.80752200
H	5.86224400	-4.64606500	1.35683100
C	5.97875700	-2.46095300	1.48862200
H	5.63412200	-1.71723900	2.21538700
H	7.06923700	-2.52140700	1.55915500
C	4.96196800	3.09396200	0.15744200
H	4.54570700	3.03826100	1.16886300
H	5.60378000	3.97742200	0.08784200
H	4.11992300	3.20708000	-0.53290000
C	0.55741500	-2.46301000	-1.34859400
H	0.05466300	-1.63691700	-0.83494200
H	-0.15568500	-3.28812100	-1.44543800
H	0.83830600	-2.12130000	-2.34965900

Compound: **INT-2d**

C	-1.47137200	-0.79213200	2.45248000
C	-1.94238100	-2.04010600	2.83649000
C	-1.00062100	-3.01551500	3.14575100
C	0.37201400	-2.75577300	3.04148600
C	0.85078200	-1.51186800	2.64413500
C	-0.10012600	-0.53664500	2.38179200
N	0.08455700	0.78211500	1.90325100
C	-1.06306900	1.34282700	1.63972800
C	-2.12505700	0.39701200	1.90169400
C	-3.23785100	0.40526000	1.18269600
C	-4.13685500	0.28092700	0.26069200
H	-3.00856800	-2.24157400	2.86391900
H	-1.33268100	-4.00275700	3.45185500
H	1.08191300	-3.54814300	3.25531800
H	1.90827600	-1.33229200	2.49537100
C	-5.87608700	3.90772500	-1.22349700
C	-5.30760600	2.77393900	-0.70295500
C	-6.12075600	1.77820400	-0.09359600
C	-7.52642000	1.98481400	-0.02750800
C	-8.07903500	3.17261100	-0.57268300
C	-7.27456400	4.11288100	-1.16041100
H	-5.24339900	4.65989500	-1.68449000
H	-4.22964800	2.63942500	-0.74824300
C	-5.57668600	0.57850900	0.44414800
C	-8.33243400	0.99229100	0.58255500
H	-9.15405200	3.32296900	-0.51426800
H	-7.70493500	5.01888700	-1.57549700
C	-7.78996900	-0.15017700	1.10979700
C	-6.39124100	-0.36346000	1.04411700
H	-9.40666700	1.14926400	0.63366600
H	-8.43263000	-0.88753700	1.57688100
C	-5.32850900	-4.15623700	-1.59296300
C	-3.99773600	-4.00059900	-1.22228500
C	-3.42849300	-2.72825400	-1.11823300
C	-4.22572800	-1.59895400	-1.36891200
C	-5.54959900	-1.76193200	-1.76636700
C	-6.10268600	-3.03503300	-1.88049300
H	-1.44016800	-3.44736200	-0.54947800
H	-5.75523400	-5.15125000	-1.67707200
H	-3.38055500	-4.87414500	-1.02788700
C	-2.00668800	-2.55984500	-0.81894700
C	-3.64739000	-0.22047700	-1.13675600

H	-6.15523900	-0.88600700	-1.98244500
H	-7.13548100	-3.14892400	-2.19627100
C	-2.13142000	-0.16529500	-1.22694000
C	-1.37604400	-1.37728900	-0.92123900
H	-0.30682500	-1.31277300	-0.72771900
O	-5.78546500	-1.47003900	1.54039700
C	-6.56619200	-2.63108900	1.74154600
H	-7.21712700	-2.53433300	2.61925400
H	-5.85477800	-3.44064300	1.90813600
H	-7.16580600	-2.86157600	0.85387700
N	-1.66666300	1.01907300	-1.41519200
N	-0.33344500	1.23826800	-1.45869900
C	0.08697300	2.54055200	-1.25773300
O	-0.58389900	3.43192300	-0.77849400
O	1.35203600	2.65323800	-1.65702200
C	2.05281400	3.81709000	-1.21846400
H	1.57878900	4.71715200	-1.61614000
H	3.06258900	3.69808500	-1.60745700
H	2.07975300	3.83875800	-0.12844400
H	1.02939800	1.18187300	1.59061100
H	0.36998900	0.49956600	-1.33590200
H	-4.06645500	0.48871300	-1.85752000
H	-1.13694600	2.31487300	1.16099500
C	6.05594200	-1.78017400	-2.25174900
C	4.33956700	-0.12937900	-1.88672800
C	3.66367900	-0.55505700	-3.03729400
C	4.23107700	-1.59159100	-3.78271400
C	5.40569800	-2.22614200	-3.39169300
H	3.71090100	-1.92820700	-4.67635700
H	5.79766900	-3.06120500	-3.96624000
O	3.84951900	-0.53034500	1.07718100
C	7.12648200	1.10836300	1.38322600
C	6.09395500	0.30633100	0.88642700
C	4.92851100	0.15151900	1.62550900
C	4.82859400	0.69128200	2.91684800
C	5.90234700	1.43362000	3.40843300
C	7.04155900	1.67256400	2.64464700
H	7.84277200	2.29632200	3.03162000
O	3.79766000	0.86297600	-1.07742300
P	2.81923800	0.35110200	0.12891100
O	1.76277700	-0.58494300	-0.36874100
O	2.38623000	1.57655100	0.90357600
C	6.52332600	-0.31808200	-0.42758200
C	5.54993800	-0.70285000	-1.52209600

H	5.82497200	1.86163500	4.40474500
C	8.22762000	1.24977400	0.35889500
H	8.58536800	2.28067700	0.26978200
H	9.09554300	0.63049300	0.62174600
C	7.54685500	0.74283400	-0.92380900
H	6.99598900	1.56116900	-1.40039000
H	8.24700300	0.33470400	-1.65937700
C	7.30325700	-2.32311900	-1.59526300
H	7.31455800	-3.41728500	-1.55668900
H	8.20599100	-2.01180600	-2.13771500
C	7.24097200	-1.68016900	-0.19861100
H	6.61973900	-2.29308500	0.46374400
H	8.22149400	-1.56181900	0.27316900
C	3.59716500	0.48756300	3.75613400
H	3.70586100	0.98076100	4.72624300
H	2.71897000	0.90445700	3.25699500
H	3.41219300	-0.57602800	3.94171500
C	2.34507700	0.03654400	-3.45373000
H	2.24626200	0.02492100	-4.54387700
H	2.22907600	1.06150900	-3.09907900
H	1.52200300	-0.55171600	-3.03391600

Compound: **TS-2**

C	1.52370100	2.35181500	-1.93936100
C	2.04269700	3.58013700	-1.55882800
C	1.27348100	4.71237000	-1.81566100
C	0.01485100	4.61401800	-2.42010200
C	-0.51934500	3.38102500	-2.78463200
C	0.26196400	2.26402600	-2.53808700
N	-0.07506900	0.90820900	-2.75170000
C	0.89321600	0.16151100	-2.31425900
C	1.96113500	0.96452500	-1.74836300
C	2.87159500	0.49657700	-0.91151200
C	3.63344000	0.12682300	0.07195200
H	2.99316400	3.64621400	-1.04039500
H	1.64586900	5.68534900	-1.51228000
H	-0.56536200	5.51554800	-2.59115600
H	-1.50939600	3.28881600	-3.21774600
C	5.04560700	-3.77808000	-1.19077400
C	4.59037400	-2.53979200	-0.82564600
C	5.50180500	-1.49105000	-0.52330000
C	6.89687700	-1.75810700	-0.61159200
C	7.33514800	-3.05018400	-1.00575700
C	6.43332300	-4.04028000	-1.28813800

H	4.33423800	-4.57022200	-1.40399000
H	3.52487600	-2.35499300	-0.74044300
C	5.07320700	-0.20073500	-0.10709200
C	7.81123200	-0.72793100	-0.28890700
H	8.40353200	-3.23929200	-1.07346300
H	6.77661000	-5.02696000	-1.58329800
C	7.38509600	0.51179900	0.11258900
C	5.99882800	0.77975700	0.20687400
H	8.87647400	-0.93180500	-0.35907500
H	8.11143200	1.27935300	0.35268300
C	3.40709700	-4.04374600	2.67656600
C	2.20371000	-3.57750000	2.16042400
C	2.04557900	-2.23029200	1.82425400
C	3.12436000	-1.34679000	2.00260600
C	4.31520700	-1.81597200	2.54751900
C	4.46159500	-3.15908000	2.88220100
H	-0.03516300	-2.43095500	1.16220900
H	3.51857900	-5.09404900	2.92820000
H	1.36889000	-4.25989400	2.02091900
C	0.76673300	-1.71637300	1.33423800
C	3.00339400	0.07288500	1.49260800
H	5.14531300	-1.12869300	2.68697000
H	5.40138600	-3.51428000	3.29322400
C	1.57430600	0.58147300	1.42900000
C	0.52469100	-0.40365800	1.17224300
H	-0.46241200	-0.07435200	0.86572200
O	5.48800500	1.97121600	0.60188300
C	6.36744500	2.99295200	1.02259500
H	7.01808900	3.32467500	0.20415000
H	5.72807100	3.81887100	1.33427800
H	6.98250300	2.66957300	1.87136400
N	1.45891900	1.86106900	1.48799500
N	0.26047400	2.44370800	1.27134900
C	0.22255100	3.81948900	1.27269500
O	1.12216700	4.57405600	1.55694800
O	-1.02005100	4.20669500	0.91603900
C	-1.21789400	5.61072700	0.87514000
H	-1.04370900	6.05832900	1.85727300
H	-0.54628100	6.07589900	0.14847800
H	-2.25641100	5.74865100	0.57247800
C	-5.44282800	-3.35133900	-1.08020300
C	-3.35191100	-2.20290800	-0.81056000
C	-2.72799800	-3.08703400	-1.69797600
C	-3.49676000	-4.11190000	-2.25051000

C	-4.85493000	-4.23807500	-1.96912000
H	-3.02469200	-4.80307800	-2.94429500
H	-5.44402300	-5.01428900	-2.44980500
O	-4.27572200	0.66458200	-1.02039700
C	-5.71525200	0.01935100	2.29182500
C	-5.30411800	-0.18524100	0.97201000
C	-4.81901000	0.88833000	0.24228600
C	-4.82352600	2.18903400	0.75855900
C	-5.30398500	2.36784100	2.05634000
C	-5.72620500	1.29356700	2.83702400
H	-6.04577600	1.45525500	3.86294100
O	-2.61996400	-1.14881400	-0.28529900
P	-2.69642600	0.30036200	-1.03911900
O	-2.38947500	0.12040900	-2.53199100
O	-1.84501700	1.22733400	-0.25055800
C	-5.52823600	-1.63341100	0.57736500
C	-4.68432400	-2.35699900	-0.45680500
H	-5.30881900	3.37073300	2.47549100
C	-6.06101300	-1.29882700	2.94392000
H	-5.65850300	-1.38217900	3.95847000
H	-7.14827200	-1.43375200	3.01729700
C	-5.43609300	-2.31692300	1.97263900
H	-4.37891800	-2.46349600	2.22039000
H	-5.92555100	-3.29569200	1.99082700
C	-6.89023800	-3.24920600	-0.66028300
H	-7.57896300	-3.35901900	-1.50398100
H	-7.14738300	-4.02922200	0.06849100
C	-6.94730800	-1.84867400	-0.02482600
H	-7.11405700	-1.09548800	-0.80260500
H	-7.73801400	-1.74049500	0.72385900
H	-1.25328200	0.50726700	-2.78463500
H	-0.53264900	1.95233500	0.84581200
C	-4.28283500	3.33933500	-0.04828600
H	-4.61994900	3.28531400	-1.08806800
H	-4.61508800	4.29232500	0.37529700
H	-3.18784300	3.31804700	-0.05122500
C	-1.28313900	-2.90333700	-2.08007700
H	-1.20057100	-2.15078300	-2.87224700
H	-0.85597900	-3.84022500	-2.44971100
H	-0.68714200	-2.55851800	-1.22860600
H	3.59988200	0.76275600	2.09782900
H	0.82808200	-0.91926900	-2.30623400

Compound: **INT-3**

C	-1.48868500	2.28986400	1.95069000
C	-1.94987200	3.51377900	1.49236800
C	-1.19950000	4.64516700	1.80769300
C	-0.01539700	4.54495700	2.54553900
C	0.45850900	3.31156500	2.98859000
C	-0.29819800	2.19134800	2.68248200
N	0.01315400	0.83889500	2.97370500
C	-0.92581400	0.11055600	2.45837800
C	-1.93022600	0.90524200	1.75424000
C	-2.81967400	0.44425100	0.89729800
C	-3.57677200	0.09506100	-0.09991600
H	-2.84001600	3.57836500	0.87520400
H	-1.53041700	5.61586300	1.45202900
H	0.55322600	5.44385700	2.76486900
H	1.39189600	3.22212500	3.53488500
C	-5.16629900	-3.72364900	1.22546000
C	-4.65616800	-2.51467600	0.83567000
C	-5.51968900	-1.43770200	0.49293100
C	-6.92582500	-1.64709300	0.56517500
C	-7.42161800	-2.91020300	0.98482000
C	-6.56491400	-3.92767600	1.30731600
H	-4.49080300	-4.53771000	1.47075500
H	-3.58306500	-2.37346900	0.76362900
C	-5.03133100	-0.17642500	0.05427500
C	-7.79318800	-0.59005800	0.20269900
H	-8.49773200	-3.05465000	1.04011200
H	-6.95223000	-4.89167200	1.62255700
C	-7.31068200	0.62114300	-0.22183700
C	-5.91344800	0.83097400	-0.29882600
H	-8.86677300	-0.74941300	0.26068000
H	-8.00115800	1.41152800	-0.49219600
C	-3.27352200	-4.14205400	-2.58985400
C	-2.08060800	-3.64362900	-2.07939500
C	-1.94538100	-2.28552900	-1.77874400
C	-3.03593500	-1.42313000	-1.98640800
C	-4.21589200	-1.92527800	-2.52559500
C	-4.33979200	-3.27907900	-2.82475500
H	0.13458200	-2.43501700	-1.10200500
H	-3.36771900	-5.20033400	-2.81380600
H	-1.23631200	-4.30921900	-1.91679200
C	-0.67727900	-1.73780900	-1.29801200
C	-2.94004200	0.01105700	-1.51296300
H	-5.05543700	-1.25480900	-2.68802400
H	-5.27189700	-3.65938900	-3.23088300

C	-1.52114000	0.54445100	-1.45301800
C	-0.45804600	-0.41784600	-1.16813000
H	0.52079000	-0.06733800	-0.86385000
O	-5.34936300	1.99158200	-0.71284600
C	-6.17906900	3.04259000	-1.15927400
H	-6.82448600	3.41446400	-0.35388800
H	-5.50091000	3.83530800	-1.47534600
H	-6.79804900	2.73294200	-2.01055800
N	-1.42564300	1.82364500	-1.53985600
N	-0.23417500	2.42630900	-1.32344400
C	-0.21874500	3.80269800	-1.31962600
O	-1.12229300	4.54463000	-1.62246700
O	1.01214300	4.20737000	-0.93883000
C	1.17988000	5.61417200	-0.86354700
H	0.97808200	6.08421800	-1.82959900
H	0.51237000	6.04275500	-0.11115900
H	2.22007600	5.76786200	-0.57416900
C	5.39313500	-3.33967100	1.04558700
C	3.31248300	-2.16359700	0.85953200
C	2.70746400	-3.03387200	1.77163100
C	3.48377000	-4.06770700	2.29673100
C	4.82784600	-4.21305600	1.96214900
H	3.02961000	-4.75097500	3.00968600
H	5.42427900	-4.99609200	2.42201900
O	4.29225000	0.69446400	1.00383100
C	5.56498100	-0.00407300	-2.36787500
C	5.21506600	-0.18918200	-1.02784600
C	4.78198900	0.90073200	-0.29090000
C	4.77318900	2.19591300	-0.81830300
C	5.19252800	2.35266000	-2.14040700
C	5.56438300	1.26441300	-2.92678300
H	5.83568300	1.41091200	-3.96860600
O	2.57060800	-1.09331500	0.36653900
P	2.72114200	0.35674900	1.07687700
O	2.55432500	0.17147300	2.62543600
O	1.81427500	1.29710400	0.38942500
C	5.43487000	-1.63622300	-0.62799400
C	4.62518500	-2.33618100	0.44876400
H	5.18768900	3.35011800	-2.57170800
C	5.86234200	-1.33356100	-3.02045000
H	5.41249200	-1.42173200	-4.01428900
H	6.94309000	-1.48404900	-3.14176100
C	5.26857300	-2.33227600	-2.01040000
H	4.19930600	-2.46638200	-2.20834900

H	5.74281700	-3.31801800	-2.04012200
C	6.82272600	-3.26357800	0.56387400
H	7.54463000	-3.37707600	1.37866800
H	7.03629700	-4.05388300	-0.16763900
C	6.87509900	-1.86946900	-0.08628400
H	7.08899300	-1.11253000	0.67616900
H	7.63334200	-1.78136100	-0.87014700
H	1.60596900	0.39554900	2.91889800
H	0.53645800	1.95841400	-0.84063500
C	4.28369300	3.36296800	-0.00280300
H	4.64779300	3.30760800	1.02745000
H	4.62732400	4.30441400	-0.44195700
H	3.18900600	3.37176600	0.02996500
C	1.27784800	-2.83227700	2.19884100
H	1.21984000	-2.08793700	3.00114800
H	0.85050800	-3.76645400	2.57436200
H	0.66054700	-2.47452800	1.36869100
H	-3.54038500	0.67471000	-2.14296100
H	-0.91219800	-0.97241400	2.50721900

Compound: **TS-3**

C	3.62095600	-1.79479800	1.23949700
C	4.91852800	-2.20271500	0.93920200
C	5.41981100	-3.33118500	1.57861800
C	4.63894800	-4.05288600	2.49280900
C	3.33677000	-3.66413500	2.78882900
C	2.83515900	-2.52881100	2.16026500
N	1.56371700	-1.95311400	2.31794700
C	1.55725000	-0.89686700	1.54047400
C	2.76777200	-0.70392900	0.80306500
C	2.73307100	0.13009900	-0.27231900
C	2.46519900	1.31552800	-0.78671700
H	5.51302300	-1.66384500	0.20941000
H	6.43060600	-3.66369300	1.36070400
H	5.05831800	-4.93080500	2.97608400
H	2.72483000	-4.21911800	3.49316200
C	-0.78880500	3.09557900	1.37324700
C	0.25951900	2.51013400	0.71307200
C	1.55649900	3.09510600	0.74124500
C	1.74096500	4.29210100	1.48643800
C	0.63893000	4.86157400	2.17766700
C	-0.60022000	4.28190200	2.12101800
H	-1.77219200	2.63680000	1.33345200
H	0.09304800	1.59587700	0.15304000

C	2.65734900	2.54271200	0.02499600
C	3.02336900	4.88978500	1.50559300
H	0.79789500	5.77251000	2.74955600
H	-1.43935600	4.72510300	2.64820800
C	4.07517300	4.35306000	0.80967600
C	3.88926600	3.17141300	0.05084400
H	3.17011900	5.79947800	2.08212000
H	5.04255200	4.84033500	0.84195300
C	-1.13765600	4.34121400	-2.80826500
C	-1.45175100	2.99736800	-2.95249100
C	-0.47124100	2.01200500	-2.79703900
C	0.84368200	2.38784200	-2.47414300
C	1.16045300	3.74102800	-2.39176900
C	0.17751100	4.71314200	-2.54378700
H	-1.82272800	0.36821500	-3.29800900
H	-1.90948200	5.09632700	-2.91909600
H	-2.46785200	2.69688200	-3.19499600
C	-0.80010100	0.60800400	-3.01660100
C	1.90162200	1.33497900	-2.21949900
H	2.18204300	4.03900600	-2.17702900
H	0.44006400	5.76213000	-2.44936000
C	1.42253900	-0.09098300	-2.40718200
C	0.09345200	-0.38895200	-2.88542200
H	-0.19084600	-1.42365700	-3.03264000
O	4.86650900	2.60637800	-0.70051500
C	6.16916000	3.14416700	-0.64057900
H	6.56599700	3.12373700	0.38177000
H	6.78230300	2.50436000	-1.27579400
H	6.20278200	4.17168600	-1.02509800
N	2.26840200	-0.93206500	-1.91114800
N	1.89728100	-2.24209600	-1.77221500
C	2.92591200	-3.15324600	-1.64172100
O	4.07027800	-2.98246700	-1.98739500
O	2.42474600	-4.28331900	-1.11595100
C	3.38156800	-5.31718100	-0.90333500
H	2.81700800	-6.15349600	-0.49124100
H	3.85654700	-5.60324600	-1.84554500
H	4.14397500	-4.98745700	-0.19444500
C	-4.94802500	0.55917400	2.36038800
C	-2.63656600	0.06901400	1.93135600
C	-2.29107700	0.41689300	3.24412100
C	-3.31830300	0.84106200	4.08915100
C	-4.64398500	0.89815100	3.66850600
H	-3.06812300	1.11086100	5.11155200

H	-5.42651500	1.19575600	4.36090100
O	-2.86332900	-2.56179900	0.53026600
C	-4.27432600	-0.58294300	-2.22256500
C	-3.98509000	-1.01212700	-0.92365400
C	-3.34155000	-2.22428200	-0.74254600
C	-3.07212800	-3.08282300	-1.81464600
C	-3.44006500	-2.65547600	-3.09111100
C	-4.01240000	-1.40220500	-3.30953900
H	-4.23986200	-1.07418600	-4.32045100
O	-1.64492100	-0.36357600	1.05346700
P	-1.39479100	-1.94917000	0.79857200
O	-1.00905400	-2.61416000	2.15978200
O	-0.46536500	-2.07816400	-0.34156300
C	-4.52125200	-0.00879600	0.07901800
C	-3.94039700	0.17669900	1.47001400
H	-3.23665800	-3.30730400	-3.93658800
C	-4.81739800	0.82719300	-2.19639800
H	-4.37835900	1.45916300	-2.97595600
H	-5.90315100	0.84048000	-2.35603300
C	-4.45382200	1.29105700	-0.77302400
H	-3.42479300	1.67090100	-0.75833900
H	-5.11118300	2.07701100	-0.38878900
C	-6.29777400	0.50619500	1.68756300
H	-7.06820600	0.06122400	2.32486600
H	-6.64409500	1.51278000	1.41796800
C	-6.00205300	-0.33692600	0.43739900
H	-6.07078600	-1.40158600	0.68585200
H	-6.68624000	-0.14235000	-0.39377200
H	-0.00174000	-2.49752700	2.35838500
H	1.04535100	-2.40206000	-1.22620600
C	-2.36492300	-4.39320800	-1.59495700
H	-2.76800500	-4.92051300	-0.72545000
H	-2.46744900	-5.03832400	-2.47184200
H	-1.29883800	-4.22613000	-1.41019600
C	-0.86974500	0.35779800	3.73385400
H	-0.46616800	-0.65711600	3.69655900
H	-0.81076700	0.70418500	4.76904200
H	-0.21912500	0.99835400	3.12993900
H	2.74191300	1.52441600	-2.90418400
H	0.69061800	-0.25485700	1.44944300

Compound: **INT-4**

C	-3.52670900	-1.58241500	-0.27876500
C	-4.65969400	-0.75965400	-0.31476900

C	-5.82085900	-1.24789500	-0.88698800
C	-5.88236100	-2.54987600	-1.41622600
C	-4.78804900	-3.39596700	-1.36184800
C	-3.62070100	-2.89744400	-0.78138700
N	-2.40726100	-3.51329900	-0.55179200
C	-1.55366000	-2.64289900	0.05430900
C	-2.18541900	-1.43498900	0.23538100
C	-1.59746500	-0.24605800	0.81781700
C	-1.64174800	1.05391200	0.45112700
H	-4.62828400	0.23116100	0.12360600
H	-6.70496700	-0.61839500	-0.92425400
H	-6.80722500	-2.90203800	-1.86283700
H	-4.83701000	-4.40910600	-1.74959700
C	-0.19729900	0.66113100	-3.61435300
C	-0.67511100	0.73202900	-2.33120800
C	-1.83243500	1.50220000	-2.03340500
C	-2.49711100	2.17045800	-3.10173200
C	-1.98968200	2.05476700	-4.42257700
C	-0.86067700	1.32412300	-4.67324400
H	0.69748400	0.08541100	-3.82173200
H	-0.16736400	0.19567200	-1.53530200
C	-2.32906000	1.65755000	-0.70584600
C	-3.63789800	2.95472200	-2.81824100
H	-2.51198800	2.56592800	-5.22744400
H	-0.46904900	1.24580800	-5.68272100
C	-4.10902800	3.09801600	-1.53912800
C	-3.44254600	2.45005100	-0.47649400
H	-4.14574900	3.45747400	-3.63702100
H	-4.98547400	3.70719300	-1.35376600
C	1.22208500	5.52593600	1.49325400
C	1.37229300	4.61441100	2.52631000
C	0.67816900	3.39722000	2.50964600
C	-0.19536000	3.11344800	1.43741200
C	-0.33915400	4.03191800	0.40243100
C	0.37566300	5.22540000	0.42649600
H	1.59278900	2.69847600	4.35486500
H	1.77053800	6.46201700	1.50915700
H	2.04225000	4.83142400	3.35396500
C	0.90857800	2.41705100	3.55929500
C	-1.02198300	1.86484200	1.55993700
H	-0.99929000	3.81216000	-0.42860100
H	0.27173700	5.92484600	-0.39698400
C	-0.44737100	0.84499400	2.45606300
C	0.39111300	1.16482500	3.55269400

H	0.67054500	0.40338700	4.26928600
O	-3.84793700	2.54985000	0.82310300
C	-5.03961800	3.25041200	1.11370300
H	-5.89640900	2.81456400	0.58641100
H	-5.19010100	3.14624600	2.18845600
H	-4.95406900	4.31418300	0.86059300
N	-0.84703100	-0.33164000	2.04420400
N	-0.60909900	-1.48357400	2.75198600
C	-1.73968700	-2.01243100	3.35052500
O	-2.76442600	-1.39727300	3.54054400
O	-1.49911900	-3.27285200	3.71198200
C	-2.58587100	-3.92460600	4.36686900
H	-2.84979800	-3.40166900	5.28928000
H	-3.45912000	-3.96085500	3.71133900
H	-2.22880900	-4.92999400	4.58610200
C	3.61700400	-0.47332500	-3.36825000
C	1.96517500	-1.49352000	-1.94549900
C	1.51003800	-2.27870200	-3.01607500
C	2.13459900	-2.13056200	-4.25372300
C	3.20095600	-1.25024500	-4.43689100
H	1.79415700	-2.74221800	-5.08545800
H	3.70653400	-1.19227800	-5.39714800
O	3.62735500	-2.06387200	0.51953400
C	3.55249700	1.57768100	0.93615200
C	3.56364600	0.31952900	0.32472900
C	3.66786800	-0.82392600	1.10596700
C	3.85219000	-0.72717600	2.49512800
C	3.91701200	0.54369800	3.06533000
C	3.75602000	1.69942900	2.30085800
H	3.78658300	2.68034500	2.76961900
O	1.41428300	-1.65952500	-0.69268500
P	2.16572000	-2.79546500	0.28155500
O	2.41099200	-4.05956700	-0.44447200
O	1.35321500	-2.72856900	1.54793500
C	3.53305100	0.48736500	-1.18375600
C	2.96976600	-0.55566800	-2.13088800
H	4.08008800	0.62556500	4.13806700
C	3.29514000	2.65795100	-0.08822200
H	2.57057000	3.40125300	0.25554700
H	4.21776800	3.20088400	-0.33328100
C	2.78586500	1.84764600	-1.29250000
H	1.70909800	1.67287500	-1.18964200
H	2.94961900	2.34172100	-2.25530000
C	4.78355100	0.48297900	-3.28745900

H	5.67753500	0.09362700	-3.78505400
H	4.54620700	1.44623900	-3.75896200
C	4.96914200	0.63570100	-1.76735500
H	5.58404800	-0.18651600	-1.38575900
H	5.44587800	1.57734100	-1.47751800
H	-2.15339200	-4.44518200	-0.83683200
H	0.15393900	-2.07749000	2.33359500
C	3.97944200	-1.97764200	3.32218700
H	4.78714600	-2.61346000	2.94500000
H	4.18854800	-1.73440500	4.36856900
H	3.05837500	-2.56550500	3.26579900
C	0.36869900	-3.23766000	-2.82139700
H	0.62807700	-3.97493100	-2.05520300
H	0.14010800	-3.76083600	-3.75478500
H	-0.53141200	-2.70128500	-2.49955700
H	-1.91852900	2.18849500	2.13558500
H	-0.54659000	-2.93451800	0.31592000

Compound: **INT-5**

C	-2.33265000	3.24218400	0.04063000
C	-1.02207100	3.30925000	-0.44970400
C	-0.61850700	4.47934200	-1.06526200
C	-1.49718200	5.57508200	-1.19077300
C	-2.80061200	5.52110400	-0.72348900
C	-3.20852500	4.33147500	-0.11159200
N	-4.42800700	3.95525800	0.42200100
C	-4.34499200	2.67382200	0.90223300
C	-3.07751800	2.19642900	0.69726200
C	-2.51398200	0.89839600	1.04070700
C	-2.61369500	-0.31073300	0.46183700
H	-0.35820100	2.45120000	-0.38579100
H	0.38418800	4.52705400	-1.47866700
H	-1.15028600	6.48032000	-1.68062100
H	-3.47693100	6.36316000	-0.83810800
C	-6.30992600	-2.53613100	0.63674400
C	-5.14157700	-1.82153800	0.58610700
C	-4.64537100	-1.32117500	-0.64948700
C	-5.39669700	-1.57246900	-1.83158800
C	-6.60374800	-2.31328200	-1.74436400
C	-7.05376400	-2.78789400	-0.54035100
H	-6.66999800	-2.91381100	1.58928100
H	-4.57616500	-1.63353000	1.49445000
C	-3.43016200	-0.58924900	-0.73627300
C	-4.91334500	-1.06713500	-3.06354800

H	-7.16765300	-2.49774400	-2.65543800
H	-7.97789100	-3.35472600	-0.48445200
C	-3.75402600	-0.34210100	-3.13531900
C	-3.00257700	-0.08743600	-1.96002100
H	-5.48364100	-1.26005000	-3.96861100
H	-3.40977100	0.04032800	-4.08900000
C	-1.18295200	-5.39690200	1.65075300
C	-0.45834900	-4.50731700	2.42546900
C	-0.60069500	-3.12422000	2.24627000
C	-1.48025200	-2.64108600	1.25564200
C	-2.18098300	-3.54321100	0.45934600
C	-2.04706400	-4.91025500	0.66879000
H	0.86440100	-2.63504700	3.77556000
H	-1.07240600	-6.46604100	1.80066600
H	0.23198200	-4.87468200	3.17991700
C	0.14888600	-2.20212000	3.08155300
C	-1.50646600	-1.16199500	1.02996700
H	-2.83568600	-3.17790700	-0.32262800
H	-2.61713900	-5.60153400	0.05581600
C	-0.97042800	-0.35513800	2.14112100
C	-0.02033600	-0.85817500	3.06754900
H	0.52686000	-0.18545200	3.71389700
O	-1.86612000	0.61959600	-1.94266900
C	-1.29305300	1.11120800	-3.14349200
H	-1.94531900	1.86122800	-3.60654300
H	-0.34569600	1.56571200	-2.84596000
H	-1.10011200	0.29236500	-3.84675600
N	-1.52961200	0.83164000	2.07596900
N	-1.25412700	1.91408300	2.89401400
C	-0.04971700	2.60815600	2.62150700
O	0.98914700	2.07503200	2.35129500
O	-0.27065900	3.90923400	2.79932700
C	0.84897100	4.75167500	2.49921100
H	1.71636300	4.46279000	3.09614500
H	1.08685300	4.67394700	1.43605900
H	0.52396400	5.76079400	2.74722700
C	3.42988800	-3.31016700	0.24322000
C	2.32498000	-1.74266800	-1.21509700
C	1.22610600	-2.61040300	-1.32551500
C	1.26316200	-3.81988200	-0.63392500
C	2.34221500	-4.16587500	0.17834400
H	0.40902900	-4.48830600	-0.70352900
H	2.32265000	-5.09064900	0.74989000
O	2.90964700	0.79809100	0.29281300

C	6.29601900	0.30666100	-0.99547100
C	5.02736100	0.03136900	-0.48162200
C	4.19158600	1.07465500	-0.11457000
C	4.64152100	2.40121800	-0.15017800
C	5.93549600	2.64302400	-0.61156800
C	6.76117600	1.61119900	-1.05793200
H	7.74798000	1.83207400	-1.45660200
O	2.27073500	-0.50815400	-1.81509400
P	1.72734900	0.74611600	-0.86152200
O	0.50517500	0.30533600	-0.11578400
O	1.72278900	1.96072900	-1.72407700
C	4.81442600	-1.46529800	-0.37768800
C	3.44952900	-2.12098100	-0.49255100
H	6.29177700	3.66990300	-0.65103500
C	6.96291100	-0.97104400	-1.45072800
H	7.45796700	-0.86477700	-2.42170000
H	7.72886300	-1.29776900	-0.73412200
C	5.78176400	-1.95910800	-1.49317300
H	5.26754100	-1.87828500	-2.45708100
H	6.08093200	-3.00363300	-1.35697800
C	4.69235700	-3.44076000	1.06386700
H	4.49538900	-3.77539100	2.08843300
H	5.38198300	-4.16972200	0.61736100
C	5.27136700	-2.01574200	1.00362200
H	4.81999900	-1.39761900	1.78770800
H	6.35834000	-1.97996500	1.12902300
H	-5.26085000	4.51987400	0.42885800
H	-2.06053900	2.53221800	2.89446600
C	3.72059900	3.51264800	0.27535300
H	3.22241000	3.25777300	1.21640300
H	4.27052100	4.45132400	0.39923400
H	2.93481500	3.64740600	-0.47557100
C	0.04244900	-2.22213600	-2.17096600
H	-0.37005100	-1.26507900	-1.84086800
H	-0.74018200	-2.98598400	-2.12132700
H	0.33567400	-2.09839000	-3.21998400
H	-0.76312400	-0.96799200	0.34388700
H	-5.20186200	2.18548800	1.34441100

Compound: **INT-6**

C	-2.31774400	3.26526400	-0.01655500
C	-1.00920300	3.41612700	-0.49528700
C	-0.64629300	4.61938100	-1.07235000
C	-1.56925800	5.67750600	-1.18828200

C	-2.87539600	5.54509200	-0.74762000
C	-3.23672000	4.32429000	-0.16981600
N	-4.44929700	3.89157100	0.32484600
C	-4.32030900	2.60279600	0.77938400
C	-3.03119800	2.17601100	0.60578200
C	-2.50740500	0.86865900	0.99550700
C	-2.69089200	-0.37136600	0.44333000
H	-0.30147200	2.59913600	-0.44982800
H	0.36075900	4.72994600	-1.46230900
H	-1.25644200	6.61028600	-1.64835600
H	-3.59227400	6.35395000	-0.85614100
C	-6.50337000	-2.39364500	0.36862700
C	-5.29275900	-1.75249700	0.39109500
C	-4.71676000	-1.23574400	-0.80339000
C	-5.43544800	-1.39682000	-2.02148600
C	-6.68771300	-2.06562000	-2.01065000
C	-7.21358700	-2.55557900	-0.84497000
H	-6.92236600	-2.78549100	1.29100400
H	-4.75037000	-1.63978200	1.32448200
C	-3.45458200	-0.57688100	-0.80626600
C	-4.87727500	-0.87492800	-3.21201400
H	-7.22325800	-2.18151900	-2.94993500
H	-8.17103900	-3.06775700	-0.84693500
C	-3.67355400	-0.22141700	-3.20634200
C	-2.95479400	-0.06231900	-1.99535700
H	-5.42118200	-0.99516800	-4.14553900
H	-3.27307400	0.17376100	-4.13237200
C	-1.37100600	-5.52576300	1.26396300
C	-0.79570300	-4.73432500	2.22866900
C	-0.98086200	-3.33139500	2.23235100
C	-1.77621400	-2.73674400	1.21089500
C	-2.33986700	-3.57255100	0.21842400
C	-2.14689200	-4.93432800	0.24708200
H	0.22897100	-3.01612400	4.00344200
H	-1.22486400	-6.60177100	1.27938700
H	-0.18735600	-5.17992000	3.01208700
C	-0.38422500	-2.52483100	3.25317500
C	-1.97186400	-1.31742900	1.26191100
H	-2.93163600	-3.12963900	-0.57370100
H	-2.59670900	-5.55678700	-0.52088900
C	-1.37534700	-0.59729900	2.29939100
C	-0.56825300	-1.17433700	3.30087400
H	-0.11562100	-0.55684400	4.06949300
O	-1.76778800	0.57811500	-1.92599400

C	-1.19157600	1.11881300	-3.09806400
H	-1.82888000	1.90170600	-3.52731000
H	-0.24138000	1.55405100	-2.78605100
H	-1.00582600	0.33792300	-3.84632500
N	-1.69643400	0.72826400	2.11595300
N	-1.39077600	1.74704200	2.98254000
C	-0.31095800	2.54297100	2.66741400
O	0.62328900	2.19143200	1.97238100
O	-0.40566400	3.72010900	3.27044200
C	0.63242400	4.65239800	2.93906200
H	1.60638700	4.26038900	3.23838700
H	0.62348200	4.84528500	1.86389100
H	0.39227500	5.55620200	3.49656500
C	3.63627300	-3.28238800	0.32718300
C	2.45869900	-1.73708400	-1.07197100
C	1.33945700	-2.57377100	-1.10947700
C	1.40659500	-3.77415600	-0.40381800
C	2.53286200	-4.12110700	0.33887500
H	0.53773600	-4.42505000	-0.40162100
H	2.53550200	-5.03540200	0.92638600
O	3.15101200	0.84945100	0.36979000
C	6.47160400	0.30388600	-1.07236800
C	5.22683900	0.04386000	-0.49465400
C	4.43993800	1.10759600	-0.08648300
C	4.89241100	2.42899900	-0.14553800
C	6.16741000	2.64867000	-0.66891900
C	6.95091900	1.60256000	-1.15428500
H	7.91997600	1.80838000	-1.60078700
O	2.36441100	-0.48627700	-1.68772800
P	1.99462400	0.77833400	-0.76562300
O	0.77657600	0.27766200	0.10159500
O	1.83352000	1.99498400	-1.58357300
C	5.00090700	-1.45238500	-0.38219200
C	3.62549100	-2.09852600	-0.41746200
H	6.53774100	3.66897600	-0.72817900
C	7.09589300	-0.98045100	-1.56611300
H	7.53987500	-0.87527600	-2.56115300
H	7.89430600	-1.31725200	-0.89169800
C	5.90188800	-1.95348700	-1.54879000
H	5.34018600	-1.86716900	-2.48535500
H	6.19458900	-3.00095900	-1.42765400
C	4.94357400	-3.42296000	1.07105800
H	4.80338200	-3.75051100	2.10629300
H	5.59890500	-4.15951000	0.58735100

C	5.53092200	-2.00398100	0.97313600
H	5.13223200	-1.38169000	1.78195200
H	6.62341600	-1.97707200	1.03447600
H	-5.30801600	4.41521700	0.30425800
H	-2.19146600	2.22140200	3.38139500
C	4.00627600	3.55962600	0.30582300
H	3.50597700	3.31497900	1.24816800
H	4.58742600	4.47676000	0.43981100
H	3.21999500	3.74243300	-0.43376200
C	0.10010300	-2.16290000	-1.85549800
H	-0.42269000	-1.35921800	-1.32795800
H	-0.58404100	-3.00905700	-1.95544300
H	0.34714400	-1.78999400	-2.85517500
H	0.61796300	0.86174800	0.88191500
H	-5.16566900	2.06653500	1.18743600

Compound: **15**

C	2.825303000	-0.937186000	-0.083296000
C	3.453488000	-0.292147000	0.991949000
C	4.667085000	-0.782288000	1.443451000
C	5.264033000	-1.911673000	0.850226000
C	4.654321000	-2.578156000	-0.198748000
C	3.428894000	-2.080416000	-0.650597000
N	2.586547000	-2.543511000	-1.637684000
C	1.480135000	-1.733714000	-1.706111000
C	1.580820000	-0.727793000	-0.783542000
C	0.553617000	0.274299000	-0.516211000
C	-0.726810000	0.101197000	-0.050932000
H	2.989281000	0.570927000	1.457454000
H	5.164790000	-0.294304000	2.276442000
H	6.216309000	-2.272084000	1.227715000
H	5.108028000	-3.456875000	-0.648008000
C	-3.901783000	-1.712566000	-2.339444000
C	-2.904070000	-1.131979000	-1.601227000
C	-2.314525000	-1.816245000	-0.501154000
C	-2.781429000	-3.126640000	-0.198097000
C	-3.818273000	-3.699142000	-0.981104000
C	-4.370482000	-3.010939000	-2.028153000
H	-4.341280000	-1.169026000	-3.170760000
H	-2.557669000	-0.132961000	-1.843907000
C	-1.275152000	-1.231475000	0.282036000
C	-2.184154000	-3.829900000	0.874134000
H	-4.165596000	-4.699154000	-0.732326000
H	-5.164709000	-3.455808000	-2.620080000

C	-1.174100000	-3.271868000	1.611270000
C	-0.713362000	-1.964842000	1.316783000
H	-2.536560000	-4.830892000	1.109894000
H	-0.734079000	-3.832330000	2.428083000
C	-5.094559000	2.960822000	1.239807000
C	-4.120916000	3.791933000	0.740605000
C	-2.862401000	3.286279000	0.337421000
C	-2.614419000	1.887658000	0.447715000
C	-3.629145000	1.056798000	0.976973000
C	-4.840967000	1.580856000	1.362009000
H	-2.077654000	5.228706000	-0.223970000
H	-6.054990000	3.365397000	1.545400000
H	-4.304983000	4.860077000	0.651769000
C	-1.849475000	4.168096000	-0.161508000
C	-1.334309000	1.405027000	0.019049000
H	-3.442211000	-0.005258000	1.085236000
H	-5.606699000	0.924649000	1.765375000
C	-0.387149000	2.324692000	-0.438996000
C	-0.620405000	3.712188000	-0.537883000
H	0.157657000	4.376649000	-0.897827000
O	0.278032000	-1.368945000	2.023242000
C	1.122264000	-2.172493000	2.816685000
H	1.536918000	-3.007549000	2.238128000
H	1.940130000	-1.523590000	3.131111000
H	0.604309000	-2.558219000	3.704450000
N	0.755265000	1.626062000	-0.744619000
N	1.873244000	2.170073000	-1.321282000
C	2.913088000	2.563203000	-0.494518000
O	2.808654000	2.828708000	0.677275000
O	4.034822000	2.647026000	-1.223561000
C	5.200715000	2.995994000	-0.477966000
H	5.059132000	3.948658000	0.036985000
H	5.423128000	2.214655000	0.253382000
H	6.003187000	3.070687000	-1.211036000
H	2.744024000	-3.358838000	-2.205464000
H	2.130463000	1.769485000	-2.213838000
H	0.676343000	-1.941168000	-2.398536000

Compound: CPA

C	2.26785900	1.72176200	0.66924200
C	1.71234400	-0.28700300	-0.51405300
C	3.05542300	-0.67732400	-0.53961700
C	3.98903800	0.17442600	0.05157000
C	3.60463200	1.35607800	0.68092000

H	5.03662900	-0.11491200	0.04368600
H	4.34476200	1.97722600	1.17764000
O	-0.74572900	-1.16521100	1.02058600
C	-2.31593700	1.69828000	-0.66060600
C	-1.35426500	0.91252000	-0.01939900
C	-1.72925000	-0.31443800	0.50044100
C	-3.05960600	-0.74577600	0.49557200
C	-4.00486600	0.09529500	-0.09327800
C	-3.64289100	1.29984600	-0.69309500
H	-4.39265600	1.90964000	-1.18948300
O	0.75941000	-1.16062400	-1.04263100
P	-0.04291300	-2.14125400	-0.04963300
O	1.05615500	-2.79945400	0.91595000
O	-0.88573000	-3.09322600	-0.78512200
C	-0.02414800	1.63955200	0.00887000
C	1.31533200	0.92623600	0.02507400
H	-5.04372200	-0.22304000	-0.11178700
C	-1.66953600	2.91474000	-1.28032500
H	-2.03475500	3.11195600	-2.29299600
H	-1.86767800	3.81599700	-0.68553900
C	-0.17610200	2.54165300	-1.25097200
H	0.07544900	1.95009100	-2.13805300
H	0.49022700	3.40913700	-1.22488600
C	1.60615100	2.91874700	1.31036200
H	1.97304600	3.10491400	2.32458200
H	1.79049100	3.83162500	0.72913800
C	0.11828600	2.52609500	1.27991700
H	-0.12381100	1.91959600	2.15952500
H	-0.55969800	3.38471600	1.26620200
C	-3.44027900	-2.08160500	1.07569100
H	-3.00488700	-2.21848700	2.07065200
H	-4.52700400	-2.16894200	1.15853400
H	-3.07199400	-2.89410500	0.44128900
C	3.46815900	-1.98436200	-1.16239600
H	3.24992800	-2.82085700	-0.49023600
H	4.54167700	-1.99001200	-1.37002400
H	2.93199200	-2.16695200	-2.09802200
H	1.04077700	-3.75899000	0.80050700

References

1 Hashimoto, T., Hirose, D. & Taniguchi, T. Catalytic Aerobic Oxidation of Arylhydrazides with Iron Phthalocyanine. *Adv. Synth. Catal.* **357**, 3346-3352 (2015).

2 Wang, C. *et al.* Phosphine-Catalyzed Asymmetric Cycloaddition Reaction of Diazenes: Enantioselective Synthesis of Chiral Dihydropyrazoles. *Org. Lett.* **21**, 7519-7523 (2019).

3 Yuan, H. *et al.* Asymmetric synthesis of atropisomeric pyrazole via an enantioselective reaction of azonaphthalene with pyrazolone. *Chem. Commun.* **55**, 12715-12718 (2019).

4 Ueda, H., Yamaguchi, M., Kameya, H., Sugimoto, K. & Tokuyama, H. Autotandem Catalysis: Synthesis of Pyrroles by Gold-Catalyzed Cascade Reaction. *Org. Lett.* **16**, 4948-4951 (2014).

5 Saito, H., Otsuka, S., Nogi, K. & Yorimitsu, H. Nickel-Catalyzed Boron Insertion into the C2–O Bond of Benzofurans. *J. Am. Chem. Soc.* **138**, 15315-15318 (2016).

6 Okamura, T., Fujiki, S., Iwabuchi, Y. & Kanoh, N. Gold(i)-catalyzed Nicholas reaction with aromatic molecules utilizing a bifunctional propargyl dicobalt hexacarbonyl complex. *Org. Biomol. Chem.* **17**, 8522-8526 (2019).

7 Xu, T. *et al.* Yb(OTf)3-Catalyzed Alkyne–Carbonyl Metathesis–Oxa-Michael Addition Relay for Diastereoselective Synthesis of Functionalized Naphtho[2,1-b]furans. *Org. Lett.* **22**, 2414-2418 (2020).

8 Yan, S. *et al.* Michael Reaction Inspired Atroposelective Construction of Axially Chiral Biaryls. *J. Am. Chem. Soc.* **142**, 7322-7327 (2020).

9 Li, H. *et al.* Rhodium(III)-Catalyzed Oxidative [3 + 2] Annulation of 2-Acetyl-1-arylhydrazines with Maleimides: Synthesis of Pyrrolo[3,4-b]indole-1,3-diones. *Org. Lett.* **21**, 8563-8567 (2019).

10 Yao, L. *et al.* Chiral Ferrocenyl N,N Ligands with Intramolecular Hydrogen Bonds for Highly Enantioselective Allylic Alkylations. *ChemCatChem* **10**, 804-809 (2018).

11 Frisch, M. J. et al. Gaussian 09, Revision D.01, Gaussian, Inc., Wallingford CT, 2016.

12 Chai, J.-D. & Head-Gordon, M. Long-range corrected hybrid density functionals with damped atom-atom dispersion corrections. *Phys. Chem. Chem. Phys.* **10**, 6615–6620 (2008).

13 Ditchfield, R., Hehre W. J. & Pople, J. A. Self-consistent molecular-orbital methods. IX. An extended Gaussian-type basis for molecular-orbital studies of organic molecules. *J. Chem. Phys.* **54**, 724-728 (1971).

14 Hehre, W. J., Ditchfield R. & Pople, J. A. Self-consistent molecular orbital methods. XII. Further extensions of Gaussian-type basis sets for use in molecular orbital studies of organic molecules. *J. Chem. Phys.* **54**, 2257-2261 (1971).

15 Hariharan, P. C., & Pople, J. A. The influence of polarization functions on molecular orbital hydrogenation energies. *Theor. Chim. Acta* **28**, 213-222 (1973).

16 Raghavachari, K., Binkley, J. S., Seeger, R. & Pople, J. A. Self-consistent molecular orbital methods. 20. Basis set for correlated wave-functions. *J. Chem. Phys.*, **72**, 650-654 (1980).

17 McLean, A. D. & Chandler, G. S. Contracted Gaussian-basis sets for molecular calculations. 1. 2nd row atoms, Z=11-18, *J. Chem. Phys.*, **72**, 5639-5648 (1980).

18 Frisch, M. J., Pople, J. A. & Binkley, J. S. Self-Consistent Molecular Orbital Methods. 25. Supplementary Functions for Gaussian Basis Sets *J. Chem. Phys.*, **80**, 3265-3269 (1984).

19 Legault, C. Y. CYLView, 1.0b, Université de Sherbrook, 2009; www.cylview.org.
